# Enantioselective copper-catalyzed B–H bond insertion reaction of α-diazo phosphonates to access chiral α-boryl phosphonates†

**DOI:** 10.1039/d4sc01271b

**Published:** 2024-04-10

**Authors:** Longlong Li, Kui Yu, Hejun An, Xinping Cai, Qiuling Song

**Affiliations:** a Key Laboratory of Molecule Synthesis and Function Discovery, Fujian Province University, College of Chemistry and College of Materials Science at Fuzhou University Fuzhou Fujian 350108 China qsong@fzu.edu.cn; b School of Chemistry and Chemical Engineering, Henan Normal University Xinxiang Henan 453007 China; c State Key Laboratory of Organometallic Chemistry, Shanghai Institute of Organic Chemistry, Chinese Academy of Sciences Shanghai 200032 China

## Abstract

Chiral phosphorus-containing compounds find applications across various fields, including asymmetric catalysis, medicinal chemistry, and materials science. Despite the abundance of reported highly enantioselective methods for synthesizing various chiral phosphorus compounds, the enantioselective synthesis of α-boryl phosphorus compounds still remains an unknown territory. Here, we report a method for the construction of chiral α-boryl phosphates by asymmetric B–H insertion reaction using α-diazo phosphates as carbene precursors, cheap and readily available copper salt as the catalyst and chiral oxazoline as the ligand. This method can directly afford a series of stable α-boryl phosphates with a yield up to 97% and an enantioselectivity up to 98% ee. The operating procedure of this method is straightforward, offering a broad substrate applicability, remarkable tolerance towards various functional groups, and gentle reaction conditions.

## Abstract 2

Chiral phosphorus-containing compounds find applications across various fields, including asymmetric catalysis, medicinal chemistry, and materials science.

## Graphical abstract

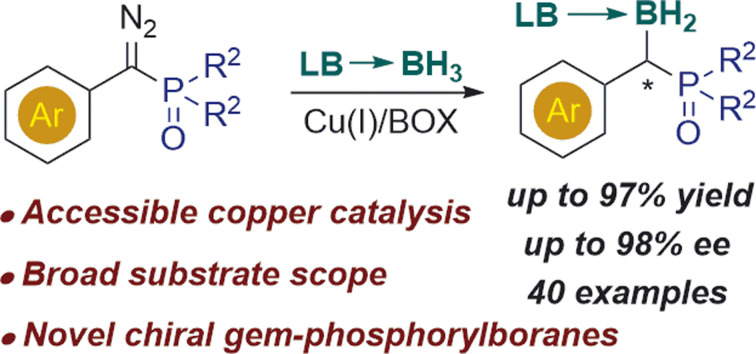

## Introduction

**Scheme 1:**
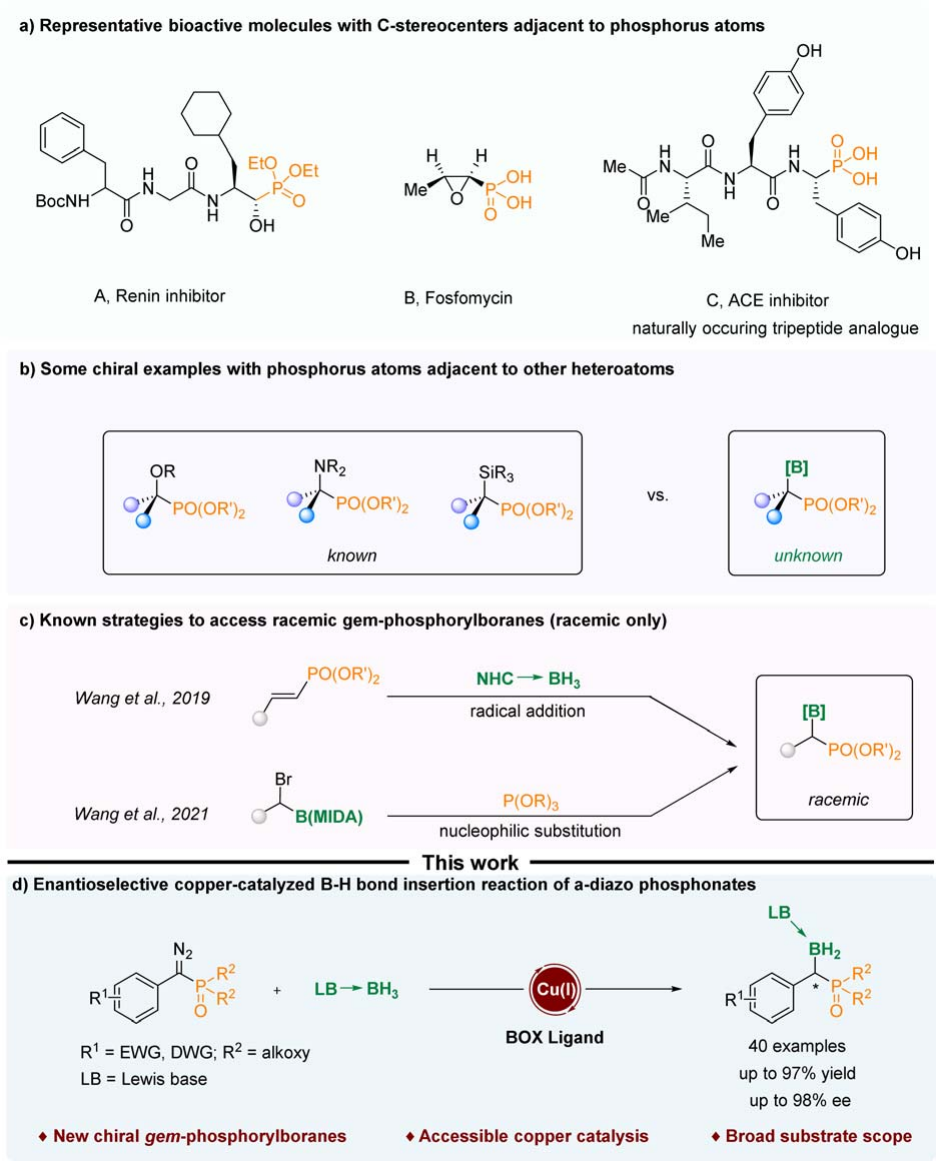
(a) Representative bioactive molecules with C-stereocenters adjacent to phosphorus atoms. (b) Some examples of phosphorus atoms adjacent to other heteroatoms. (c) Known strategies to access *gem*-phosphorylboranes (racemic only). (d) Enantioselective copper-catalyzed B–H bond insertion reaction of α-diazo phosphonates.

Organophosphorus compounds with C-stereocenters adjacent to phosphorus atoms have been widely used in various fields. For instance, they can act as active ingredients in drugs for the treatment of diseases and as ligands in metal catalytic reactions (Scheme 1a).^1–7^ In the past few decades, various compounds containing phosphorus atoms and other non-phosphorus heteroatoms attached to the same chiral carbon atom have been reported, such as chiral α-hydroxy phosphonates,^8–12^ chiral α-amino phos-phonates^13,14^ and chiral α-silyl phosphonates.^15^ However, the construction of chiral α-boryl phosphonates has not been reported (Scheme 1b). One of the difficulties might be that α-boryl phosphates are thermodynamically unstable, and due to the electron-withdrawing properties of phosphoryl groups, they are prone to undergo a simple deboronation reaction.^16–18^ In 2019, Wang and Zhou realized the free radical addition reaction of NHC-boranes to styrylphosphates.^16^ In 2021, Wang treated α-Br alkyl MIDA boronates with triethylphosphites to generate α-boryl phosphonates in good yields (Scheme 1c).^17^ The boryl moieties are tetracoordinate boron species, which increase the stability of the final α-boryl phosphonates. Despite these advances, the current strategies are confined to the construction of racemic α-boryl phosphates. As a result, there is a need to devise new methods for the assembly of chiral α-boryl phosphates.

Implementing asymmetric carbene insertion into the B–H bond through transition metal catalysis emerges as a potent strategy for crafting chiral boron-containing compounds.^19–27^ Therefore, we hypothesized that α-diazo phosphonates might serve as suitable precursors for α-phosphoryl carbene,^28,29^ which could undergo enantioselective B–H insertion to afford chiral α-boryl phosphates.

To accomplish this transformation, it is essential to tackle challenges in at least two aspects: (1) the α-phosphoryl carbene has higher steric hindrance than its ester counterpart.^30^ (2) We needed to achieve efficient chiral induction to control the enantioselectivity of the B–H bond insertion.^31^

Recently, our group has realized the asymmetric B–H bond insertion reaction of α-diazo phenylacetate^32^ and ene-yne-ketones^33^ with tetracoordinate boranes under copper catalysis. Herein, we report a copper-catalyzed asymmetric B–H insertion reaction of α-phosphoryl carbenes generated from α-diazo phosphates, enabling the rapid synthesis of chiral α-boryl phosphonates with high enantioselectivities and excellent yields (Scheme 1d). The resulting compounds represent a new class of chiral boron compounds as well as chiral phosphorus compounds characterized by the presence of a tetrahedrally coordinated boron moiety and a phosphonate species.^34^

## Results and discussion

To validate the conjecture, we first studied the reaction of diethyl(diazo(phenyl)methyl)phosphonate 1a and methyldiphenylphosphane borane 2a in the presence of Cu(MeCN)_4_PF_6_ in DCM at 20 °C, and the α-phosphoryl organoboron 3aa could not be obtained (Table 1, entry 1), even when phosphoramidite L1 was added as a ligand (entry 2). Surprisingly, when we added the phosphine ligand L2 containing the oxazoline skeleton, the reaction could lead to 42% of the desired compound 3aa, although the ee value was only 2% (entry 3). We further evaluated a series of ligands L3–L6, and found that when the carbon atom in the middle of the oxazoline scaffolds increased the steric hindrance, the enantiomeric excess (ee) values were increased accordingly (entries 4–7). Subsequently, we further examined L7, L8, L9, L10 as ligands, and found that when L10 was used as the ligand, the yield was increased to 80% with an 88% ee value (entries 8–11). Furthermore, DCM, PhCl, THF, MTBE and CPME were assessed with L10 as the ligand (entries 12–16). It was pleasing to note that when the solvent used was changed to CPME, the reaction afforded the desired α-boryl phosphates 3aa in 86% yield and 92% ee (entry 16). And it was considered as the optimal reaction condition. The absolute configuration of 3aa was unambiguously determined by X-ray analysis (Cambridge Crystallographic Data Centre [CCDC] 2312601).

**Table 1:** Optimization of the reaction conditions^a^

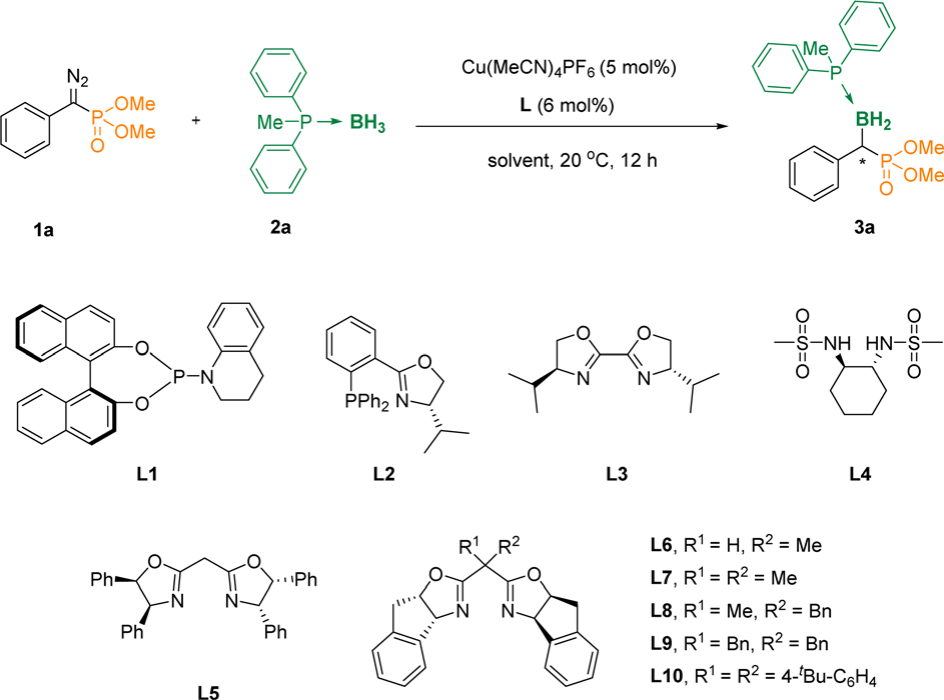				
Entry^a^	Ligand	Solvent	Yield (%)	ee (%)
1	None	DCM	Trace	—
2	L1	DCM	Trace	—
3	L2	DCM	42	2
4	L3	DCM	Trace	—
5	L4	DCM	Trace	—
6	L5	DCM	75	47
7	L6	DCM	83	73
8	L7	DCM	90	77
9	L8	DCM	75	84
10	L9	DCM	80	88
11	L10	DCM	82	88
12	L10	DCE	76	90
13	L10	PhCl	80	91
14	L10	THF	69	90
15	L10	MTBE	81	92
16	L10	CPME	86	92

a Reaction conditions: 1a/2a/Cu(MeCN)_4_PF_6_/L = 0.2/0.4/0.01/0.012 (mmol), in 2 mL of solvent. Isolated yields were given. The ee values were determined by HPLC.

### Substrate scope

With the optimal conditions accessible, we then evaluated the suitability of substrates containing different substituents. In the beginning, we try to modify the benzene ring in the *para*-position of the aryl diazo phosphate esters, and all of them led to products with fine enantioselectivity (3aa–3na). Among them, when it was an electron-withdrawing functional group, the ee value of the product was usually higher, but the yield was relatively lower. For example, the ee value of *para*-nitro substitution could reach 96%, while the yield was only 70% (3ga). An inverse trend becomes apparent in instances where an electron-donating functional group occupies the *para* position, exemplified by the *para*-benzyloxy substitution showcasing an ee of 90% alongside a yield of 85% (3ma). Sulfur is known to have a toxic effect on metals, and under our standard conditions, the product with a thiomethyl substitution was also able to obtain a 92% ee value, albeit in 44% yield. In the meta position, favorable yields and high ee were observed when the substituent was either electron-donating (3oa) or electron-withdrawing (3pa) groups. However, for the *ortho*-substitution (methoxy, carboxylic ester group and fluorine atoms), only the fluorine-substituted substrate could obtain the corresponding product 3ra through the reaction. This could be attributed to the large spatial hindrance introduced by the *ortho* substituents, resulting in difficulties in the progression of the reaction. Multi-substituted substrates at different positions could also be well compatible with the reaction system (3sa–3ua).

**Scheme 2:**
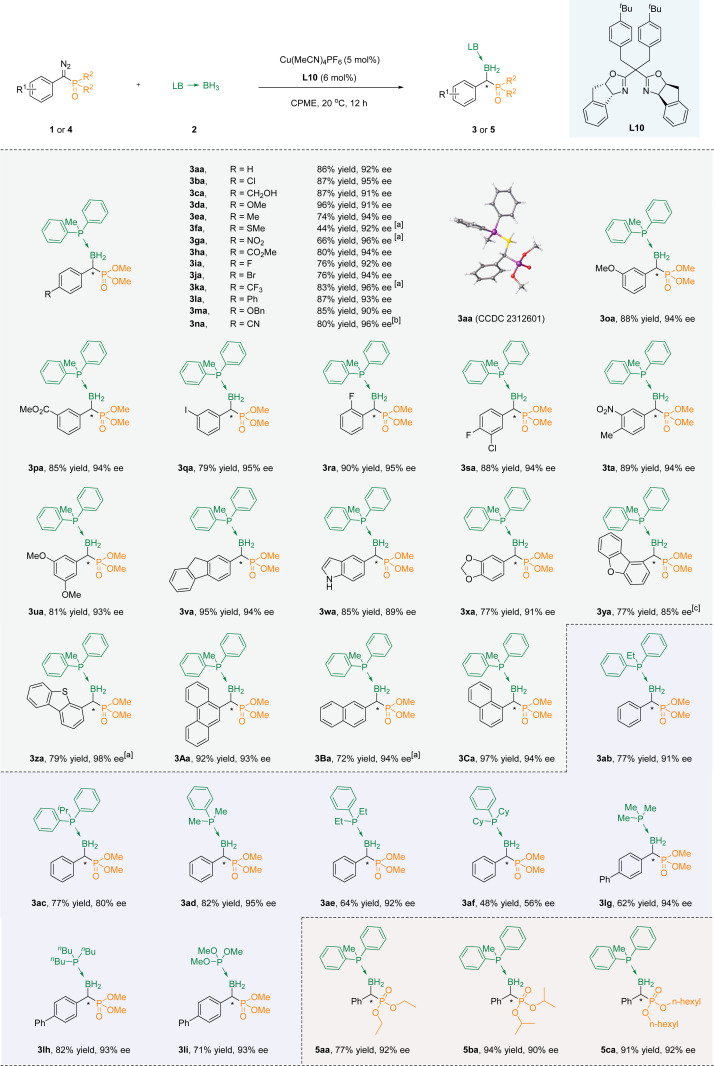
Substrate scope of copper-catalyzed asymmetric B–H bond insertion reactions of α-diazo phosphonates and phosphine–borane adducts. Reaction conditions: 1 (0.2 mmol), 2a (0.4 mmol), Cu(MeCN)_4_PF_6_ (5 mol%), L10 (6 mol%) in 2 mL of CPME. Isolated yields were given, ee values were determined by HPLC. ^[a]^ Reaction time: 36 h. ^[b]^ Reaction time: 48 h. ^[c]^ Reaction conditions: 1 (0.2 mmol), 2a (0.4 mmol), Cu(MeCN)_4_PF_6_ (10 mol%), L10 (12 mol%) in 2 mL of CPME.

Satisfactory yields and ee values could be achieved by converting the benzene ring into fused and heterocyclic frameworks. Surprisingly, indole without a protective group on the nitrogen atom was also compatible with the reaction conditions to achieve a good yield, although the ee value was only 89% (3wa). When fluorene (3va), 1,2-methylenedioxybenzene (3xa), dibenzofuran (3ya), dibenzothiophene(3za), anthracene (3Aa), and naphthalene (3Ba, 3Ca) were used as aromatic skeletons, the corresponding products could be obtained under the standard conditions, with excellent yields (up to 97%) and high ee values (up to 98%) (Scheme 2).

Next, the reaction of a series of aryl phosphoborane adducts 2b–2f with phenyl diazo phosphate (1a, 1j, 1l) was evaluated. The B–H bond insertion reaction was successfully carried out to obtain the target products, but the ee value decreased with the increase of the steric hindrance of the substituents on the phosphorus of phosphoboranes (3ab–3af). It is noteworthy that when triphenylphosphine borane was used as a reactant, the product exhibited significant tailing, making it difficult to separate effectively. At the same time, due to poor color intensity (254 nm) when alkyl phosphine boranes are used as reactants, dimethyl([1,1′-biphenyl]-yl(diazo)methyl)phosphonate 1l was tested as the reaction substrate with alkyl phosphine boranes 2g–2f and trimethyl phosphite borane 2i as the reaction substrate. The corresponding products could be acquired successfully with a high level of optical purity (3lg–3li). Last but not the least, the phosphorus-linked alkoxy groups of α-diazo phosphonates were extended to obtain the corresponding products (5aa–5ca) with high yields and good enantioselectivities.

### Exploration of synthetic applications

**Scheme 3:**
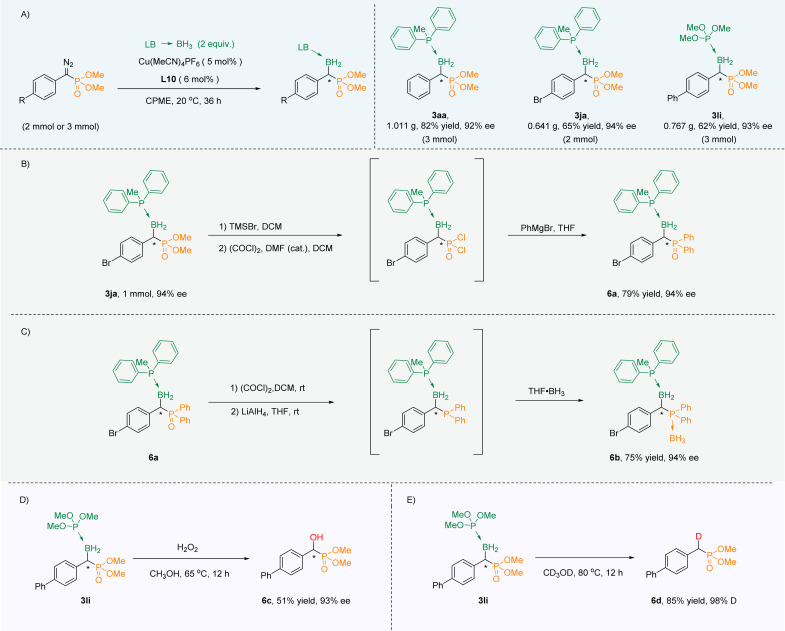
(A) Scale up experiments. (B) Phosphate converted into aryl phosphorus. (C) Phosphine oxide converted into phosphoborane. (D) Oxidation, and (E) deuteration.

In order to show its practicability, the amplification experiments were carried out. When the reaction was extended to 36 h, α-boryl phosphonates (3aa, 3ja, 3li) were obtained in gram scale with excellent yields and high ee values (Scheme 3A). At first, phosphate 3ja could be smoothly converted into aryl phosphorus 6a through a three-step one-pot reaction while maintaining the ee value (Scheme 3B). Phosphine oxide 6a could be reduced to trivalent organophosphorus, which in turn could be converted into phosphoborane 6b and the ee value remained unchanged (Scheme 3C). Next, 3li was oxidized with H_2_O_2_ to obtain chiral α-hydroxy phosphate 6c with unchanged chirality (Scheme 3D). Interestingly, 3li could also undergo deuterated deboronation to deliver α-deuterated phosphate 6d in CD_3_OD (Scheme 3E).

### Mechanistic studies

**Scheme 4:**
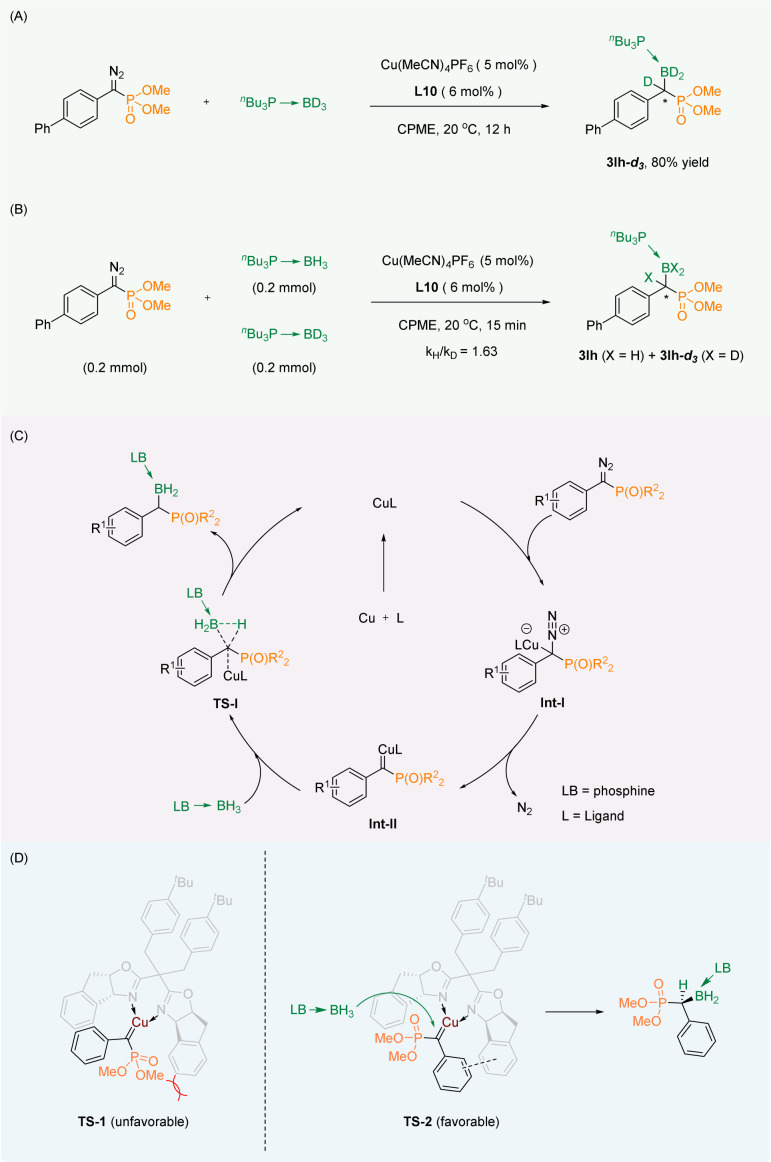
(A) Isotope labelling experiment. (B) KIE experiment. (C) Plausible mechanism. (D) Empirical stereocontrol model.

For research into the mechanism of the reaction, the isotope labelling experiment (Scheme 4A) and the kinetic isotope effect (KIE) experiment (Scheme 4B) were carried out. We successfully obtained the deuterated labeling product 3la-*d*_3_, which indicated that the D atom from the borane adduct was transferred to the carbene carbon. The KIE experiment (*k*_H_/*k*_D_ = 1.63) indicated that B–H bond insertion was not the rate-limiting step of the reaction.

Based on the conclusions drawn from the above experiments as well as the reported mechanism of the B–H bond insertion reaction,^35–40^ we proposed a plausible mechanism. First, the diazo group generates copper carbene Int-II through Int-I. Then the B–H bond of the phosphine borane adduct is inserted in a synergistic manner by the transition state TS-I to obtain the corresponding product. Finally, the catalyst is released to continue to participate in the reaction (Scheme 4C). To explain the observed stereochemical outcomes, we have proposed an empirical stereocontrol model based on the precedents.^33,36^ Our proposal involves the interaction of a copper salt coordinated with the chiral oxazoline ligand L10 to create a copper carbene complex with the substrate, utilizing a dual-transition-state mechanism. In comparison to TS-1, the TS-2 transition state alleviates steric hindrance by separating the larger ester group from the phenyl group of the ligand spatially. Additionally, there is a likelihood of π–π stacking between the two benzene rings in the TS-2 transition state. As a result, the B–H insertion occurs selectively on the unhindered face of the carbene close to the phosphate group in the TS-2 transition state, leading to the formation of the S-configured product (Scheme 4D).

## Conclusions

In summary, we have established a copper-catalyzed asymmetric B–H bond insertion reaction of α-phosphonocarbene with borane adducts. By using this method, we have synthesized stable chiral α-boryl phosphates with high yields and good to excellent enantioselectivities. This approach exhibits a wide range of substrates and demonstrates excellent tolerance towards various functional groups. The obtained products can be easily converted into chiral α-boryl diarylphosphine oxide, chiral α-boryl diarylphosphine borane, highly enantiomerically retained α-hydroxy phosphate and α-deuterated phosphate, showing that this method has application potential in organic synthesis.

## Data availability

The data supporting this article have been included in ESI.†

## Author contributions

Q. S. conceived and directed the project. L. L. designed and performed experiments. Y. K., A. J. & C. X. helped with the collection of some new compounds and data analysis. Q. S. & L. L. wrote the paper with input from all other authors. All authors discussed the results and commented on the manuscript.

## Conflicts of interest

The authors declare no competing interests.

## Supplementary Material

SC-015-D4SC01271B-s001

SC-015-D4SC01271B-s002
