# Supplementary material for: Enantioselective copper-catalyzed B–H bond insertion reaction of α-diazo phosphonates to access chiral α-boryl phosphonates
Source: Chem Sci. 2024 Apr 10;15(19):7130–5. doi: 10.1039/d4sc01271b (PMC11095379; doi:10.1039/d4sc01271b)

Electronic Supplementary Material (ESI) for Chemical Science.
This journal is © The Royal Society of Chemistry 2024

Enantioselective copper-catalyzed B-H bond insertion reaction of α-diazo phosphonates to access chiral α-boryl phosphonates

Longlong Li, Kui Yu, Hejun An, Xinping Cai, Qiuling Song*

Key Laboratory of Molecule Synthesis and Function Discovery, Fujian Province University, College of Chemistry at Fuzhou University, Fuzhou, Fujian, 350108, China

Table of Content

1. Experimental studies S2

1.1 General information S2

1.2 General procedure for preparation of α-diazo phosphonates S2

1.3 General procedure C for preparation of phosphine-borane adducts S16

1.4 General procedure D for the synthesis of chiral α-boryl phosphonates S19

1.5 Further synthetic transformation S81

1.6 Mechanism study of the B-H bond insertion reaction........ S87

1.7 Crystal structure of compound 3aa S89

2. References S91

3. NMR spectra S92

1. Experimental studies

1.1 General information

All experiments were conducted with a schlenk tube. Flash column chromatography was performed over silica gel (200-300 mesh). ^{1}H NMR and ^{13}C NMR spectra were recorded at ambient temperature using Bruker 400M spectrometers and JEOL 500M spectrometers, chemical shifts (in ppm) were referenced to $CDCl_3$ (δ = 7.26 ppm) as internal standards. ^{13}C NMR spectra were obtained by using the same NMR spectrometers and were calibrated with $CDCl_3$ (δ = 77.0 ppm). Data for ^{1}H NMR are recorded as following abbreviations: multiplicity (s = singlet, d = doublet, t = triplet, q = quarter, m = multiplet), coupling constant (J, Hz). High resolution mass spectroscopy (HRMS) analyses were performed at an Exactive Plus (Thermo Scientific) or Agilent Mass Spectrometer. Unless otherwise noted, materials obtained from commercial suppliers were used without further purification.

1.2 General procedure for preparation of α-diazo phosphonates

General procedure A: Synthesis of α-diazo phosphonates (1a-1p, 1r-1C):

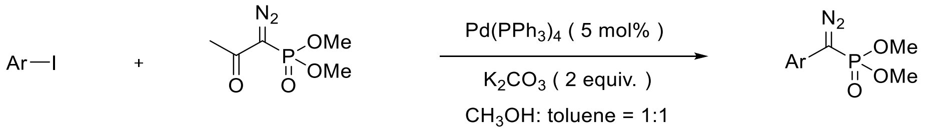

$Pd(PPh_3)_4$ (0.29 g, 5 mol%), K_2CO_3 (1.38 g, 10.0 mmol) were suspended in methanol (10 mL) and toluene (10 mL) in a 100 mL flask under argon. Aryl iodide (5.0 mmol) and dimethyl (1-diazo-2-oxopropyl)phosphonate (1.25 g, 1.3 equiv) was then added, and the resulting solution was stirred at room temperature for 12 h. Then, this reaction was quenched by 20 mL H_2O, extracted with ethyl acetate, dried over Na_2SO_4, and evaporated in vacuo to remove the volatile materials. The crude residue was purified by column chromatography (silica gel, petroleum ether:ethyl acetate = 3:1) to afford the final products. [1]

General procedure B: Synthesis of α-diazo phosphonates (1q-1p, 4a-4c):

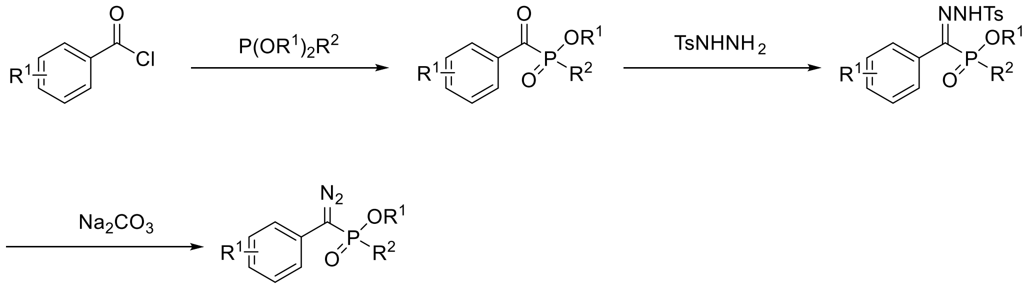

To a 50 mL flask was added acid chloride (10 mmol, 1.0 equiv) under argon at 0 °C. Then $P(OR^1)_2R^2$ (10 mmol, 1.0 equiv) was added dropwise and the mixture was stirred at room temperature for 4 h. The resulting yellow oil **A** was used in the next step without further purification.

A suspension of $TsNHNH_2$ (1.86 g, 10 mmol, 1.0 equiv) in THF (10 mL) in a 25 mL flask was chilled to 0 °C and then concentrated HCl (0.42 mL, 5.0 mmol, 0.5 equiv) was added. The resulting solution was stirred at 0 °C while the resulting yellow oil **A** was added dropwise. The flask was stoppered and the mixture was allowed to warm to room temperature and stirred for 12 h. The organic layers were concentrated under reduced pressure to yield the crude product **B** (the corresponding N-tosylhydrazone).

Na_2CO_3 (2.4 g, 22 mmol, 2.2 equiv) was added to the corresponding N-tosylhydrazone **B** without further purification. Then water (25 mL) and Et_2O (5 mL) was added and the mixture was stirred at room temperature for 24 h. When the stirring was complete, the mixture was extracted with ethyl acetate three times, washed with water and brine, dried over Na_2SO_4 and concentrated under reduced pressure. The crude residue was purified by chromatography (silica gel, PE: EtOAc =3:1), to give final product. [3]

Dimethyl (diazo(phenyl)methyl)phosphonate (1a)

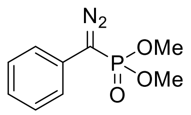

1a

Following the procedure A on 5 mmol scale, orange oil, yield: 62% (700.6 mg), column chromatography (silica gel, PE: EtOAc = 3:1, v/v). Spectroscopic data are in agreement with those previously reported.[1]

^{1}H NMR (400 MHz, Chloroform-*d*) δ 7.38 – 7.32 (m, 2H), 7.18 – 7.11 (m, 3H), 3.80 (d, *J* = 11.9 Hz, 6H).

Dimethyl ((4-chlorophenyl)(diazo)methyl)phosphonate (1b)

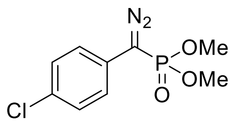

1b

Following the procedure A on 5 mmol scale, orange oil, yield: 60% (781.1 mg), column chromatography (silica gel, PE: EtOAc = 3:1, v/v). Spectroscopic data are in agreement with those previously reported. [2]

^{1}H NMR (500 MHz, Chloroform-*d*) δ 7.33 – 7.29 (m, 2H), 7.10 – 7.06 (m, 2H), 3.80 (d, *J* = 11.9 Hz, 6H).

Dimethyl (diazo(4-(hydroxymethyl)phenyl)methyl)phosphonate (1c)

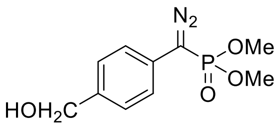

1c

Following the procedure A on 5 mmol scale, orange oil, yield: 55% (704.0 mg), column chromatography (silica gel, PE: EtOAc = 3:1, v/v). Spectroscopic data are in agreement with those previously reported. [2]

^{1}H NMR (500 MHz, Chloroform-*d*) δ 7.36 – 7.31 (m, 2H), 7.15 – 7.07 (m, 2H), 4.62 (s, 2H), 3.77 (d, *J* = 11.9 Hz, 6H), 2.68 (s, 1H).

Dimethyl (diazo(4-methoxyphenyl)methyl)phosphonate (1d)

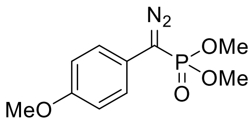

1d

Following the procedure A on 5 mmol scale, orange oil, 53% (678.2 mg), column chromatography (silica gel, PE: EtOAc = 3:1, v/v). Spectroscopic data are in agreement with those previously reported. [2]

^{1}H NMR (500 MHz, Chloroform-*d*) δ 7.09 – 7.05 (m, 2H), 6.92 – 6.87 (m, 2H), 3.79 – 3.75 (m, 9H).

Dimethyl (diazo(p-tolyl)methyl)phosphonate (1e)

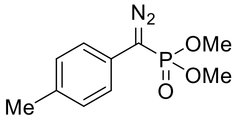

1e

Following the procedure A on 5 mmol scale, orange oil, yield: 67% (803.9 mg), column chromatography (silica gel, PE: EtOAc = 3:1, v/v). Spectroscopic data are in agreement with those previously reported. [2]

^{1}H NMR (500 MHz, Chloroform-*d*) δ 7.19 – 7.15 (m, 2H), 7.07 – 7.03 (m, 2H), 3.80 (d, *J* = 12.0 Hz, 6H), 2.32 (s, 3H).

Dimethyl (diazo(4-(methylthio)phenyl)methyl)phosphonate (1f)

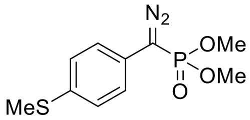

1f

Following the procedure A on 5 mmol scale, orange oil, yield: 31% (421.6 mg), column chromatography (silica gel, PE: EtOAc = 3:1, v/v).

^{1}H NMR (500 MHz, Chloroform-*d*) δ 7.28 – 7.24 (m, 2H), 7.10 – 7.07 (m, 2H), 3.81 (d, *J* = 12.0 Hz, 6H), 2.47 (s, 3H).

^{13}C NMR (126 MHz, Chloroform-*d*) δ 135.4, 127.9, 123.1 (d, *J* = 4.6 Hz), 122.9 (d, *J* = 9.7 Hz), 53.1 (d, *J* = 5.2 Hz), 16.1.

^{31}P NMR (202 MHz, Chloroform-*d*) δ 21.41.

Dimethyl (diazo(4-nitrophenyl)methyl)phosphonate (1g)

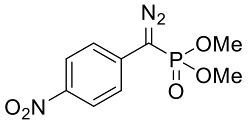

1g

Following the procedure A on 5 mmol scale, orange oil, yield: 74% (1.0 g), column chromatography (silica gel, PE: EtOAc = 3:1, v/v). Spectroscopic data are in agreement with those previously reported.[1]

¹H NMR (500 MHz, Chloroform-*d*) δ 8.23 – 8.19 (m, 2H), 7.30 – 7.26 (m, 2H), 3.86 (d, *J* = 11.9 Hz, 6H).

Methyl-4-(diazo(dimethoxyphosphoryl)methyl)benzoate (1h)

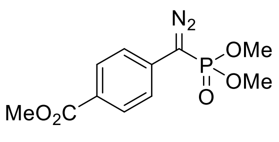

1h

Following the procedure A on 5 mmol scale, orange oil, yield: 77 % (1.10 g), column chromatography (silica gel, PE: EtOAc = 3:1, v/v). Spectroscopic data are in agreement with those previously reported. [2]

¹H NMR (500 MHz, Chloroform-*d*) δ 7.99 – 7.96 (m, 2H), 7.19 – 7.15 (m, 2H), 3.87 (s, 3H), 3.80 (d, *J* = 12.0 Hz, 6H).

Dimethyl (diazo(4-fluorophenyl)methyl)phosphonate (1i)

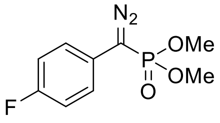

1i

Following the procedure A on 5 mmol scale, orange oil, yield: 65% (793.7 mg), column chromatography (silica gel, PE: EtOAc = 3:1, v/v). Spectroscopic data are in agreement with those previously reported. [2]

¹H NMR (500 MHz, Chloroform-*d*) δ 7.15 – 7.10 (m, 2H), 7.08 – 7.03 (m, 2H), 3.80 (d, *J* = 11.9 Hz, 6H).

Dimethyl ((4-bromophenyl)(diazo)methyl)phosphonate (1j)

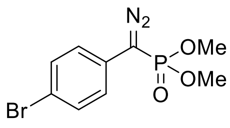

1j

Following the procedure A on 5 mmol scale, orange oil, yield: 63% (961.1 mg), column chromatography (silica gel, PE: EtOAc = 3:1, v/v). Spectroscopic data are in agreement with those

previously reported. [2]

^{1}H NMR (500 MHz, Chloroform-*d*) δ 7.46 – 7.43 (m, 2H), 7.03 – 6.99 (m, 2H), 3.79 (d, *J* = 11.9 Hz, 6H).

Dimethyl (diazo(4-(trifluoromethyl)phenyl)methyl)phosphonate (1k)

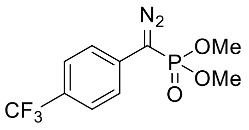

1k

Following the procedure A on 5 mmol scale, orange oil, yield: 68% (1.0 g), column chromatography (silica gel, PE: EtOAc = 3:1, v/v).

^{1}H NMR (400 MHz, Chloroform-*d*) δ 7.57 (d, *J* = 8.3 Hz, 2H), 7.24 (d, *J* = 8.3 Hz, 2H), 3.81 (d, *J* = 11.9 Hz, 6H).

^{13}C NMR (101 MHz, Chloroform-*d*) δ 131.2 (d, *J* = 9.9 Hz), 127.1 (q, *J* = 32.8 Hz), 126.1 (q, *J* = 3.9 Hz), 124.0 (q, *J* = 271.6 Hz), 122.3 (d, *J* = 4.5 Hz), 53.2 (d, *J* = 5.1 Hz).

^{31}P NMR (162 MHz, Chloroform-*d*) δ 19.19.

^{19}F NMR (376 MHz, Chloroform-*d*) δ -62.48.

Dimethyl ([1,1'-biphenyl]-4-yl(diazo)methyl)phosphonate (1l)

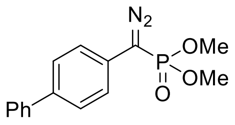

1l

Following the procedure A on 5 mmol scale, orange solid, yield: 47 % (709.2 mg), column chromatography (silica gel, PE: EtOAc = 3:1, v/v). Spectroscopic data are in agreement with those previously reported. [2]

^{1}H NMR (500 MHz, Chloroform-*d*) δ 7.62 – 7.56 (m, 4H), 7.46 – 7.42 (m, 2H), 7.37 – 7.33 (m, 1H), 7.25 – 7.22 (m, 2H), 3.84 (d, *J* = 11.9 Hz, 6H).

Dimethyl ((4-(benzyloxy)phenyl)(diazo)methyl)phosphonate (1m)

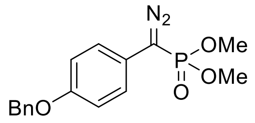

1m

Following the procedure A on 5 mmol scale, orange solid (mp: 41.3 – 42.5 °C), yield: 34% (564.9 mg), column chromatography (silica gel, PE: EtOAc = 3:1, v/v).

^{1}H NMR (400 MHz, Chloroform-*d*) δ 7.45 – 7.30 (m, 5H), 7.13 – 7.07 (m, 2H), 7.02 – 6.97 (m, 2H), 5.05 (s, 2H), 3.80 (d, J = 11.9 Hz, 6H).

^{13}C NMR (101 MHz, Chloroform-*d*) δ 156.8, 136.7, 128.5, 127.9, 127.3, 124.3 (d, J = 4.4 Hz), 117.7 (d, J = 9.6 Hz), 116.0, 70.0, 53.0 (d, J = 5.1 Hz).

^{31}P NMR (162 MHz, Chloroform-*d*) δ 21.57.

Dimethyl ((4-cyanophenyl)(diazo)methyl)phosphonate (1n)

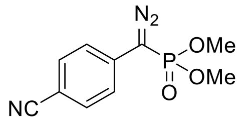

1n

Following the procedure A on 5 mmol scale, orange oil, yield: 64% (803.8 mg), column chromatography (silica gel, PE: EtOAc = 3:1, v/v). Spectroscopic data are in agreement with those previously reported.[3]

^{1}H NMR (500 MHz, Chloroform-*d*) δ 7.62 – 7.59 (m, 2H), 7.24 – 7.21 (m, 2H), 3.82 (d, J = 11.9 Hz, 6H).

Dimethyl (diazo(3-methoxyphenyl)methyl)phosphonate (1o)

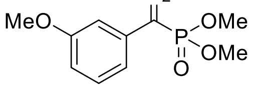

1o

Following the procedure A on 5 mmol scale, orange oil, yield: 67% (858.7 mg), column chromatography (silica gel, PE: EtOAc = 3:1, v/v). Spectroscopic data are in agreement with those previously reported. [2]

^{1}H NMR (500 MHz, Chloroform-*d*) δ 7.25 (t, *J* = 8.0 Hz, 1H), 6.75 – 6.71 (m, 1H), 6.70 – 6.66 (m, 2H), 3.81 – 3.77 (m, 9H).

Methyl 3-(diazo(dimethoxyphosphoryl)methyl)benzoate (1p)

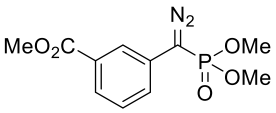

1p

Following the procedure A on 5 mmol scale, red oil, yield: 62% (880.6 mg), column chromatography (silica gel, PE: EtOAc = 3:1, v/v).

^{1}H NMR (500 MHz, Chloroform-*d*) δ 7.80 – 7.76 (m, 2H), 7.41 (t, *J* = 7.2 Hz, 1H), 7.37 – 7.34 (m, 1H), 3.90 (s, 3H), 3.80 (d, *J* = 11.9 Hz, 6H).

^{13}C NMR (126 MHz, Chloroform-*d*) δ 166.3, 131.1, 129.2, 127.2 (d, *J* = 9.8 Hz), 126.6 (d, *J* = 3.7 Hz), 126.1, 123.1 (d, *J* = 5.1 Hz), 53.1 (d, *J* = 5.1 Hz), 52.1.

^{31}P NMR (202 MHz, Chloroform-*d*) δ 20.6.

Dimethyl (diazo(3-iodophenyl)methyl)phosphonate (1q)

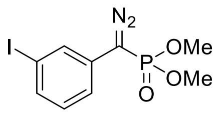

1q

Following the procedure B on 10 mmol scale, orange oil, yield: 50% (1.73 g), column chromatography (silica gel, PE: EtOAc = 3:1, v/v).

^{1}H NMR (400 MHz, Chloroform-*d*) δ 7.49 – 7.39 (m, 2H), 7.14 – 7.00 (m, 2H), 3.79 (dd, *J* = 11.9, 3.7 Hz, 6H).

^{13}C NMR (101 MHz, Chloroform-*d*) δ 134.1, 130.8 (d, *J* = 4.8 Hz), 130.6, 128.9 (d, *J* = 9.8 Hz), 121.7 (d, *J* = 4.1 Hz), 95.0, 53.2 (d, *J* = 5.1 Hz).

^{31}P NMR (162 MHz, Chloroform-*d*) δ 19.65.

Dimethyl (diazo(2-fluorophenyl)methyl)phosphonate (1r)

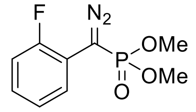

1r

Following the procedure B on 10 mmol scale, orange oil, yield: 70% (1.71 g), column chromatography (silica gel, PE: EtOAc = 3:1, v/v). Spectroscopic data are in agreement with those previously reported.[3]

^{1}H NMR (500 MHz, Chloroform-*d*) δ 7.31 – 7.26 (m, 1H), 7.18 – 7.13 (m, 1H), 7.12 – 7.07 (m, 1H), 7.07 – 7.01 (m, 1H), 3.79 (m, 6H).

Dimethyl ((3-chloro-4-fluorophenyl)(diazo)methyl)phosphonate (1s)

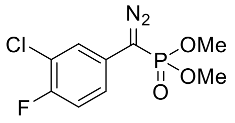

1s

Following the procedure A on 5 mmol scale, orange oil, yield: 40% (558.2 mg), column chromatography (silica gel, PE: EtOAc = 3:1, v/v).

^{1}H NMR (400 MHz, Chloroform-*d*) δ 7.15 – 7.02 (m, 2H), 7.00 – 6.91 (m, 1H), 3.74 (d, *J* = 12.2 Hz, 6H).

^{13}C NMR (101 MHz, Chloroform-*d*) δ 155.6 (d, *J* = 248.1 Hz), 124.3 (d, *J* = 4.4 Hz), 123.7 (dd, *J* = 9.8, 3.8 Hz), 122.2 (dd, *J* = 7.0, 4.4 Hz), 121.9 (d, *J* = 18.6 Hz), 117.2 (d, *J* = 21.7 Hz), 53.1 (d, *J* = 5.0 Hz).

^{31}P NMR (162 MHz, Chloroform-*d*) δ 19.49.

^{19}F NMR (376 MHz, Chloroform-*d*) δ -119.61.

Dimethyl (diazo(4-methyl-3-nitrophenyl)methyl)phosphonate (1t)

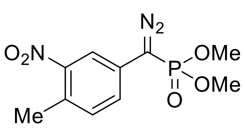

1t

Following the procedure A on 5 mmol scale, orange soild (mp: 67.5 – 68.6 °C), yield: 38% (541.9

mg), column chromatography (silica gel, PE: EtOAc = 3:1, v/v).

^{1}H NMR (400 MHz, Chloroform-*d*) δ 7.74 (d, *J* = 2.0 Hz, 1H), 7.36 – 7.29 (m, 2H), 3.85 (d, *J* = 11.9 Hz, 6H), 2.56 (s, 3H).

^{13}C NMR (101 MHz, Chloroform-*d*) δ 149.8, 133.6, 130.0, 126.5 (d, *J* = 4.2 Hz), 126.4 (d, *J* = 10.0 Hz), 118.1 (d, *J* = 4.9 Hz), 53.3 (d, *J* = 5.2 Hz), 19.8.

^{31}P NMR (162 MHz, Chloroform-*d*) δ 19.00.

Dimethyl (diazo(3,5-dimethoxyphenyl)methyl)phosphonate (1u)

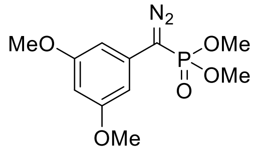

1u

Following the procedure A on 5 mmol scale, orange oil. yield: 53% (753.6 mg), column chromatography (silica gel, PE: EtOAc = 3:1, v/v).

^{1}H NMR (500 MHz, Chloroform-*d*) δ 6.28 (d, *J* = 2.1 Hz, 2H), 6.22 (t, *J* = 2.2 Hz, 1H), 3.77 (d, *J* = 12.0 Hz, 6H), 3.74 (s, 6H).

^{13}C NMR (101 MHz, Chloroform-*d*) δ 161.3, 128.4 (d, *J* = 9.6 Hz), 100.9 (d, *J* = 4.6 Hz), 97.0, 55.1, 53.0 (d, *J* = 5.0 Hz).

^{31}P NMR (162 MHz, Chloroform-*d*) δ 20.55.

Dimethyl (diazo(9H-fluoren-2-yl)methyl)phosphonate (1v)

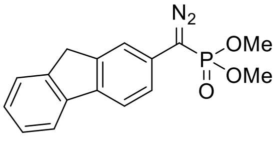

1v

Following the procedure A on 5 mmol scale, red solid (mp: 88.0 – 88.9 °C), yield: 55% (863.3 mg), column chromatography (silica gel, PE: EtOAc = 3:1, v/v).

^{1}H NMR (500 MHz, Chloroform-*d*) δ 7.76 – 7.72 (m, 2H), 7.54 – 7.50 (m, 1H), 7.38 – 7.34 (m, 2H), 7.29 (td, *J* = 7.4, 1.2 Hz, 1H), 7.17 (dd, *J* = 8.2, 1.8 Hz, 1H), 3.88 (s, 2H), 3.84 (d, *J* = 12.0 Hz, 6H).

^{13}C NMR (126 MHz, Chloroform-*d*) δ 143.6 (d, *J* = 219.0 Hz), 140.1 (d, *J* = 202.8 Hz), 126.7 (d, *J* = 12.1 Hz), 124.9, 124.1 (d, *J* = 9.6 Hz), 121.3 (d, *J* = 4.7 Hz), 120.6, 119.6, 119.2 (d, *J* = 4.5 Hz), 53.1 (d,

J = 5.0 Hz), 36.8.

^{31}P NMR (202 MHz, Chloroform-*d*) δ 21.84.

Dimethyl (diazo(1H-indol-5-yl)methyl)phosphonate (1w)

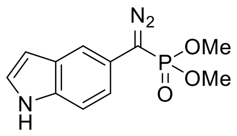

1w

Following the procedure A on 5 mmol scale, red oil, yield: 42% (448 mg), column chromatography (silica gel, PE: EtOAc = 3:1, v/v).

^{1}H NMR (400 MHz, Chloroform-*d*) δ 8.67 (s, 1H), 7.48 (s, 1H), 7.40 (d, *J* = 8.6 Hz, 1H), 7.21 (t, *J* = 2.8 Hz, 1H), 7.01 (dd, *J* = 8.6, 1.9 Hz, 1H), 6.53 – 6.48 (m, 1H), 3.82 (d, *J* = 11.9 Hz, 6H).

^{13}C NMR (101 MHz, Chloroform-*d*) δ 134.3, 128.8, 125.4, 117.8 (d, *J* = 4.5 Hz), 116.2 (d, *J* = 9.0 Hz), 115.6 (d, *J* = 4.2 Hz), 112.2, 102.1 (d, *J* = 1.9 Hz), 53.1 (d, *J* = 5.0 Hz).

^{31}P NMR (162 MHz, Chloroform-*d*) δ 22.8.

Dimethyl (benzo[d][1,3]dioxol-5-yl(diazo)methyl)phosphonate (1x)

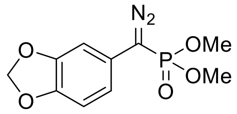

1x

Following the procedure A on 5 mmol scale, red solid (mp: 68.2 – 68.8 °C), yield: 33% (446 mg), column chromatography (silica gel, PE: EtOAc = 3:1, v/v).

^{1}H NMR (400 MHz, Chloroform-*d*) δ 6.78 (d, *J* = 8.2 Hz, 1H), 6.66 (d, *J* = 2.0 Hz, 1H), 6.60 (d, *J* = 8.2 Hz, 1H), 5.92 (s, 2H), 3.77 (d, *J* = 11.7 Hz, 6H).

^{13}C NMR (101 MHz, Chloroform-*d*) δ 148.6, 145.7, 119.1 (d, *J* = 9.6 Hz), 116.3 (d, *J* = 4.8 Hz), 109.1, 104.0 (d, *J* = 4.4 Hz), 101.2, 53.0 (d, *J* = 5.1 Hz).

^{31}P NMR (162 MHz, Chloroform-*d*) δ 21.17.

Dimethyl (diazo(dibenzo[b,d]furan-1-yl)methyl)phosphonate (1y)

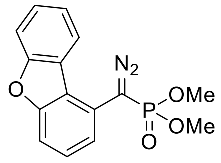

1y

Following the procedure A on 5 mmol scale, orange oil, yield: 65% (1.02 g), column chromatography (silica gel, PE: EtOAc = 3:1, v/v).

^{1}H NMR (400 MHz, Chloroform-*d*) δ 7.89 (d, *J* = 7.4 Hz, 1H), 7.62 (d, *J* = 8.0 Hz, 1H), 7.58 – 7.41 (m, 5H), 3.82 (d, *J* = 11.8 Hz, 6H).

^{13}C NMR (101 MHz, Chloroform-*d*) δ 156.6, 156.3, 127.6, 127.5, 124.1, 123.3, 122.5, 122.4, 119.8, 119.7, 111.6, 111.5, 53.3 (d, *J* = 5.5 Hz).

^{31}P NMR (162 MHz, Chloroform-*d*) δ 20.79.

Dimethyl (diazo(dibenzo[b,d]thiophen-4-yl)methyl)phosphonate (1z)

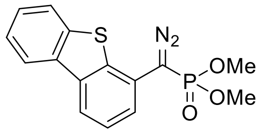

1z

Following the procedure A on 5 mmol scale, orange oil, yield: 28% (646.3 mg), column chromatography (silica gel, PE: EtOAc = 3:1, v/v).

^{1}H NMR (400 MHz, Chloroform-*d*) δ 8.20 – 8.13 (m, 1H), 8.14 – 8.07 (m, 1H), 7.93 – 7.85 (m, 1H), 7.55 – 7.46 (m, 4H), 3.83 (d, *J* = 11.9 Hz, 6H).

^{13}C NMR (101 MHz, Chloroform-*d*) δ 138.6, 138.3 (d, *J* = 6.4 Hz), 136.8, 135.5, 127.3, 126.7, 125.5, 124.8, 122.8, 121.8, 121.0, 120.0 (d, *J* = 9.7 Hz), 53.4 (d, *J* = 5.3 Hz).

^{31}P NMR (162 MHz, Chloroform-*d*) δ 20.89.

Dimethyl (diazo(phenanthren-9-yl)methyl)phosphonate (1A)

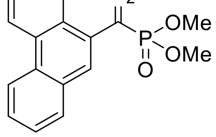

1A

Following the procedure A on 5 mmol scale, orange oil, yield: 58% (946.3 mg), column

chromatography (silica gel, PE: EtOAc = 3:1, v/v).

^{1}H NMR (400 MHz, Chloroform-*d*) δ 8.80 – 8.73 (m, 1H), 8.67 (d, *J* = 8.3 Hz, 1H), 8.17 – 8.08 (m, 1H), 7.95 (s, 1H), 7.90 (d, *J* = 7.8 Hz, 1H), 7.75 – 7.66 (m, 3H), 7.62 (t, *J* = 7.4 Hz, 1H), 3.83 (d, *J* = 11.7 Hz, 6H).

^{13}C NMR (101 MHz, Chloroform-*d*) δ 131.3, 131.1, 130.7 (d, *J* = 2.6 Hz), 130.4, 130.2 (d, *J* = 4.2 Hz), 128.9, 127.5, 127.3, 127.0 (d, *J* = 1.9 Hz), 124.6, 123.5, 122.5, 120.7 (d, *J* = 8.2 Hz), 53.4 (d, *J* = 5.8 Hz).

^{31}P NMR (202 MHz, Chloroform-*d*) δ 22.57.

Dimethyl (diazo(naphthalen-2-yl)methyl)phosphonate (1B)

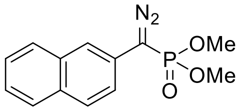

1B

Following the procedure A on 5 mmol scale, orange oil, yield: 45% (620.8 mg), column chromatography (silica gel, PE: EtOAc = 3:1, v/v).

^{1}H NMR (400 MHz, Chloroform-*d*) δ 7.84 (d, *J* = 8.7 Hz, 1H), 7.78 (t, *J* = 8.8 Hz, 2H), 7.59 (d, *J* = 2.0 Hz, 1H), 7.51 – 7.45 (m, 1H), 7.45 – 7.40 (m, 1H), 7.28 (dd, *J* = 8.7, 2.1 Hz, 1H), 3.85 (d, *J* = 12.0 Hz, 6H).

^{13}C NMR (101 MHz, Chloroform-*d*) δ 133.7, 131.1, 129.1, 127.6, 127.2, 126.7, 125.5, 123.4 (d, *J* = 9.5 Hz), 120.9 (d, *J* = 5.0 Hz), 120.6 (d, *J* = 4.1 Hz), 53.1 (d, *J* = 5.1 Hz), 50.2 (d, *J* = 227.8 Hz).

^{31}P NMR (162 MHz, Chloroform-*d*) δ 20.90.

Dimethyl (diazo(naphthalen-1-yl)methyl)phosphonate (1C)

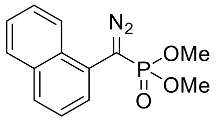

1C

Following the procedure A on 5 mmol scale, orange oil, yield: 55% (759.5 mg), column chromatography (silica gel, PE: EtOAc = 3:1, v/v). Spectroscopic data are in agreement with those previously reported.[4]

^{1}H NMR (500 MHz, Chloroform-*d*) δ 8.04 – 8.00 (m, 1H), 7.93 – 7.90 (m, 1H), 7.88 – 7.84 (m, 1H),

7.65 – 7.60 (m, 2H), 7.57 – 7.53 (m, 1H), 7.50 (dd, J = 8.2, 7.2 Hz, 1H), 3.80 (d, J = 11.7 Hz, 6H).

Diethyl (diazo(phenyl)methyl)phosphonate (4a)

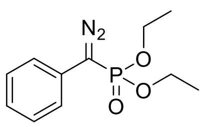

4a

Following the procedure B on 10 mmol scale, orange oil, yield: 67% (1.71 g), column chromatography (silica gel, PE: EtOAc = 3:1, v/v). Spectroscopic data are in agreement with those previously reported.[4]

^{1}H NMR (500 MHz, Chloroform-*d*) δ 7.36 – 7.32 (m, 2H), 7.19 – 7.15 (m, 2H), 7.15 – 7.11 (m, 1H), 4.26 – 4.08 (m, 4H), 1.33 (td, J = 7.1, 0.8 Hz, 6H).

Diisopropyl (diazo(phenyl)methyl)phosphonate (4b)

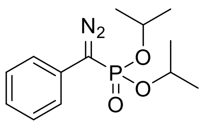

4b

Following the procedure B on 10 mmol scale, orange oil, yield: 40% (1.12 g), column chromatography (silica gel, PE: EtOAc = 3:1, v/v). Spectroscopic data are in agreement with those previously reported.[4]

^{1}H NMR (500 MHz, Chloroform-*d*) δ 7.36 – 7.31 (m, 2H), 7.19 – 7.16 (m, 2H), 7.13 – 7.08 (m, 1H), 4.75 (dp, J = 8.2, 6.2 Hz, 2H), 1.39 (d, J = 6.2 Hz, 6H), 1.24 (d, J = 6.2 Hz, 6H).

Dihexyl (diazo(phenyl)methyl)phosphonate (4c)

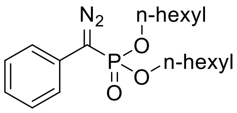

4c

Following the procedure B on 10 mmol scale, orange oil, yield: 58% (1.13 g), column chromatography (silica gel, PE: EtOAc = 3:1, v/v).

^{1}H NMR (500 MHz, Chloroform-*d*) δ 7.36 – 7.31 (m, 2H), 7.19 – 7.15 (m, 2H), 7.12 (t, J = 7.4 Hz,

1H), 4.18 – 3.99 (m, 4H), 1.69 – 1.62 (m, 4H), 1.38 – 1.30 (m, 4H), 1.28 – 1.20 (m, 8H), 0.85 (t, J = 6.9 Hz, 6H).

^{13}C NMR (126 MHz, Chloroform-*d*) δ 129.1, 126.7 (d, J = 9.6 Hz), 125.0, 122.6 (d, J = 4.5 Hz), 66.8 (d, J = 5.2 Hz), 31.2, 30.1 (d, J = 7.0 Hz), 25.1, 22.4, 13.9.

^{31}P NMR (202 MHz, Chloroform-*d*) δ 18.11.

1.3 General procedure C for preparation of phosphine-borane adducts

A solution of phosphine (1.0 equiv) in anhydrous THF (20 mL) was added a borane-tetrahydrofuran complex solution (1.0 mol/L in THF) (20 mL, 20 mmol, 2.0 equiv) dropwise at 0 °C. The resulting mixture was stirred overnight. The solution was then slowly added sat. $NaHCO_3$ (10 mL) and extracted with ethyl acetate (3 x 20 mL). The combined organic layer was dried over anhydrous Na_2SO_4 and the solvent was evaporated. The crude residue was purified by column chromatography (silica gel, PE: EtOAc =10:1) to afford the final products.

Methyldiphenylphosphane borane (2a)

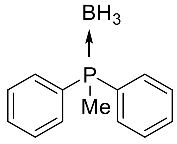

2a

Following the procedure C on 10 mmol scale, white solid, yield: 95% (2.03 g), column chromatography (silica gel, PE: EtOAc =10:1, v/v). Spectroscopic data are in agreement with those previously reported.[5]

^{1}H NMR (500 MHz, Chloroform-*d*) δ 7.70 – 7.63 (m, 4H), 7.51 – 7.46 (m, 2H), 7.46 – 7.41 (m, 4H), 1.87 (d, J = 10.2 Hz, 3H), 1.41 – 0.58 (m, 3H).

Ethyldiphenylphosphane borane (2b)

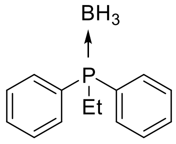

2b

Following the procedure C on 10 mmol scale, white solid, yield: 92% (2.09 g), column chromatography (silica gel, PE: EtOAc =10:1, v/v). Spectroscopic data are in agreement with those

previously reported.[6]

^{1}H NMR (500 MHz, Chloroform-*d*) δ 7.7 – 7.6 (m, 4H), 7.5 – 7.4 (m, 6H), 1.9 (d, *J* = 10.2 Hz, 3H), 1.4 – 0.6 (m, 3H).

Isopropyldiphenylphosphane borane (2c)

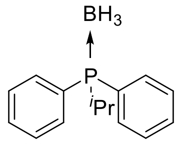

2c

Following the procedure C on 10 mmol scale, white solid, yield: 93% (2.25 g), column chromatography (silica gel, PE: EtOAc =10:1, v/v). Spectroscopic data are in agreement with those previously reported.[7]

^{1}H NMR (400 MHz, Chloroform-*d*) δ 7.82 – 7.70 (m, 4H), 7.51 – 7.39 (m, 6H), 2.72 (dhept, *J* = 14.0, 7.0 Hz, 1H), 1.16 (m, 9H).

Dimethyl(phenyl)phosphane borane (2d)

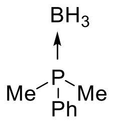

2d

Following the procedure C on 10 mmol scale, white solid, yield: 91% (1.37 g), column chromatography (silica gel, PE: EtOAc =10:1, v/v). Spectroscopic data are in agreement with those previously reported. [5]

^{1}H NMR (500 MHz, Chloroform-*d*) δ 7.76 – 7.69 (m, 2H), 7.52 – 7.43 (m, 3H), 1.56 (d, *J* = 10.4 Hz, 6H), 1.12 – 0.41 (m, 3H).

Diethyl(phenyl)phosphane borane (2e)

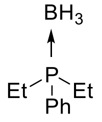

2e

Following the procedure C on 10 mmol scale, white solid, yield: 94% (1.69 g), column

chromatography (silica gel, PE: EtOAc =10:1, v/v). Spectroscopic data are in agreement with those previously reported.[5]

^{1}H NMR (500 MHz, Chloroform-*d*) δ 7.76 – 7.65 (m, 2H), 7.53 – 7.42 (m, 3H), 1.94 – 1.80 (m, 4H), 1.05 (dt, *J* = 16.5, 7.6 Hz, 6H), 1.00 – 0.31 (m, 3H).

Dicyclohexyl(phenyl)phosphane borane (2f)

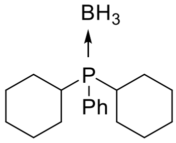

2f

Following the procedure C on 10 mmol scale, white solid, yield: 91% (2.61 g), column chromatography (silica gel, PE: EtOAc =10:1, v/v). Spectroscopic data are in agreement with those previously reported.[8]

^{1}H NMR (400 MHz, Chloroform-*d*) δ 7.68 (t, *J* = 8.3 Hz, 2H), 7.52 – 7.39 (m, 3H), 2.15 – 2.00 (m, 2H), 1.94 (d, *J* = 10.5 Hz, 2H), 1.85 – 1.55 (m, 8H), 1.37 – 1.05 (m, 10H), 0.61 (q, *J* = 81.0Hz, 3H).

Trimethylphosphane borane (2g)

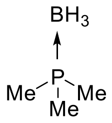

2g

Following the procedure C on 10 mmol scale, white solid, yield: 80% (0.71 g), column chromatography (silica gel, PE: EtOAc =10:1, v/v). Spectroscopic data are in agreement with those previously reported.[5]

^{1}H NMR (500 MHz, Chloroform-*d*) δ 1.32 (d, *J* = 10.6 Hz, 9H), 0.82 – 0.16 (m, 3H).

Tributylphosphane borane (2h)

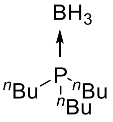

2h

Following the procedure C on 10 mmol scale, colorless oil, yield: 94% (2.05 g), column

chromatography (silica gel, PE: EtOAc =10:1, v/v). Spectroscopic data are in agreement with those previously reported.[5]

^{1}H NMR (500 MHz, Chloroform-*d*) δ 1.60 – 1.51 (m, 6H), 1.50 – 1.32 (m, 12H), 0.92 (t, *J* = 7.1 Hz, 9H), 0.74 – 0.06 (m, 3H).

Trimethylphosphite borane (2i)

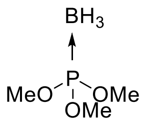

2i

A solution of phosphine (1.0 equiv) in anhydrous THF (20 mL) was added a borane-tetrahydrofuran complex solution (1.0 mol/L in THF) (20 mL, 20 mmol, 2.0 equiv) dropwise at 0 °C. The resulting mixture was stirred overnight at rt. The solution was then slowly added sat. $NaHCO_3$ (10 mL) and extracted with ethyl acetate (3 x 20 mL). The combined organic layer was dried over anhydrous Na_2SO_4 and the solvent was evaporated. The crude residue was purified by column chromatography (silica gel, PE: EtOAc =10:1) to afford the final products. colorless oil, yield: 85% (1.18 g). Spectroscopic data are in agreement with those previously reported.[9]

^{1}H NMR (500 MHz, Chloroform-*d*) δ 3.70 (dd, *J* = 11.0, 1.6 Hz, 9H), 0.76 – 0.04 (m, 3H).

1.4 General procedure D for the synthesis of chiral α-boryl phosphonates

In air, a 25 mL schlenk tube was charged with $Cu(MeCN)_4PF_6$ (5 mol%), **L1** (6 mol%). The tube was evacuated and filled with argon for three cycles. Then, 2 mL of CPME , **1** or **4** (0.20 mmol, 1.0 equiv) and **2** (0.40 mmol, 2.0 equiv) was added under argon. The reaction was allowed to stir at 20 ^{o}C for 12 hours. Upon completion, proper amount of silica gel was added to the reaction mixture. After removal of the solvent, the crude reaction mixture was purified on silica gel (petroleum ether and ethyl acetate) to afford the desired products.

(S)-dimethyl(((methyldiphenylphosphane)boryl)(phenyl)methyl)phosphonate(3aa)

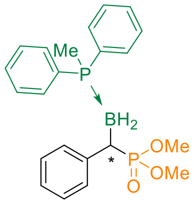

3aa

Following the general procedure D, dimethyl (diazo(phenyl)methyl)phosphonate (0.20 mmol, 1.0 equiv) and methyldiphenylphosphane borane (0.40 mmol, 2.0 equiv) were employed and stirred at 20 °C for 12 h to afford **3aa** as a white solid in 86% yield (71.1 mg) with 92% ee.

mp: 128.0 – 129.3 °C

$\mathbf{R}_f$ = 0.50 (silica gel, EtOAc:PE = 3:1).

^{1}H NMR (500 MHz, Chloroform-*d*) δ 7.52 – 7.38 (m, 8H), 7.35 – 7.30 (m, 2H), 7.14 – 7.07 (m, 4H), 7.05 – 7.00 (m, 1H), 3.60 (dd, *J* = 14.9, 10.5 Hz, 6H), 2.50 – 2.39 (m, 1H), 1.30 (d, *J* = 10.2 Hz, 3H).

^{13}C NMR (126 MHz, Chloroform-*d*) δ 141.5 (dd, *J* = 8.7, 5.4 Hz), 131.8 (dd, *J* = 25.7, 8.9 Hz), 131.1 (dd, *J* = 27.9, 2.5 Hz), 129.7 (d, *J* = 58.1 Hz), 129.4 (d, *J* = 7.7 Hz), 128.8 (dd, *J* = 30.4, 9.9 Hz), 128.0 (d, *J* = 2.8 Hz), 127.8 (d, *J* = 55.4 Hz), 125.0 (d, *J* = 3.5 Hz), 52.7 (dd, *J* = 66.7, 6.9 Hz), 9.2 (d, *J* = 36.8 Hz).

^{11}B NMR (160 MHz, Chloroform-*d*) δ -26.40.

^{31}P NMR (202 MHz, Chloroform-*d*) δ 39.31 (d, *J* = 83.6 Hz), 6.28.

HRMS (ESI): calcd for $(M+H)^+$ $C_{22}H_{28}BO_3P_2^+$ 413.1601; found 413.1611.

HPLC analysis: DAICEL CHIRALCEL ID-3, hexane/isopropanol = 60/40, 1 mL/min, λ = 254 nm, t_R (major) = 15.135 min, t_R (minor) = 11.938 min, 92% ee.

$[\alpha]^{25}_D$: +73.2 (*c* 0.5, $CHCl_3$).

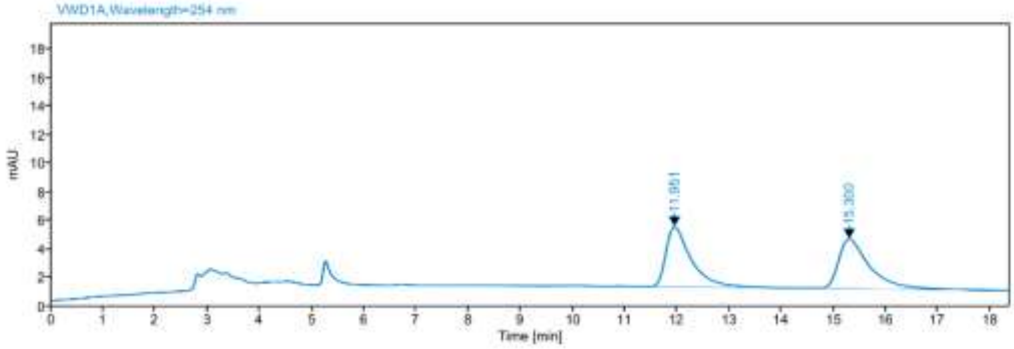

Signal:	VWD1A,Wavelength=254 nm					
RT [min]	Type	Width [min]	Area	Height	Area%	Name
11.951	BB	2.77	143.10	4.21	50.10	
15.300	BB	3.23	142.55	3.45	49.90	
		Sum	285.65			

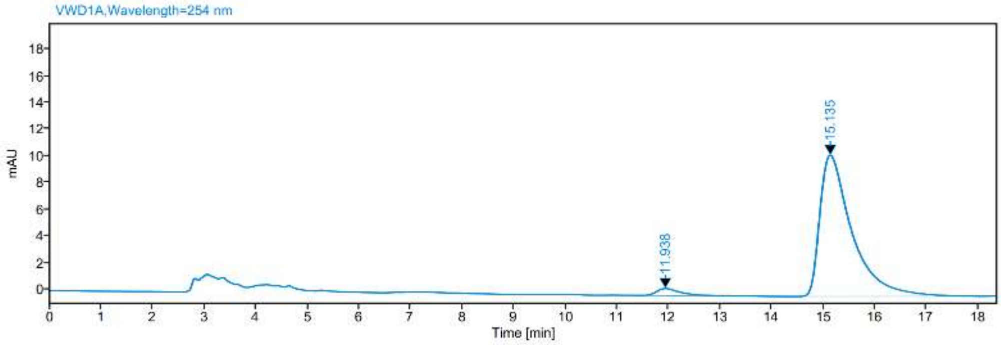

Signal:	VWD1A,Wavelength=254 nm					
RT [min]	Type	Width [min]	Area	Height	Area%	Name
11.938	MM m	2.42	18.26	0.53	3.83	
15.135	BB	3.55	457.99	10.56	96.17	
		Sum	476.25			

(S)-dimethyl(((methyldiphenylphosphane)boryl)(4-chlorophenyl)methyl)phosphonate(3ba)

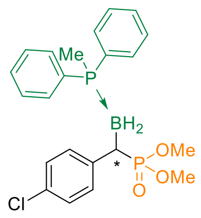

3ba

Following the general procedure D, dimethyl ((4-chlorophenyl)(diazo)methyl)phosphonate (0.20 mmol, 1.0 equiv) and methyldiphenylphosphane borane (0.40 mmol, 2.0 equiv) were employed and stirred at 20 °C for 12 h to afford **3ba** as a colorless oil in 87% yield (77.9 mg) with 95% ee.

R_f = 0.60 (silica gel, EtOAc:PE = 3:1).

^{1}H NMR (500 MHz, Chloroform-*d*) δ 7.51 – 7.39 (m, 8H), 7.36 – 7.30 (m, 2H), 7.06 – 6.99 (m, 4H),

3.60 (dd, J = 14.2, 10.5 Hz, 6H), 2.50 – 2.36 (m, 1H), 1.44 (d, J = 10.2 Hz, 3H).

^{13}C NMR (126 MHz, Chloroform-*d*) δ 140.2 (dd, J = 8.0, 5.8 Hz), 131.7 (dd, J = 24.0, 8.9 Hz), 131.2 (dd, J = 28.4, 2.5 Hz), 130.6 (d, J = 7.7 Hz), 130.5 (d, J = 4.3 Hz), 129.1 (d, J = 57.9 Hz), 128.8 (dd, J = 25.0, 10.0 Hz), 127.9 (d, J = 2.9 Hz), 127.8 (d, J = 55.8 Hz), 52.7 (dd, J = 61.2, 7.1 Hz), 9.5 (d, J = 37.3 Hz).

^{11}B NMR (160 MHz, Chloroform-*d*) δ -26.85.

^{31}P NMR (202 MHz, Chloroform-*d*) δ 38.54 (d, J = 81.0 Hz), 6.05.

HRMS (ESI): calcd for $(M+H)^+$ $C_{22}H_{26}BClO_3P_2^+$ 447.1212, found 447.1212.

HPLC analysis: DAICEL CHIRALCEL ID-3, hexane/isopropanol = 80/20, 1 mL/min, λ = 254 nm, t_R (major) = 25.642 min, t_R (minor) = 28.328 min, 95% ee.

[α]$^{25}_{D}$: +95.8 (c 0.5, $CHCl_3$).

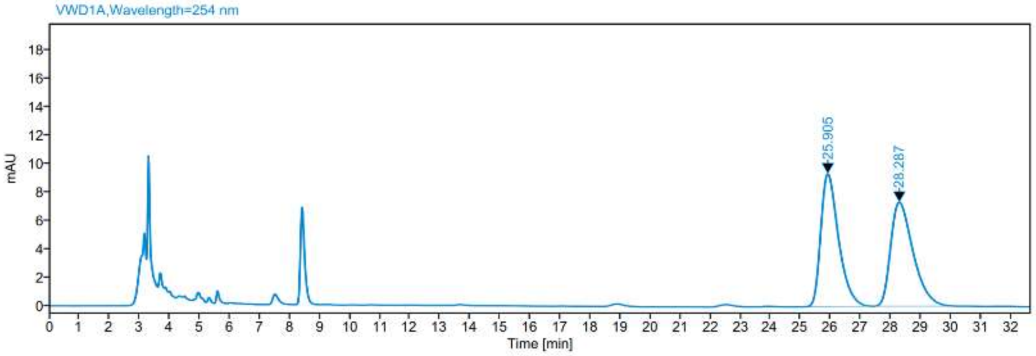

Signal: VWD1A,Wavelength=254 nm

RT [min]	Type	Width [min]	Area	Height	Area%	Name
25.905	BB	2.38	384.13	9.35	50.25	
28.287	BB	2.87	380.29	7.32	49.75	
		Sum	764.43			

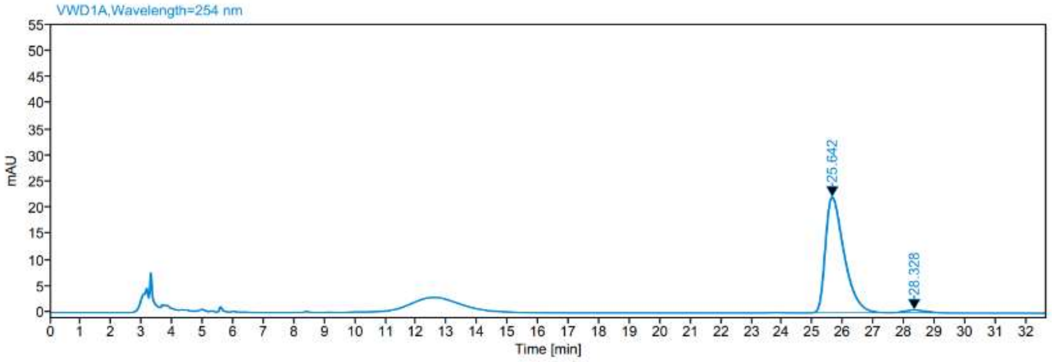

Signal: VWD1A,Wavelength=254 nm

RT [min]	Type	Width [min]	Area	Height	Area%	Name
25.642	BB	2.69	939.28	22.11	97.44	
28.328	MM m	1.97	24.68	0.51	2.56	
		Sum	963.96			

(S)-dimethyl(((methyldiphenylphosphane)boryl)(4-(hydroxymethyl)phenyl)methyl) phosphonate(3ca)

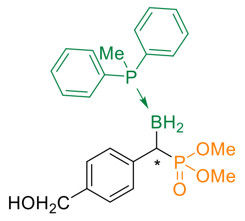

3ca

Following the general procedure D, dimethyl (diazo(4-(hydroxymethyl)phenyl)methyl) phosphonate (0.20 mmol, 1.0 equiv) and methyldiphenylphosphane borane (0.40 mmol, 2.0 equiv) were employed and stirred at 20 °C for 12 h to afford **3ca** as a colorless oil in 87% yield (77.0 mg) with 91% ee.

R_f = 0.18 (silica gel, EtOAc:PE = 3:1).

^{1}H NMR (400 MHz, Chloroform-*d*) δ 7.52 – 7.38 (m, 8H), 7.36 – 7.29 (m, 2H), 7.09 (s, 4H), 4.57 (d, *J* = 1.7 Hz, 2H), 3.59 (dd, *J* = 17.0, 10.6 Hz, 6H), 2.52 – 2.37 (m, 1H), 1.34 (d, *J* = 10.2 Hz, 3H).

^{13}C NMR (126 MHz, Chloroform-*d*) δ 140.7, 137.9 (d, *J* = 3.1 Hz), 131.8 (dd, *J* = 24.6, 8.9 Hz), 131.1 (dd, *J* = 30.7, 2.0 Hz), 129.8, 129.4 (d, *J* = 7.8 Hz), 128.8 (dd, *J* = 29.7, 9.9 Hz), 127.8 (d, *J* = 55.6 Hz), 126.8 (d, *J* = 2.6 Hz), 65.0, 52.7 (dd, *J* = 52.7, 6.8 Hz), 9.3 (d, *J* = 37.0 Hz).

^{11}B NMR (128 MHz, Chloroform-*d*) δ -26.06.

^{31}P NMR (162 MHz, Chloroform-*d*) δ 38.76 (d, *J* = 83.0 Hz), 5.55.

HRMS (ESI): calc'd for $(M+H)^+$ $C_{23}H_{30}BO_4P_2^+$ 443.1707, found 443.1707.

HPLC analysis: DAICEL CHIRALCEL AD-H, hexane/isopropanol = 80/20, 1 mL/min, λ = 254 nm, t_R (major) = 12.609 min, t_R (minor) = 14.270 min, 91% ee.

$[\alpha]^{25}_D$: +62.9 (*c* 0.5, $CHCl_3$).

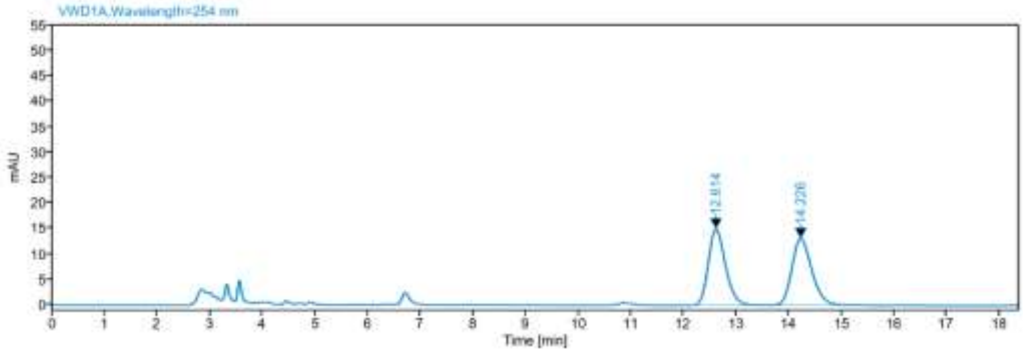

Signal:	VWD1A,Wavelength=254 nm					
RT [min]	Type	Width [min]	Area	Height	Area%	Name
12.614	BB	1.44	340.82	15.05	49.54	
14.226	BB	2.16	347.10	13.15	50.46	
		Sum	687.92			

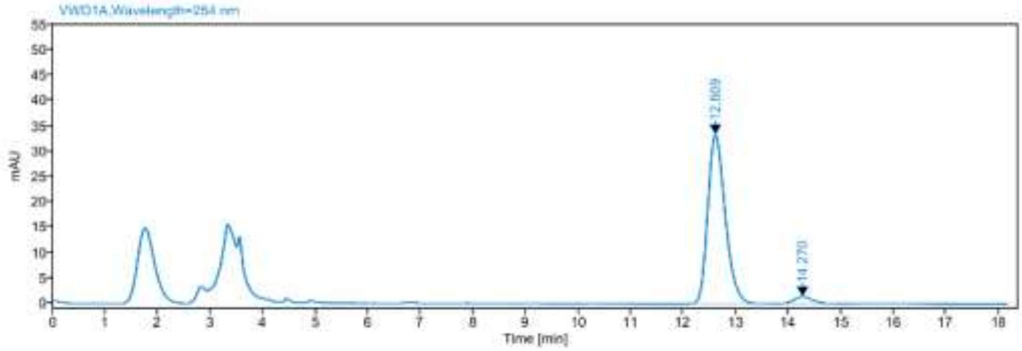

Signal:	VWD1A,Wavelength=254 nm					
RT [min]	Type	Width [min]	Area	Height	Area%	Name
12.609	BB	1.78	770.36	33.36	95.70	
14.270	MM m	1.19	34.61	1.36	4.30	
		Sum	804.97			

(S)-dimethyl(((methyldiphenylphosphane)boryl)(4-methoxyphenyl)methyl)phosphonate(3da)

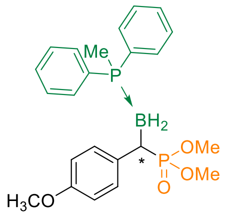

3da

Following the general procedure D, dimethyl(diazo(4-methoxyphenyl)methyl)phosphonate (0.20 mmol, 1.0 equiv) and methyldiphenylphosphane borane (0.40 mmol, 2.0 equiv) were employed and stirred at 20 °C for 12 h to afford **3da** as a colorless oil in 96% yield (85.3 mg) with 90% ee.

$\mathbf{R_f}$ = 0.50 (silica gel, EtOAc:PE = 3:1).

^{1}H NMR (400 MHz, Chloroform-*d*) δ 7.52 – 7.37 (m, 8H), 7.36 – 7.28 (m, 2H), 7.03 (dd, *J* = 8.6, 2.6 Hz, 2H), 6.65 (d, *J* = 8.3 Hz, 2H), 3.72 (s, 3H), 3.59 (dd, *J* = 15.1, 10.5 Hz, 6H), 2.46 – 2.30 (m, 1H),

1.34 (d, J = 10.2 Hz, 3H).

^{13}C NMR (101 MHz, Chloroform-*d*) δ 157.2 (d, J = 3.5 Hz), 133.1 (dd, J = 8.9, 4.8 Hz), 131.8 (dd, J = 18.3, 8.8 Hz), 131.1 (dd, J = 22.4, 2.5 Hz), 130.2 (d, J = 7.6 Hz), 129.7 (d, J = 57.7 Hz), 128.7 (dd, J = 23.6, 9.9 Hz), 127.9 (d, J = 55.6 Hz), 113.4 (d, J = 2.8 Hz), 55.1, 52.6 (dd, J = 48.4, 7.0 Hz), 9.4 (d, J = 36.9 Hz).

^{11}B NMR (128 MHz, Chloroform-*d*) δ -26.16.

^{31}P NMR (162 MHz, Chloroform-*d*) δ 39.09 (d, J = 84.4 Hz), 5.76.

HRMS (ESI): calc'd for $(M+H)^+$ $C_{23}H_{30}BO_4P_2^+$ 443.1707, found 443.1707.

HPLC analysis: DAICEL CHIRALCEL IA-3, hexane/isopropanol = 80/20, 1 mL/min, λ = 254 nm, t_R (major) = 14.597 min, t_R (minor) = 12.354 min, 90% ee.

$[\alpha]^{25}_D$: +51.4 (*c* 0.5, $CHCl_3$).

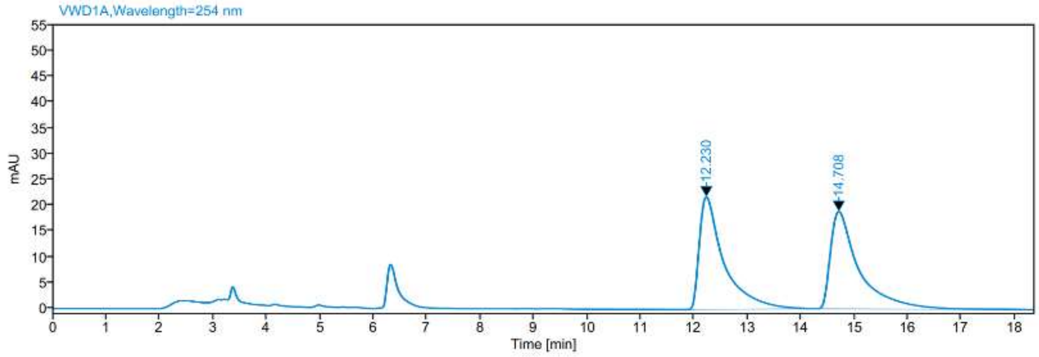

Signal: VWD1A,Wavelength=254 nm

RT [min]	Type	Width [min]	Area	Height	Area%	Name
12.230	BB	2.49	700.66	21.86	49.79	
14.708	BB	4.37	706.44	18.92	50.21	
		Sum	1407.10			

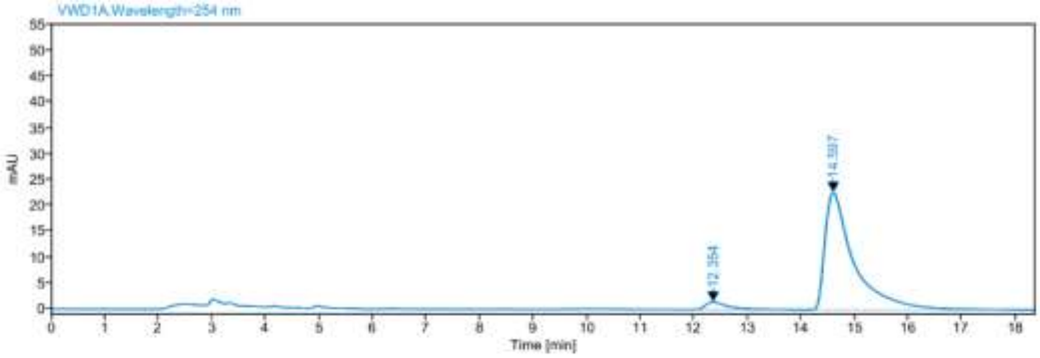

Signal: VWD1A,Wavelength=254 nm

RT [min]	Type	Width [min]	Area	Height	Area%	Name
12.354	MM m	1.78	44.68	1.45	4.95	
14.597	BB	4.55	857.27	22.71	95.05	
		Sum	901.95			

(S)-dimethyl(((methyldiphenylphosphane)boryl)(p-tolyl)methyl)phosphonate(3ea)

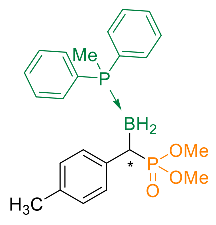

3ea

Following the general procedure D, dimethyl (diazo(p-tolyl)methyl)phosphonate (0.20 mmol, 1.0 equiv) and methyldiphenylphosphane borane (0.40 mmol, 2.0 equiv) were employed and stirred at 20 °C for 12 h to afford **3ea** as a colorless oil in 74% yield (63.3 mg) with 94% ee.

$\mathbf{R}_f$ = 0.63 (silica gel, EtOAc:PE = 3:1).

^{1}H NMR (500 MHz, Chloroform-*d*) δ 7.51 – 7.39 (m, 8H), 7.35 – 7.30 (m, 2H), 7.00 (dd, *J* = 8.1, 2.4 Hz, 2H), 6.90 (d, *J* = 7.7 Hz, 2H), 3.60 (dd, *J* = 10.5, 8.7 Hz, 6H), 2.48 – 2.35 (m, 1H), 2.24 (s, 3H), 1.33 (d, *J* = 10.3 Hz, 3H).

^{13}C NMR (126 MHz, Chloroform-*d*) δ 138.1 (dd, *J* = 8.3, 5.7 Hz), 134.3 (d, *J* = 3.6 Hz), 131.8 (dd, *J* = 26.4, 8.8 Hz), 131.0 (d, *J* = 32.2 Hz), 129.8 (d, *J* = 58.0 Hz), 129.2 (d, *J* = 7.6 Hz), 129.1 – 128.4 (m, 3C), 128.0 (d, *J* = 55.2 Hz), 52.6 (dd, *J* = 64.8, 6.8 Hz), 20.9, 9.3 (d, *J* = 36.7 Hz).

^{11}B NMR (128 MHz, Chloroform-*d*) δ -26.02.

^{31}P NMR (202 MHz, Chloroform-*d*) δ 39.64 (d, *J* = 84.2 Hz), 6.39.

HRMS (ESI): calcd for $(M+H)^+$ $C_{23}H_{30}BO_3P_2^+$ 427.1758, found 427.1758.

HPLC analysis: DAICEL CHIRALCEL IA-3, hexane/isopropanol = 92/08, 1 mL/min, λ = 254 nm, t_R (major) = 36.832 min, t_R (minor) = 32.049 min, 94% ee.

$[\alpha]^{25}_D$: +76.8 (*c* 0.5, $CHCl_3$).

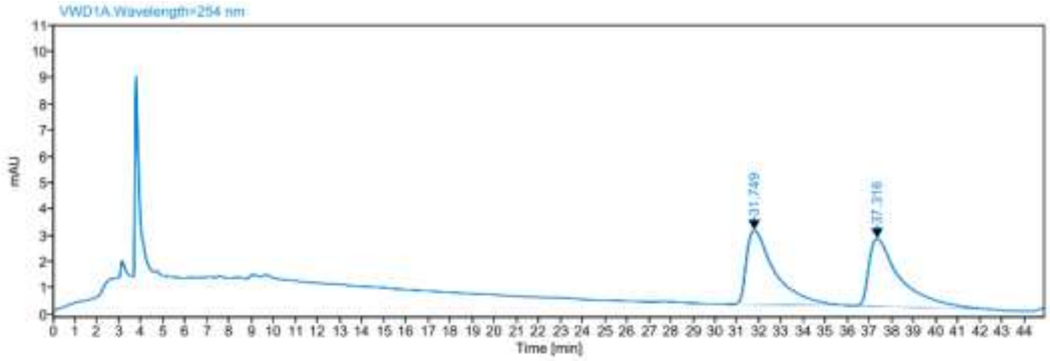

Signal: VWD1A,Wavelength=254 nm

RT [min]	Type	Width [min]	Area	Height	Area%	Name
31.749	BB	5.27	252.09	2.82	49.94	
37.316	BB	5.94	252.69	2.57	50.06	
		Sum	504.78			

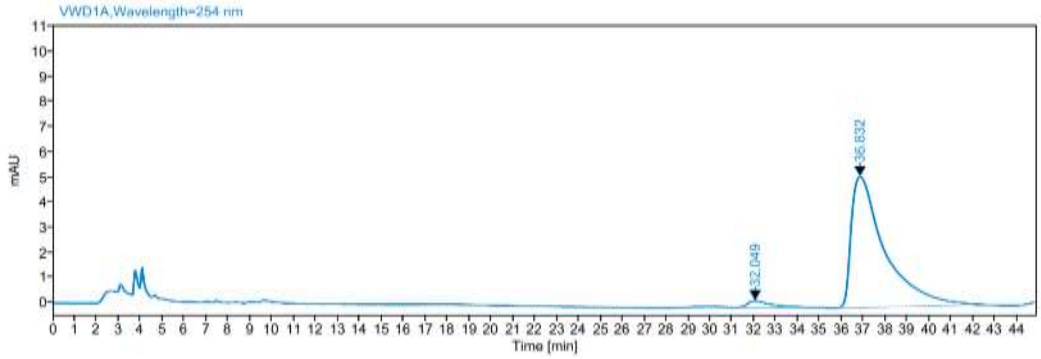

Signal: VWD1A,Wavelength=254 nm

RT [min]	Type	Width [min]	Area	Height	Area%	Name
32.049	MM m	3.46	18.50	0.26	3.21	
36.832	BB	7.06	556.96	5.22	96.79	
		Sum	575.45			

(S)-dimethyl(((methyldiphenylphosphane)boryl)(4-(methylthio))methyl)phosphonate(3fa)

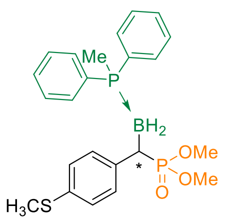

3fa

Following the general procedure D, dimethyl (diazo(4-(methylthio)phenyl)methyl) phosphonate (0.20 mmol, 1.0 equiv) and methyldiphenylphosphane borane (0.40 mmol, 2.0 equiv) were employed and stirred at 20 °C for 36 h to afford **3fa** as a yellow oil in 44% yield (40.6 mg) with 92% ee.

$\mathbf{R}_f$ = 0.58 (silica gel, EtOAc:PE = 3:1).

^{1}H NMR (400 MHz, Chloroform-*d*) δ 7.52 – 7.38 (m, 8H), 7.37 – 7.29 (m, 2H), 7.07 – 6.97 (m, 4H), 3.60 (t, *J* = 10.8 Hz, 6H), 2.41 (s, 4H), 1.40 (d, *J* = 10.2 Hz, 3H).

^{13}C NMR (101 MHz, Chloroform-*d*) δ 138.7 (dd, *J* = 8.6, 5.6 Hz), 134.1 (d, *J* = 4.0 Hz), 131.8 (dd, *J* = 20.9, 8.9 Hz), 131.1 (dd, *J* = 23.1, 2.5 Hz), 129.9 (d, *J* = 7.8 Hz), 129.5 (d, *J* = 58.4 Hz), 128.8 (dd, *J* = 21.0, 10.0 Hz), 127.9 (d, *J* = 55.6 Hz), 126.9 (d, *J* = 2.8 Hz), 52.7 (dd, *J* = 49.3, 7.0 Hz), 16.3, 9.5 (d, *J* = 37.2 Hz).

^{11}B NMR (128 MHz, Chloroform-*d*) δ -26.09.

^{31}P NMR (162 MHz, Chloroform-*d*) δ 38.44 (d, *J* = 82.2 Hz), 5.53.

HRMS (ESI): calcd for $(M+H)^+$ $C_{23}H_{30}BO_3P_2S^+$ 459.1478, found 459.1477.

HPLC analysis: DAICEL CHIRALCEL ID-3, hexane/isopropanol = 80/20, 1 mL/min, λ = 254 nm, t_R (major) = 52.985 min, t_R (minor) = 49.269 min, 92% ee.

$[\alpha]^{25}_D$: +79.0 (*c* 0.5, $CHCl_3$).

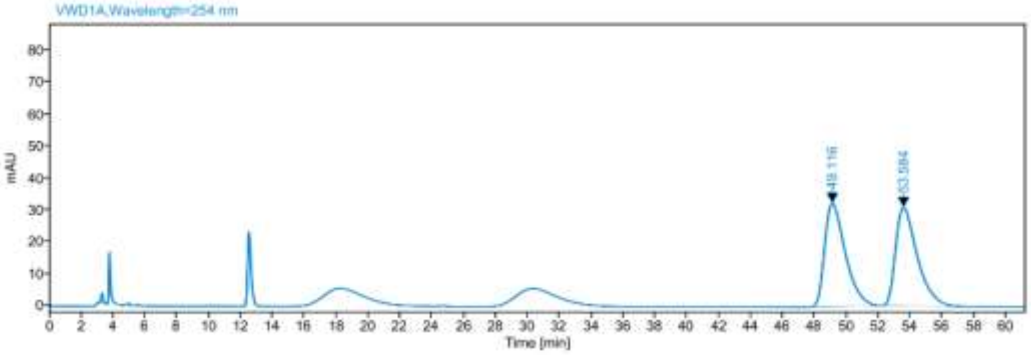

Signal: VWD1A,Wavelength=254 nm

RT [min]	Type	Width [min]	Area	Height	Area%	Name
49.116	BB	4.59	2917.99	32.34	49.93	
53.584	BB	5.81	2926.61	30.82	50.07	
		Sum	5844.60			

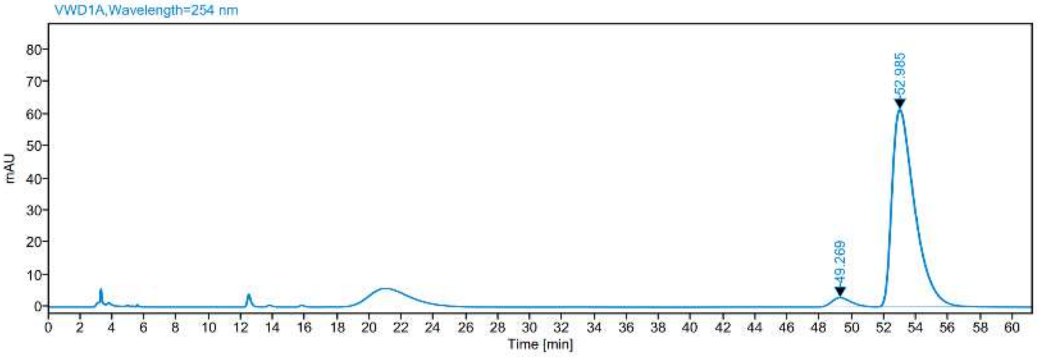

Signal: VWD1A,Wavelength=254 nm

RT [min]	Type	Width [min]	Area	Height	Area%	Name
49.269	BB	3.79	248.38	2.92	3.97	
52.985	BB	6.79	6015.18	61.43	96.03	
		Sum	6263.57			

(S)-dimethyl(((methyldiphenylphosphane)boryl)(4-nitrophenyl)methyl)phosphonate(3ga)

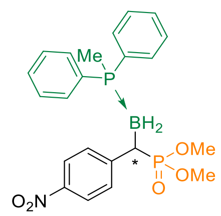

3ga

Following the general procedure D, dimethyl (diazo(4-nitrophenyl)methyl)phosphonate (0.20 mmol, 1.0 equiv) and methyldiphenylphosphane borane (0.40 mmol, 2.0 equiv) were employed and stirred at 20 °C for 36 h to afford **3ga** as a colorless oil in 70% yield (64.3 mg) with 96% ee.

R_f = 0.49 (silica gel, EtOAc:PE = 3:1).

^{1}H NMR (500 MHz, Chloroform-*d*) δ 7.89 – 7.84 (m, 2H), 7.50 – 7.36 (m, 8H), 7.35 – 7.30 (m, 2H), 7.25 – 7.20 (m, 2H), 3.63 (dd, *J* = 10.6, 6.2 Hz, 6H), 2.67 – 2.54 (m, 1H), 1.56 (d, *J* = 10.2 Hz, 3H).

^{13}C NMR (126 MHz, Chloroform-*d*) δ 150.8 (dd, *J* = 8.0, 6.1 Hz), 145.3 (d, *J* = 3.6 Hz), 131.7 (dd, *J* = 15.5, 9.0 Hz), 131.4 (dd, *J* = 21.4, 2.5 Hz), 129.7 (d, *J* = 7.9 Hz), 128.9 (dd, *J* = 19.4, 10.1 Hz), 128.4 (d, *J* = 58.2 Hz), 127.7 (d, *J* = 57.0 Hz), 123.1 (d, *J* = 2.4 Hz), 52.9 (dd, *J* = 55.4, 7.0 Hz), 9.6 (d, *J* = 38.2 Hz).

^{11}B NMR (160 MHz, Chloroform-*d*) δ -26.62.

^{31}P NMR (202 MHz, Chloroform-*d*) δ 36.86 (d, *J* = 73.4 Hz), 5.50.

HRMS (ESI): calcd for $(M+H)^+$ $C_{22}H_{27}BNO_5P_2^+$ 458.1452, found 458.1452.

HPLC analysis: DAICEL CHIRALCEL IA-3, hexane/isopropanol = 80/20, 1 mL/min, λ = 254 nm,

t_R (major) = 15.233 min, t_R (minor) = 12.811 min, 96% ee.

$[\alpha]^{25}_D$: +90.4 (*c* 0.5, $CHCl_3$).

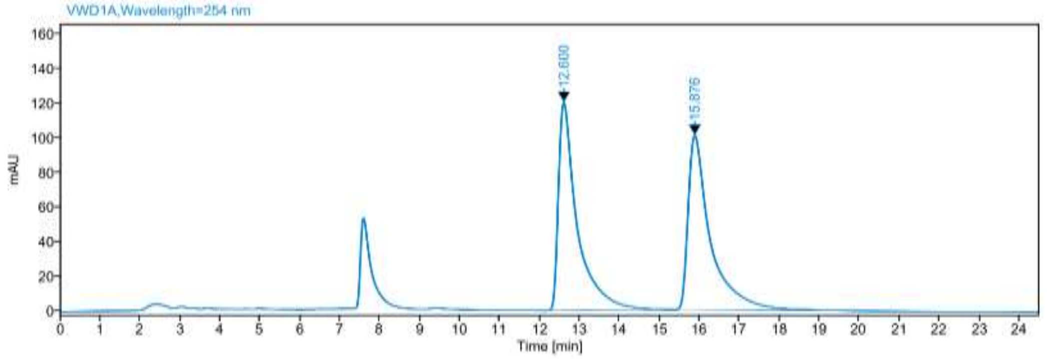

Signal: VWD1A,Wavelength=254 nm

RT [min]	Type	Width [min]	Area	Height	Area%	Name
12.600	BB	3.20	3916.24	119.70	49.97	
15.876	BB	5.93	3921.31	100.42	50.03	
		**Sum**	**7837.55**			

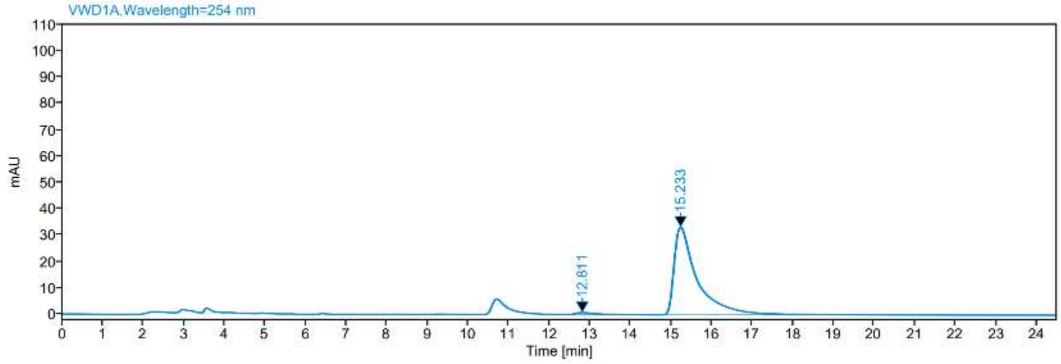

Signal: VWD1A,Wavelength=254 nm

RT [min]	Type	Width [min]	Area	Height	Area%	Name
12.811	MM m	1.71	25.51	0.78	1.90	
15.233	BB	4.65	1315.11	33.28	98.10	
		**Sum**	**1340.62**			

(S)-methyl-4-(((methyldiphenylphosphane)boryl) (dimethoxyphosphoryl)methyl)benzoate(3ha)

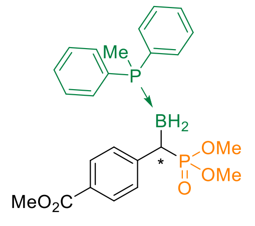

3ha

Following the general procedure D, methyl-4-(diazo(dimethoxyphosphoryl)methyl)benzoate (0.20 mmol, 1.0 equiv) and methyldiphenylphosphane borane (0.40 mmol, 2.0 equiv) were employed and stirred at 20 °C for 12 h to afford **3ha** as a colorless oil in 80% yield (75.6 mg) with 94% ee.

R_f = 0.49 (silica gel, EtOAc:PE = 3:1).

^{1}H NMR (500 MHz, Chloroform-*d*) δ 7.74 (d, *J* = 8.5 Hz, 2H), 7.50 – 7.37 (m, 8H), 7.33 – 7.28 (m, 2H), 7.16 (dd, *J* = 8.4, 2.3 Hz, 2H), 3.85 (d, *J* = 1.2 Hz, 3H), 3.62 – 3.55 (m, 6H), 2.59 – 2.46 (m, 1H), 1.35 (d, *J* = 10.2 Hz, 3H).

^{13}C NMR (126 MHz, Chloroform-*d*) δ 167.2, 147.8 (dd, *J* = 8.1, 5.5 Hz), 131.7 (dd, *J* = 23.7, 8.9 Hz), 131.2 (dd, *J* = 29.3, 2.6 Hz), 129.2, 129.2, 128.8 (dd, *J* = 30.0, 9.9 Hz), 127.5 (d, *J* = 56.0 Hz), 126.7 (d, *J* = 3.4 Hz), 52.7 (dd, *J* = 64.3, 7.0 Hz), 51.8, 9.4 (d, *J* = 37.4 Hz).

^{11}B NMR (160 MHz, Chloroform-*d*) δ -26.79.

^{31}P NMR (202 MHz, Chloroform-*d*) δ 37.99 (d, *J* = 80.3 Hz), 5.89.

HRMS (ESI): calc'd for $(M+H)^+$ $C_{24}H_{30}BO_5P_2^+$ 471.1656, found 471.1656.

HPLC analysis: DAICEL CHIRALCEL AD-H, hexane/isopropanol = 80/20, 1 mL/min, λ = 254 nm, t_R (major) = 20.630 min, t_R (minor) = 14.240 min, 94% ee.

$[\alpha]^{25}_D$: +95.8 (*c* 0.5, $CHCl_3$).

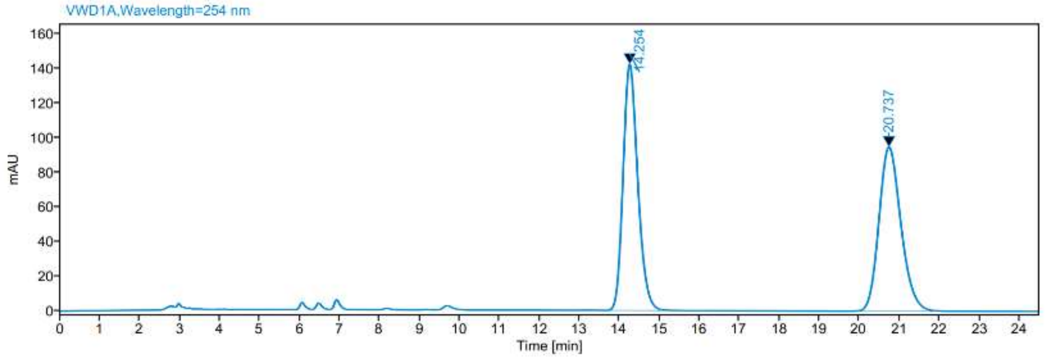

Signal: VWD1A,Wavelength=254 nm

RT [min]	Type	Width [min]	Area	Height	Area%	Name
14.254	BB	2.20	3615.81	141.62	50.02	
20.737	BB	3.36	3613.47	94.40	49.98	
		Sum	7229.28			

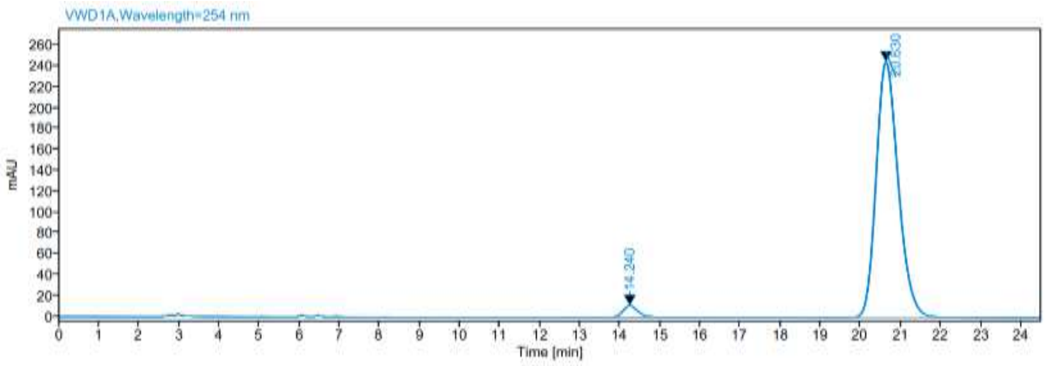

Signal: VWD1A,Wavelength=254 nm

RT [min]	Type	Width [min]	Area	Height	Area%	Name
14.240	BB	1.72	287.74	11.23	2.98	
20.630	BB	3.42	9359.54	244.57	97.02	
		**Sum**	**9647.28**			

(S)-dimethyl(((methyldiphenylphosphane)boryl)(4-fluorophenyl)methyl)phosphonate(3ia)

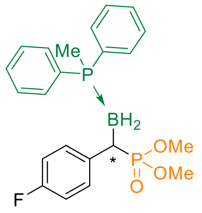

3ia

Following the general procedure D, dimethyl (diazo(4-fluorophenyl)methyl)phosphonate (0.20 mmol, 1.0 equiv) and methyldiphenylphosphane borane (0.40 mmol, 2.0 equiv) were employed and stirred at 20 °C for 12 h to afford **3ia** as a orange solid in 76% yield (65.5 mg) with 92% ee.

mp: 149.3 – 150.2 °C

R_f = 0.51 (silica gel, EtOAc:PE = 3:1).

^{1}H NMR (400 MHz, Chloroform-*d*) δ 7.52 – 7.37 (m, 8H), 7.37 – 7.30 (m, 2H), 7.11 – 7.03 (m, 2H), 6.76 (t, *J* = 8.6 Hz, 2H), 3.66 – 3.53 (m, 6H), 2.52 – 2.35 (m, 1H), 1.40 (d, *J* = 10.2 Hz, 3H).

^{13}C NMR (101 MHz, Chloroform-*d*) δ 160.7 (dd, *J* = 242.9, 3.8 Hz), 137.0, 131.7 (dd, *J* = 17.5, 8.9 Hz), 131.2 (dd, *J* = 21.0, 2.5 Hz), 130.6 (t, *J* = 7.6 Hz), 128.8 (dd, *J* = 21.3, 10.1 Hz), 128.6 (dd, *J* = 148.0, 56.7 Hz), 114.6 (dd, *J* = 20.9, 2.8 Hz), 52.7 (dd, *J* = 47.4, 7.0 Hz), 9.5 (d, *J* = 37.3 Hz).

^{11}B NMR (128 MHz, Chloroform-*d*) δ -25.98.

^{31}P NMR (162 MHz, Chloroform-*d*) δ 38.37 (dd, *J* = 82.2, 5.7 Hz), 5.50.

^{19}F NMR (376 MHz, Chloroform-*d*) δ -118.73 (t, *J* = 4.6 Hz).

HRMS (ESI): calc'd for $(M+H)^+$ $C_{22}H_{27}BFO_3P_2^+$ 431.1507, found 431.1507.

HPLC analysis: DAICEL CHIRALCEL AD-H, hexane/isopropanol = 80/20, 1 mL/min, λ = 254 nm, t_R (major) = 9.875 min, t_R (minor) = 8.944 min, 92% ee.
$[\alpha]^{25}_D$: +53.0 (*c* 0.5, $CHCl_3$).

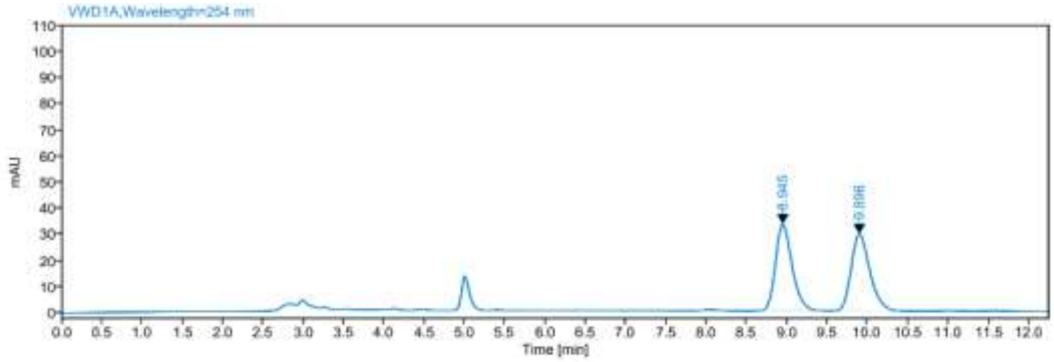

Signal: VWD1A,Wavelength=254 nm

RT [min]	Type	Width [min]	Area	Height	Area%	Name
8.945	BB	1.06	477.88	33.04	50.09	
9.896	BB	1.15	476.23	29.28	49.91	
		Sum	954.11			

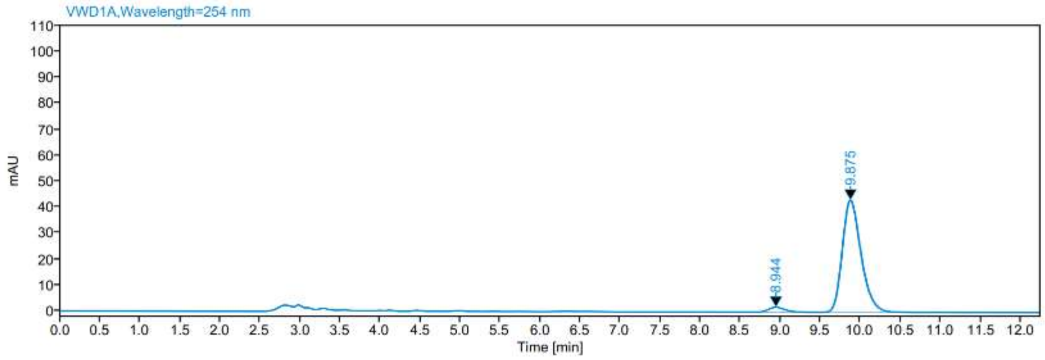

Signal: VWD1A,Wavelength=254 nm

RT [min]	Type	Width [min]	Area	Height	Area%	Name
8.944	BB	0.86	27.76	1.92	3.79	
9.875	BB	1.30	704.36	43.26	96.21	
		Sum	732.12			

(S)-dimethy(((methyldiphenylphosphane)boryl)(4-bromophenyl)methyl)phosphonate(3ja)

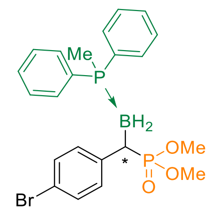

3ja

Following the general procedure D, dimethyl ((4-bromophenyl)(diazo)methyl)phosphonate (0.20

mmol, 1.0 equiv) and methyldiphenylphosphane borane (0.40 mmol, 2.0 equiv) were employed and stirred at 20 °C for 12 h to afford **3ja** as a white solid in 76% yield (74.4 mg) with 94% ee.

mp: 132.0 –132.9 °C

R$_f$ = 0.58 (silica gel, EtOAc:PE = 3:1).

^{1}H NMR (500 MHz, Chloroform-*d*) δ 7.51 – 7.38 (m, 8H), 7.36 – 7.31 (m, 2H), 7.17 – 7.13 (m, 2H), 7.00 – 6.96 (m, 2H), 3.60 (dd, *J* = 13.6, 10.5 Hz, 6H), 2.48 – 2.36 (m, 1H), 1.45 (d, *J* = 10.2 Hz, 3H).

^{13}C NMR (126 MHz, Chloroform-*d*) δ 140.7 (dd, *J* = 9.0, 5.5 Hz), 131.7 (dd, *J* = 24.9, 9.0 Hz), 131.3 (d, *J* = 2.6 Hz), 131.1, 131.0 (d, *J* = 5.3 Hz), 130.9 (d, *J* = 2.8 Hz), 128.8 (dd, *J* = 23.7, 10.0 Hz), 128.4 (dd, *J* = 162.0, 57.2 Hz), 118.6 (d, *J* = 4.4 Hz), 52.7 (dd, *J* = 61.1, 7.0 Hz), 9.5 (d, *J* = 37.3 Hz).

^{11}B NMR (160 MHz, Chloroform-*d*) δ -26.68.

^{31}P NMR (202 MHz, Chloroform-*d*) δ 38.33 (d, *J* = 78.7 Hz), 5.98.

HRMS (ESI): calc'd for $(M+H)^+$ $C_{22}H_{27}BBrO_3P_2^+$ 491.0706, found 491.0706.

HPLC analysis: DAICEL CHIRALCEL ID-3, hexane/isopropanol = 80/20, 1 mL/min, λ = 254 nm, t_R (major) = 25.949 min, t_R (minor) = 28.827 min, 94% ee.

[α]$^{25}_D$: +92.0 (*c* 0.5, $CHCl_3$).

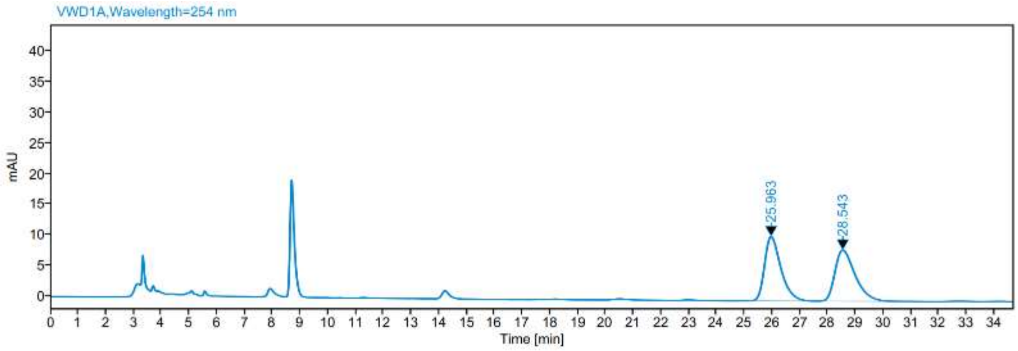

Signal: VWD1A,Wavelength=254 nm

RT [min]	Type	Width [min]	Area	Height	Area%	Name
25.963	BB	2.51	427.93	10.48	50.36	
28.543	BB	3.15	421.83	8.33	49.64	
		Sum	849.76			

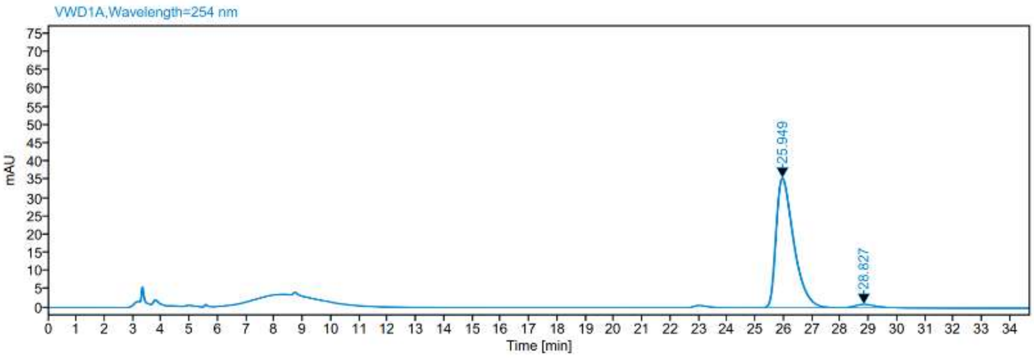

Signal:	VWD1A,Wavelength=254 nm					
RT [min]	Type	Width [min]	Area	Height	Area%	Name
25.949	BB	3.13	1533.38	35.49	97.15	
28.827	MM m	2.23	44.99	0.92	2.85	
		Sum	1578.37			

(S)-dimethyl(((methyldiphenylphosphane)boryl)(4-(trifluoromethyl)phenyl)methyl) phosphonate(3ka)

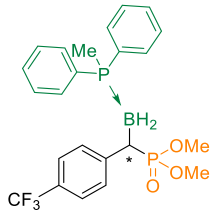

3ka

Following the general procedure D, dimethyl (diazo(4-(trifluoromethyl)phenyl)methyl) phosphonate (0.20 mmol, 1.0 equiv) and methyldiphenylphosphane borane (0.40 mmol, 2.0 equiv) were employed and stirred at 20 °C for 36 h to afford **3ka** as a colorless oil in 83% yield (80.0 mg) with 96% ee.

R_f = 0.47 (silica gel, EtOAc:PE = 3:1).

^{1}H NMR (400 MHz, Chloroform-*d*) δ 7.51 – 7.37 (m, 8H), 7.38 – 7.25 (m, 4H), 7.21 (d, *J* = 6.1 Hz, 2H), 3.63 (t, *J* = 9.9 Hz, 6H), 2.66 – 2.48 (m, 1H), 1.52 (d, *J* = 10.2 Hz, 3H).

^{13}C NMR (101 MHz, Chloroform-*d*) δ 146.2, 131.7 (dd, *J* = 19.7, 9.0 Hz), 131.2 (dd, *J* = 17.6, 2.5 Hz), 129.4 (d, *J* = 7.8 Hz), 128.8 (dd, *J* = 14.9, 10.0 Hz), 128.3 (dd, *J* = 83.4, 57.2 Hz), 127.0 (dd, *J* = 32.2, 3.6 Hz), 124.7 (q, *J* = 3.5 Hz), 124.4 (d, *J* = 271.1 Hz), 52.8 (dd, *J* = 46.0, 7.1 Hz), 9.5 (d, *J* = 37.8 Hz).

^{11}B NMR (128 MHz, Chloroform-*d*) δ -26.03.

^{31}P NMR (162 MHz, Chloroform-*d*) δ 37.34 (d, *J* = 78.7 Hz), 5.15.

^{19}F NMR (376 MHz, Chloroform-*d*) δ -62.18 (d, *J* = 2.8 Hz).

HRMS (ESI): calc'd for $(M+H)^+$ $C_{23}H_{27}BF_3O_3P_2^+$ 481.1475, found 481.1474.

HPLC analysis: DAICEL CHIRALCEL ID-3, hexane/isopropanol = 85/15, 1 mL/min, λ = 254 nm, t_R (major) = 20.838 min, t_R (minor) = 22.634 min, 96% ee.

$[\alpha]^{25}_D$: +51.4 (*c* 0.5, $CHCl_3$).

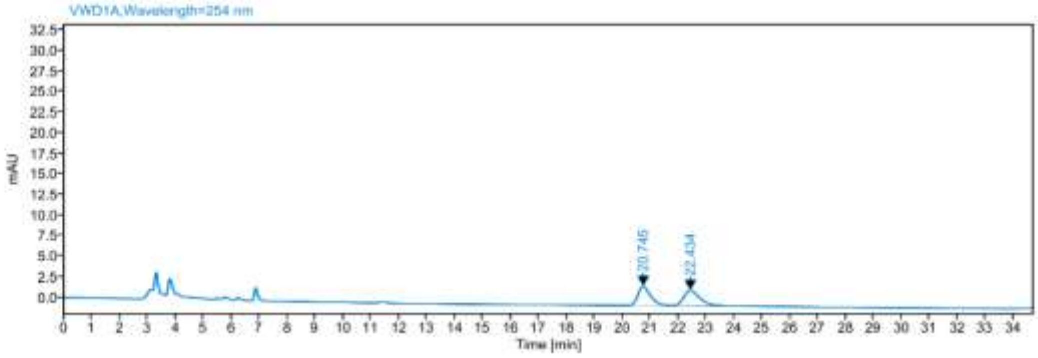

Signal: VWD1A,Wavelength=254 nm

RT [min]	Type	Width [min]	Area	Height	Area%	Name
20.745	BB	1.63	72.49	2.33	49.41	
22.434	BB	2.25	74.22	1.86	50.59	
		Sum	146.71			

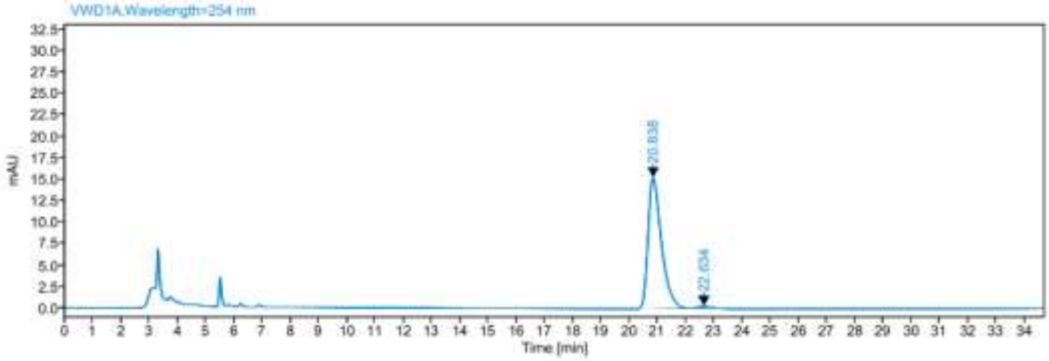

Signal: VWD1A,Wavelength=254 nm

RT [min]	Type	Width [min]	Area	Height	Area%	Name
20.838	BB	1.92	503.11	15.26	98.35	
22.634	MM m	1.38	8.45	0.25	1.65	
		Sum	511.56			

(S)-dimethyl(((methyldiphenylphosphane)boryl)([1,1'-biphenyl]-4-yl)methyl) phosphonate(3la)

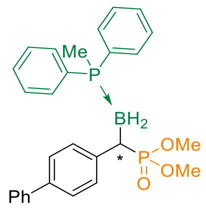

3la

Following the general procedure D, dimethyl ([1,1'-biphenyl]-4-yl(diazo)methyl)phosphonate (0.20 mmol, 1.0 equiv) and methyldiphenylphosphane borane (0.40 mmol, 2.0 equiv) were employed and stirred at 20 °C for 12 h to afford **3la** as a colorless oil in 87% yield (84.9 mg) with 93% ee.

R*f* = 0.47 (silica gel, EtOAc:PE = 3:1).

^{1}H NMR (400 MHz, Chloroform-*d*) δ 7.56 – 7.37 (m, 12H), 7.37 – 7.25 (m, 5H), 7.19 (dd, *J* = 8.3, 2.5 Hz, 2H), 3.64 (t, *J* = 9.9 Hz, 6H), 2.62 – 2.45 (m, 1H), 1.43 (d, *J* = 10.2 Hz, 3H).

^{13}C NMR (101 MHz, Chloroform-*d*) δ 141.0 (d, *J* = 1.5 Hz), 140.7 (dd, *J* = 8.3, 5.8 Hz), 137.6 (d, *J* = 3.7 Hz), 131.8 (dd, *J* = 23.1, 8.9 Hz), 131.0 (dd, *J* = 25.6, 2.5 Hz), 129.7 (d, *J* = 7.8 Hz), 129.2, 128.7 (dd, *J* = 21.0, 10.0 Hz), 128.6, 128.0 (d, *J* = 55.7 Hz), 126.7, 126.7, 126.5 (d, *J* = 2.9 Hz), 52.7 (dd, *J* = 48.9, 7.0 Hz), 9.4 (d, *J* = 37.0 Hz).

^{11}B NMR (128 MHz, Chloroform-*d*) δ -25.86.

^{31}P NMR (162 MHz, Chloroform-*d*) δ 38.54 (d, *J* = 81.9 Hz), 5.57.

HRMS (ESI): calc'd for $(M+H)^+$ $C_{28}H_{32}BO_3P_2^+$ 489.1914, found 489.1914.

HPLC analysis: DAICEL CHIRALCEL AD-H, hexane/isopropanol = 70/30, 1 mL/min, λ = 254 nm, t_R (major) = 14.542 min, t_R (minor) = 8.101 min, 93% ee.

$[\alpha]^{25}_D$: +84.5 (*c* 0.5, $CHCl_3$).

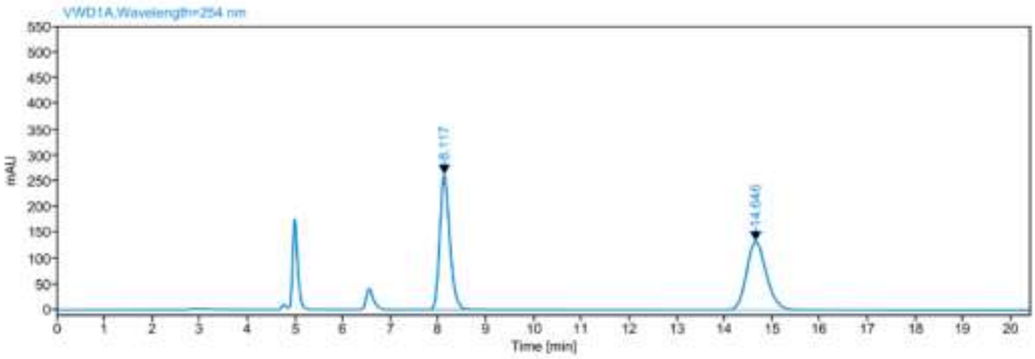

Signal: VWD1A,Wavelength=254 nm

RT [min]	Type	Width [min]	Area	Height	Area%	Name
8.117	BB	2.24	3840.68	261.37	50.03	
14.646	BB	2.60	3836.14	132.58	49.97	
		Sum	7676.82			

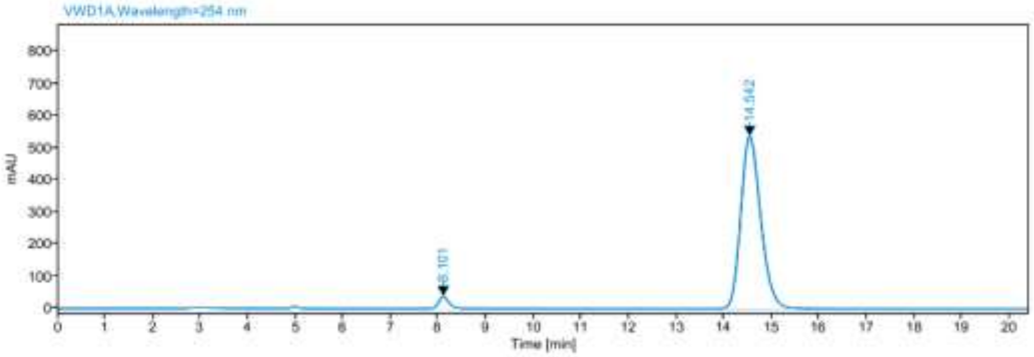

Signal: VWD1A,Wavelength=254 nm

RT [min]	Type	Width [min]	Area	Height	Area%	Name
8.101	BB	1.61	561.36	37.60	3.49	
14.542	BB	2.99	15539.46	535.21	96.51	
		Sum	16100.81			

(S)-dimethyl(((methyldiphenylphosphane)boryl)(4-(benzyloxy)phenyl)methyl) phosphonate(3ma)

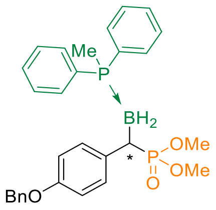

3ma

Following the general procedure D, dimethyl ((4-(benzyloxy)phenyl)(diazo)methyl)phosphonate (0.20 mmol, 1.0 equiv) and methyldiphenylphosphane borane (0.40 mmol, 2.0 equiv) were employed and stirred at 20 °C for 12 h to afford **3ma** as a colorless oil in 85% yield (88.1 mg) with 90% ee.

$\mathbf{R_f}$ = 0.37 (silica gel, EtOAc:PE = 3:1).

^{1}H NMR (400 MHz, Chloroform-*d*) δ 7.52 – 7.27 (m, 15H), 7.03 (dd, *J* = 8.6, 2.6 Hz, 2H), 6.74 (d, *J* = 8.2 Hz, 2H), 4.99 (s, 2H), 3.60 (dd, *J* = 16.3, 10.4 Hz, 6H), 2.49 – 2.32 (m, 1H), 1.32 (d, *J* = 10.2 Hz, 3H).

^{13}C NMR (101 MHz, Chloroform-*d*) δ 156.4 (d, *J* = 3.5 Hz), 137.2, 133.3 (dd, *J* = 8.9, 5.7 Hz), 131.7 (dd, *J* = 19.1, 8.9 Hz), 131.0 (dd, *J* = 22.4, 2.5 Hz), 130.2 (d, *J* = 7.6 Hz), 129.6 (d, *J* = 58.4 Hz), 128.7 (dd, *J* = 23.0, 10.0 Hz), 128.4, 127.8 (d, *J* = 55.3 Hz), 127.7, 127.4, 114.5 (d, *J* = 2.9 Hz), 69.8, 52.6 (dd, *J* = 48.1, 7.0 Hz), 9.3 (d, *J* = 36.8 Hz).

^{11}B NMR (128 MHz, Chloroform-*d*) δ -25.98.

^{31}P NMR (162 MHz, Chloroform-*d*) δ 39.14 (d, *J* = 85.2 Hz), 5.75.

HRMS (ESI): calc'd for $(M+H)^+$ $C_{29}H_{34}BO_4P_2^+$ 519.2020 found 519.2020.

HPLC analysis: DAICEL CHIRALCEL AD-H, hexane/isopropanol = 80/20, 1 mL/min, λ = 254 nm, t_R (major) = 17.916 min, t_R (minor) = 15.814 min, 90% ee.

$[\alpha]^{25}_{D}$: +76.4(*c* 0.5, $CHCl_3$).

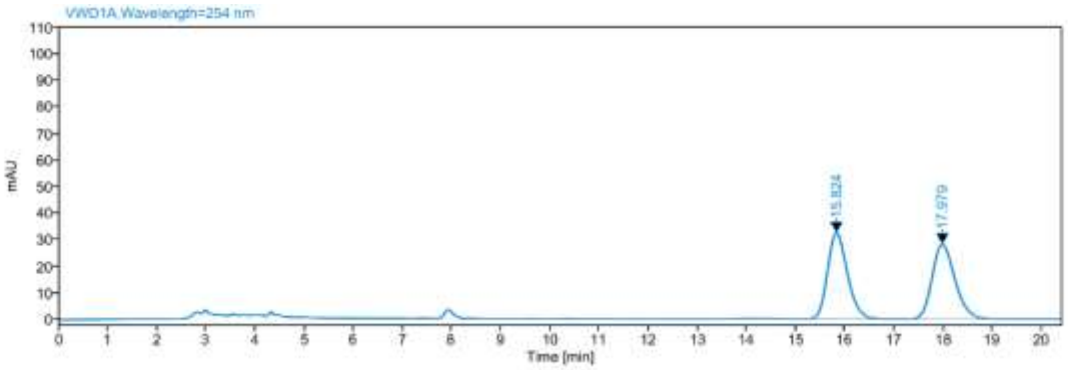

Signal: VWD1A,Wavelength=254 nm

RT [min]	Type	Width [min]	Area	Height	Area%	Name
15.824	BB	1.99	909.69	32.39	49.98	
17.979	BB	2.41	910.25	28.13	50.02	
		Sum	1819.94			

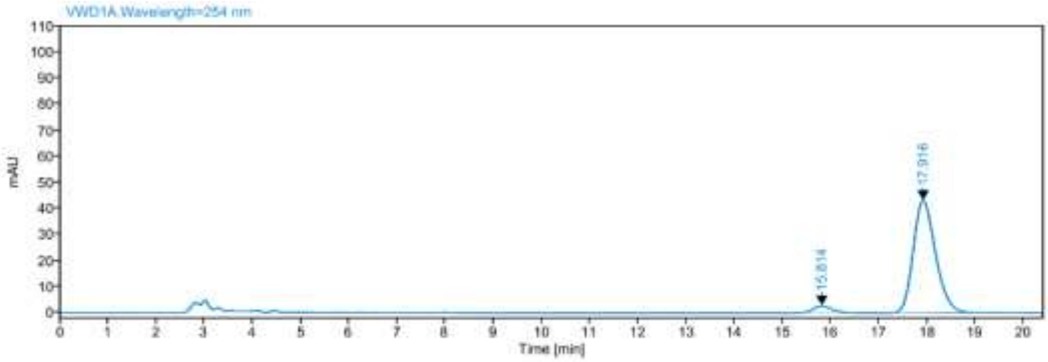

Signal:	VWD1A,Wavelength=254 nm					
RT [min]	Type	Width [min]	Area	Height	Area%	Name
15.814	BB	1.53	74.00	2.67	5.07	
17.916	BB	1.91	1384.51	43.02	94.93	
		Sum	1458.52			

(S)-dimethy(((methyldiphenylphosphane)boryl)(4-cyanophenyl)methyl)phosphonate(3na)

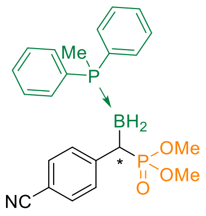

3na

Following the general procedure D, dimethyl ((4-cyanophenyl)(diazo)methyl)phosphonate (0.20 mmol, 1.0 equiv) and methyldiphenylphosphane borane (0.40 mmol, 2.0 equiv) were employed and stirred at 20 °C for 48 h to afford **3na** as a white solid in 80% yield (70.3 mg) with 96% ee.

mp: 110.2 –111.5 ℃

R*f* = 0.33 (silica gel, EtOAc:PE = 3:1).

^{1}H NMR (500 MHz, Chloroform-*d*) δ 7.51 – 7.37 (m, 8H), 7.36 – 7.28 (m, 4H), 7.18 (dd, *J* = 8.3, 2.3 Hz, 2H), 3.61 (t, *J* = 9.6 Hz, 6H), 2.59 – 2.46 (m, 1H), 1.52 (d, *J* = 10.2 Hz, 3H).

^{13}C NMR (126 MHz, Chloroform-*d*) δ 148.2 (t, *J* = 7.3 Hz), 131.7 (d, *J* = 8.8 Hz), 131.6 (d, *J* = 8.9 Hz), 131.3 (dd, *J* = 21.5, 2.5 Hz), 129.9 (d, *J* = 7.7 Hz), 128.9 (dd, *J* = 19.0, 10.0 Hz), 128.1 (dd, *J* = 102.2, 57.6 Hz), 119.3, 108.3, 52.8 (dd, *J* = 55.3, 6.9 Hz), 9.5 (d, *J* = 38.0 Hz).

^{11}B NMR (128 MHz, Chloroform-*d*) δ -26.18.

^{31}P NMR (202 MHz, Chloroform-*d*) δ 37.21 (d, *J* = 75.1 Hz), 5.60.

HRMS (ESI): calc'd for $(M+H)^+$ $C_{23}H_{27}BNO_3P_2^+$ 438.1554, found 438.1554.

HPLC analysis: DAICEL CHIRALCEL AD-H, hexane/isopropanol = 80/20, 1 mL/min, λ = 254 nm, t_R (major) = 16.937 min, t_R (minor) = 15.614 min, 96% ee.

[α]^{25}D: +48.2 (*c* 0.5, $CHCl_3$).

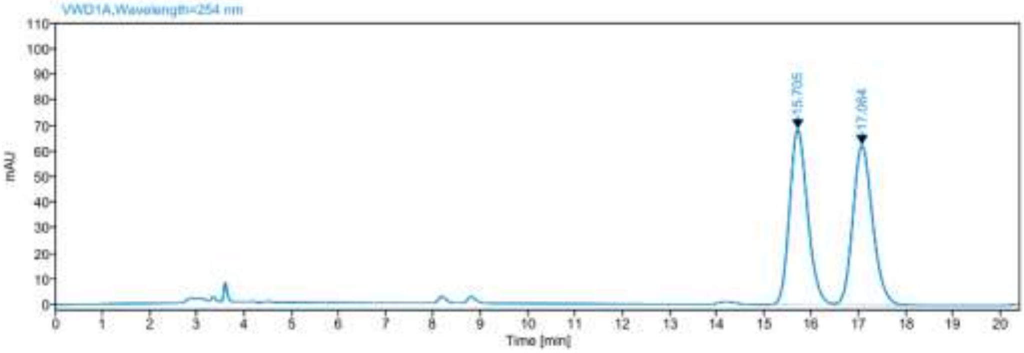

Signal: VWD1A,Wavelength=254 nm

RT [min]	Type	Width [min]	Area	Height	Area%	Name
15.705	BV	1.45	1813.97	68.51	49.95	
17.064	VB	3.01	1817.65	62.43	50.05	
		Sum	3631.62			

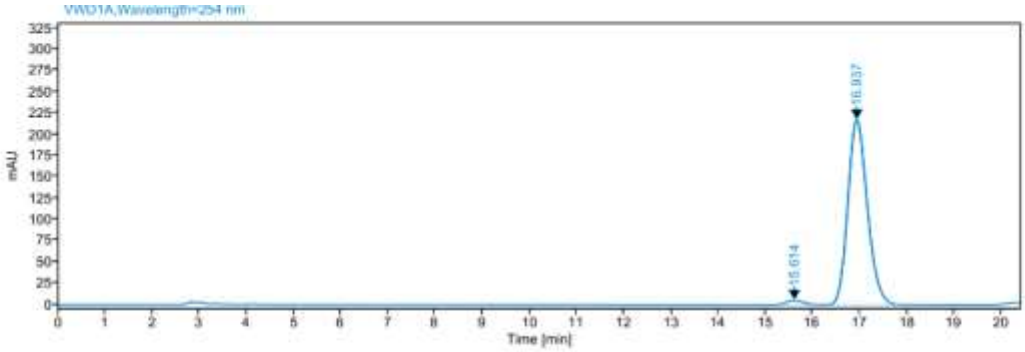

Signal: VWD1A,Wavelength=254 nm

RT [min]	Type	Width [min]	Area	Height	Area%	Name
15.614	BB	1.21	138.96	5.44	2.17	
16.937	BB	2.40	6263.30	217.46	97.83	
		Sum	6402.26			

(S)-dimethy(((methyldiphenylphosphane)boryl)(3-methoxyphenyl)methyl)phosphonate(3oa)

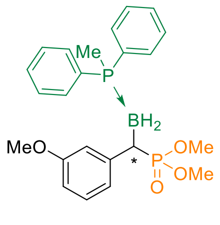

3oa

Following the general procedure D, dimethyl ((3-methoxyphenyl)(diazo)methyl)phosphonate (0.20 mmol, 1.0 equiv) and methyldiphenylphosphane borane (0.40 mmol, 2.0 equiv) were employed and stirred at 20 °C for 12 h to afford **3oa** as a colorless oil in 88% yield (78.2 mg) with 94% ee.

R*f* = 0.33 (silica gel, EtOAc:PE = 3:1)

^{1}H NMR (400 MHz, Chloroform-*d*) δ 7.51 – 7.38 (m, 8H), 7.37 – 7.30 (m, 2H), 7.01 (t, *J* = 8.1 Hz, 1H), 6.69 (dd, *J* = 4.6, 2.3 Hz, 2H), 6.62 – 6.54 (m, 1H), 3.68 (s, 3H), 3.62 (t, *J* = 10.6 Hz, 6H), 2.54 – 2.36 (m, 1H), 1.35 (d, *J* = 10.3 Hz, 3H).

^{13}C NMR (101 MHz, Chloroform-*d*) δ 159.2 (d, *J* = 2.7 Hz), 142.7 (dd, *J* = 8.7, 5.1 Hz), 131.8 (dd, *J* = 24.5, 8.9 Hz), 131.2 (dd, *J* = 24.8, 2.5 Hz), 129.6 (d, *J* = 57.5 Hz), 128.8 (d, *J* = 2.6 Hz), 128.8 (dd, *J* = 25.4, 10.0 Hz), 127.8 (d, *J* = 55.4 Hz), 121.9 (d, *J* = 8.0 Hz), 114.7 (d, *J* = 7.7 Hz), 111.1 (d, *J* = 3.4 Hz), 55.0, 52.9 (dd, *J* = 48.8, 7.1 Hz), 9.3 (d, *J* = 37.0 Hz).

^{11}B NMR (128 MHz, Chloroform-*d*) δ -26.15.

^{31}P NMR (162 MHz, Chloroform-*d*) δ 38.78 (d, *J* = 84.8 Hz), 5.54.

HRMS (ESI): calc'd for $(M+H)^+$ $C_{23}H_{30}BO_4P_2^+$ 443.1707, found 443.1714.

HPLC analysis: DAICEL CHIRALCEL IA-3, hexane/isopropanol = 70/30, 1 mL/min, λ = 254 nm, t_R (major) = 13.742 min, t_R (minor) = 8.597 min, 94% ee.

$[\alpha]^{25}_D$: +42.8 (*c* 0.5, $CHCl_3$).

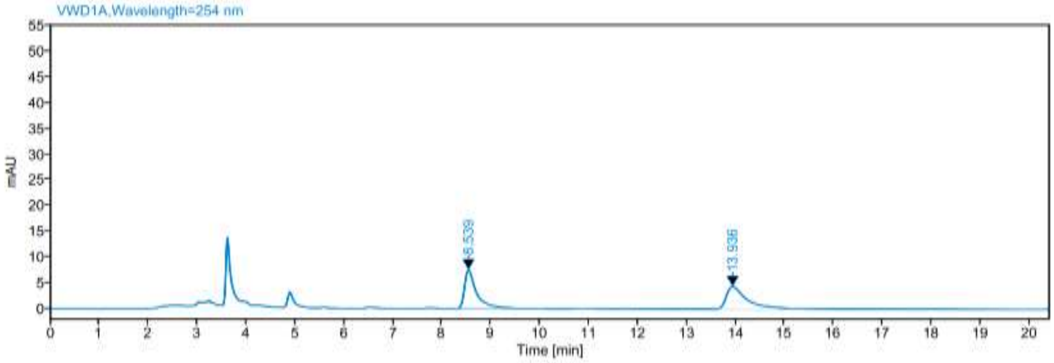

Signal: VWD1A,Wavelength=254 nm

RT [min]	Type	Width [min]	Area	Height	Area%	Name
8.539	MM m	2.27	129.68	7.49	49.95	
13.936	MM m	2.56	129.96	4.34	50.05	
		**Sum**	**259.64**			

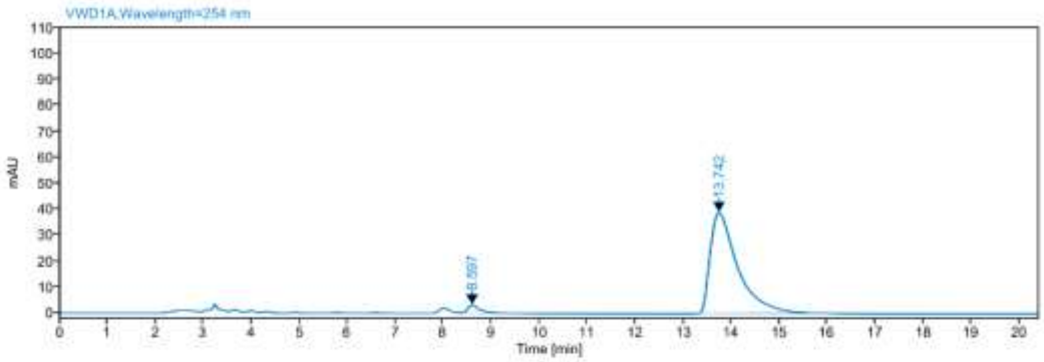

Signal: VWD1A,Wavelength=254 nm

RT [min]	Type	Width [min]	Area	Height	Area%	Name
8.597	MM m	0.97	49.48	3.03	3.10	
13.742	MM m	3.41	1548.09	38.99	96.90	
		Sum	1597.58			

(S)-methyl-3-(((methyldiphenylphosphane)boryl) (dimethoxyphosphoryl)methyl)benzoate (3pa)

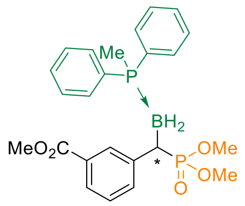

3pa

Following the general procedure D, methyl 3-(diazo(dimethoxyphosphoryl)methyl)benzoate (0.20 mmol, 1.0 equiv) and methyldiphenylphosphane borane (0.40 mmol, 2.0 equiv) were employed and stirred at 20 °C for 12 h to afford **3pa** as a colorless oil in 85% yield (79.9 mg) with 94% ee.

R_f = 0.33 (silica gel, EtOAc:PE = 3:1)

^{1}H NMR (400 MHz, Chloroform-*d*) δ 7.70 – 7.62 (m, 2H), 7.50 – 7.36 (m, 9H), 7.35 – 7.26 (m, 2H), 7.16 (t, *J* = 7.7 Hz, 1H), 3.84 (s, 3H), 3.60 (dd, *J* = 10.5, 5.0 Hz, 6H), 2.59 – 2.44 (m, 1H), 1.42 (d, *J* = 10.1 Hz, 3H).

^{13}C NMR (101 MHz, Chloroform-*d*) δ 167.2, 140.5 (dd, *J* = 8.8, 5.6 Hz), 134.1 (d, *J* = 6.8 Hz), 131.7 (dd, *J* = 15.3, 8.9 Hz), 131.1 (dd, *J* = 18.7, 2.5 Hz), 130.4 (d, *J* = 8.5 Hz), 129.6 (d, *J* = 2.5 Hz), 129.1 (d, *J* = 57.9 Hz), 128.8 (dd, *J* = 19.4, 10.1 Hz), 128.0 (d, *J* = 2.8 Hz), 127.9 (d, *J* = 56.2 Hz), 126.3 (d, *J* = 3.3 Hz), 52.7 (dd, *J* = 46.4, 7.0 Hz), 51.8, 9.5 (d, *J* = 37.4 Hz).

^{11}B NMR (128 MHz, Chloroform-*d*) δ -25.93.

^{31}P NMR (162 MHz, Chloroform-*d*) δ 37.85 (d, *J* = 79.1 Hz), 5.34.

HRMS (ESI): calc’d for $(M+H)^+$ $C_{24}H_{30}BO_5P_2^+$ 471.1656, found 471.1655.

HPLC analysis: DAICEL CHIRALCEL IA-3, hexane/isopropanol = 80/20, 1 mL/min, λ = 254 nm, t_R (major) = 21.276min, t_R (minor) = 12.364 min, 94% ee.

[α]^{25}$_D$: +30.6 (*c* 0.5, $CHCl_3$).

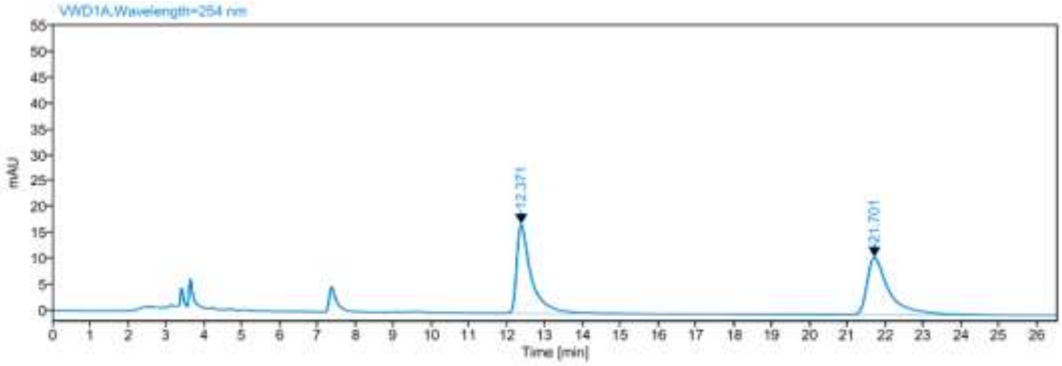

Signal: VWD1A,Wavelength=254 nm

RT [min]	Type	Width [min]	Area	Height	Area%	Name
12.371	BB	3.85	460.69	17.15	50.43	
21.701	BB	3.99	452.82	11.04	49.57	
		Sum	913.50			

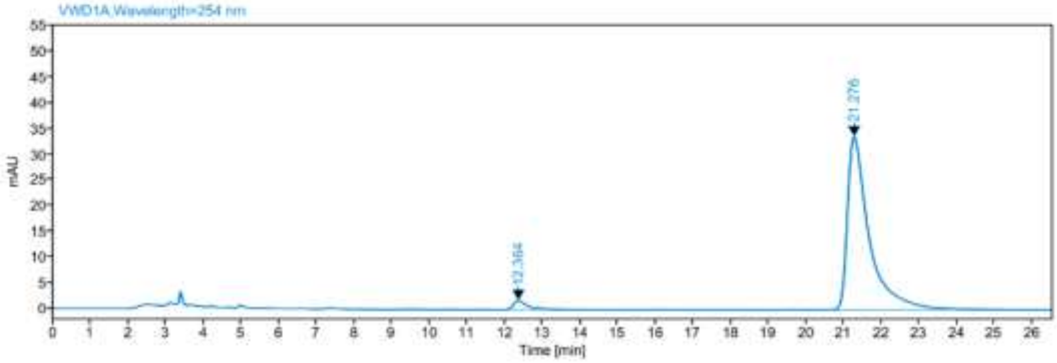

Signal: VWD1A,Wavelength=254 nm

RT [min]	Type	Width [min]	Area	Height	Area%	Name
12.364	BB	1.83	45.19	1.75	3.24	
21.276	BB	4.75	1347.88	33.63	96.76	
		Sum	1393.07			

(S)-dimethyl(((methyldiphenylphosphane)boryl)(3-iodophenyl)methyl)phosphonate(3qa)

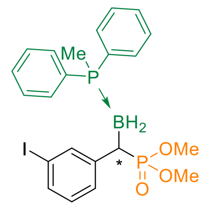

3qa

Following the general procedure D, dimethyl (diazo(3-iodophenyl)methyl)phosphonate (0.20 mmol, 1.0 equiv) and methyldiphenylphosphane borane (0.40 mmol, 2.0 equiv) were employed and stirred

at 20 °C for 12 h to afford **3qa** as a colorless oil in 79% yield (84.7 mg) with 95% ee.

R*f* = 0.47 (silica gel, EtOAc:PE = 3:1)

^{1}H NMR (400 MHz, Chloroform-*d*) δ 7.53 – 7.39 (m, 8H), 7.38 – 7.30 (m, 3H), 7.27 (d, *J* = 2.1 Hz, 1H), 7.18 (d, *J* = 7.8 Hz, 1H), 6.82 (t, *J* = 7.8 Hz, 1H), 3.61 (dd, *J* = 10.6, 4.2 Hz, 6H), 2.44 – 2.28 (m, 1H), 1.41 (d, *J* = 10.2 Hz, 3H).

^{13}C NMR (101 MHz, Chloroform-*d*) δ 144.1 (dd, *J* = 8.4, 5.5 Hz), 138.0 (d, *J* = 8.7 Hz), 134.0 (d, *J* = 3.5 Hz), 131.7 (dd, *J* = 15.7, 8.9 Hz), 131.3 (dd, *J* = 15.7, 2.5 Hz), 129.7 (d, *J* = 2.9 Hz), 129.0 (d, *J* = 58.2 Hz), 128.8 (dd, *J* = 23.2, 10.0 Hz), 128.6 (d, *J* = 7.1 Hz), 127.6 (d, *J* = 56.0 Hz), 94.0 (d, *J* = 3.0 Hz), 52.8 (dd, *J* = 45.3, 7.1 Hz), 9.5 (d, *J* = 37.7 Hz).

^{11}B NMR (128 MHz, Chloroform-*d*) δ -25.96.

^{31}P NMR (162 MHz, Chloroform-*d*) δ 37.71 (d, *J* = 81.2 Hz), 5.34.

HRMS (ESI): calc'd for $(M+H)^+$ $C_{22}H_{27}BIO_3P_2^+$ 539.0568, found 539.0586.

HPLC analysis: DAICEL CHIRALCEL IA-3, hexane/isopropanol = 85/15, 1 mL/min, λ = 254 nm, t_R (major) = 15.157 min, t_R (minor) = 12.817 min, 95% ee.

$[\alpha]^{25}_D$: +37.6 (*c* 0.5, $CHCl_3$).

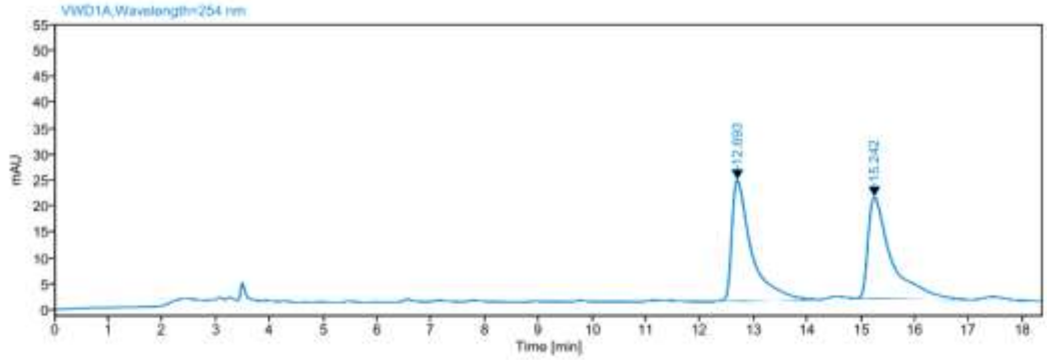

Signal: VWD1A,Wavelength=254 nm

RT [min]	Type	Width [min]	Area	Height	Area%	Name
12.693	BB	2.04	591.94	23.32	50.24	
15.242	BB	2.13	586.17	19.54	49.76	
		Sum	1178.10			

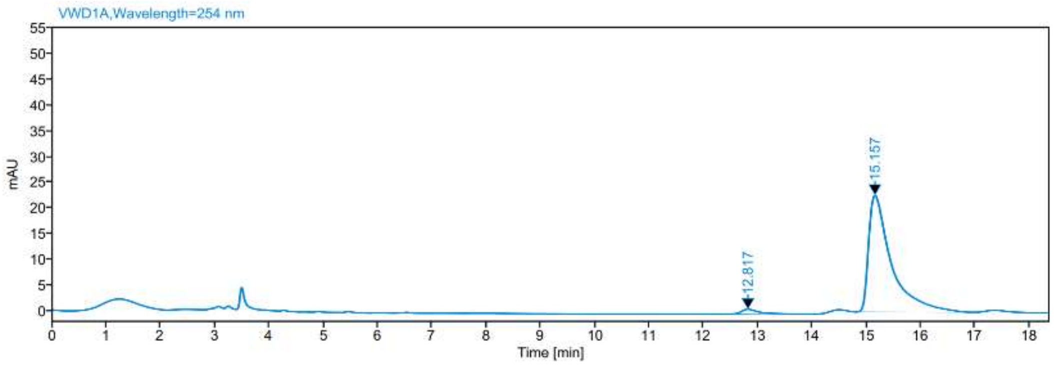

Signal: VWD1A,Wavelength=254 nm

RT [min]	Type	Width [min]	Area	Height	Area%	Name
12.817	MM m	0.88	17.51	0.89	2.57	
15.157	BB	2.16	662.94	22.70	97.43	
		Sum	680.44			

(S)-dimethyl(((methyldiphenylphosphane)boryl)(2-fluorophenyl)methyl)phosphonate(3ra)

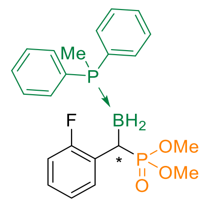

3ra

Following the general procedure D, dimethyl (diazo(2-fluorophenyl)methyl)phosphonate (0.20 mmol, 1.0 equiv) and methyldiphenylphosphane borane (0.40 mmol, 2.0 equiv) were employed and stirred at 20 °C for 12 h to afford **3ra** as a white solid in 90% yield (77.1 mg) with 95% ee.

mp: 119.3 –120.6 °C

$\mathbf{R}_f$ = 0.53 (silica gel, EtOAc:PE = 3:1)

^{1}H NMR (500 MHz, Chloroform-*d*) δ 7.65 – 7.60 (m, 1H), 7.50 – 7.34 (m, 8H), 7.31 – 7.26 (m, 2H), 6.94 – 6.87 (m, 2H), 6.75 – 6.64 (m, 1H), 3.59 (dd, *J* = 10.5, 6.5 Hz, 6H), 3.03 – 2.89 (m, 1H), 1.58 (d, *J* = 10.2 Hz, 3H).

^{13}C NMR (101 MHz, Chloroform-*d*) δ 160.7 (d, *J* = 9.7 Hz), 158.3 (d, *J* = 9.9 Hz), 131.7 (dd, *J* = 11.9, 9.0 Hz), 131.0 (dd, *J* = 18.7, 2.5 Hz), 128.9, 128.7 (dd, *J* = 18.0, 10.0 Hz), 128.2 (d, *J* = 14.4 Hz), 126.1 (dd, *J* = 8.2, 3.5 Hz), 123.7 (t, *J* = 3.5 Hz), 114.2 (dd, *J* = 23.6, 2.6 Hz), 52.6 (dd, *J* = 44.9, 6.9 Hz), 9.0 (d, *J* = 37.5 Hz).

^{11}B NMR (160 MHz, Chloroform-*d*) δ -26.96.

^{31}P NMR (202 MHz, Chloroform-*d*) δ 38.75 (d, J = 80.8 Hz), 5.78.

^{19}F NMR (471 MHz, Chloroform-*d*) δ -119.96.

HRMS (ESI): calc'd for $(M+H)^+$ $C_{22}H_{27}BFO_3P_2^+$ 431.1507, found 431.1519.

HPLC analysis: DAICEL CHIRALCEL AS-H, hexane/isopropanol = 80/20, 1 mL/min, λ = 254 nm, t_R (major) = 26.975 min, t_R (minor) = 10.328 min, 95% ee.

$[\alpha]^{25}_D$: +40.2 (*c* 0.5, $CHCl_3$).

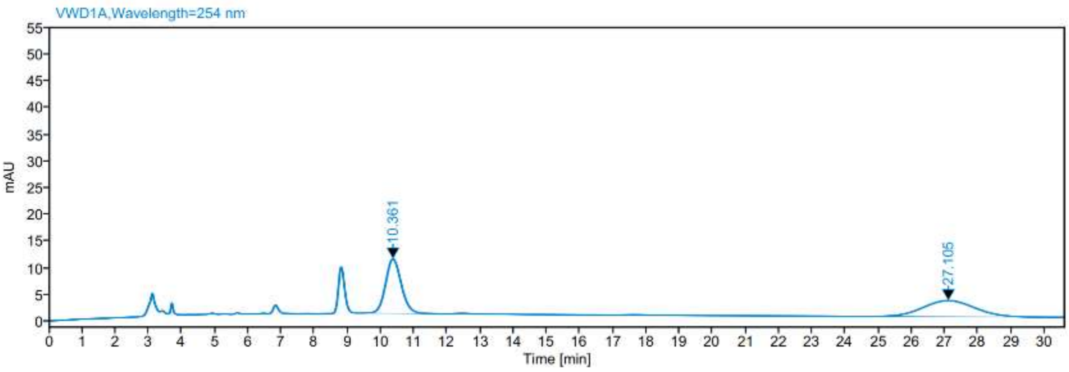

Signal: VWD1A,Wavelength=254 nm

RT [min]	Type	Width [min]	Area	Height	Area%	Name
10.361	BB	2.47	336.94	10.21	50.47	
27.105	BB	5.14	330.63	3.03	49.53	
		**Sum**	**667.57**			

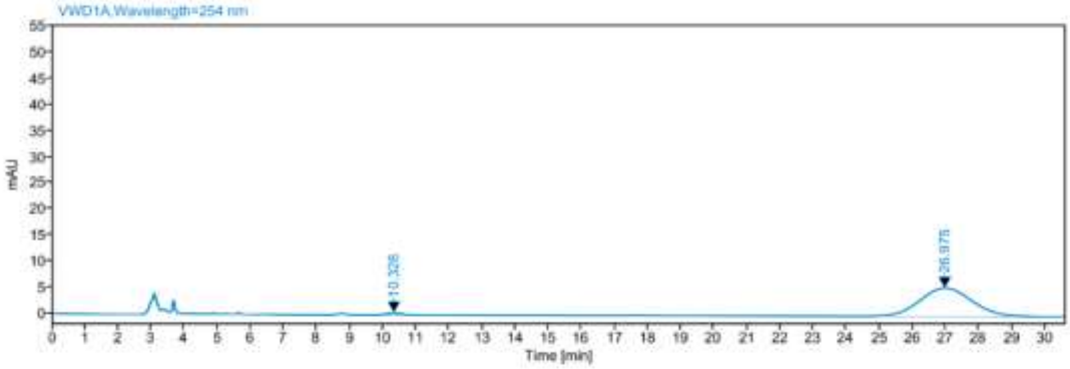

Signal: VWD1A,Wavelength=254 nm

RT [min]	Type	Width [min]	Area	Height	Area%	Name
10.328	MM m	2.04	14.29	0.45	2.36	
26.975	BB	5.40	591.35	5.44	97.64	
		**Sum**	**605.64**			

(S)-dimethyl(((methyldiphenylphosphane)boryl)(3-chloro-4-fluorophenyl)methyl) phosphonate(3sa)

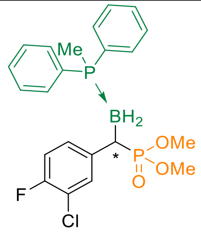

3sa

Following the general procedure D, dimethyl ((3-chloro-4-fluorophenyl)(diazo)methyl)phosphonate (0.20 mmol, 1.0 equiv) and methyldiphenylphosphane borane (0.40 mmol, 2.0 equiv) were employed and stirred at 20 °C for 12 h to afford **3sa** as a white solid in 88% yield (82.1 mg) with 94% ee.

mp: 132.0 –132.9 °C

R_f = 0.28 (silica gel, EtOAc:PE = 3:1)

^{1}H NMR (400 MHz, Chloroform-*d*) δ 7.51 – 7.38 (m, 8H), 7.37 – 7.30 (m, 2H), 7.06 – 6.96 (m, 2H), 6.80 (t, *J* = 8.8 Hz, 1H), 3.62 (dd, *J* = 10.5, 5.5 Hz, 6H), 2.51 – 2.31 (m, 1H), 1.53 (d, *J* = 10.2 Hz, 3H).

^{13}C NMR (101 MHz, Chloroform-*d*) δ 157.0 (d, *J* = 3.9 Hz), 154.5 (d, *J* = 3.9 Hz), 138.6, 131.7 (dd, *J* = 14.3, 9.0 Hz), 131.3 (dd, *J* = 16.2, 2.5 Hz), 131.0 (d, *J* = 8.1 Hz), 128.8 (dd, *J* = 17.7, 10.0 Hz), 128.5 (d, *J* = 34.3 Hz), 127.8 (d, *J* = 56.6 Hz), 119.8 (dd, *J* = 17.4, 3.1 Hz), 115.7 (dd, *J* = 20.6, 2.9 Hz), 52.8 (dd, *J* = 43.4, 7.0 Hz), 9.6 (d, *J* = 37.8 Hz).

^{11}B NMR (128 MHz, Chloroform-*d*) δ -26.10.

^{31}P NMR (162 MHz, Chloroform-*d*) δ 37.49 (dd, *J* = 79.2, 5.8 Hz), 5.16.

^{19}F NMR (376 MHz, Chloroform-*d*) δ -121.19 (d, *J* = 5.6 Hz).

HRMS (ESI): calc'd for $(M+H)^+$ $C_{22}H_{26}BClFO_3P_2^+$ 465.1117, found 465.1110.

HPLC analysis: DAICEL CHIRALCEL ID-3, hexane/isopropanol = 80/20, 1 mL/min, λ = 254 nm, t_R (major) = 27.683 min, t_R (minor) = 23.646 min, 94% ee.

$[\alpha]^{25}_D$: +54.4 (*c* 0.5, $CHCl_3$).

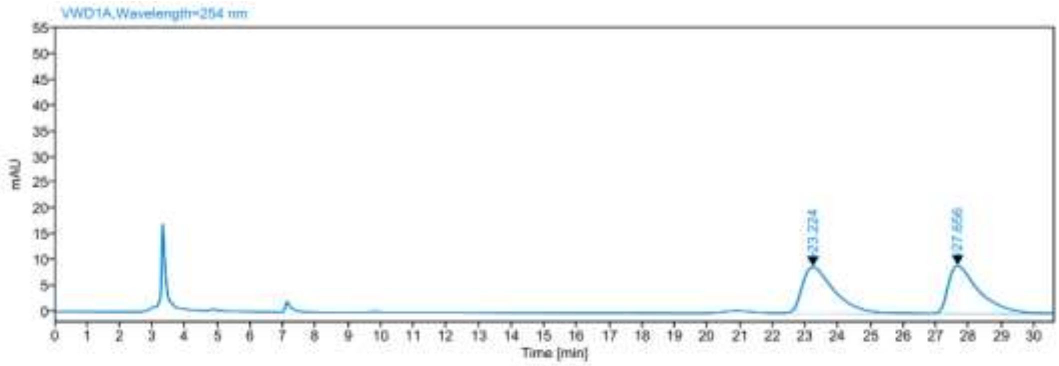

Signal: VWD1A,Wavelength=254 nm

RT [min]	Type	Width [min]	Area	Height	Area%	Name
23.224	BB	4.47	607.47	8.99	50.14	
27.656	BB	4.14	604.12	9.25	49.86	
		Sum	1211.58			

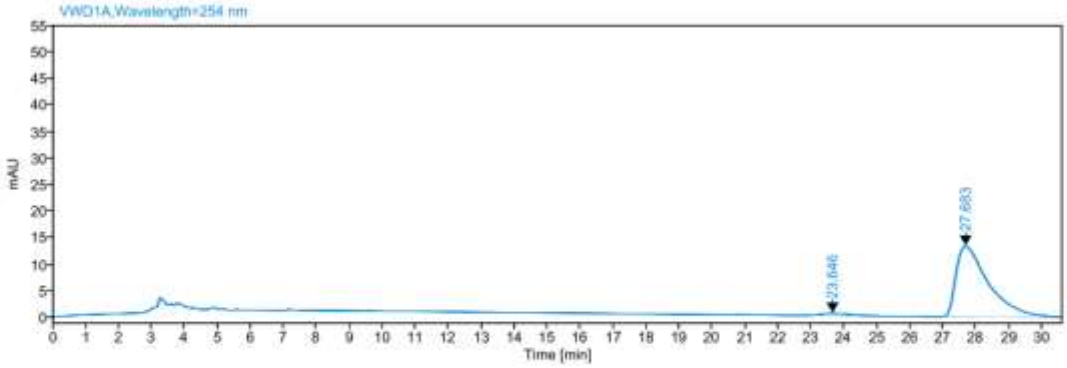

Signal: VWD1A,Wavelength=254 nm

RT [min]	Type	Width [min]	Area	Height	Area%	Name
23.646	MM m	2.74	26.76	0.43	2.85	
27.683	BB	4.98	912.75	13.31	97.15	
		Sum	939.51			

(S)-dimethyl(((methyldiphenylphosphane)boryl)(4-methyl-3-nitrophenyl)methyl) phosphonate(3ta)

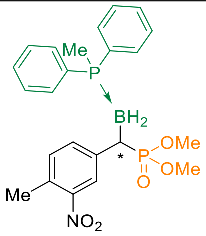

3ta

Following the general procedure D, dimethyl (diazo(4-methyl-3-nitrophenyl)methyl)phosphonate (0.20 mmol, 1.0 equiv) and methyldiphenylphosphane borane (0.40 mmol, 2.0 equiv) were employed and stirred at 20 °C for 12 h to afford **3ta** as a colorless oil in 89% yield (84.3 mg) with 94% ee.

R$_f$ = 0.22 (silica gel, EtOAc:PE = 3:1)

^{1}H NMR (400 MHz, Chloroform-*d*) δ 7.52 (t, *J* = 2.4 Hz, 1H), 7.48 – 7.34 (m, 9H), 7.33 – 7.27 (m, 2H), 6.98 (d, *J* = 7.9 Hz, 1H), 3.62 (dd, *J* = 10.6, 2.2 Hz, 6H), 2.59 – 2.37 (m, 4H), 1.58 (d, *J* = 10.0 Hz, 3H).

^{13}C NMR (101 MHz, Chloroform-*d*) δ 148.4 (d, *J* = 2.6 Hz), 141.3 (dd, *J* = 8.2, 5.7 Hz), 134.1 (d, *J* = 6.6 Hz), 132.1 (d, *J* = 2.9 Hz), 131.7 (t, *J* = 9.5 Hz), 131.2 (dd, *J* = 23.4, 2.6 Hz), 129.7 (d, *J* = 2.7 Hz), 128.8 (dd, *J* = 15.6, 10.0 Hz), 128.4 (d, *J* = 45.5 Hz), 127.8 (d, *J* = 45.1 Hz), 125.0 (d, *J* = 8.8 Hz), 52.8 (dd, *J* = 39.4, 7.0 Hz), 19.9, 9.6 (d, *J* = 38.0 Hz).

^{11}B NMR (128 MHz, Chloroform-*d*) δ -26.09.

^{31}P NMR (162 MHz, Chloroform-*d*) δ 37.05 (d, *J* = 76.4 Hz), 5.07.

HRMS (ESI): calc'd for $(M+H)^+$ $C_{23}H_{29}BNO_5P_2^+$ 472.1609, found 472.1628.

HPLC analysis: DAICEL CHIRALCEL IA-3, hexane/isopropanol = 80/20, 1 mL/min, λ = 254 nm, t_R (major) = 15.027 min, t_R (minor) = 12.719 min, 94% ee.

[α]$^{25}_{D}$: +35.0 (*c* 0.5, $CHCl_3$).

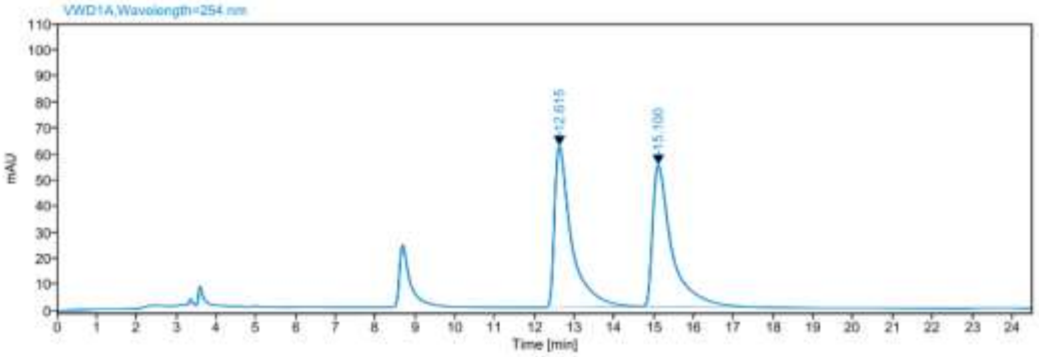

Signal: VWD1A,Wavelength=254 nm

RT [min]	Type	Width [min]	Area	Height	Area%	Name
12.615	BB	2.45	1822.42	61.81	49.94	
15.100	BB	4.27	1826.88	54.30	50.06	
		Sum	3649.29			

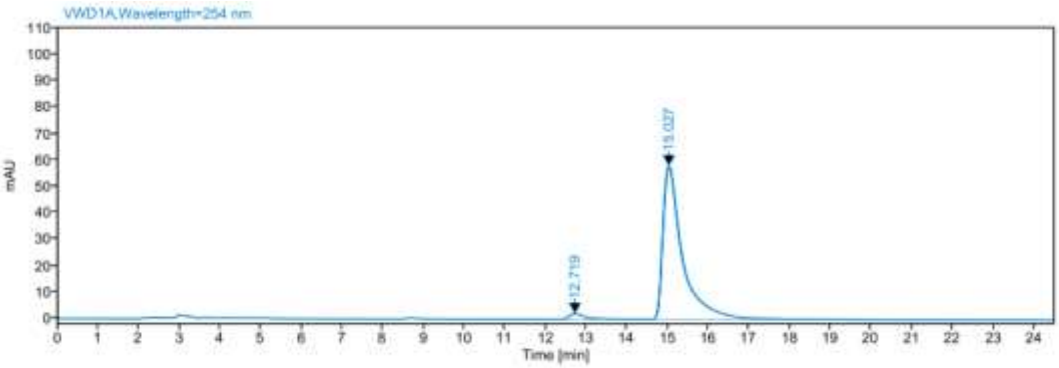

Signal: VWD1A,Wavelength=254 nm

RT [min]	Type	Width [min]	Area	Height	Area%	Name
12.719	BB	1.88	62.13	2.17	3.09	
15.027	BB	5.68	1951.42	58.15	96.91	
		Sum	2013.54			

(S)-dimethyl(((methyldiphenylphosphane)boryl)(3,5-dimethoxyphenyl)methyl) phosphonate(3ua)

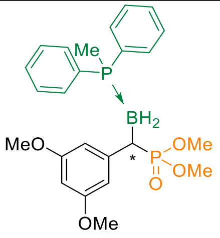

3ua

Following the general procedure D, dimethyl (diazo(3,5-dimethoxyphenyl)methyl)phosphonate (0.20 mmol, 1.0 equiv) and methyldiphenylphosphane borane (0.40 mmol, 2.0 equiv) were employed and stirred at 20 °C for 12 h to afford **3ua** as a colorless oil in 81% yield (76.2 mg) with 93% ee.

R*f* = 0.19 (silica gel, EtOAc:PE = 3:1)

^{1}H NMR (400 MHz, Chloroform-*d*) δ 7.52 – 7.36 (m, 8H), 7.35 – 7.27 (m, 2H), 6.29 (t, *J* = 2.4 Hz, 2H), 6.13 (q, *J* = 2.2 Hz, 1H), 3.67 – 3.56 (m, 12H), 2.48 – 2.32 (m, 1H), 1.40 (d, *J* = 10.2 Hz, 3H).

^{13}C NMR (101 MHz, Chloroform-*d*) δ 160.2 (d, *J* = 2.6 Hz), 143.5 (dd, *J* = 7.8, 5.4 Hz), 131.7 (dd, *J* = 26.6, 8.9 Hz), 131.1 (dd, *J* = 26.0, 2.6 Hz), 129.6 (d, *J* = 58.1 Hz), 128.7 (dd, *J* = 26.5, 9.9 Hz), 127.8 (d, *J* = 55.7 Hz), 107.3 (d, *J* = 8.1 Hz), 97.7 (d, *J* = 3.2 Hz), 55.0, 52.8 (dd, *J* = 42.3, 7.0 Hz), 9.2 (d, *J* = 37.1 Hz).

^{11}B NMR (128 MHz, Chloroform-*d*) δ -26.05.

^{31}P NMR (162 MHz, Chloroform-*d*) δ 38.51 (d, *J* = 84.5 Hz), 5.41.

HRMS (ESI): calc'd for $(M+H)^+$ $C_{24}H_{32}BO_5P_2^+$ 473.1813, found 473.1825.

HPLC analysis: DAICEL CHIRALCEL AD-H, hexane/isopropanol = 70/30, 1 mL/min, λ = 254 nm, t_R (major) = 23.206 min, t_R (minor) = 12.841 min, 93% ee.

[α]^{25}$_D$: +41.6 (*c* 0.5, $CHCl_3$).

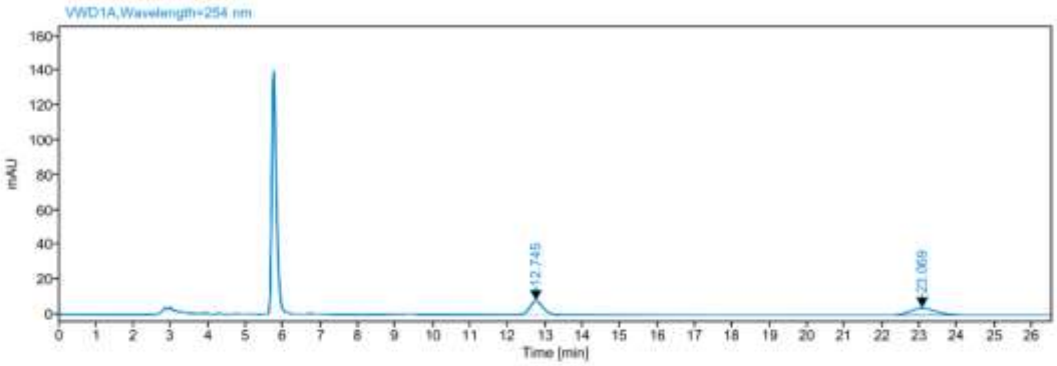

Signal: VWD1A,Wavelength=254 nm

RT [min]	Type	Width [min]	Area	Height	Area%	Name
12.745	BB	1.67	194.00	7.94	50.09	
23.069	BB	2.62	193.28	3.96	49.91	
		Sum	387.27			

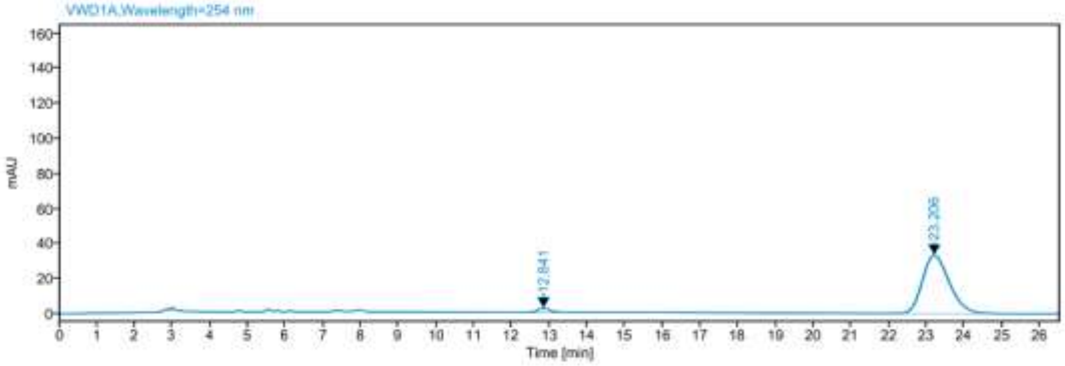

Signal: VWD1A,Wavelength=254 nm

RT [min]	Type	Width [min]	Area	Height	Area%	Name
12.841	BB	1.56	58.48	2.39	3.43	
23.206	BB	3.31	1648.91	33.22	96.57	
		Sum	1707.40			

(S)-dimethyl(((methyldiphenylphosphane)boryl)(9H-fluoren-2-yl)methyl)phosphonate(3va)

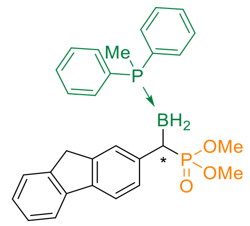

3va

Following the general procedure D, dimethyl (diazo(9H-fluoren-2-yl)methyl)phosphonate (0.20 mmol, 1.0 equiv) and methyldiphenylphosphane borane (0.40 mmol, 2.0 equiv) were employed and stirred

at 20 °C for 12 h to afford **3va** as a yellow oil in 95% yield (95.2 mg) with 94% ee.

R_f = 0.27 (silica gel, EtOAc:PE = 3:1)

^{1}H NMR (400 MHz, Chloroform-*d*) δ 7.69 (d, *J* = 7.5 Hz, 1H), 7.58 – 7.38 (m, 9H), 7.37 – 7.21 (m, 6H), 7.10 (d, *J* = 7.9 Hz, 1H), 3.75 – 3.56 (m, 8H), 2.66 – 2.48 (m, 1H), 1.32 (d, *J* = 10.2 Hz, 3H).

^{13}C NMR (101 MHz, Chloroform-*d*) δ 143.1, 143.0, 141.8, 139.9 (dd, *J* = 8.5, 5.7 Hz), 138.8 (d, *J* = 3.6 Hz), 131.7 (dd, *J* = 27.7, 8.9 Hz), 131.0 (dd, *J* = 32.3, 2.5 Hz), 129.5 (d, *J* = 57.9 Hz), 128.7 (dd, *J* = 27.0, 10.0 Hz), 127.9 (d, *J* = 8.3 Hz), 127.8 (d, *J* = 55.6 Hz), 126.5, 126.1, 126.0, 124.8, 119.3, 119.3, 52.8 (dd, *J* = 47.9, 7.1 Hz), 36.7, 9.4 (d, *J* = 37.0 Hz).

^{11}B NMR (128 MHz, Chloroform-*d*) δ -25.80.

^{31}P NMR (162 MHz, Chloroform-*d*) δ 38.94 (d, *J* = 85.4 Hz), 5.53.

HRMS (ESI): calc'd for $(M+H)^+$ $C_{29}H_{32}BO_3P_2^+$ 501.1914, found 501.1924.

HPLC analysis: DAICEL CHIRALCEL AD-H, hexane/isopropanol = 80/20, 1 mL/min, λ = 254 nm, t_R (major) = 20.721 min, t_R (minor) = 15.399 min, 94% ee.

$[\alpha]^{25}_D$: +107.4 (*c* 0.5, $CHCl_3$).

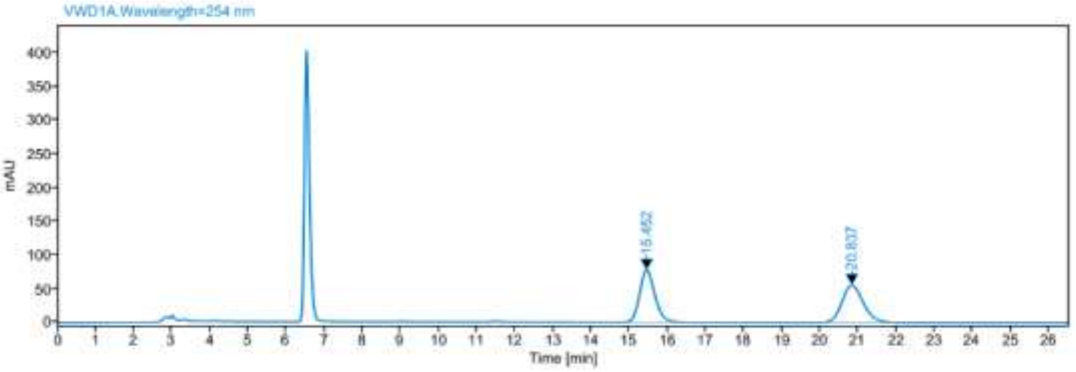

Signal: VWD1A,Wavelength=254 nm

RT [min]	Type	Width [min]	Area	Height	Area%	Name
15.452	BB	2.65	2118.42	77.10	49.95	
20.837	BB	3.01	2122.57	55.28	50.05	
		Sum	4240.98			

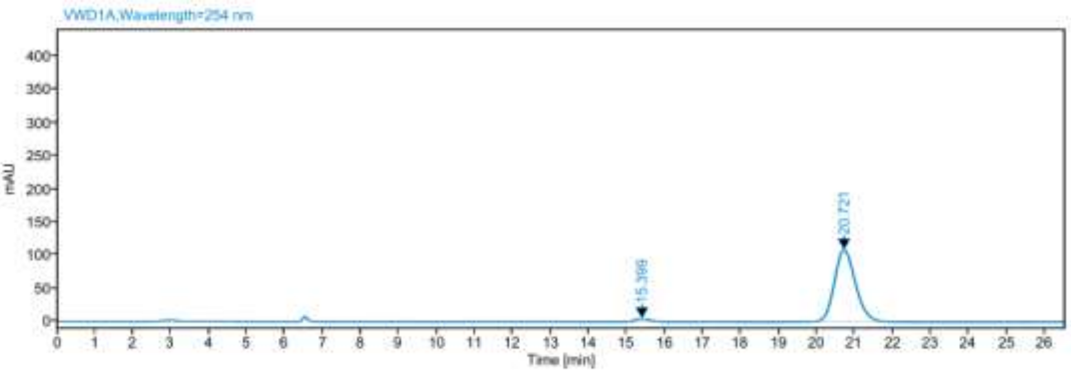

Signal: VWD1A,Wavelength=254 nm

RT [min]	Type	Width [min]	Area	Height	Area%	Name
15.399	BB	1.46	136.83	5.01	3.14	
20.721	BB	4.41	4224.39	108.81	96.86	
		Sum	4361.22			

(S)-dimethyl(((methyldiphenylphosphane)boryl)(1H-indol-5-yl)methyl)phosphonate(3wa)

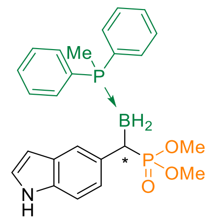

3wa

Following the general procedure D, dimethyl (diazo(1H-indol-5-yl)methyl)phosphonate (0.20 mmol, 1.0 equiv) and methyldiphenylphosphane borane (0.40 mmol, 2.0 equiv) were employed and stirred at 20 °C for 12 h to afford **3wa** as a colorless oil in 85% yield (76.4 mg) with 89% ee.

R_f = 0.13 (silica gel, EtOAc:PE = 3:1)

^{1}H NMR (400 MHz, Chloroform-*d*) δ 8.6 (s, 1H), 7.5 – 7.3 (m, 8H), 7.3 – 7.2 (m, 3H), 7.1 (d, J = 8.4 Hz, 1H), 7.1 (t, J = 2.8 Hz, 1H), 7.1 (d, J = 8.3 Hz, 1H), 6.3 (s, 1H), 3.6 (dd, J = 28.8, 10.4 Hz, 6H), 2.6 – 2.5 (m, 1H), 1.2 (dd, J = 10.3, 2.1 Hz, 3H).

^{13}C NMR (101 MHz, Chloroform-*d*) δ 134.4, 131.8 (dd, J = 24.3, 8.8 Hz), 131.0 (dd, J = 26.4, 2.5 Hz), 130.1 (d, J = 57.8 Hz), 128.7 (dd, J = 28.2, 9.9 Hz), 128.1 (d, J = 54.8 Hz), 127.9 (d, J = 2.3 Hz), 124.1, 124.0 (d, J = 7.1 Hz), 120.9 (d, J = 9.1 Hz), 110.8, 101.9, 52.7 (dd, J = 47.3, 7.0 Hz), 9.3 (d, J = 36.5 Hz).

^{11}B NMR (128 MHz, Chloroform-*d*) δ -25.15.

^{31}P NMR (162 MHz, Chloroform-*d*) δ 39.97 (d, J = 88.0 Hz), 5.92.

HRMS (ESI): calc'd for $(M+H)^+$ $C_{24}H_{29}BNO_3P_2^+$ 452.1710, found 452.1709.

HPLC analysis: DAICEL CHIRALCEL AD-H, hexane/isopropanol = 60/40, 1 mL/min, λ = 254 nm, t_R (major) = 11.858 min, t_R (minor) = 10.448 min, 89% ee.

[α]$^{25}_{D}$: +81.2 (*c* 0.25, $CHCl_3$).

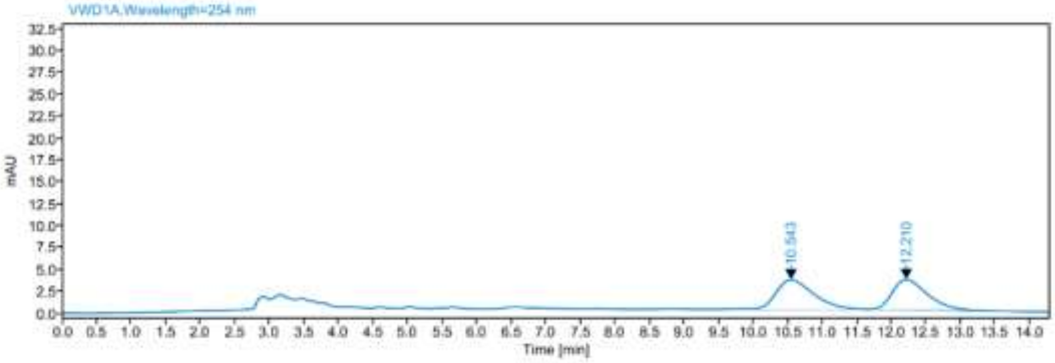

Signal: VWD1A,Wavelength=254 nm

RT [min]	Type	Width [min]	Area	Height	Area%	Name
10.543	BB	2.32	127.49	3.36	50.75	
12.210	BB	2.05	123.74	3.45	49.25	
		Sum	251.24			

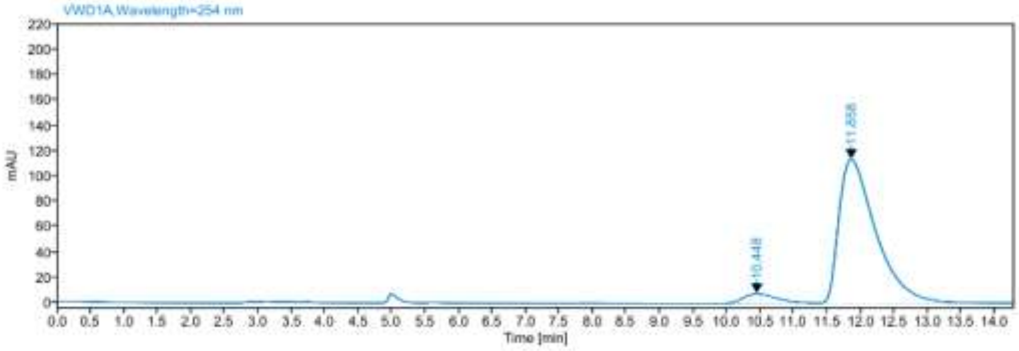

Signal: VWD1A,Wavelength=254 nm

RT [min]	Type	Width [min]	Area	Height	Area%	Name
10.448	MM m	1.30	255.85	7.52	5.53	
11.858	MM m	4.28	4371.36	113.62	94.47	
		Sum	4627.21			

(S)-dimethyl(((methyldiphenylphosphane)boryl)(benzo[d][1,3]dioxol-5-yl)methyl) phosphonate(3xa)

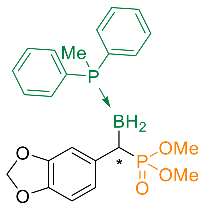

3xa

Following the general procedure D, dimethyl (benzo[d][1,3]dioxol-5-yl(diazo)methyl)phosphonate

(0.20 mmol, 1.0 equiv) and methyldiphenylphosphane borane (0.40 mmol, 2.0 equiv) were employed and stirred at 20 °C for 12 h to afford **3xa** as a colorless oil in 77% yield (70.5 mg) with 91% ee.

R_f = 0.23 (silica gel, EtOAc:PE = 3:1)

^{1}H NMR (500 MHz, Chloroform-*d*) δ 7.51 – 7.38 (m, 8H), 7.36 – 7.30 (m, 2H), 6.75 (t, J = 2.0 Hz, 1H), 6.51 (d, J = 7.9 Hz, 1H), 6.48 – 6.42 (m, 1H), 5.85 – 5.78 (m, 2H), 3.61 (dd, J = 10.4, 7.4 Hz, 6H), 2.46 – 2.33 (m, 1H), 1.46 (d, J = 10.2 Hz, 3H).

^{13}C NMR (126 MHz, Chloroform-*d*) δ 147.1 (d, J = 3.0 Hz), 145.0 (d, J = 3.7 Hz), 135.0 (dd, J = 9.1, 5.7 Hz), 131.7 (dd, J = 24.8, 8.9 Hz), 131.1 (dd, J = 31.9, 2.4 Hz), 129.4 (d, J = 58.3 Hz), 128.7 (dd, J = 30.0, 9.9 Hz), 128.0 (d, J = 55.8 Hz), 121.9 (d, J = 9.1 Hz), 110.0 (d, J = 6.6 Hz), 107.6 (d, J = 2.9 Hz), 100.5, 52.7 (dd, J = 54.9, 7.1 Hz), 9.3 (d, J = 37.0 Hz).

^{31}P NMR (202 MHz, Chloroform-*d*) δ 39.25 (d, J = 84.1 Hz), 6.19.

^{11}B NMR (160 MHz, Chloroform-*d*) δ -26.79.

HRMS (ESI): calc'd for $(M+H)^+$ $C_{23}H_{28}BO_5P_2^+$ 457.1500, found 457.1500.

HPLC analysis: DAICEL CHIRALCEL IA-3, hexane/isopropanol = 80/20, 1 mL/min, λ = 254 nm, t_R (major) = 30.272 min, t_R (minor) = 19.796 min, 91% ee.

$[\alpha]^{25}_D$: +70.2 (*c* 0.5, $CHCl_3$).

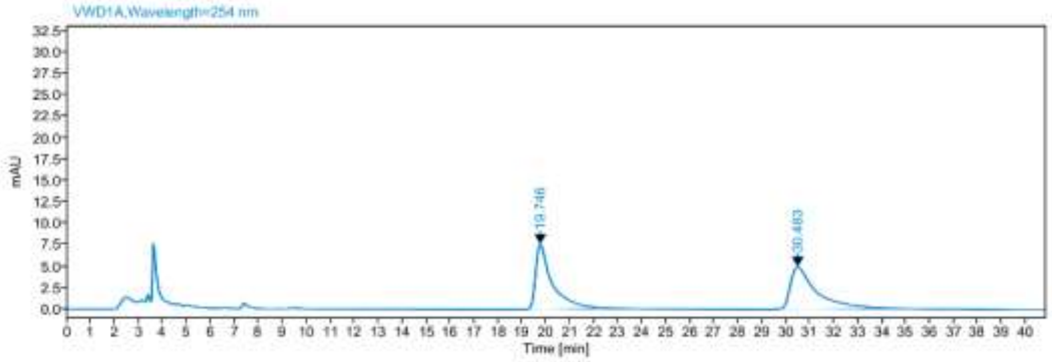

Signal: VWD1A,Wavelength=254 nm

RT [min]	Type	Width [min]	Area	Height	Area%	Name
19.746	BB	3.91	367.69	7.56	49.25	
30.483	BB	5.30	378.84	4.92	50.75	
		Sum	746.53			

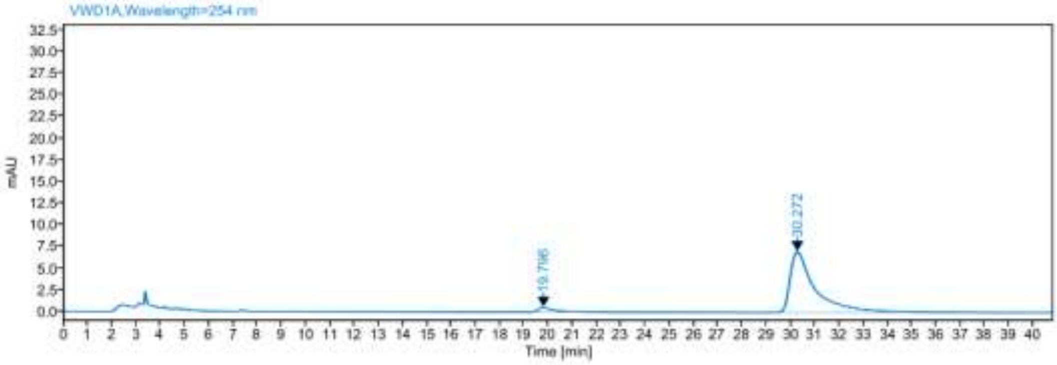

Signal:	VWD1A,Wavelength=254 nm					
RT [min]	Type	Width [min]	Area	Height	Area%	Name
19.796	MM m	3.79	23.48	0.52	4.45	
30.272	BM m	7.82	504.53	7.00	95.55	
		Sum	528.01			

(S)-dimethyl(((methyldiphenylphosphane)boryl)(dibenzo[b,d]furan-1-yl)methyl) phosphonate(3ya)

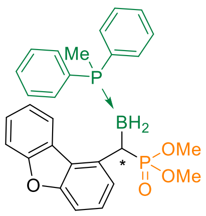

3ya

In air, a 25 mL schlenk tube was charged with $Cu(MeCN)_4PF_6$ (10 mol%), **L1** (12 mol%). The tube was evacuated and filled with argon for three cycles. Then, 2 mL of CPME, dimethyl (diazo(dibenzo[b,d]furan-1-yl)phosphonate (0.20 mmol, 1.0 equiv) and methyldiphenylphosphane borane (0.40 mmol, 2.0 equiv) were employed and stirred at 20 °C for 12 h to afford **3ya** as a colorless oil in 77% yield (77.4 mg) with 85% ee.

R_f = 0.52 (silica gel, EtOAc:PE = 3:1)

^{1}H NMR (400 MHz, Chloroform-*d*) δ 7.67 (dd, *J* = 7.0, 2.7 Hz, 1H), 7.53 (d, *J* = 7.8 Hz, 1H), 7.47 (d, *J* = 8.2 Hz, 1H), 7.43 – 7.21 (m, 11H), 7.21 – 7.14 (m, 2H), 7.01 (t, *J* = 7.6 Hz, 1H), 3.59 (d, *J* = 10.5 Hz, 6H), 3.50 – 3.34 (m, 1H), 1.35 (d, *J* = 10.3 Hz, 3H).

^{13}C NMR (101 MHz, Chloroform-*d*) δ 155.8 (d, *J* = 2.0 Hz), 155.7, 137.9 (dd, *J* = 8.5, 6.1 Hz), 131.7 (dd, *J* = 8.9, 5.0 Hz), 131.0 (dd, *J* = 4.8, 2.5 Hz), 129.2 (d, *J* = 58.5 Hz), 128.7 (dd, *J* = 12.1, 10.0 Hz), 127.9 (d, *J* = 56.2 Hz), 126.8 (d, *J* = 3.8 Hz), 125.9, 124.7 (d, *J* = 5.4 Hz), 124.4, 122.2 (d, *J* = 6.3 Hz), 122.0 (d, *J* = 9.6 Hz), 111.1, 107.9 (d, *J* = 3.7 Hz), 52.8 (dd, *J* = 56.7, 7.0 Hz), 9.6 (d, *J* = 37.0 Hz).

¹¹B NMR (128 MHz, Chloroform-*d*) δ -25.76.

³¹P NMR (162 MHz, Chloroform-*d*) δ 38.08, 5.22.

HRMS (ESI): calc'd for $(M+H)^+$ $C_{28}H_{30}BO_4P_2^+$ 503.1707, found 503.1707.

HPLC analysis: DAICEL CHIRALCEL ID-3, hexane/isopropanol = 80/20, 1 mL/min, λ = 254 nm, t_R (major) = 36.510 min, t_R (minor) = 29.812 min, 85% ee.

$[\alpha]^{25}_D$: -46.8 (*c* 0.5, $CHCl_3$).

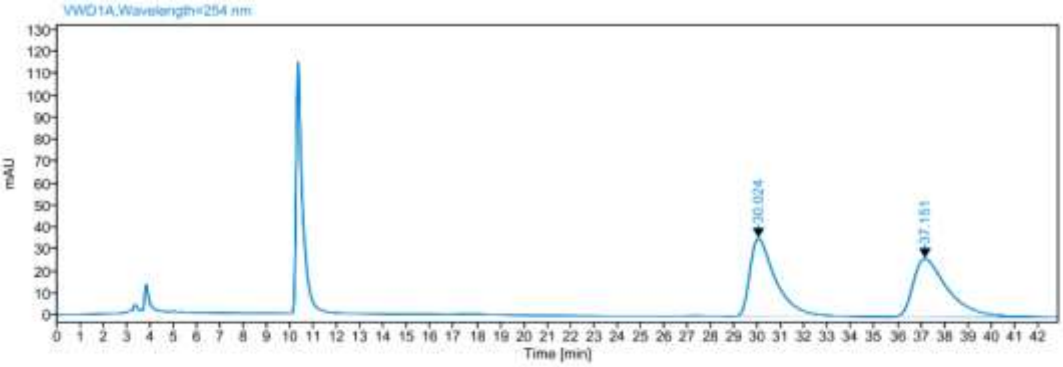

Signal: VWD1A,Wavelength=254 nm

RT [min]	Type	Width [min]	Area	Height	Area%	Name
30.024	BB	6.49	2721.55	35.42	49.96	
37.151	BB	8.31	2726.32	26.37	50.04	
		Sum	5447.87			

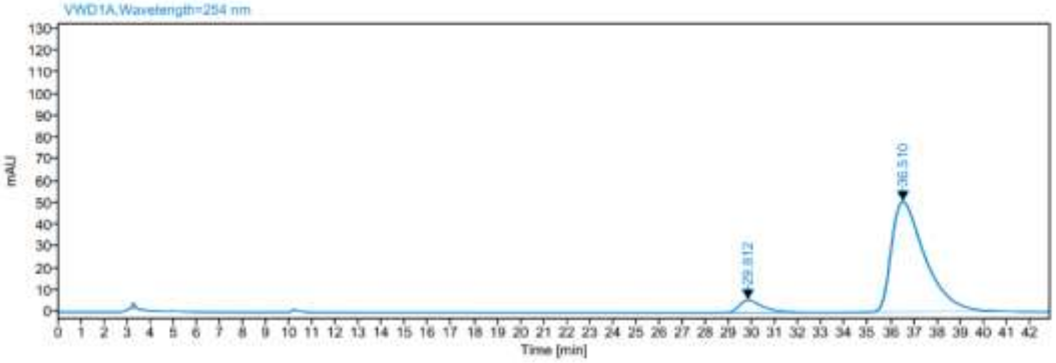

Signal: VWD1A,Wavelength=254 nm

RT [min]	Type	Width [min]	Area	Height	Area%	Name
29.812	BB	4.02	406.63	5.71	7.32	
36.510	BB	7.33	5149.35	50.72	92.68	
		Sum	5555.98			

(S)-dimethyl(((methyldiphenylphosphane)boryl)(dibenzo[b,d]thiophen-4-yl)methyl)phosphonate(3za)

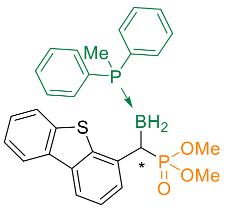

3za

Following the general procedure D, dimethyl (diazo(dibenzo[b,d]thiophen-4-yl)methyl)phosphonate (0.20 mmol, 1.0 equiv) and methyldiphenylphosphane borane (0.40 mmol, 2.0 equiv) were employed and stirred at 20 °C for 36 h to afford **3za** as a colorless oil in 79% yield (82.2 mg) with 98% ee.

R_f = 0.13 (silica gel, EtOAc:PE = 3:1)

^{1}H NMR (500 MHz, Chloroform-*d*) δ 8.06 – 8.02 (m, 1H), 7.79 (dd, *J* = 7.8, 1.2 Hz, 1H), 7.76 – 7.70 (m, 2H), 7.47 – 7.39 (m, 5H), 7.34 – 7.25 (m, 6H), 7.20 – 7.14 (m, 2H), 3.60 (dd, *J* = 28.8, 10.5 Hz, 6H), 2.85 – 2.71 (m, 1H), 1.47 (d, *J* = 10.2 Hz, 3H).

^{13}C NMR (126 MHz, Chloroform-*d*) δ 139.4 (d, *J* = 10.1 Hz), 138.8, 136.6, 136.1 (dd, *J* = 8.9, 5.0 Hz), 134.9, 131.6 (dd, *J* = 57.7, 8.9 Hz), 131.0 (dd, *J* = 45.2, 2.5 Hz), 128.8 (d, *J* = 9.9 Hz), 128.5 (dd, *J* = 64.0, 57.3 Hz), 128.4 (d, *J* = 10.0 Hz), 127.8, 126.2, 124.8, 124.1, 122.5, 121.4, 118.3 (d, *J* = 3.6 Hz), 52.8 (dd, *J* = 51.3, 6.9 Hz), 9.2 (d, *J* = 37.0 Hz).

^{11}B NMR (160 MHz, Chloroform-*d*) δ -26.64.

^{31}P NMR (202 MHz, Chloroform-*d*) δ 38.12 (d, *J* = 80.1 Hz), 5.28.

HRMS (ESI): calc'd for $(M+H)^+$ $C_{28}H_{30}BO_3P_2S+$ 519.1478, found 519.1493.

HPLC analysis: DAICEL CHIRALCEL IA-3, hexane/isopropanol = 80/20, 1 mL/min, λ = 254 nm, t_R (major) = 13.777 min, t_R (minor) = 12.097 min, 98% ee.

$[\alpha]^{25}_D$: -10.0 (*c* 0.5, $CHCl_3$).

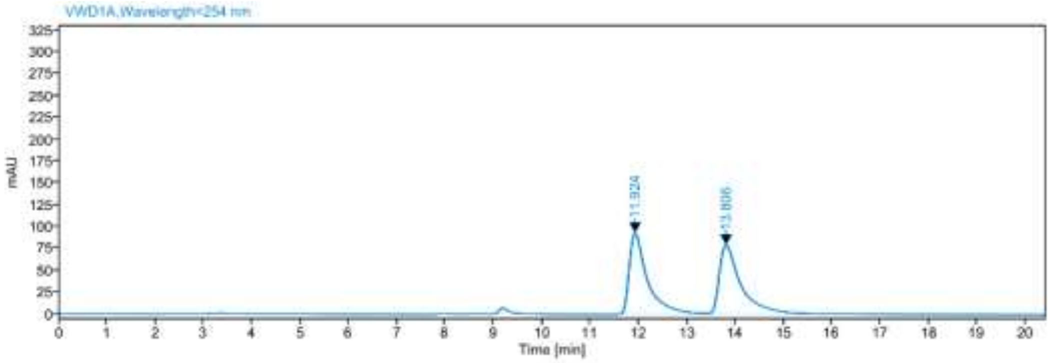

Signal: VWD1A,Wavelength=254 nm

RT [min]	Type	Width [min]	Area	Height	Area%	Name
11.924	BV	1.96	2492.34	93.12	49.01	
13.806	VB	5.76	2592.79	79.44	50.99	
		Sum	5085.13			

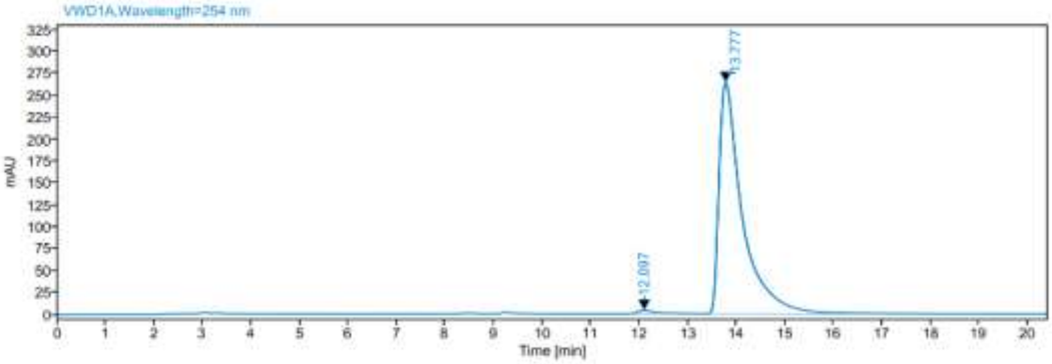

Signal: VWD1A,Wavelength=254 nm

RT [min]	Type	Width [min]	Area	Height	Area%	Name
12.097	BB	1.06	86.76	3.79	0.97	
13.777	BBA	7.83	8864.01	263.58	99.03	
		Sum	8950.77			

(S)-dimethyl(((methyldiphenylphosphane)boryl)(dibenzo[b,d]thiophen-4-yl)methyl) phosphonate(3Aa)

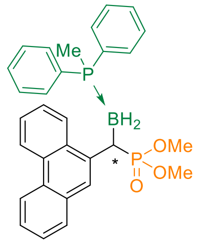

3Aa

Following the general procedure D, dimethyl (diazo(phenanthren-9-yl)methyl)phosphonate (0.20 mmol, 1.0 equiv) and methyldiphenylphosphane borane (0.40 mmol, 2.0 equiv) were employed and stirred at 20 °C for 12 h to afford **3Aa** as a colorless oil in 92% yield (94.2 mg) with 93% ee.

R_f = 0.48 (silica gel, EtOAc:PE = 3:1)

^{1}H NMR (400 MHz, Chloroform-*d*) δ 8.63 (d, *J* = 8.3 Hz, 1H), 8.53 (d, *J* = 7.8 Hz, 1H), 8.11 (d, *J* = 4.4 Hz, 1H), 7.77 (t, *J* = 6.7 Hz, 2H), 7.58 – 7.43 (m, 3H), 7.42 – 7.32 (m, 2H), 7.31 – 7.18 (m, 7H), 7.15 – 7.07 (m, 2H), 3.65 – 3.37 (m, 7H), 1.32 (d, *J* = 10.2 Hz, 3H).

^{13}C NMR (101 MHz, Chloroform-*d*) δ 134.9 (dd, *J* = 8.3, 5.6 Hz), 131.7, 131.5 (dd, *J* = 28.4, 8.9 Hz), 131.2, 131.1, 130.8 (dd, *J* = 27.9, 2.5 Hz), 130.5, 129.3, 128.8, 128.5 (dd, *J* = 29.0, 10.1 Hz), 128.4, 128.3, 128.0 (d, *J* = 52.1 Hz), 126.2, 126.0, 125.5, 123.8, 122.9, 122.0 (d, *J* = 1.6 Hz), 52.9 (dd, *J* = 47.6, 7.1 Hz), 9.6 (d, *J* = 37.0 Hz).

^{11}B NMR (128 MHz, Chloroform-*d*) δ -25.37.

^{31}P NMR (162 MHz, Chloroform-*d*) δ 39.06 (d, *J* = 79.3 Hz), 4.90.

HRMS (ESI): calc'd for $(M+H)^{+}$ $C_{30}H_{32}BO_{3}P_{2}^{+}$ 513.1914, found 513.1914.

HPLC analysis: DAICEL CHIRALCEL IA-3, hexane/isopropanol = 80/20, 1 mL/min, λ = 254 nm, t_R (major) = 26.488 min, t_R (minor) = 17.401 min, 93% ee.

$[\alpha]^{25}_{D}$: +48.2 (*c* 0.5, $CHCl_3$).

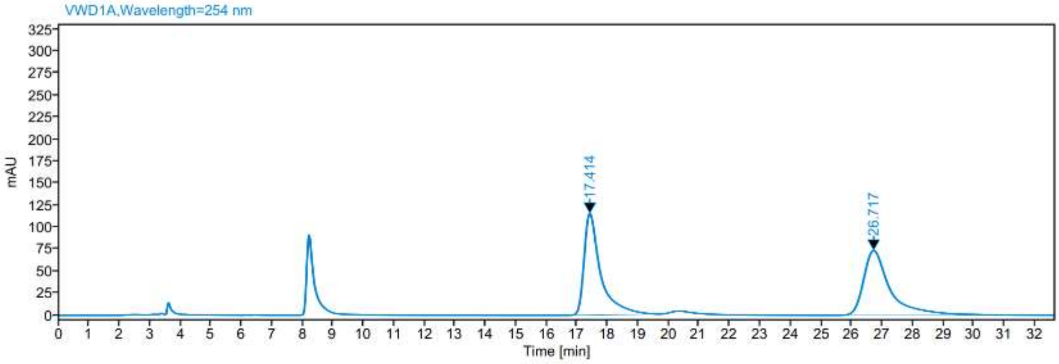

Signal: VWD1A,Wavelength=254 nm

RT [min]	Type	Width [min]	Area	Height	Area%	Name
17.414	BB	2.98	4329.78	115.22	49.01	
26.717	BB	8.15	4505.25	73.54	50.99	
		Sum	8835.03			

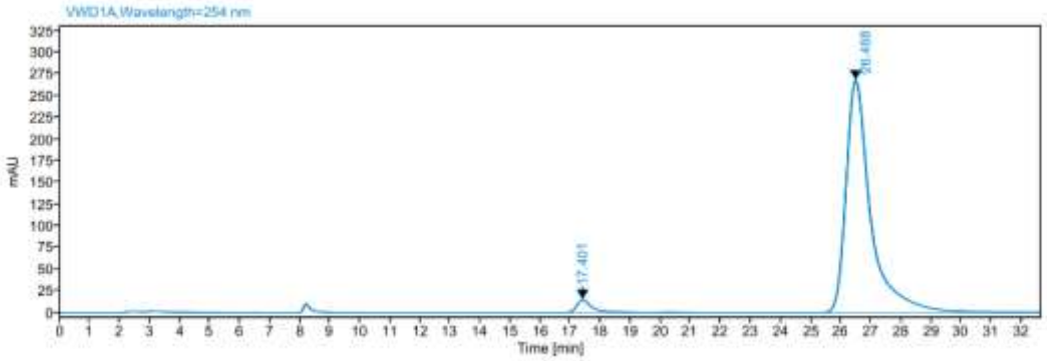

Signal: VWD1A,Wavelength=254 nm

RT [min]	Type	Width [min]	Area	Height	Area%	Name
17.401	BB	2.99	542.30	14.65	3.40	
26.488	BB	9.38	15405.00	267.61	96.60	
		Sum	15947.30			

(S)-dimethyl(((methyldiphenylphosphane)boryl)(naphthalen-2-yl)methyl)phosphonate(3Ba)

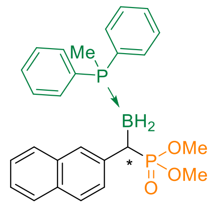

3Ba

Following the general procedure D, dimethyl(diazo(naphthalen-2-yl)methyl)phosphonate (0.20 mmol, 1.0 equiv) and methyldiphenylphosphane borane (0.40 mmol, 2.0 equiv) were employed and stirred at 20 °C for 36 h to afford **3Ba** as a colorless oil in 72% yield (66.7 mg) with 94% ee.

R_f = 0.42 (silica gel, EtOAc:PE = 3:1)

^{1}H NMR (400 MHz, Chloroform-*d*) δ 7.70 (d, *J* = 9.0 Hz, 1H), 7.64 – 7.55 (m, 2H), 7.50 – 7.29 (m, 12H), 7.28 – 7.20 (m, 2H), 3.62 (dd, *J* = 19.1, 10.5 Hz, 6H), 2.75 – 2.57 (m, 1H), 1.27 (d, *J* = 10.2 Hz, 3H).

^{13}C NMR (101 MHz, Chloroform-*d*) δ 139.0 (dd, *J* = 8.7, 5.4 Hz), 133.3 (d, *J* = 2.6 Hz), 131.7 (dd, *J* = 26.5, 8.9 Hz), 131.6 (d, *J* = 2.4 Hz), 131.0 (dd, *J* = 29.0, 2.5 Hz), 129.3 (d, *J* = 57.5 Hz), 128.7 (dd, *J* = 27.5, 10.0 Hz), 128.4 (d, *J* = 5.8 Hz), 127.7 (d, *J* = 55.5 Hz), 127.4 – 127.2 (m, 3C), 127.1 (d, *J* = 10.4 Hz), 125.4, 124.6, 52.7 (dd, *J* = 50.3, 7.0 Hz), 9.4 (d, *J* = 37.1 Hz).

^{11}B NMR (128 MHz, Chloroform-*d*) δ -25.99.

^{31}P NMR (162 MHz, Chloroform-*d*) δ 38.55 (d, *J* = 83.2 Hz), 5.51.

HRMS (ESI): calc'd for $(M+H)^+$ $C_{26}H_{30}BO_3P_2^+$ 463.1758, found 463.1770.

HPLC analysis: DAICEL CHIRALCEL IA-3, hexane/isopropanol = 80/20, 1 mL/min, λ = 254 nm, t_R (major) = 16.978 min, t_R (minor) = 13.350 min, 94% ee.

$[\alpha]^{25}_D$: +80.8 (*c* 0.5, $CHCl_3$).

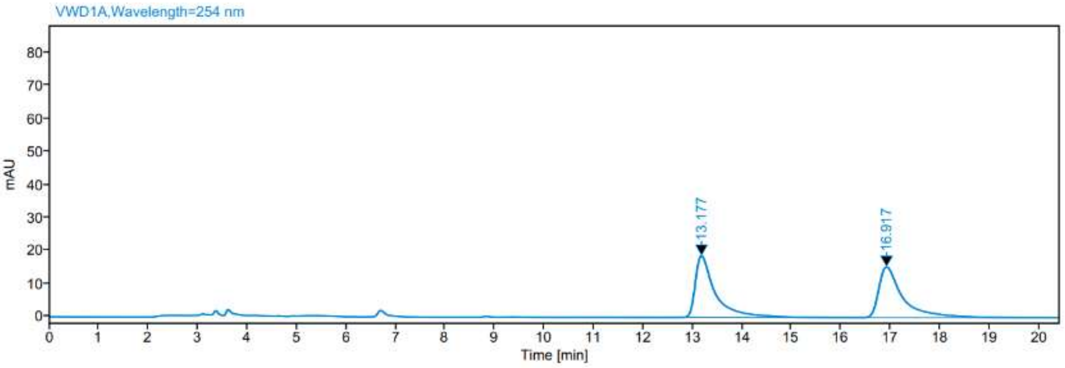

Signal: VWD1A,Wavelength=254 nm

RT [min]	Type	Width [min]	Area	Height	Area%	Name
13.177	BB	3.67	528.25	18.72	50.27	
16.917	BB	3.99	522.54	15.39	49.73	
		Sum	**1050.78**			

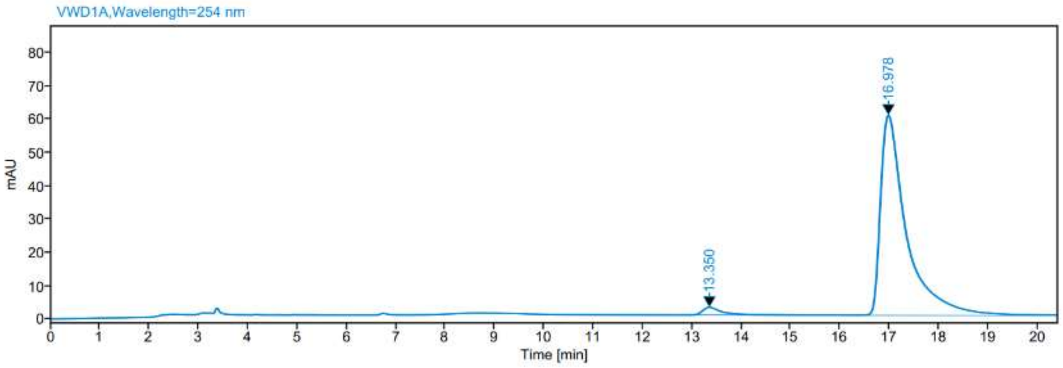

Signal: VWD1A,Wavelength=254 nm

RT [min]	Type	Width [min]	Area	Height	Area%	Name
13.350	BB	2.66	67.16	2.28	2.96	
16.978	BB	5.61	2200.44	60.00	97.04	
		Sum	**2267.60**			

(S)-dimethyl(((methyldiphenylphosphane)boryl)(naphthalen-1-yl)methyl)phosphonate(3Ca)

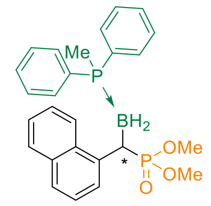

3Ca

Following the general procedure D, dimethyl (diazo(naphthalen-1-yl)methyl)phosphonate (0.20 mmol, 1.0 equiv) and methyldiphenylphosphane borane (0.40 mmol, 2.0 equiv) were employed and stirred at 20 °C for 12 h to afford **3Ca** as a colorless oil in 97% yield (89.8 mg) with 94% ee.

R$_f$ = 0.3 (silica gel, EtOAc:PE = 3:1)

^{1}H NMR (400 MHz, Chloroform-*d*) δ 7.94 (dd, *J* = 7.3, 3.7 Hz, 1H), 7.74 (d, *J* = 8.2 Hz, 1H), 7.56 (t, *J* = 8.9 Hz, 2H), 7.48 – 7.42 (m, 1H), 7.41 – 7.24 (m, 11H), 7.19 (t, *J* = 7.7 Hz, 1H), 3.56 (dd, *J* = 38.8, 10.5 Hz, 6H), 3.45 – 3.31 (m, 1H), 1.20 (d, *J* = 10.1 Hz, 3H).

^{13}C NMR (101 MHz, Chloroform-*d*) δ 137.3 (dd, *J* = 8.0, 5.6 Hz), 133.7 (d, *J* = 2.0 Hz), 131.6 (dd, *J* = 17.3, 8.9 Hz), 131.0 (dd, *J* = 15.8, 2.5 Hz), 129.4 (d, *J* = 57.6 Hz), 129.0 – 128.3 (m, 3C), 127.8 (d, *J* = 55.5 Hz), 127.7, 127.7, 125.5 (d, *J* = 4.2 Hz), 125.4 (d, *J* = 4.1 Hz), 125.1, 124.7, 123.1, 52.7 (dd, *J* = 51.3, 7.0 Hz), 9.3 (d, *J* = 36.7 Hz).

^{11}B NMR (128 MHz, Chloroform-*d*) δ -25.28.

^{31}P NMR (162 MHz, Chloroform-*d*) δ 38.94 (d, *J* = 80.9 Hz), 5.17.

HRMS (ESI): calc'd for $(M+H)^+$ $C_{26}H_{30}BO_3P_2^+$ 463.1758, found 463.1770.

HPLC analysis: DAICEL CHIRALCEL IA-3, hexane/isopropanol = 80/20, 1 mL/min, λ = 254 nm, t_R (major) = 13.785 min, t_R (minor) = 10.270 min, 94% ee.

$[\alpha]^{25}_{D}$: +80.8 (*c* 0.5, $CHCl_3$).

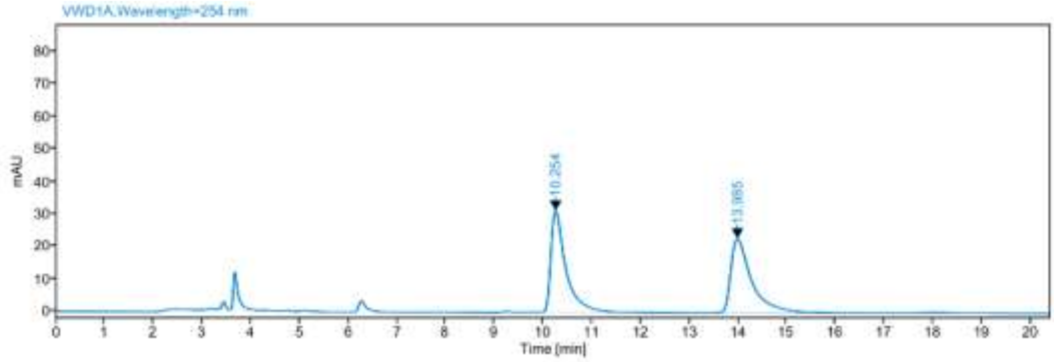

Signal: VWD1A,Wavelength=254 nm

RT [min]	Type	Width [min]	Area	Height	Area%	Name
10.254	BB	3.24	677.99	31.21	49.65	
13.985	BB	3.72	687.48	22.75	50.35	
		Sum	1365.47			

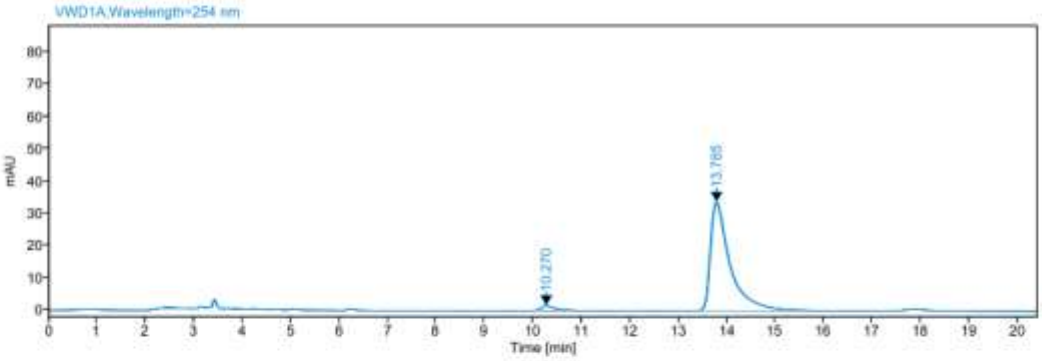

Signal: VWD1A,Wavelength=254 nm

RT [min]	Type	Width [min]	Area	Height	Area%	Name
10.270	MM m	1.61	29.92	1.49	3.05	
13.785	BB	3.60	950.08	33.57	96.95	
		Sum	980.01			

(S)-dimethyl(((ethyldiphenylphosphane)boryl)(phenyl)methyl)phosphonate(3ab)

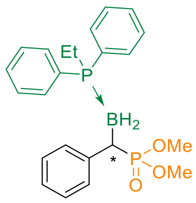

3ab

Following the general procedure D, dimethyl(diazo(phenyl)ethyl)phosphonate (0.20 mmol, 1.0 equiv) and ethyldiphenylphosphane borane (0.40 mmol, 2.0 equiv) were employed and stirred at 20 °C for 12 h to afford **3ab** as a colorless oil in 77% yield (65.5 mg) with 91% ee.

R_f = 0.48 (silica gel, EtOAc:PE = 3:1)

^{1}H NMR (400 MHz, Chloroform-*d*) δ 7.51 – 7.37 (m, 8H), 7.36 – 7.30 (m, 2H), 7.13 – 6.98 (m, 5H), 3.57 (dd, *J* = 21.1, 10.4 Hz, 6H), 2.39 – 2.23 (m, 2H), 1.99 – 1.83 (m, 1H), 1.45 – 1.29 (m, 1H), 0.79 (dt, *J* = 17.2, 7.5 Hz, 3H).

^{13}C NMR (101 MHz, Chloroform-*d*) δ 141.4 (dd, *J* = 8.8, 4.7 Hz), 132.4 (dd, *J* = 34.0, 8.2 Hz), 131.0 (d, *J* = 2.1 Hz), 129.3 (d, *J* = 7.9 Hz), 128.6 (dd, *J* = 18.4, 9.6 Hz), 127.8 (d, *J* = 2.9 Hz), 127.5 (t, *J* = 54.3 Hz), 125.0 (d, *J* = 3.5 Hz), 52.6 (dd, *J* = 43.5, 7.0 Hz), 16.0 (d, *J* = 34.4 Hz), 6.6.

^{11}B NMR (128 MHz, Chloroform-*d*) δ -27.98.

^{31}P NMR (162 MHz, Chloroform-*d*) δ 38.90 (d, *J* = 84.3 Hz), 14.17.

HRMS (ESI): calc'd for $(M+H)^+$ $C_{23}H_{30}BO_3P_2^+$ 427.1758, found 427.1778.

HPLC analysis: DAICEL CHIRALCEL AS-H, hexane/isopropanol = 80/20, 1 mL/min, λ = 254 nm, t_R (major) = 20.252 min, t_R (minor) = 8.056 min, 91% ee.

[α]$^{25}_{D}$: +54.8 (*c* 0.5, $CHCl_3$).

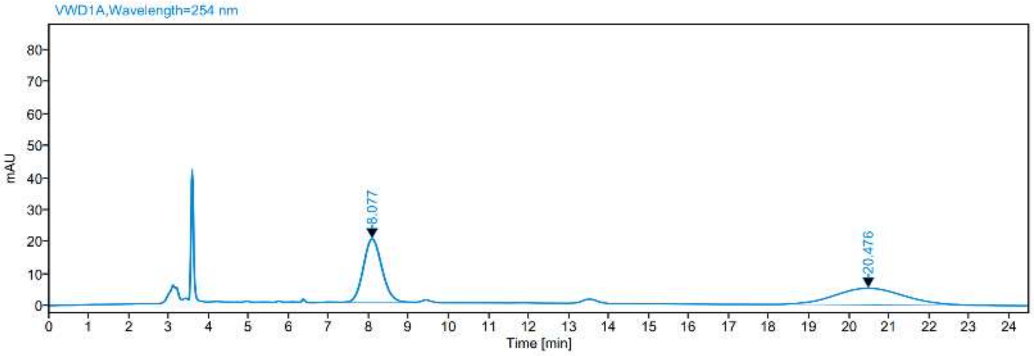

Signal: VWD1A,Wavelength=254 nm

RT [min]	Type	Width [min]	Area	Height	Area%	Name
8.077	BB	1.86	636.19	19.80	50.16	
20.476	BB	5.85	632.24	5.22	49.84	
		Sum	1268.43			

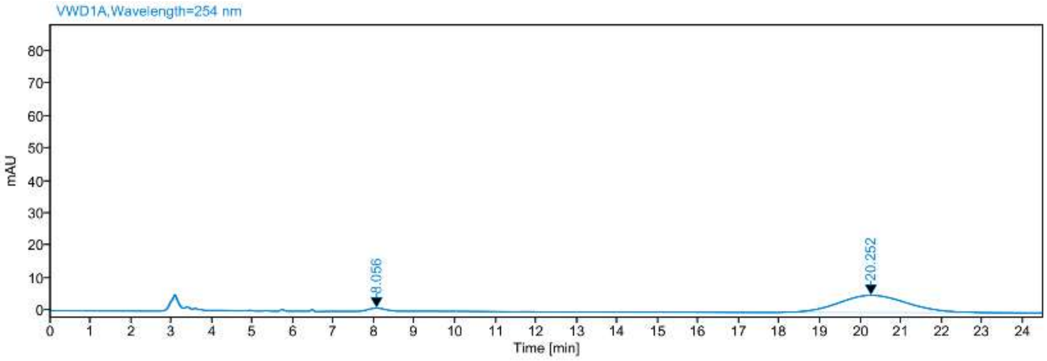

Signal: VWD1A,Wavelength=254 nm

RT [min]	Type	Width [min]	Area	Height	Area%	Name
8.056	MM m	1.49	29.06	0.98	4.39	
20.252	MM m	6.80	633.04	5.33	95.61	
		Sum	662.10			

(S)-dimethyl(((isopropyldiphenylphosphane)boryl)(phenyl)methyl)phosphonate(3ac)

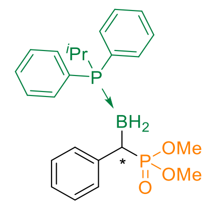

3ac

Following the general procedure D, dimethyl(diazo(phenyl)methyl)phosphonate (0.20 mmol, 1.0 equiv) and isopropyldiphenylphosphane borane (0.40 mmol, 2.0 equiv) were employed and stirred at 20 °C for 12 h to afford **3ac** as a colorless oil in 77% yield (68.1 mg) with 80% ee.

R_f = 0.48 (silica gel, EtOAc:PE = 3:1)

^{1}H NMR (400 MHz, Chloroform-*d*) δ 7.56 – 7.32 (m, 10H), 7.15 – 7.03 (m, 4H), 7.01 – 6.95 (m, 1H), 3.51 (dd, *J* = 21.4, 10.4 Hz, 6H), 2.25 – 2.10 (m, 2H), 0.98 (dd, *J* = 14.5, 7.0 Hz, 3H), 0.89 (dd, *J* = 15.9, 7.0 Hz, 3H).

^{13}C NMR (101 MHz, Chloroform-*d*) δ 139.9 (dd, *J* = 8.7, 3.9 Hz), 133.5 (dd, *J* = 43.2, 7.8 Hz), 131.0 (dd, *J* = 18.0, 2.5 Hz), 129.6 (d, *J* = 7.7 Hz), 128.4 (dd, *J* = 9.5, 2.8 Hz), 127.7 (d, *J* = 2.9 Hz), 125.3 (d, *J* = 16.0 Hz), 124.9, 124.8 (d, *J* = 10.5 Hz), 52.6 (dd, *J* = 44.3, 7.1 Hz), 22.5 (d, *J* = 32.2 Hz), 16.8 (d, *J* = 2.9 Hz), 16.7.

^{11}B NMR (128 MHz, Chloroform-*d*) δ -26.75.

^{31}P NMR (162 MHz, Chloroform-*d*) δ 39.07 (d, *J* = 81.2 Hz), 25.28.

HRMS (ESI): calc'd for $(M+H)^+$ $C_{24}H_{32}BO_3P_2^+$ 441.1914, found 441.1934.

HPLC analysis: DAICEL CHIRALCEL AS-H, hexane/isopropanol = 85/15, 1 mL/min, λ = 254 nm, t_R (major) = 24.569min, t_R (minor) = 9.162 min, 80% ee.

$[\alpha]^{25}_D$: +60.8 (*c* 0.25, $CHCl_3$).

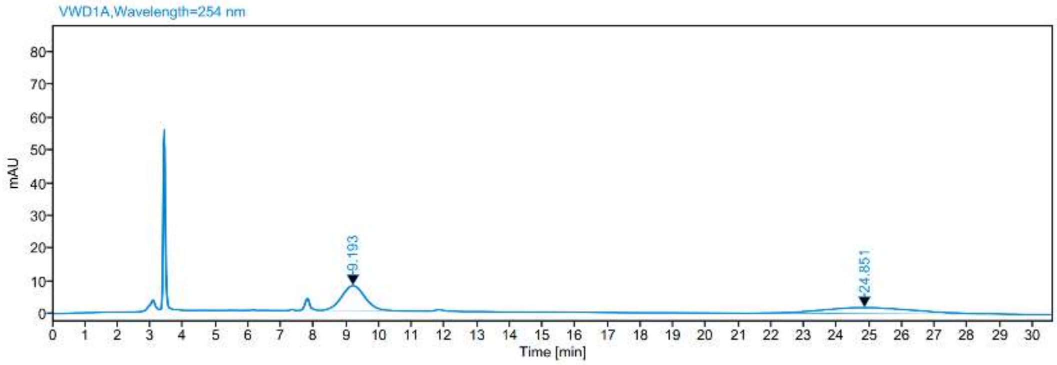

Signal: VWD1A,Wavelength=254 nm

RT [min]	Type	Width [min]	Area	Height	Area%	Name
9.193	MM m	2.12	370.15	7.56	50.84	
24.851	MM m	7.91	357.98	1.80	49.16	
		Sum	728.14			

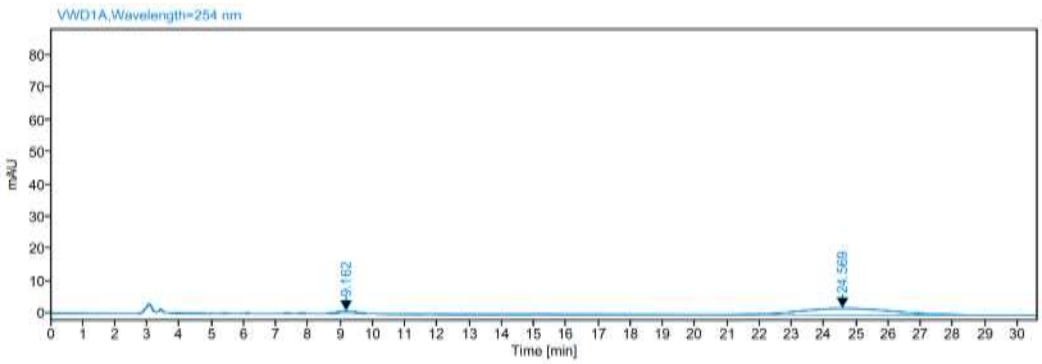

Signal: VWD1A,Wavelength=254 nm

RT [min]	Type	Width [min]	Area	Height	Area%	Name
9.162	MM m	2.43	45.29	0.94	9.78	
24.569	MM m	10.25	417.68	2.09	90.22	
		**Sum**	**462.97**			

(S)-dimethyl(((dimethyl(phenyl)phosphane)boryl)(phenyl)methyl)phosphonate(3ad)

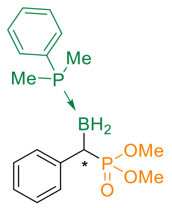

3ad

Following the general procedure D, dimethyl (diazo(phenyl)methyl)phosphonate (0.20 mmol, 1.0 equiv) and dimethyl(phenyl)phosphane borane (0.40 mmol, 2.0 equiv) were employed and stirred at 20 °C for 12 h to afford **3ad** as a colorless oil in 82% yield (57.7 mg) with 95% ee.

R*f* = 0.32 (silica gel, EtOAc:PE = 3:1)

^{1}H NMR (500 MHz, Chloroform-*d*) δ 7.52 – 7.38 (m, 5H), 7.21 – 7.16 (m, 2H), 7.12 (t, *J* = 7.5 Hz, 2H), 7.06 – 7.00 (m, 1H), 3.59 (dd, *J* = 10.3, 8.2 Hz, 6H), 2.55 – 2.43 (m, 1H), 1.29 (d, *J* = 10.7 Hz, 3H), 1.17 (d, *J* = 10.5 Hz, 3H).

^{13}C NMR (126 MHz, Chloroform-*d*) δ 141.6 (t, *J* = 7.1 Hz), 131.0, 130.5 (d, *J* = 8.6 Hz), 129.2 (d, *J* = 7.8 Hz), 129.2 (d, *J* = 54.8 Hz), 128.8 (d, *J* = 9.7 Hz), 128.0 (d, *J* = 2.7 Hz), 125.0 (d, *J* = 3.4 Hz), 52.6 (d, *J* = 62.8 Hz), 11.5 (d, *J* = 39.2 Hz), 9.5 (d, *J* = 37.2 Hz).

^{11}B NMR (202 MHz, Chloroform-*d*) δ -26.30 (d, *J* = 95.9 Hz).

^{31}P NMR (160 MHz, Chloroform-*d*) δ 39.27 (d, *J* = 77.6 Hz), -0.42.

HRMS (ESI): calc'd for $(M+H)^+$ $C_{17}H_{26}BO_3P_2^+$ 351.1445, found 351.1457

HPLC analysis: DAICEL CHIRALCEL IA-3, hexane/isopropanol = 85/15, 1 mL/min, λ = 254 nm,

t_R (major) = 14.190min, t_R (minor) = 11.937 min, 95% ee.

[α]^{25}$_D$: +46.2 (*c* 0.5, $CHCl_3$).

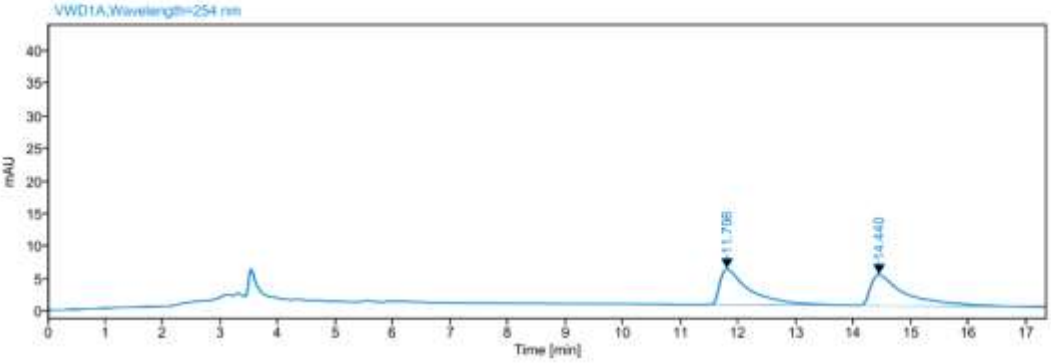

Signal: VWD1A,Wavelength=254 nm

RT [min]	Type	Width [min]	Area	Height	Area%	Name
11.798	BB	2.68	197.37	5.48	49.85	
14.440	BB	3.22	198.54	4.69	50.15	
		Sum	395.90			

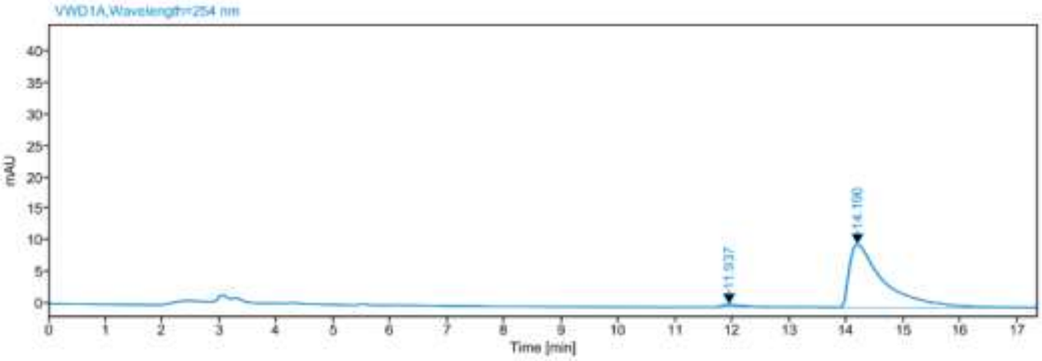

Signal: VWD1A,Wavelength=254 nm

RT [min]	Type	Width [min]	Area	Height	Area%	Name
11.937	MM m	1.78	10.80	0.42	2.51	
14.190	BB	3.47	419.69	9.98	97.49	
		Sum	430.50			

(S)-dimethyl(((diethyl(phenyl)phosphane)boryl)(phenyl)methyl)phosphonate(3ae)

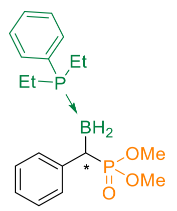

3ae

Following the general procedure D, dimethyl (diazo(phenyl)methyl)phosphonate (0.20 mmol, 1.0 equiv) and diethyl(phenyl)phosphane borane (0.40 mmol, 2.0 equiv) were employed and stirred at 20 °C for 12 h to afford **3ae** as a colorless oil in 64% yield (48.4 mg) with 92% ee.

R_f = 0.22 (silica gel, EtOAc:PE = 3:1)

^{1}H NMR (500 MHz, Chloroform-*d*) δ 7.52 – 7.38 (m, 5H), 7.21 – 7.17 (m, 2H), 7.12 (t, *J* = 7.4 Hz, 2H), 7.06 – 7.01 (m, 1H), 3.60 (dd, *J* = 17.5, 10.5 Hz, 6H), 2.49 – 2.37 (m, 1H), 1.78 – 1.64 (m, 2H), 1.56 – 1.45 (m, 1H), 1.43 – 1.32 (m, 1H), 0.95 – 0.87 (m, 3H), 0.87 – 0.78 (m, 3H).

^{13}C NMR (126 MHz, Chloroform-*d*) δ 141.9 (dd, *J* = 41.1, 8.2, 5.1 Hz), 131.7 (d, *J* = 7.4 Hz), 131.0 (d, *J* = 2.5 Hz), 129.2 (d, *J* = 7.9 Hz), 128.7 (d, *J* = 9.1 Hz), 127.9 (d, *J* = 2.7 Hz), 126.0 (d, *J* = 51.6 Hz), 125.0 (d, *J* = 3.4 Hz), 52.7 (dd, *J* = 62.9, 6.8 Hz), 15.8 (d, *J* = 35.7 Hz), 14.0 (d, *J* = 34.4 Hz), 6.5 (d, *J* = 2.4 Hz), 6.4 (d, *J* = 3.6 Hz).

^{11}B NMR (128 MHz, Chloroform-*d*) δ -28.58.

^{31}P NMR (202 MHz, Chloroform-*d*) δ 39.57 (d, *J* = 77.7 Hz), 14.88.

HRMS (ESI): calc'd for $(M+H)^+$ $C_{19}H_{30}BO_3P_2^+$ 379.1763, found 379.1755.

HPLC analysis: DAICEL CHIRALCEL AD-H, hexane/isopropanol = 80/20, 1 mL/min, λ = 254 nm, t_R (major) = 6.715min, t_R (minor) = 6.190 min, 92% ee.

$[\alpha]^{25}_{D}$: +102.0 (*c* 0.1, $CHCl_3$).

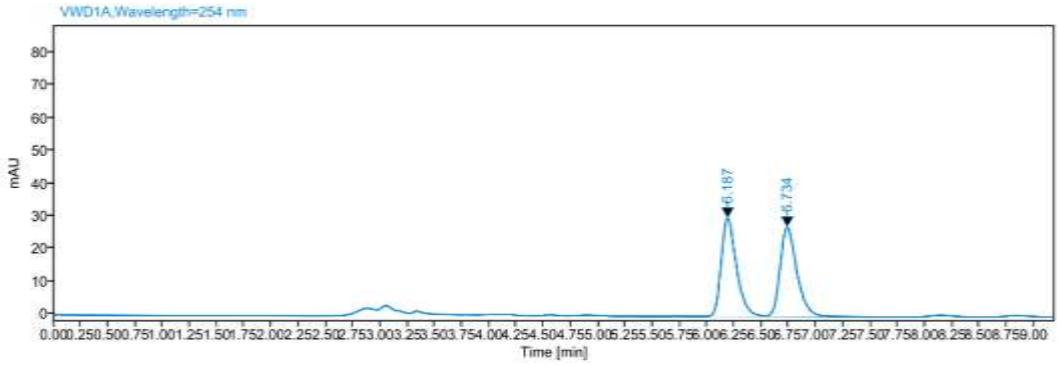

Signal: VWD1A,Wavelength=254 nm

RT [min]	Type	Width [min]	Area	Height	Area%	Name
6.187	BV	0.55	292.34	30.03	49.96	
6.734	VB	1.06	292.84	27.31	50.04	
		Sum	585.18			

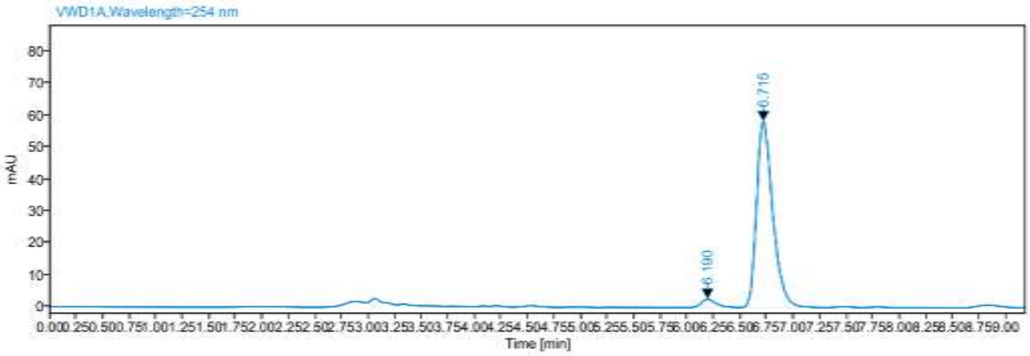

Signal: VWD1A,Wavelength=254 nm

RT [min]	Type	Width [min]	Area	Height	Area%	Name
6.190	BV	0.48	24.65	2.59	3.83	
6.715	VB	0.82	618.35	58.28	96.17	
		Sum	642.99			

(S)-dimethyl(((dicyclohexyl(phenyl)phosphane)boryl)(phenyl)methyl)phosphonate(3af)

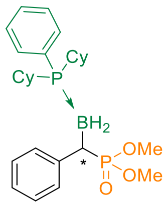

3af

Following the general procedure D, dimethyl (diazo(phenyl)methyl)phosphonate (0.20 mmol, 1.0 equiv) and dicyclohexyl(phenyl)phosphane borane (0.40 mmol, 2.0 equiv) were employed and stirred at 20 °C for 12 h to afford **3af** as a colorless oil in 48% yield (46.2 mg) with 56% ee.

R_f = 0.61 (silica gel, EtOAc:PE = 3:1).

^{1}H NMR (400 MHz, Chloroform-*d*) δ 7.62 – 7.53 (m, 2H), 7.47 – 7.32 (m, 5H), 7.17 (t, *J* = 7.5 Hz, 2H), 7.10 – 7.01 (m, 1H), 3.61 (dd, *J* = 47.0, 10.4 Hz, 6H), 2.72 – 2.55 (m, 1H), 2.16 – 2.04 (m, 1H), 1.86 – 1.44 (m, 11H), 1.30 – 1.03 (m, 7H), 0.91 (d, *J* = 12.5 Hz, 2H), 0.79 – 0.65 (m, 1H).

^{13}C NMR (101 MHz, Chloroform-*d*) δ 142.1 (d, *J* = 7.8 Hz), 133.3 (d, *J* = 6.6 Hz), 130.8 (d, *J* = 2.5 Hz), 130.0 (d, *J* = 7.6 Hz), 128.3 (d, *J* = 9.0 Hz), 127.8 (d, *J* = 3.2 Hz), 124.9 (d, *J* = 3.9 Hz), 124.0 (d, *J* = 49.0 Hz), 52.8 (dd, *J* = 49.6, 7.1 Hz), 30.9 (dd, *J* = 30.9, 20.4 Hz), 27.2 – 26.1 (m, 8C), 25.7 (d, *J* = 8.4 Hz).

^{31}P NMR (162 MHz, Chloroform-*d*) δ 39.12 (d, *J* = 78.9 Hz), 16.91.

^{11}B NMR (128 MHz, Chloroform-*d*) δ -29.53.

HRMS (ESI): calc'd for $(M+H)^+$ $C_{27}H_{42}BO_3P_2^+$ 487.2697, found 487.2695.

HPLC analysis: DAICEL CHIRALCEL IA-3, hexane/isopropanol = 80/20, 1 mL/min, λ = 254 nm, t_R (major) = 15.159min, t_R (minor) = 13.182 min, 56% ee.

[α]^{25}$_D$: +8.6 (*c* 0.5, $CHCl_3$).

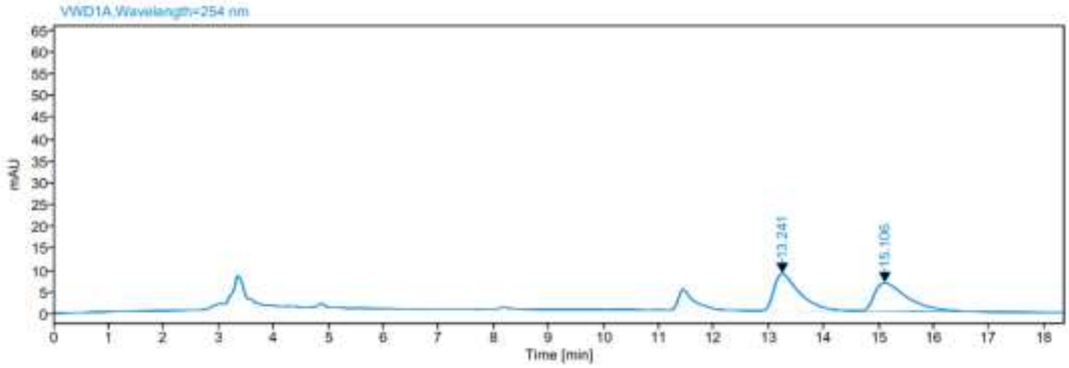

Signal: VWD1A,Wavelength=254 nm

RT [min]	Type	Width [min]	Area	Height	Area%	Name
13.241	BB	1.83	272.84	8.45	50.19	
15.106	BB	2.82	270.79	6.46	49.81	
		Sum	543.63			

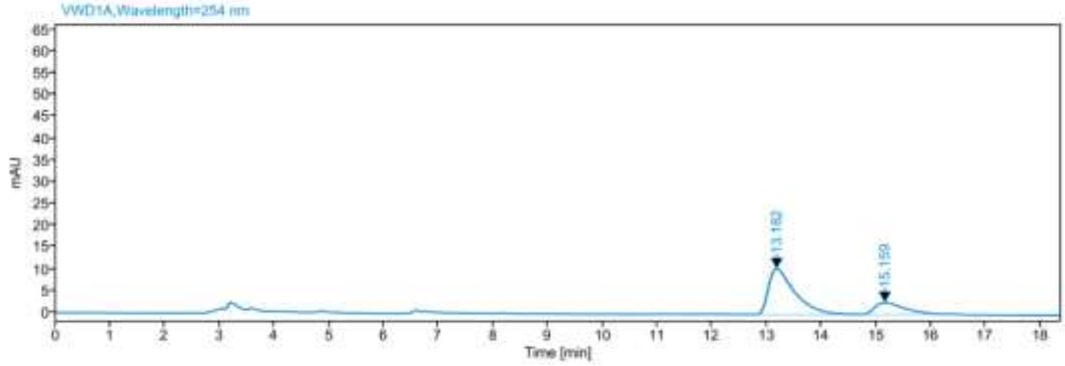

Signal: VWD1A,Wavelength=254 nm

RT [min]	Type	Width [min]	Area	Height	Area%	Name
13.182	BB	1.95	350.67	10.52	77.93	
15.159	BB	1.51	99.32	2.72	22.07	
		Sum	449.99			

(S)-dimethyl(((trimethylphosphane)boryl)([1,1'-biphenyl]-4-yl)methyl)phosphonate(3lg)

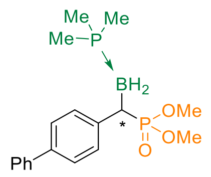

3lg

Following the general procedure D, dimethyl ([1,1'-biphenyl]-4-yl(diazo)methyl)phosphonate (0.20 mmol, 1.0 equiv) and trimethylphosphane borane (0.40 mmol, 2.0 equiv) were employed and stirred

at 20 °C for 12 h to afford **3lg** as a colorless oil in 62% yield (44.9 mg) with 94% ee.

R_f = 0.15 (silica gel, EtOAc:PE = 3:1)

^{1}H NMR (400 MHz, Chloroform-*d*) δ 7.61 – 7.53 (m, 2H), 7.51 – 7.44 (m, 2H), 7.44 – 7.36 (m, 4H), 7.32 – 7.27 (m, 1H), 3.67 (dd, *J* = 10.5, 2.8 Hz, 6H), 2.71 – 2.53 (m, 1H), 1.12 (d, *J* = 10.8 Hz, 9H).

^{13}C NMR (101 MHz, Chloroform-*d*) δ 141.2 (t, *J* = 8.4 Hz), 141.0, 137.8 (d, *J* = 3.5 Hz), 129.5 (d, *J* = 7.8 Hz), 128.6, 126.8 (d, *J* = 2.6 Hz), 126.8, 126.7, 52.7 (dd, *J* = 53.9, 7.0 Hz), 10.9 (d, *J* = 37.8 Hz).

^{11}B NMR (128 MHz, Chloroform-*d*) δ -25.14 (d, *J* = 83.7 Hz).

^{31}P NMR (162 MHz, Chloroform-*d*) δ 38.68 (d, *J* = 68.3 Hz), -5.94.

HRMS (ESI): calc'd for $(M+H)^+$ $C_{18}H_{28}BO_3P_2^+$ 365.1601, found 365.1601.

HPLC analysis: DAICEL CHIRALCEL ID-3, hexane/isopropanol = 80/20, 1 mL/min, λ = 254 nm, t_R (major) = 36.678 min, t_R (minor) = 26.971 min, 94% ee.

$[\alpha]^{25}_D$: +36.8 (*c* 0.5, $CHCl_3$).

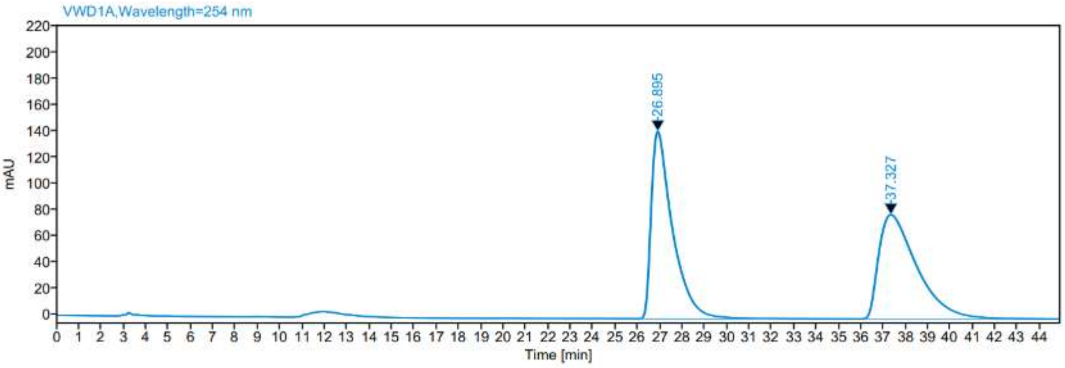

Signal: VWD1A,Wavelength=254 nm

RT [min]	Type	Width [min]	Area	Height	Area%	Name
26.895	BB	7.52	9493.18	142.67	50.07	
37.327	BB	9.08	9467.34	79.54	49.93	
		**Sum**	**18960.52**			

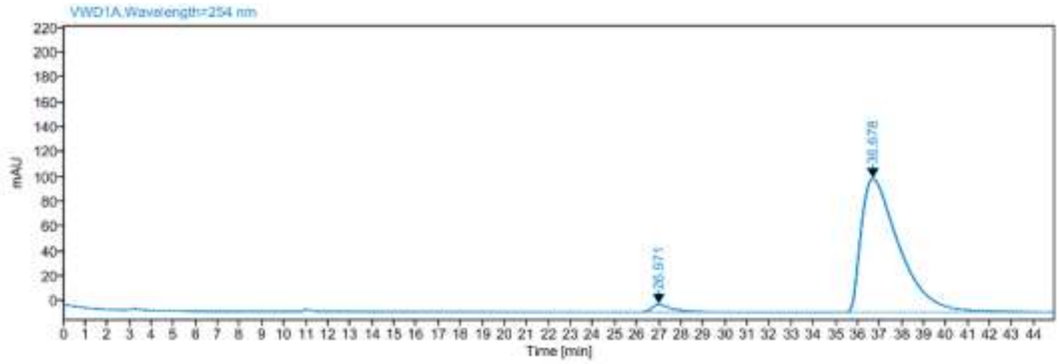

Signal: VWD1A,Wavelength=254 nm

RT [min]	Type	Width [min]	Area	Height	Area%	Name
26.971	BB	4.09	378.97	6.04	2.88	
36.678	BB	11.06	12796.93	107.25	97.12	
		Sum	13175.90			

(S)-dimethyl(((tributylphosphane)boryl)([1,1'-biphenyl]-4-yl)methyl)phosphonate(3lh)

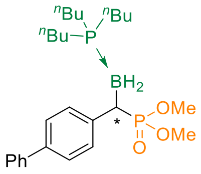

3lh

Following the general procedure D, dimethyl ([1,1'-biphenyl]-4-yl(diazo)methyl)phosphonate (0.20 mmol, 1.0 equiv) and tributylphosphane borane (0.40 mmol, 2.0 equiv) were employed and stirred at 20 °C for 12 h to afford **3lh** as a colorless oil in 82% yield (80.0 mg) with 93% ee.

R*f* = 0.72 (silica gel, EtOAc:PE = 3:1)

^{1}H NMR (500 MHz, Chloroform-*d*) δ 7.56 – 7.51 (m, 2H), 7.44 (d, *J* = 8.0 Hz, 2H), 7.42 – 7.37 (m, 4H), 7.30 – 7.26 (m, 1H), 3.66 (dd, *J* = 14.2, 10.5 Hz, 6H), 2.59 – 2.48 (m, 1H), 1.45 – 1.36 (m, 3H), 1.32 – 1.21 (m, 15H), 0.84 (t, *J* = 7.0 Hz, 9H).

^{13}C NMR (126 MHz, Chloroform-*d*) δ 141.6 (dd, *J* = 8.7, 5.2 Hz), 141.1, 137.8 (d, *J* = 3.6 Hz), 129.5 (d, *J* = 7.8 Hz), 128.5, 126.7, 52.7 (dd, *J* = 52.5, 7.0 Hz), 24.3 (d, *J* = 3.2 Hz), 24.2 (d, *J* = 6.6 Hz), 20.6 (d, *J* = 33.3 Hz), 13.4.

^{11}B NMR (128 MHz, Chloroform-*d*) δ -28.05.

^{31}P NMR (202 MHz, Chloroform-*d*) δ 39.44 (d, *J* = 75.8 Hz), 9.29.

HRMS (ESI): calc'd for $(M+H)^+$ $C_{27}H_{46}BO_3P_2^+$ 491.3010, found 491.3010.

HPLC analysis: DAICEL CHIRALCEL AD-H, hexane/isopropanol = 90/10, 1 mL/min, λ = 254

nm, t_R (major) = 5.987 min, t_R (minor) = 5.549 min, 93% ee.

$[\alpha]^{25}{}_D$: +45.0 (*c* 0.5, $CHCl_3$).

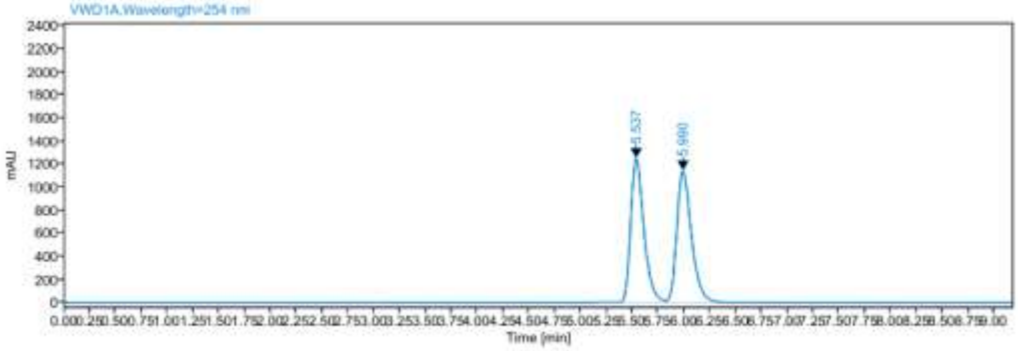

Signal: VWD1A,Wavelength=254 nm

RT [min]	Type	Width [min]	Area	Height	Area%	Name
5.537	VV	0.45	11187.92	1251.36	49.50	
5.990	VV	0.72	11414.42	1144.41	50.50	
		Sum	22602.34			

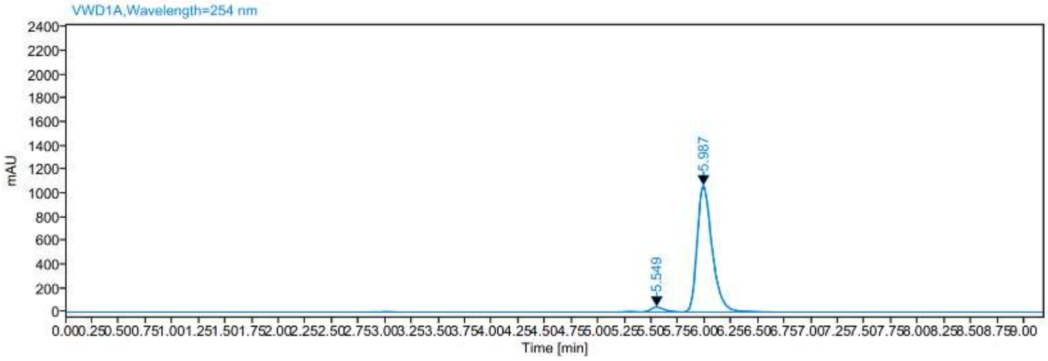

Signal: VWD1A,Wavelength=254 nm

RT [min]	Type	Width [min]	Area	Height	Area%	Name
5.549	VV	0.38	373.78	42.34	3.45	
5.987	VB	1.86	10463.83	1062.27	96.55	
		Sum	10837.60			

(S)-dimethyl(((trimethylphosphite)boryl)([1,1'-biphenyl]-4-yl)methyl)phosphonate(3li)

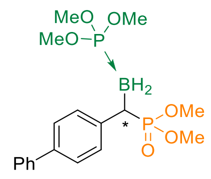

3li

Following the general procedure D, dimethyl([1,1'-biphenyl]-4-yl(diazo)methyl)phosphonate (0.20 mmol, 1.0 equiv) and trimethylphosphite borane (0.40 mmol, 2.0 equiv) were employed and stirred at 20 °C for 12 h to afford **3li** as a white solid in 71% yield (58.4 mg) with 93% ee.

mp = 71.9 – 72.5 ℃

R$_f$ = 0.10 (silica gel, EtOAc)

^{1}H NMR (500 MHz, Chloroform-*d*) δ 7.59 – 7.55 (m, 2H), 7.47 (d, *J* = 7.9 Hz, 2H), 7.43 – 7.37 (m, 4H), 7.32 – 7.27 (m, 1H), 3.66 (dd, *J* = 33.9, 10.3 Hz, 6H), 3.53 (d, *J* = 10.6 Hz, 9H), 2.67 – 2.51 (m, 1H).

^{13}C NMR (101 MHz, Chloroform-*d*) δ 141.2 (dd, *J* = 8.2, 6.0 Hz), 141.0, 137.5 (d, *J* = 3.4 Hz), 129.5 (d, *J* = 8.0 Hz), 128.6, 126.7, 126.7, 126.5 (d, *J* = 2.6 Hz), 53.1 (d, *J* = 5.0 Hz), 53.0 (d, *J* = 6.8 Hz), 52.4 (d, *J* = 6.9 Hz).

^{11}B NMR (160 MHz, Chloroform-*d*) δ -32.52 (d, *J* = 105.6 Hz).

^{31}P NMR (202 MHz, Chloroform-*d*) δ 106.12, 39.16 (d, *J* = 101.0 Hz).

HRMS (ESI): calc'd for $(M+H)^+$ $C_{18}H_{28}BO_6P_2$+ 413.1449, found 413.1448.

HPLC analysis: DAICEL CHIRALCEL AD-H, hexane/isopropanol = 70/30, 1 mL/min, λ = 254 nm, t_R (major) = 6.109 min, t_R (minor) = 5.149 min, 93% ee.

$[\alpha]^{25}_D$: +67.2 (*c* 0.5, $CHCl_3$).

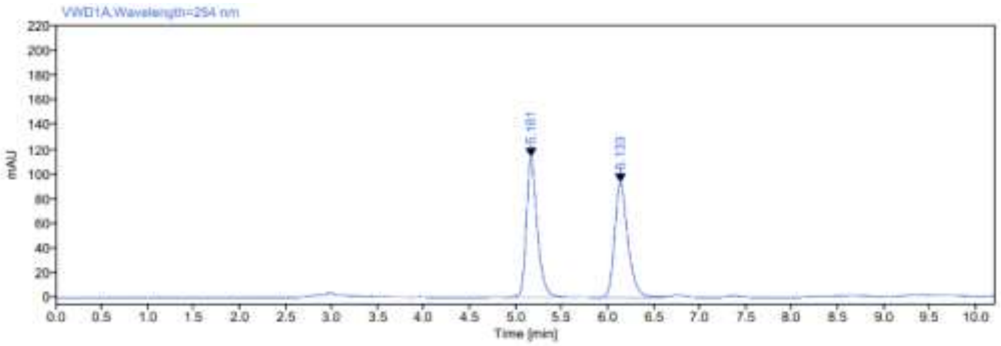

Signal: VWD1A,Wavelength=254 nm

RT [min]	Type	Width [min]	Area	Height	Area%	Name
5.161	VB	0.75	932.21	112.77	50.04	
6.133	BV	0.71	930.84	92.24	49.96	
		Sum	1863.04			

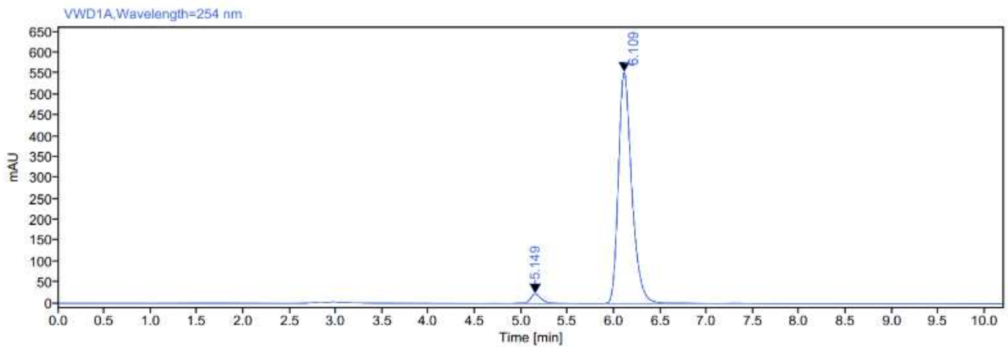

Signal: VWD1A,Wavelength=254 nm

RT [min]	Type	Width [min]	Area	Height	Area%	Name
5.149	BB	0.89	193.37	22.94	3.35	
6.109	BB	1.29	5576.40	553.78	96.65	
		**Sum**	**5769.78**			

(S)-diethyl(((methyldiphenylphosphane)boryl)(phenyl)methyl)phosphonate(5aa)

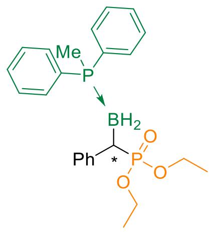

5aa

Following the general procedure D, diethyl (diazo(phenyl)methyl)phosphonate (0.20 mmol, 1.0 equiv) and methyldiphenylphosphane borane (0.40 mmol, 2.0 equiv) were employed and stirred at 20 °C for 12 h to afford **5aa** as a white solid in 77% yield (67.8 mg) with 92% ee.

mp: 112.2 – 113.3 °C

$\mathbf{R_f}$ = 0.56 (silica gel, EtOAc:PE = 3:1)

^{1}H NMR (500 MHz, Chloroform-*d*) δ 7.53 – 7.37 (m, 8H), 7.32 (t, *J* = 7.5 Hz, 2H), 7.15 – 7.05 (m, 4H), 7.00 (t, *J* = 7.2 Hz, 1H), 4.04 – 3.80 (m, 4H), 2.48 – 2.33 (m, 1H), 1.30 (d, *J* = 10.2 Hz, 3H), 1.17 (t, *J* = 6.9 Hz, 3H), 1.11 (t, *J* = 7.0 Hz, 3H).

^{13}C NMR (101 MHz, Chloroform-*d*) δ 141.8 (dd, *J* = 8.4, 5.6 Hz), 131.8 (dd, *J* = 20.9, 8.8 Hz), 131.0 (dd, *J* = 21.6, 2.5 Hz), 130.1, 129.5 (d, *J* = 7.8 Hz), 128.7 (dd, *J* = 22.7, 9.9 Hz), 127.9 (d, *J* = 54.8 Hz), 127.8 (d, *J* = 2.8 Hz), 124.8 (d, *J* = 3.4 Hz), 61.4 (dd, *J* = 61.0, 6.9 Hz), 16.3 (t, *J* = 5.7 Hz), 9.3 (d, *J* = 36.6 Hz).

^{11}B NMR (160 MHz, Chloroform-*d*) δ -26.75.

^{31}P NMR (202 MHz, Chloroform-*d*) δ 36.86 (d, *J* = 82.3 Hz), 6.44.

HRMS (ESI): calc'd for $(M+H)^+$ $C_{24}H_{32}BO_3P_2^+$ 441.1914, found 441.1926.

HPLC analysis: DAICEL CHIRALCEL AD-H, hexane/isopropanol = 70/30, 1 mL/min, λ = 254 nm, t_R (major) = 7.087 min, t_R (minor) = 5.908 min, 92% ee.

$[\alpha]^{25}_D$: +29.8 (*c* 0.5, $CHCl_3$).

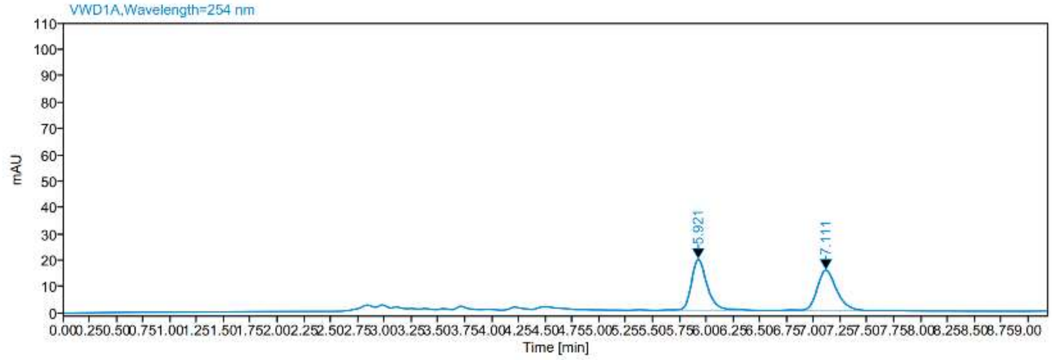

Signal: VWD1A,Wavelength=254 nm

RT [min]	Type	Width [min]	Area	Height	Area%	Name
5.921	BB	1.09	200.41	19.43	50.96	
7.111	BB	0.94	192.88	15.35	49.04	
		Sum	393.29			

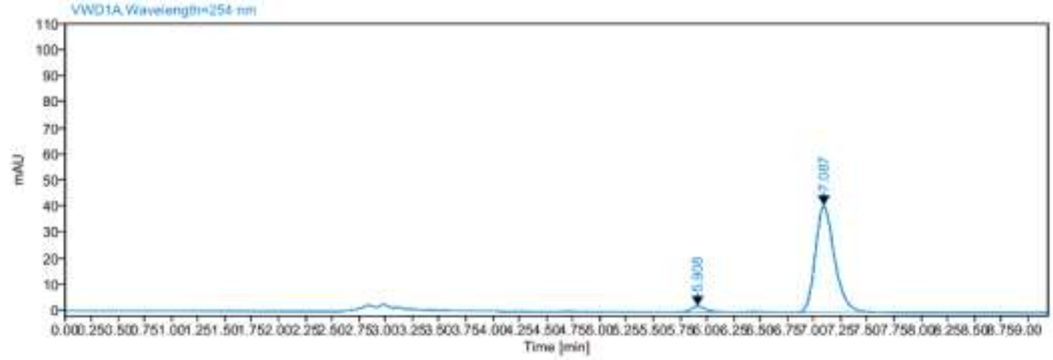

Signal: VWD1A,Wavelength=254 nm

RT [min]	Type	Width [min]	Area	Height	Area%	Name
5.908	BB	0.59	21.62	2.17	4.07	
7.087	BB	1.59	510.07	40.76	95.93	
		Sum	531.69			

(S)-diisopropyl(((methyldiphenylphosphane)boryl)(phenyl)methyl)phosphonate(5ba)

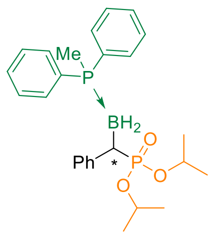

5ba

Following the general procedure D, diisopropyl(diazo(phenyl)methyl)phosphonate (0.20 mmol, 1.0

equiv) and methyldiphenylphosphane borane (0.40 mmol, 2.0 equiv) were employed and stirred at 20 °C for 12 h to afford **5ba** as a yellow oil in 94% yield (88.4 mg) with 90% ee.

R_f = 0.67 (silica gel, EtOAc:PE = 3:1)

^{1}H NMR (400 MHz, Chloroform-*d*) δ 7.51 – 7.36 (m, 8H), 7.35 – 7.28 (m, 2H), 7.15 – 7.02 (m, 4H), 6.98 (dd, J = 8.3, 6.1 Hz, 1H), 4.68 – 4.42 (m, 2H), 2.33 (m, 1H), 1.29 (d, J = 10.2 Hz, 3H), 1.23 – 1.15 (m, 9H), 0.89 (d, J = 6.2 Hz, 3H).

^{13}C NMR (101 MHz, Chloroform-*d*) δ 142.5 (dd, J = 8.3, 4.9 Hz), 131.8 (dd, J = 20.3, 8.9 Hz), 131.0 (dd, J = 20.5, 2.5 Hz), 130.0 (d, J = 58.9 Hz), 129.6 (d, J = 7.9 Hz), 128.7 (dd, J = 21.8, 9.9 Hz), 128.1 (d, J = 54.4 Hz), 127.6 (d, J = 2.7 Hz), 124.6 (d, J = 3.4 Hz), 69.3 (dd, J = 64.5, 7.3 Hz), 24.2 (dd, J = 23.4, 3.0 Hz), 23.6 (dd, J = 64.6, 5.7 Hz), 9.3 (d, J = 36.3 Hz).

^{11}B NMR (128 MHz, Chloroform-*d*) δ -25.90.

^{31}P NMR (162 MHz, Chloroform-*d*) δ 34.57 (d, J = 85.4 Hz), 6.20.

HRMS (ESI): calc'd for $(M+H)^+$ $C_{26}H_{36}BO_3P_2^+$ 469.2227, found 469.2227.

HPLC analysis: DAICEL CHIRALCEL IA-3, hexane/isopropanol = 90/10, 1 mL/min, λ = 254 nm, t_R (major) = 17.076 min, t_R (minor) = 14.861 min, 90% ee.

$[\alpha]^{25}_D$: +49.0 (c 0.5, $CHCl_3$).

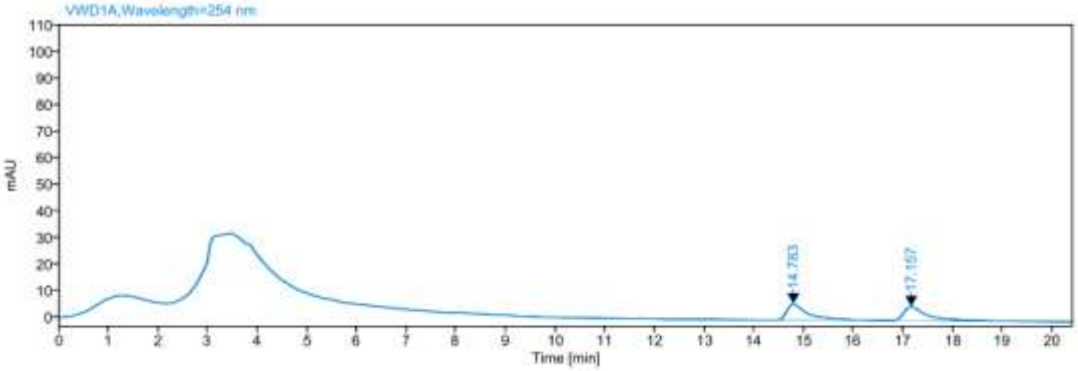

Signal: VWD1A,Wavelength=254 nm

RT [min]	Type	Width [min]	Area	Height	Area%	Name
14.783	BB	2.35	184.37	5.97	50.68	
17.157	BB	2.58	179.44	5.21	49.32	
		Sum	363.80			

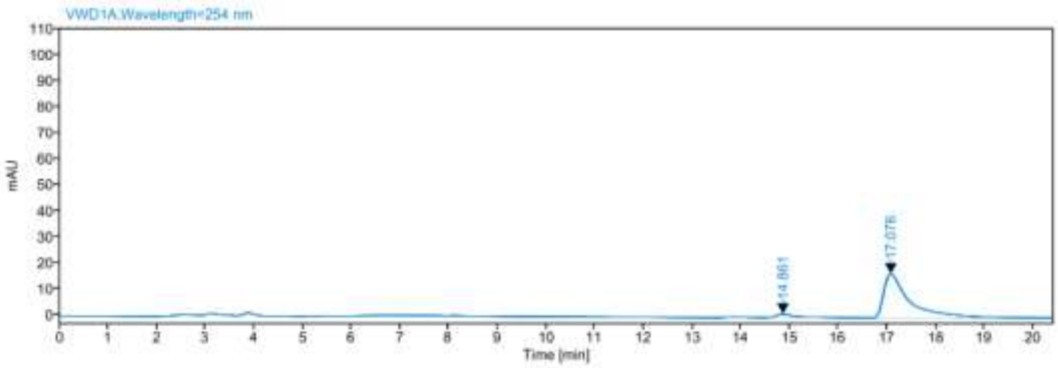

Signal: VWD1A,Wavelength=254 nm

RT [min]	Type	Width [min]	Area	Height	Area%	Name
14.861	MM m	1.18	32.92	1.33	4.93	
17.076	BB	3.71	634.45	16.90	95.07	
		Sum	667.36			

(S)-dihexyl(((methyldiphenylphosphane)boryl)(phenyl)methyl)phosphonate(5ca)

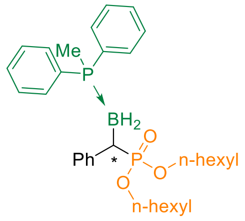

5ca

Following the general procedure D, dihexyl(diazo(phenyl)methyl)phosphonate (0.20 mmol, 1.0 equiv) and methyldiphenylphosphane borane (0.40 mmol, 2.0 equiv) were employed and stirred at 20 °C for 12 h to afford **5ca** as a yellow oil in 91% yield (100.6 mg) with 92% ee.

$\mathbf{R}_{\boldsymbol{f}}$ = 0.86 (silica gel, EtOAc:PE = 3:1)

^{1}H NMR (400 MHz, Chloroform-*d*) δ 7.51 – 7.36 (m, 8H), 7.34 – 7.28 (m, 2H), 7.15 – 7.04 (m, 4H), 7.03 – 6.95 (m, 1H), 3.95 – 3.74 (m, 4H), 2.49 – 2.33 (m, 1H), 1.56 – 1.40 (m, 4H), 1.31 (d, *J* = 10.2 Hz, 3H), 1.27 – 1.14 (m, 12H), 0.83 (t, *J* = 6.7 Hz, 6H).

^{13}C NMR (101 MHz, Chloroform-*d*) δ 141.9 (dd, *J* = 8.5, 5.3 Hz), 131.8 (dd, *J* = 19.3, 8.9 Hz), 131.0 (dd, *J* = 19.9, 2.5 Hz), 129.8 (d, *J* = 57.6 Hz), 129.5 (d, *J* = 7.7 Hz), 128.7 (dd, *J* = 22.1, 9.9 Hz), 128.0 (d, *J* = 54.8 Hz), 127.7 (d, *J* = 2.8 Hz), 124.8 (d, *J* = 3.5 Hz), 65.5 (dd, *J* = 58.7, 7.2 Hz), 31.3, 30.5 (d, *J* = 6.3 Hz), 25.1 (d, *J* = 2.6 Hz), 22.4 (d, *J* = 1.7 Hz), 13.9, 9.3 (d, *J* = 36.6 Hz).

^{31}P NMR (162 MHz, Chloroform-*d*) δ 35.94 (d, *J* = 84.0 Hz), 5.84.

^{11}B NMR (128 MHz, Chloroform-*d*) δ -25.97.

HRMS (ESI): calc'd for $(M+H)^+$ $C_{32}H_{48}BO_3P_2^+$ 553.3166, found 553.3166.

HPLC analysis: DAICEL CHIRALCEL IA-3, hexane/isopropanol = 85/15, 1 mL/min, λ = 254 nm,

t_R (major) = 12.220 min, t_R (minor) = 10.736 min, 92% ee.

[α]^{25}$_D$: +36.4 (*c* 0.5, $CHCl_3$).

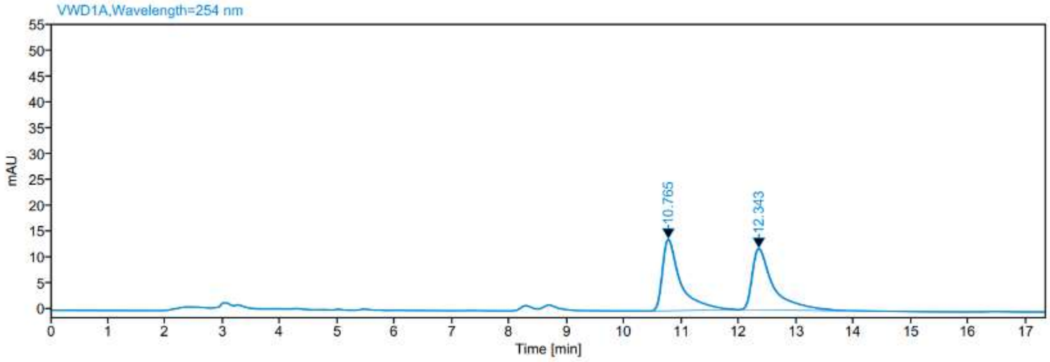

Signal:	VWD1A,Wavelength=254 nm					
RT [min]	Type	Width [min]	Area	Height	Area%	Name
10.765	BB	1.58	303.63	13.81	49.81	
12.343	BB	2.54	305.89	11.84	50.19	
		**Sum**	**609.53**			

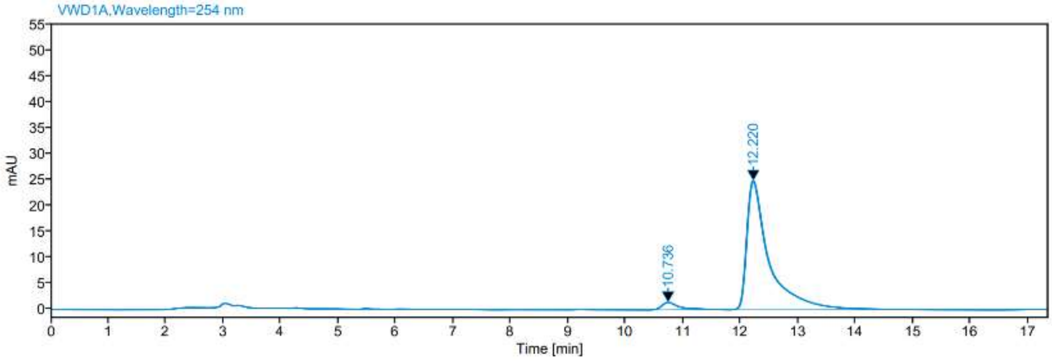

Signal:	VWD1A,Wavelength=254 nm					
RT [min]	Type	Width [min]	Area	Height	Area%	Name
10.736	MM m	1.04	28.13	1.40	4.03	
12.220	BB	3.21	669.54	24.86	95.97	
		**Sum**	**697.67**			

1.5 Further synthetic transformation

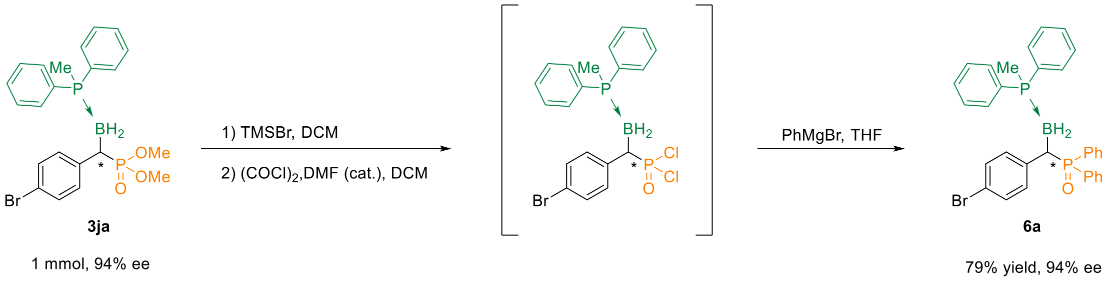

(S)- (((methyldiphenylphosphane)boryl)(4-bromophenyl)methyl)diphenylphosphine oxide (6a)

An oven-dried 25 mL vial with a magnetic stir bar was charged with (S)-dimethy((methyldiphenylphosphane-boryl)(4-bromophenyl)methyl)phosphonate (**3ja**, 491.1 mg,

1.0 mmol, 1.0 equiv) and capped under argon. DCM (10 mL) was added via syringe and the mixture was stirred for 1 min. TMSBr (0.4 mL, 3.0 mmol, 3.0 equiv) was added dropwise under r.t., and the mixture was stirred for 30 min under r.t.

Volatiles were removed under reduced pressure. The crude residue was redissolved in DCM (10 mL) and DMF (50 μL) was added, followed by $(COCl)_2$ (0.35 mL, 4.0 mmol, 4.0 equiv) under 0 °C. The mixture was warmed to r.t. and stirred for 2 h.

The mixture was concentrated under reduced pressure and THF (10 mL) was added. Then, PhMgBr (6.0 mL, 6.0 mmol, 6.0 equiv, 1.0 mol in THF) were added dropwise under -78 °C. After the addition is completed, the mixture was warmed to r.t. and stirred overnight.

Aqueous NH_4Cl (10 mL) was added and the mixture was extracted with EtOAc (5 mL×3). The organic layers were combined, washed with brine (5.0 mL), dried over Na_2SO_4, and concentrated under reduced pressure. The crude product was purified by flash column chromatography to afford **6a** as a white solid in 79% yield (462.7 mg) with 94% ee.[10]

mp: 220.1 – 221.1 °C

R*f* = 0.31 (silica gel, EtOAc:PE = 3:1)

^{1}H NMR (500 MHz, Chloroform-*d*) δ 7.86 – 7.77 (m, 2H), 7.54 (t, *J* = 7.4 Hz, 1H), 7.46 – 7.38 (m, 8H), 7.36 – 7.31 (m, 4H), 7.30 – 7.26 (m, 2H), 7.20 (t, *J* = 7.2 Hz, 1H), 7.13 (td, *J* = 7.6, 2.6 Hz, 2H), 7.05 (d, *J* = 8.1 Hz, 2H), 6.96 (d, *J* = 6.4 Hz, 2H), 2.84 – 2.72 (m, 1H), 1.30 (d, *J* = 10.1 Hz, 3H).

^{13}C NMR (126 MHz, Chloroform-*d*) δ 140.8 (dd, *J* = 6.9, 3.1 Hz), 134.9 (d, *J* = 92.5 Hz), 134.2 (d, *J* = 96.4 Hz), 131.8 (d, *J* = 9.0 Hz), 131.6 (d, *J* = 7.8 Hz), 131.4 (d, *J* = 8.1 Hz), 131.2 (d, *J* = 37.8 Hz), 130.8 (d, *J* = 8.4 Hz), 130.7, 130.4 (d, *J* = 54.6 Hz), 129.4 (d, *J* = 58.1 Hz), 128.8 (dd, *J* = 32.3, 10.0 Hz), 127.7 (dd, *J* = 35.1, 11.2 Hz), 127.4 (d, *J* = 54.4 Hz), 118.4 (d, *J* = 3.8 Hz), 9.5 (d, *J* = 36.9 Hz).

^{11}B NMR (160 MHz, Chloroform-*d*) δ -26.84.

^{31}P NMR (162 MHz, Chloroform-*d*) δ 36.42 (d, *J* = 74.8 Hz), 7.11.

HRMS (ESI): calc'd for $(M+H)^+$ $C_{32}H_{31}BBrOP_2^+$ 583.1121, found 583.1127.

HPLC analysis: DAICEL CHIRALCEL AD-H, hexane/isopropanol = 60/40, 1 mL/min, λ = 254 nm, t_R (major) = 14.894 min, t_R (minor) = 13.333 min, 94% ee.

$[\alpha]^{25}_D$: -20.8 (*c* 0.5, $CHCl_3$).

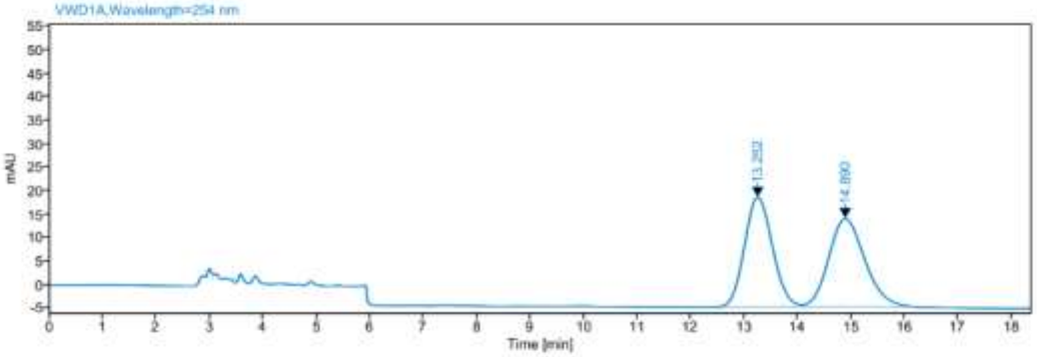

Signal: VWD1A,Wavelength=254 nm

RT [min]	Type	Width [min]	Area	Height	Area%	Name
13.252	BM m	1.65	849.15	23.33	49.11	
14.890	MM m	2.09	880.07	18.79	50.89	
		**Sum**	**1729.22**			

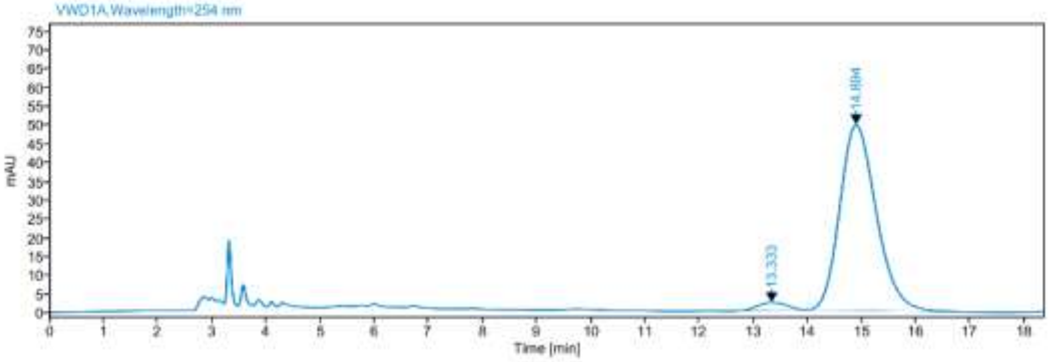

Signal: VWD1A,Wavelength=254 nm

RT [min]	Type	Width [min]	Area	Height	Area%	Name
13.333	BB	1.35	77.51	2.01	3.23	
14.894	BB	3.43	2321.87	49.43	96.77	
		**Sum**	**2399.38**			

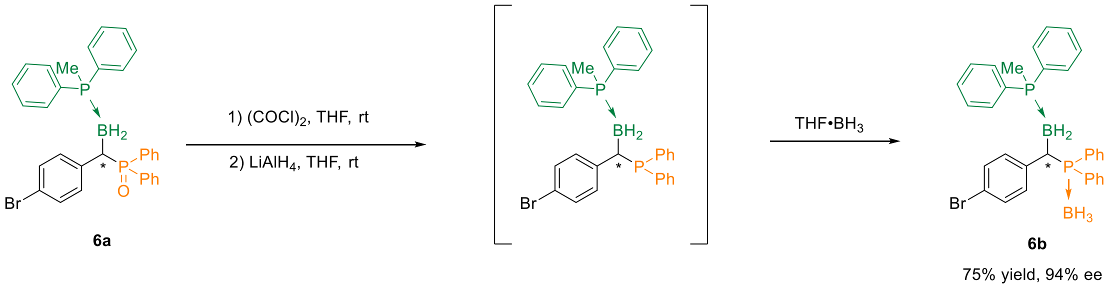

(S)-(((methyldiphenylphosphane)boryl)(4-bromophenyl)methyl)diphenylphosphane-borane (6b)

Under argon, a mixture of **6a** (58.3 mg, 0.10 mmol) in DCM (1.0 mL), oxalyl chloride (20.0 μL, 0.23 mmol, 2.3 equiv) was added dropwise at 0 °C, and then stirred to room temperature for 0.5 h. then the reaction mixture was evaporated to dryness in vacuo. To a solution of mixture in THF (1.0 mL) was added $LiAlH_4$ (0.1 mL, 0.25 mmol, 2.5 equiv, 2.5 mol/L in THF) at 0 °C, and then the

stirred mixture to room temperature for 12 h.

BH_3•THF (0.20 mL, 0.20 mmol, 2.0 equiv, 1 mol/L in THF) was then added dropwise to the obtained mixture at 0 °C and stirred at the same temperature for 3 h. Then, aqueous $NaHCO_3$ (5 mL) was slowly added and the mixture was extracted with EtOAc (3 mL×3). The organic layers were combined, washed with brine (5 mL), dried over Na_2SO_4, and concentrated under reduced pressure. The crude product was purified by flash column chromatography to afford **6b** as a colorless oil in 75% yield (43.6 mg) with 94% ee.[11]

R_f =0.10 (silica gel, EtOAc:PE = 1:10)

^{1}H NMR (400 MHz, Chloroform-*d*) δ 7.79 (t, *J* = 8.8 Hz, 2H), 7.56 (t, *J* = 7.5 Hz, 1H), 7.50 – 7.27 (m, 14H), 7.23 (d, *J* = 7.1 Hz, 1H), 7.16 (d, *J* = 7.5 Hz, 2H), 7.08 (d, *J* = 8.0 Hz, 2H), 6.89 (d, *J* = 8.0 Hz, 2H), 2.85 – 2.71 (m, 1H), 1.26 (d, *J* = 9.7 Hz, 3H).

^{13}C NMR (101 MHz, Chloroform-*d*) δ 140.9, 133.6 (d, *J* = 8.1 Hz), 132.3 (d, *J* = 5.7 Hz), 132.2 (d, *J* = 8.1 Hz), 131.8 (dd, *J* = 12.3, 9.0 Hz), 131.3 (dd, *J* = 26.9, 2.5 Hz), 130.5 (d, *J* = 2.9 Hz), 129.9, 129.7 (d, *J* = 2.2 Hz), 129.4 (d, *J* = 58.1 Hz), 128.9 (dd, *J* = 20.6, 10.0 Hz), 127.8 (dd, *J* = 27.3, 9.4 Hz), 127.4, 118.9 (d, *J* = 4.1 Hz), 9.5 (d, *J* = 36.3 Hz).

^{11}B NMR (160 MHz, Chloroform-*d*) δ -26.72, -40.49.

^{31}P NMR (202 MHz, Chloroform-*d*) δ 26.75, 7.38.

HRMS (ESI): calc'd for $(M-H)^-$ $C_{32}H_{32}B_2BrP_2^-$ 579.1354, found 579.1351.

HPLC analysis: DAICEL CHIRALCEL AD-H, hexane/isopropanol = 90/10, 1 mL/min, λ = 254 nm, t_R (major) = 13.199 min, t_R (minor) = 9.667 min, 94% ee.

$[\alpha]^{25}_D$: -46.4 (*c* 0.5, $CHCl_3$).

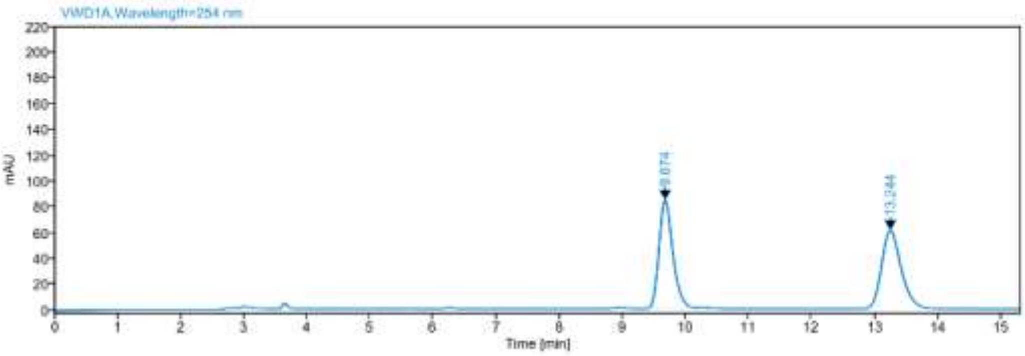

Signal: VWD1A,Wavelength=254 nm

RT [min]	Type	Width [min]	Area	Height	Area%	Name
9.674	BV	0.86	1296.34	84.14	49.52	
13.244	BB	2.31	1321.61	60.71	50.48	
		**Sum**	**2617.95**			

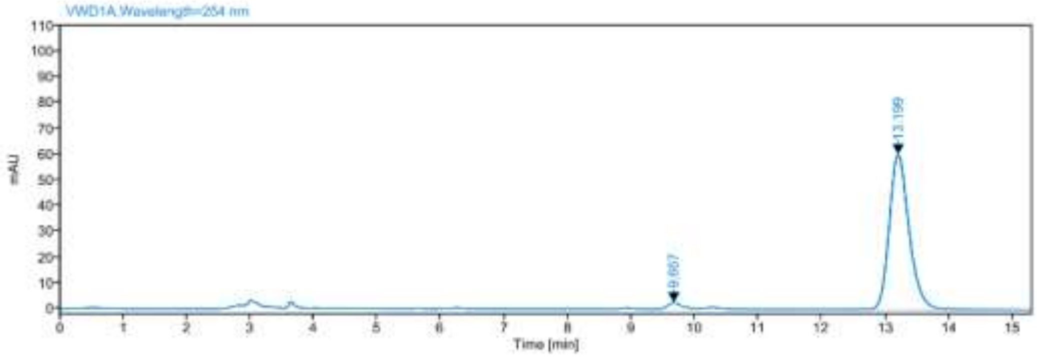

Signal: VWD1A,Wavelength=254 nm

RT [min]	Type	Width [min]	Area	Height	Area%	Name
9.667	BM m	0.68	40.37	2.49	3.02	
13.199	BB	1.94	1295.13	60.12	96.98	
		**Sum**	**1335.50**			

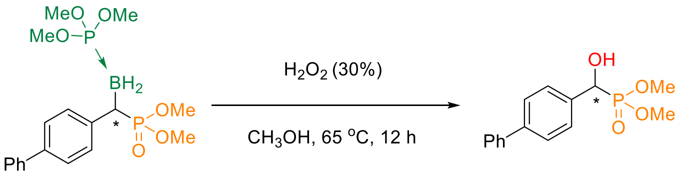

3li **6c**

51% yield, 93% ee

(R)-dimethyl([1,1'-biphenyl]-4-yl(hydroxy)methyl)phosphonate (6c)

Under agron, to a solution of **3li** (41.2 mg, 0.10 mmol, 1.0 equiv) in 0.50 mL MeOH, 0.25 mL 30% H_2O_2 was added one portion. The resulting mixture was heated to 65 °C for 12 h. After cooling to room temperature. The aqueous layer was extracted with EtOAc (3 x 5 mL). The combined organic extract was washed with brine, then dried over anhydrous Na_2SO_4, filtered, and concentrated

to give crude product. The crude product was purified by silica gel column chromatography to afford **6c** as a white solid (14.8 mg, 51% yield, 93% ee).[12]

mp = 120.1 – 121.8 °C

$\mathbf{R}_f$ = 0.22 (silica gel, EtOAc:PE = 1:1)

^{1}H NMR (400 MHz, Chloroform-*d*) δ 7.66 – 7.53 (m, 6H), 7.44 (t, *J* = 7.5 Hz, 2H), 7.35 (t, *J* = 7.3 Hz, 1H), 5.11 (d, *J* = 11.0 Hz, 1H), 3.79 – 3.60 (m, 7H).

^{13}C NMR (101 MHz, Chloroform-*d*) δ 141.2, 140.5, 135.1 (d, *J* = 2.6 Hz), 128.8, 127.5, 127.4, 127.2 (d, *J* = 2.5 Hz), 127.1, 70.5 (d, *J* = 159.4 Hz), 53.8 (dd, *J* = 12.8, 7.3 Hz).

^{31}P NMR (162 MHz, Chloroform-*d*) δ 23.41.

HRMS (ESI): calc'd for $(M+H)^+$ $C_{15}H_{18}O_4P^+$ 293.0937, found 293.0933.

HPLC analysis: DAICEL CHIRALCEL AD-H, hexane/isopropanol = 85/15, 1 mL/min, λ = 254 nm, t_R (major) = 8.237 min, t_R (minor) = 7.704 min, 93% ee.

$[\alpha]^{25}_D$: +16.8 (*c* 0.5, $CHCl_3$).

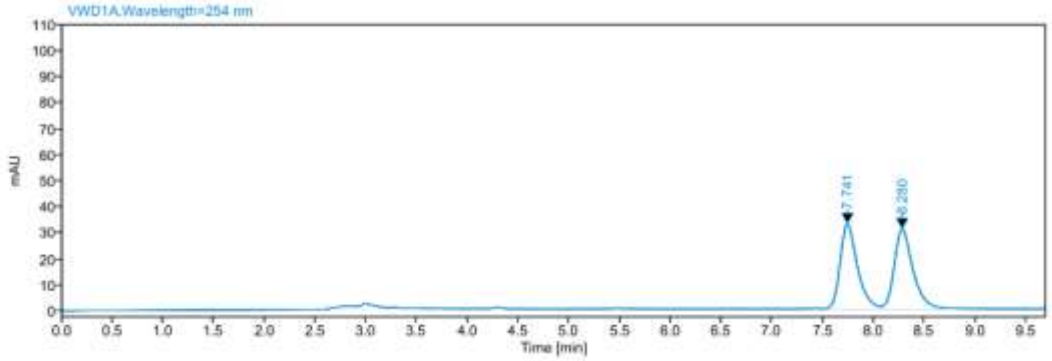

Signal: VWD1A,Wavelength=254 nm

RT [min]	Type	Width [min]	Area	Height	Area%	Name
7.741	BV	0.85	385.33	32.70	49.32	
8.280	VB	1.52	395.90	30.68	50.68	
		Sum	781.23			

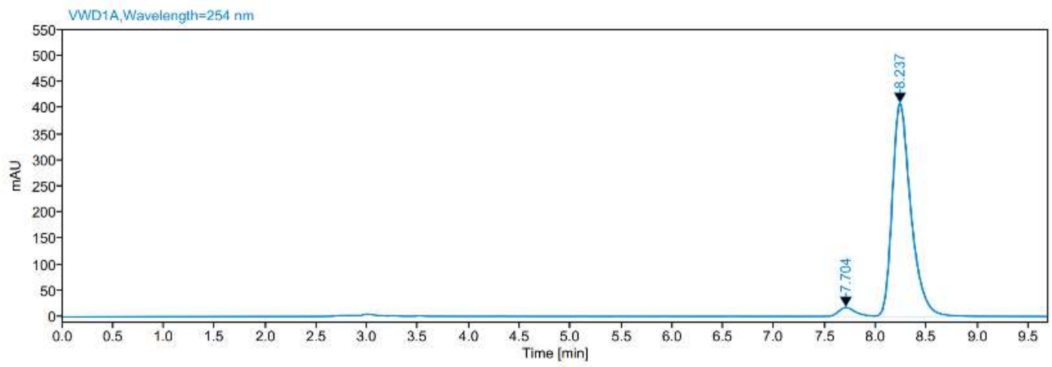

Signal: VWD1A,Wavelength=254 nm

RT [min]	Type	Width [min]	Area	Height	Area%	Name
7.704	MM m	0.56	199.12	17.19	3.68	
8.237	MB m	2.01	5216.70	408.34	96.32	
		**Sum**	**5415.82**			

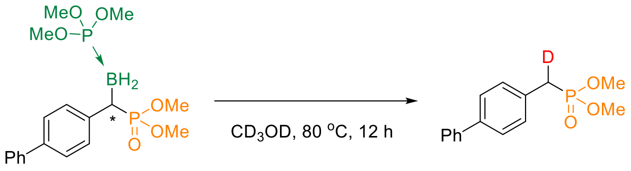

dimethyl ([1,1'-biphenyl]-4-ylmethyl-d)phosphonate(6d)

Under agron, to a solution of **3li** (41.2 mg, 0.1 mmol, 1.0 equiv) in 1 mL CD_3OD, the resulting mixture was heated to 80 ºC for 12 h. After cooling to room temperature, concentrated to give crude product. The crude product was purified by silica gel column chromatography to afford **6d** as a white solid (23.6 mg, 85% yield, 98% D).

mp = 65.6 – 66.9 °C

R_f = 0.30 (silica gel, EtOAc:PE = 1:1)

^{1}H NMR (400 MHz, Chloroform-*d*) δ 7.61 – 7.53 (m, 4H), 7.43 (t, *J* = 7.6 Hz, 2H), 7.39 – 7.31 (m, 3H), 3.70 (d, *J* = 10.8 Hz, 6H), 3.20 (d, *J* = 21.7 Hz, 1H).

^{13}C NMR (101 MHz, Chloroform-*d*) δ 140.6 (d, *J* = 1.6 Hz), 139.9 (d, *J* = 3.9 Hz), 130.2 (d, *J* = 9.4 Hz), 130.1 (d, *J* = 6.6 Hz), 128.7, 127.3 (d, *J* = 3.1 Hz), 127.3, 127.0, 52.9 (d, *J* = 6.9 Hz).

^{31}P NMR (162 MHz, Chloroform-*d*) δ 28.86.

HRMS (ESI): calc'd for $(M+H)^+$ $C_{15}H_{17}DO_3P^+$ 278.1051, found 278.1051.

1.6 Mechanism Study of the B-H bond insertion reaction

(A) Deuterium labeling experiment

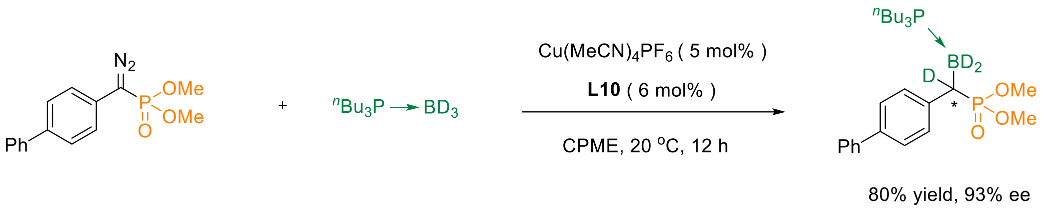

In air, a 25 mL schlenk tube was charged with $Cu(MeCN)_4PF_6$ (5 mol%), **L10** (6 mol%). The tube was evacuated and filled with argon for three cycles. Then, 2 mL of CPME , dimethyl ([1,1'-biphenyl]-4-yl(diazo)methyl)phosphonate (0.20 mmol, 1.0 equiv) and tributylphosphane borane-***d₃*** (0.4 mmol, 2.0 equiv) was added under argon. The reaction was allowed to stir at 20 °C for 12 hours. Upon completion, proper amount of silica gel was added to the reaction mixture. After removal of the solvent, the crude reaction mixture was purified on silica gel (petroleum ether and ethyl acetate) to afford the desired products as a colorless oil in 80% yield (78.6 mg) with 93% ee.

(B) Procedure for One-Pot competition KIE experiment

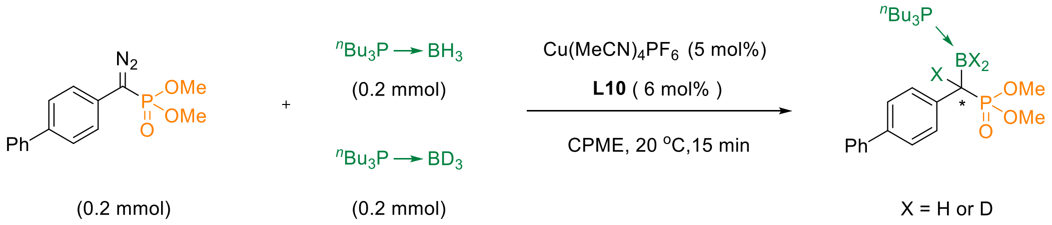

In air, a 25 mL schlenk tube was charged with $Cu(MeCN)_4PF_6$ (5 mol%), **L10** (6 mol%). The tube was evacuated and filled with argon for three cycles. Then, 2 mL of CPME ,dimethyl ([1,1'-biphenyl]-4-yl(diazo)methyl)phosphonate (0.20 mmol, 1.0 equiv), tributylphosphane borane (0.20 mmol, 1.0 equiv) and tributylphosphane borane-***d_3*** (0.20 mmol, 1.0 equiv) was added under argon. The reaction was allowed to stir at 20 °C for 15 minutes. Upon completion, proper amount of silica gel was added to the reaction mixture. After removal of the solvent, the crude reaction mixture was purified on silica gel (petroleum ether and ethyl acetate) to afford **3lh** and **3lh-*d_3*** as the mixture of colorless oil (50.1 mg). KIE was determined by ^1H NMR.

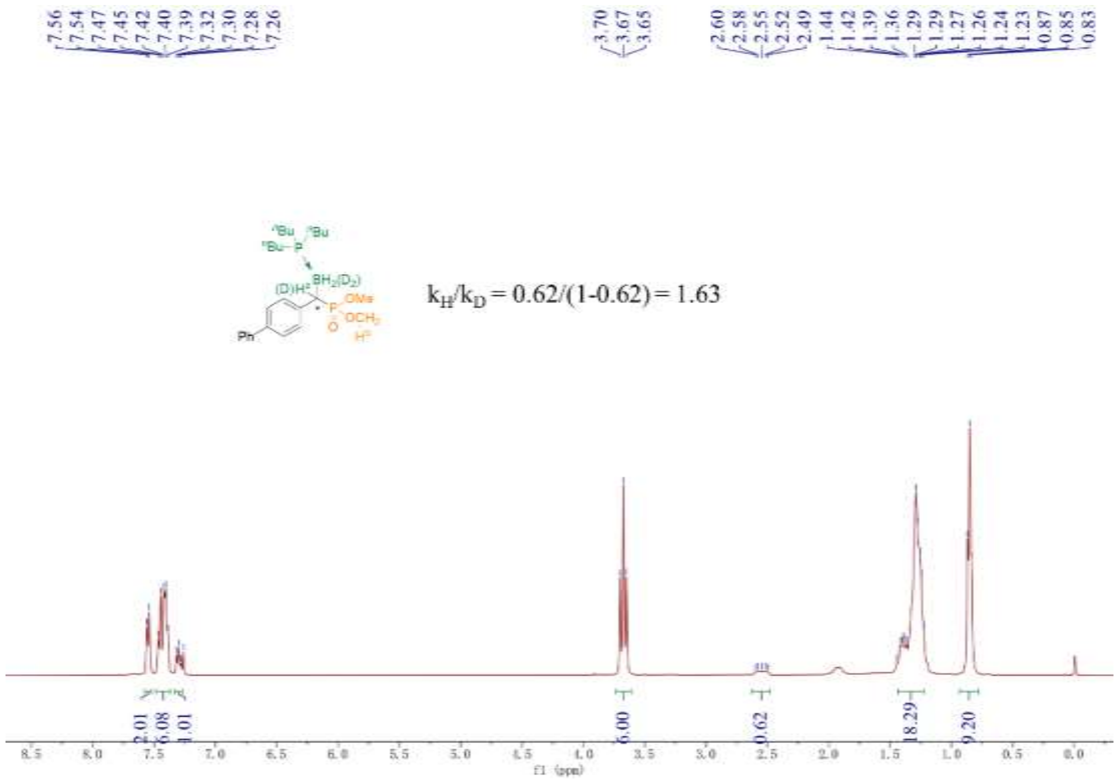

1.7 Crystal structure of compound 3aa

For **3aa**: the data was collected by using molybdenum (Mo) irradiation source at room temperature. The crystal sample of **3aa** was recrystallized from a mixture of DCM and PE.

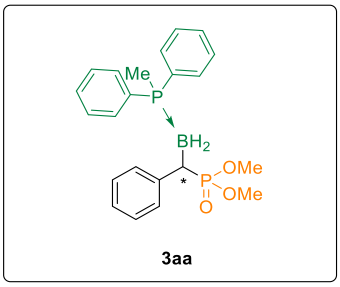

CCDC 2312601

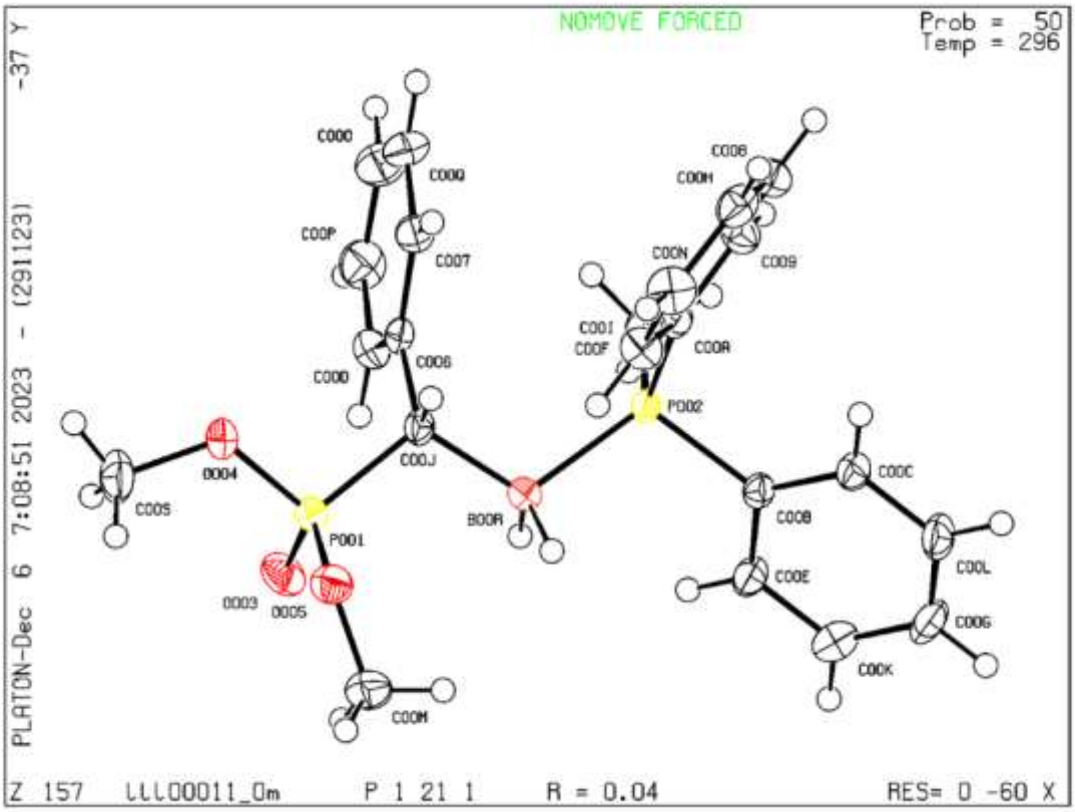

Table 1 Crystal data and structure refinement for LLL00011_0m.

Identification code	LLL00011_0m
Empirical formula	$C_{22}H_{27}BO_3P_2$
Formula weight	412.18
Temperature/K	296.15
Crystal system	monoclinic
Space group	$P2_1$
a/Å	10.778(2)
b/Å	9.8031(17)
c/Å	11.594(3)
α/°	90
β/°	117.183(7)
γ/°	90
Volume/Å^3	1089.8(4)
Z	2
ρ_{calc}g/cm^3	1.256
μ/mm^{-1}	0.219
F(000)	436.0

Crystal size/mm^3	0.15 × 0.12 × 0.014
Radiation	MoKα (λ = 0.71073)
2Θ range for data collection/°	3.95 to 63.78
Index ranges	$-13 \leq h \leq 15$, $-14 \leq k \leq 12$, $-15 \leq l \leq 16$
Reflections collected	20561
Independent reflections	5636 [R_{int} = 0.0487, R_{sigma} = 0.0560]
Data/restraints/parameters	5636/1/264
Goodness-of-fit on F^2	1.072
Final R indexes [I>=2σ (I)]	R_1 = 0.0358, wR_2 = 0.0809
Final R indexes [all data]	R_1 = 0.0572, wR_2 = 0.1021
Largest diff. peak/hole / e $Å^{-3}$	0.34/-0.41
Flack parameter	-0.01(3)

2. References

(1) Ye, F.; Wang, C.; Zhang, Y.; Wang, J. *Angew. Chem. Int. Ed.* **2014**, *53*, 11625-11628.

(2) Zhou, Y.; Ye, F.; Zhou, Q.; Zhang, Y.; Wang, J. *Org. Lett.* **2016**, *18*, 2024-2027.

(3) Zhou, Y.; Zhang, Y.; Wang, J. *Org. Biomol. Chem.* **2016**, *14*, 10444-10453.

(4) Sun, S.; Wei, Y.; Xu, J. *Org. Lett.* **2022**, *24*, 6024-6030.

(5) Busacca, C. A.; Raju, R.; Grinberg, N.; Haddad, N.; James-Jones, P.; Lee, H.; Lorenz, J. C.; Saha, A.; Senanayake, C. H. *J. Org. Chem.* **2008**, 73, 1524-1531.

(6) Woźnicki, P.; Korzeniowska, E.; Stankevič, M. *J. Org. Chem.* **2017**, *82*, 10271- 10296.

(7) Wang, C.; Ge, Q.; Xu, C.; Xing, Z.; Xiong, J.; Zheng, Y.; Duan, W. L. *Org. Lett.* **2023**, *25*, 1583-1588.

(8) Lloyd-Jones, G. C.; Taylor, N. P. *Chem. - Eur. J.* **2015**, *21*, 5423-5428.

(9) Paul, S.; Roy, S.; Monfregola, L.; Shang, S.; Shoemaker, R.; Caruthers, M. H. *J. Am. Chem. Soc.* **2015**, *137*, 3253-3264.

(10) Lei, Z.; Zhang, W.; Wu, J. *ACS Catal.* **2023**, *13*, 16105-16113.

(11) Wei, X.-H.; Bai, C.-Y.; Wang, A.-J.; Feng, Q.-L.; Zhao, L.-B.; Zhang, P.; Li, Z.-H.; Su, Q.; Wang, Y.-B. *Org. Lett.* **2021**, *23*, 7100-7105.

(12) Pang, Y.; He, Q.; Li, Z. Q.; Yang, J. M.; Yu, J. H.; Zhu, S. F.; Zhou, Q. L. *J. Am. Chem. Soc.* **2018**, *140*, 10663-10668.

3. NMR spectra

Diethyl (diazo(phenyl)methyl)phosphonate (1a)

^{1}H NMR (400 MHz, Chloroform-*d*)

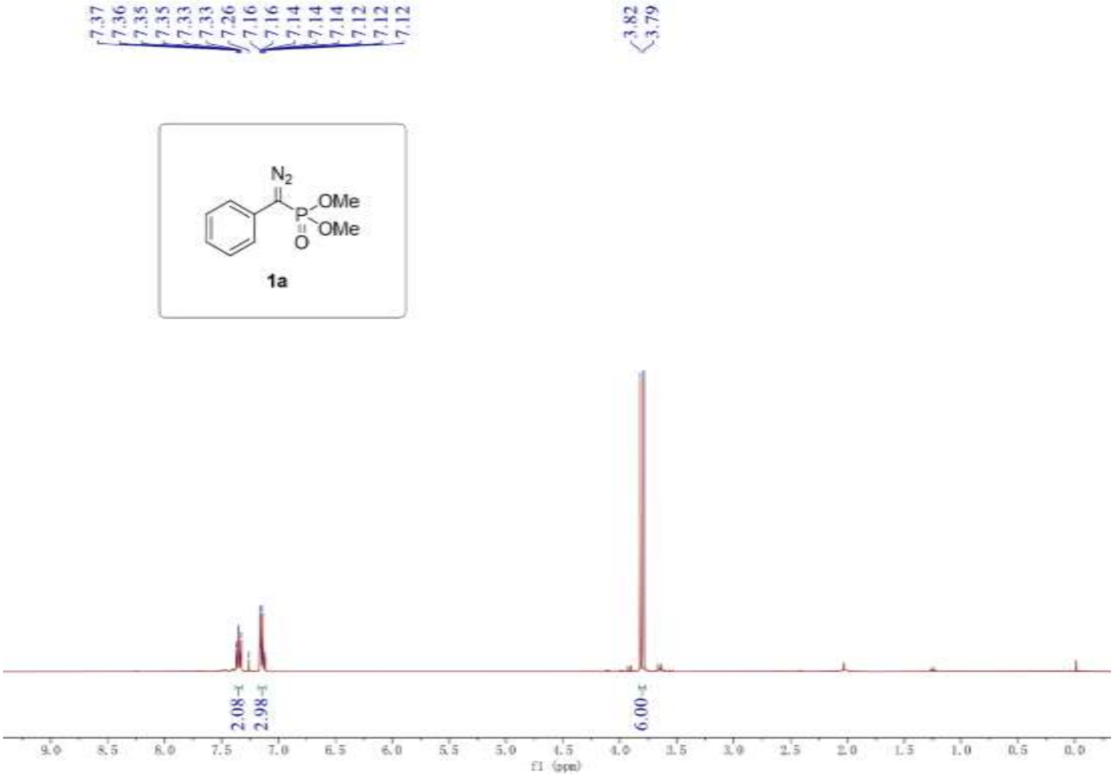

Dimethyl ((4-chlorophenyl)(diazo)methyl)phosphonate (1b)

^{1}H NMR (500 MHz, Chloroform-*d*)

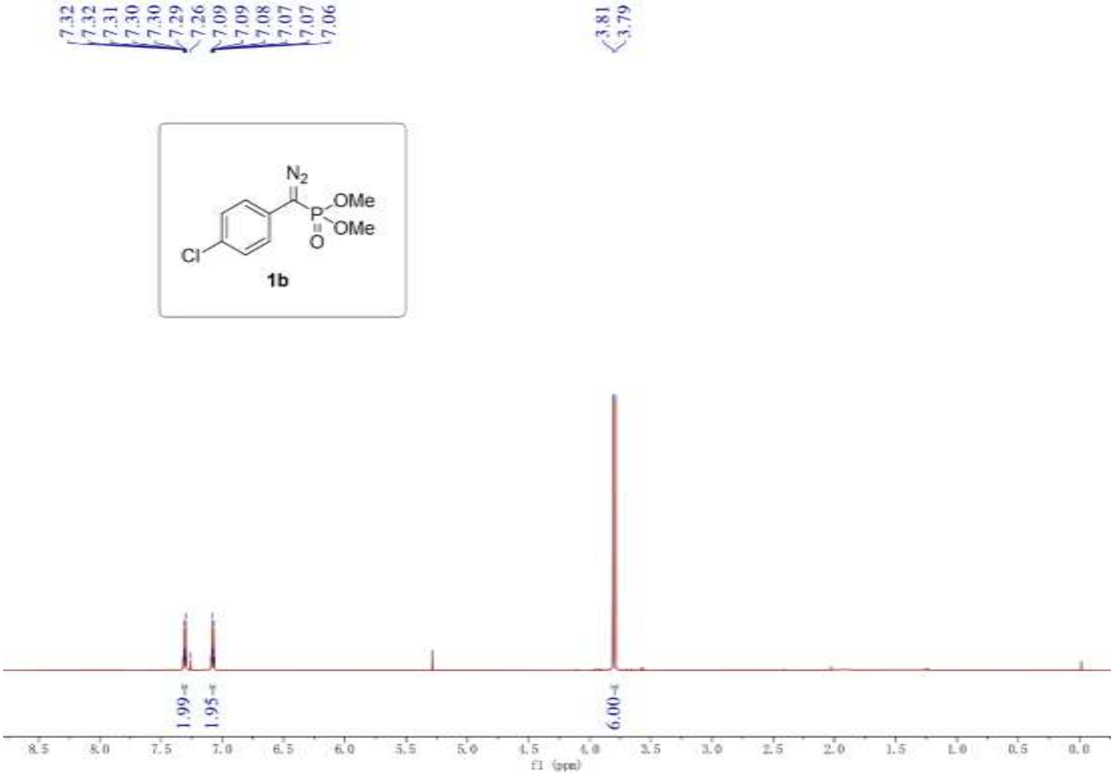

Dimethyl (diazo(4-(hydroxymethyl)phenyl)methyl)phosphonate (1c)

^{1}H NMR (500 MHz, Chloroform-*d*)

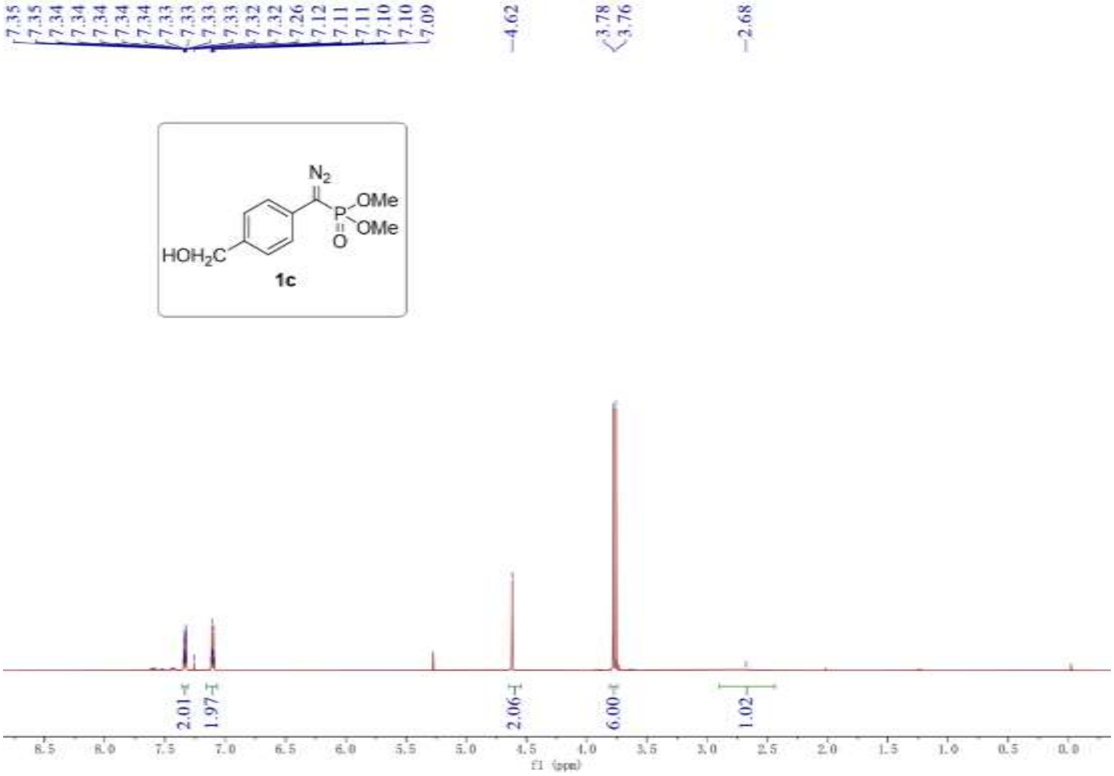

Dimethyl (diazo(4-methoxyphenyl)methyl)phosphonate (1d)

^{1}H NMR (500 MHz, Chloroform-*d*)

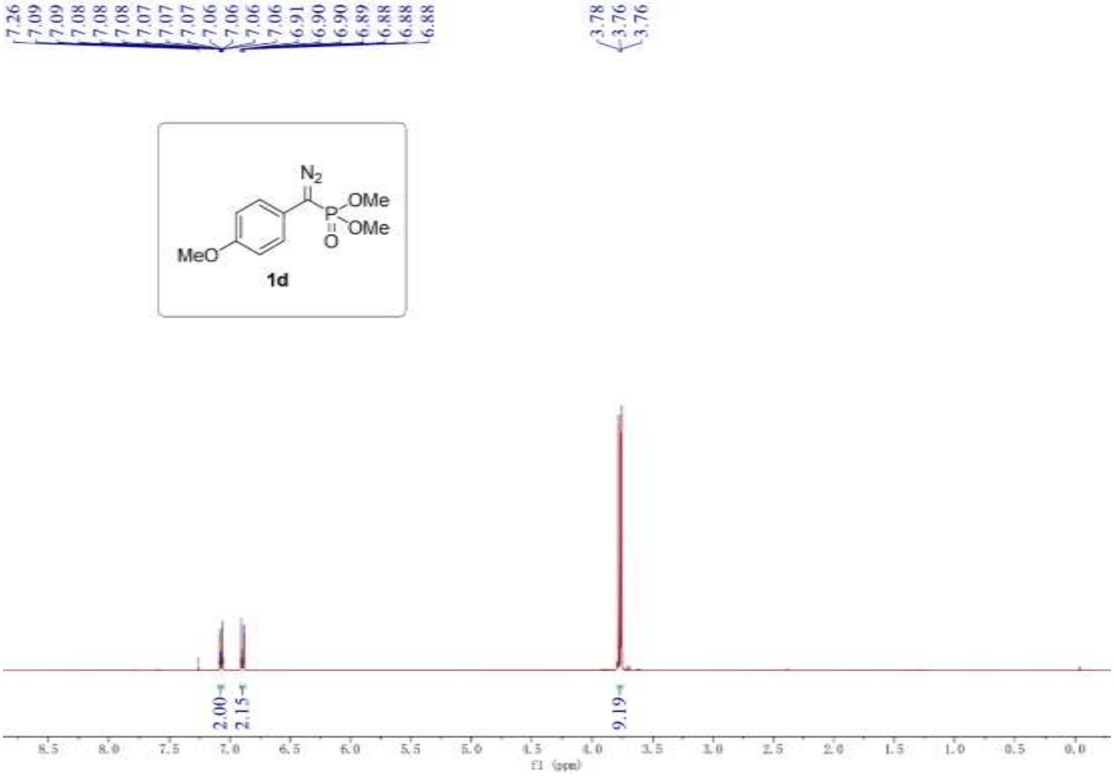

Dimethyl (diazo(p-tolyl)methyl)phosphonate (1e)

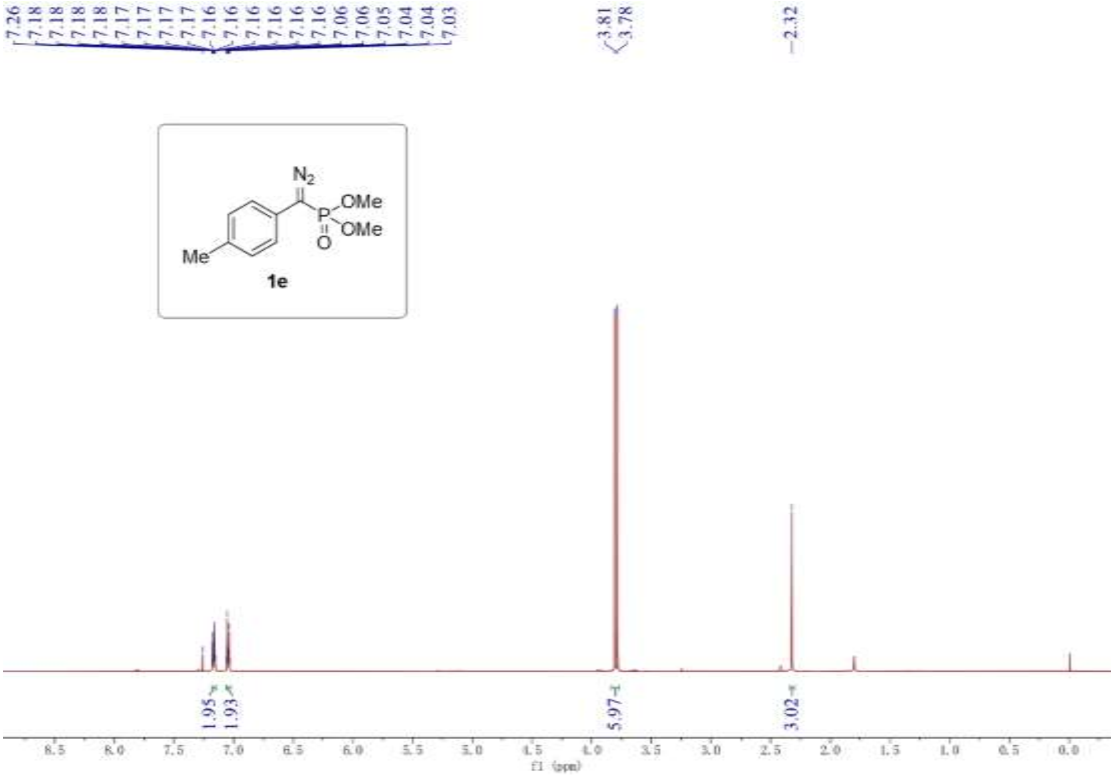

Dimethyl (diazo(4-(methylthio)phenyl)methyl)phosphonate (1f)

^{1}H NMR (500 MHz, Chloroform-*d*)

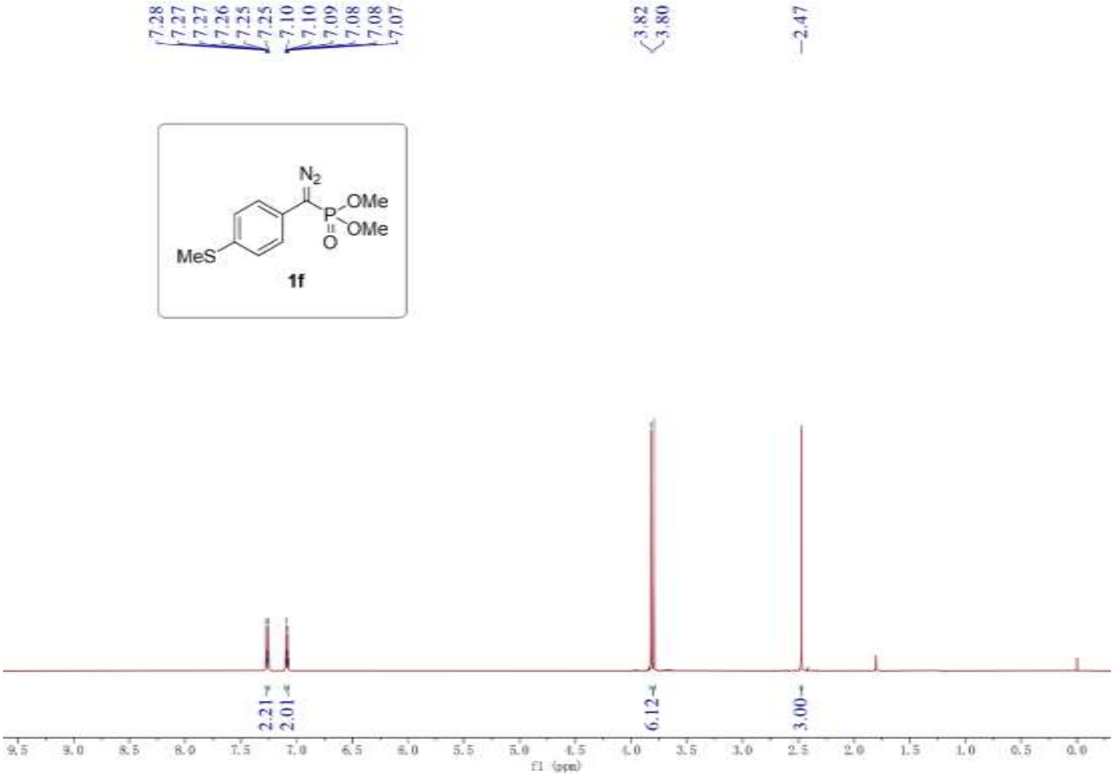

^{13}C NMR (126 MHz, Chloroform-*d*)

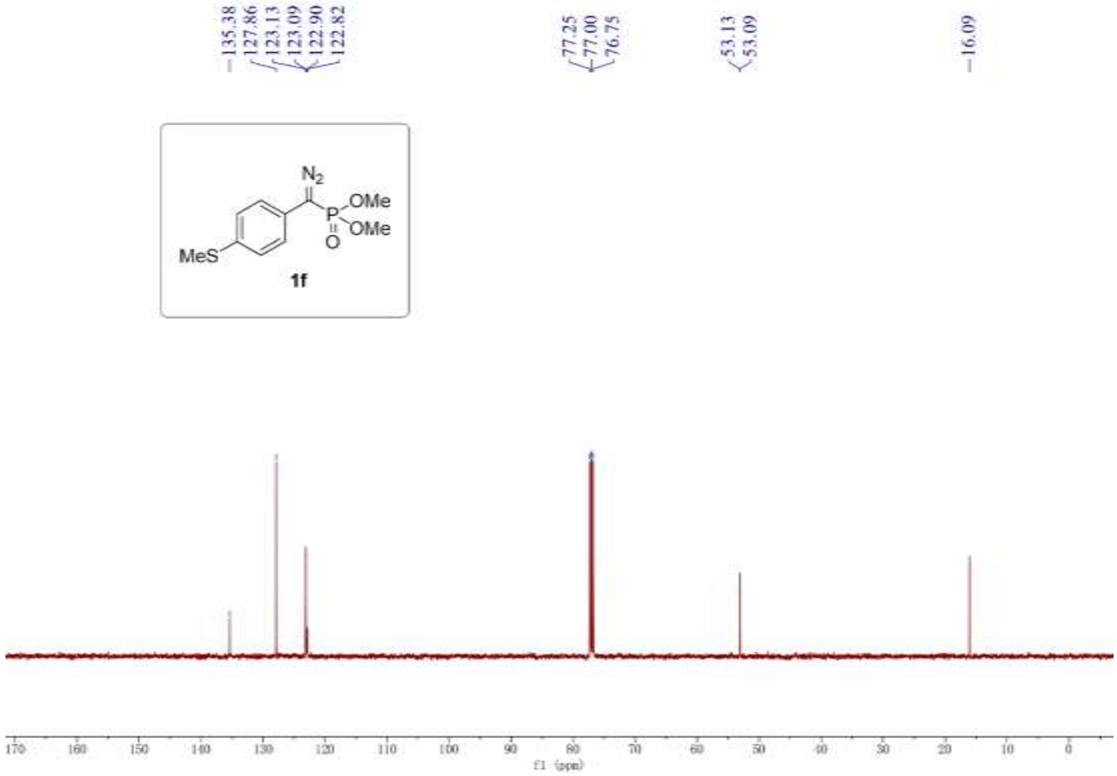

^{31}P NMR (202 MHz, Chloroform-*d*)

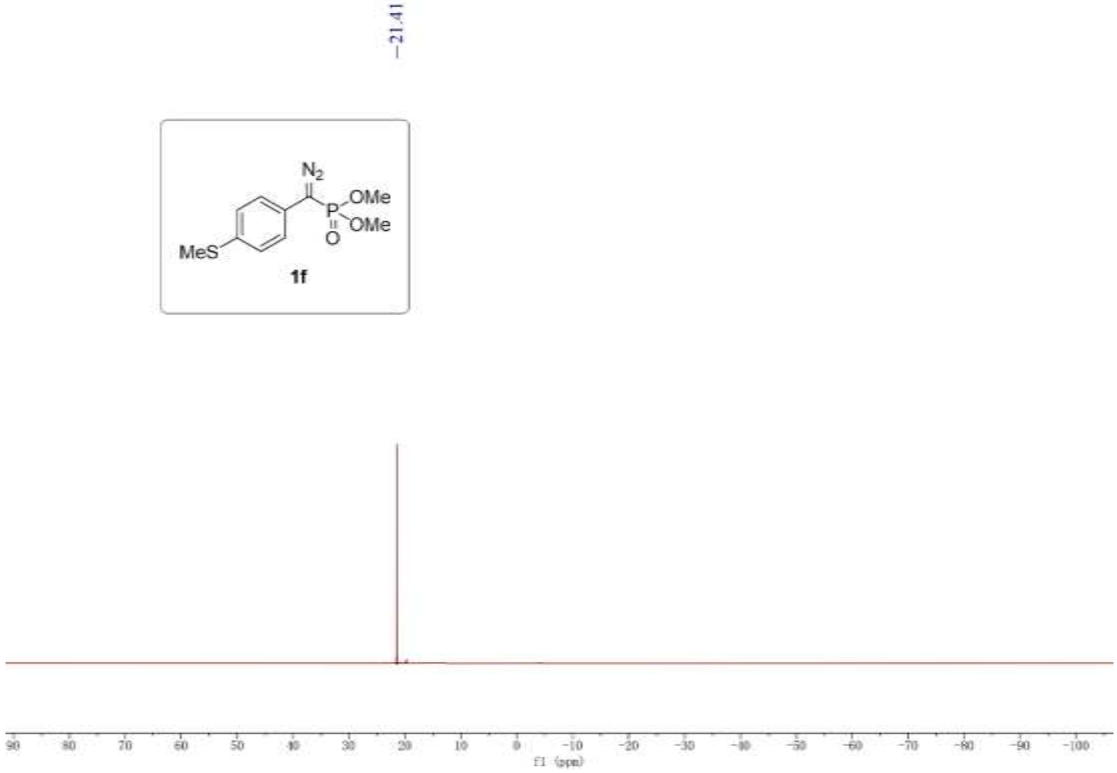

Dimethyl (diazo(4-nitrophenyl)methyl)phosphonate (1g)

^{1}H NMR (500 MHz, Chloroform-*d*)

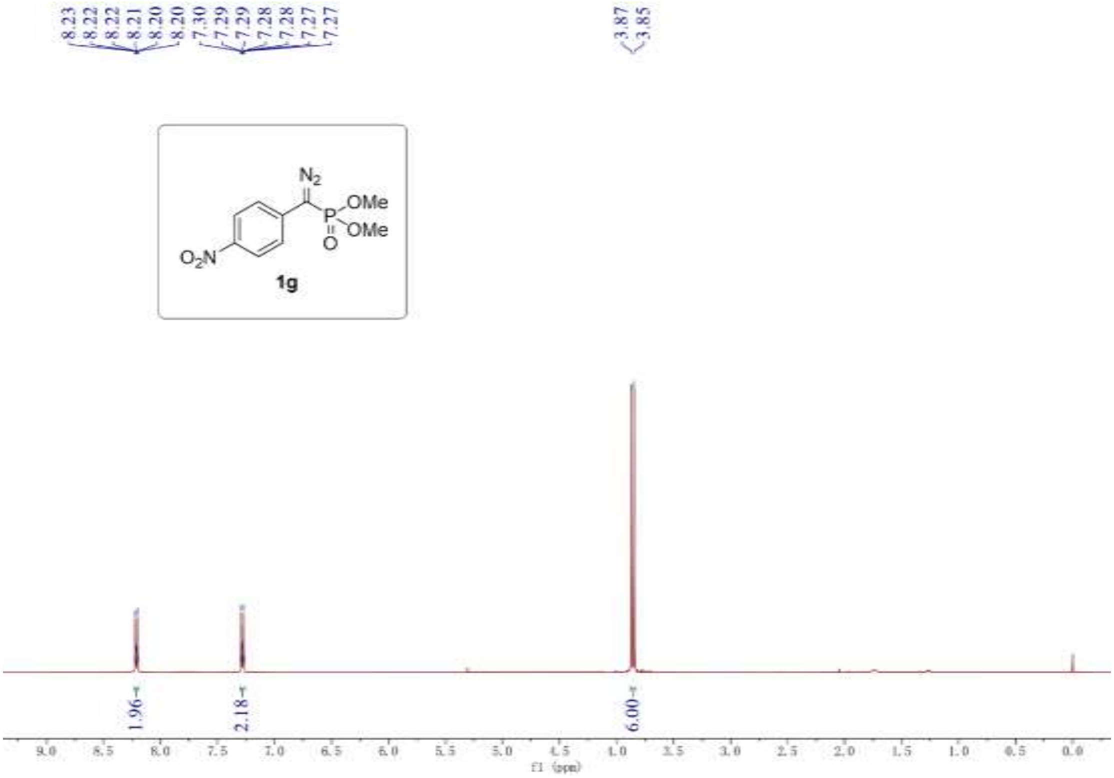

Methyl-4-(diazo(dimethoxyphosphoryl)methyl)benzoate (1h)

^{1}H NMR (500 MHz, Chloroform-*d*)

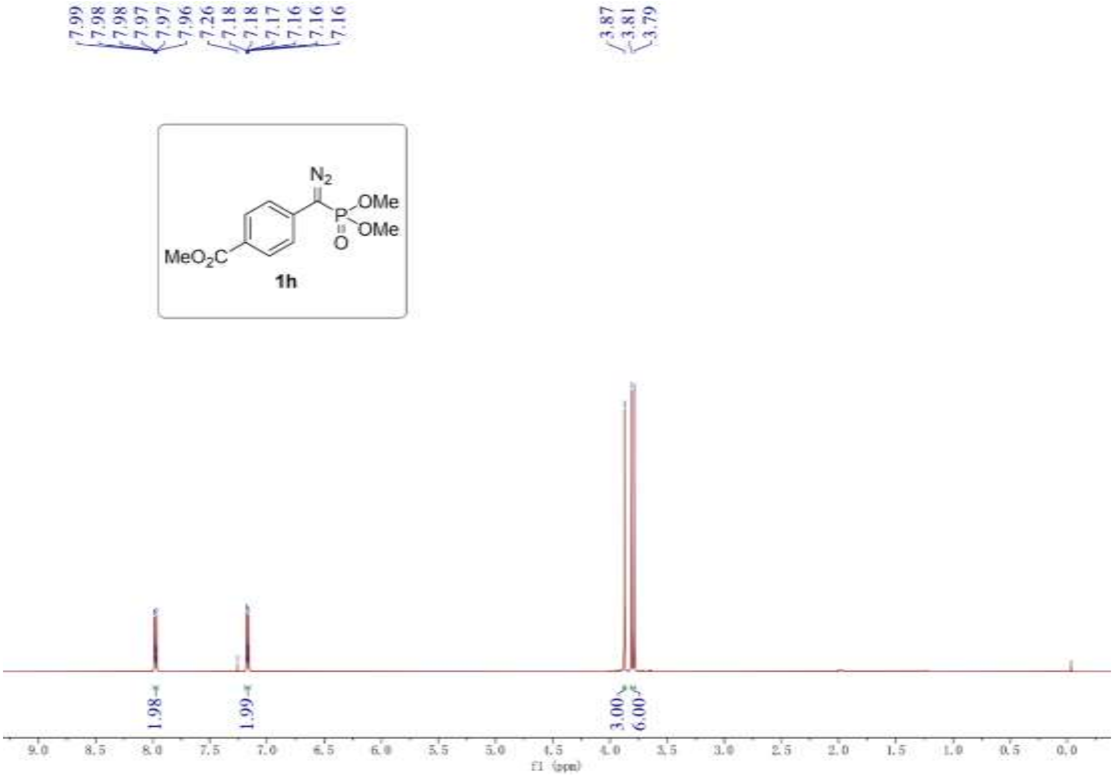

Dimethyl (diazo(4-fluorophenyl)methyl)phosphonate (1i)

^{1}H NMR (500 MHz, Chloroform-*d*)

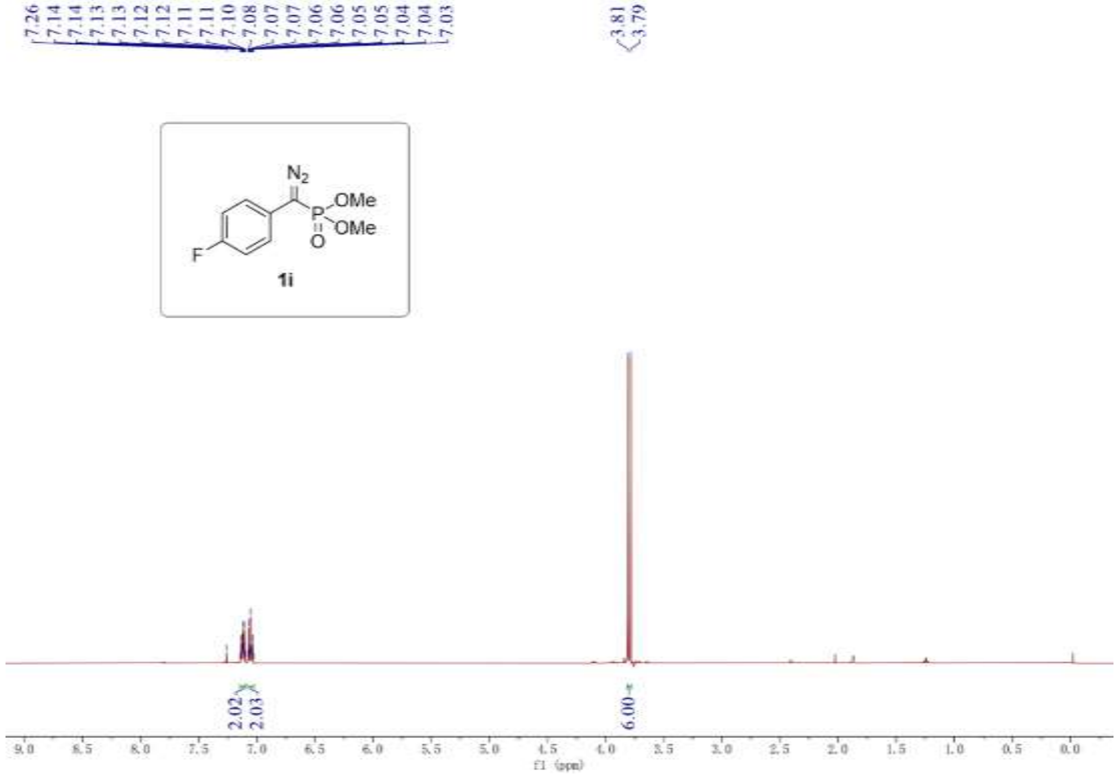

Dimethyl ((4-bromophenyl)(diazo)methyl)phosphonate (1j)

^{1}H NMR (500 MHz, Chloroform-*d*)

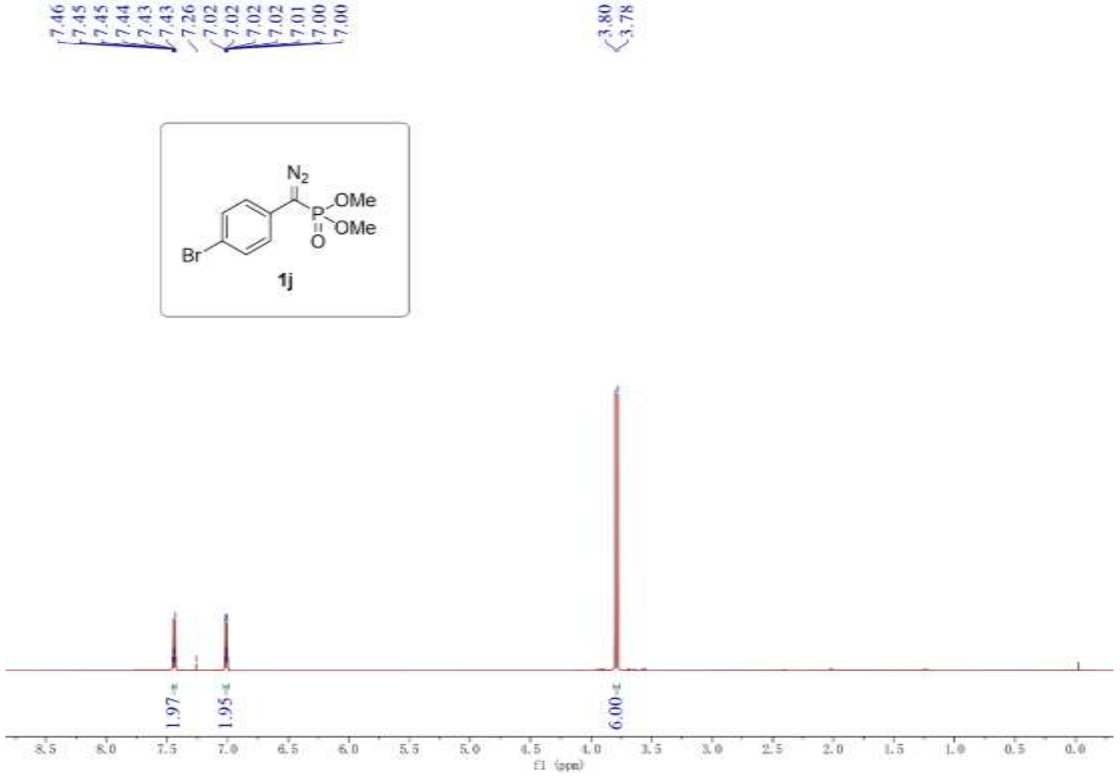

Dimethyl (diazo(4-(trifluoromethyl)phenyl)methyl)phosphonate (1k)

^{1}H NMR (400 MHz, Chloroform-*d*)

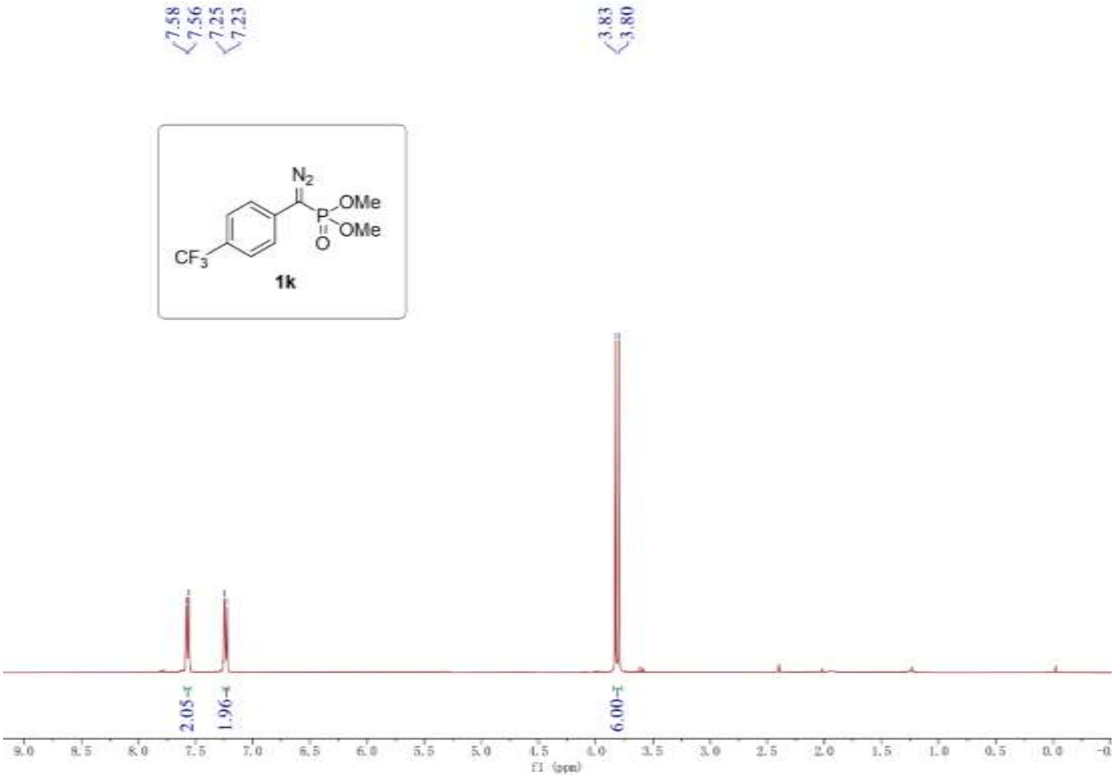

^{13}C NMR (101 MHz, Chloroform-*d*)

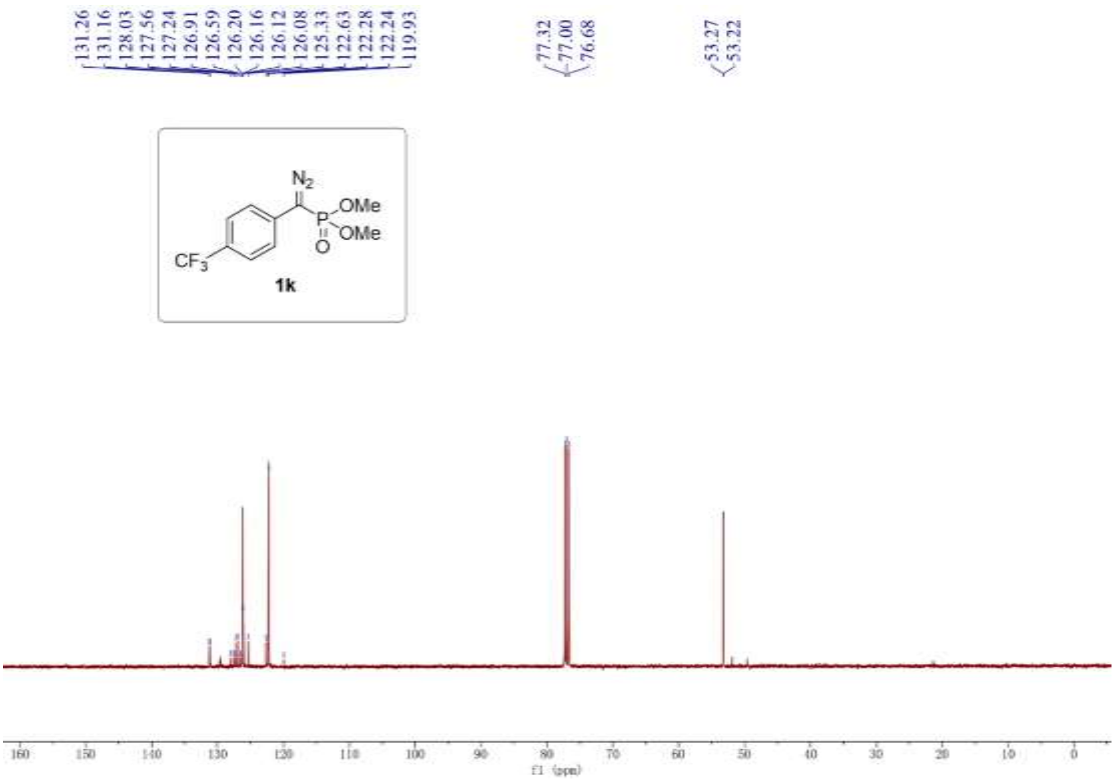

^{31}P NMR (162 MHz, Chloroform-*d*)

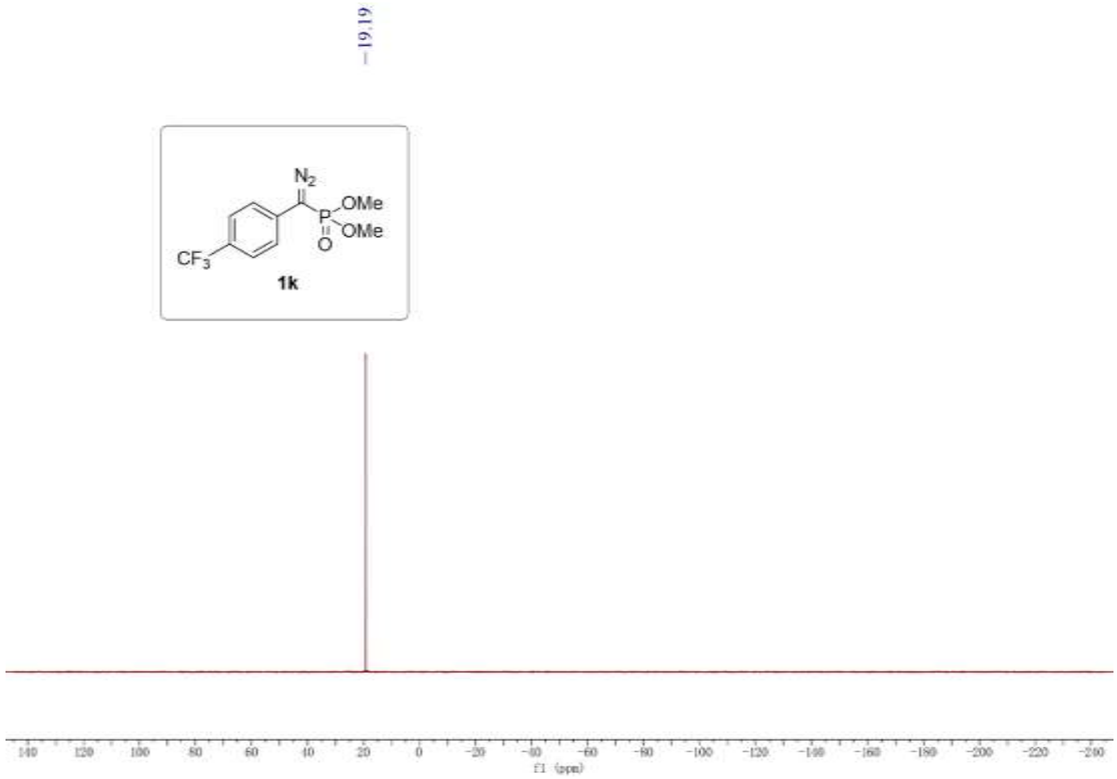

^{19}F NMR (376 MHz, Chloroform-*d*)

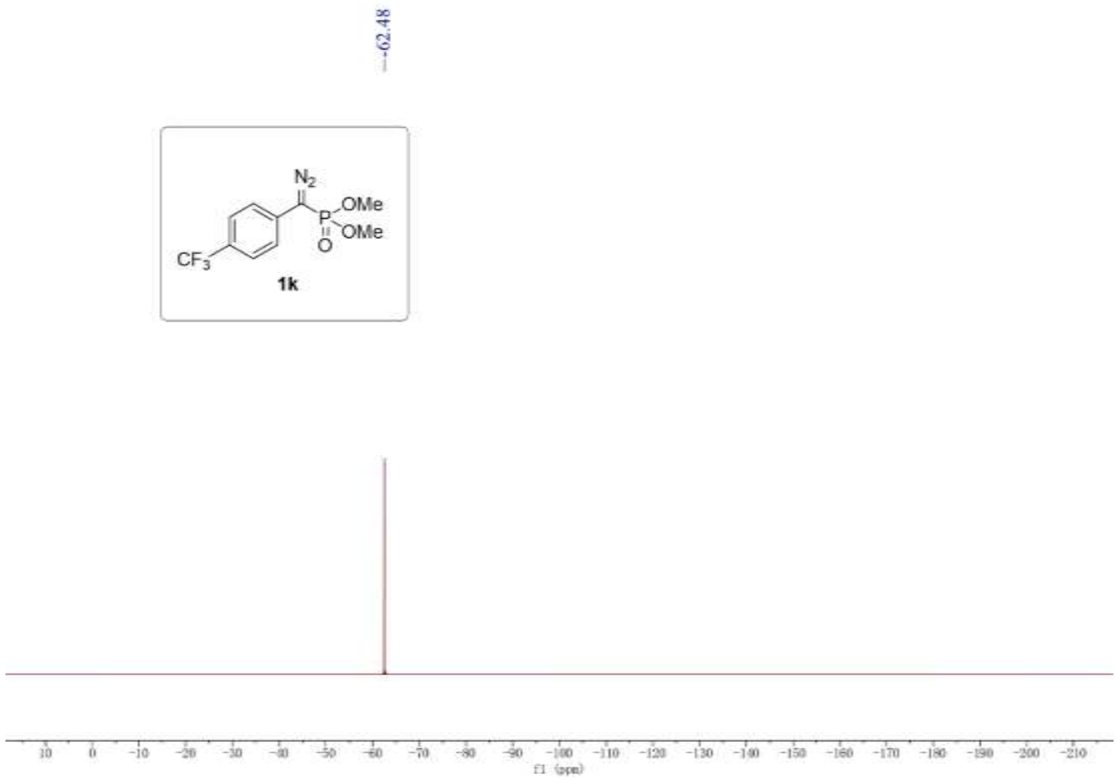

Dimethyl ([1,1'-biphenyl]-4-yl(diazo)methyl)phosphonate (1l)

^{1}H NMR (500 MHz, Chloroform-*d*)

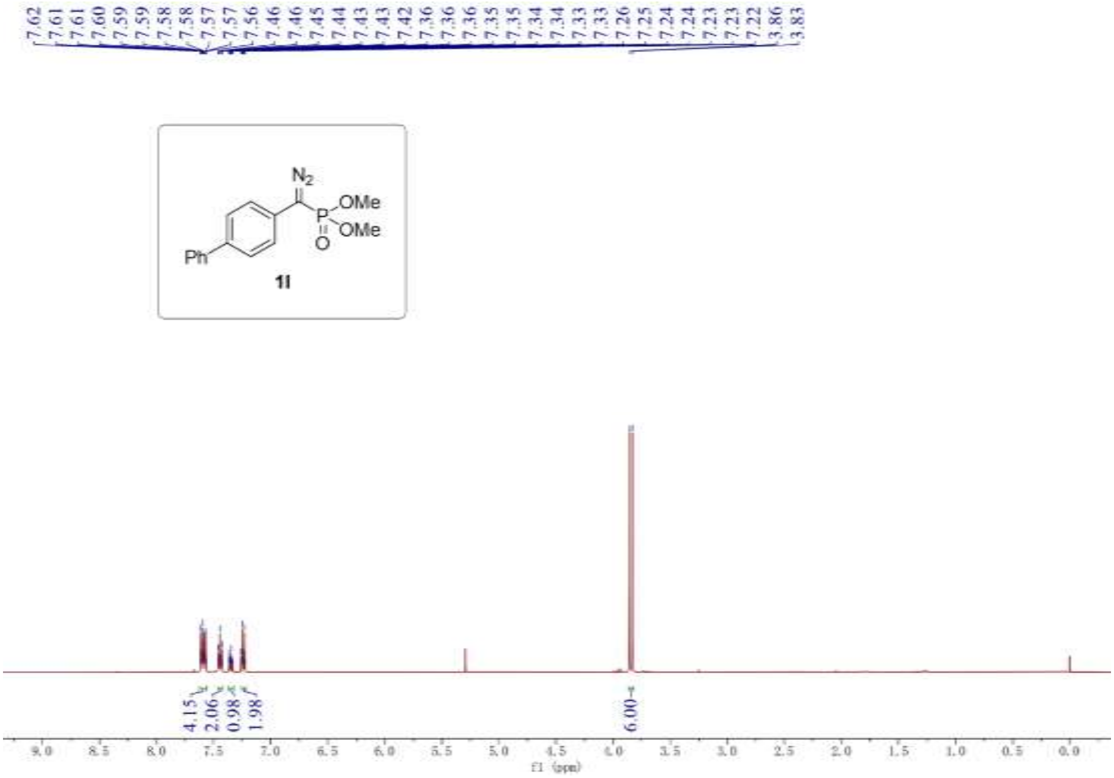

Dimethyl ((4-(benzyloxy)phenyl)(diazo)methyl)phosphonate (1m)

^{1}H NMR (400 MHz, Chloroform-*d*)

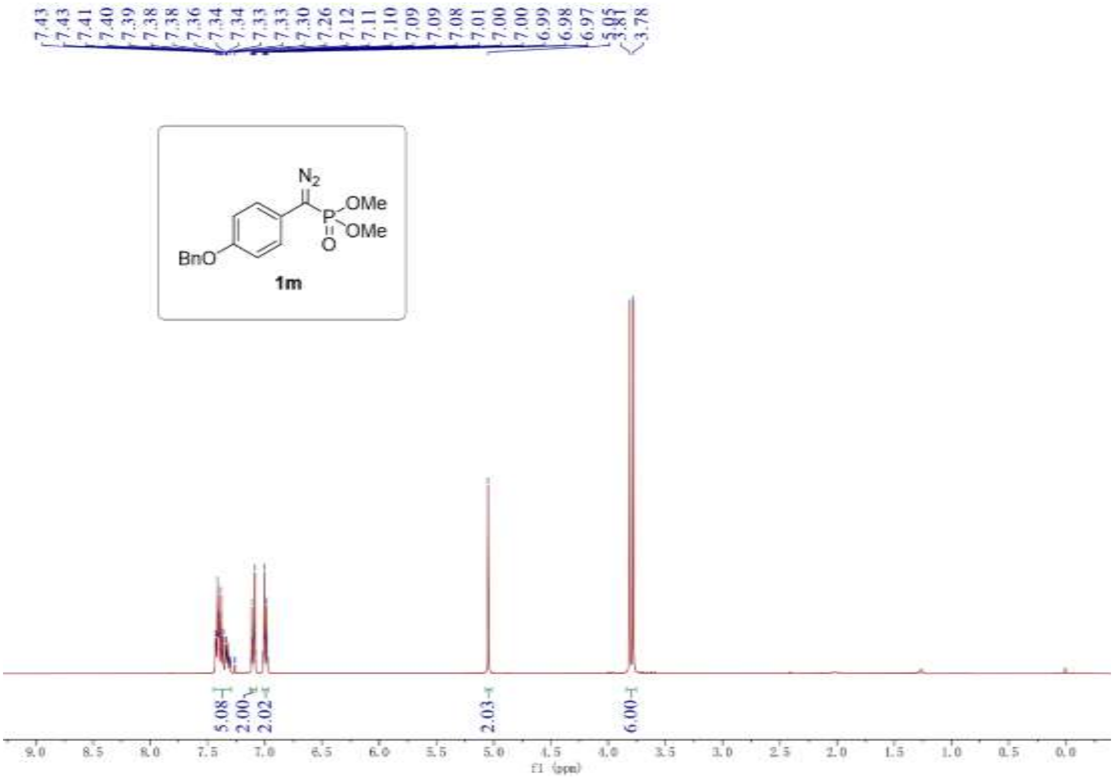

^{13}C NMR (101 MHz, Chloroform-*d*)

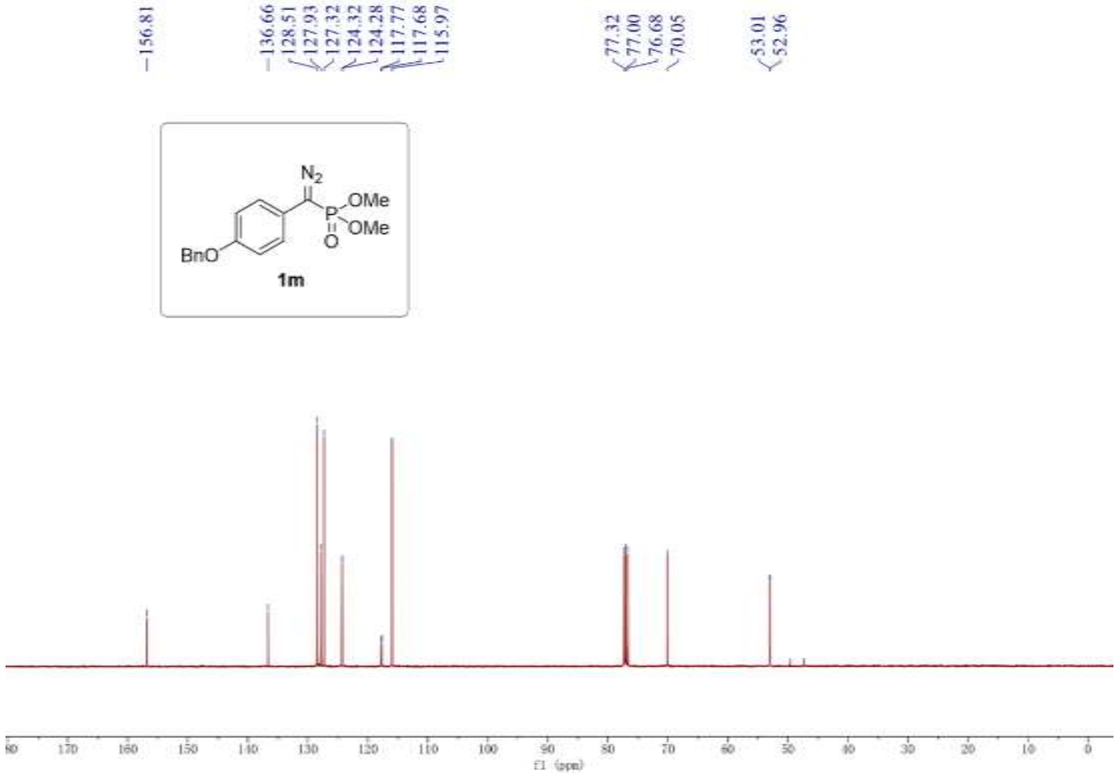

^{31}P NMR (162 MHz, Chloroform-*d*)

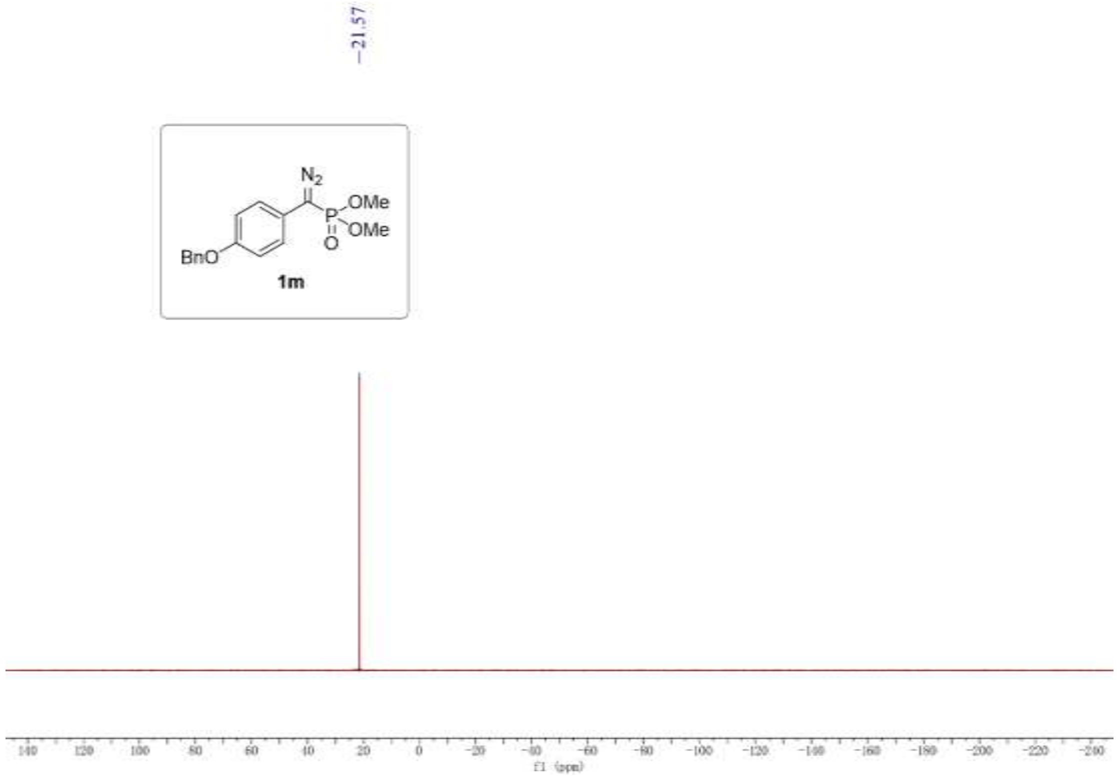

Dimethyl ((4-cyanophenyl)(diazo)methyl)phosphonate (1n)

^{1}H NMR (500 MHz, Chloroform-*d*)

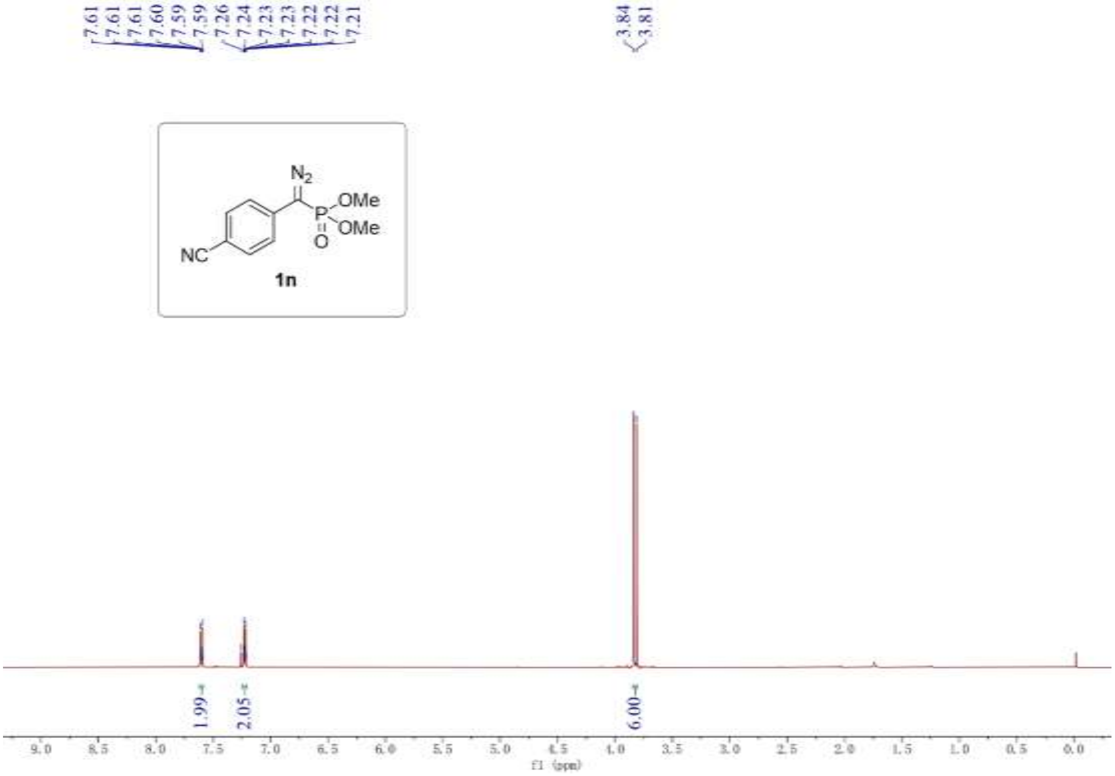

Dimethyl (diazo(3-methoxyphenyl)methyl)phosphonate (1o)

^{1}H NMR (500 MHz, Chloroform-*d*)

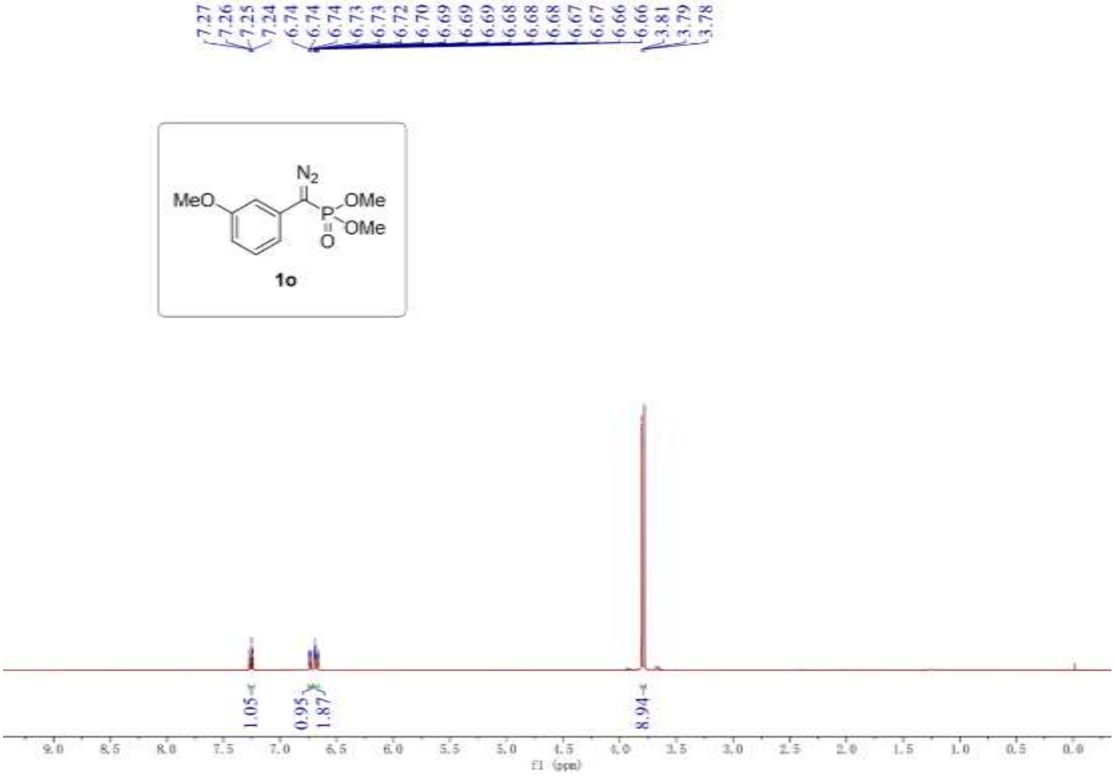

Methyl 3-(diazo(dimethoxyphosphoryl)methyl)benzoate (1p)

^{1}H NMR (500 MHz, Chloroform-*d*)

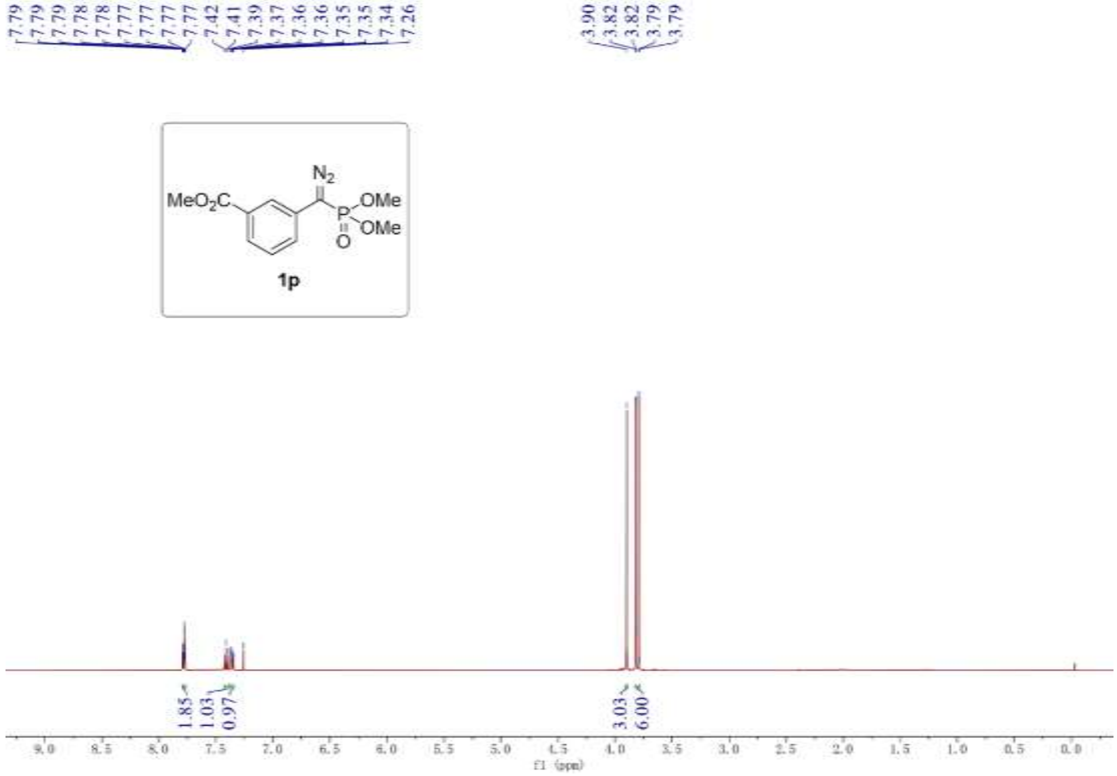

^{13}C NMR (101 MHz, Chloroform-*d*)

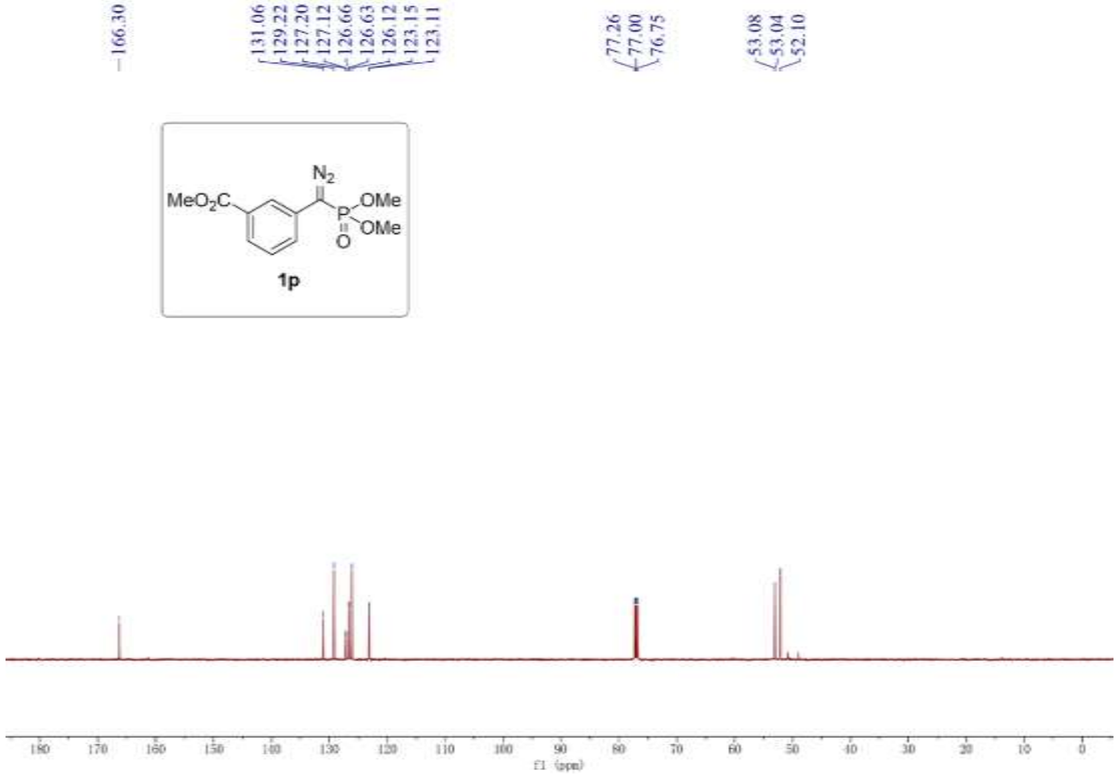

^{31}P NMR (202 MHz, Chloroform-*d*)

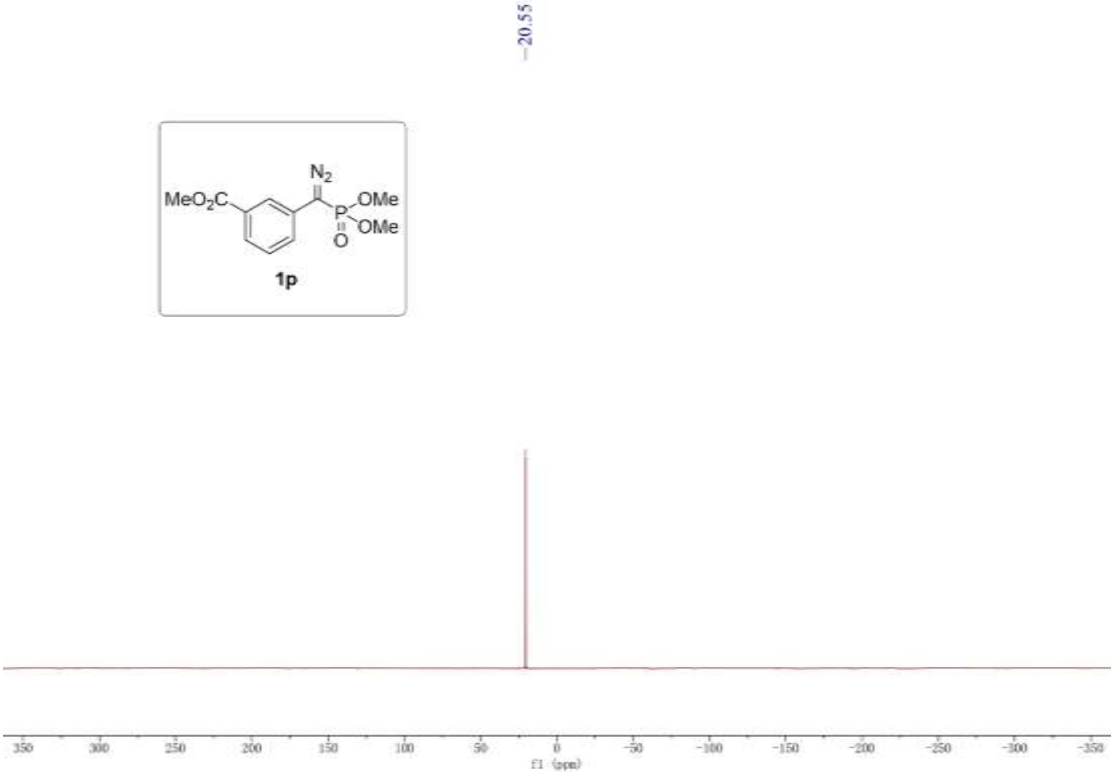

Dimethyl (diazo(3-iodophenyl)methyl)phosphonate (1q)

^{1}H NMR (400 MHz, Chloroform-*d*)

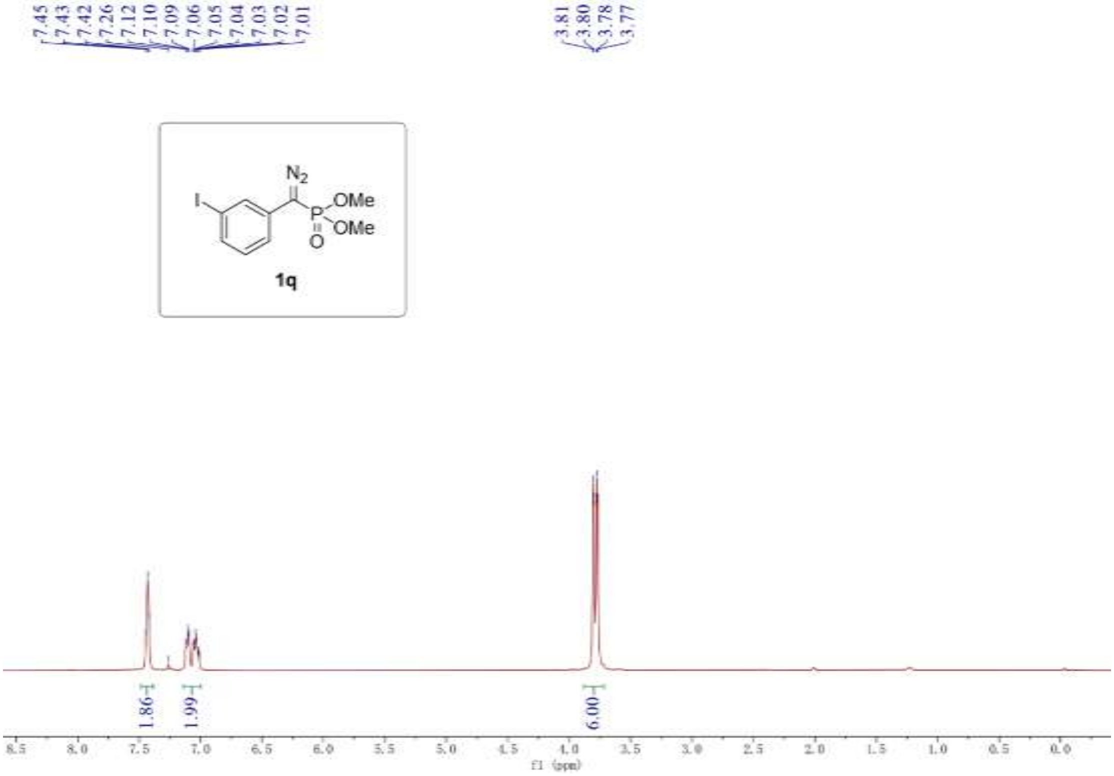

^{13}C NMR (101 MHz, Chloroform-*d*)

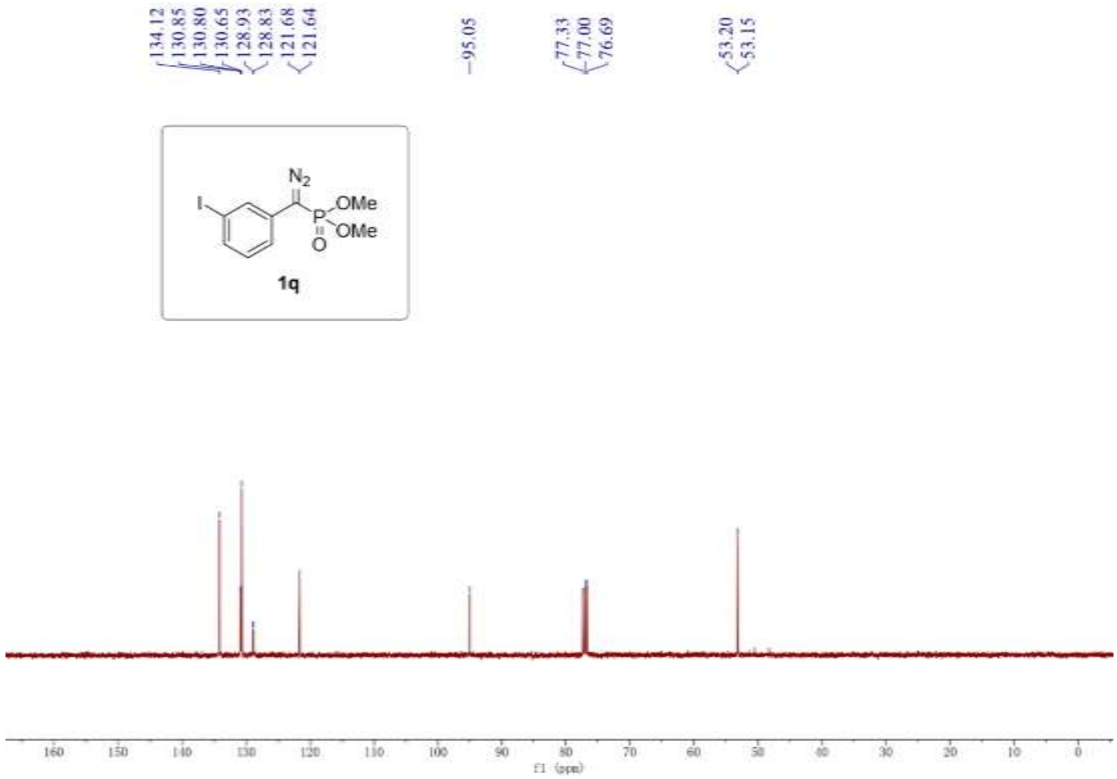

^{31}P NMR (162 MHz, Chloroform-*d*)

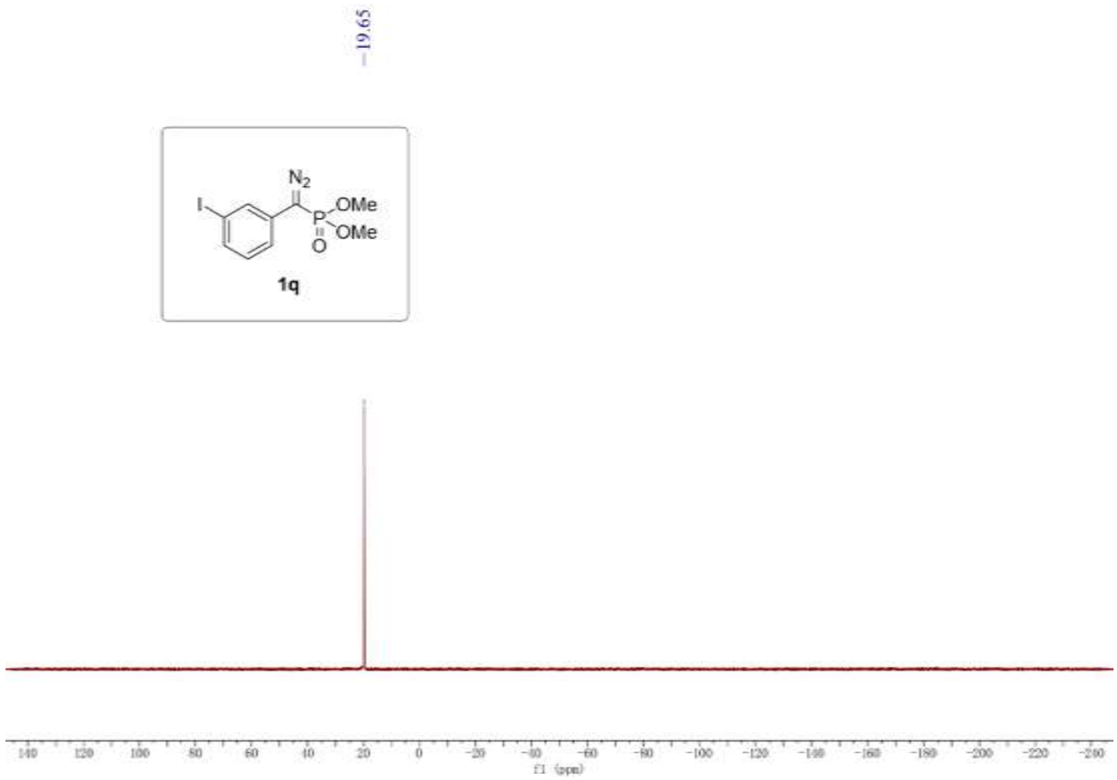

Dimethyl (diazo(2-fluorophenyl)methyl)phosphonate (1r)

^{1}H NMR (500 MHz, Chloroform-*d*)

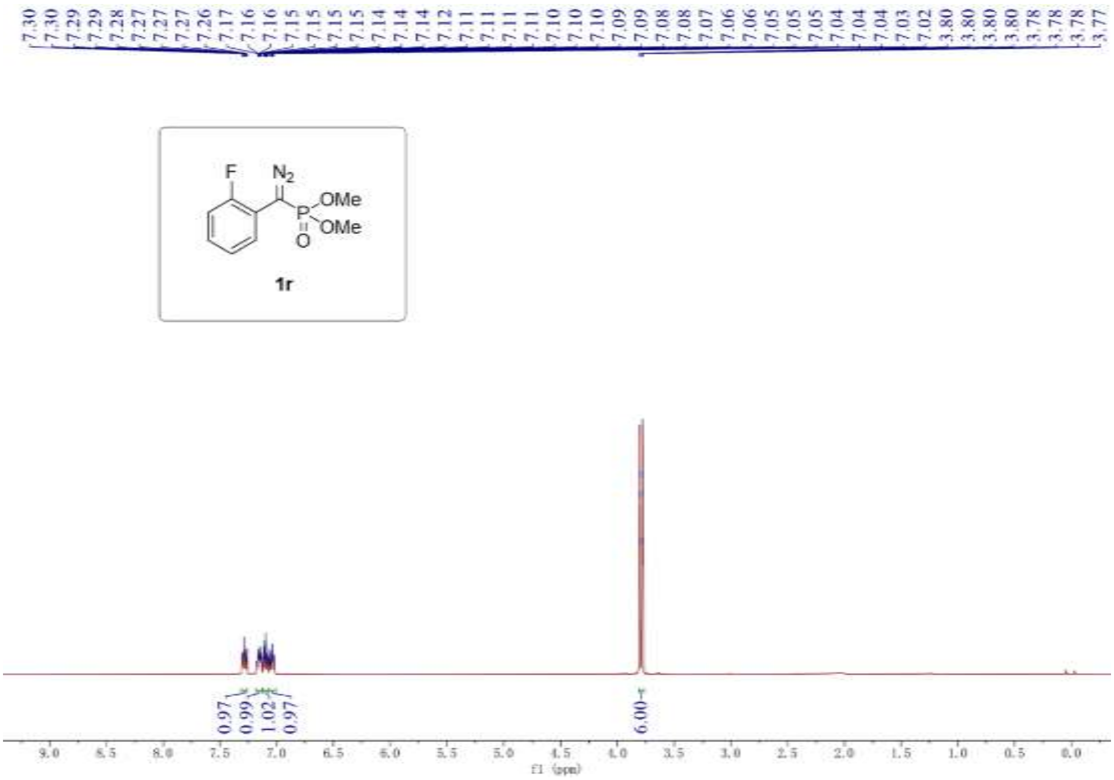

Dimethyl ((3-chloro-4-fluorophenyl)(diazo)methyl)phosphonate (1s)

^{1}H NMR (400 MHz, Chloroform-*d*)

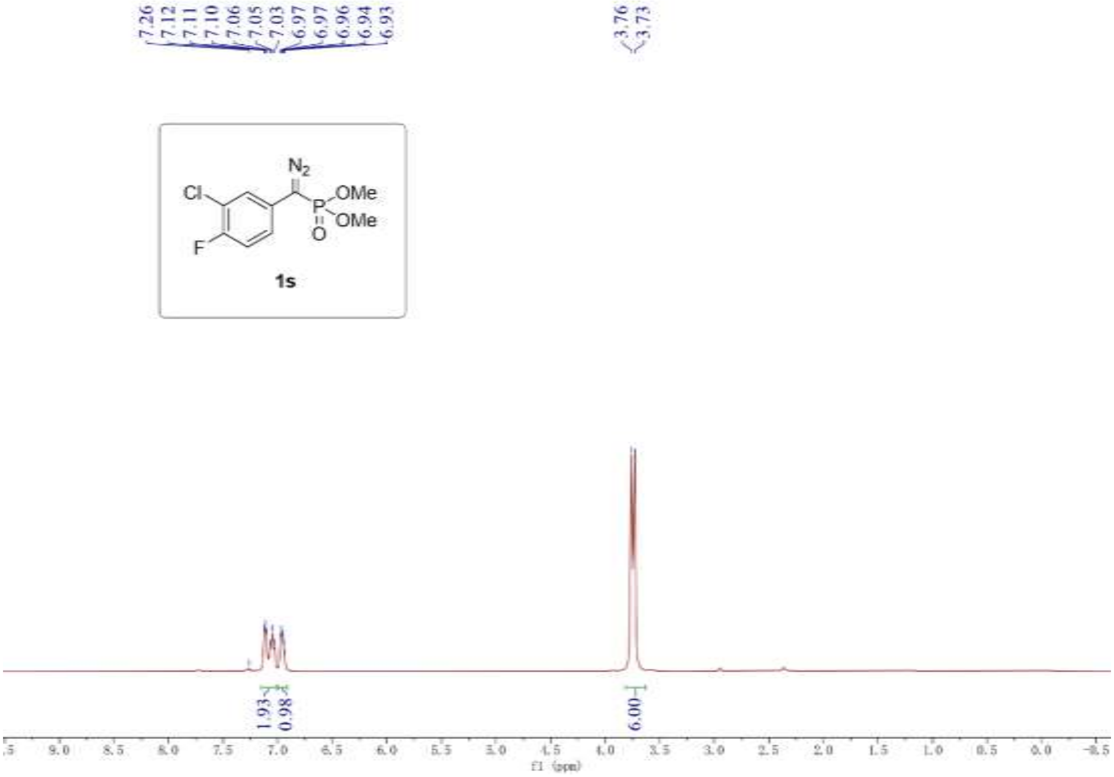

^{13}C NMR (101 MHz, Chloroform-*d*)

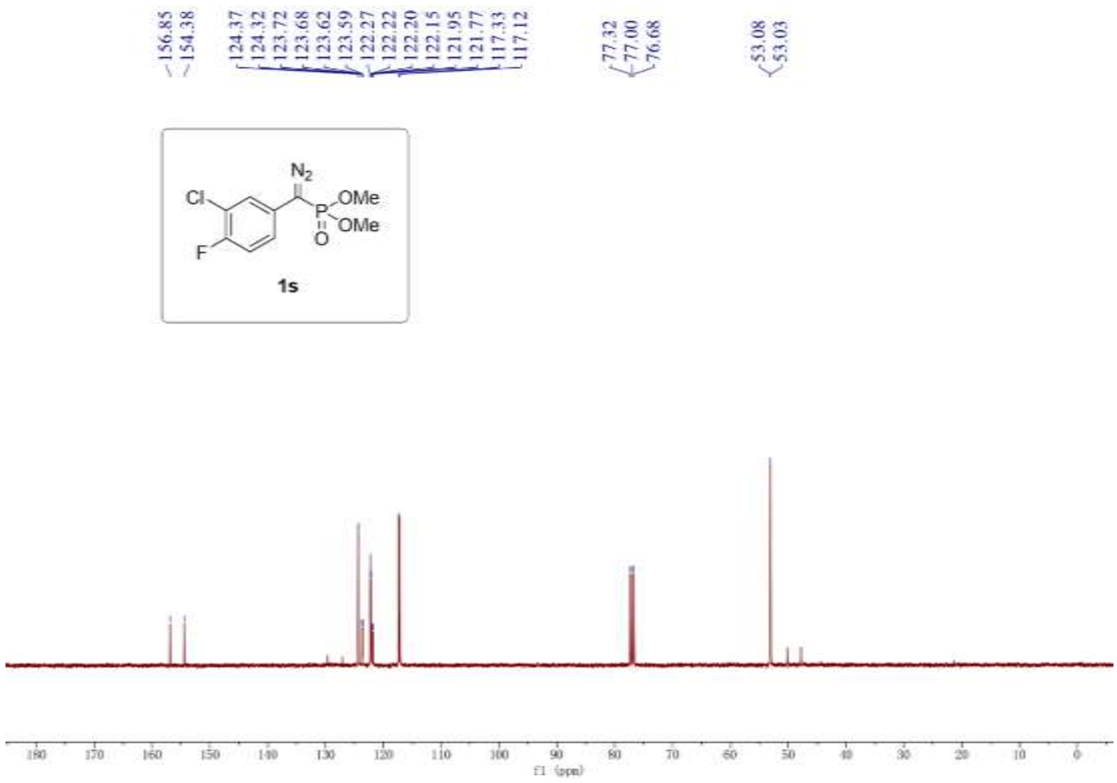

^{31}P NMR (162 MHz, Chloroform-*d*)

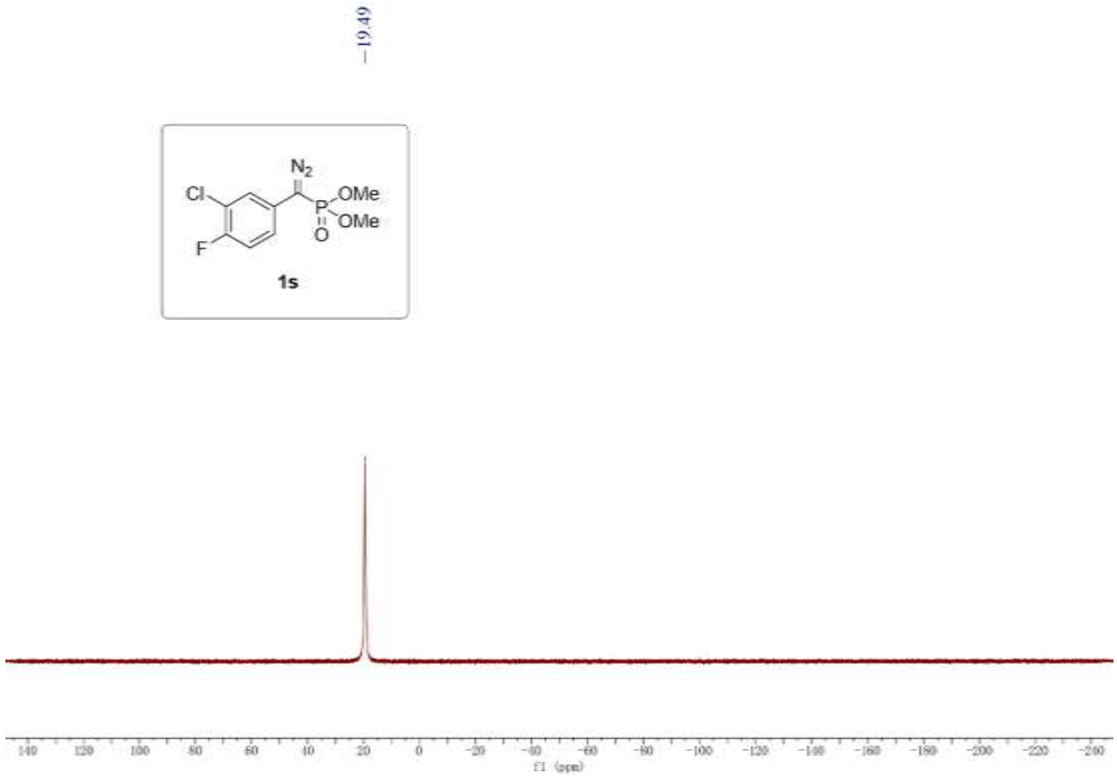

^{19}F NMR (376 MHz, Chloroform-*d*)

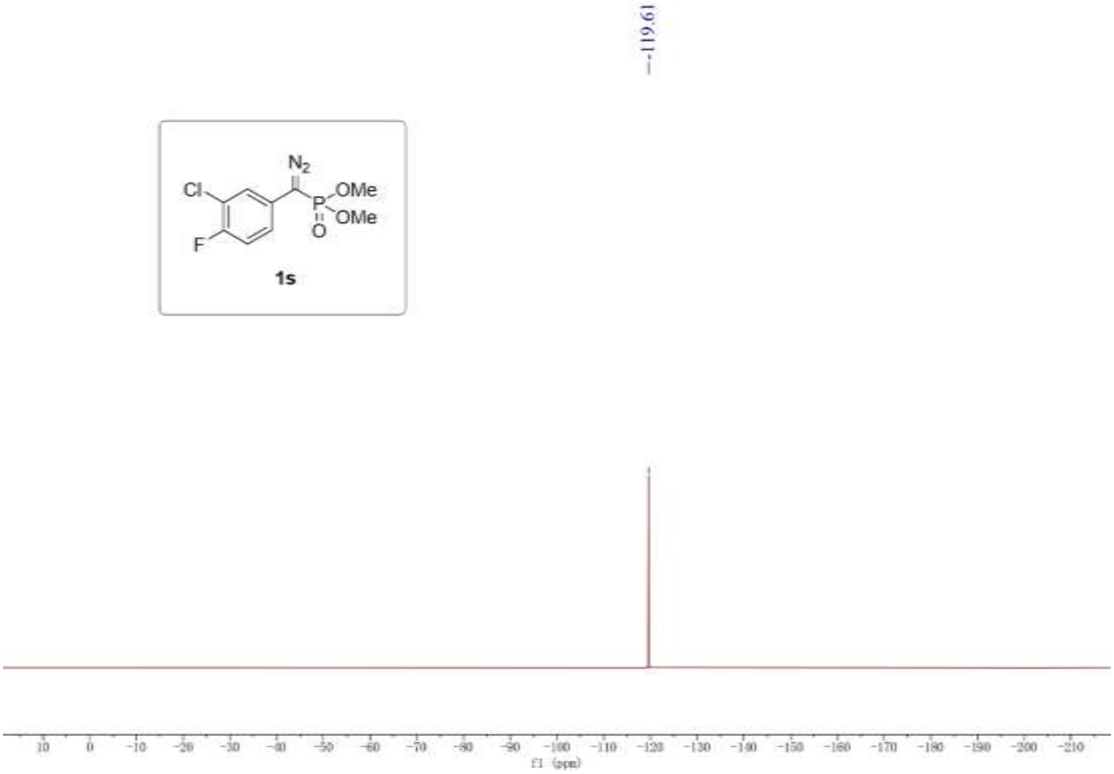

Dimethyl (diazo(4-methyl-3-nitrophenyl)methyl)phosphonate (1t)

^{1}H NMR (400 MHz, Chloroform-*d*)

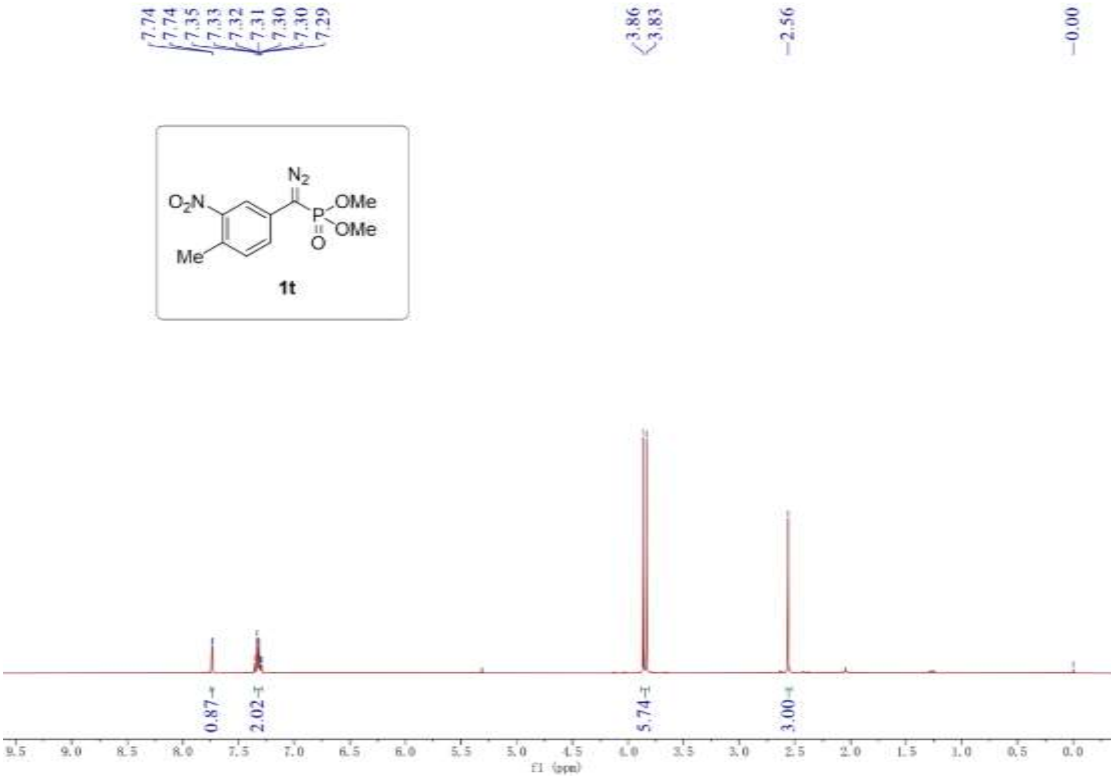

^{13}C NMR (101 MHz, Chloroform-*d*)

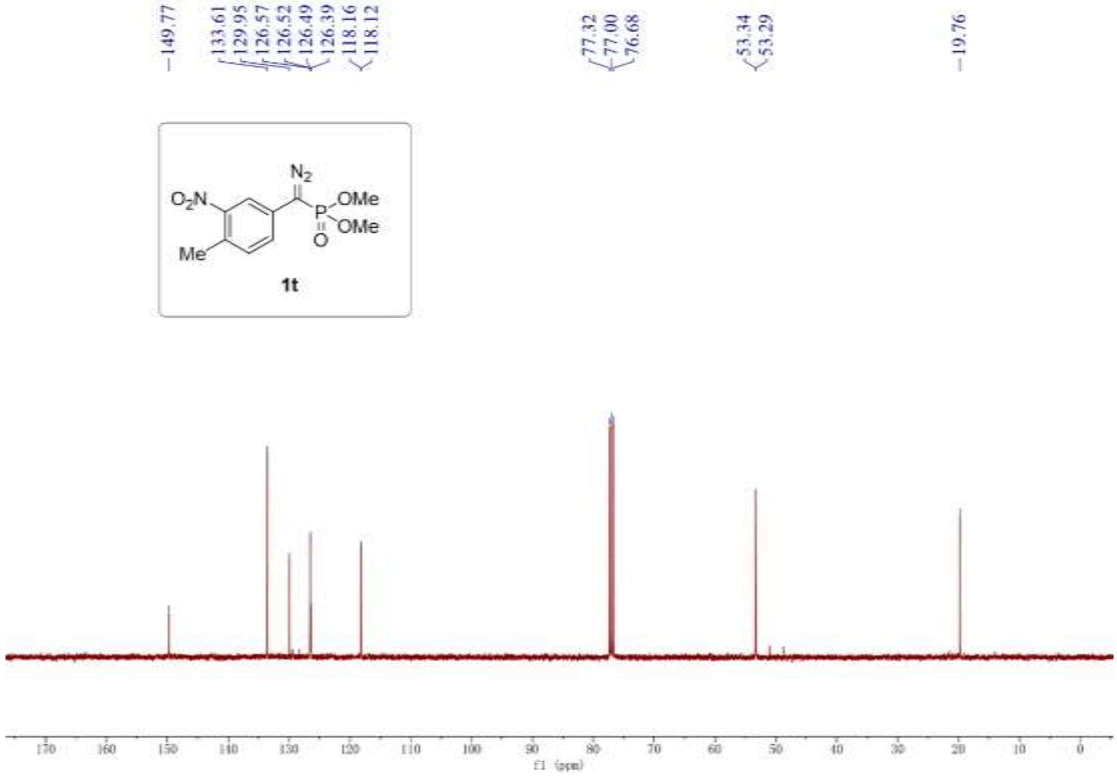

^{31}P NMR (162 MHz, Chloroform-*d*)

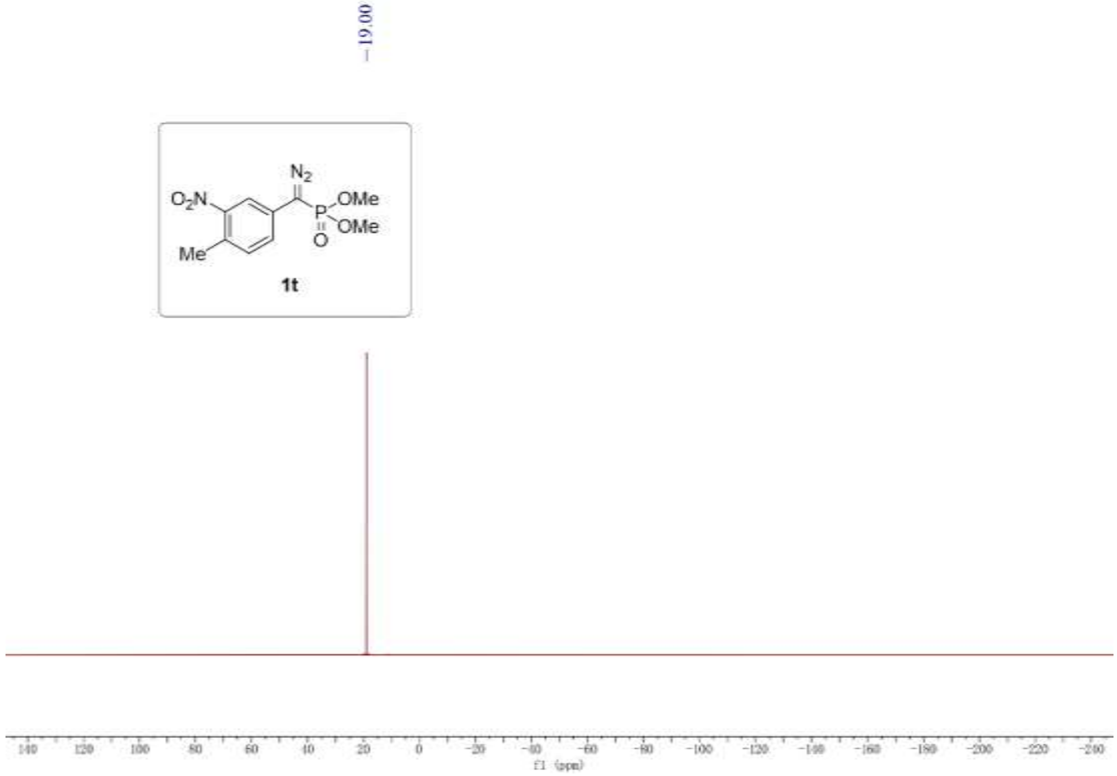

Dimethyl (diazo(3,5-dimethoxyphenyl)methyl)phosphonate (1u)

^{1}H NMR (500 MHz, Chloroform-*d*)

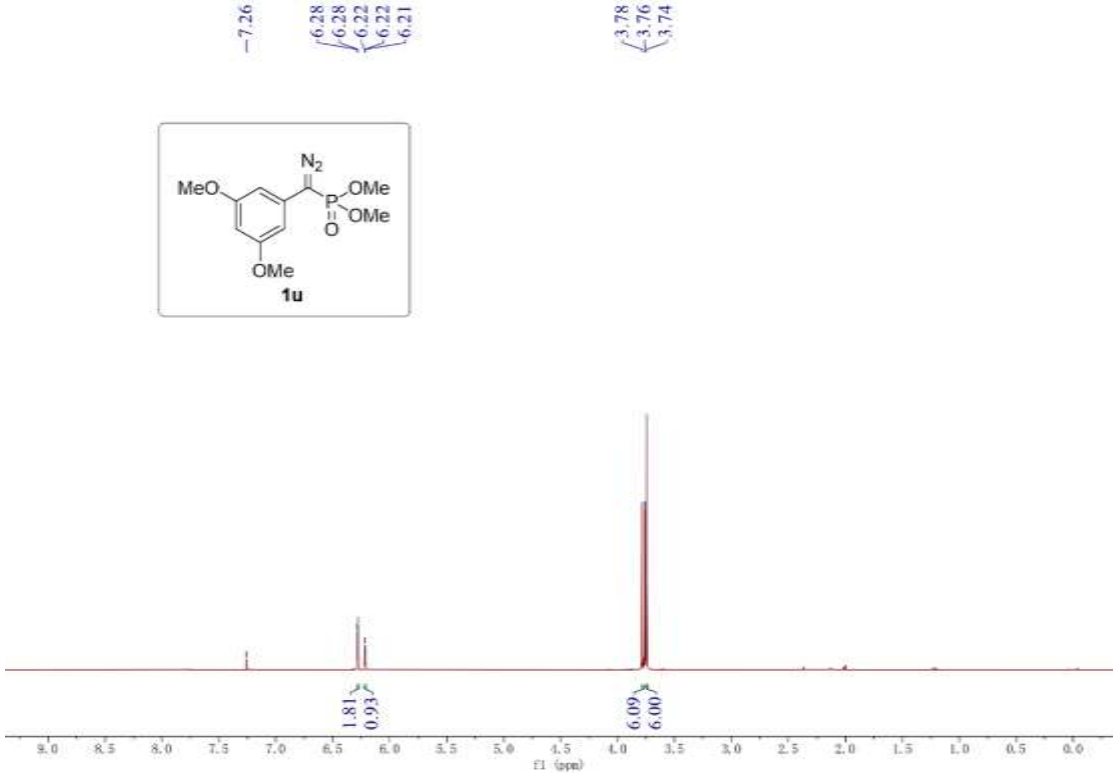

^{13}C NMR (101 MHz, Chloroform-*d*)

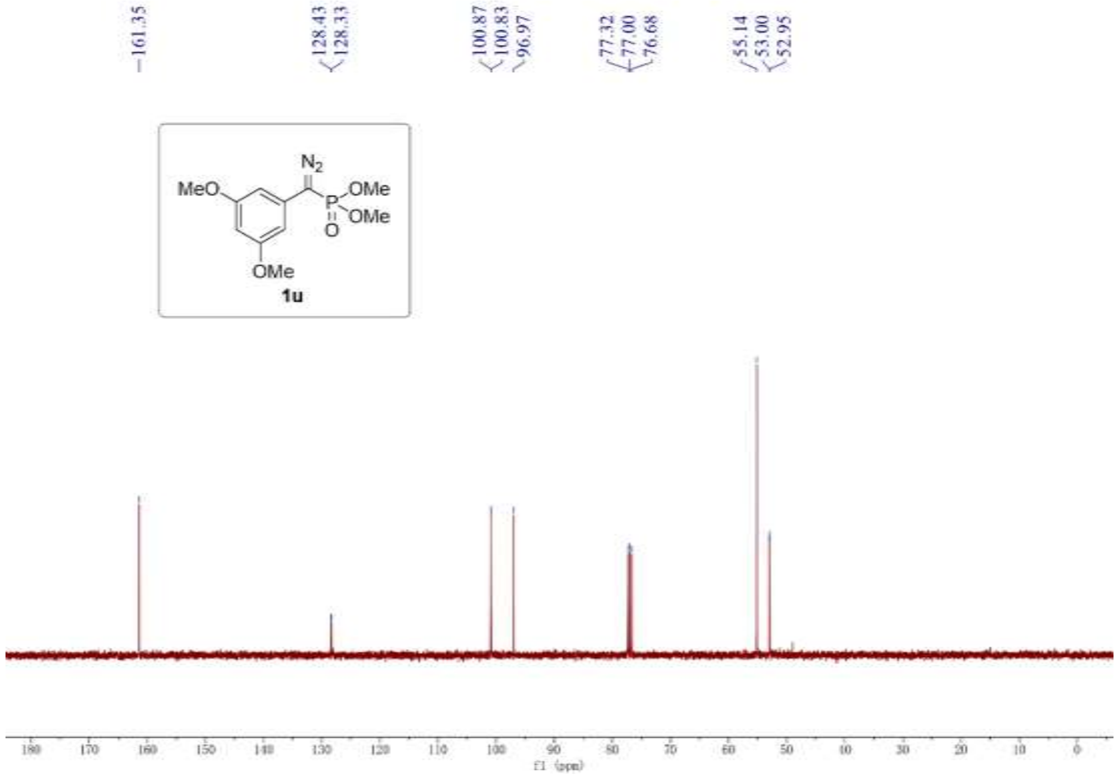

^{31}P NMR (162 MHz, Chloroform-*d*)

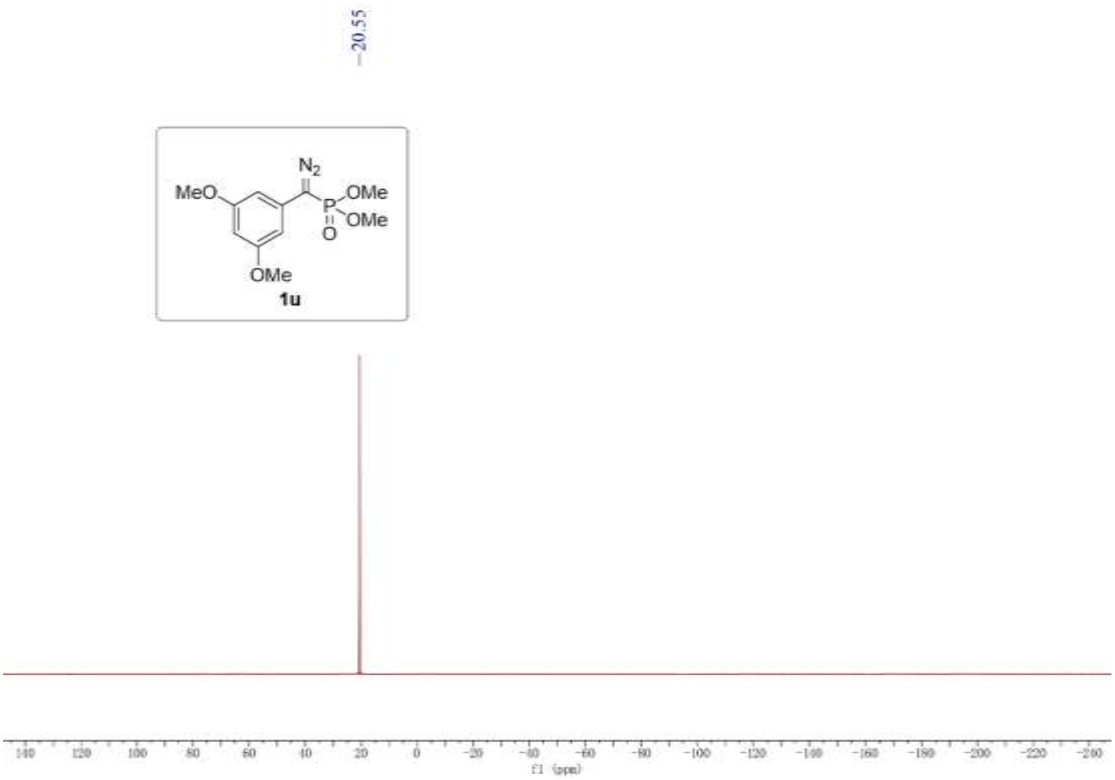

Dimethyl (diazo(9H-fluoren-2-yl)methyl)phosphonate (1v)

^{1}H NMR (500 MHz, Chloroform-*d*)

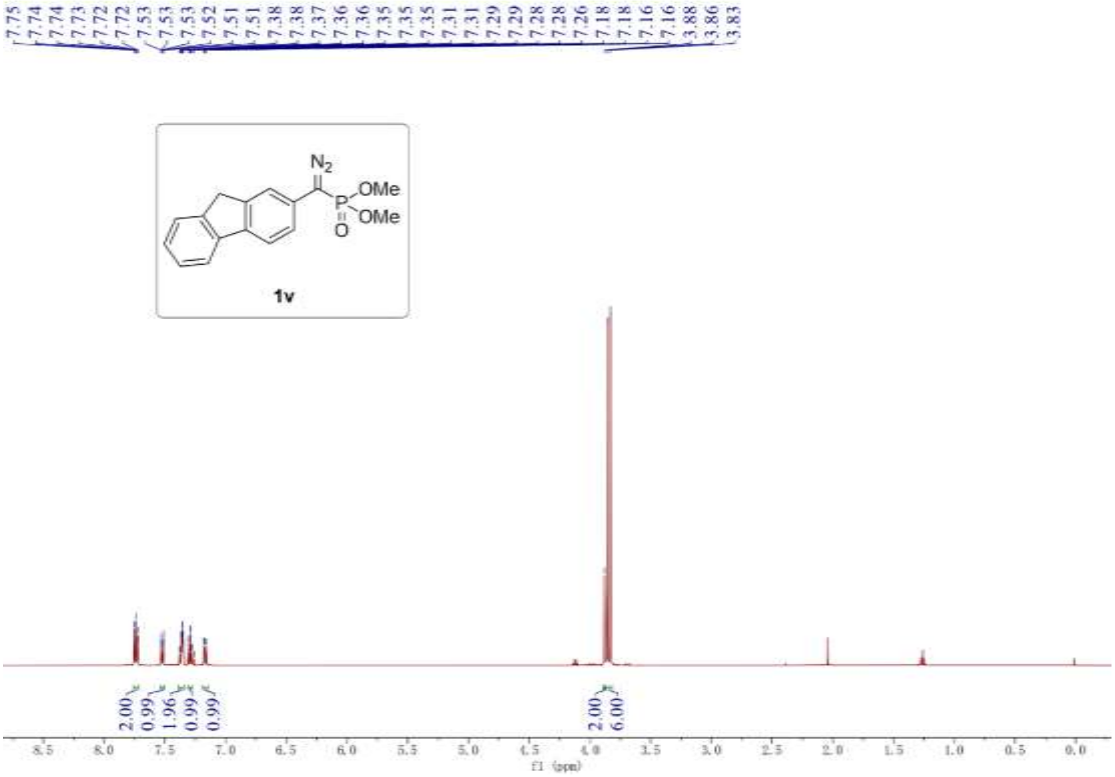

^{13}C NMR (126 MHz, Chloroform-*d*)

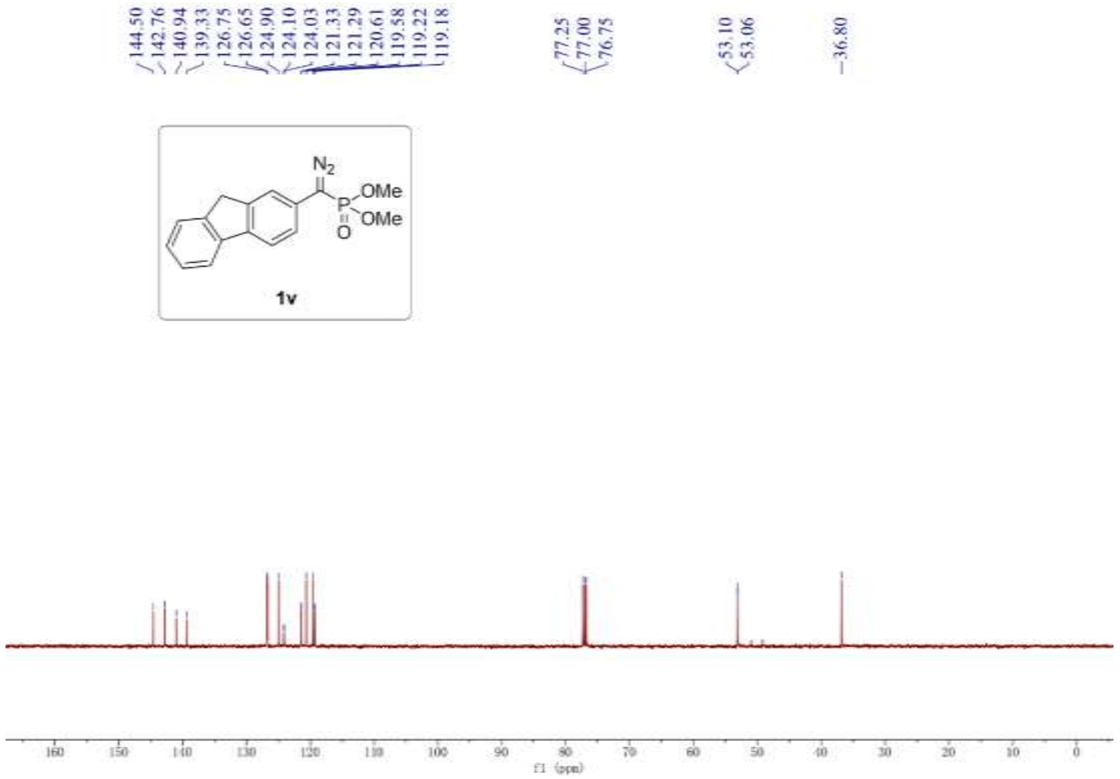

^{31}P NMR (202 MHz, Chloroform-*d*)

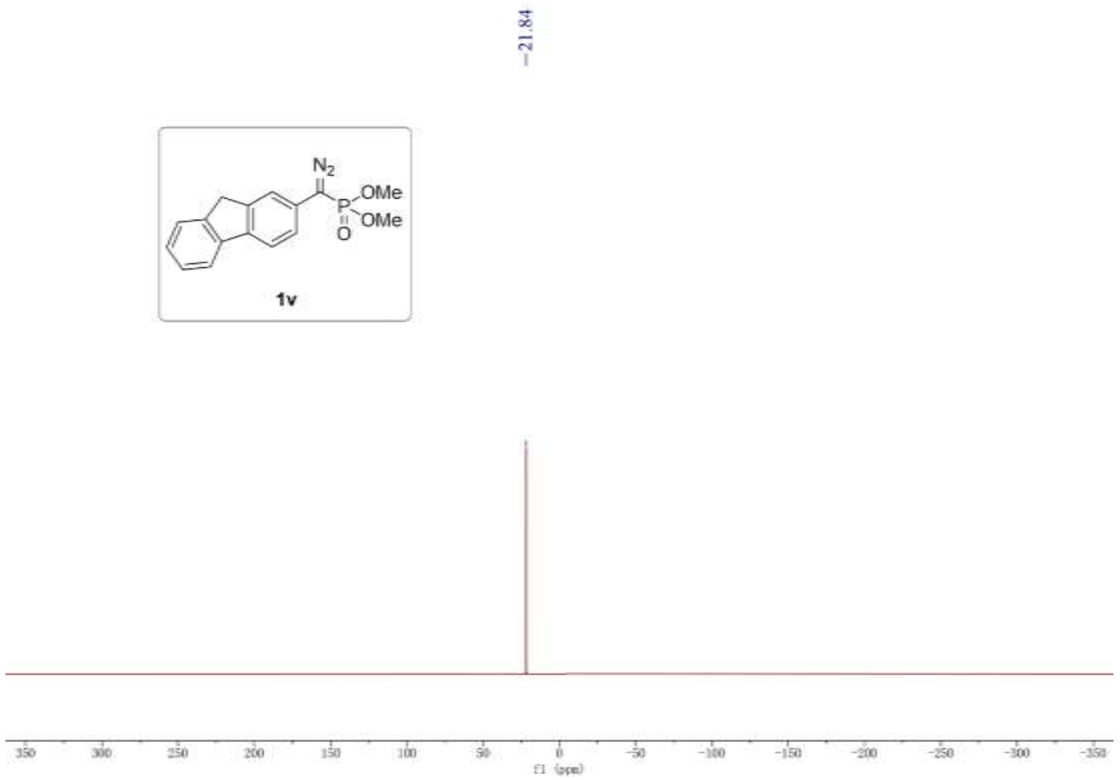

Dimethyl (diazo(1H-indol-5-yl)methyl)phosphonate (1w)

^{1}H NMR (400 MHz, Chloroform-*d*)

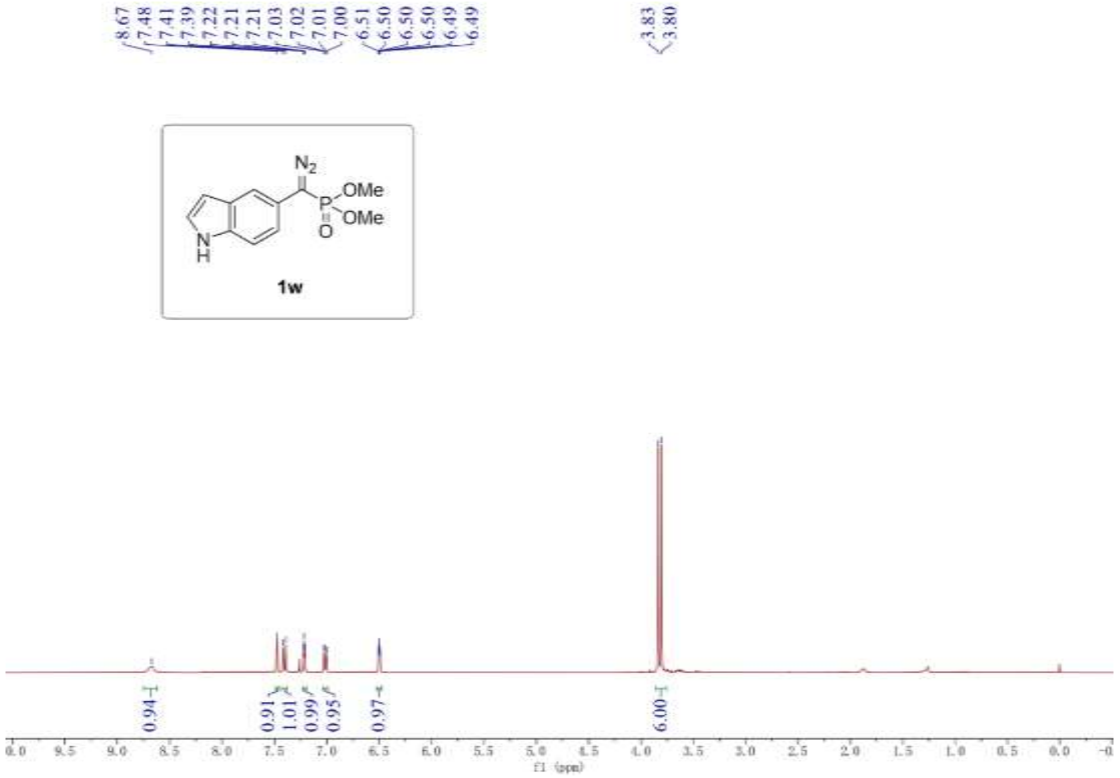

^{13}C NMR (101 MHz, Chloroform-*d*)

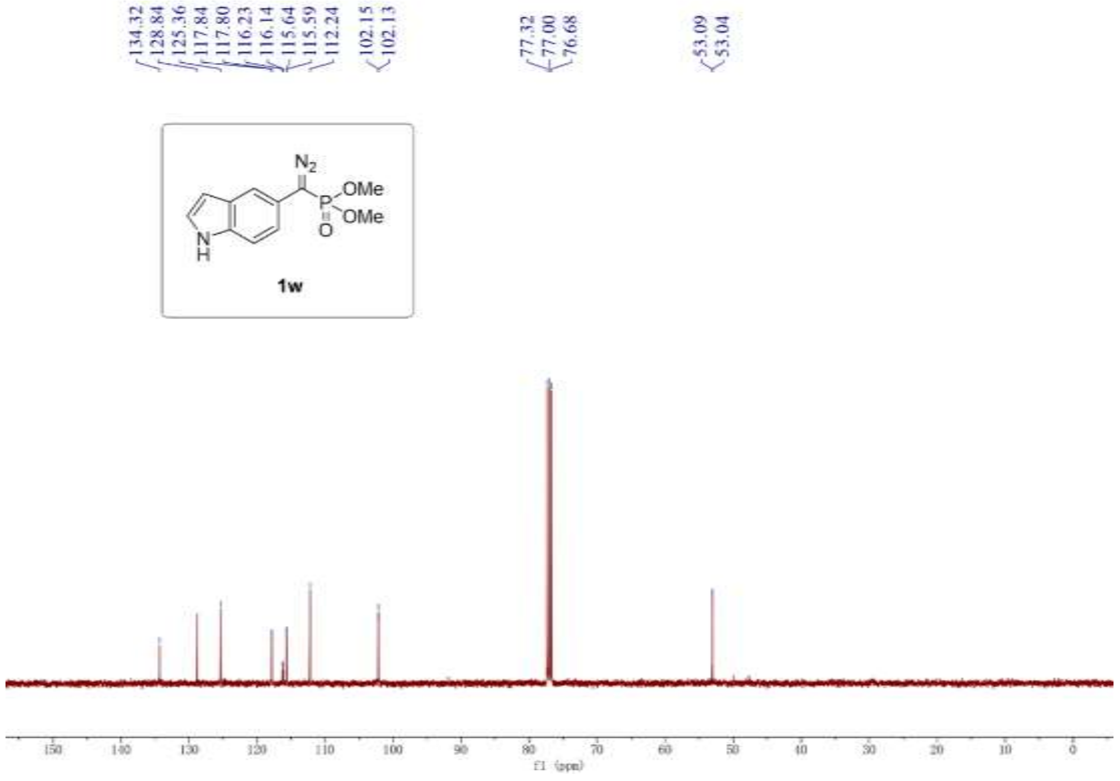

^{31}P NMR (162 MHz, Chloroform-*d*)

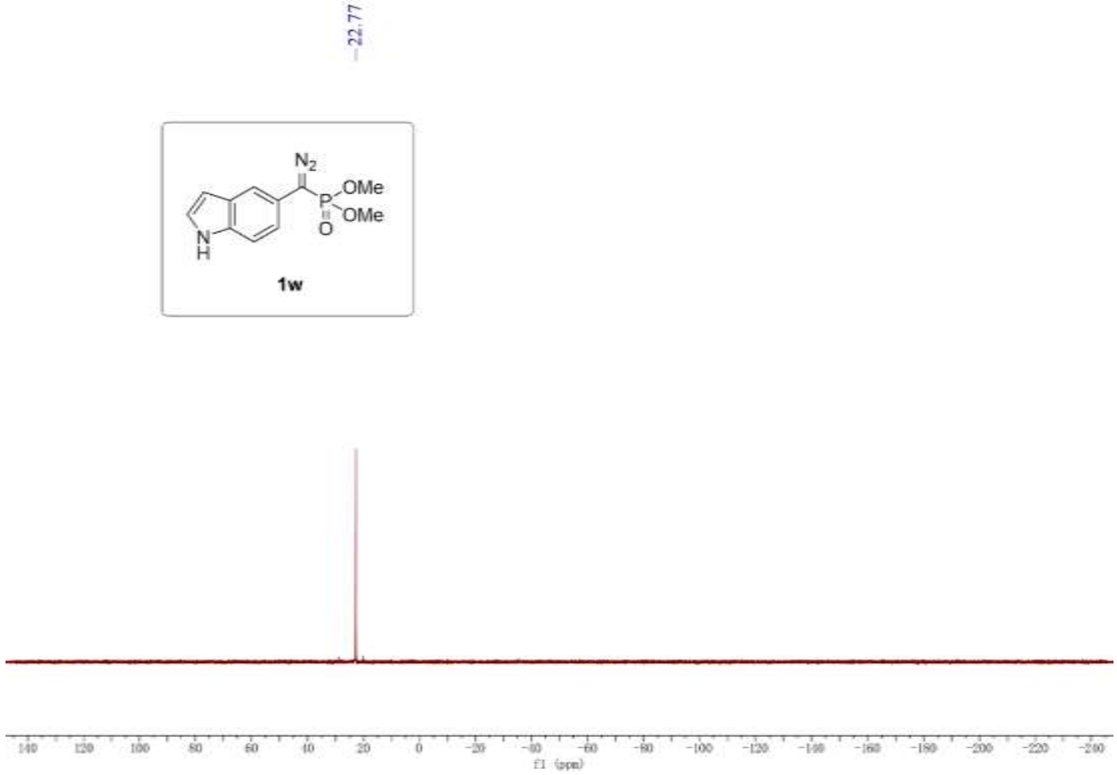

Dimethyl (benzo[d][1,3]dioxol-5-yl(diazo)methyl)phosphonate (1x)

^{1}H NMR (400 MHz, Chloroform-*d*)

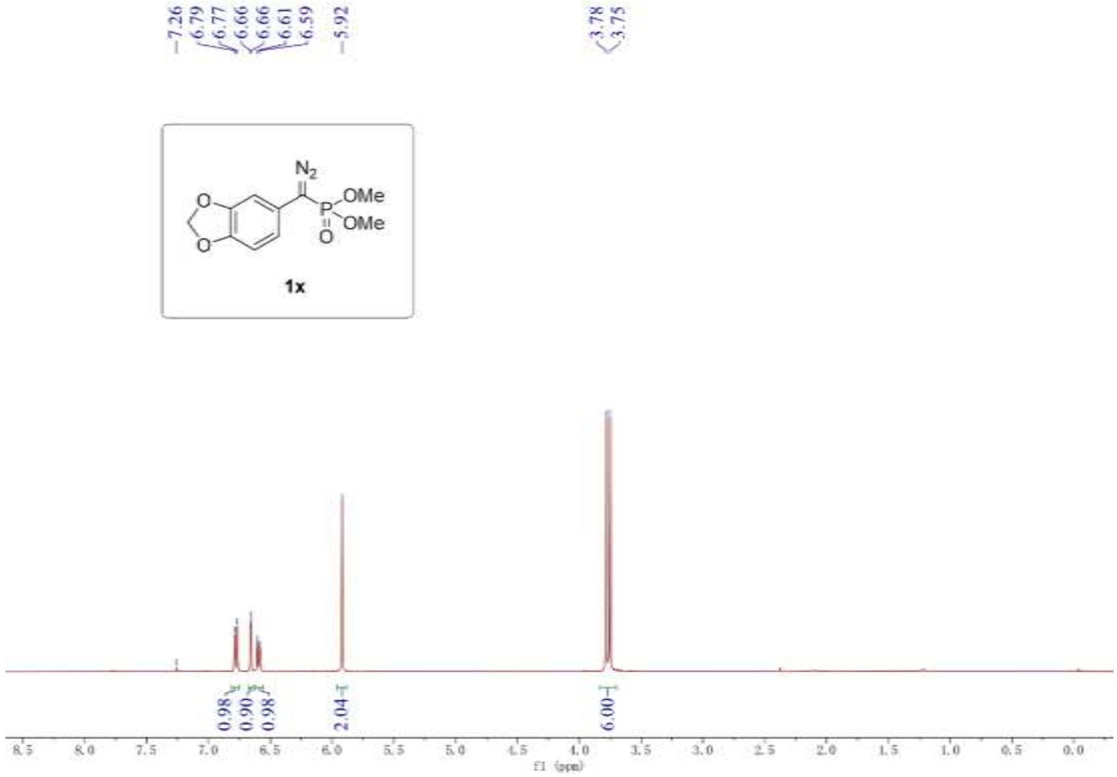

^{13}C NMR (101 MHz, Chloroform-*d*)

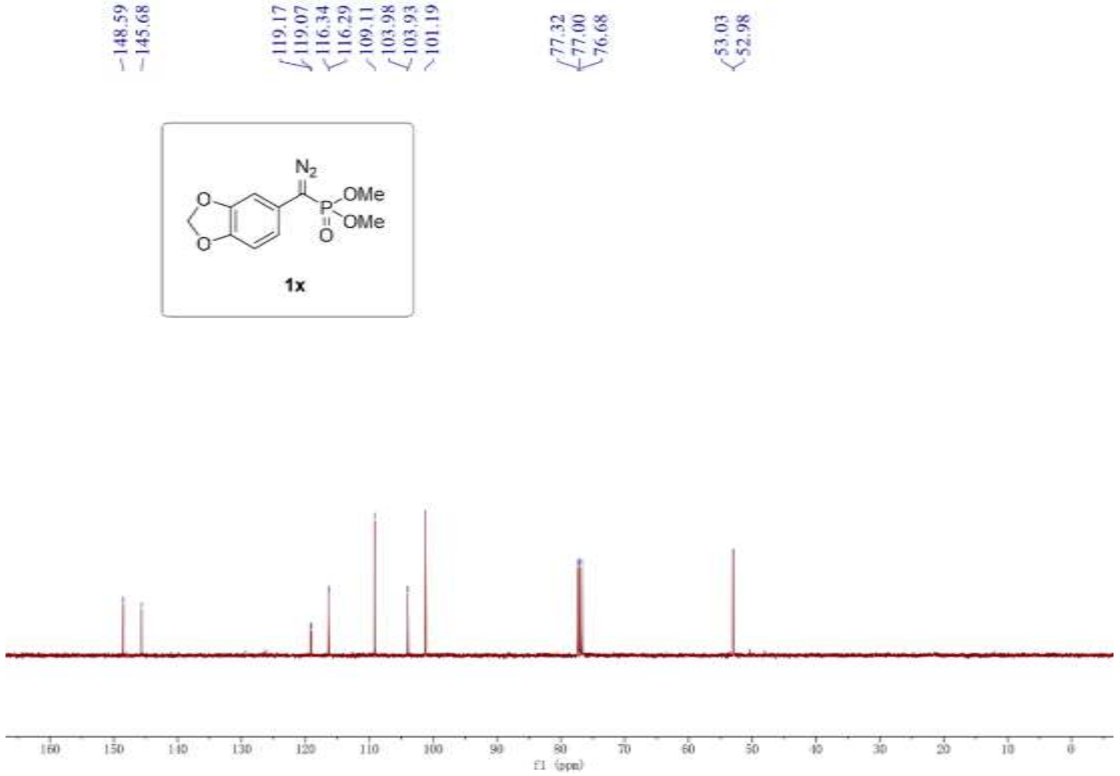

^{31}P NMR (162 MHz, Chloroform-*d*)

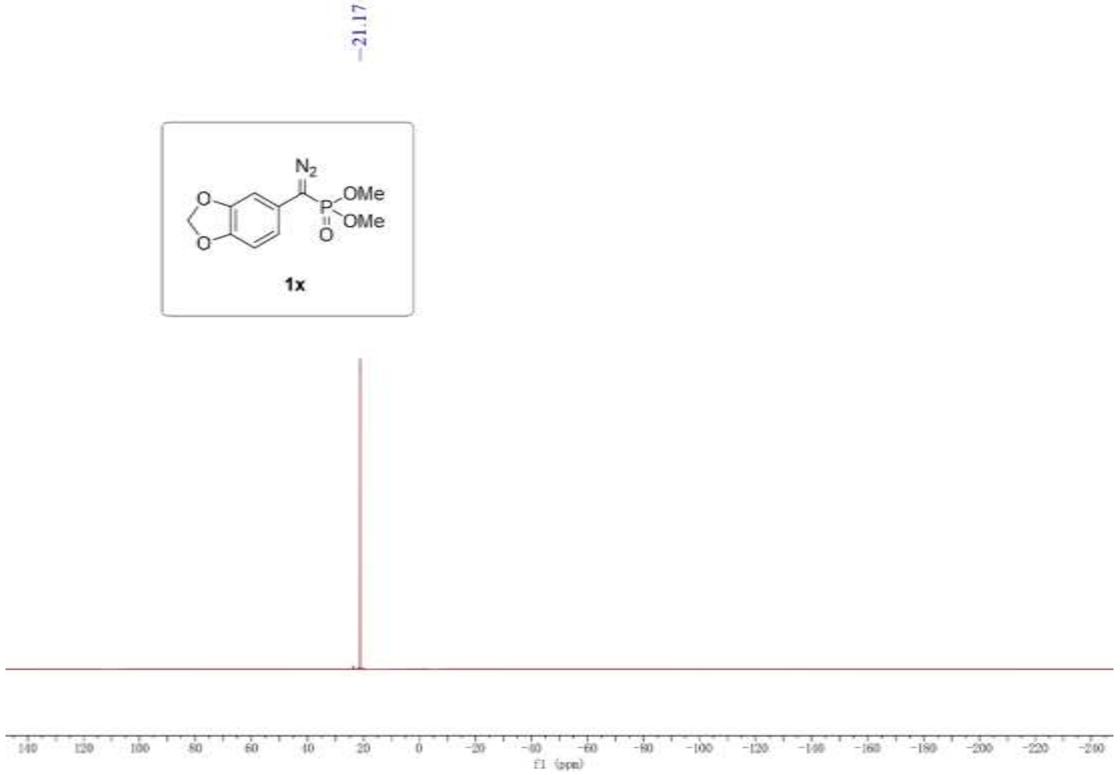

Dimethyl (diazo(dibenzo[b,d]furan-1-yl)methyl)phosphonate (1y)

^{1}H NMR (400 MHz, Chloroform-*d*)

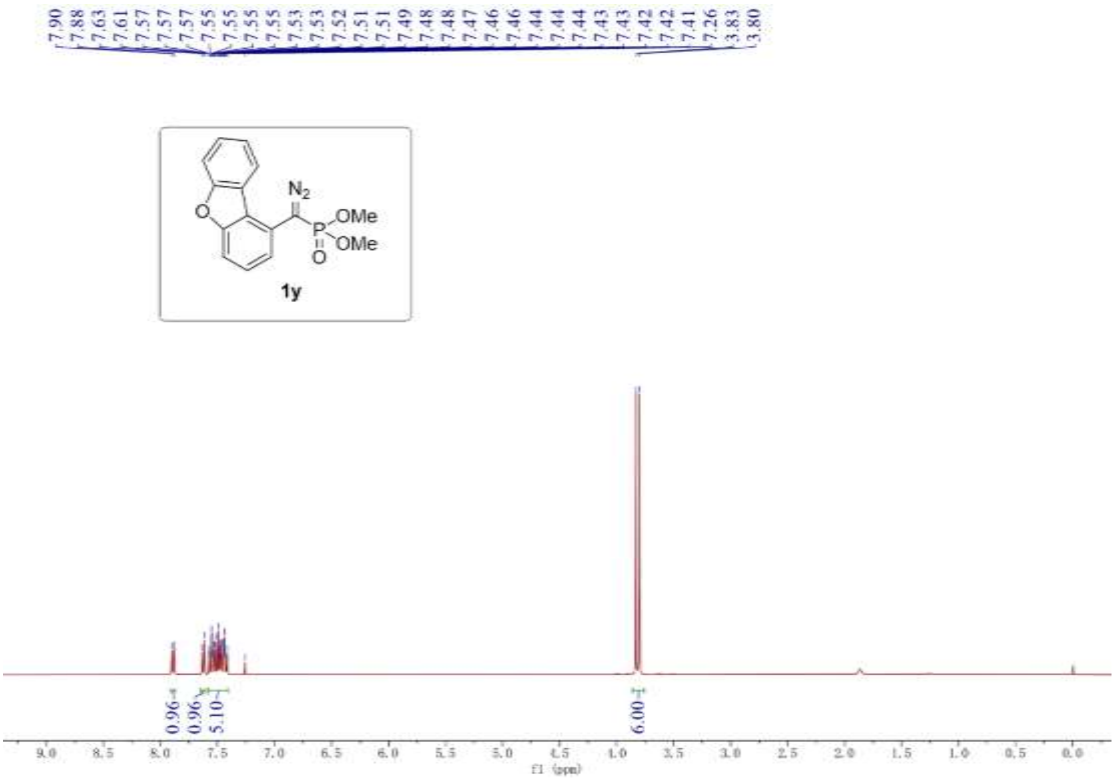

^{13}C NMR (101 MHz, Chloroform-*d*)

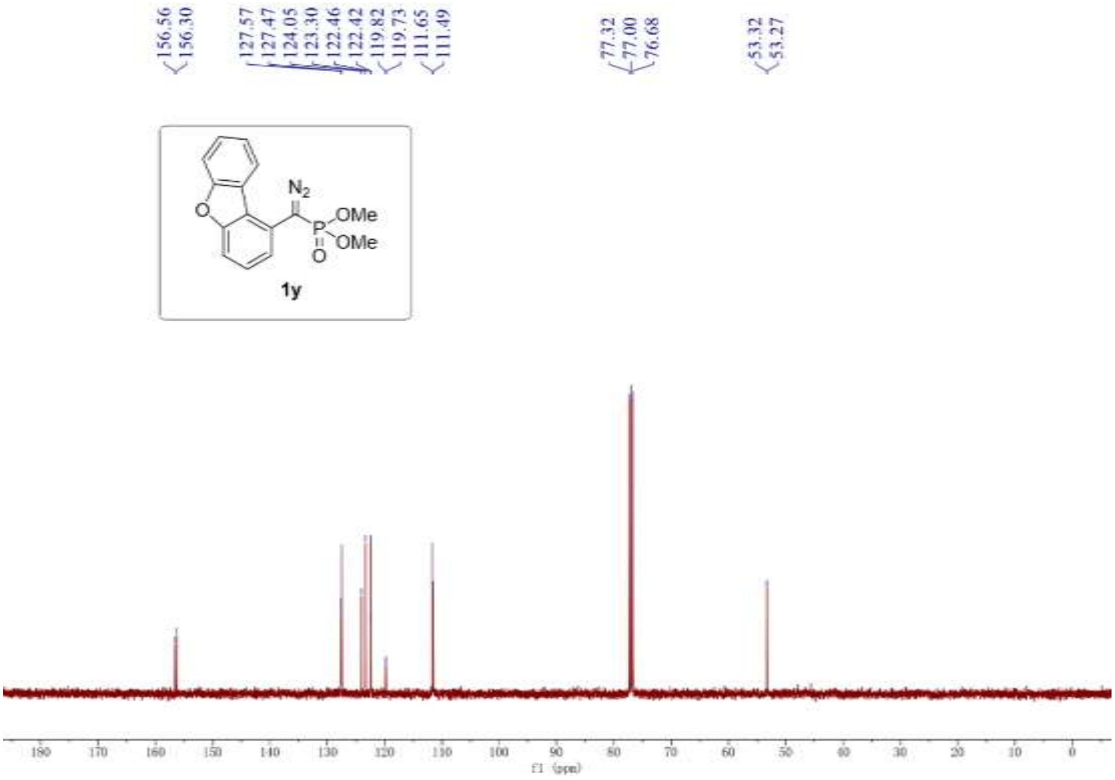

^{31}P NMR (162 MHz, Chloroform-*d*)

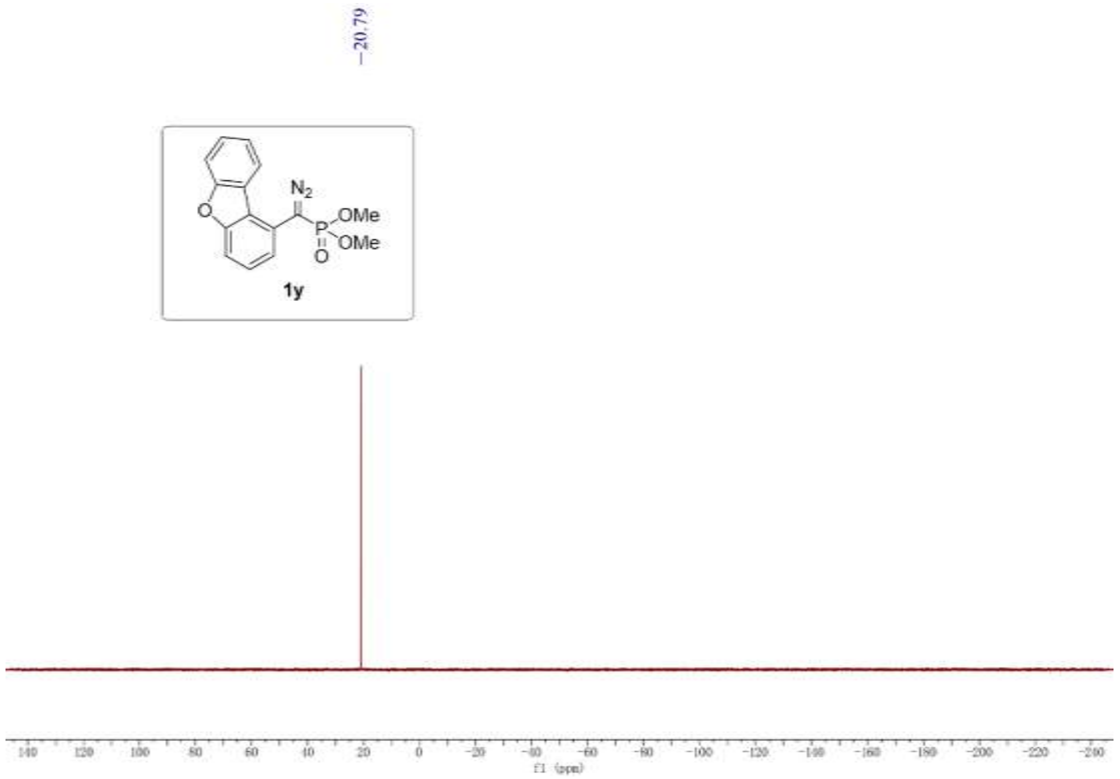

Dimethyl (diazo(dibenzo[b,d]thiophen-4-yl)methyl)phosphonate (1z)

^{1}H NMR (400 MHz, Chloroform-*d*)

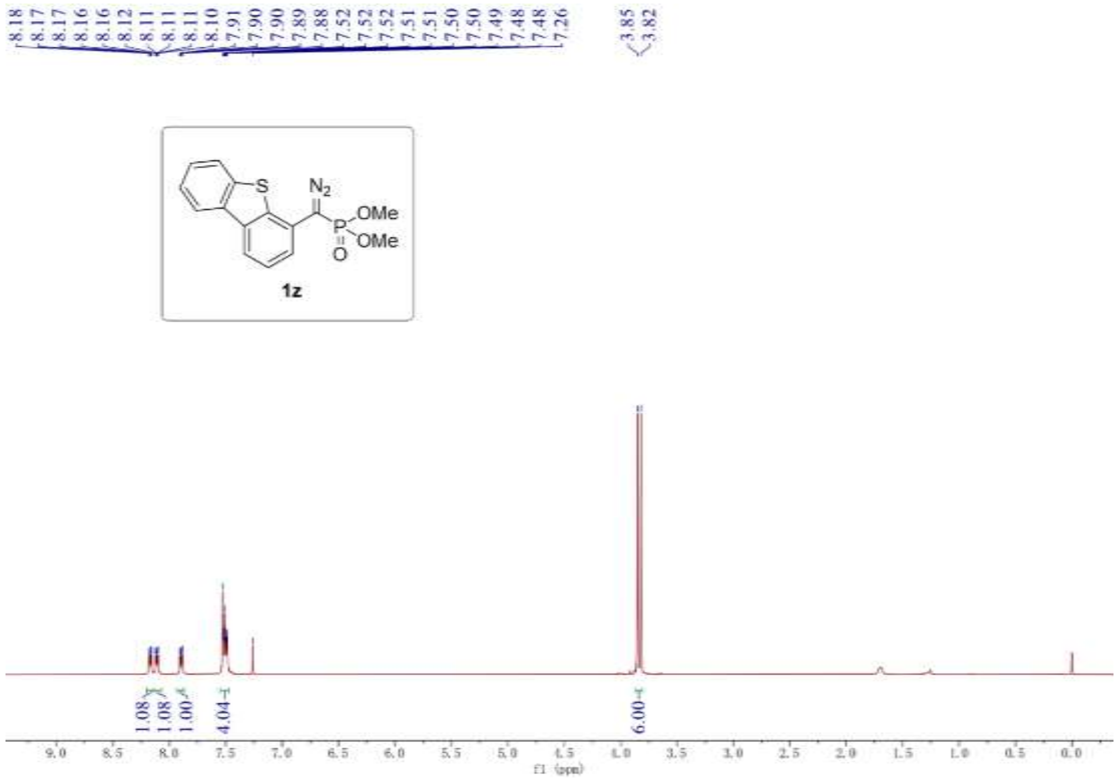

^{13}C NMR (101 MHz, Chloroform-d)

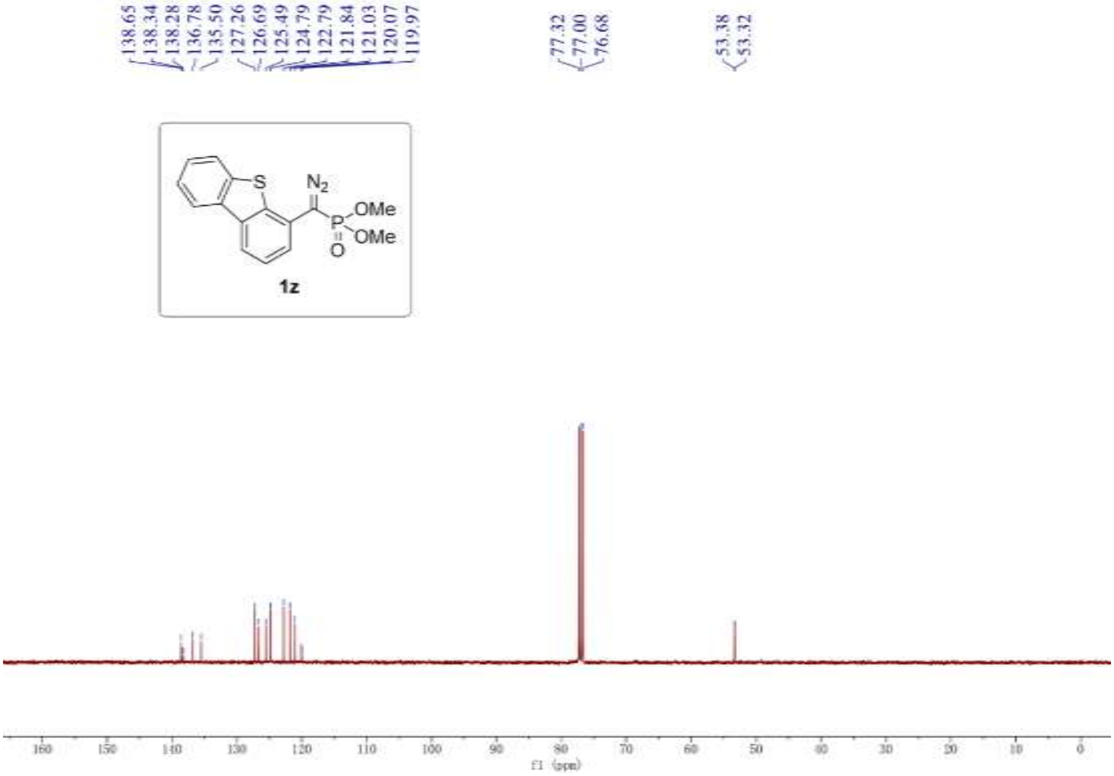

^{31}P NMR (162 MHz, Chloroform-d)

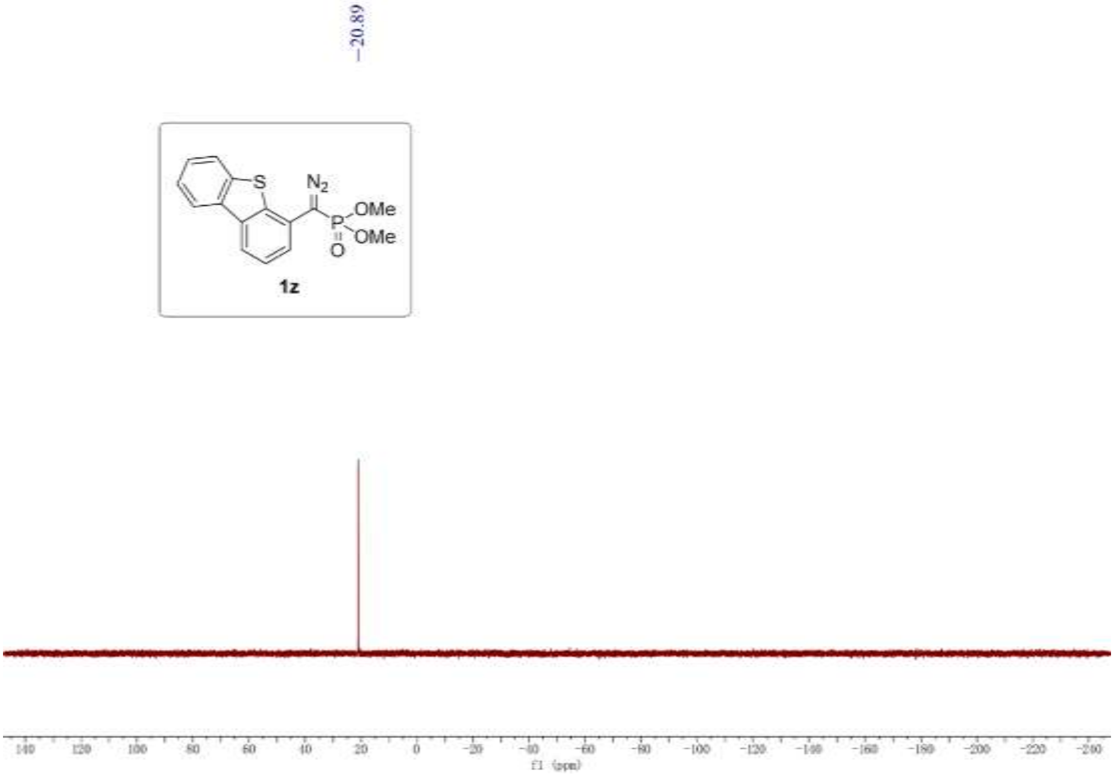

Dimethyl (diazo(phenanthren-9-yl)methyl)phosphonate (1A)

^{1}H NMR (400 MHz, Chloroform-*d*)

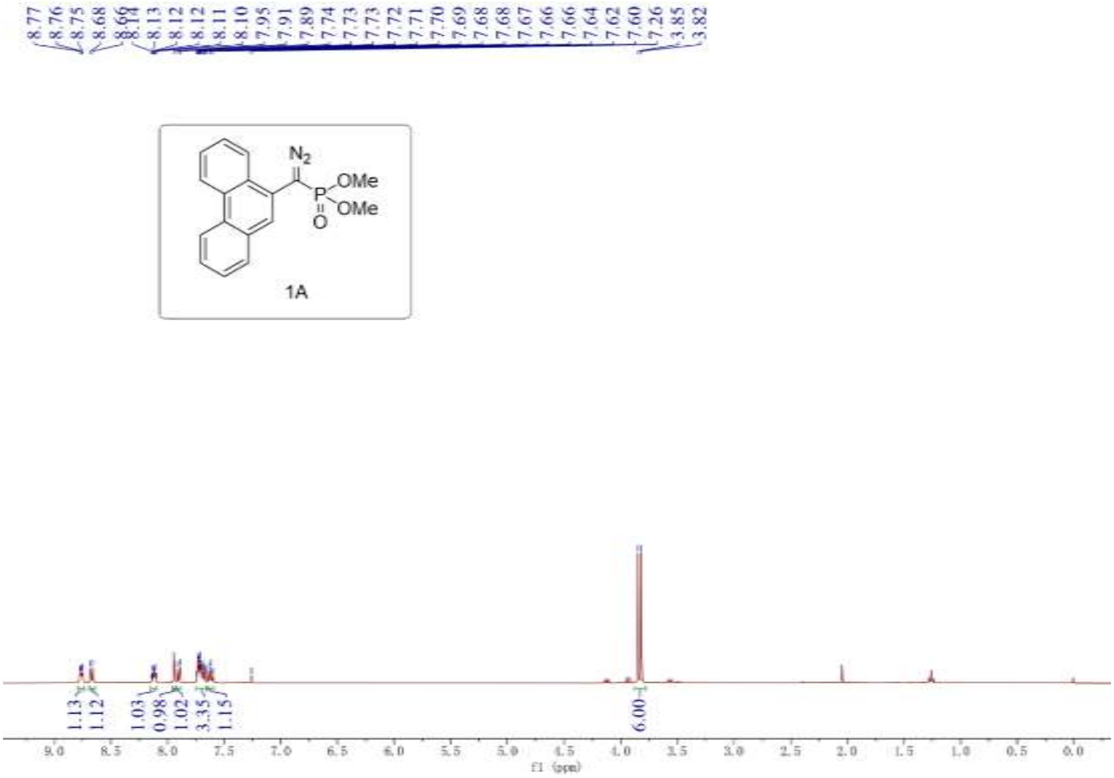

^{13}C NMR (101 MHz, Chloroform-*d*)

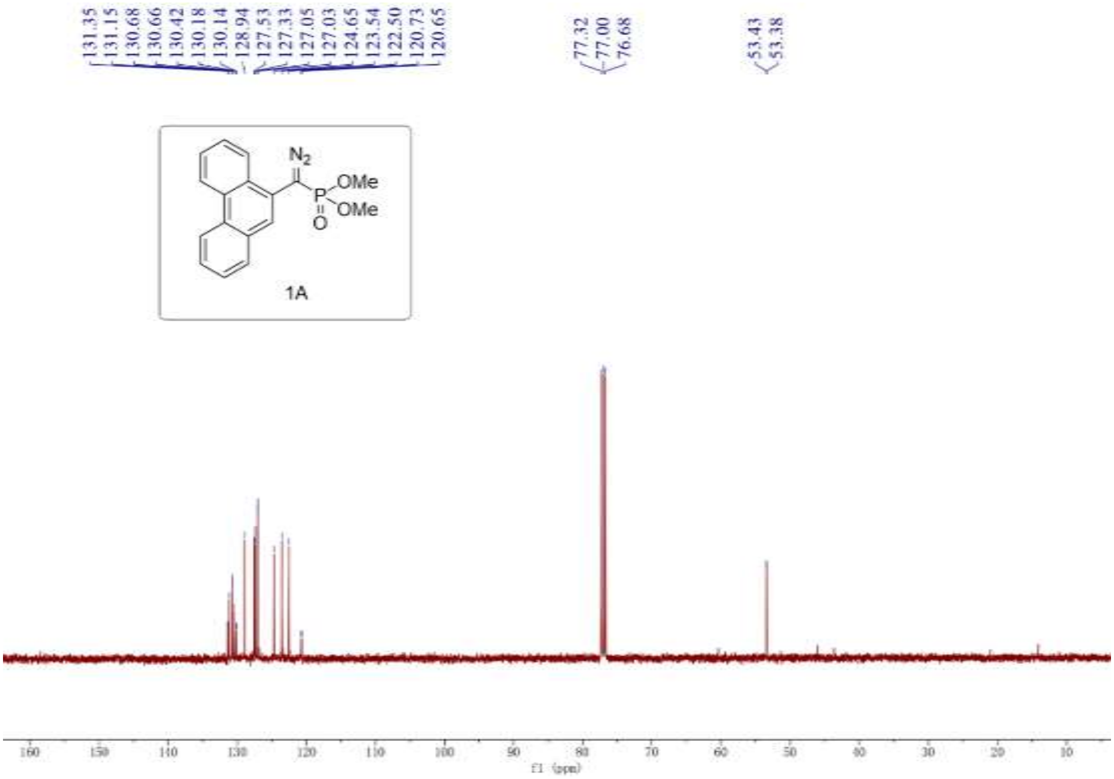

^{31}P NMR (202 MHz, Chloroform-*d*)

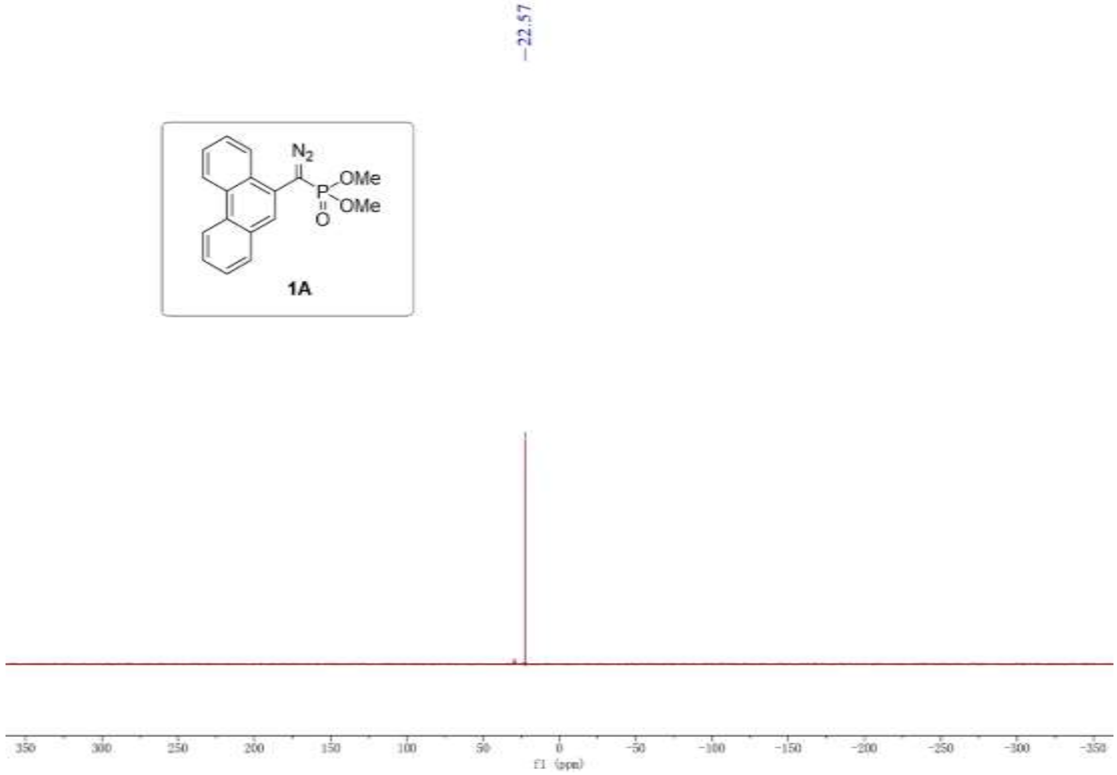

Dimethyl (diazo(naphthalen-2-yl)methyl)phosphonate (1B)

^{1}H NMR (400 MHz, Chloroform-*d*)

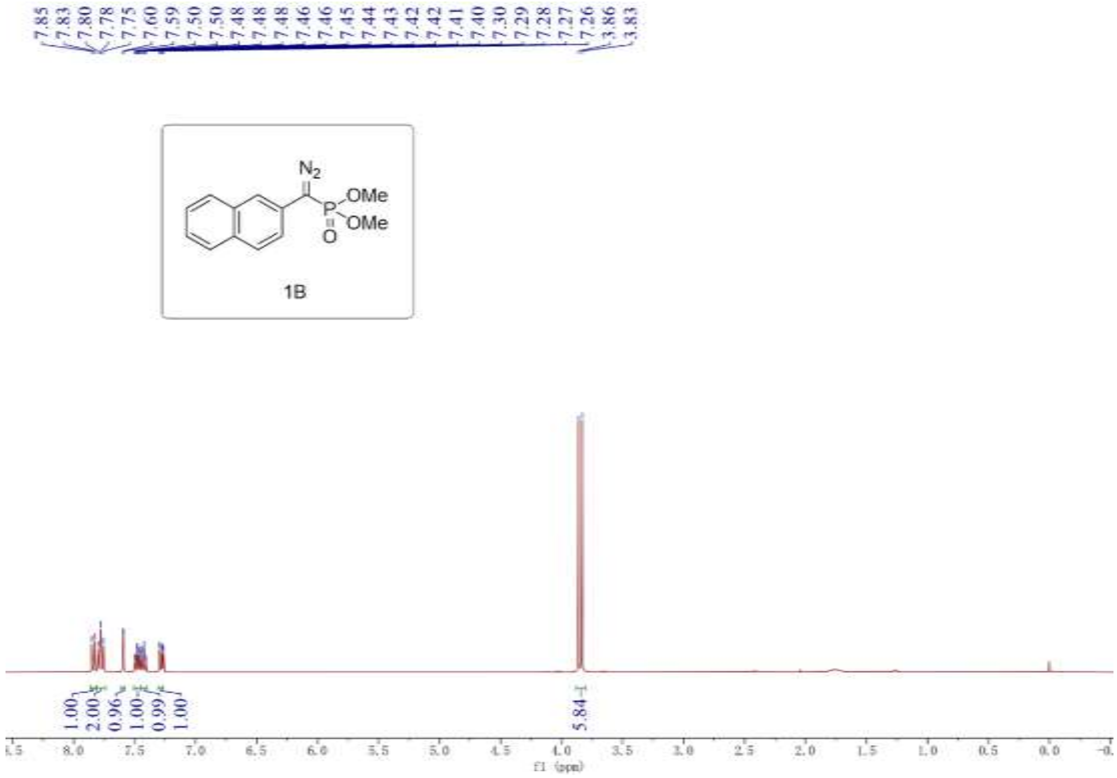

^{13}C NMR (101 MHz, Chloroform-*d*)

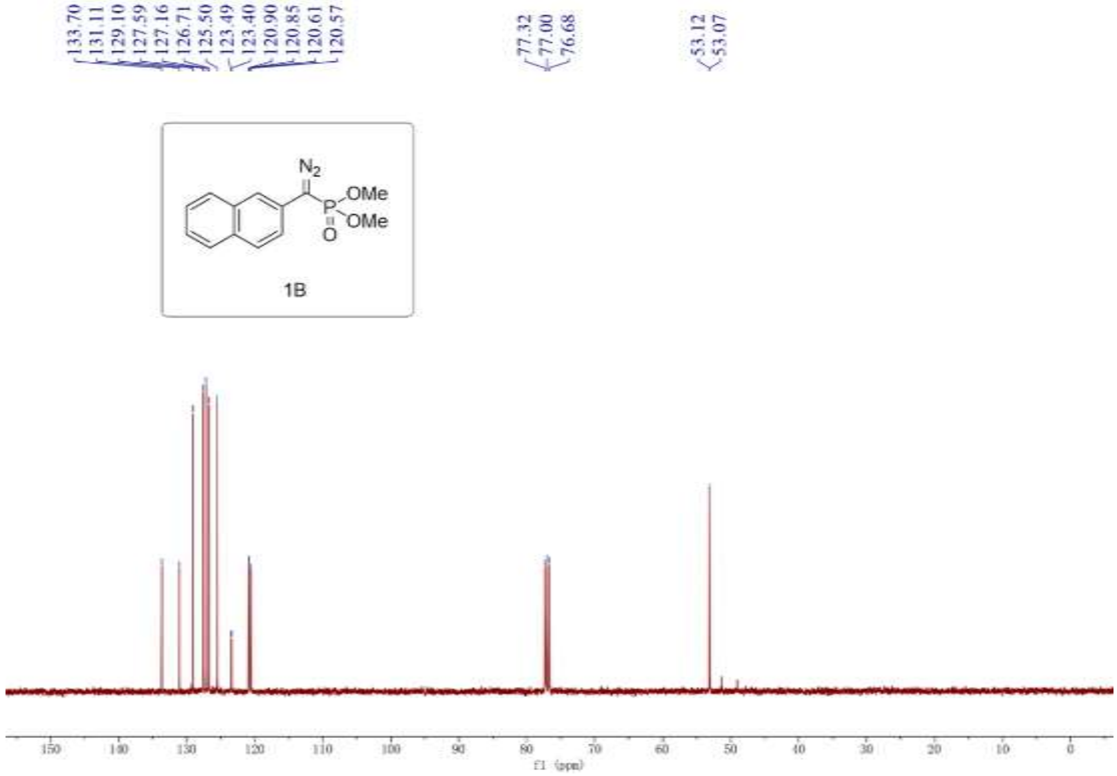

^{31}P NMR (162 MHz, Chloroform-*d*)

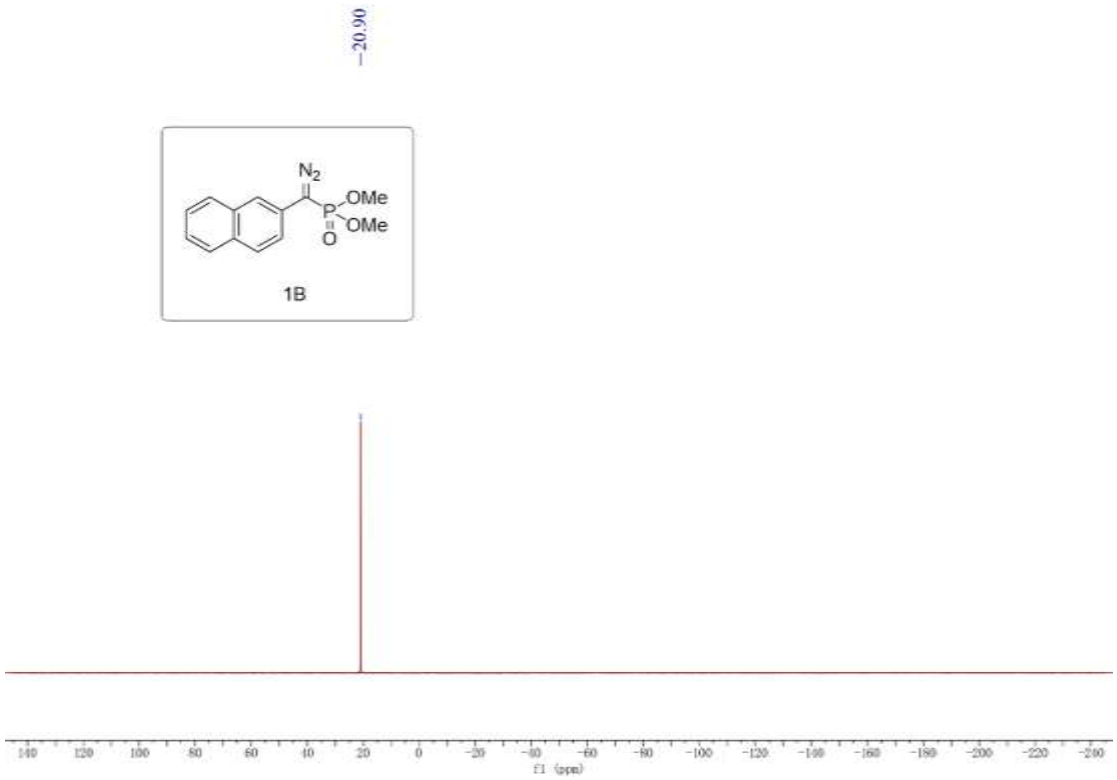

Dimethyl (diazo(naphthalen-1-yl)methyl)phosphonate (1C)

^{1}H NMR (500 MHz, Chloroform-*d*)

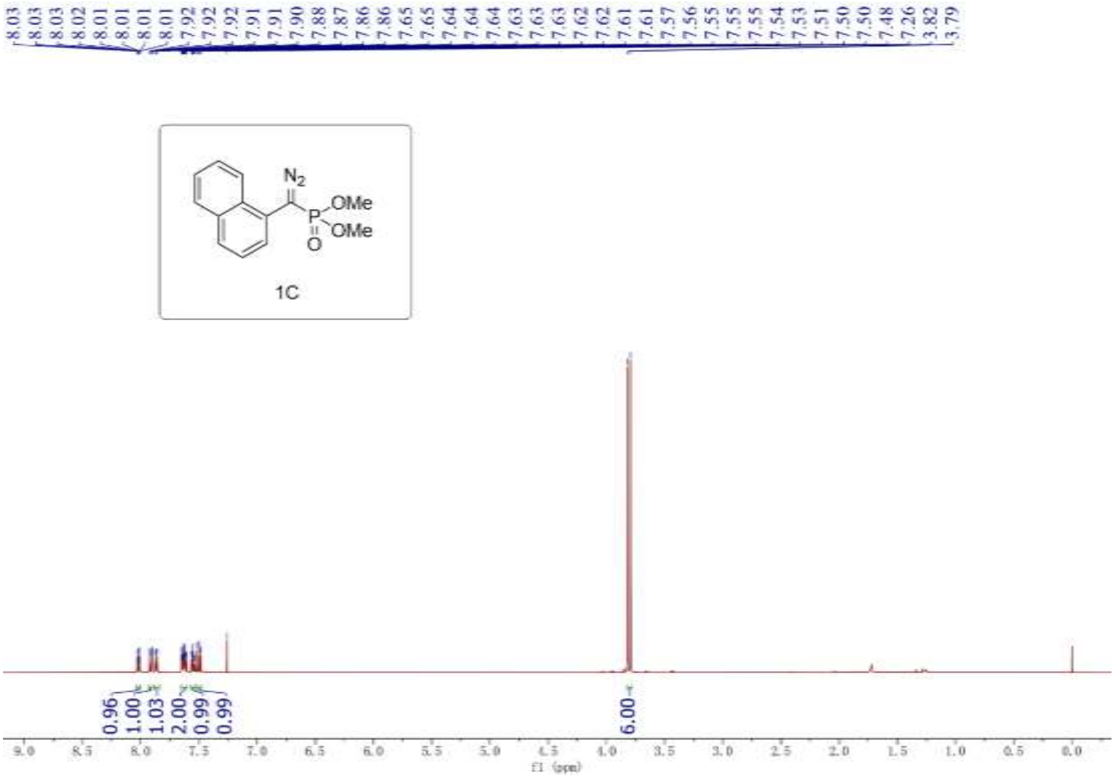

Diethyl (diazo(phenyl)methyl)phosphonate (4a)

^{1}H NMR (500 MHz, Chloroform-*d*)

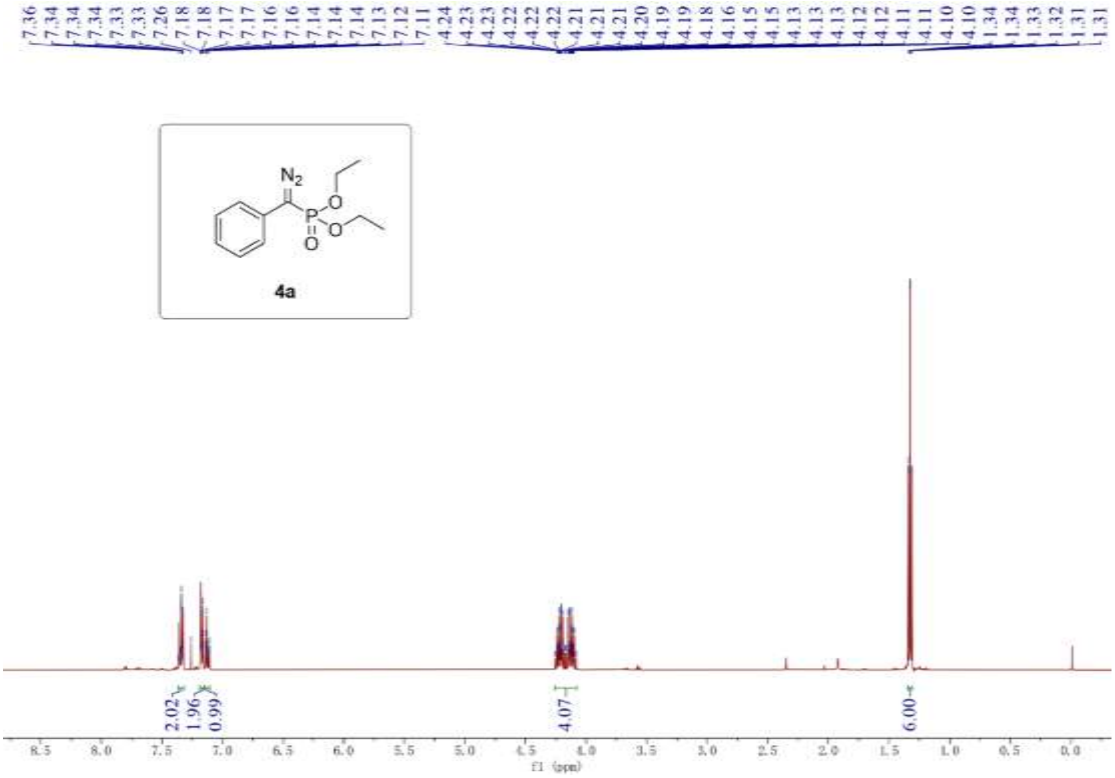

Diisopropyl (diazo(phenyl)methyl)phosphonate (4b)

^{1}H NMR (500 MHz, Chloroform-*d*)

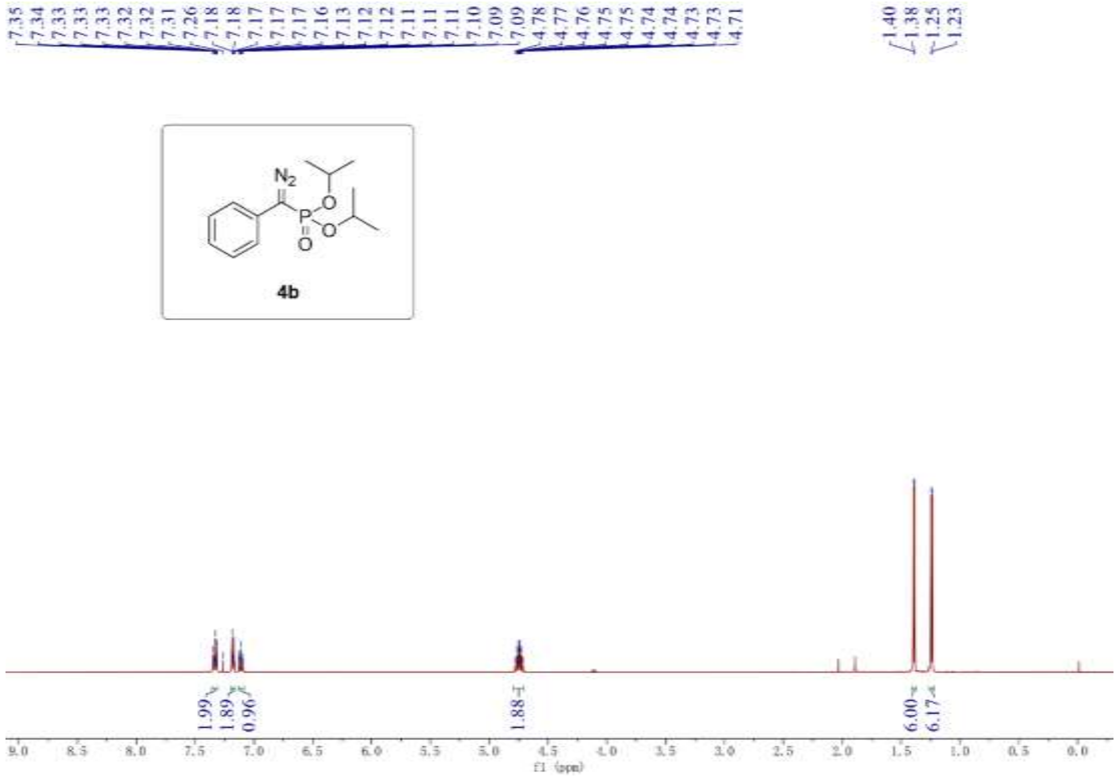

Dihexyl (diazo(phenyl)methyl)phosphonate (4c)

^{1}H NMR (500 MHz, Chloroform-*d*)

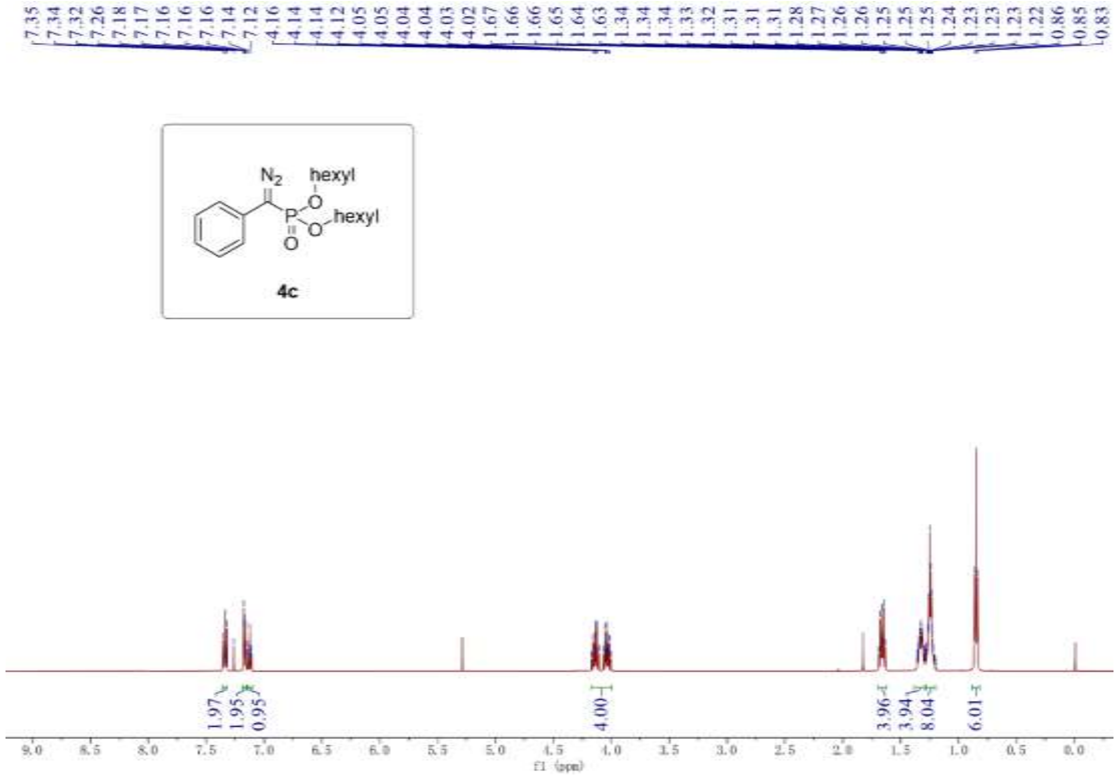

^{13}C NMR (126 MHz, Chloroform-*d*)

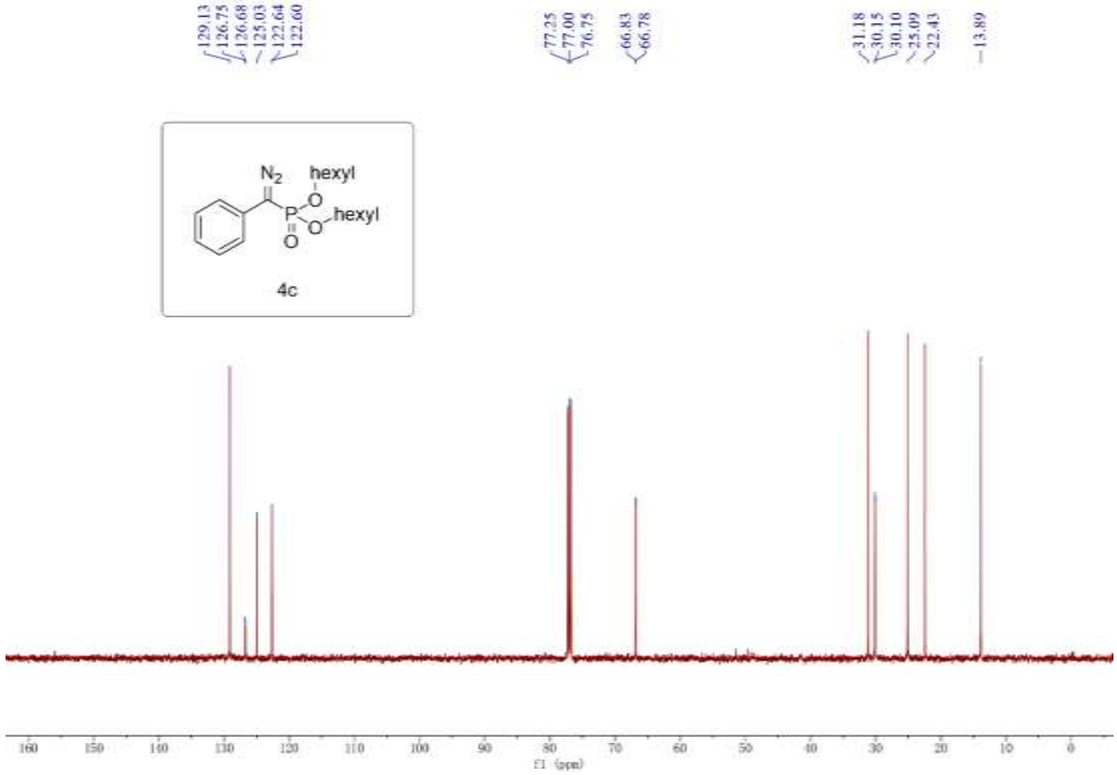

^{31}P NMR (202 MHz, Chloroform-*d*)

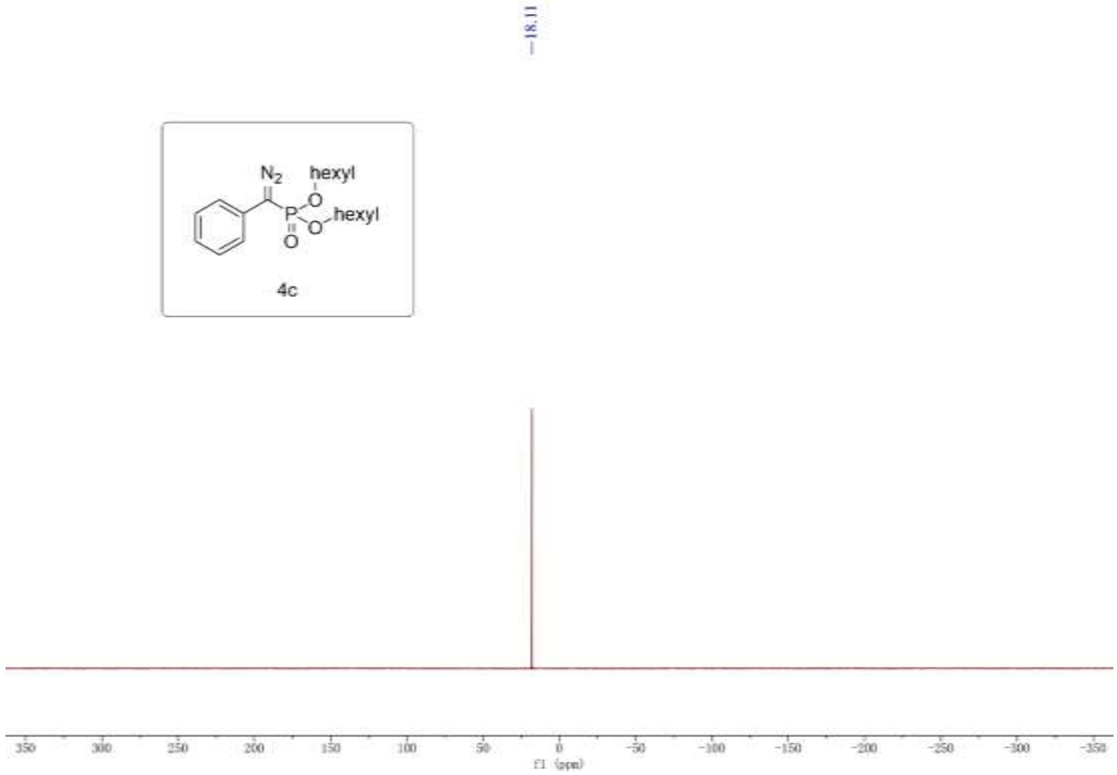

Methyldiphenylphosphane borane (2a)

^{1}H NMR (500 MHz, Chloroform-*d*)

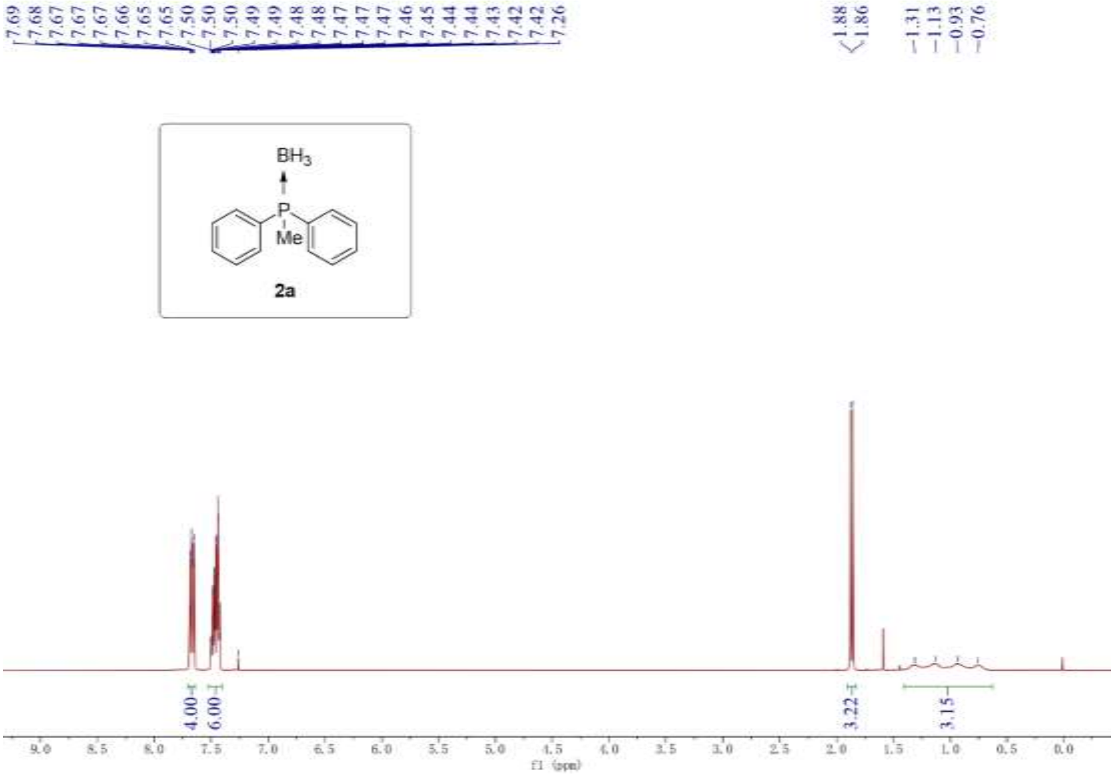

Ethyldiphenylphosphane borane (2b)

^{1}H NMR (400 MHz, Chloroform-*d*)

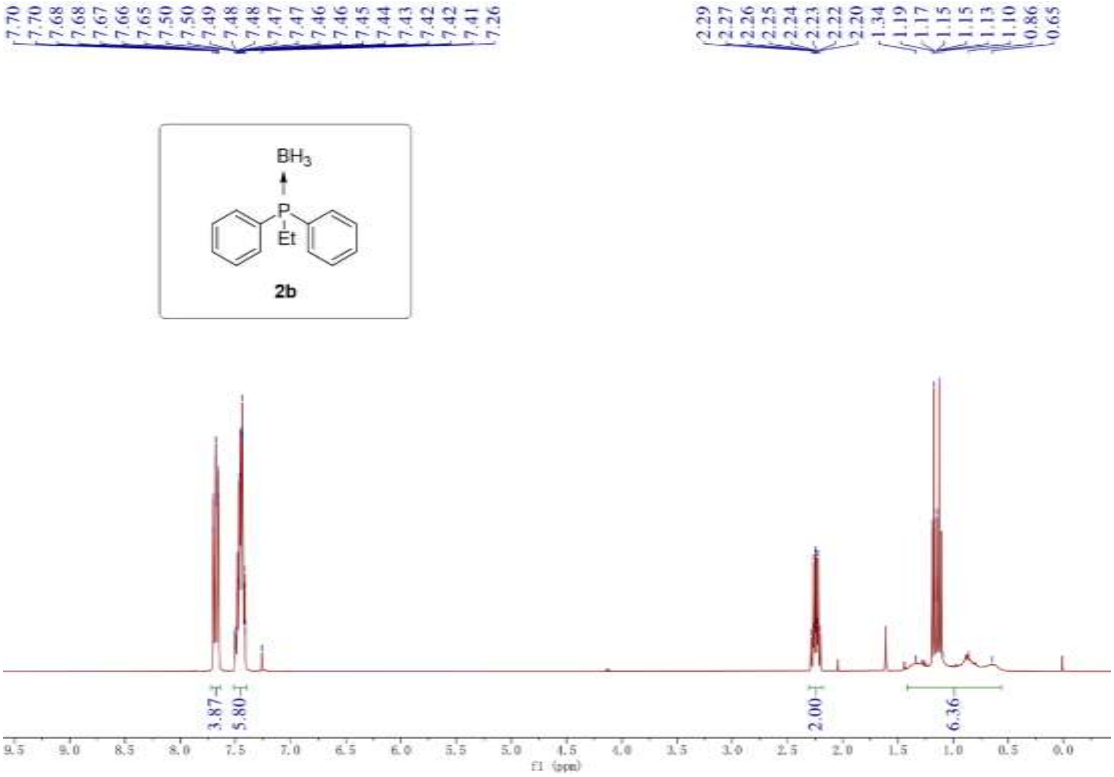

Isopropyldiphenylphosphane borane (2c)

^{1}H NMR (400 MHz, Chloroform-*d*)

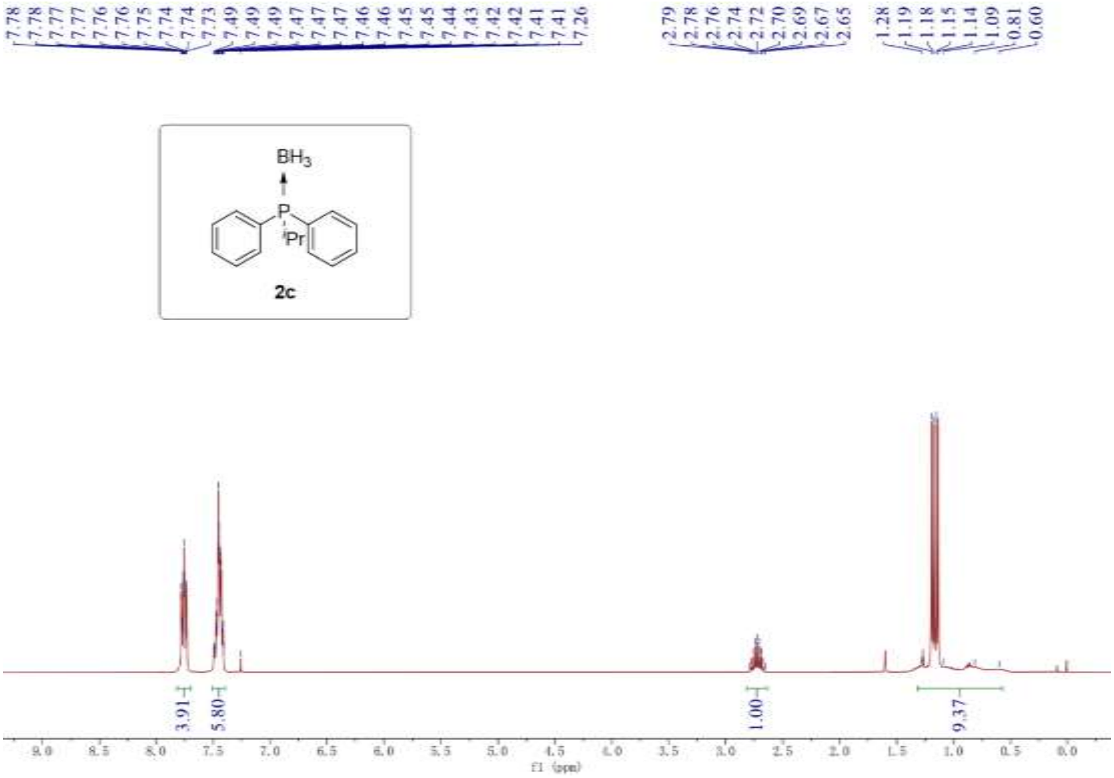

Dimethyl(phenyl)phosphane borane (2d)

^{1}H NMR (500 MHz, Chloroform-*d*)

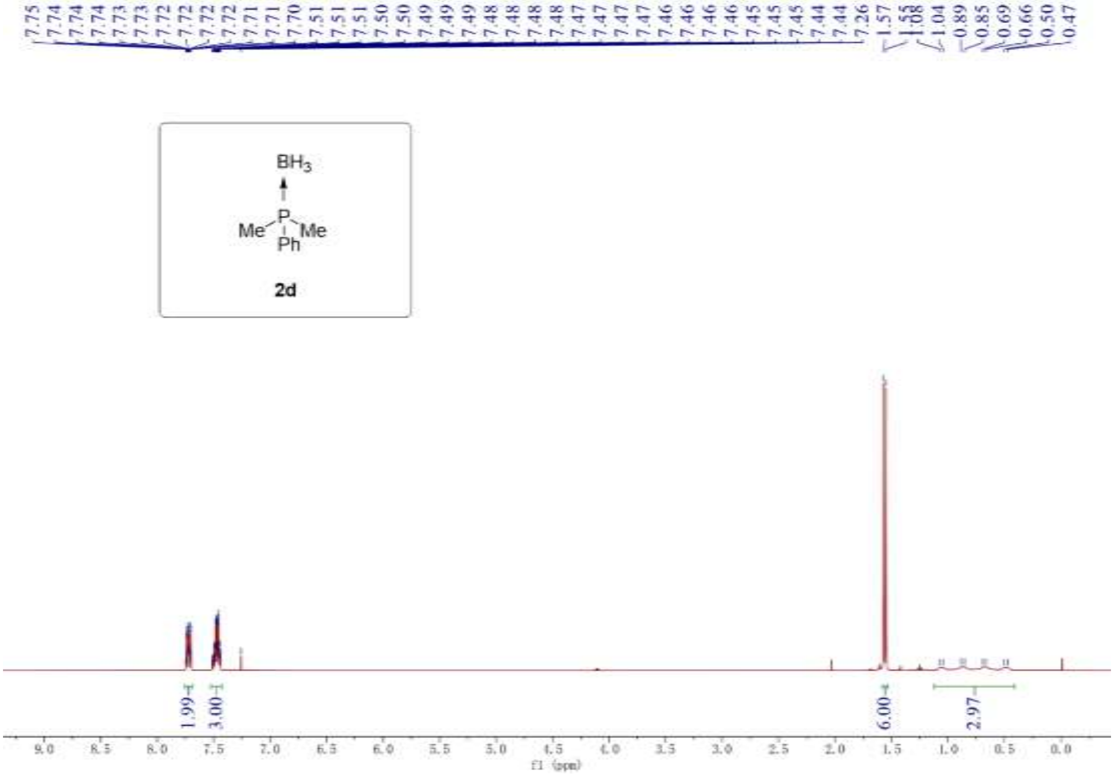

Diethyl(phenyl)phosphane borane (2e)

^{1}H NMR (500 MHz, Chloroform-*d*)

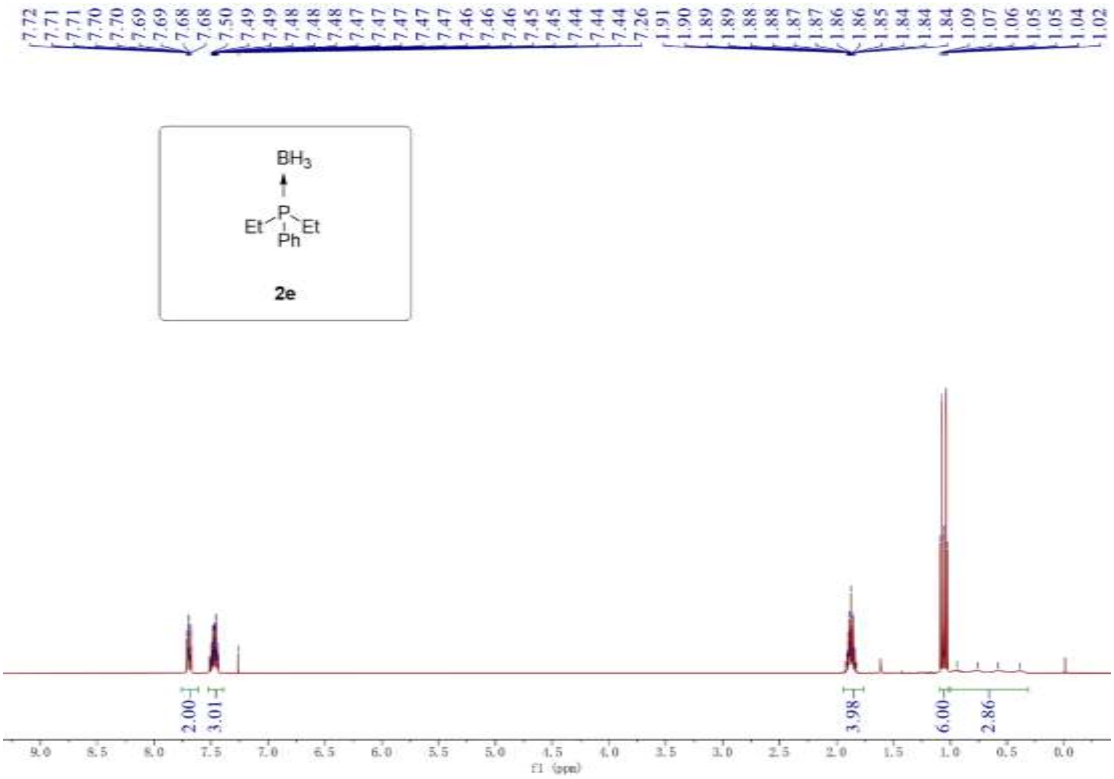

Dicyclohexyl(phenyl)phosphane borane (2f)

^{1}H NMR (400 MHz, Chloroform-*d*)

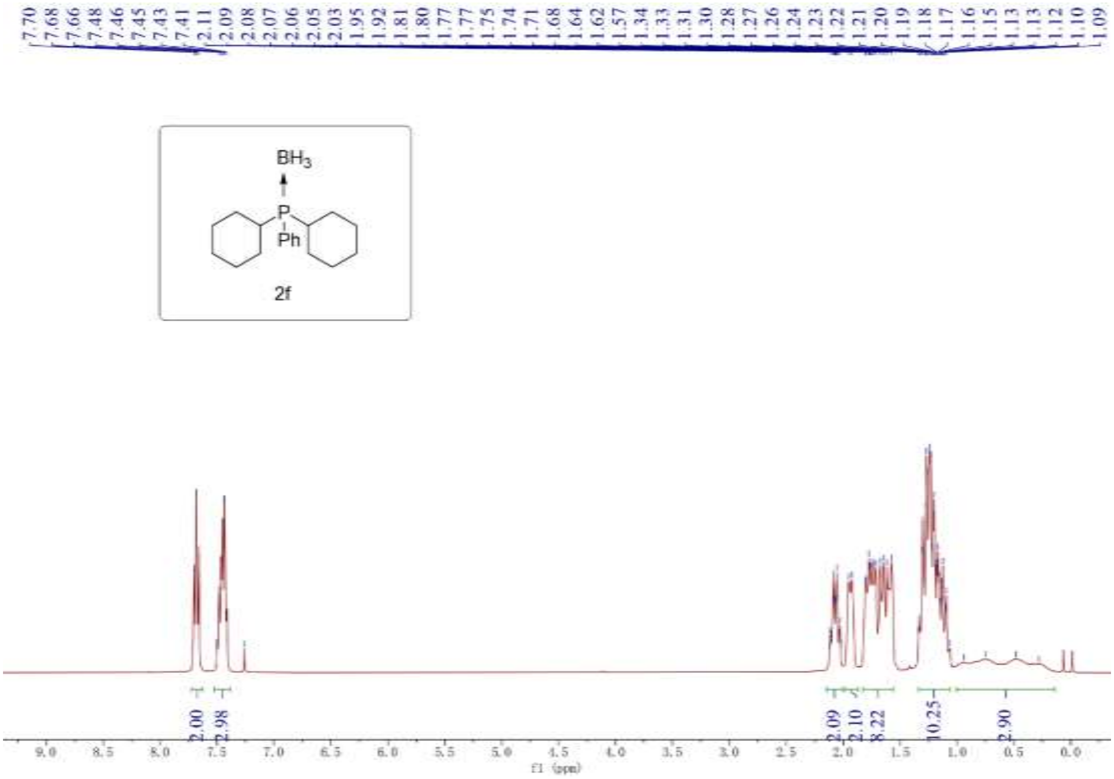

Trimethylphosphane borane (2g)

^{1}H NMR (500 MHz, Chloroform-*d*)

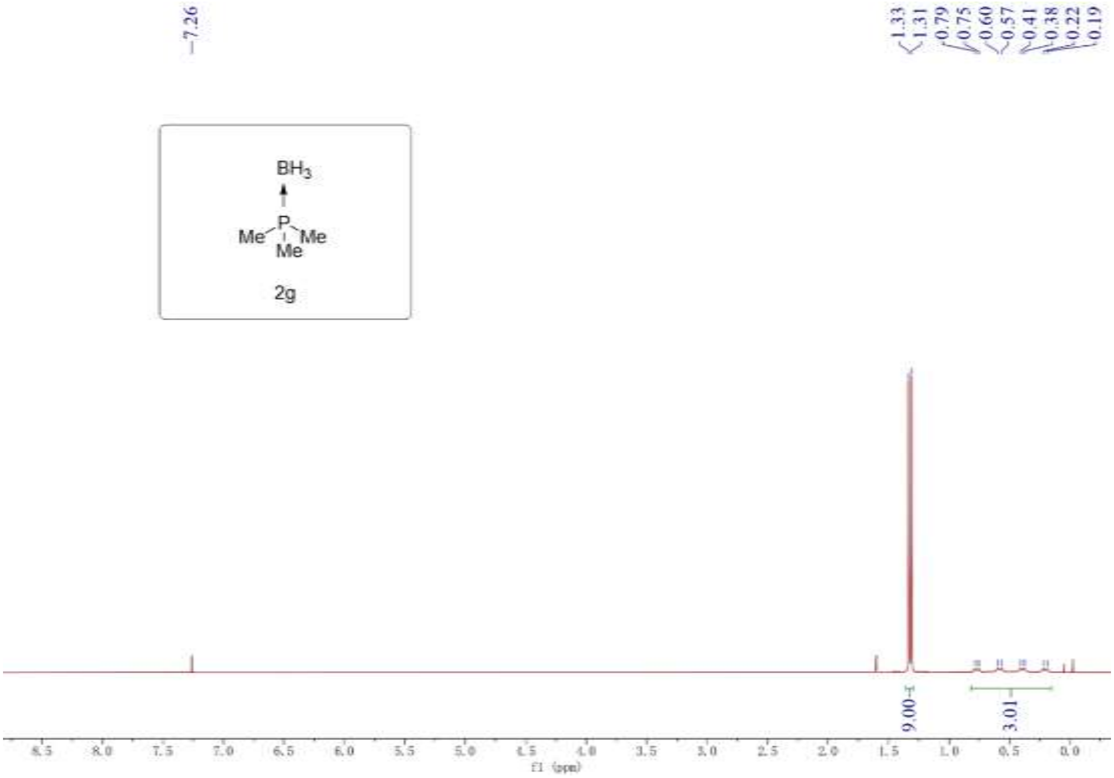

Tributylphosphane borane (2h)

^{1}H NMR (500 MHz, Chloroform-*d*)

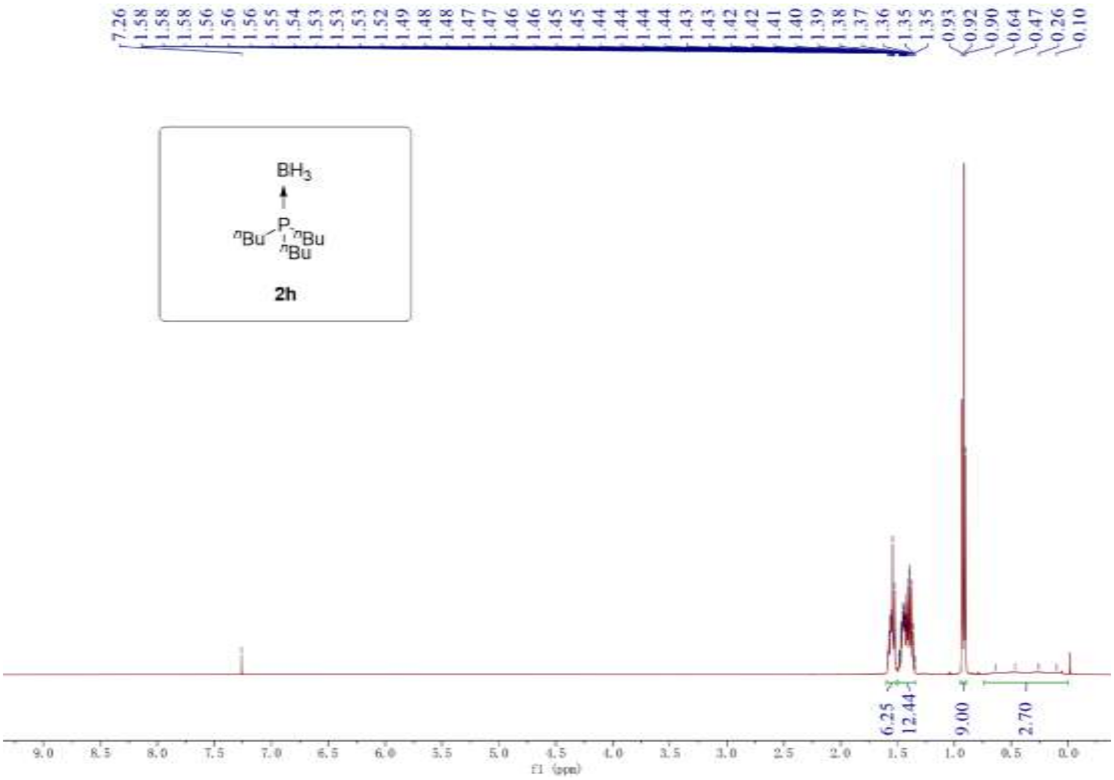

Trimethylphosphite borane (2i)

^{1}H NMR (500 MHz, Chloroform-*d*)

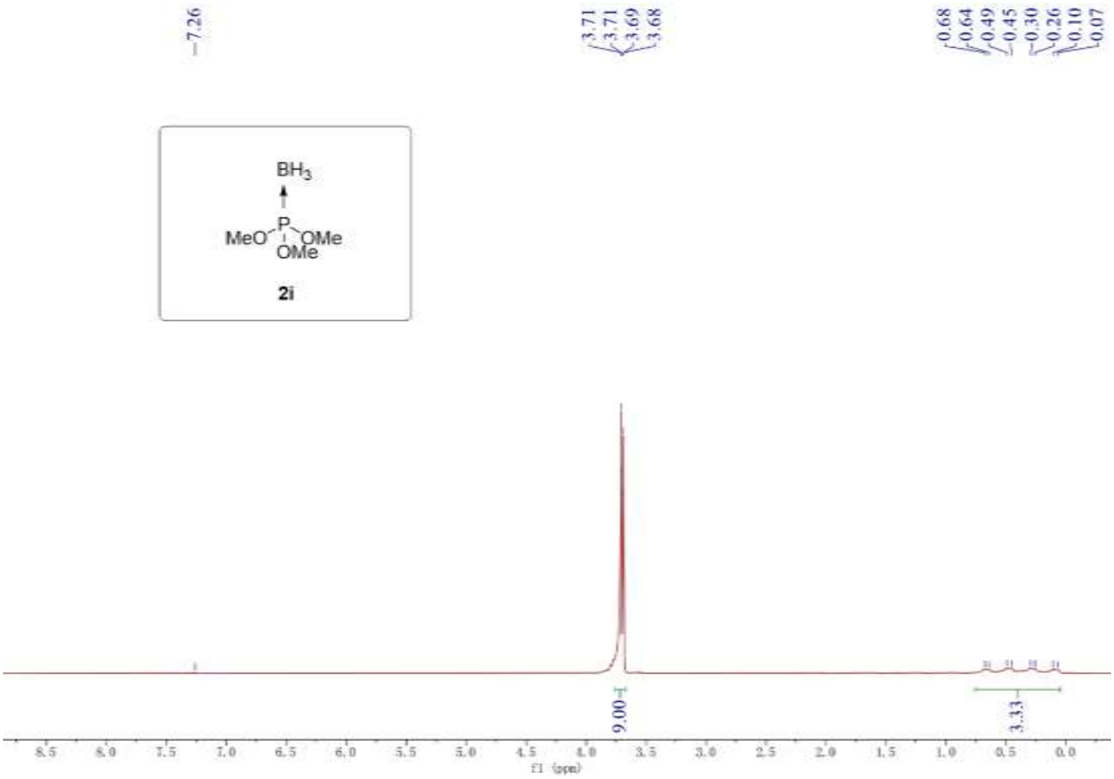

(S)-dimethyl(((methyldiphenylphosphane)boryl)(phenyl)methyl)phosphonate(3aa)

^{1}H NMR (500 MHz, Chloroform-*d*)

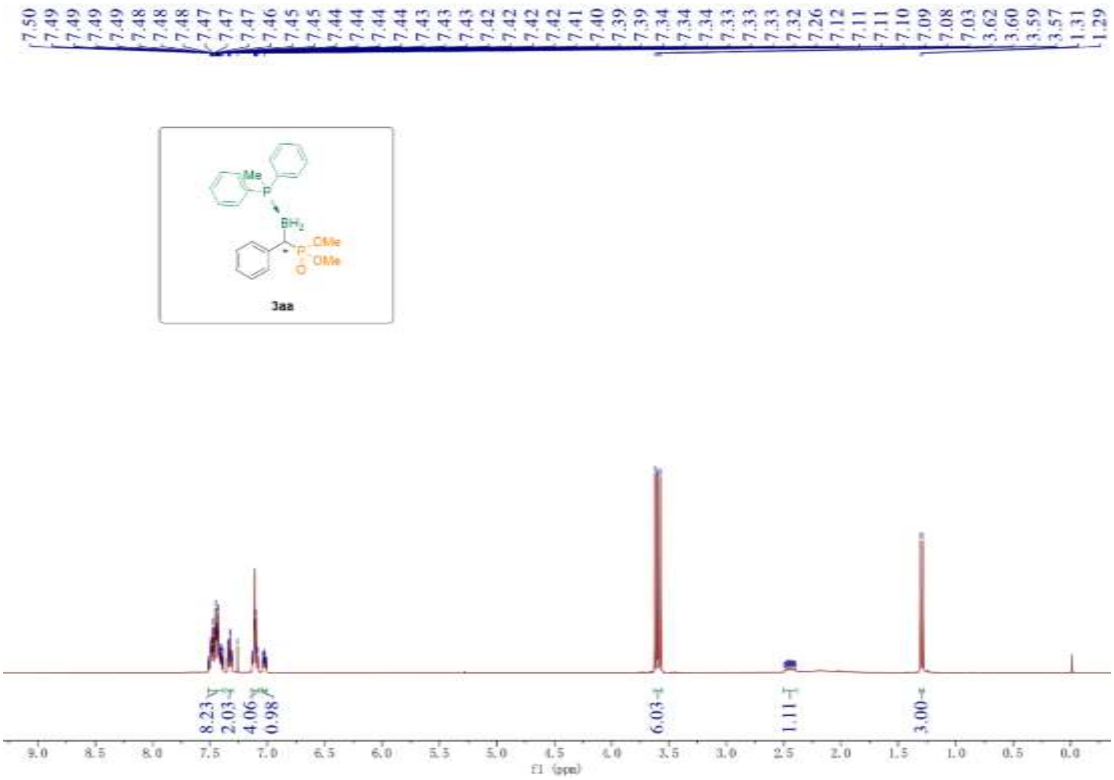

^{13}C NMR (126 MHz, Chloroform-*d*)

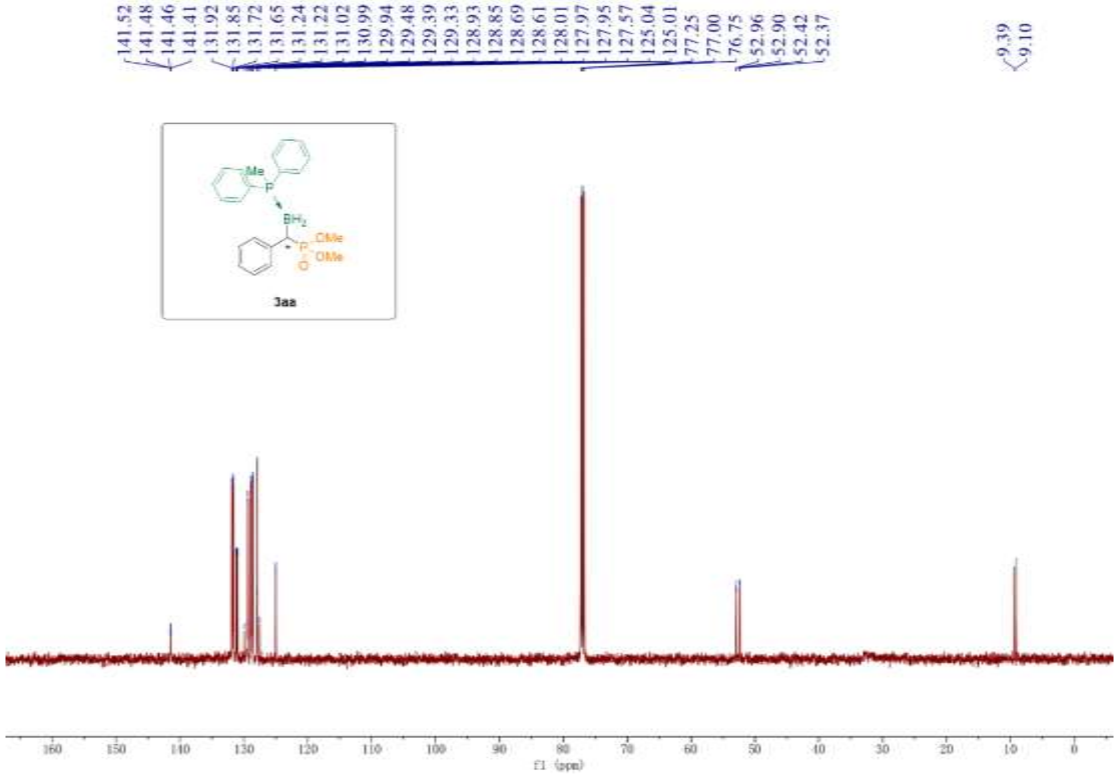

^{11}B NMR (160 MHz, Chloroform-*d*)

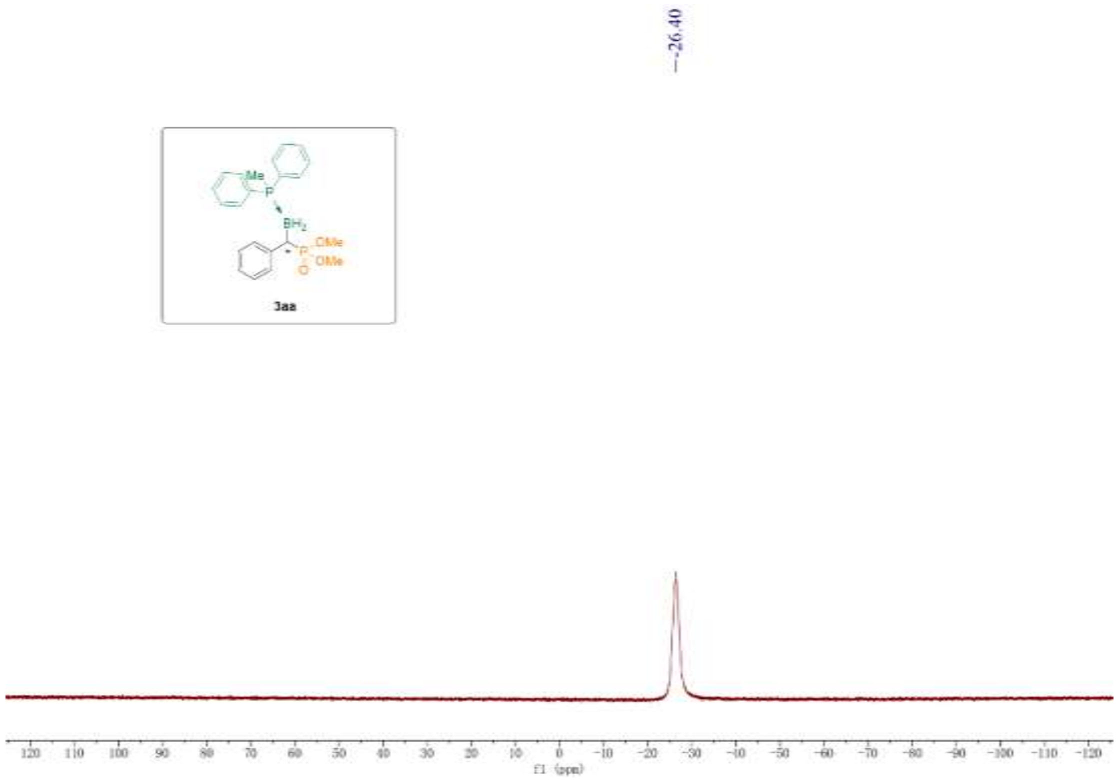

^{31}P NMR (202 MHz, Chloroform-*d*)

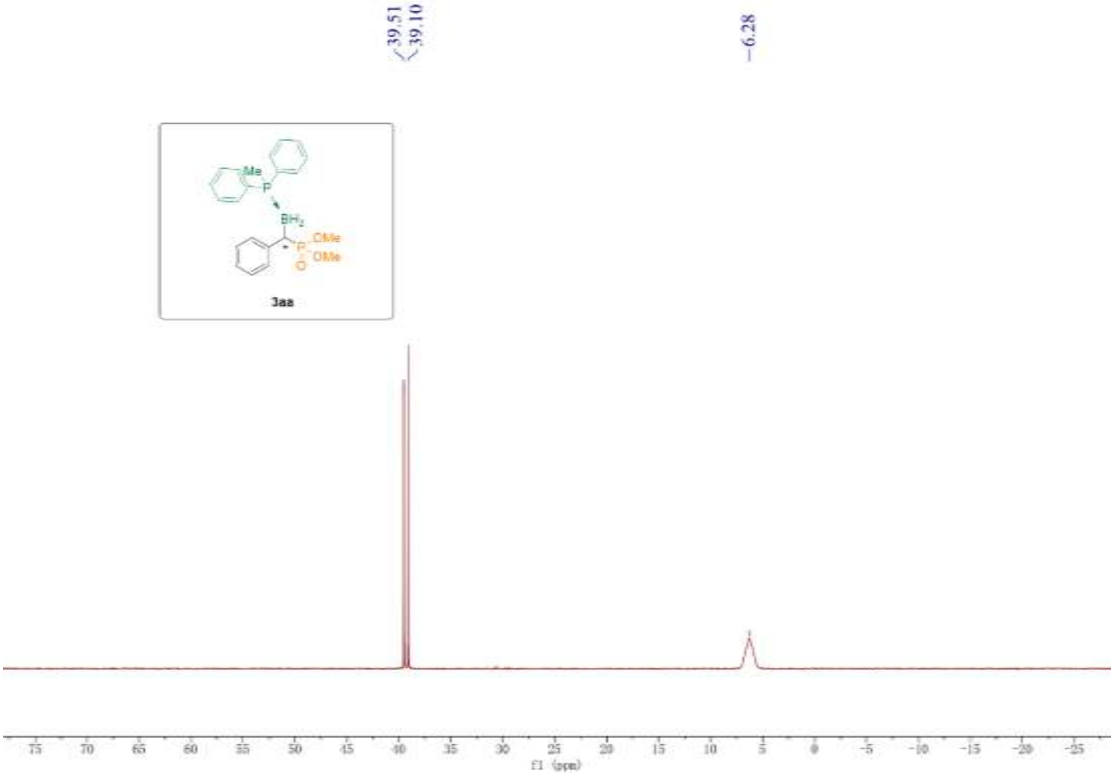

(S)-dimethyl(((methyldiphenylphosphane)boryl)(4-chlorophenyl)methyl)phosphonate(3ba)

^{1}H NMR (500 MHz, Chloroform-*d*)

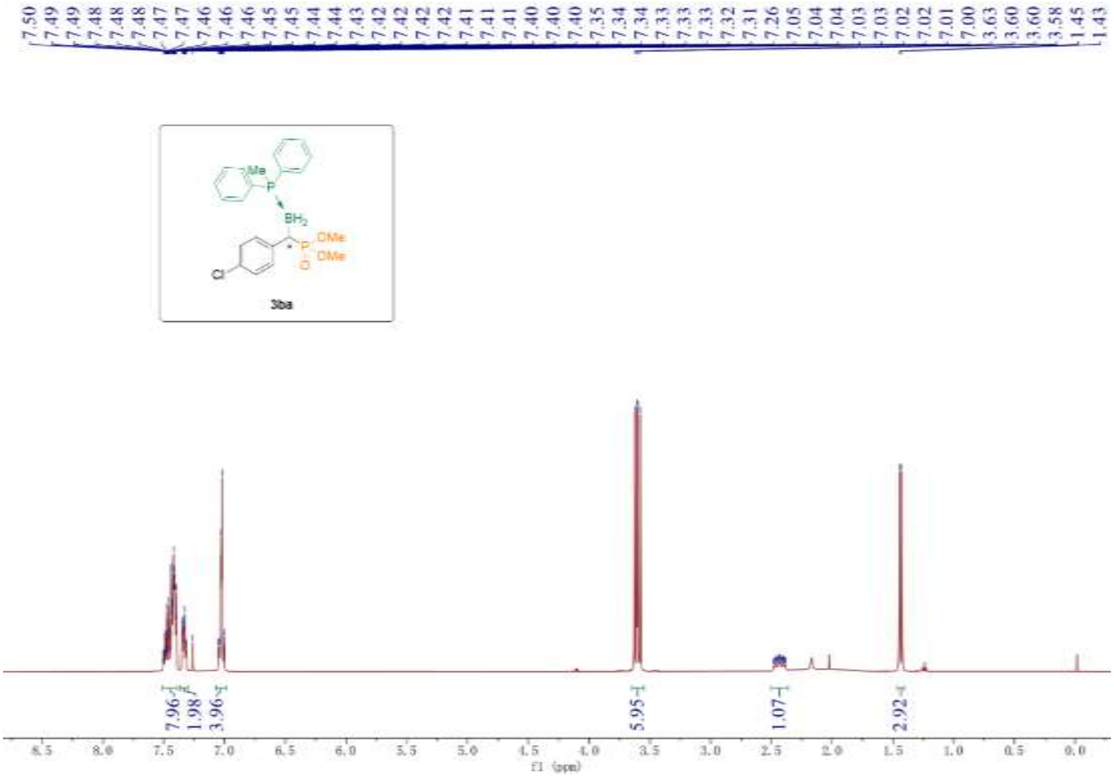

^{13}C NMR (126 MHz, Chloroform-*d*)

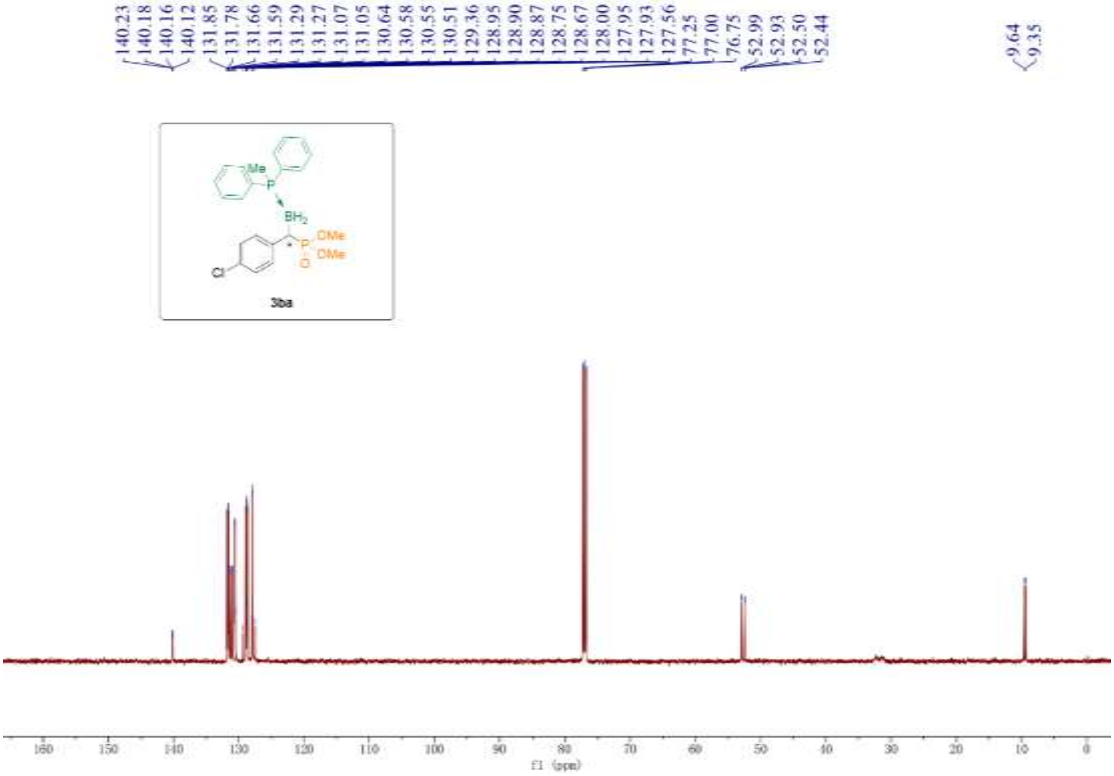

^{11}B NMR (160 MHz, Chloroform-*d*)

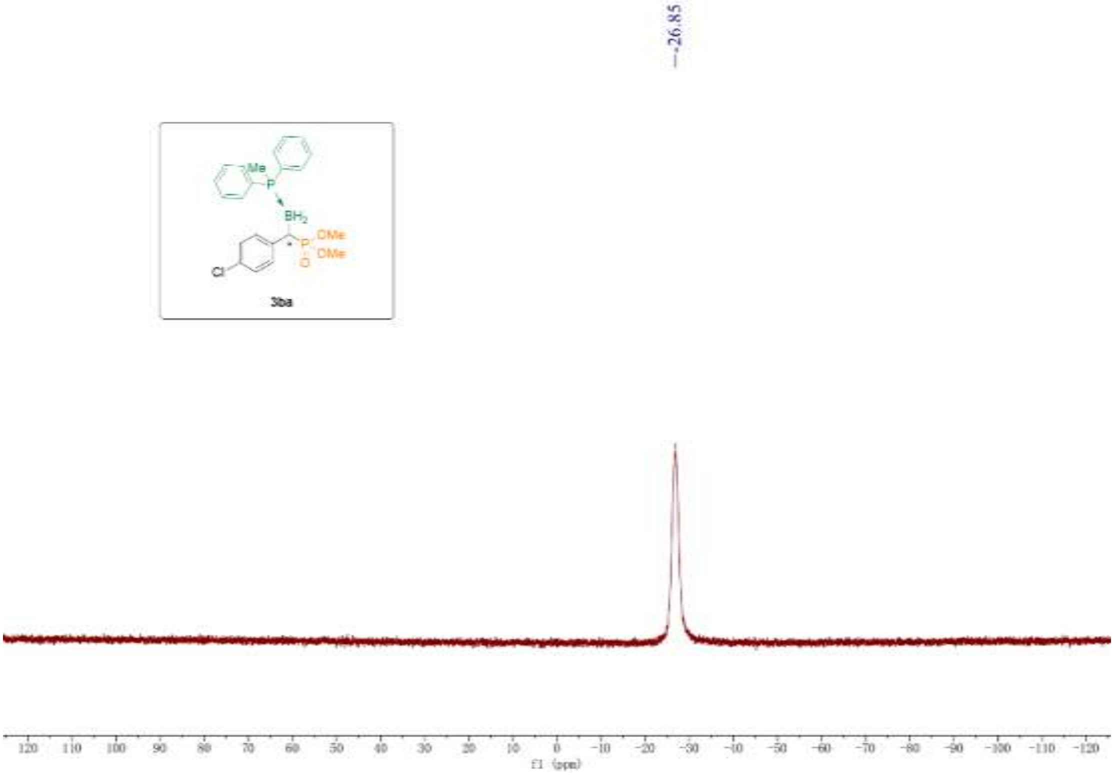

^{31}P NMR (202 MHz, Chloroform-*d*)

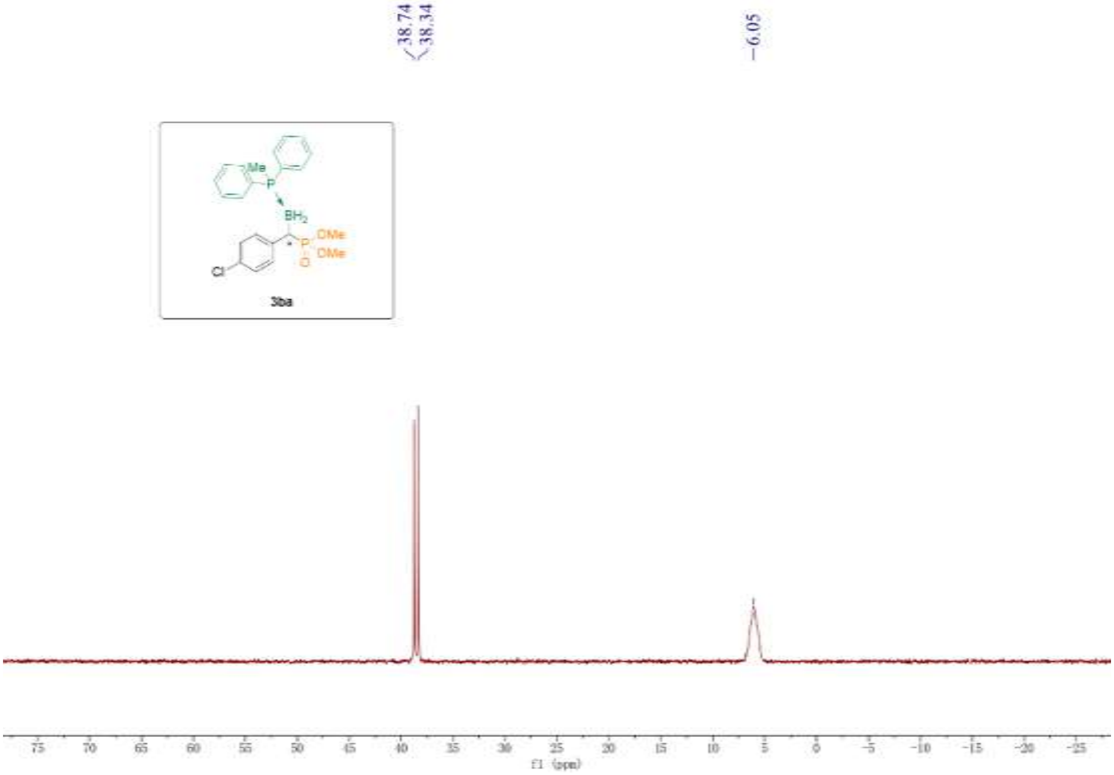

(S)-dimethyl(((methyldiphenylphosphane)boryl)(4-(hydroxymethyl)phenyl)methyl) phosphonate(3ca)

^{1}H NMR (400 MHz, Chloroform-*d*)

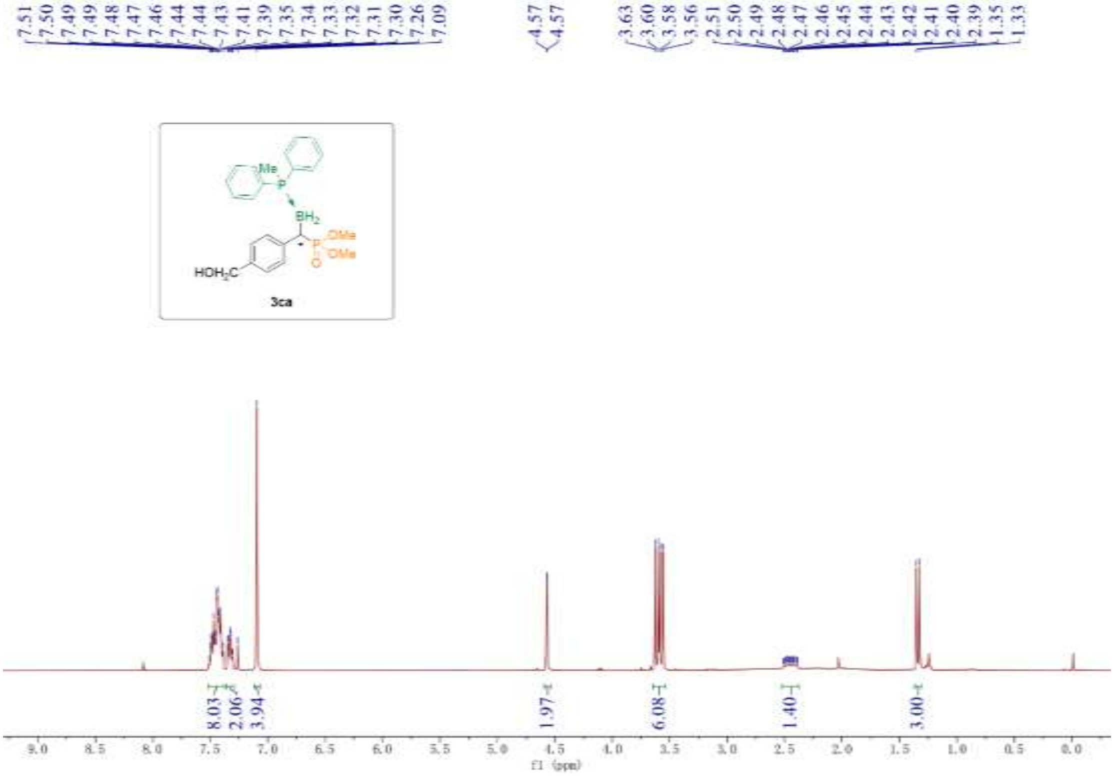

^{13}C NMR (126 MHz, Chloroform-*d*)

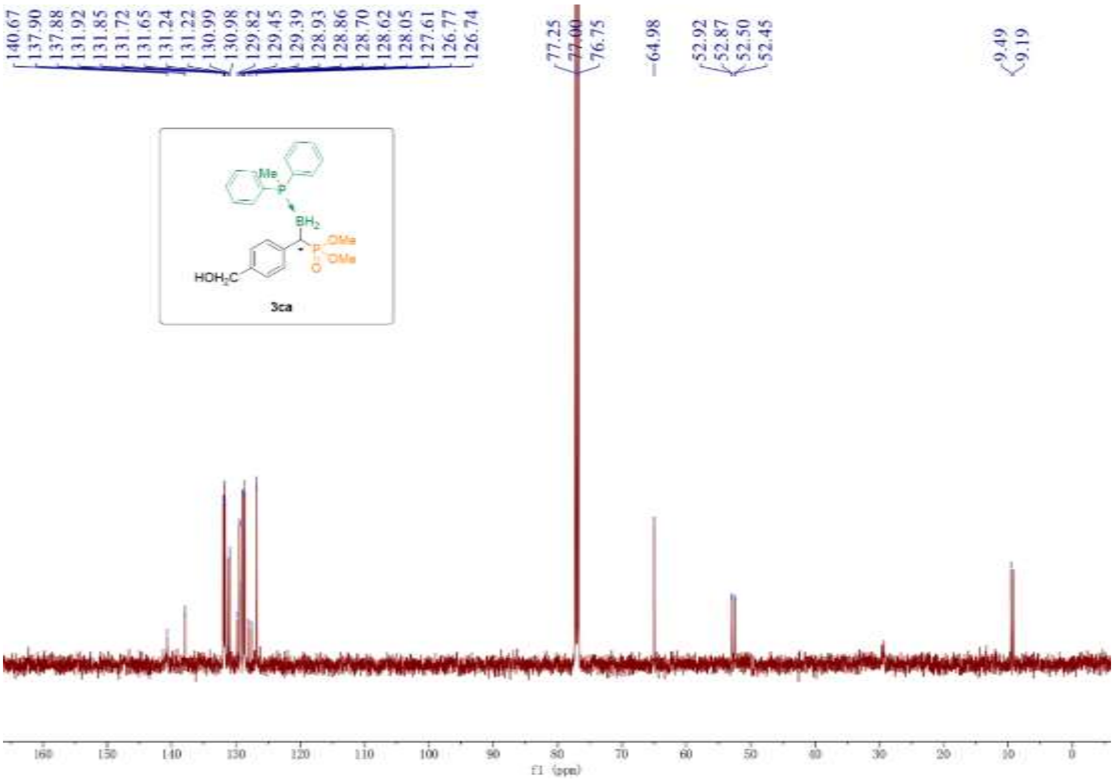

^{11}B NMR (128 MHz, Chloroform-*d*)

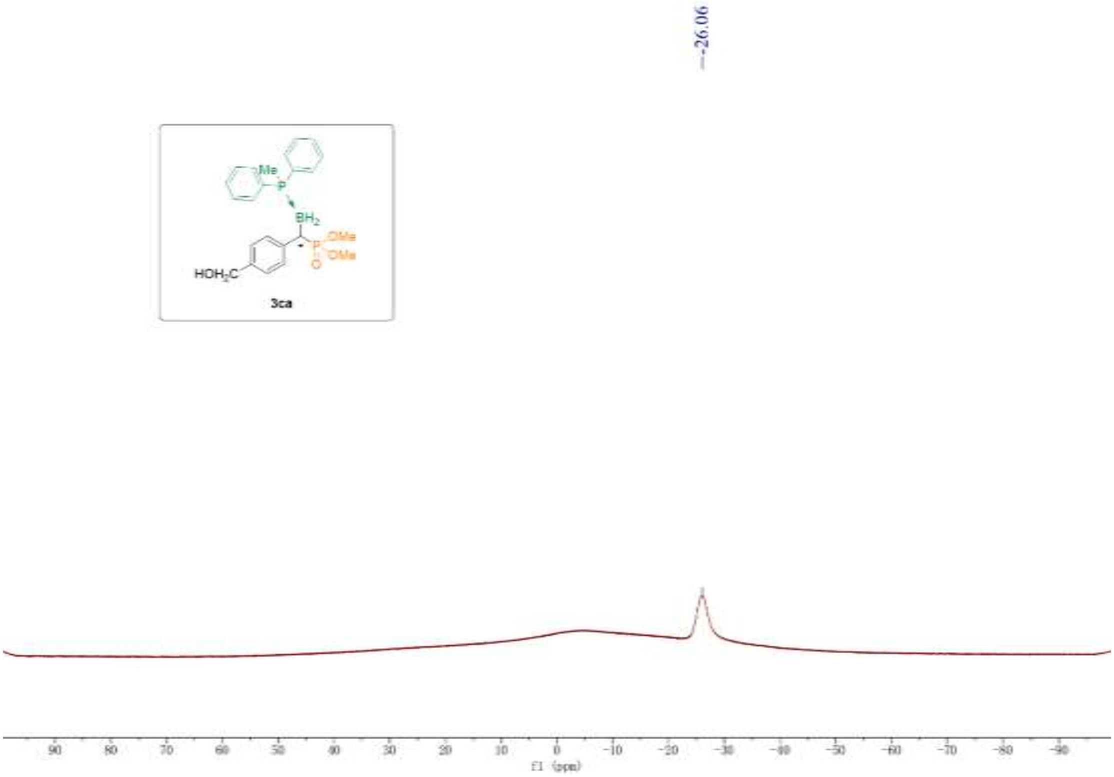

^{31}P NMR (162 MHz, Chloroform-*d*)

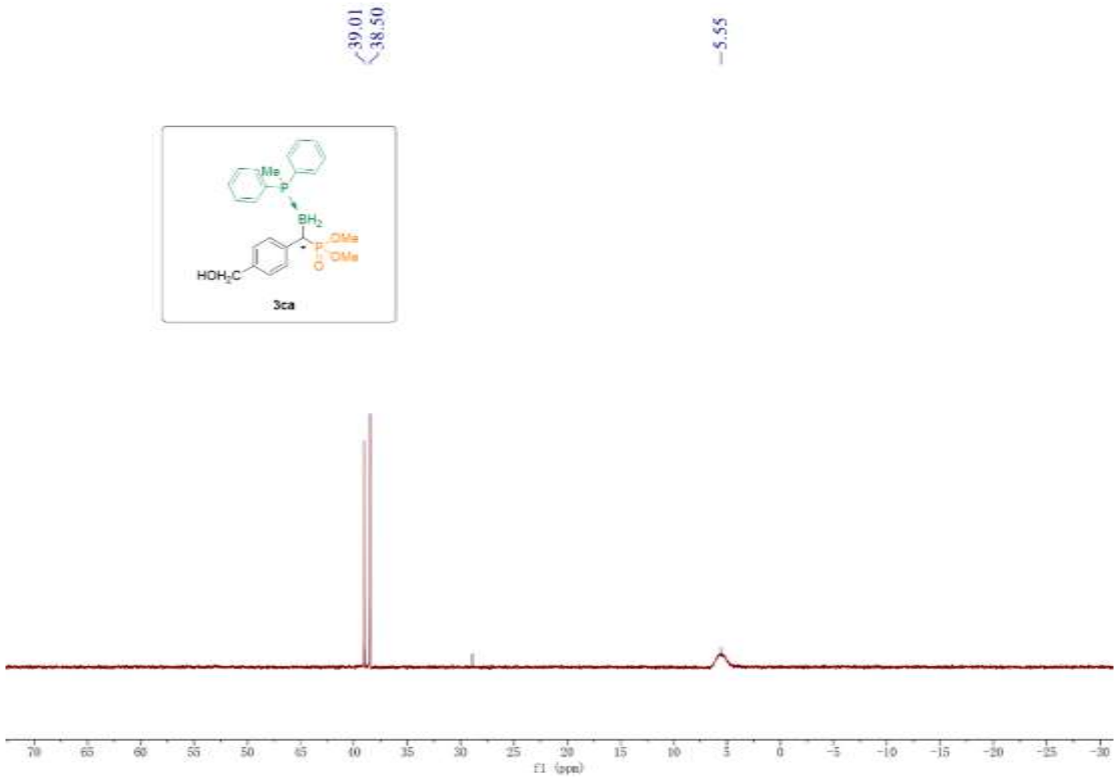

(S)-dimethyl(((methyldiphenylphosphane)boryl)(4-methoxyphenyl)methyl)phosphonate(3da)

^{1}H NMR (400 MHz, Chloroform-*d*)

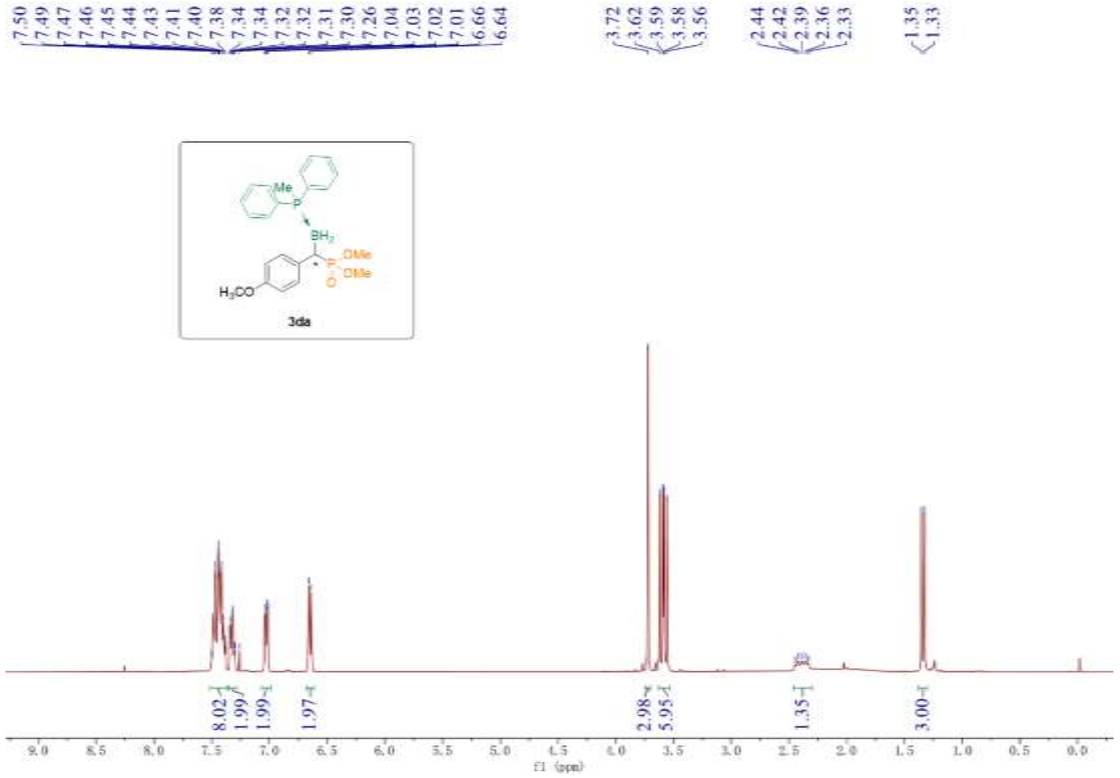

^{13}C NMR (101 MHz, Chloroform-*d*)

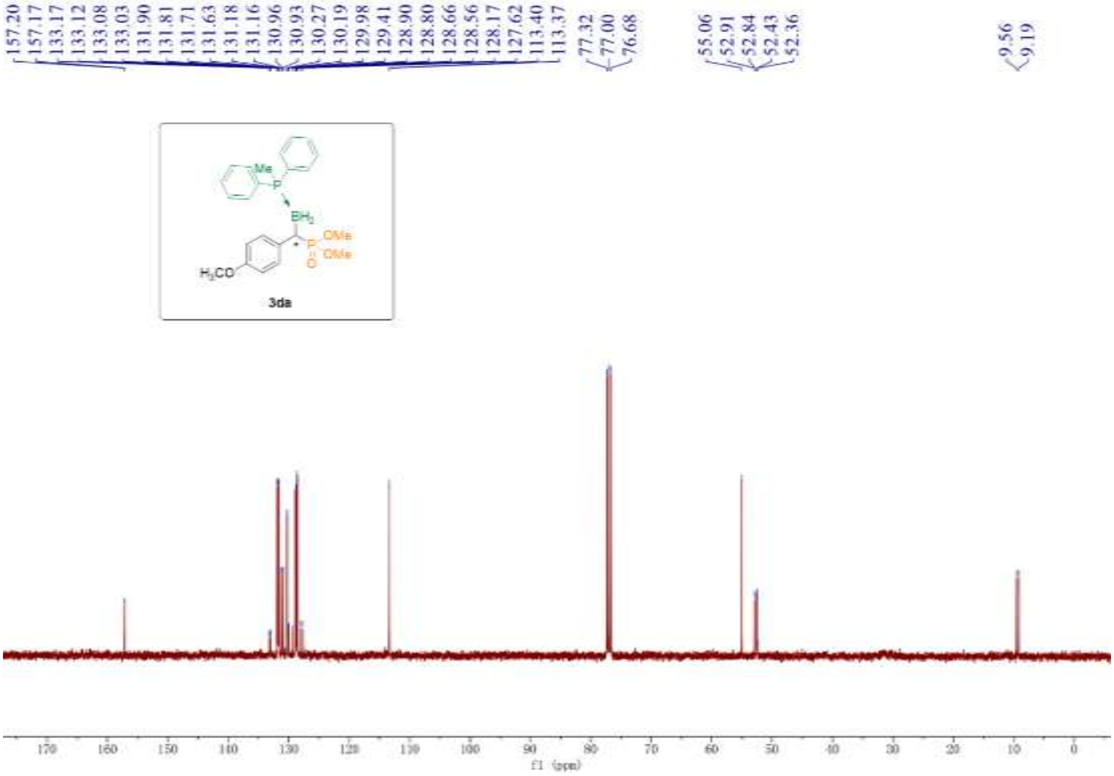

^{11}B NMR (128 MHz, Chloroform-*d*)

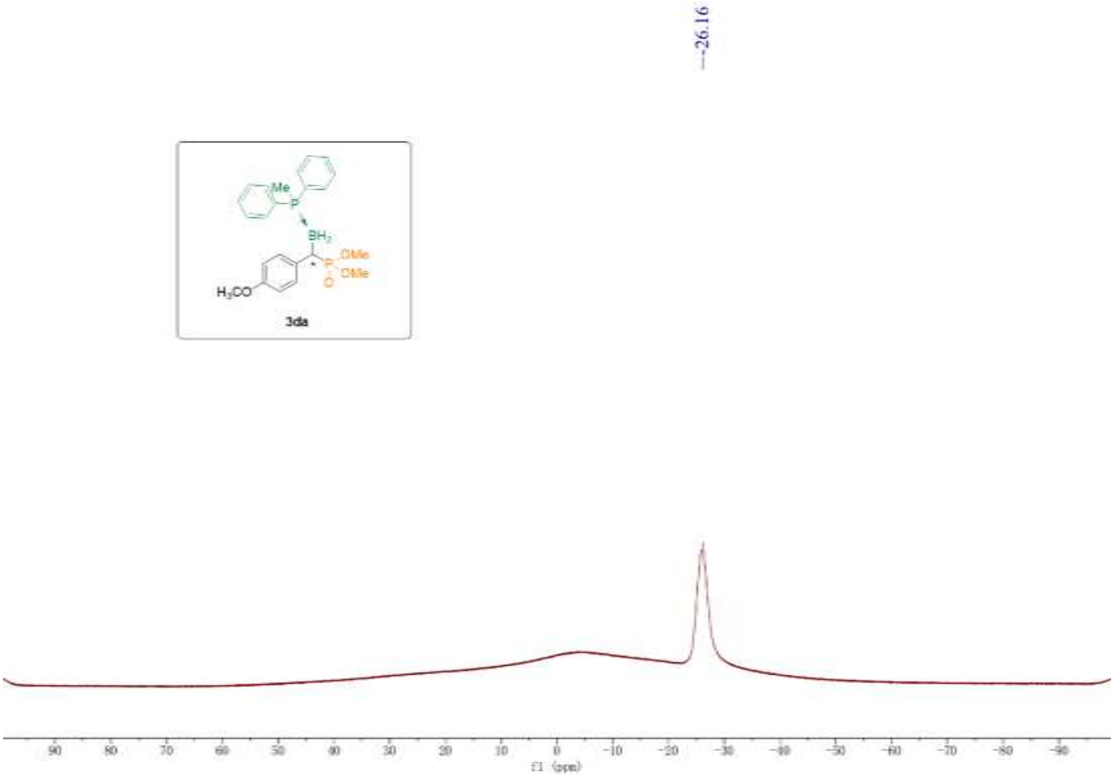

^{31}P NMR (162 MHz, Chloroform-*d*)

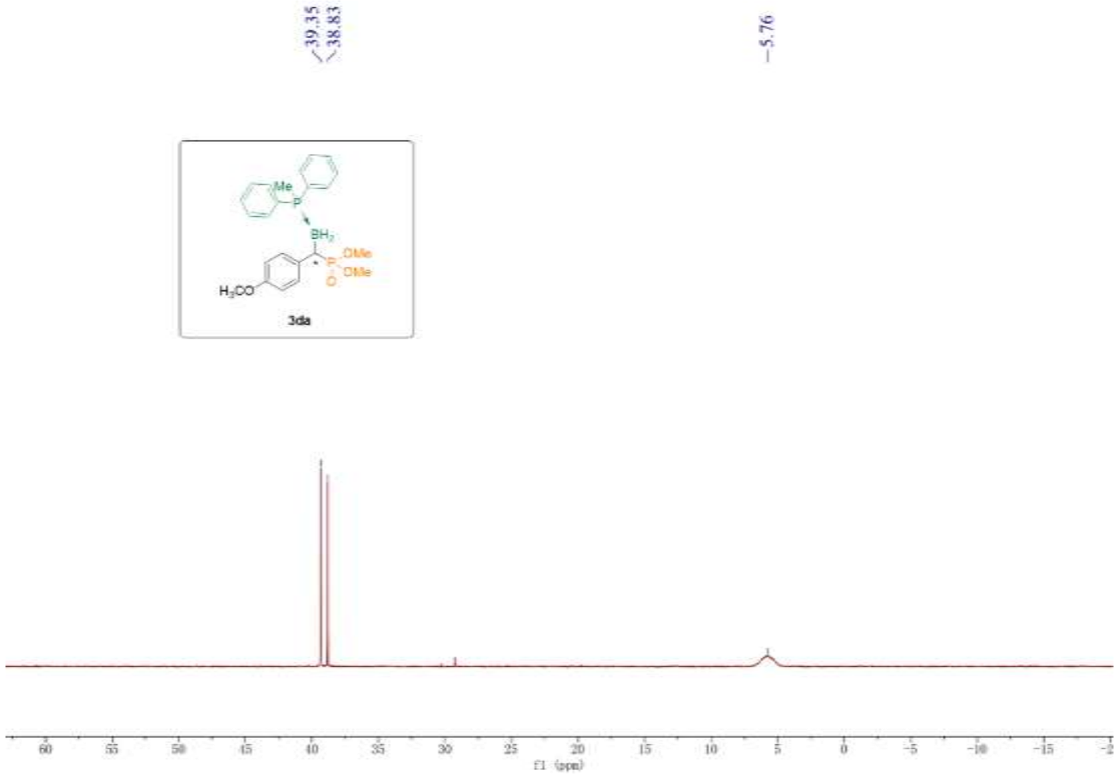

(S)-dimethyl(((methyldiphenylphosphane)boryl)(p-tolyl)methyl)phosphonate(3ea)

^{1}H NMR (500 MHz, Chloroform-*d*)

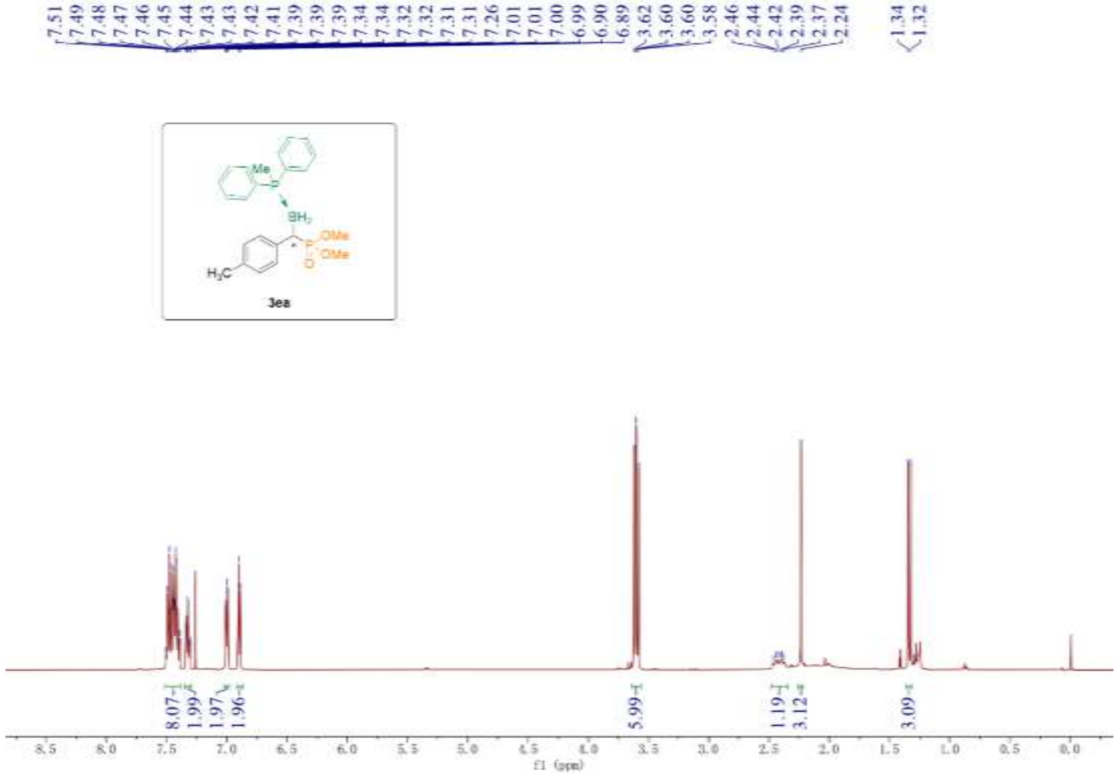

^{13}C NMR (126 MHz, Chloroform-*d*)

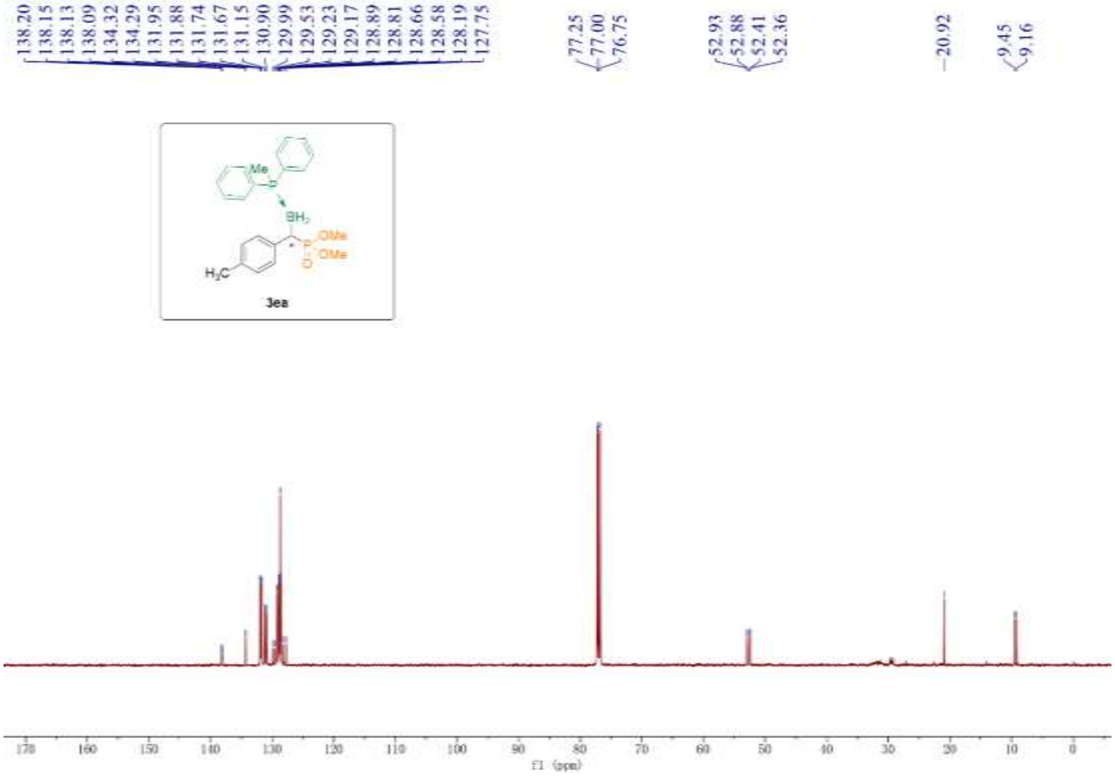

^{11}B NMR (128 MHz, Chloroform-*d*)

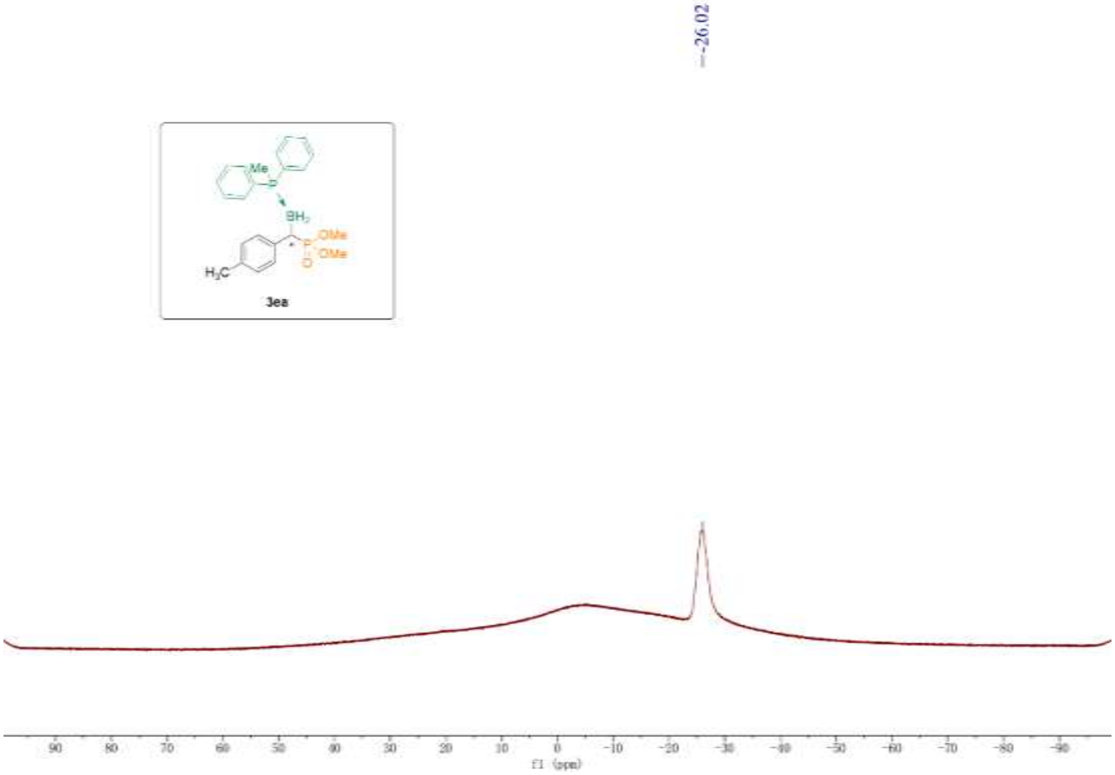

^{31}P NMR (202 MHz, Chloroform-*d*)

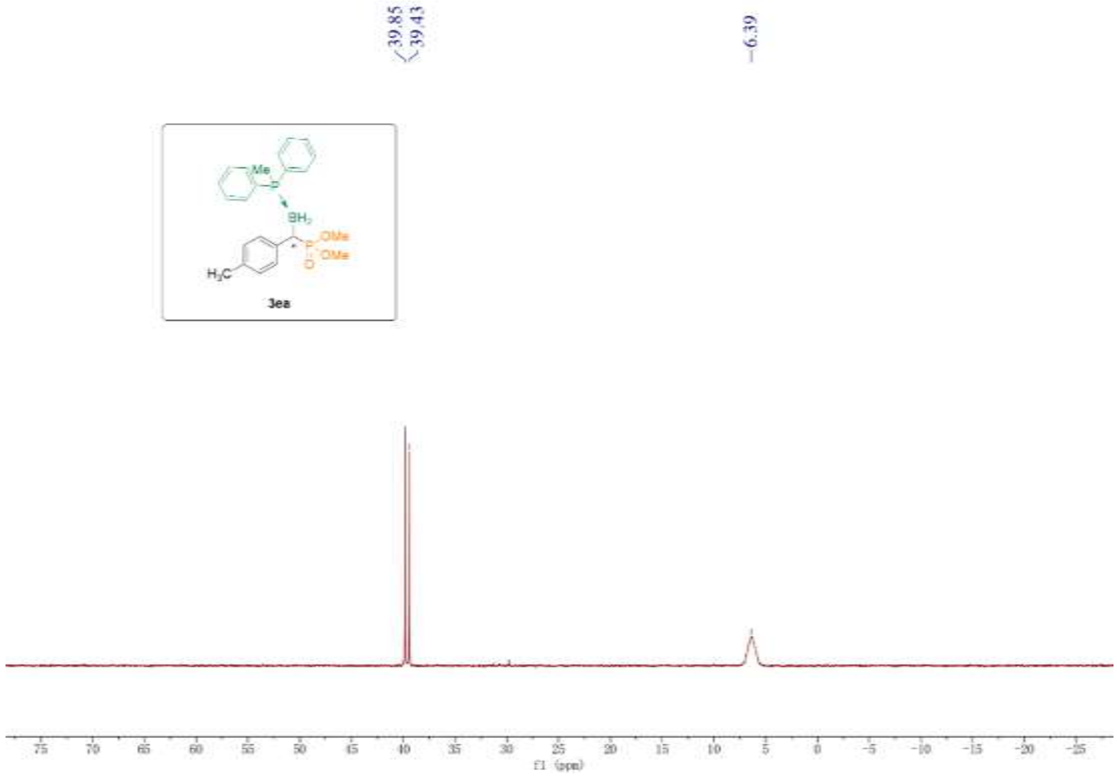

(S)-dimethyl(((methyldiphenylphosphane)boryl)(4-(methylthio))methyl)phosphonate(3fa)

^{1}H NMR (400 MHz, Chloroform-*d*)

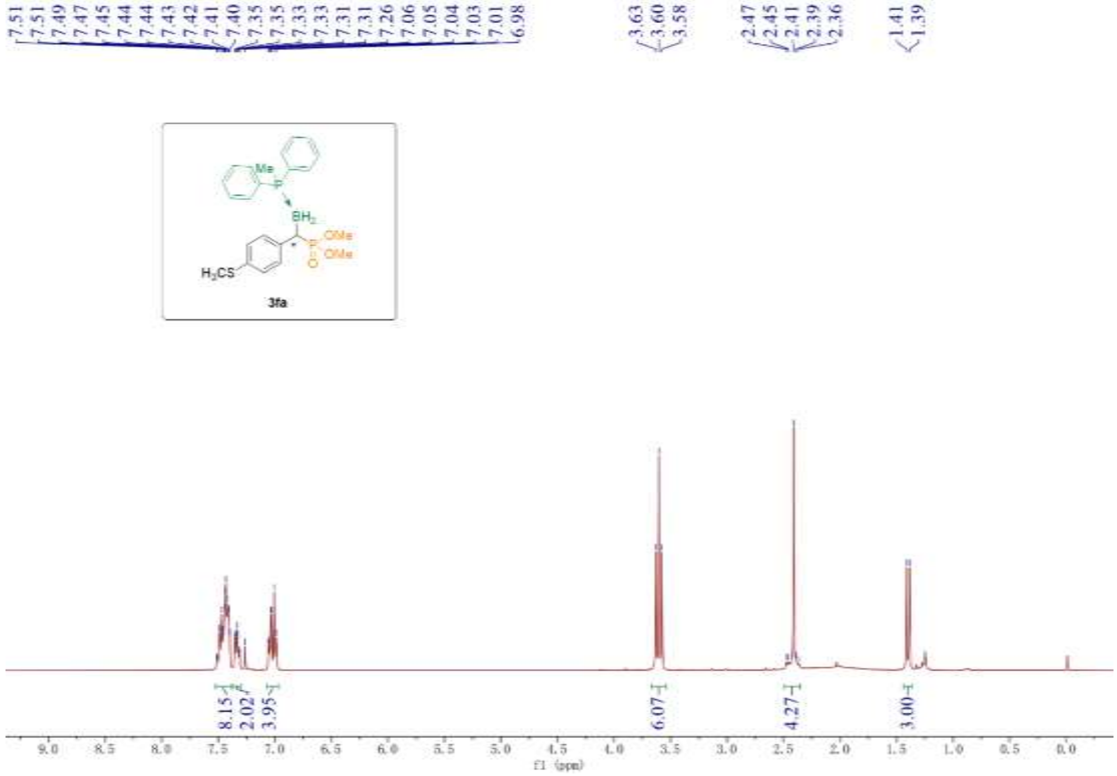

^{13}C NMR (101 MHz, Chloroform-*d*)

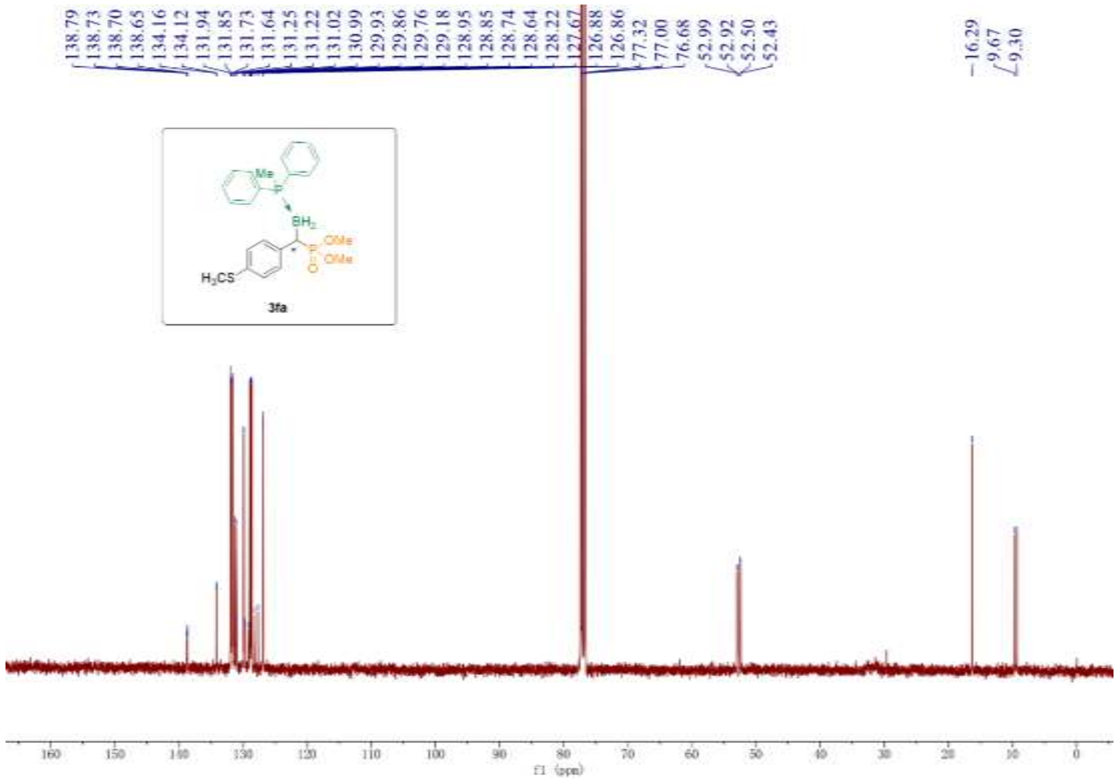

^{11}B NMR (128 MHz, Chloroform-*d*)

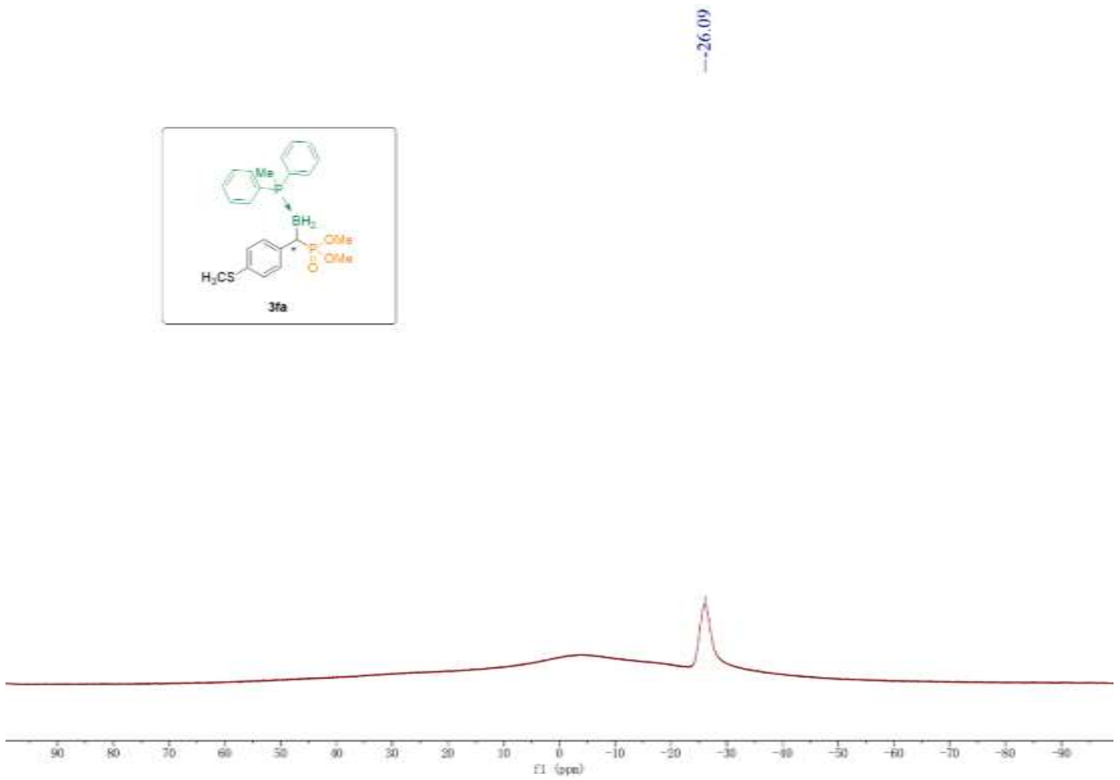

^{31}P NMR (162 MHz, Chloroform-*d*)

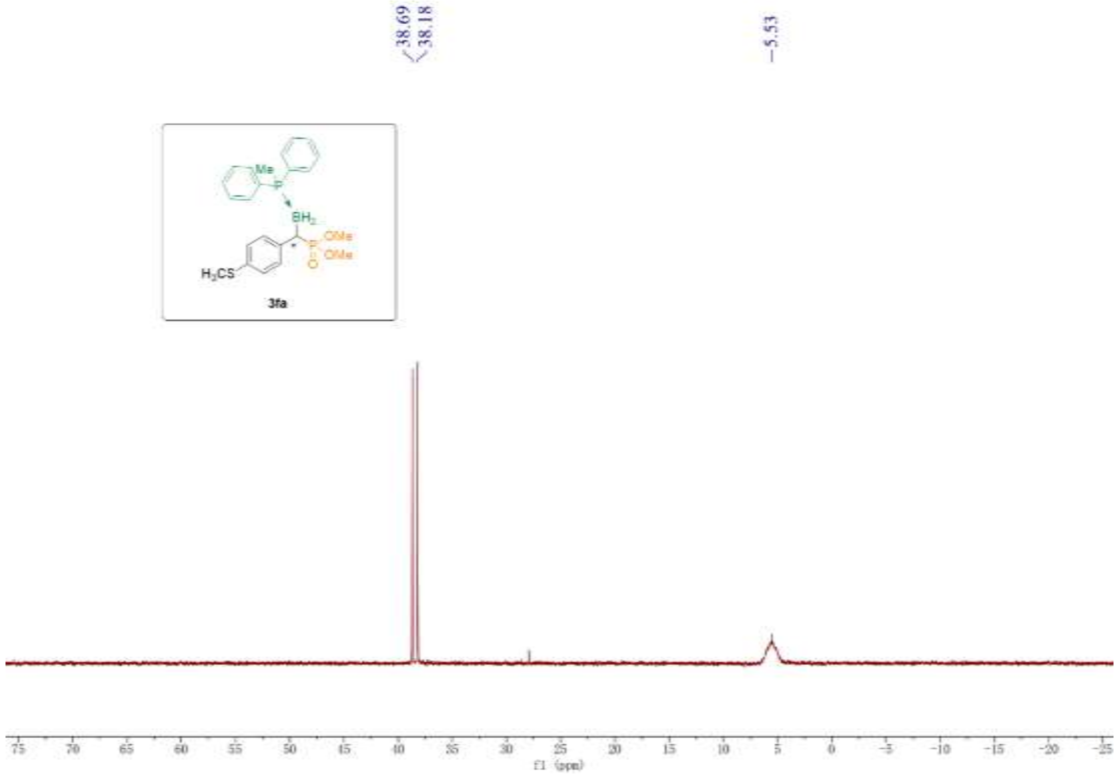

(S)-dimethyl(((methyldiphenylphosphane)boryl)(4-nitrophenyl)methyl)phosphonate(3ga)

^{1}H NMR (500 MHz, Chloroform-*d*)

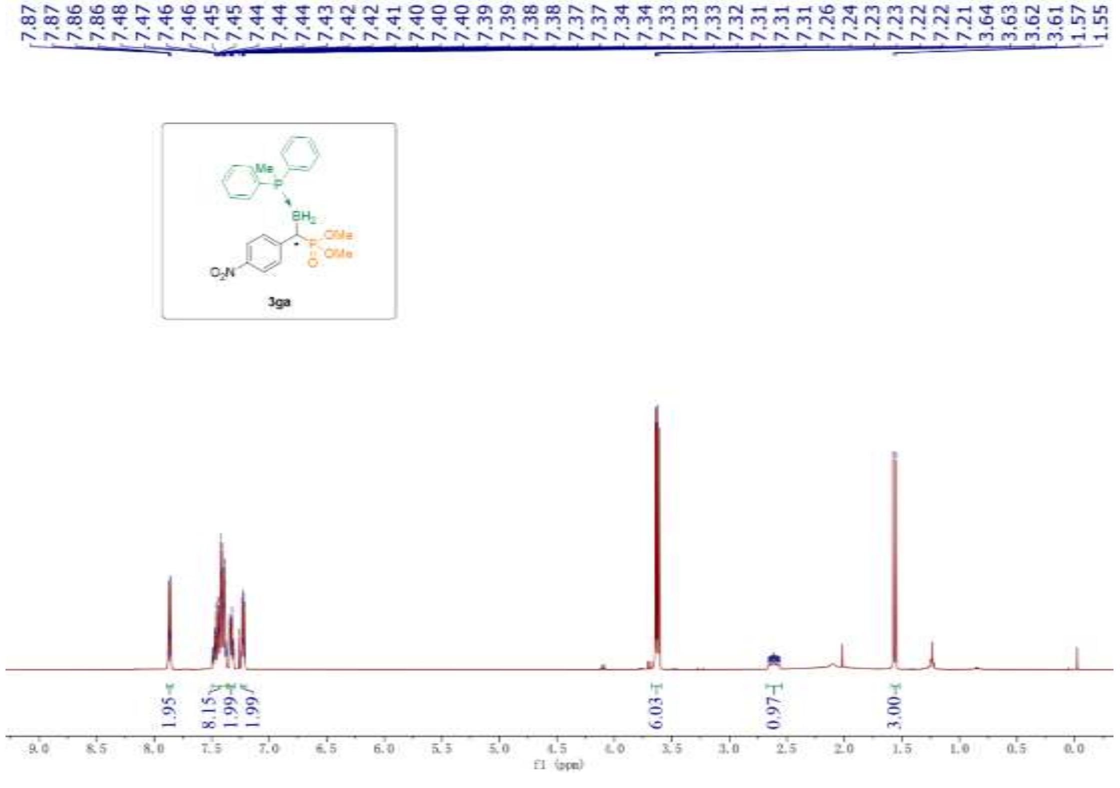

^{13}C NMR (126 MHz, Chloroform-*d*)

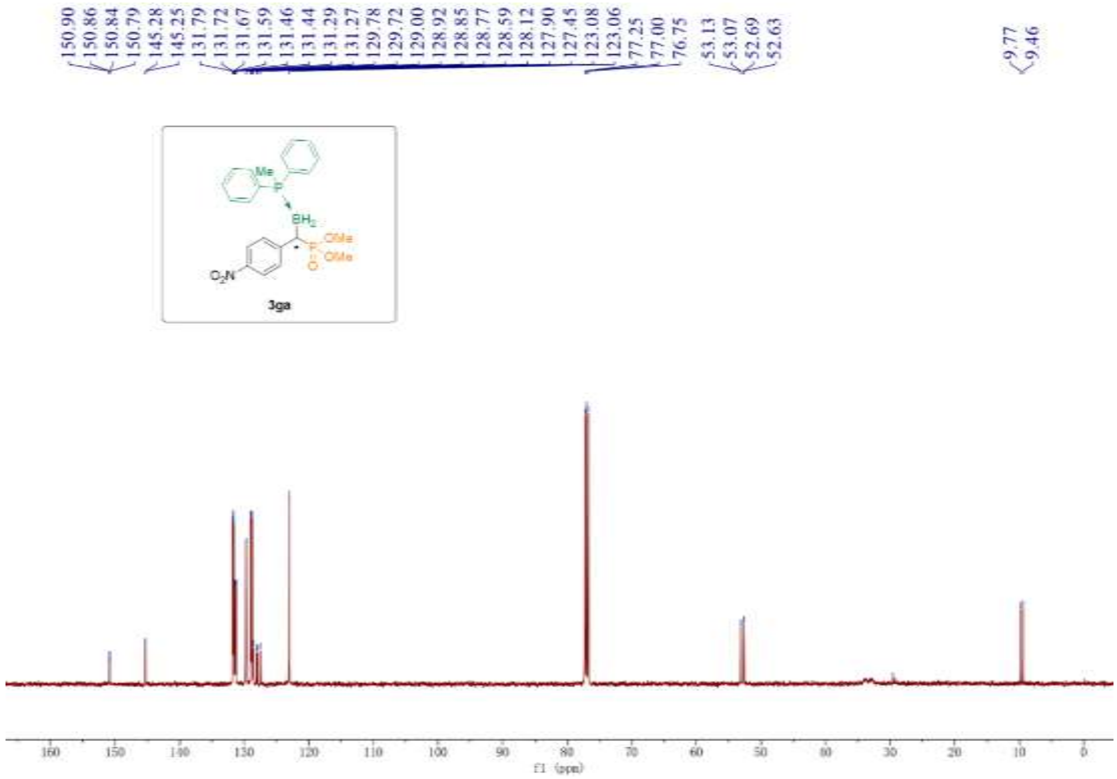

^{11}B NMR (160 MHz, Chloroform-*d*)

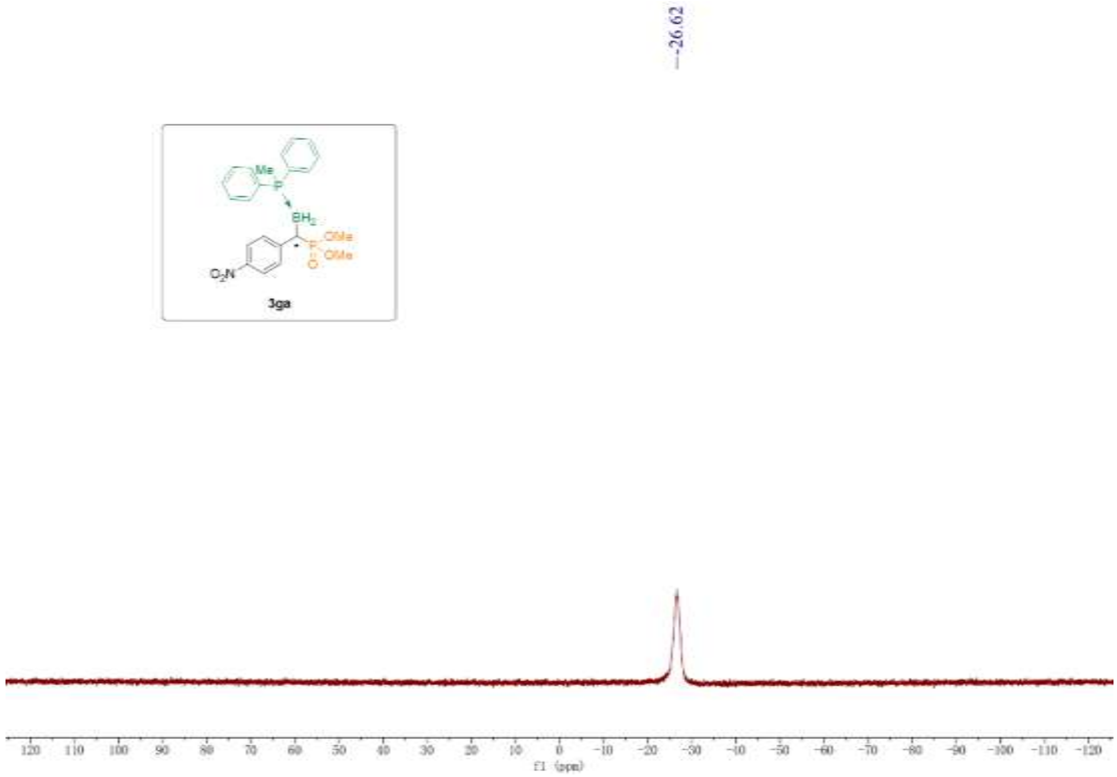

^{31}P NMR (202 MHz, Chloroform-*d*)

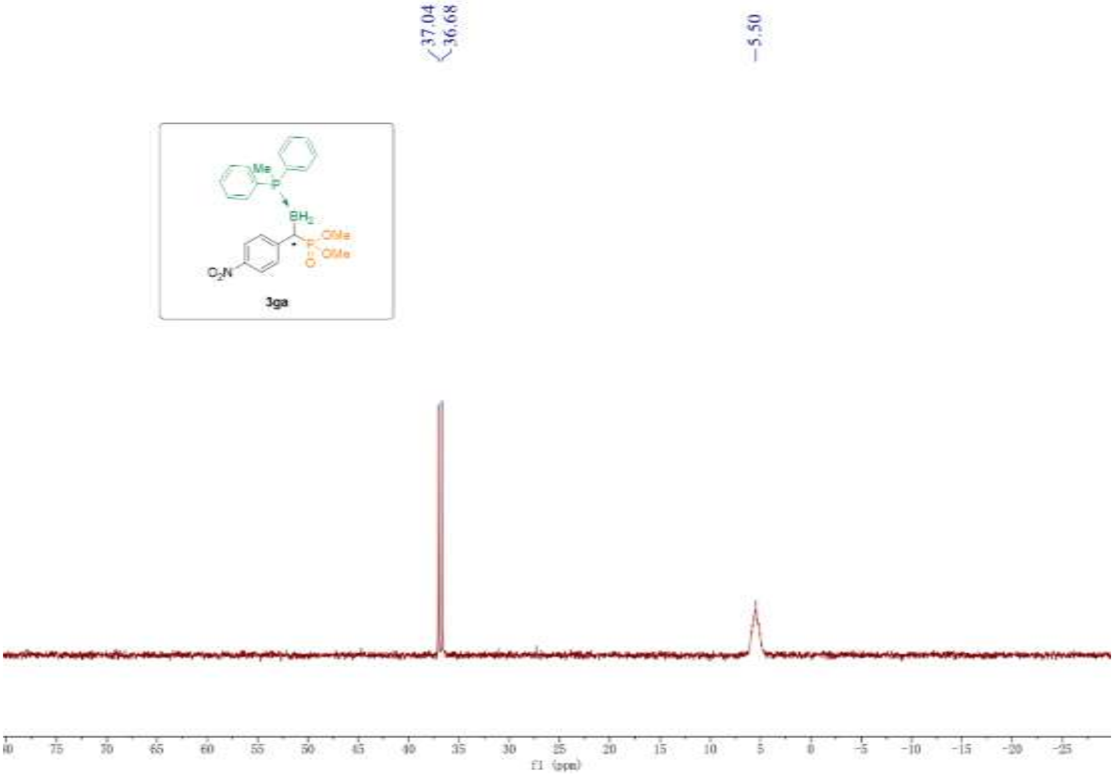

(S)-methyl-4-(((methyldiphenylphosphane)boryl)(dimethoxyphosphoryl)methyl)benzoate(3ha)

^{1}H NMR (500 MHz, Chloroform-*d*)

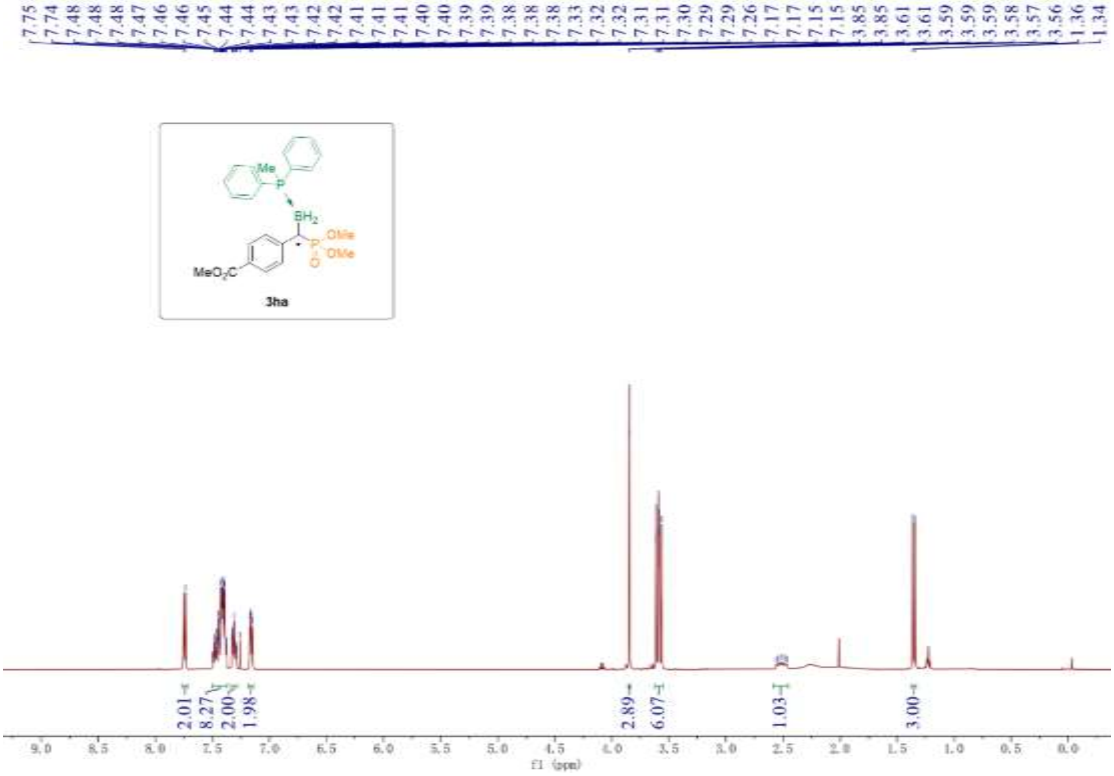

^{13}C NMR (126 MHz, Chloroform-*d*)

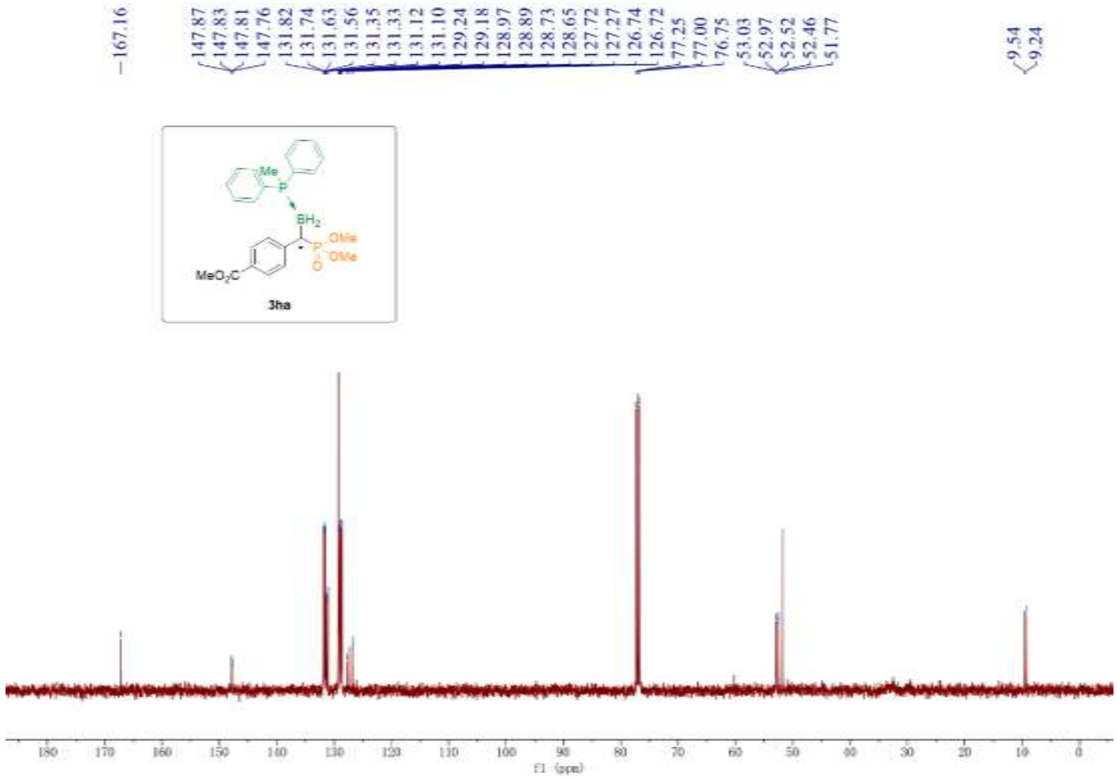

^{11}B NMR (160 MHz, Chloroform-*d*)

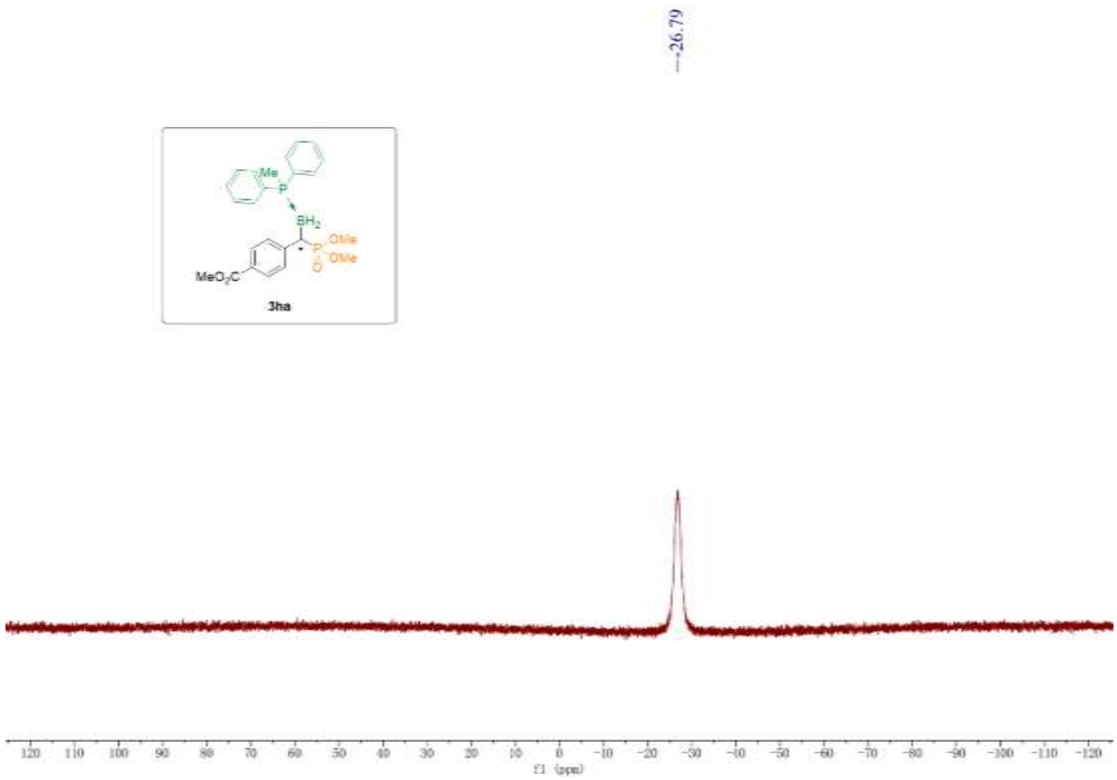

^{31}P NMR (202 MHz, Chloroform-*d*)

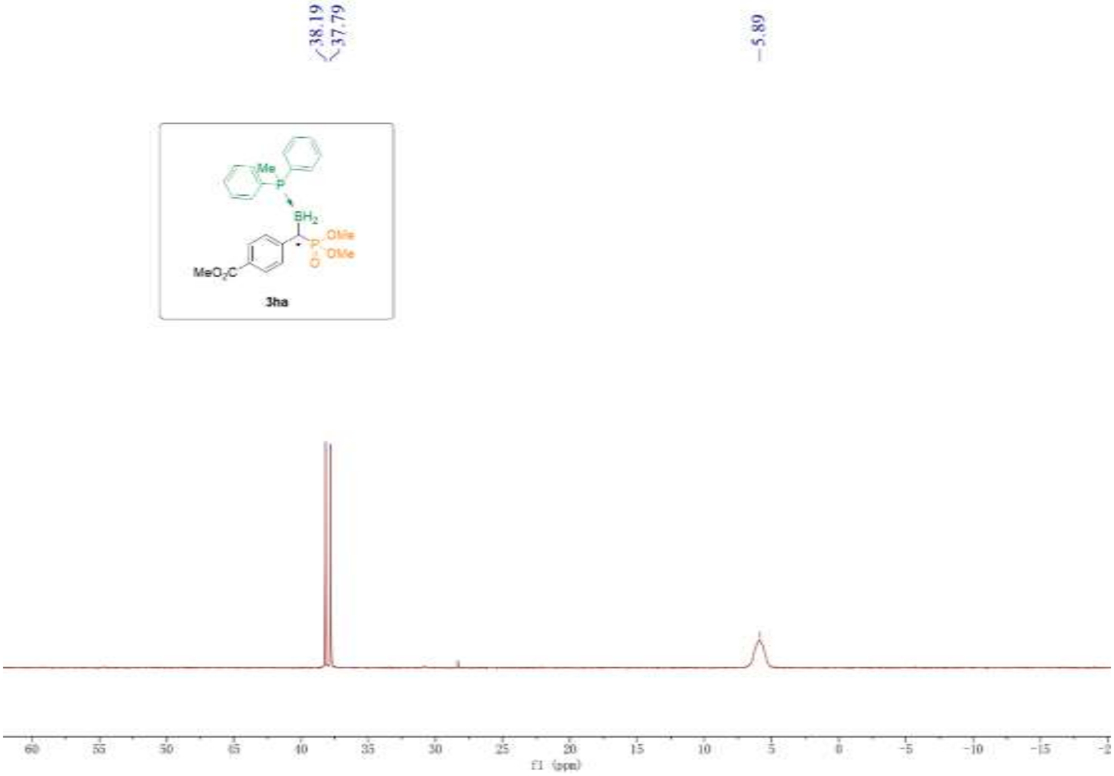

(S)-dimethyl(((methyldiphenylphosphane)boryl)(4-fluorophenyl)methyl)phosphonate(3ia)

^{1}H NMR (400 MHz, Chloroform-*d*)

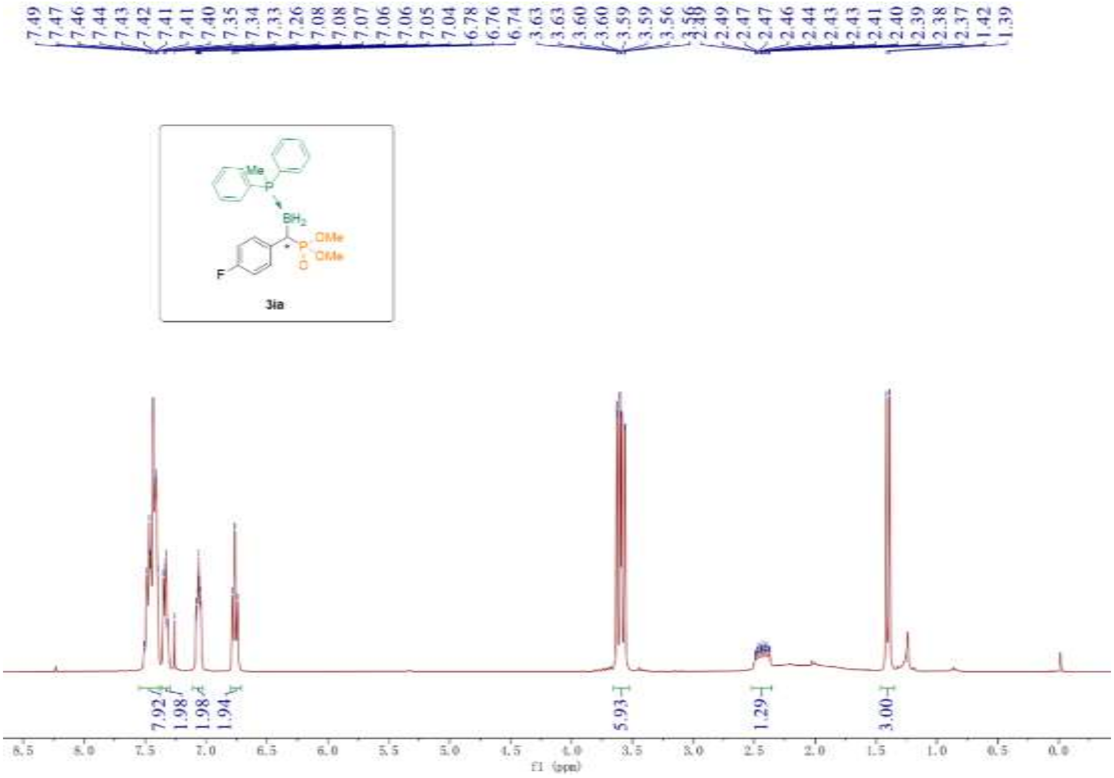

^{13}C NMR (101 MHz, Chloroform-*d*)

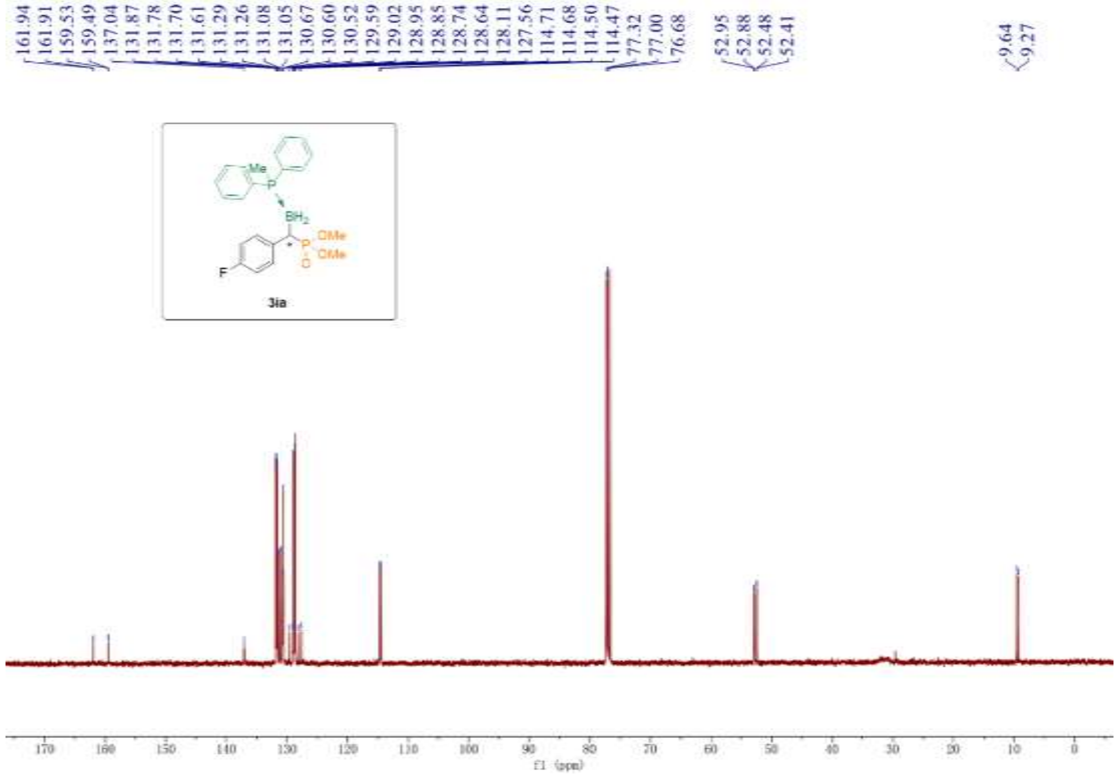

^{11}B NMR (128 MHz, Chloroform-*d*)

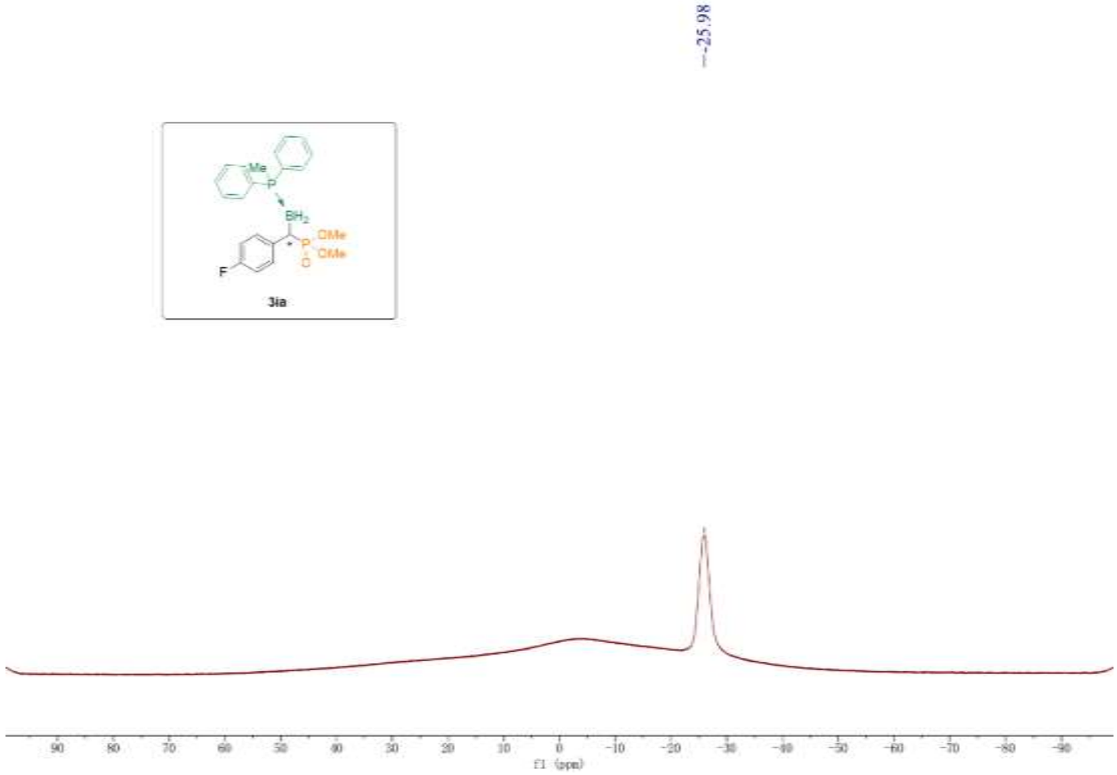

^{31}P NMR (162 MHz, Chloroform-*d*)

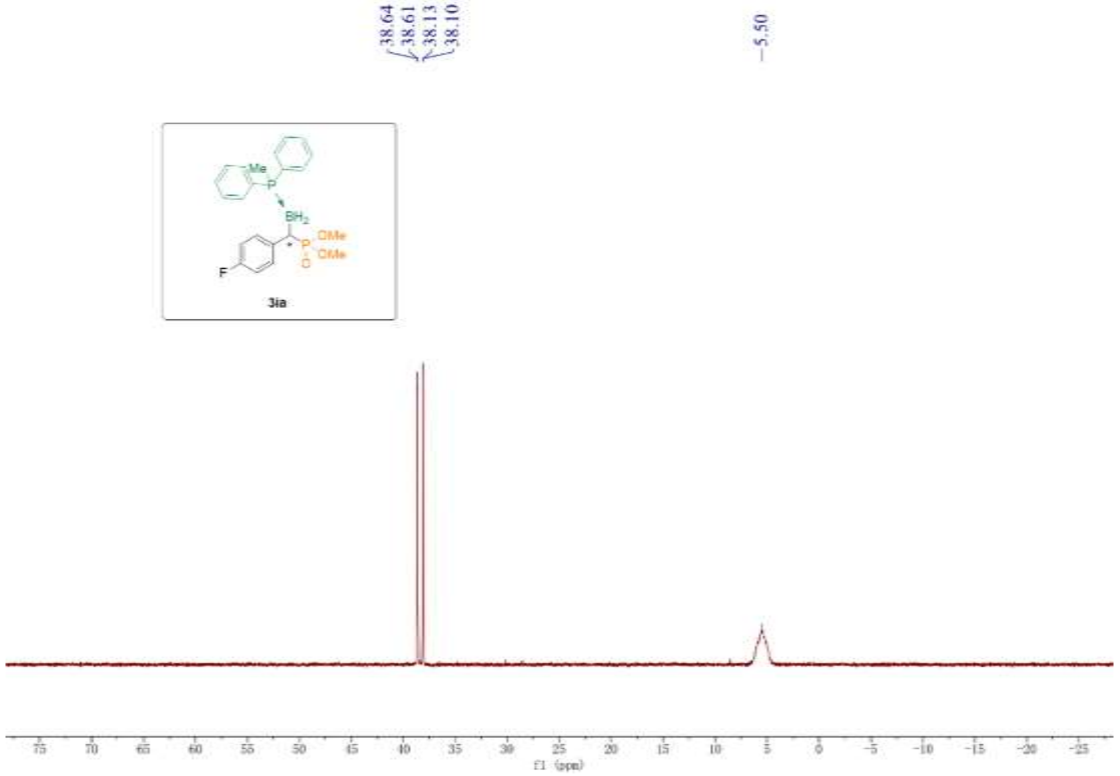

^{19}F NMR (376 MHz, Chloroform-*d*)

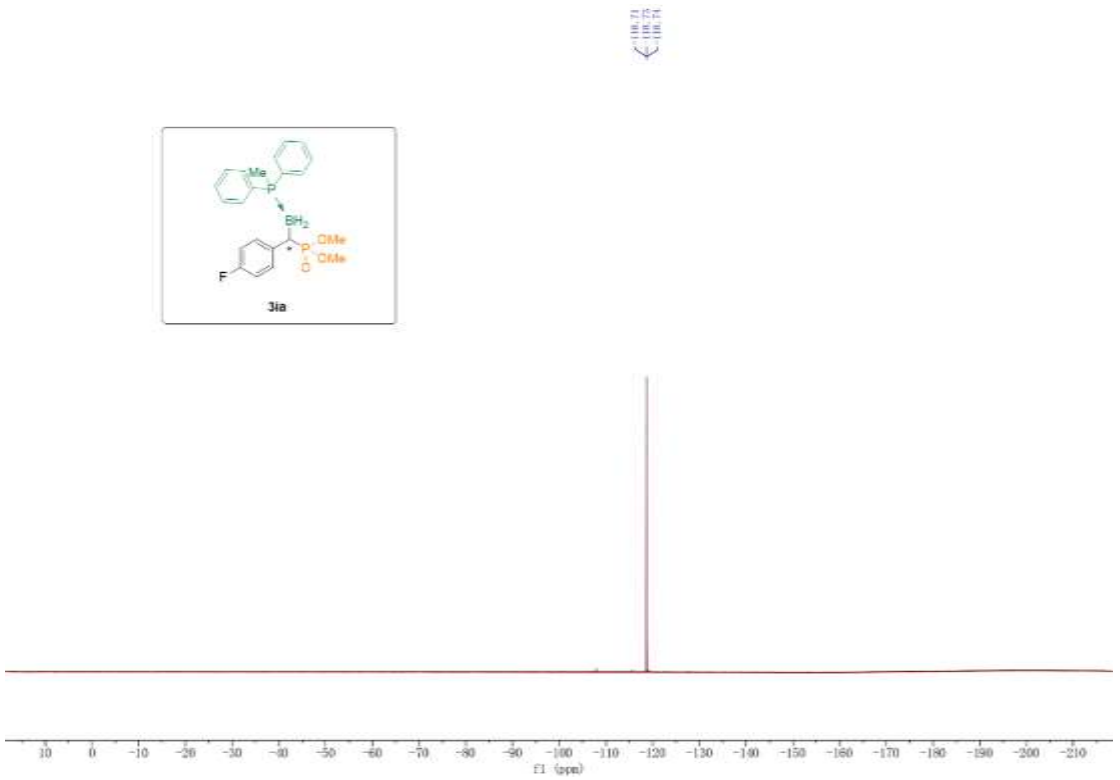

(S)-dimethy(((methyldiphenylphosphane)boryl)(4-bromophenyl)methyl)phosphonate(3ja)

^{1}H NMR (500 MHz, Chloroform-*d*)

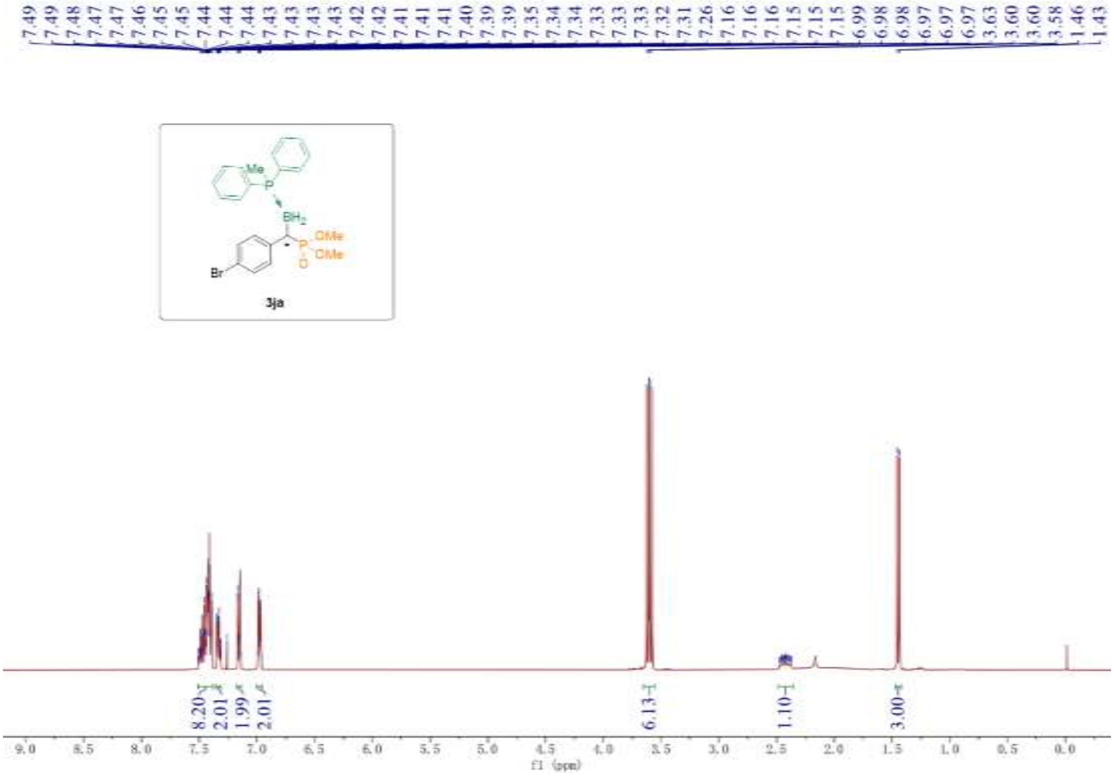

^{13}C NMR (126 MHz, Chloroform-*d*)

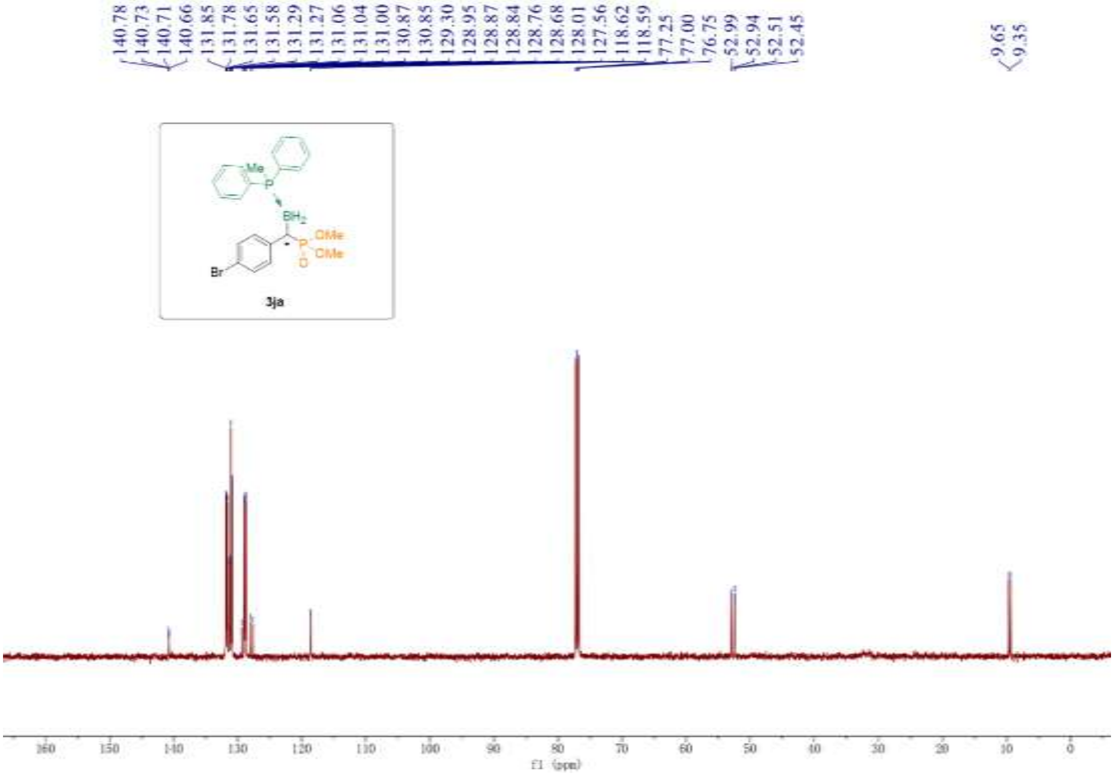

^{11}B NMR (160 MHz, Chloroform-*d*)

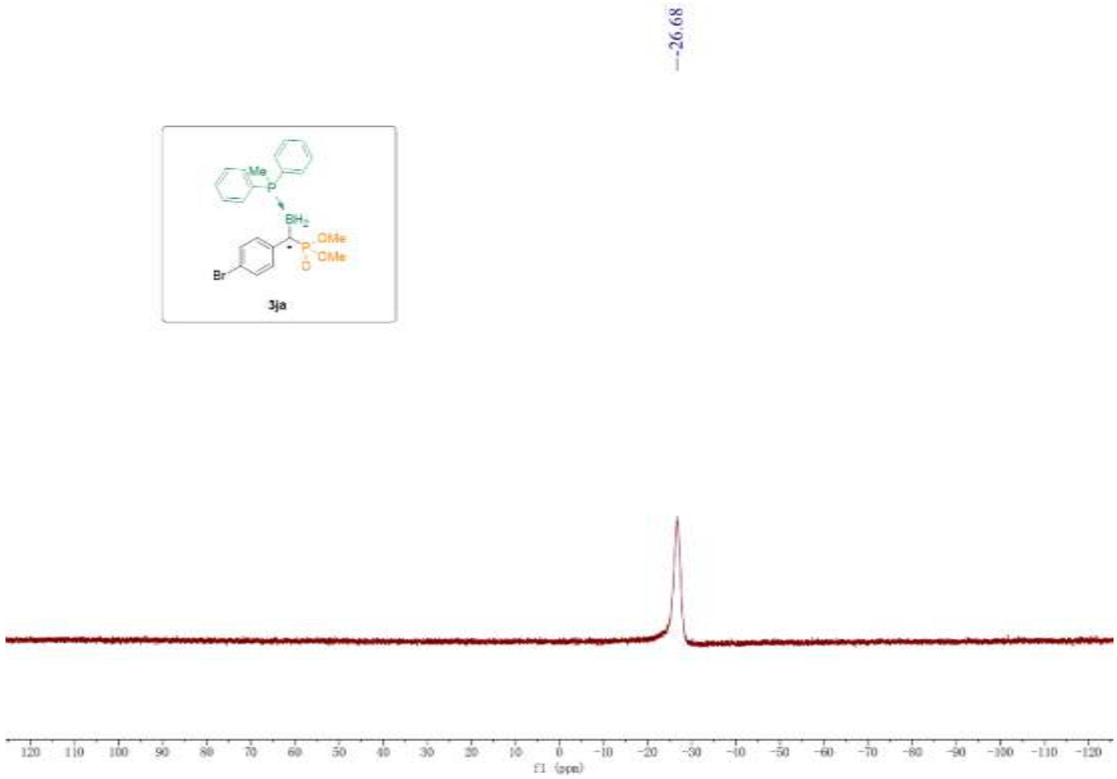

^{31}P NMR (202 MHz, Chloroform-*d*)

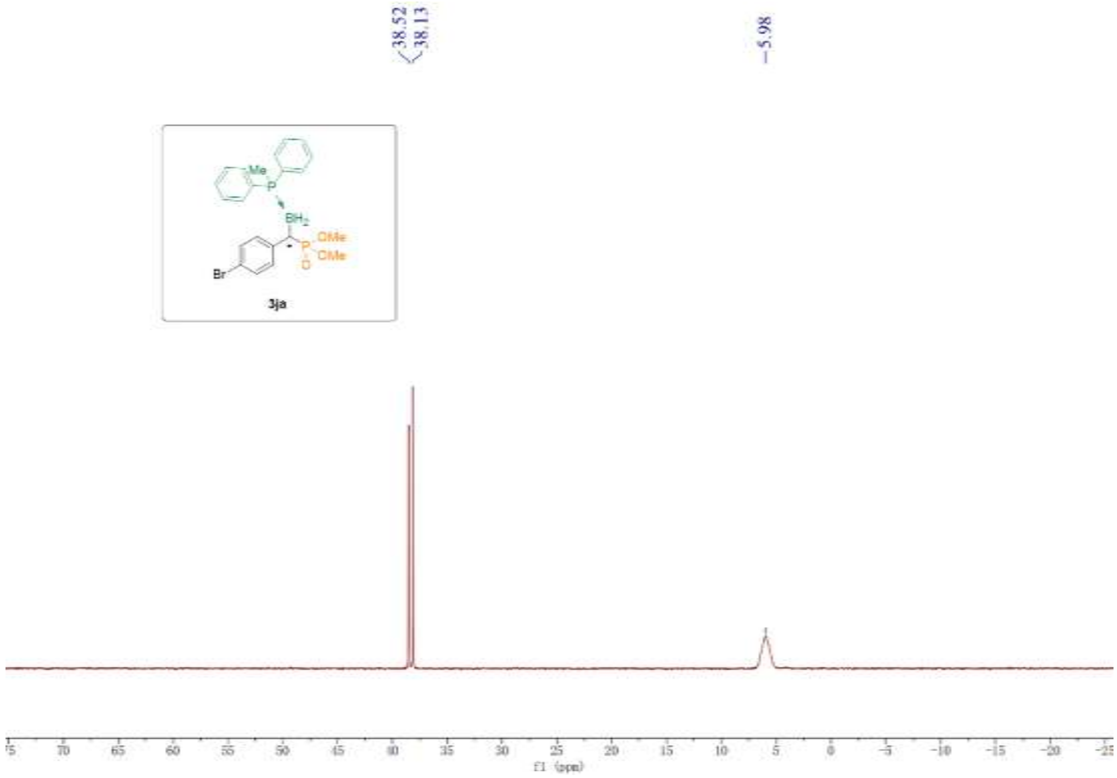

(S)-dimethyl(((methyldiphenylphosphane)boryl)(4-(trifluoromethyl)phenyl)methyl) phosphonate(3ka)

^{1}H NMR (400 MHz, Chloroform-*d*)

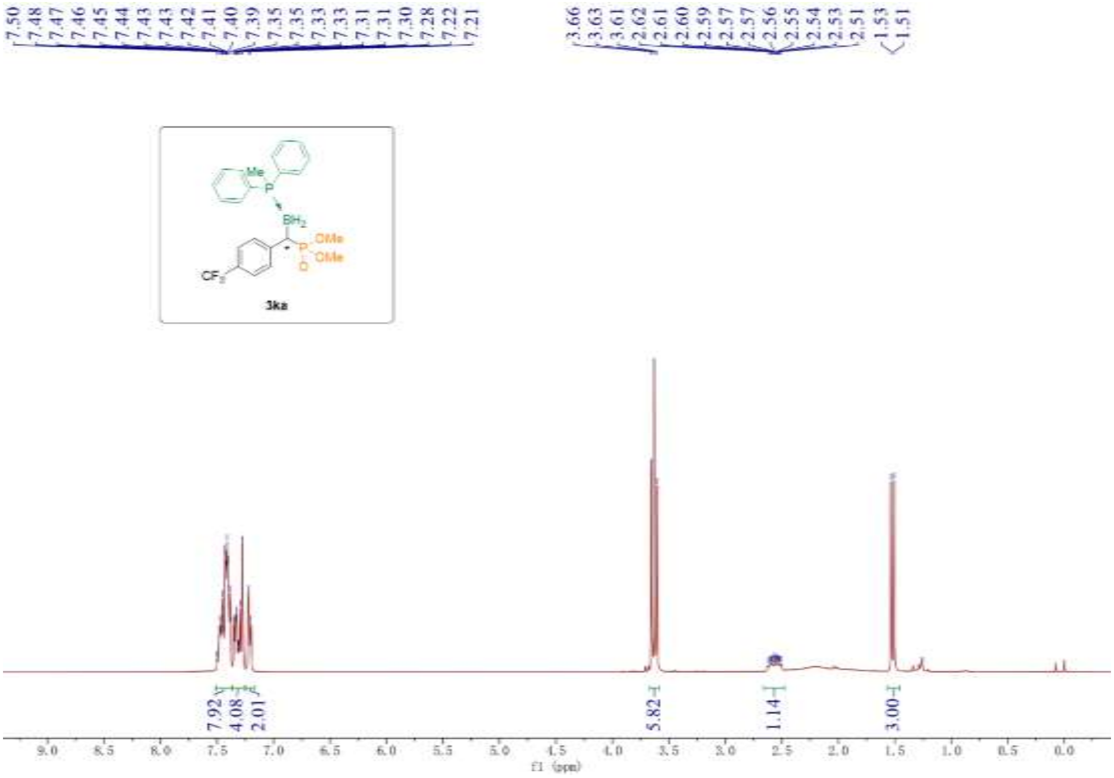

^{13}C NMR (101 MHz, Chloroform-*d*)

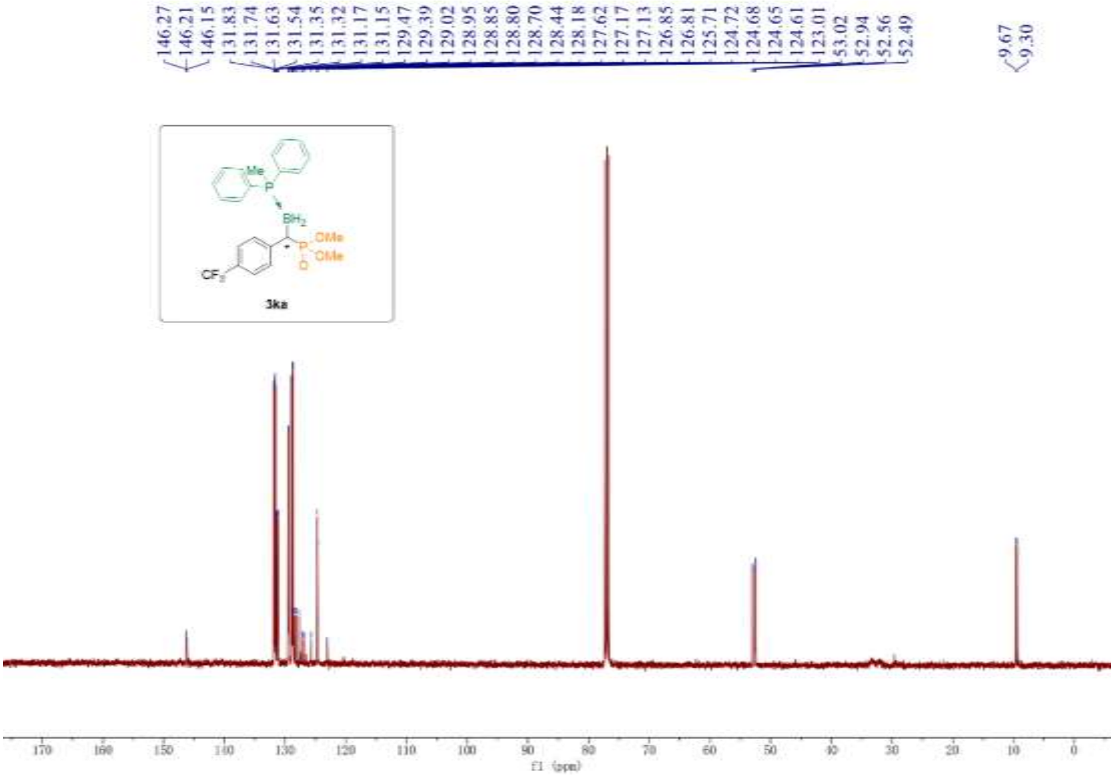

^{11}B NMR (128 MHz, Chloroform-*d*)

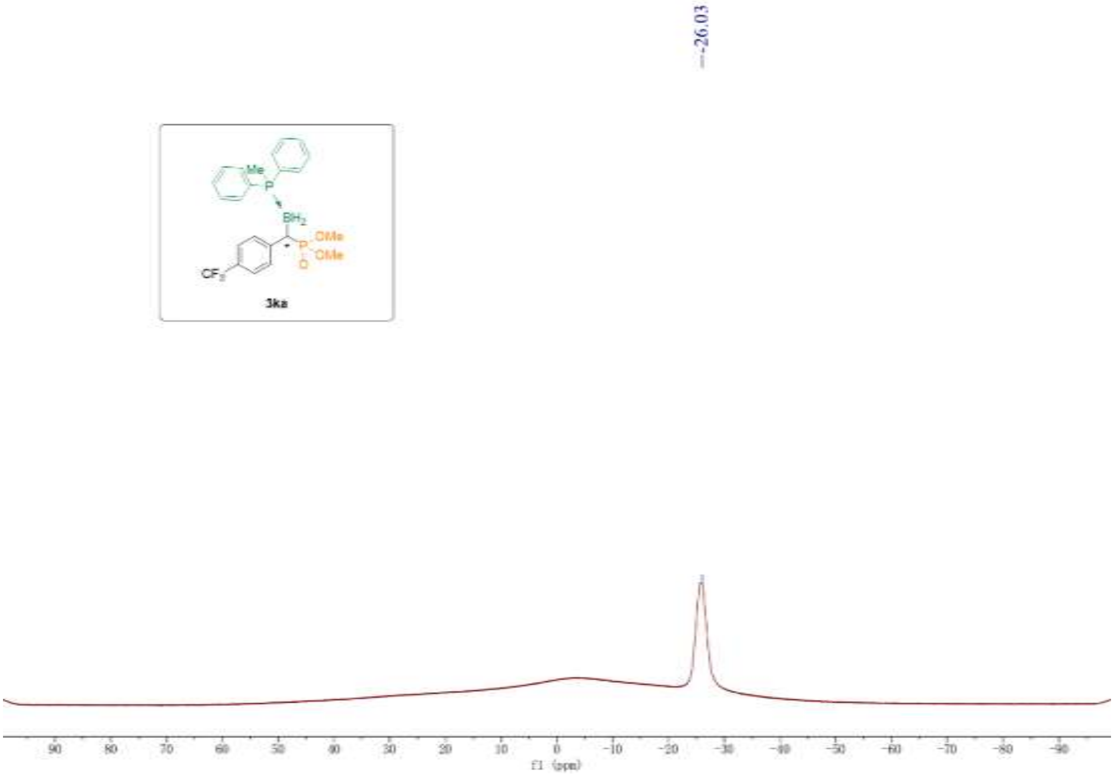

^{31}P NMR (162 MHz, Chloroform-*d*)

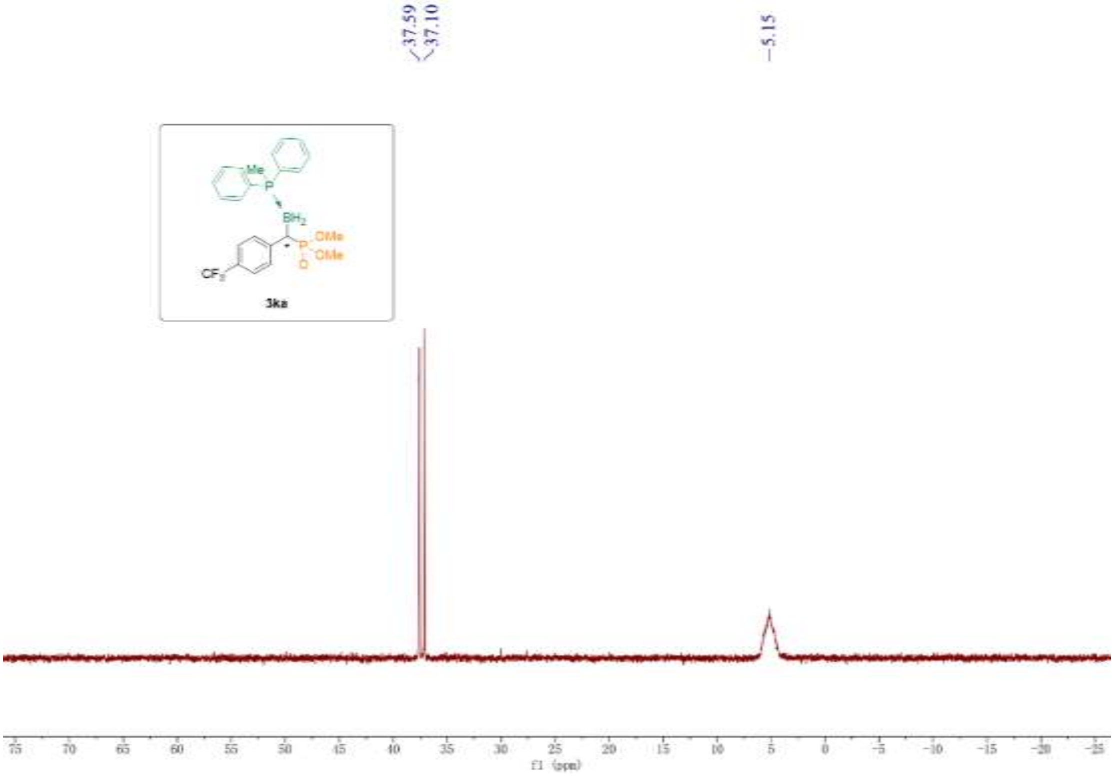

^{19}F NMR (376 MHz, Chloroform-*d*)

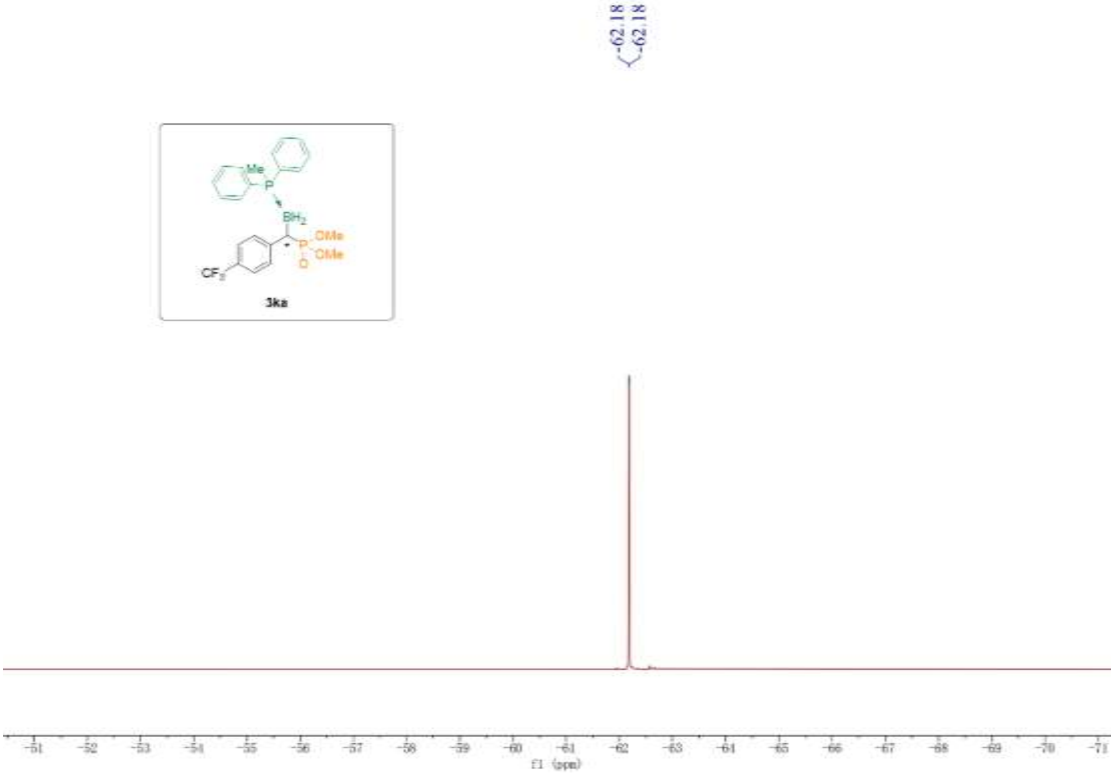

(S)-dimethyl(((methyldiphenylphosphane)boryl)([1,1'-biphenyl]-4-yl)methyl)phosphonate(3la)

^{1}H NMR (400 MHz, Chloroform-*d*)

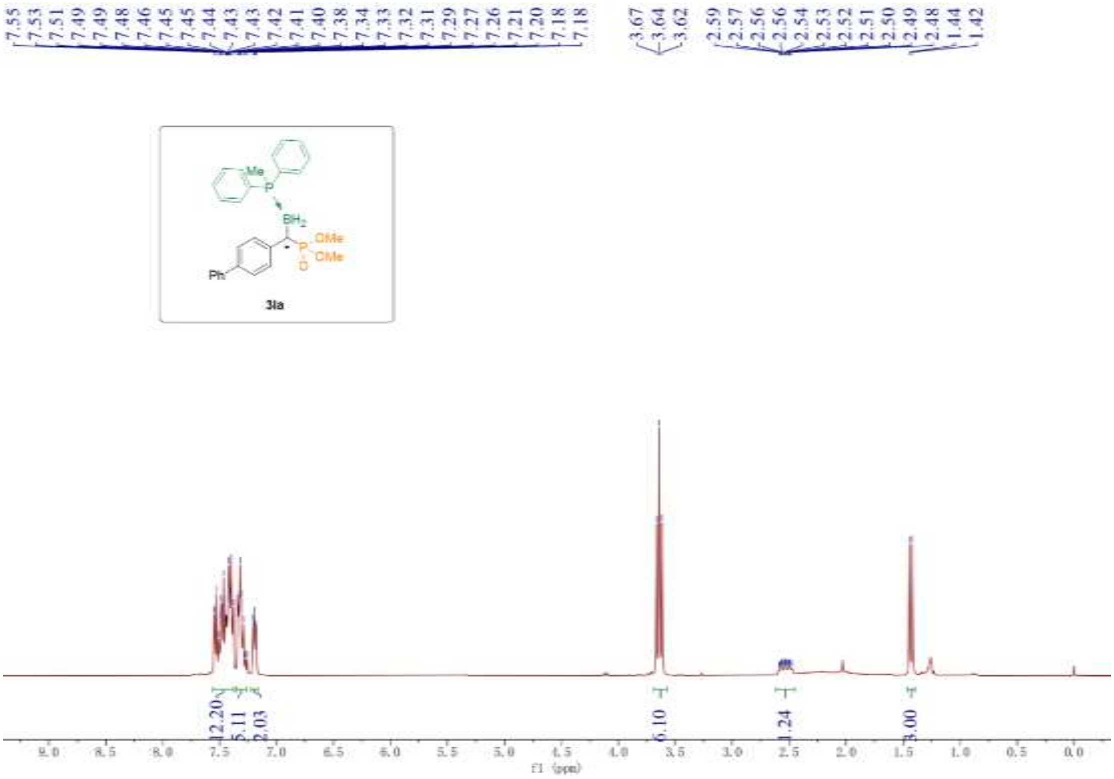

^{13}C NMR (101 MHz, Chloroform-*d*)

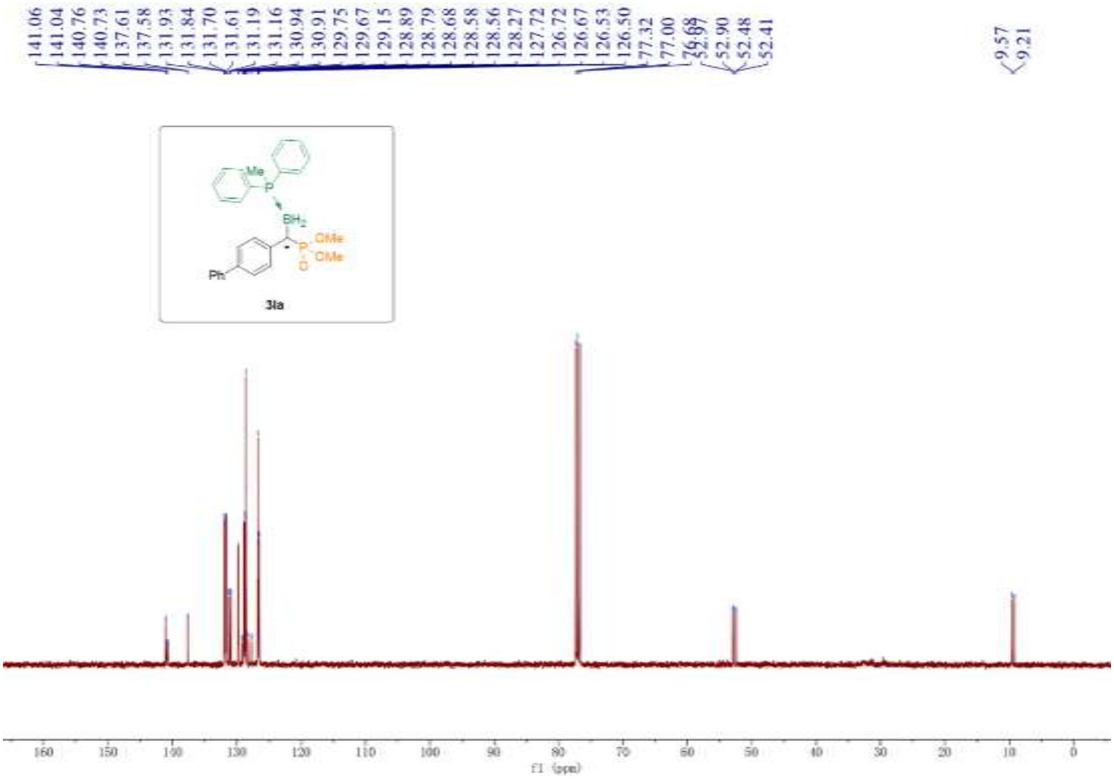

^{11}B NMR (128 MHz, Chloroform-*d*)

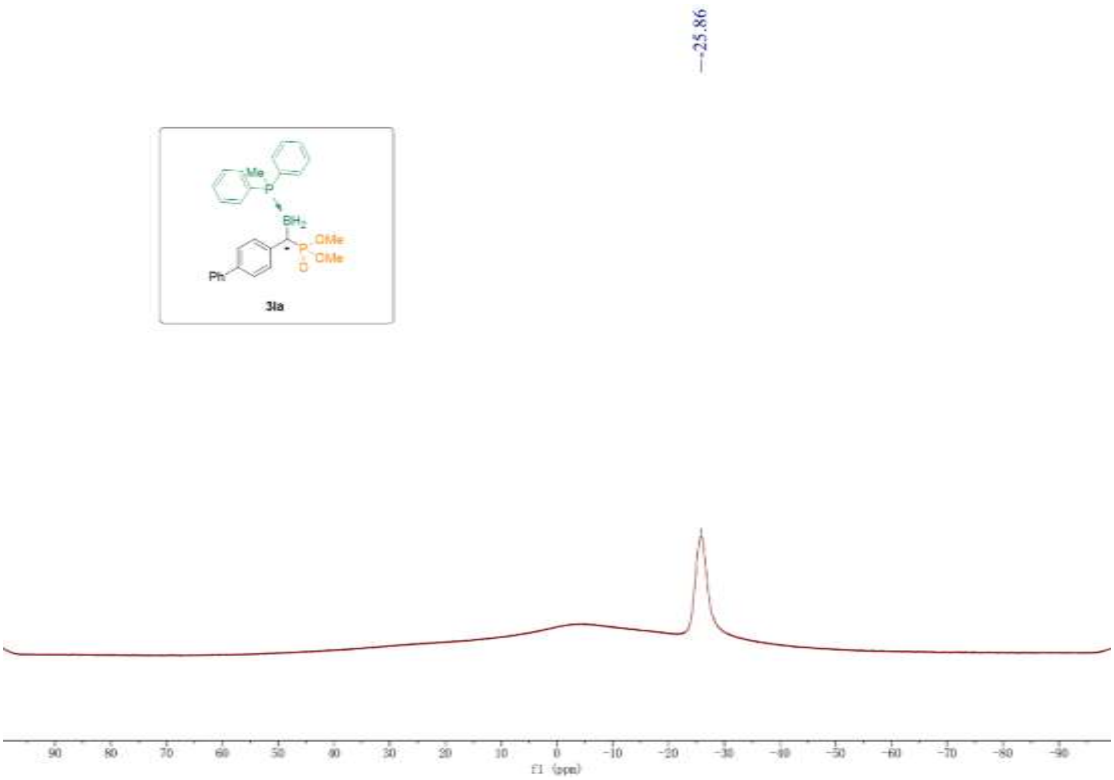

^{31}P NMR (162 MHz, Chloroform-*d*)

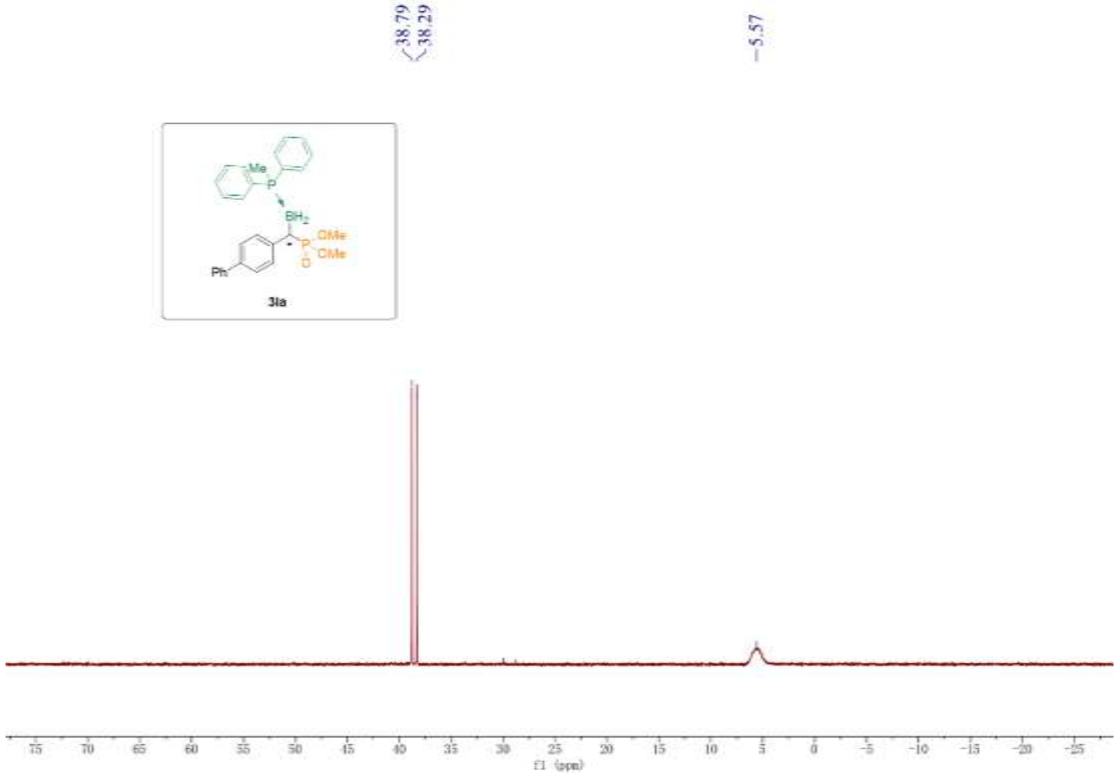

(S)-dimethyl(((methyldiphenylphosphane)boryl)(4-(benzyloxy)phenyl)methyl) phosphonate(3ma)

^{1}H NMR (400 MHz, Chloroform-*d*)

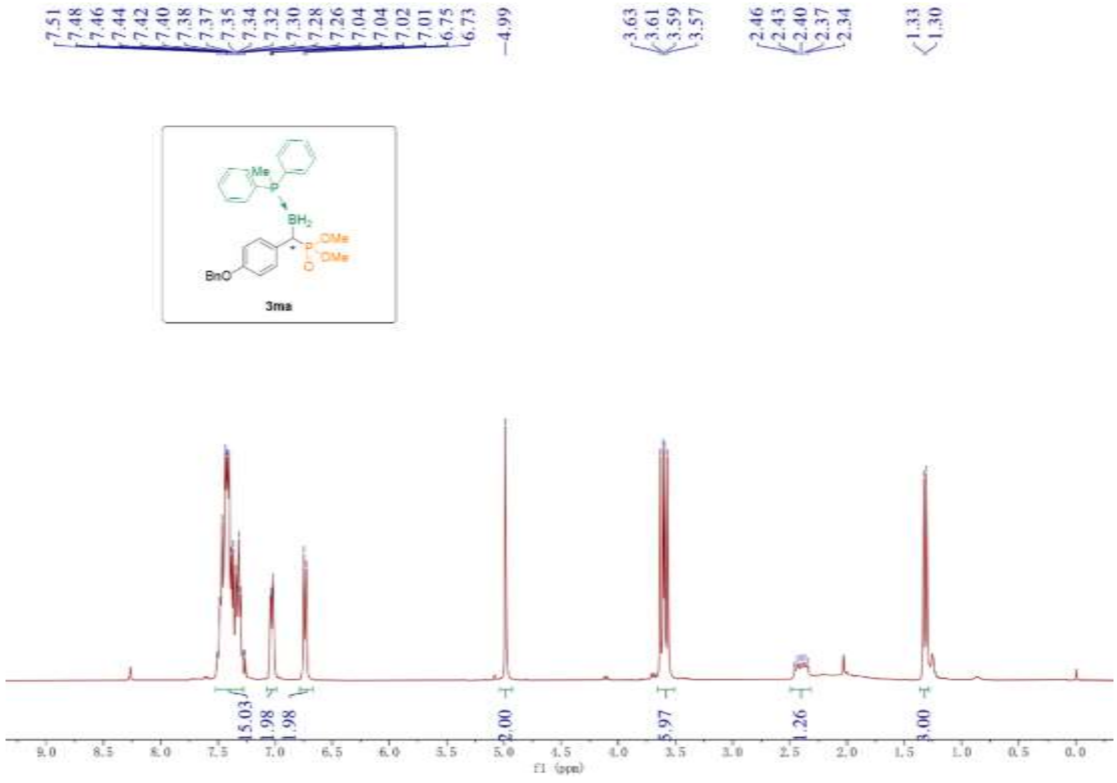

^{13}C NMR (101 MHz, Chloroform-*d*)

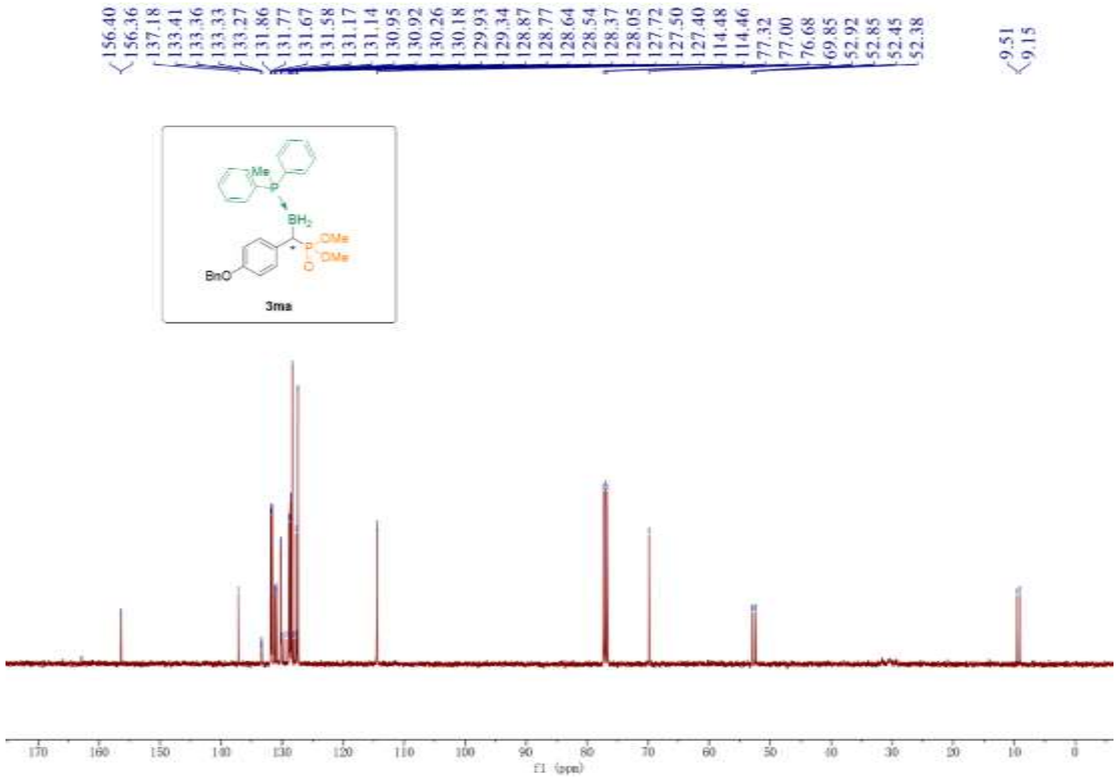

^{11}B NMR (128 MHz, Chloroform-*d*)

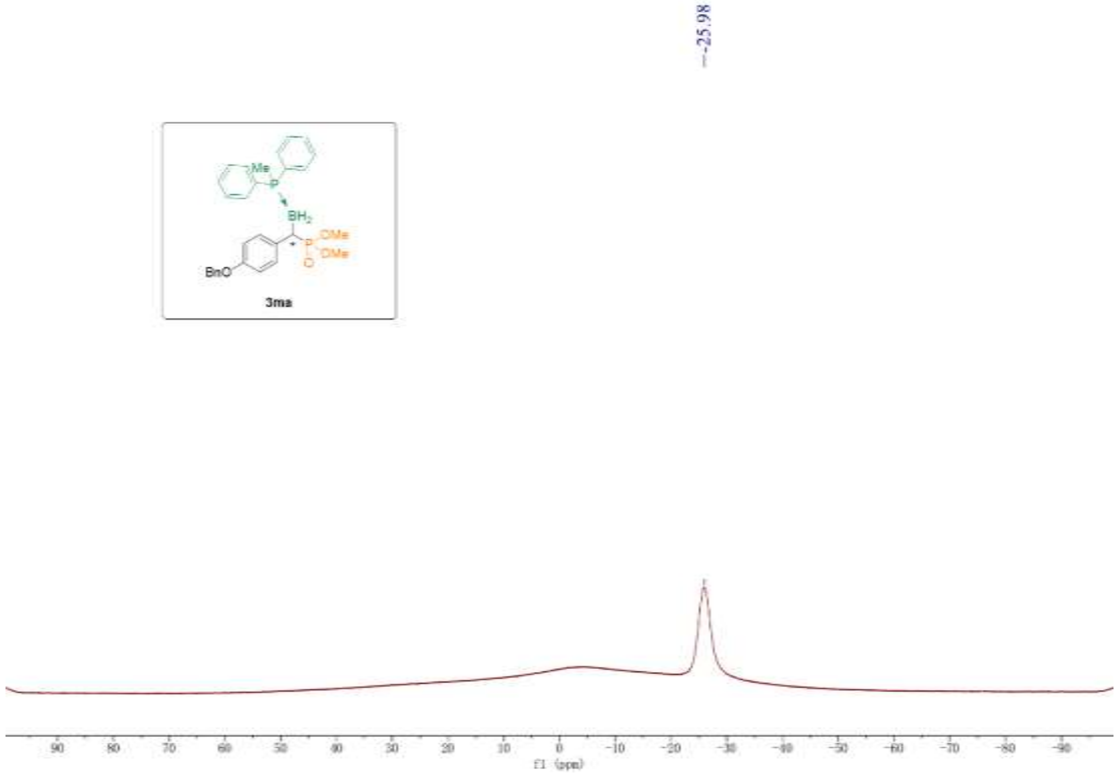

^{31}P NMR (162 MHz, Chloroform-*d*)

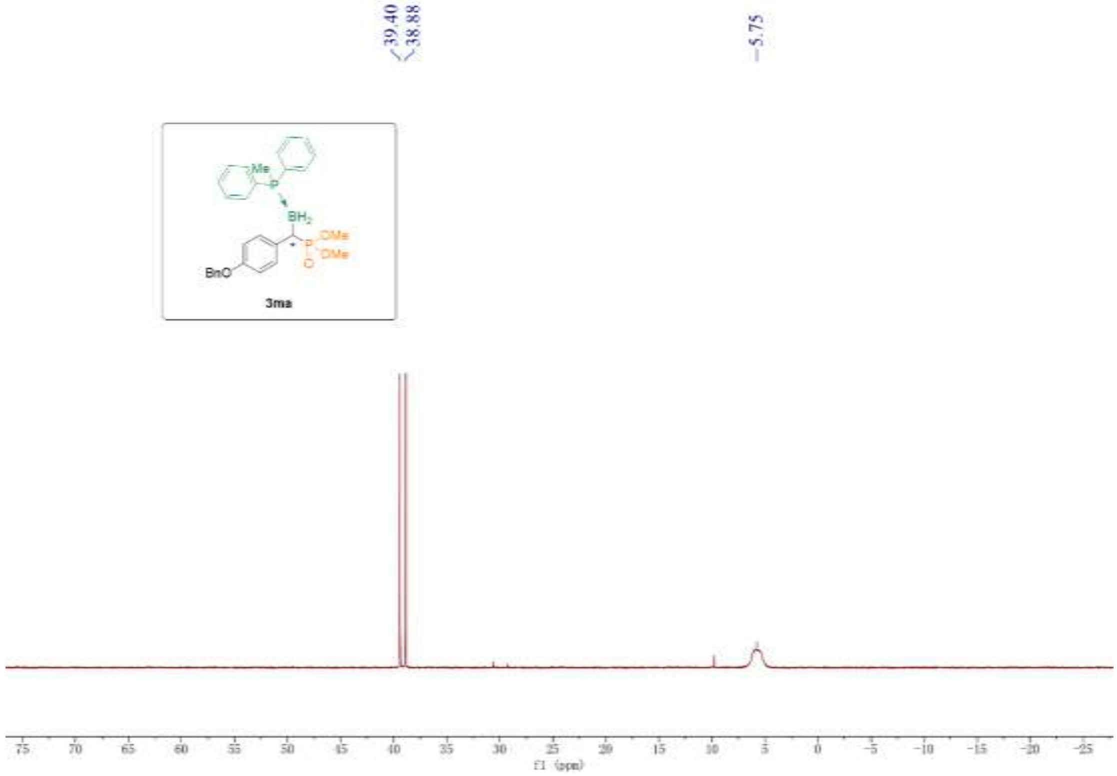

(S)-dimethy(((methyldiphenylphosphane)boryl)(4-cyanophenyl)methyl)phosphonate(3na)

^{1}H NMR (500 MHz, Chloroform-*d*)

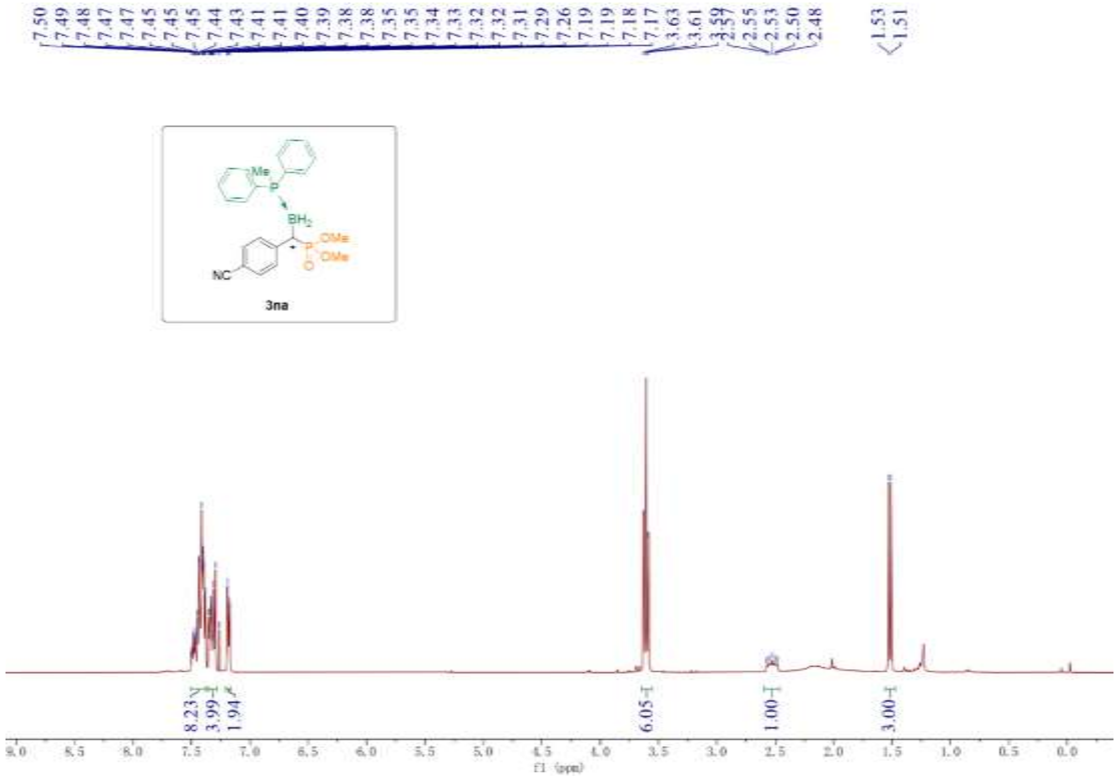

^{13}C NMR (126 MHz, Chloroform-*d*)

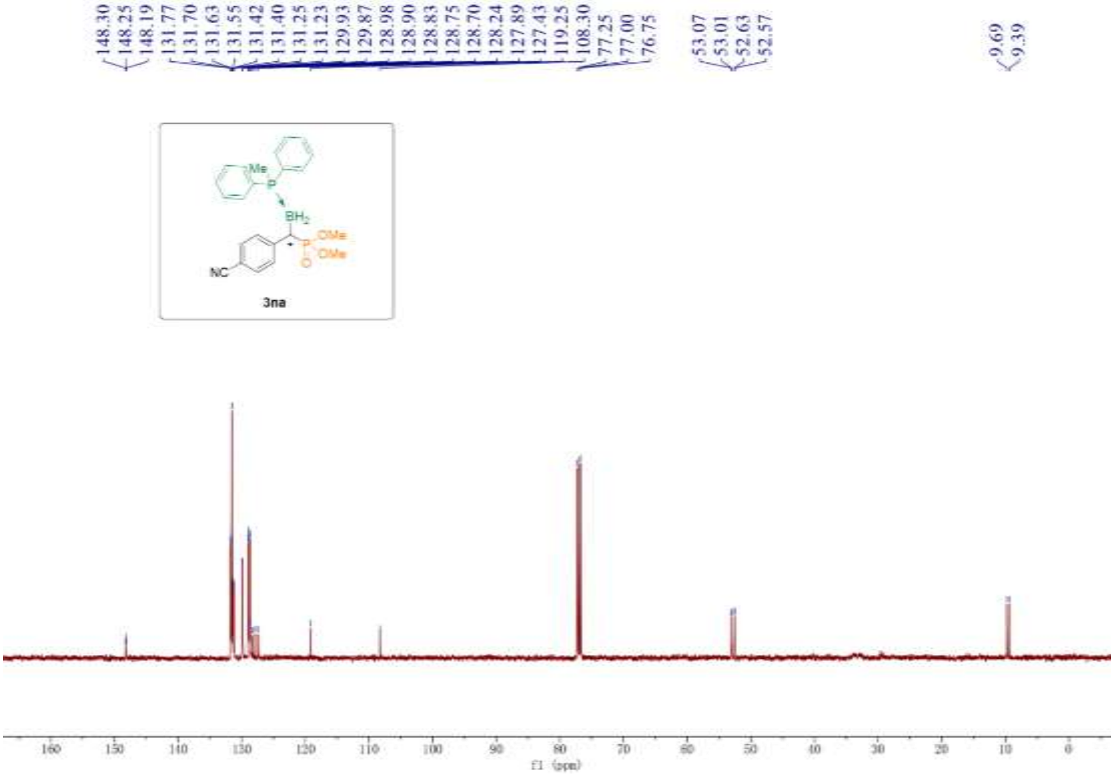

^{11}B NMR (128 MHz, Chloroform-*d*)

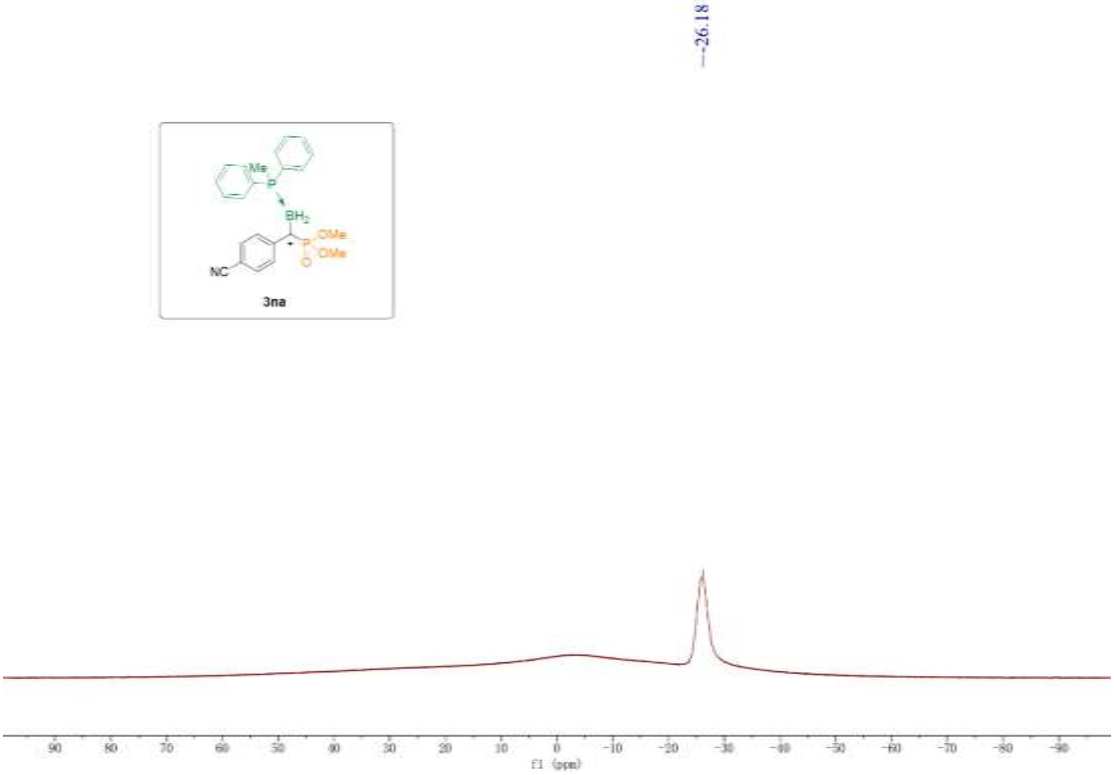

^{31}P NMR (202 MHz, Chloroform-*d*)

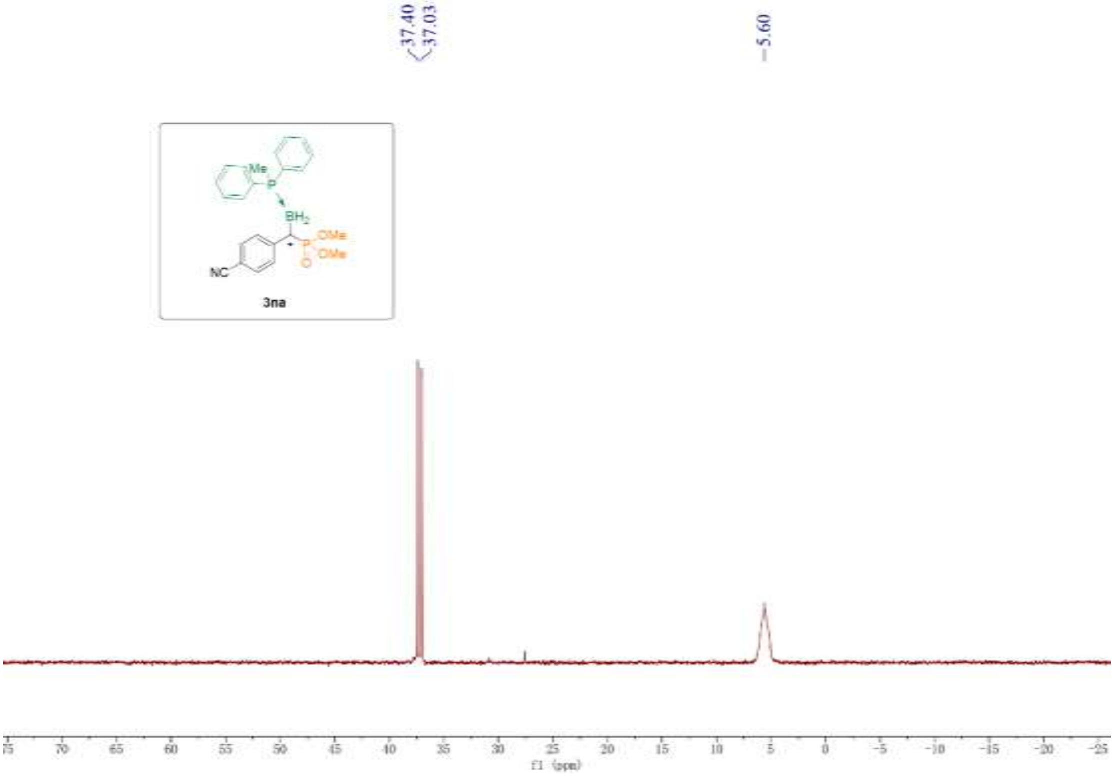

(S)-dimethy(((methyldiphenylphosphane)boryl)(3-methoxyphenyl)methyl)phosphonate(3oa)

^{1}H NMR (400 MHz, Chloroform-*d*)

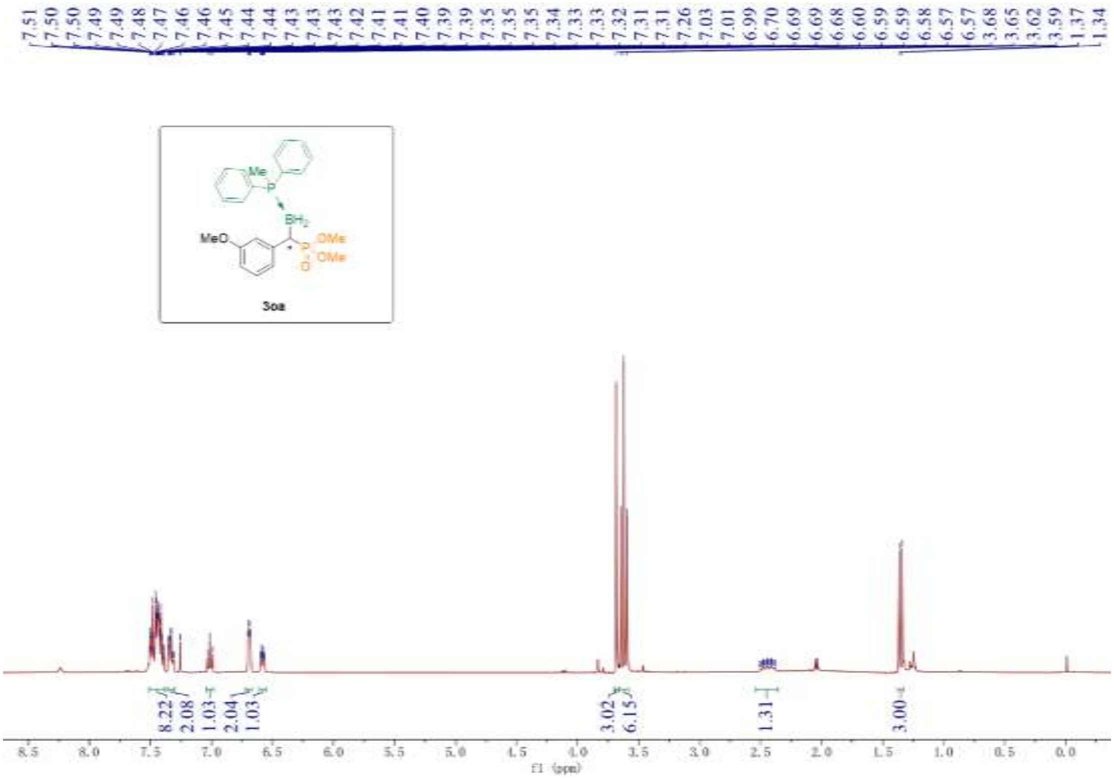

^{13}C NMR (101 MHz, Chloroform-*d*)

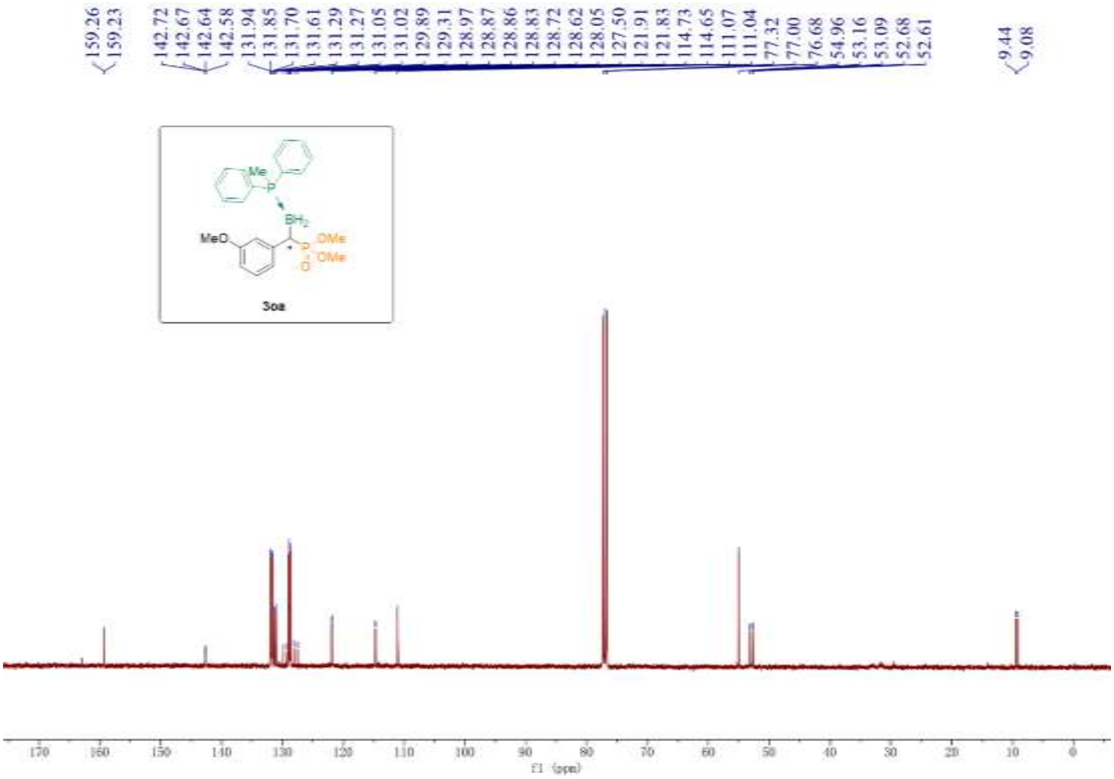

^{11}B NMR (128 MHz, Chloroform-*d*)

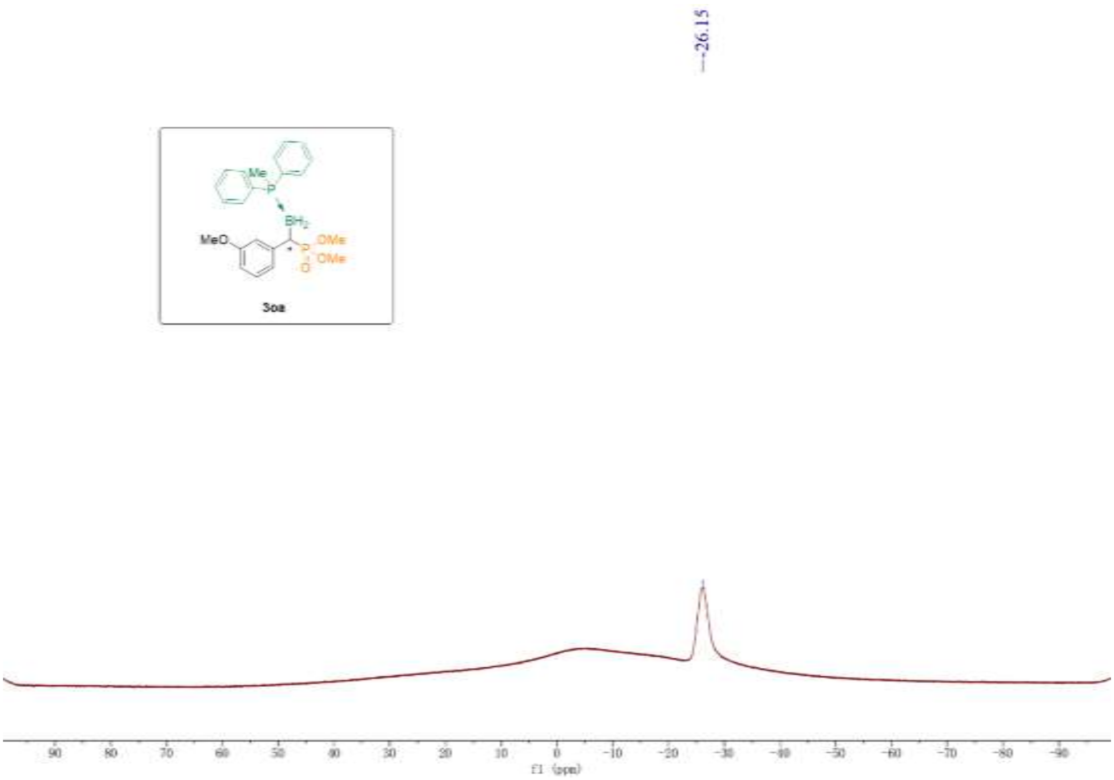

^{31}P NMR (162 MHz, Chloroform-*d*)

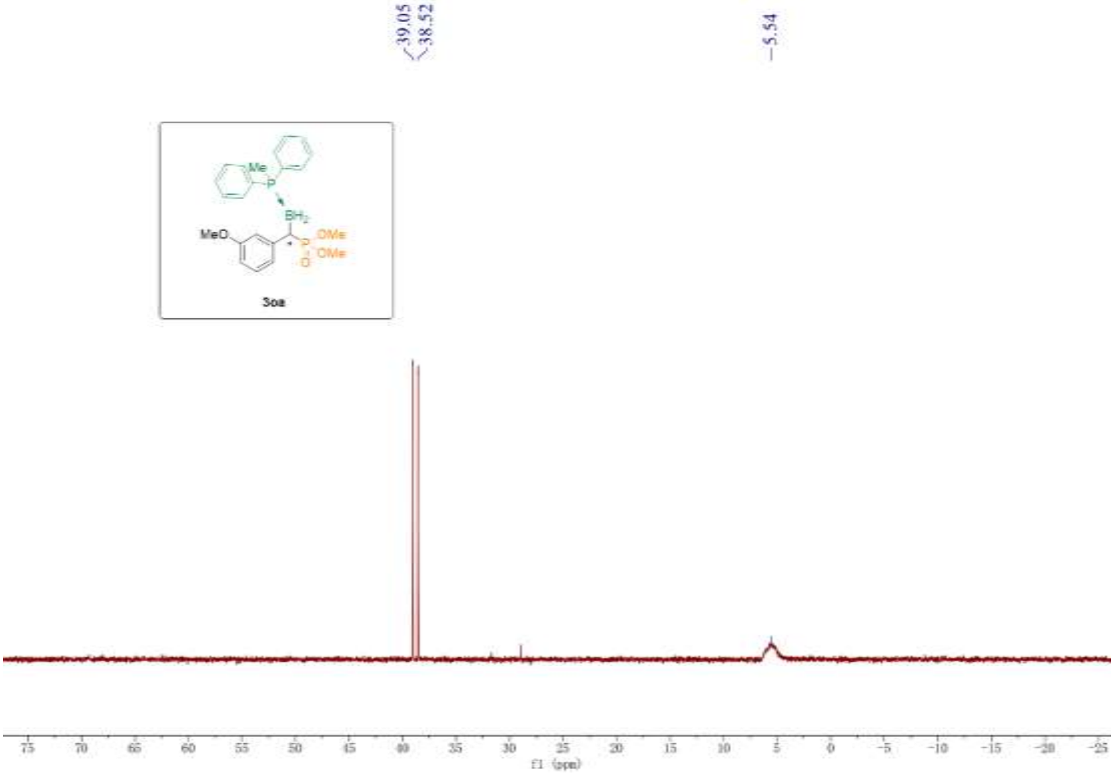

(S)-methyl-3-(((methyldiphenylphosphane)boryl) (dimethoxyphosphoryl)methyl)benzoate (3pa)

^{1}H NMR (400 MHz, Chloroform-*d*)

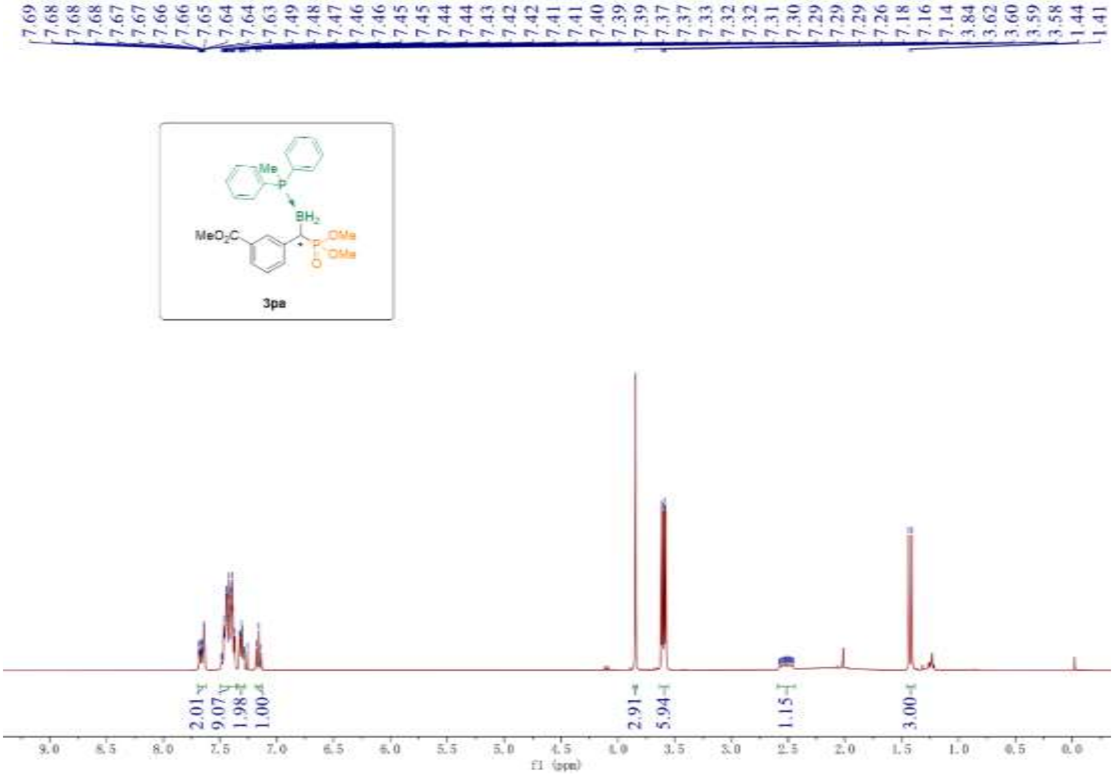

^{13}C NMR (101 MHz, Chloroform-*d*)

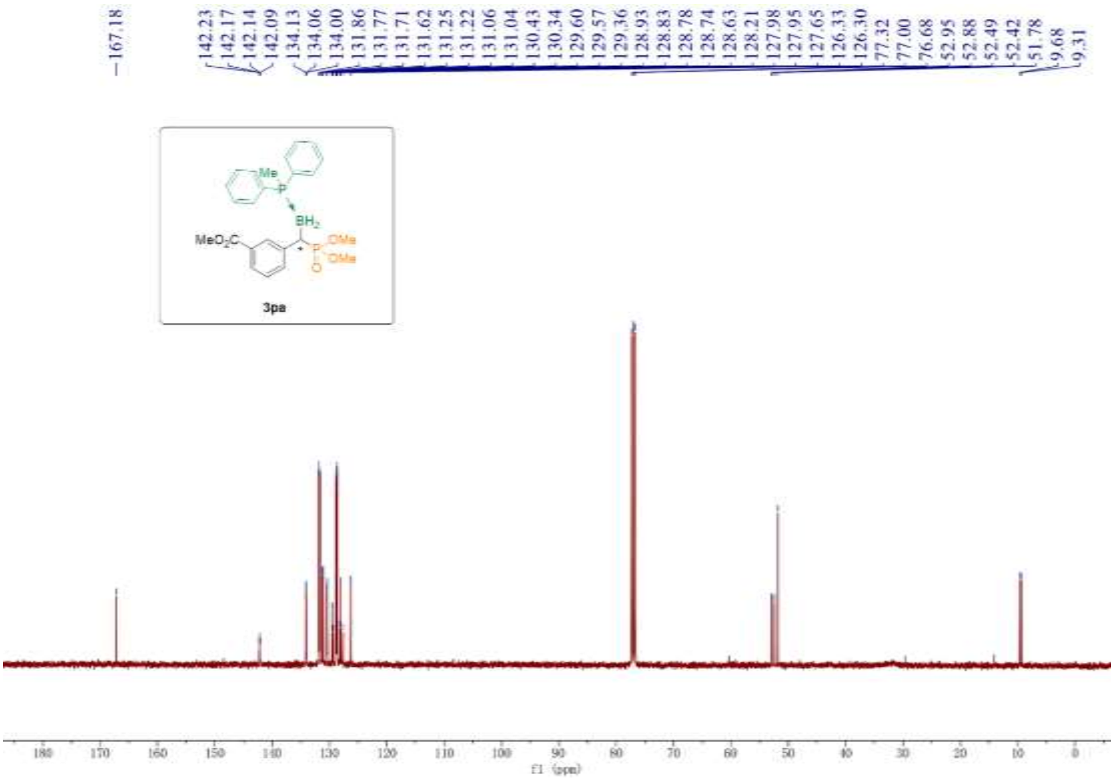

^{11}B NMR (128 MHz, Chloroform-*d*)

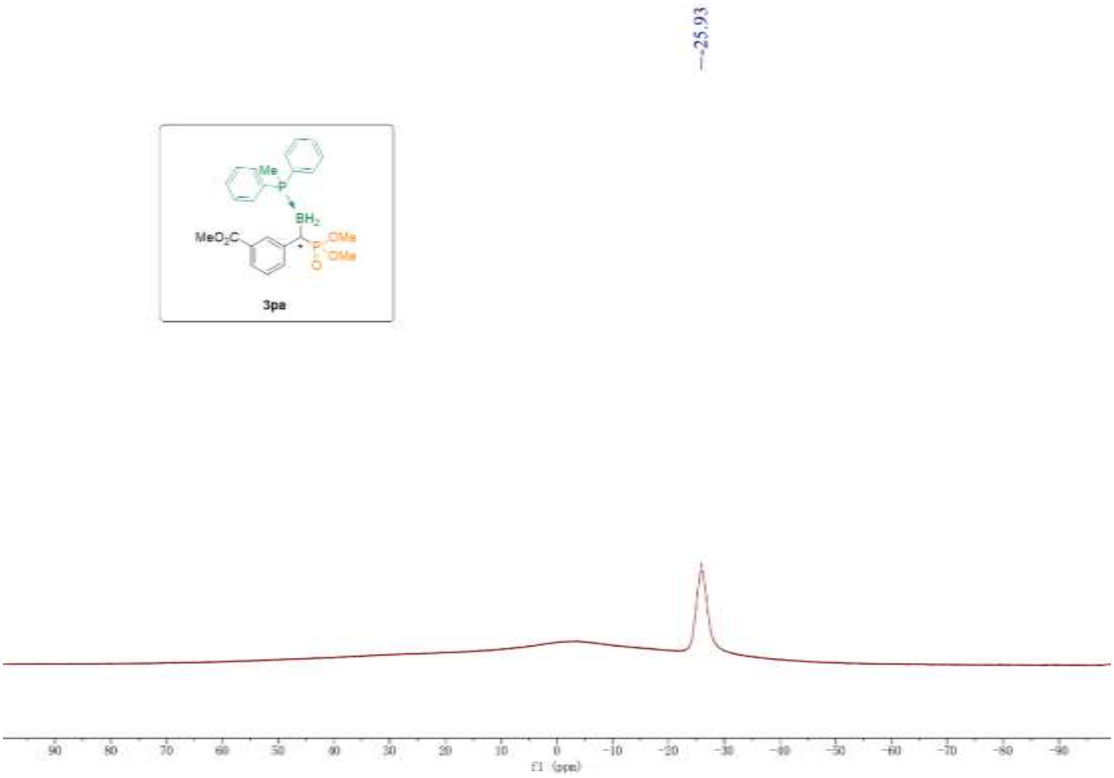

^{31}P NMR (162 MHz, Chloroform-*d*)

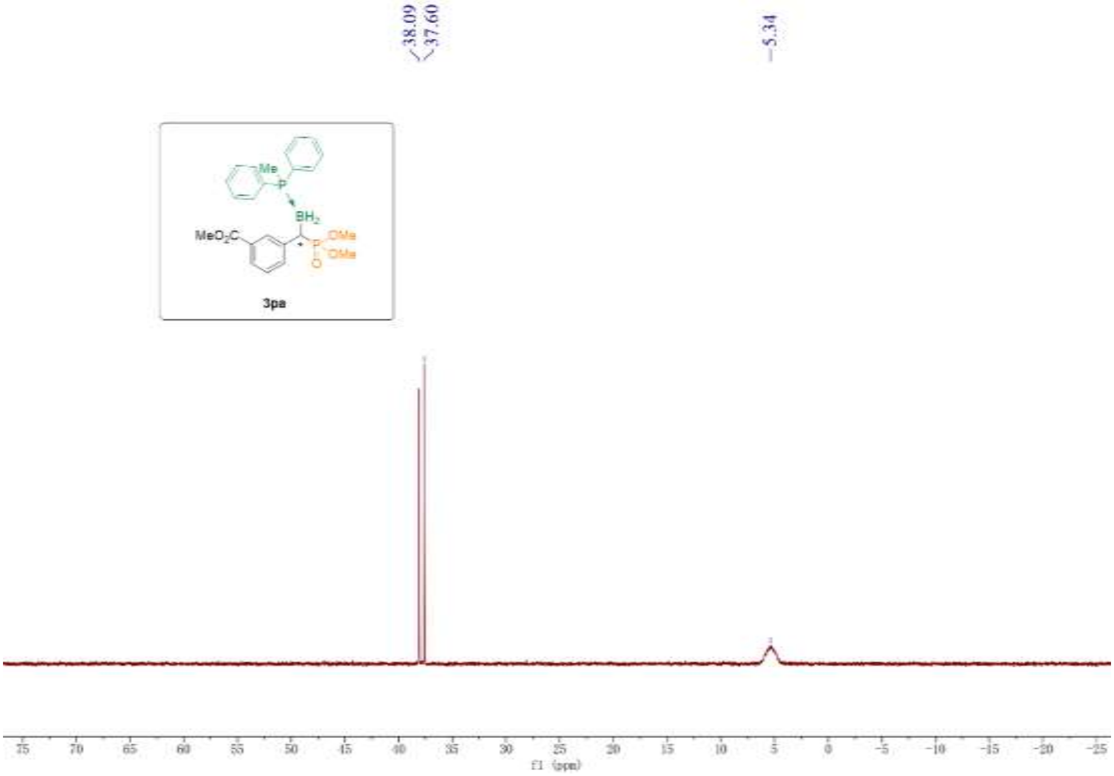

(S)-dimethyl(((methyldiphenylphosphane)boryl)(3-iodophenyl)methyl)phosphonate(3qa)

^{1}H NMR (400 MHz, Chloroform-*d*)

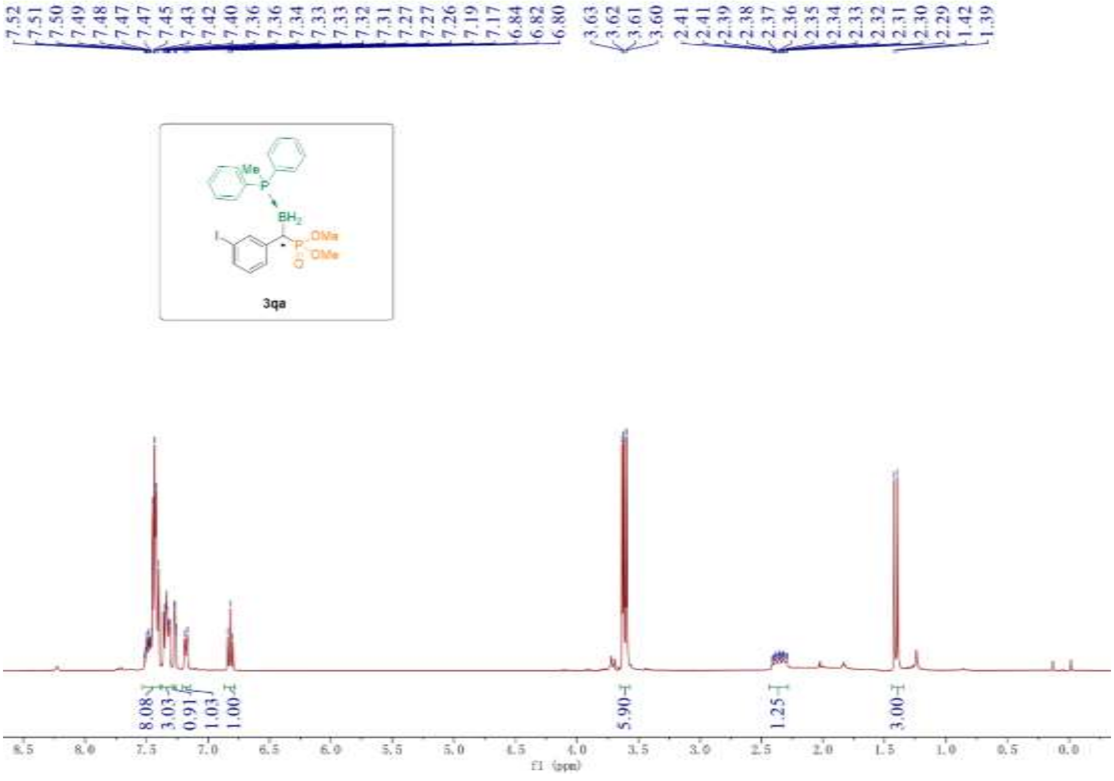

^{13}C NMR (101 MHz, Chloroform-*d*)

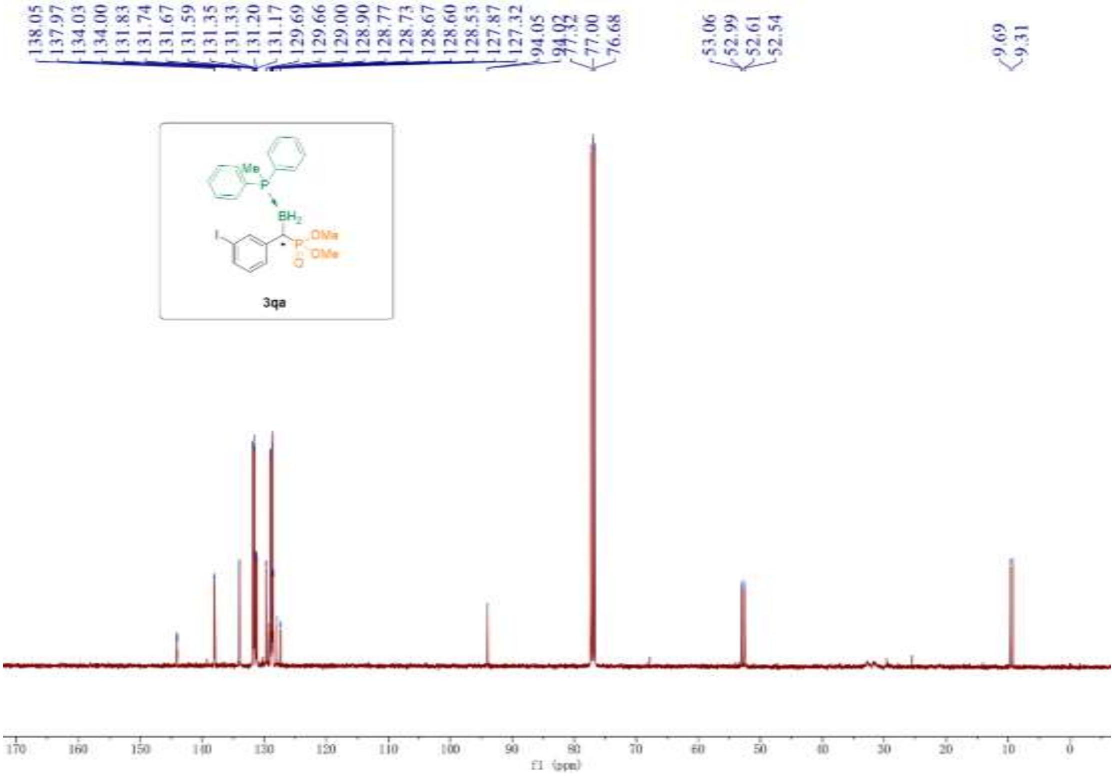

^{11}B NMR (128 MHz, Chloroform-*d*)

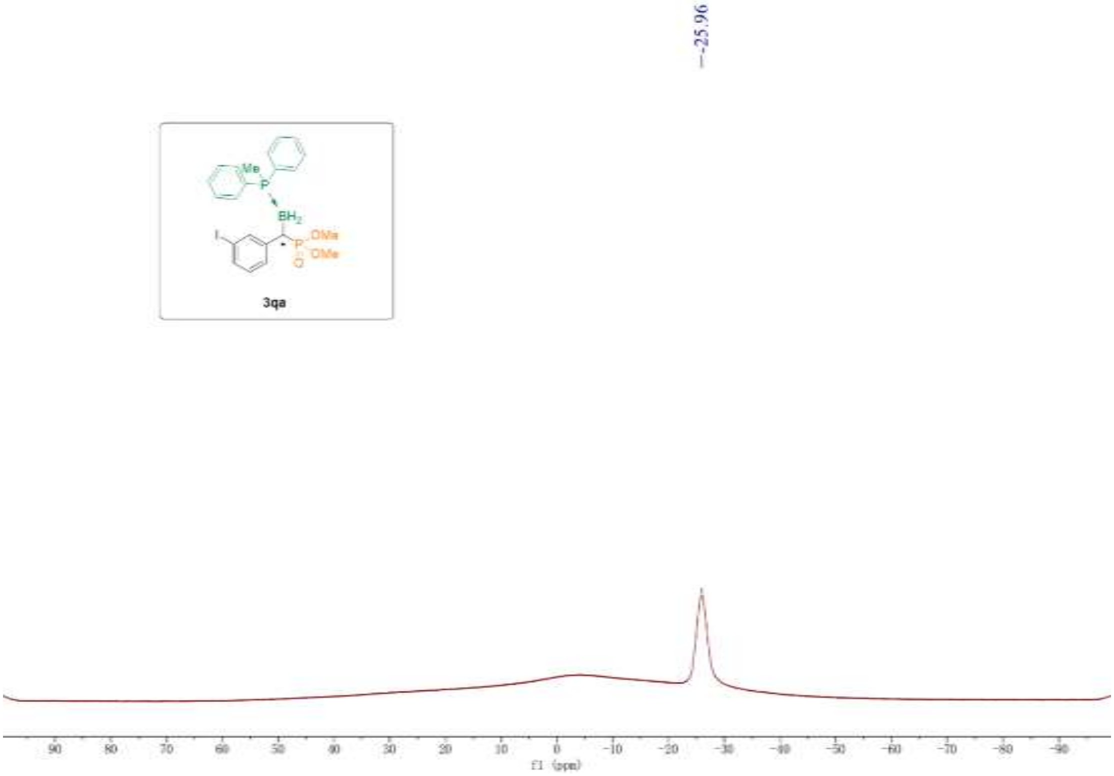

^{31}P NMR (162 MHz, Chloroform-*d*)

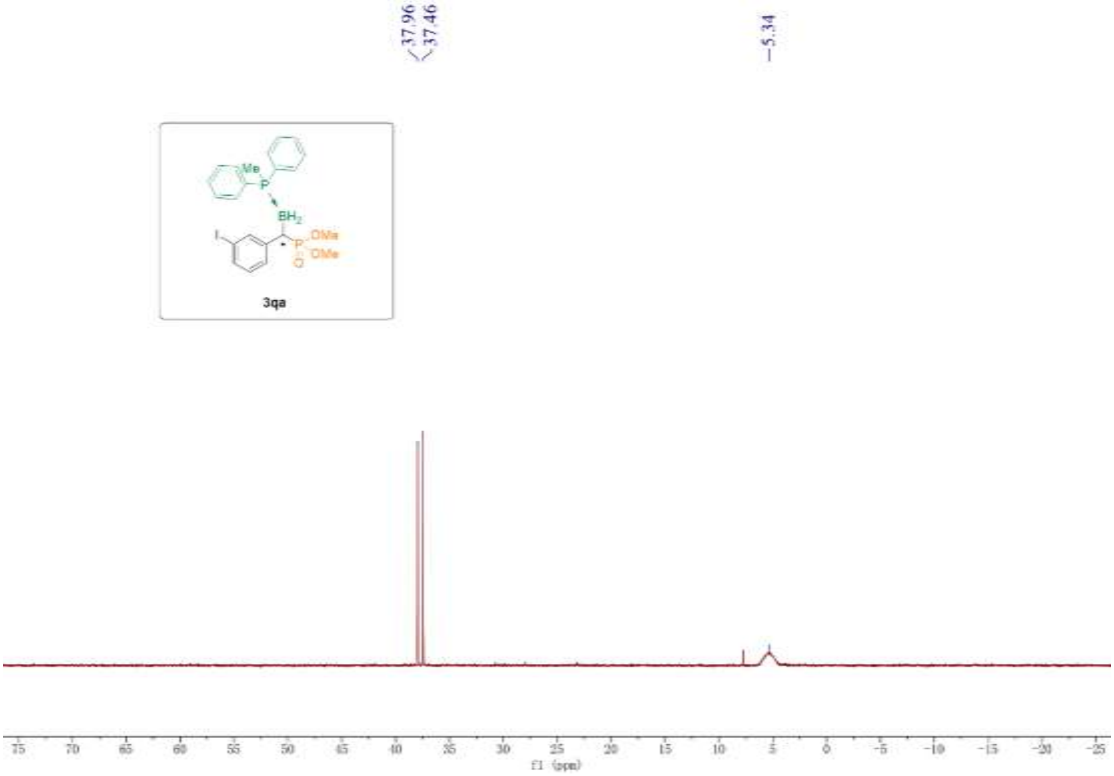

(S)-dimethyl(((methyldiphenylphosphane)boryl)(2-fluorophenyl)methyl)phosphonate(3ra)

^{1}H NMR (500 MHz, Chloroform-*d*)

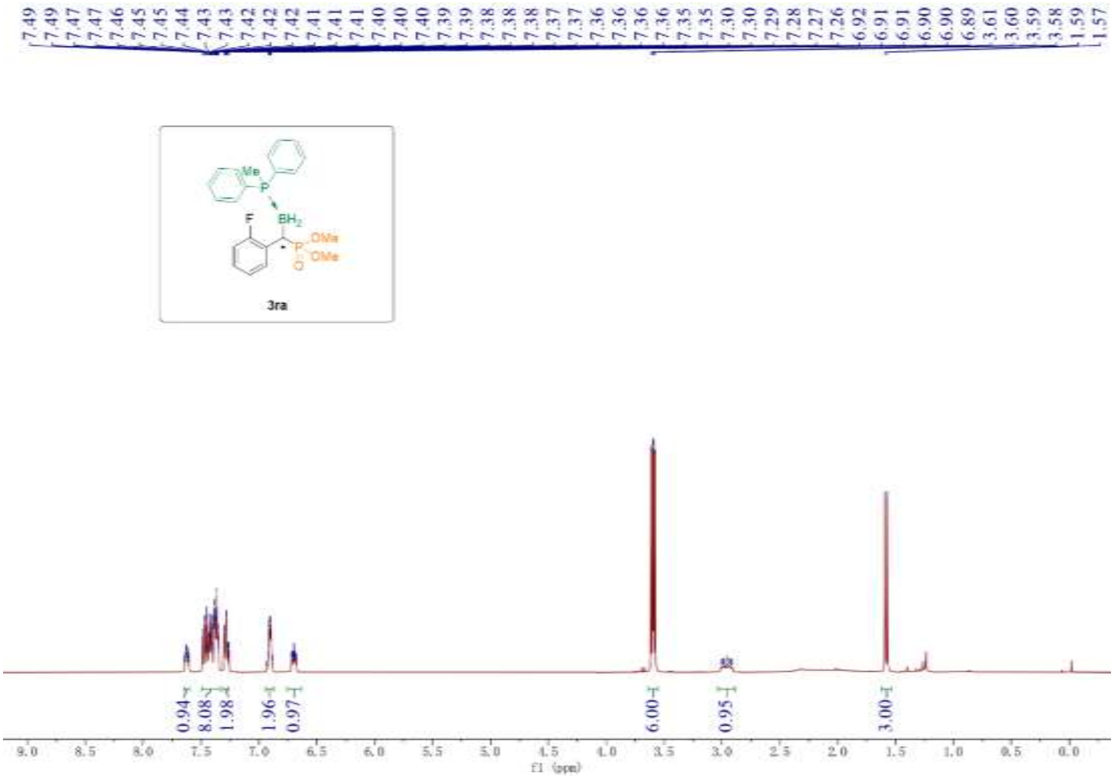

^{13}C NMR (101 MHz, Chloroform-*d*)

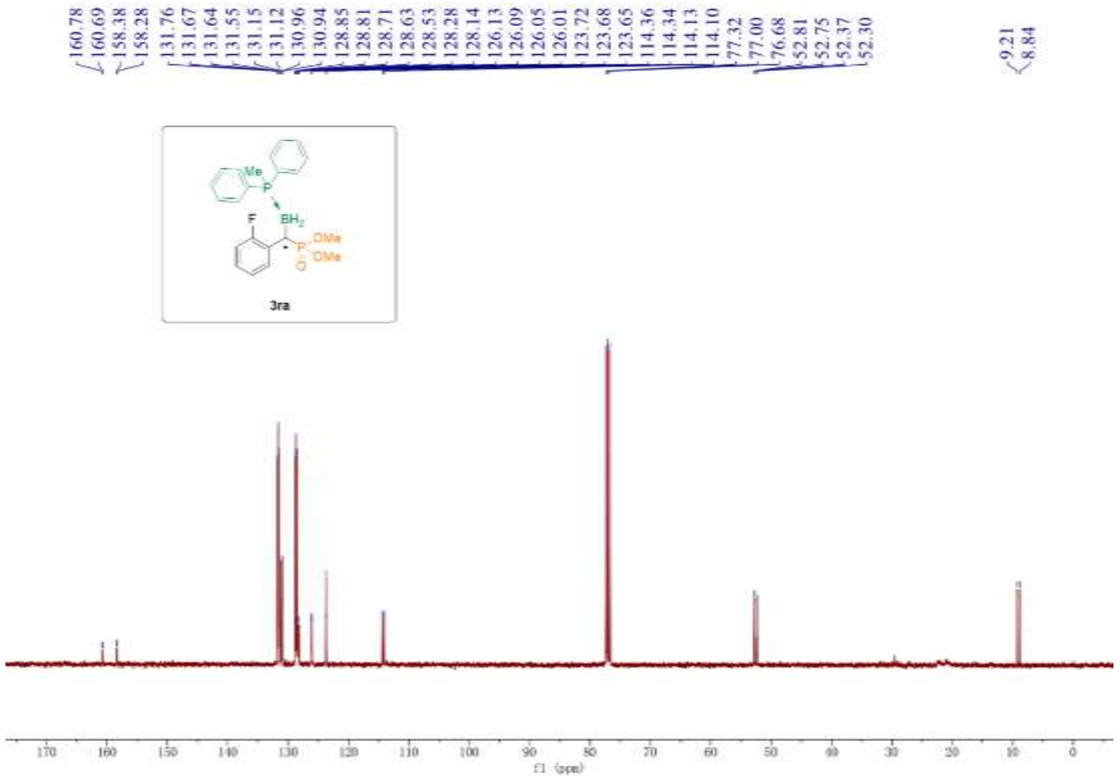

^{11}B NMR (160 MHz, Chloroform-*d*)

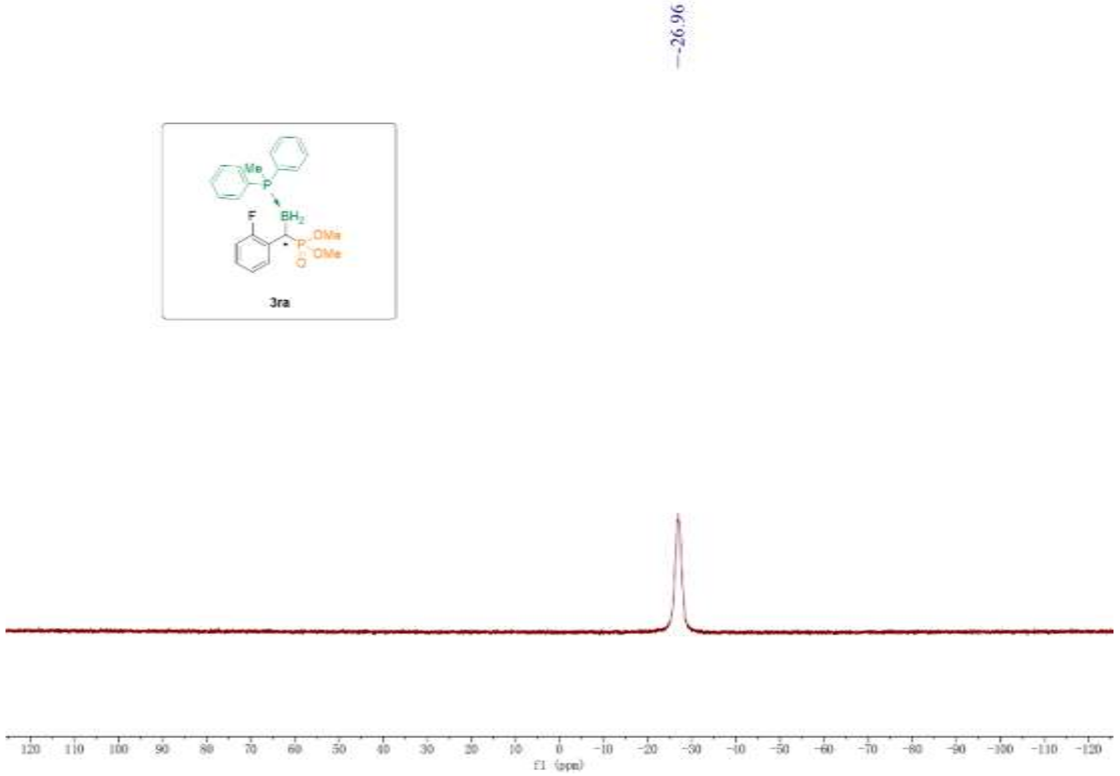

^{31}P NMR (202 MHz, Chloroform-*d*)

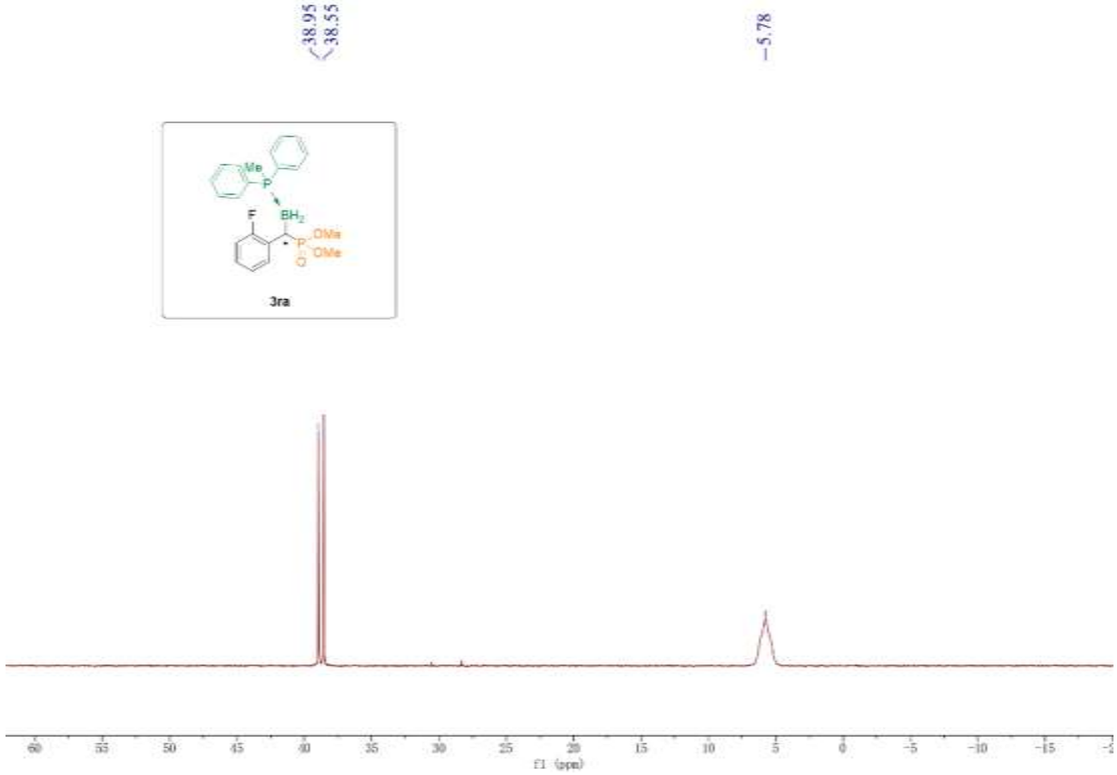

^{19}F NMR (471 MHz, Chloroform-*d*)

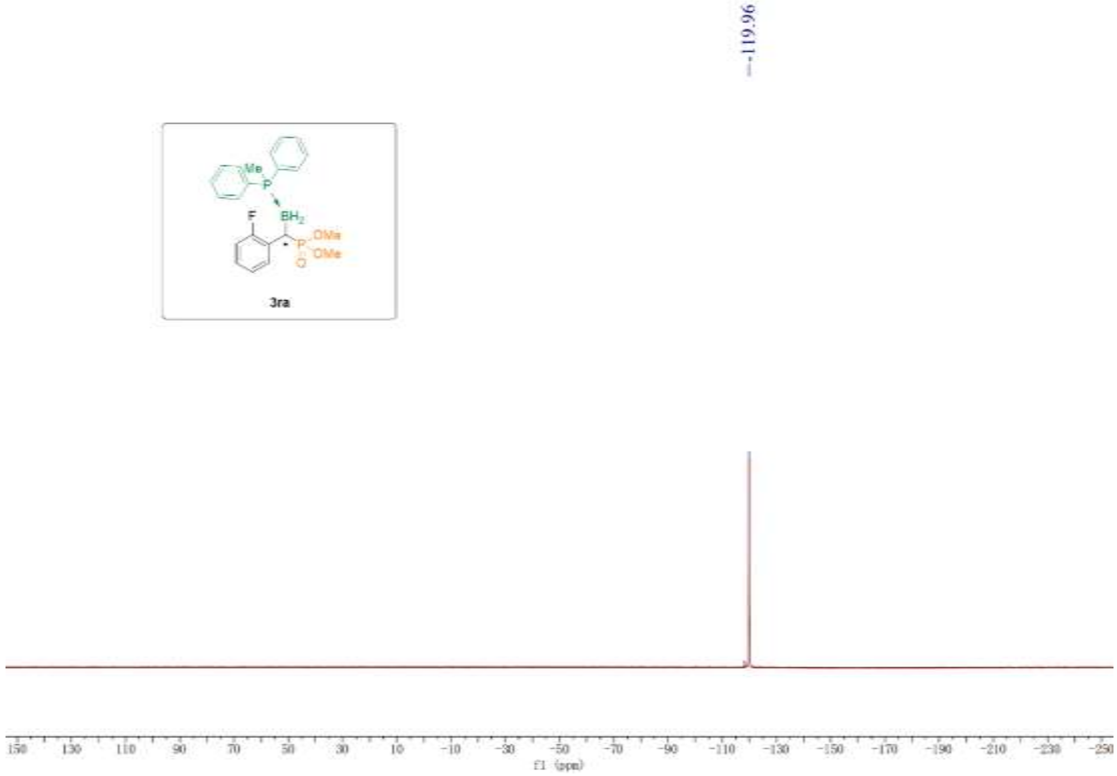

(S)-dimethyl(((methyldiphenylphosphane)boryl)(3-chloro-4-fluorophenyl)methyl) phosphonate(3sa)

^{1}H NMR (400 MHz, Chloroform-*d*)

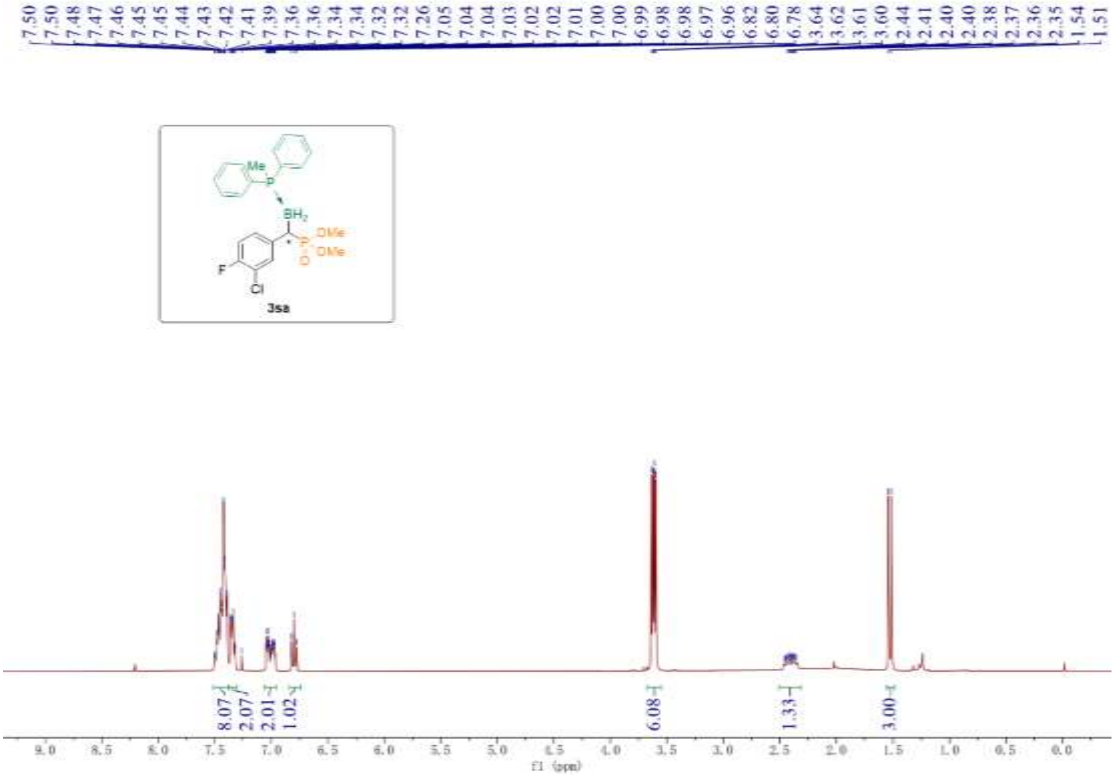

^{13}C NMR (101 MHz, Chloroform-*d*)

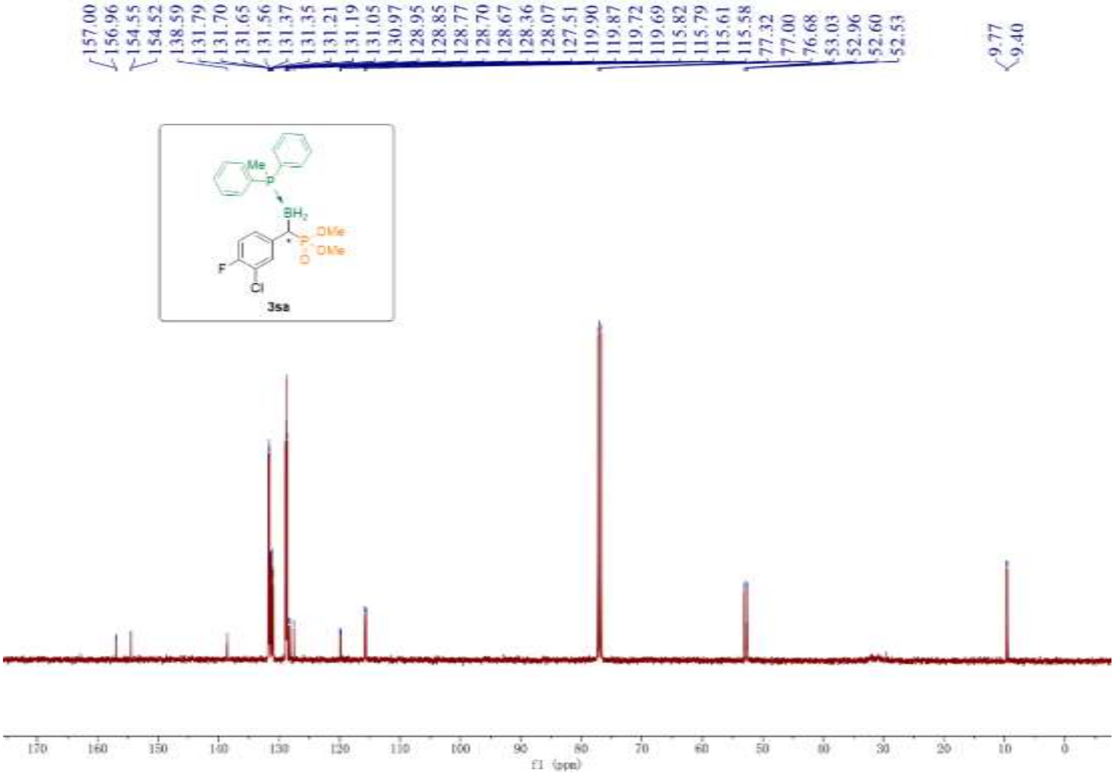

^{11}B NMR (128 MHz, Chloroform-*d*)

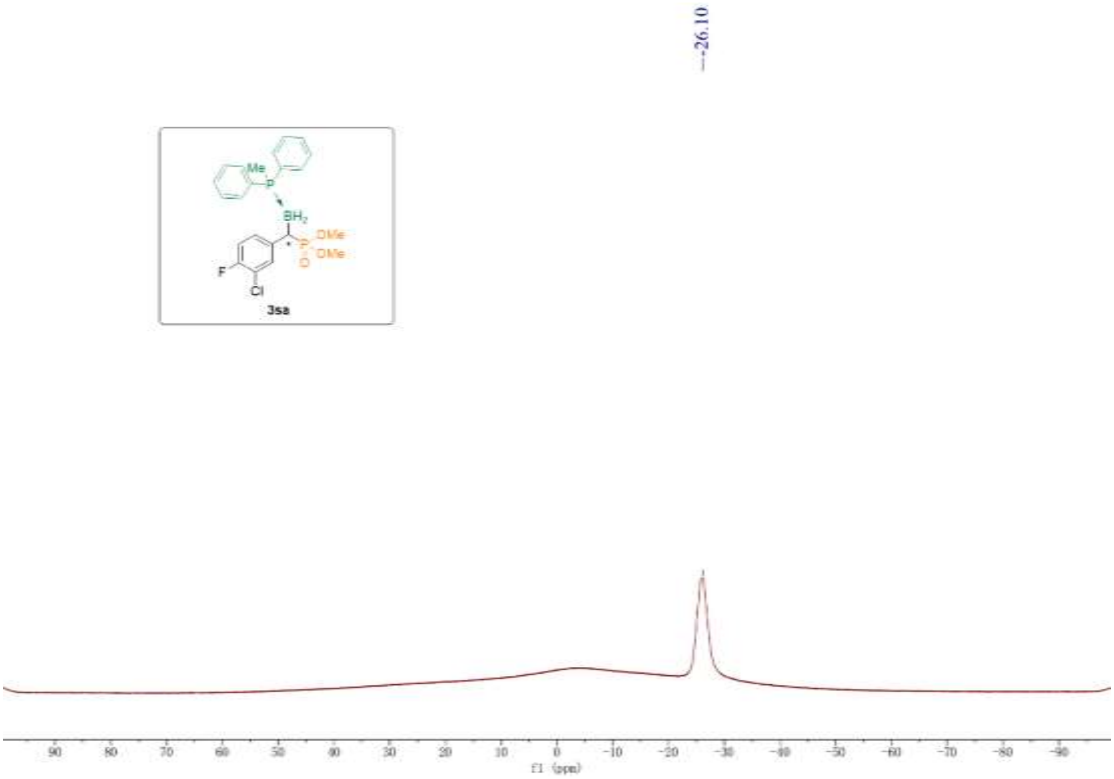

^{31}P NMR (162 MHz, Chloroform-*d*)

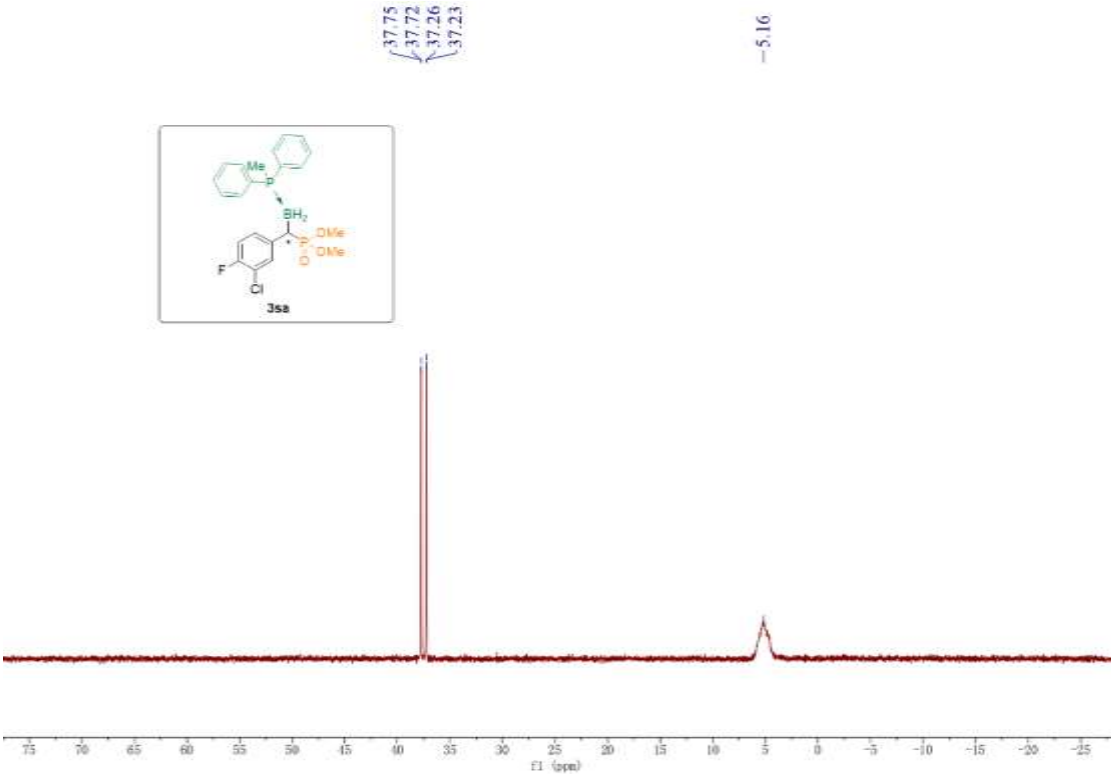

^{19}F NMR (376 MHz, Chloroform-*d*)

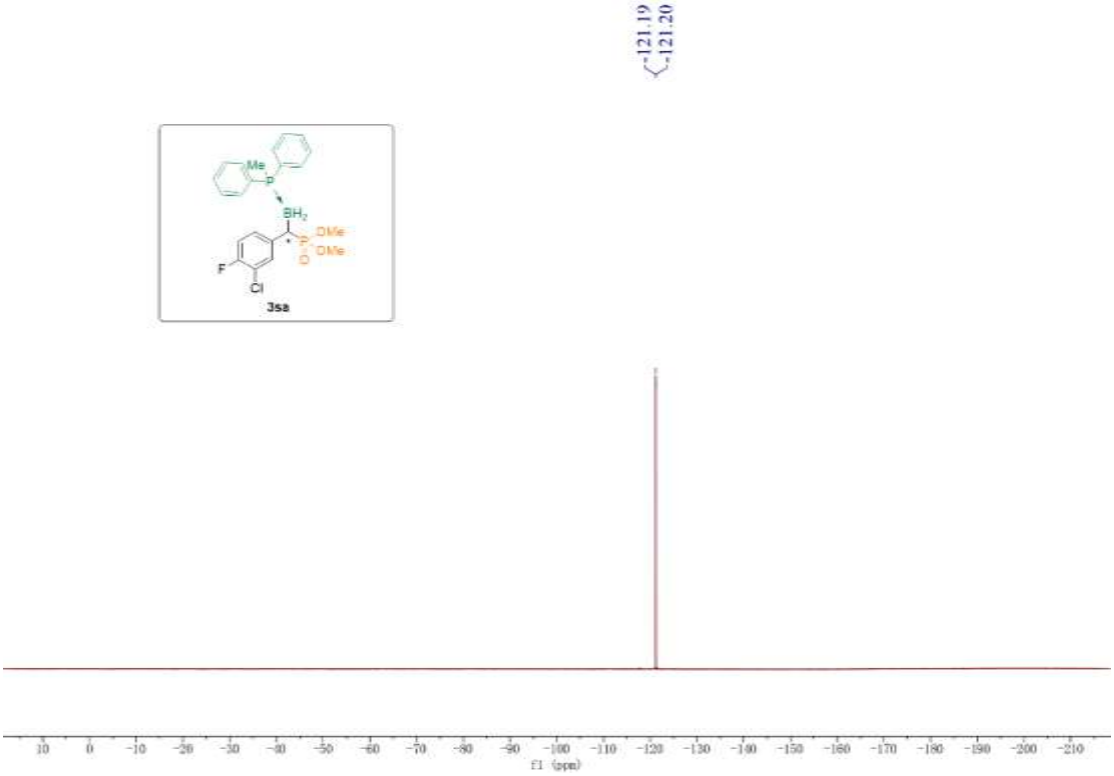

(S)-dimethyl(((methyldiphenylphosphane)boryl)(4-methyl-3-nitrophenyl)methyl)

phosphonate(3ta)

^{1}H NMR (400 MHz, Chloroform-*d*)

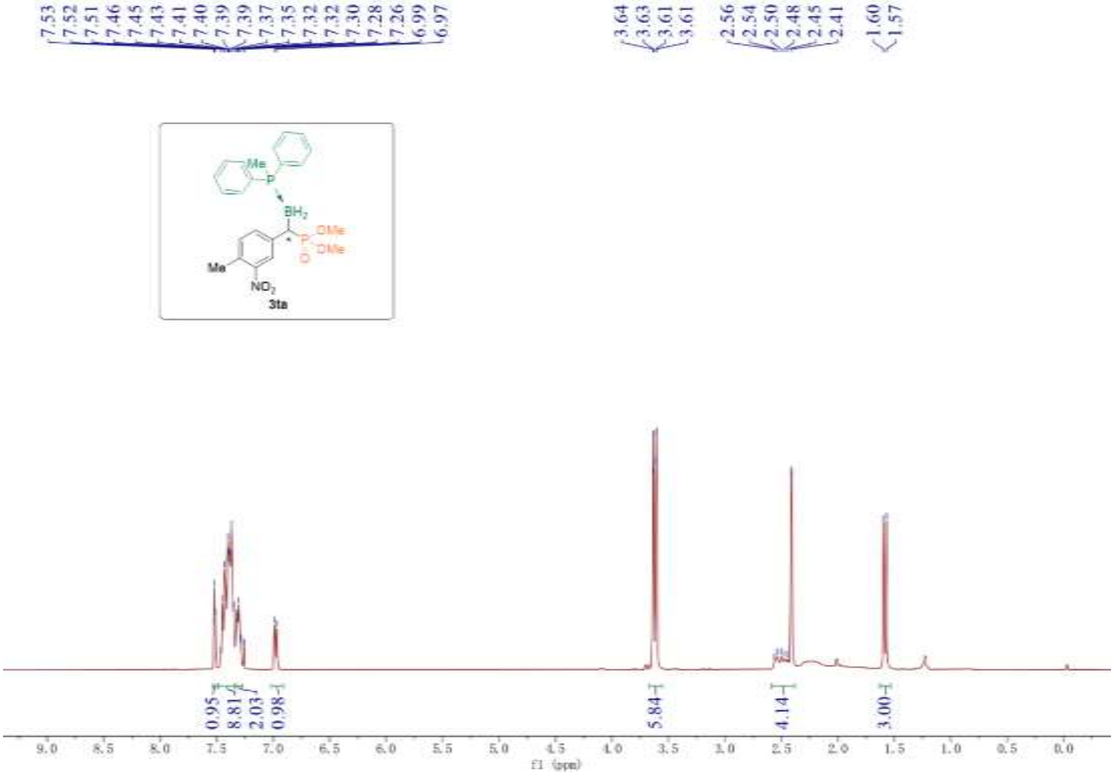

^{13}C NMR (101 MHz, Chloroform-*d*)

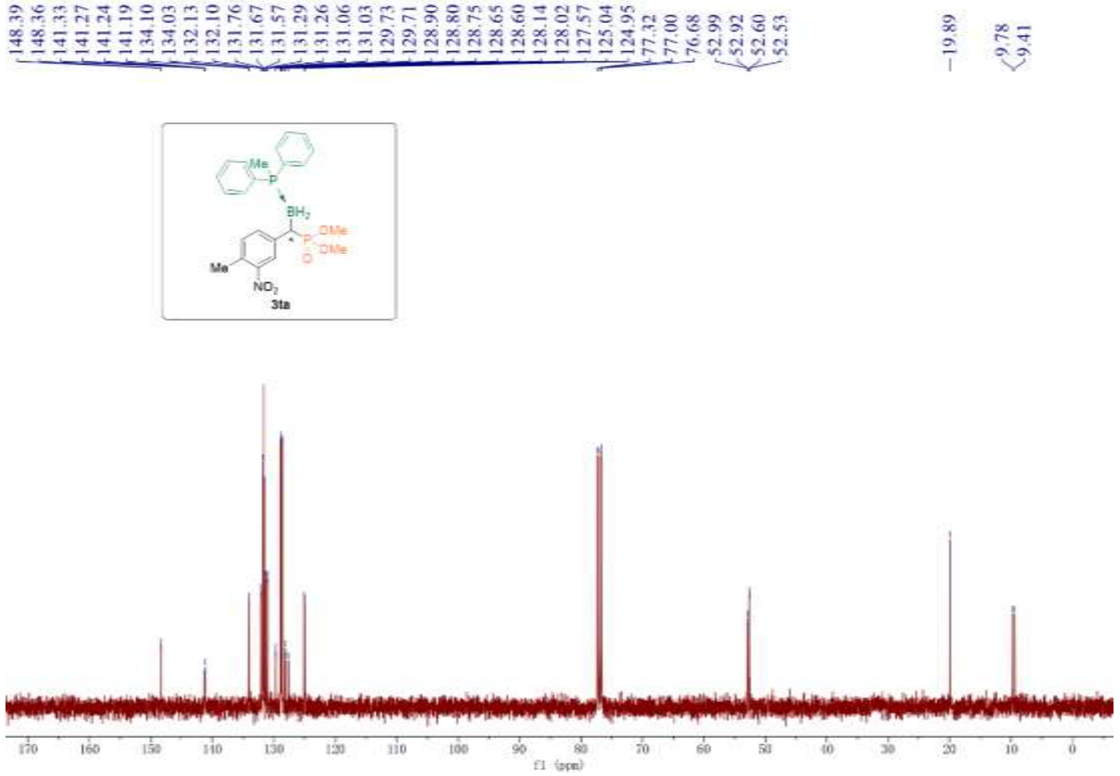

^{11}B NMR (128 MHz, Chloroform-*d*)

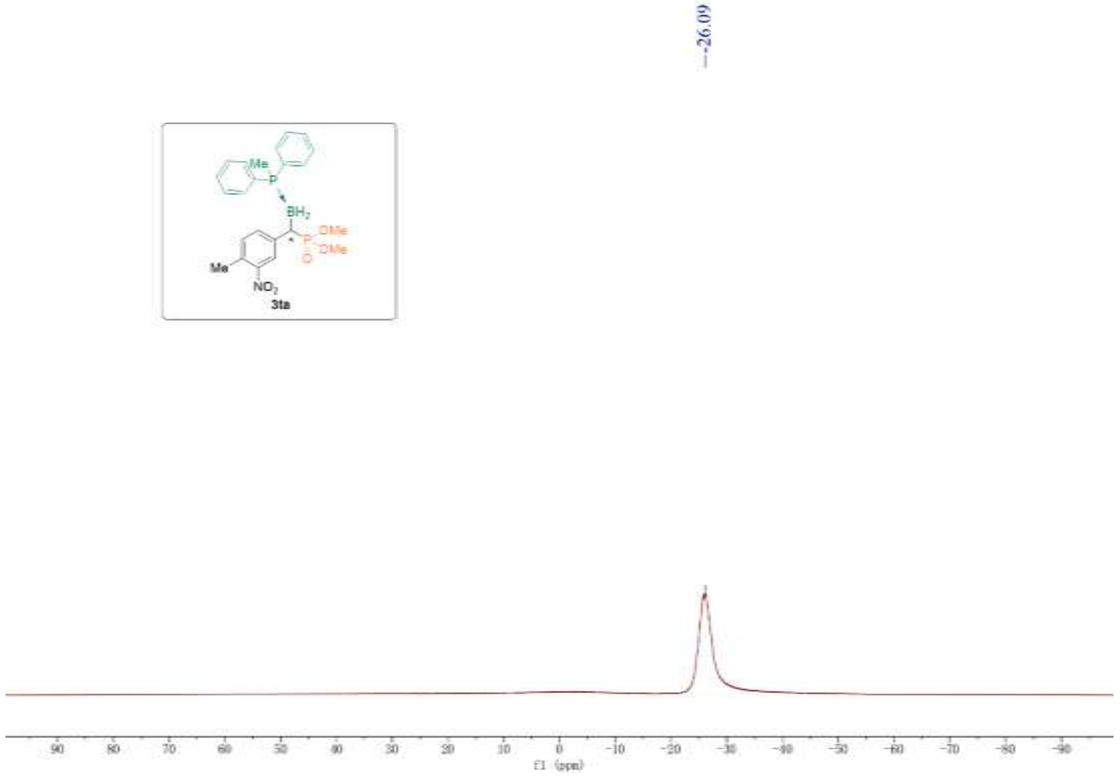

^{31}P NMR (162 MHz, Chloroform-*d*)

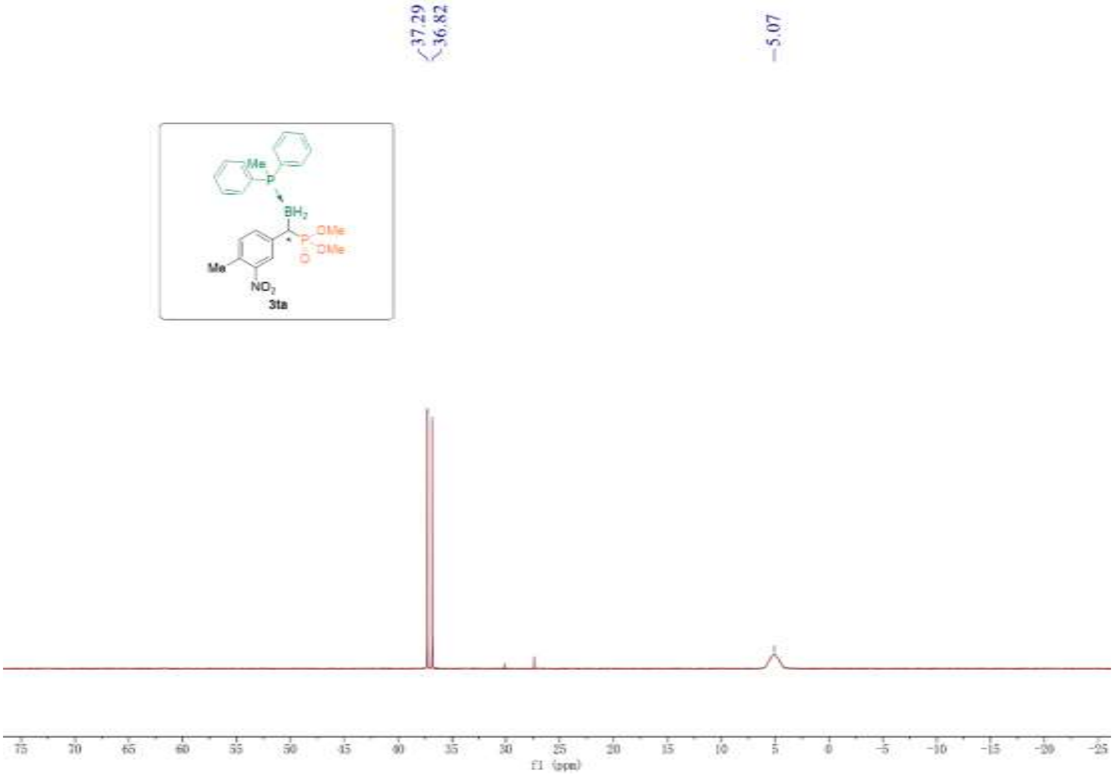

(S)-dimethyl(((methyldiphenylphosphane)boryl)(3,5-dimethoxyphenyl)methyl)

phosphonate(3ua)

^{1}H NMR (400 MHz, Chloroform-*d*)

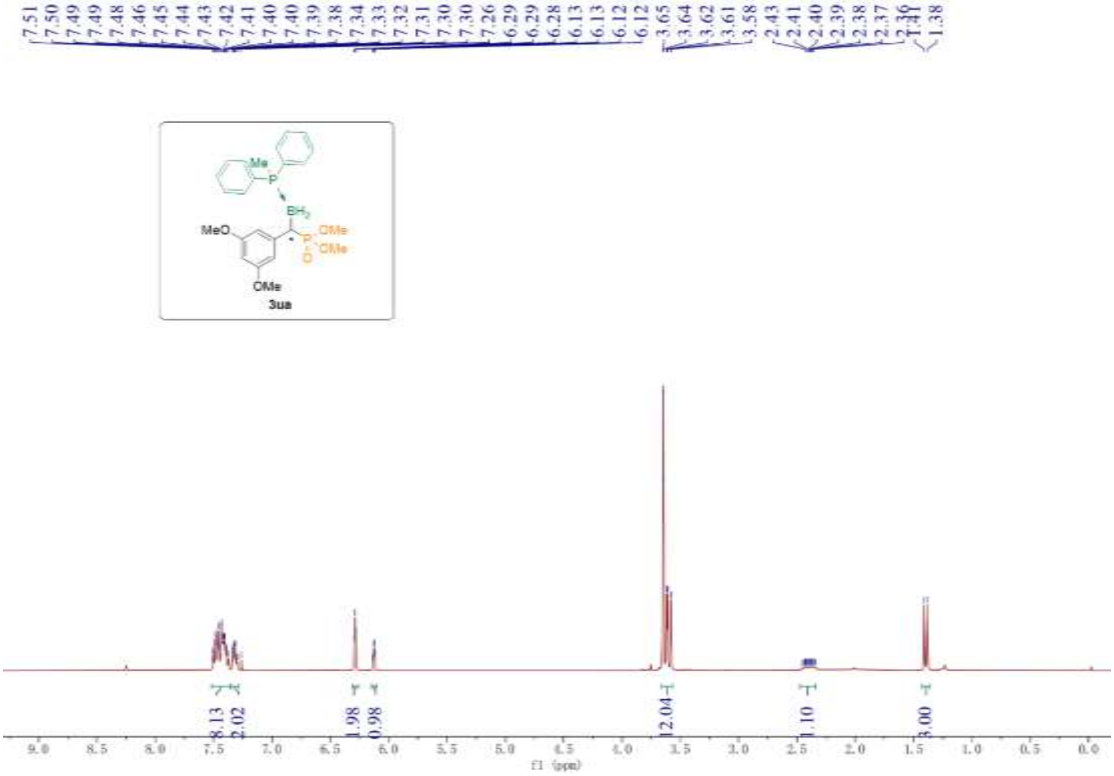

^{13}C NMR (101 MHz, Chloroform-*d*)

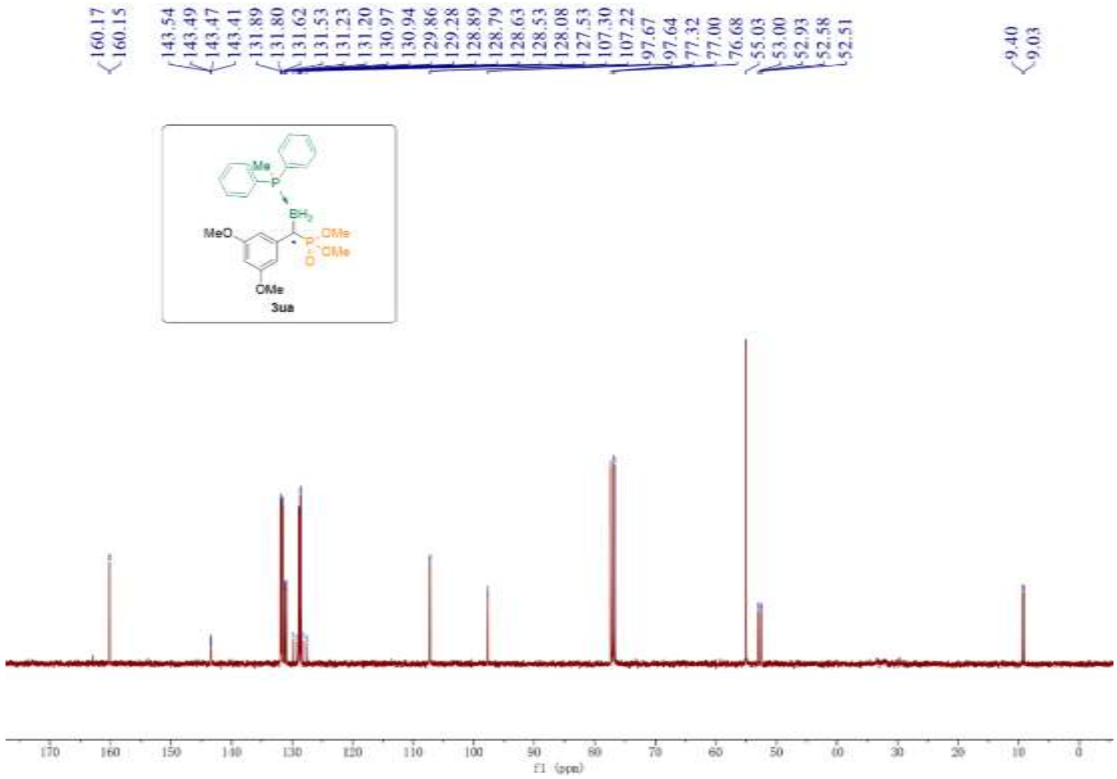

^{11}B NMR (128 MHz, Chloroform-*d*)

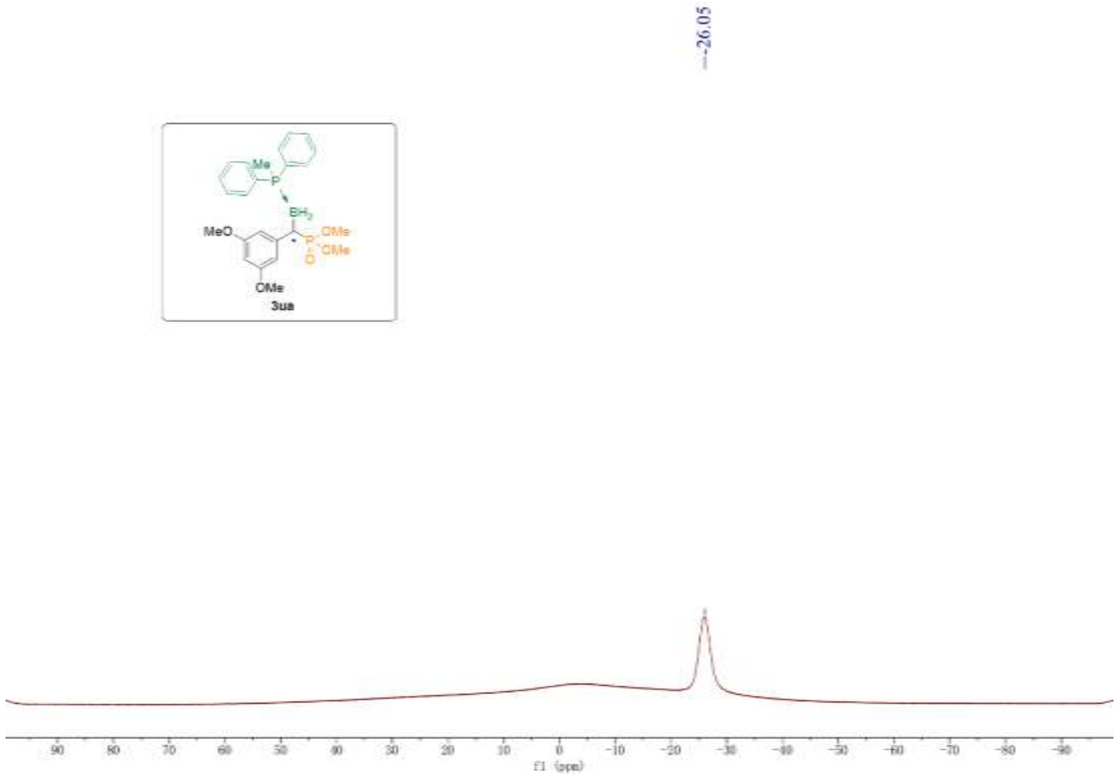

^{31}P NMR (162 MHz, Chloroform-*d*)

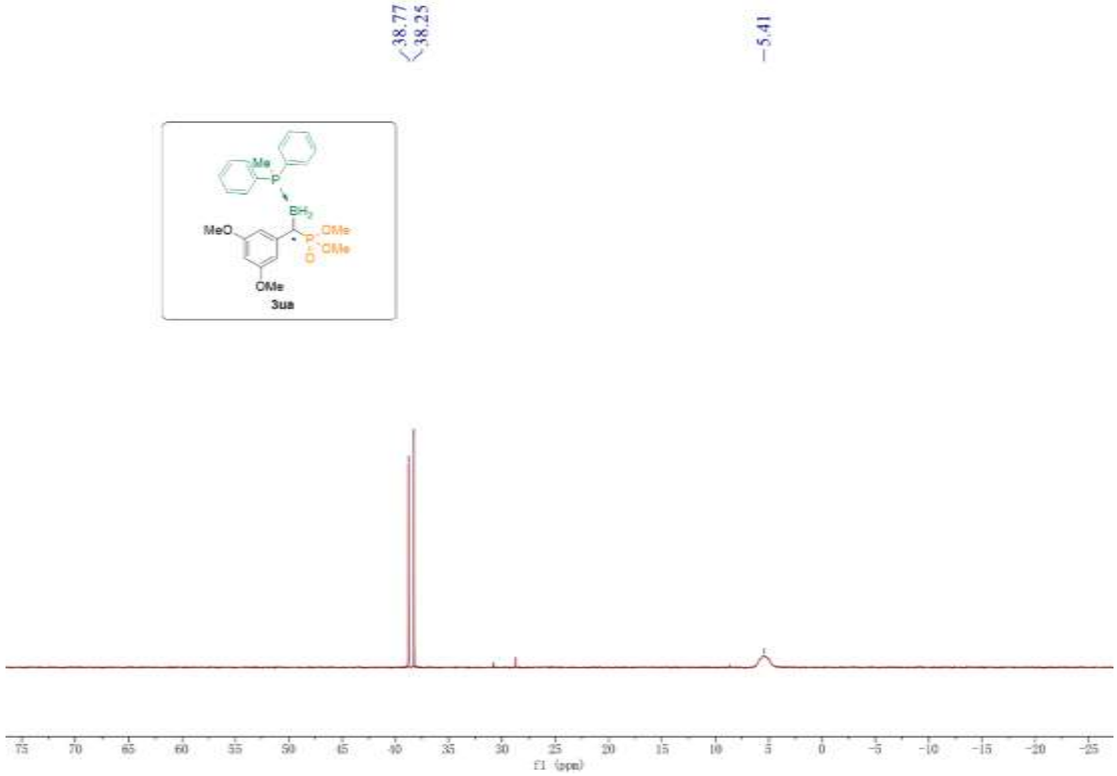

(S)-dimethyl(((methyldiphenylphosphane)boryl)(9H-fluoren-2-yl)methyl)phosphonate(3va)

^{1}H NMR (400 MHz, Chloroform-*d*)

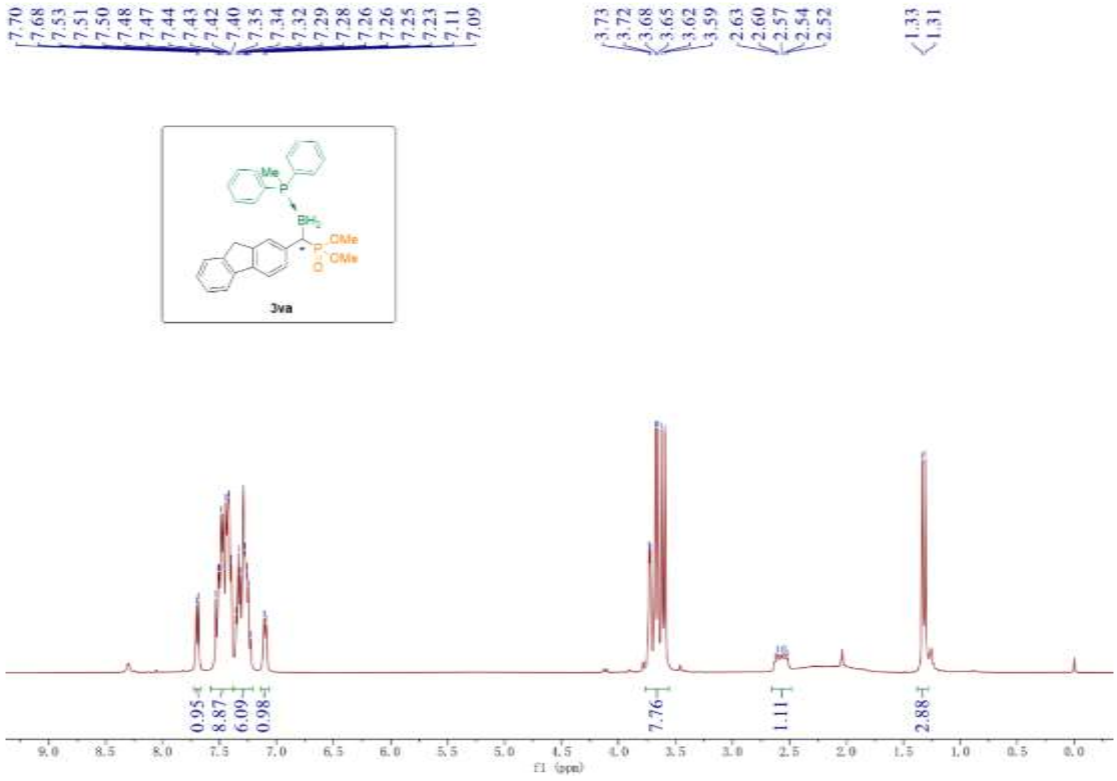

^{13}C NMR (101 MHz, Chloroform-*d*)

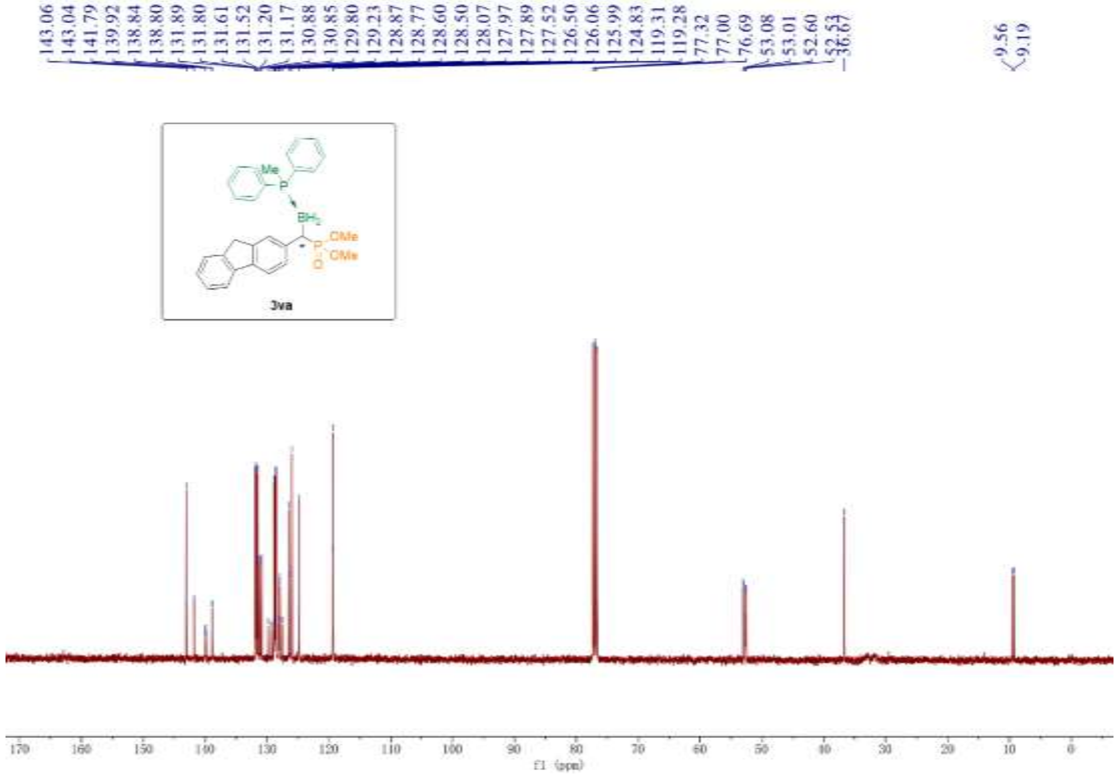

^{11}B NMR (128 MHz, Chloroform-*d*)

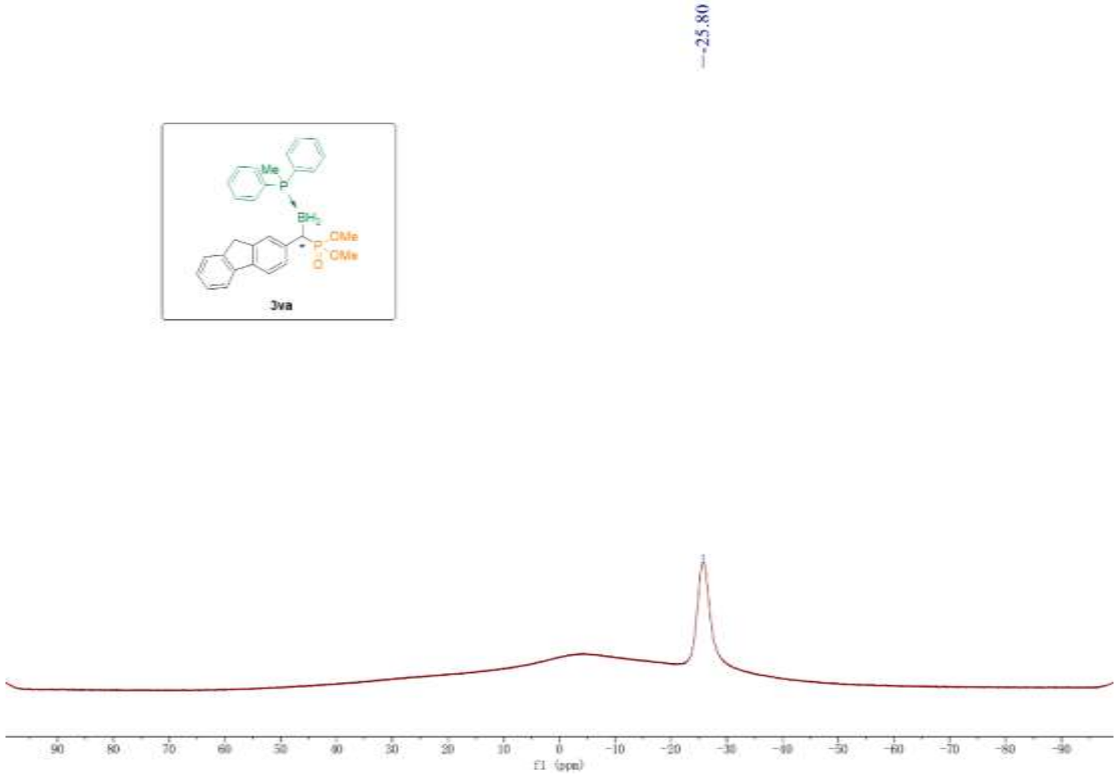

^{31}P NMR (162 MHz, Chloroform-*d*)

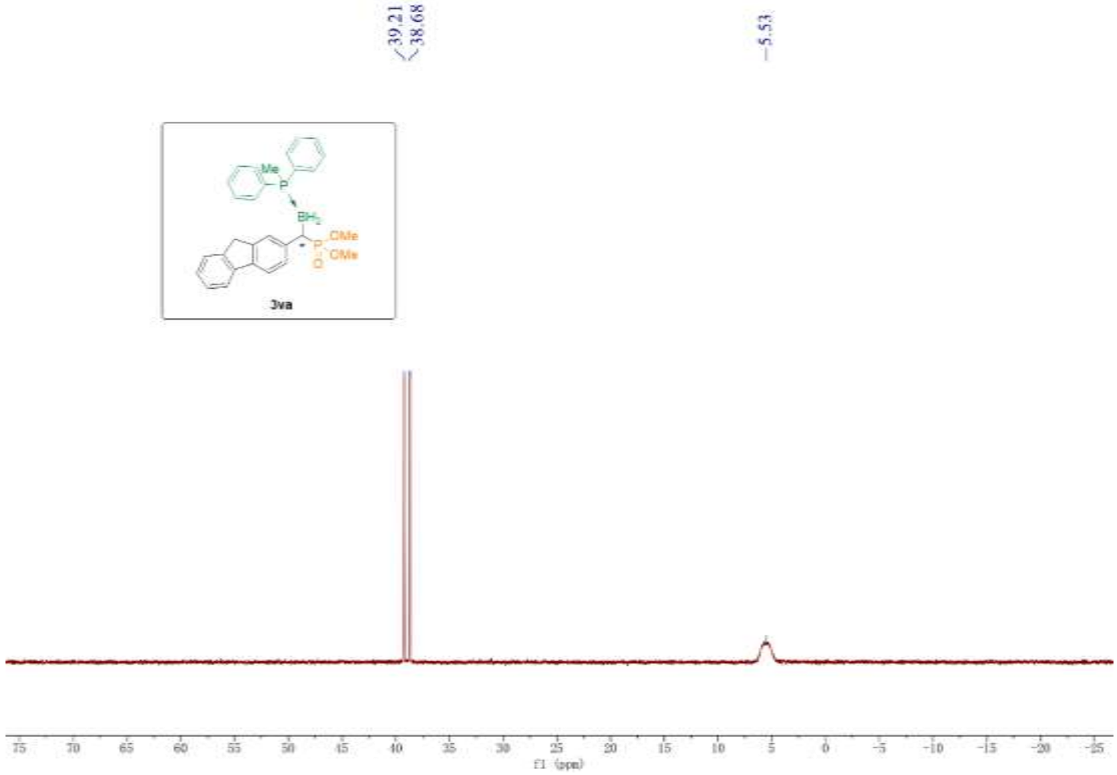

(S)-dimethyl(((methyldiphenylphosphane)boryl)(1H-indol-5-yl)methyl)phosphonate(3wa)

^{1}H NMR (400 MHz, Chloroform-*d*)

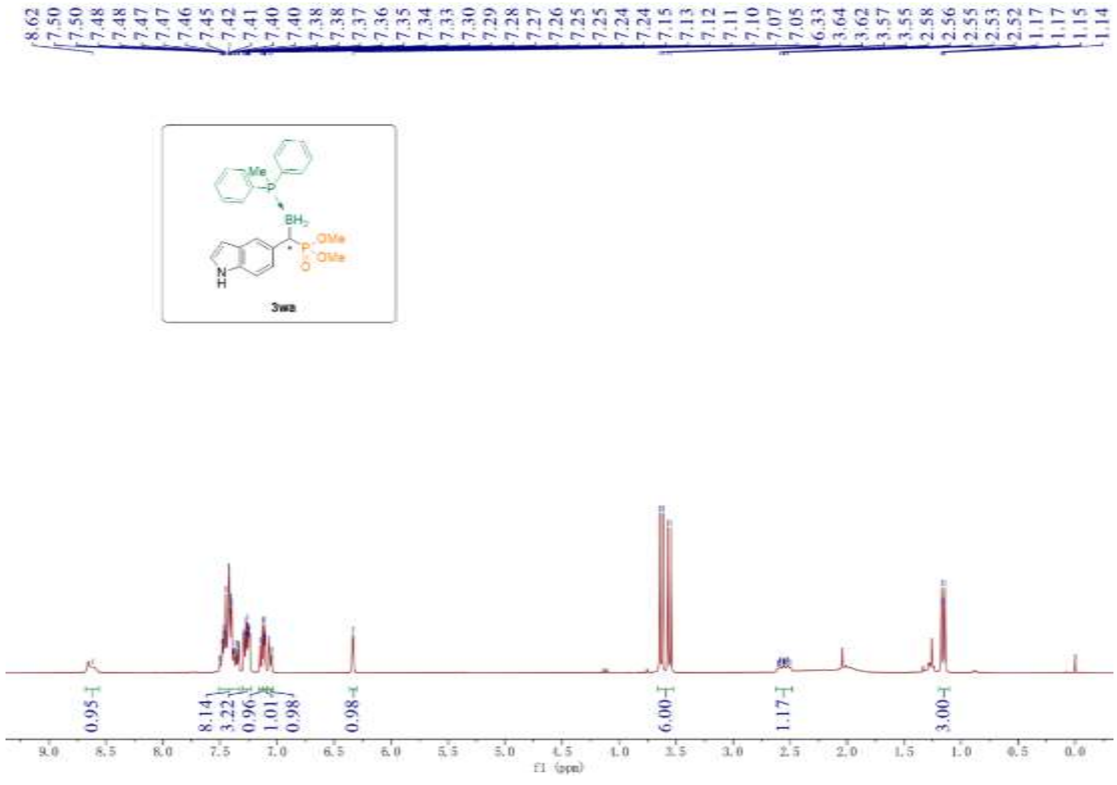

^{13}C NMR (101 MHz, Chloroform-*d*)

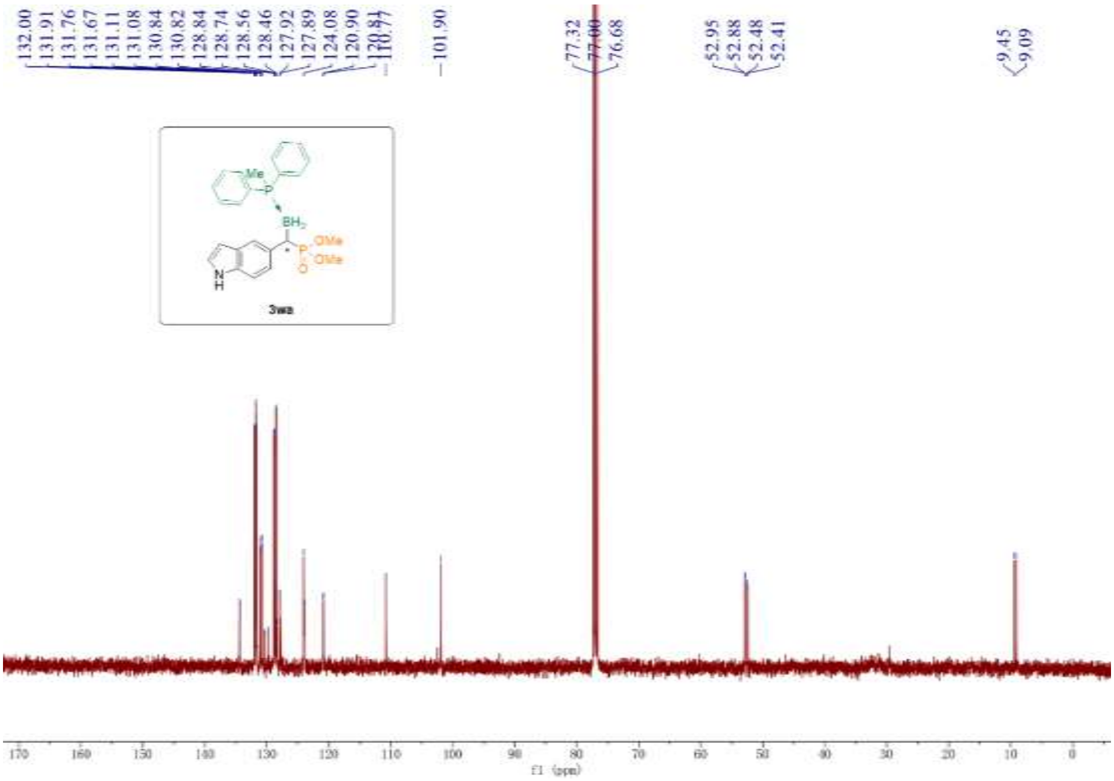

^{11}B NMR (128 MHz, Chloroform-*d*)

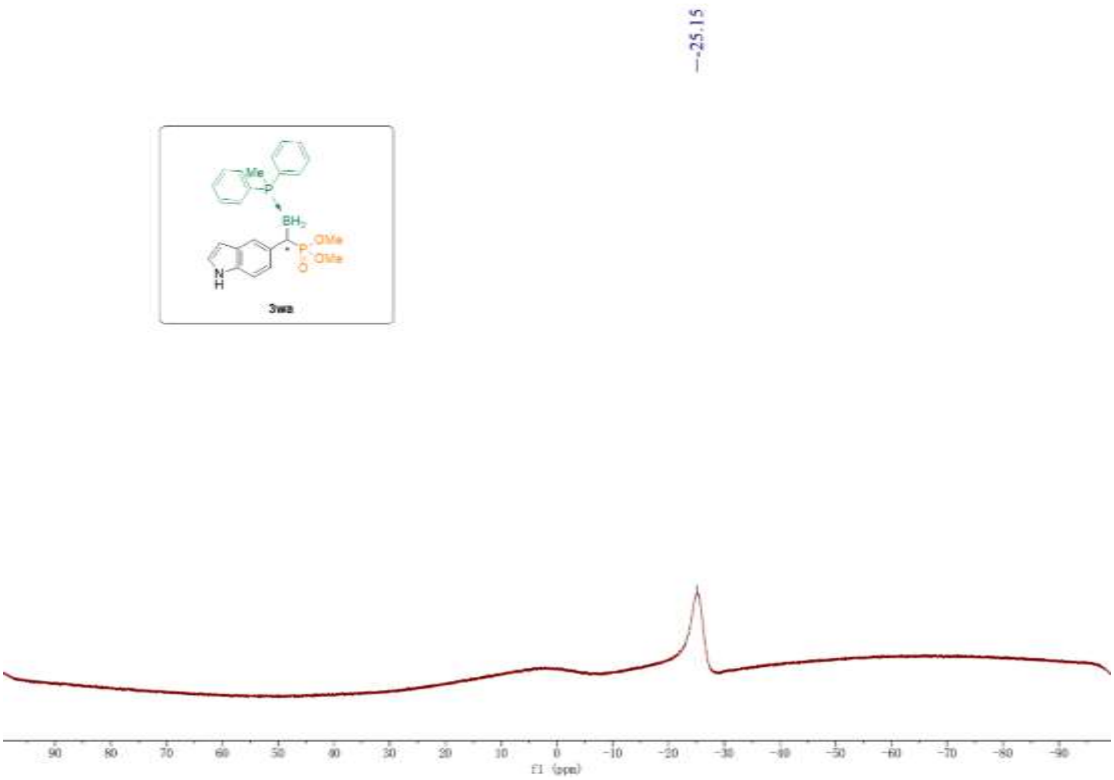

^{31}P NMR (162 MHz, Chloroform-*d*)

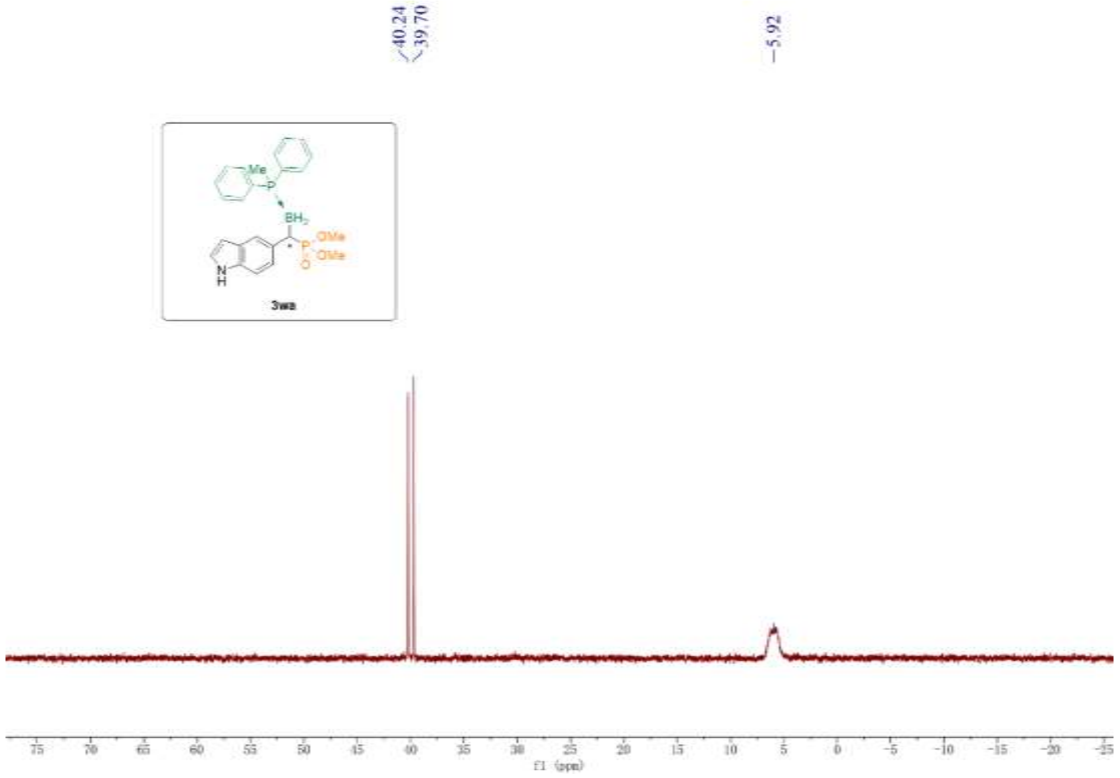

(S)-dimethyl(((methyldiphenylphosphane)boryl)(benzo[d][1,3]dioxol-5-yl)methyl)

phosphonate(3xa)

^{1}H NMR (500 MHz, Chloroform-*d*)

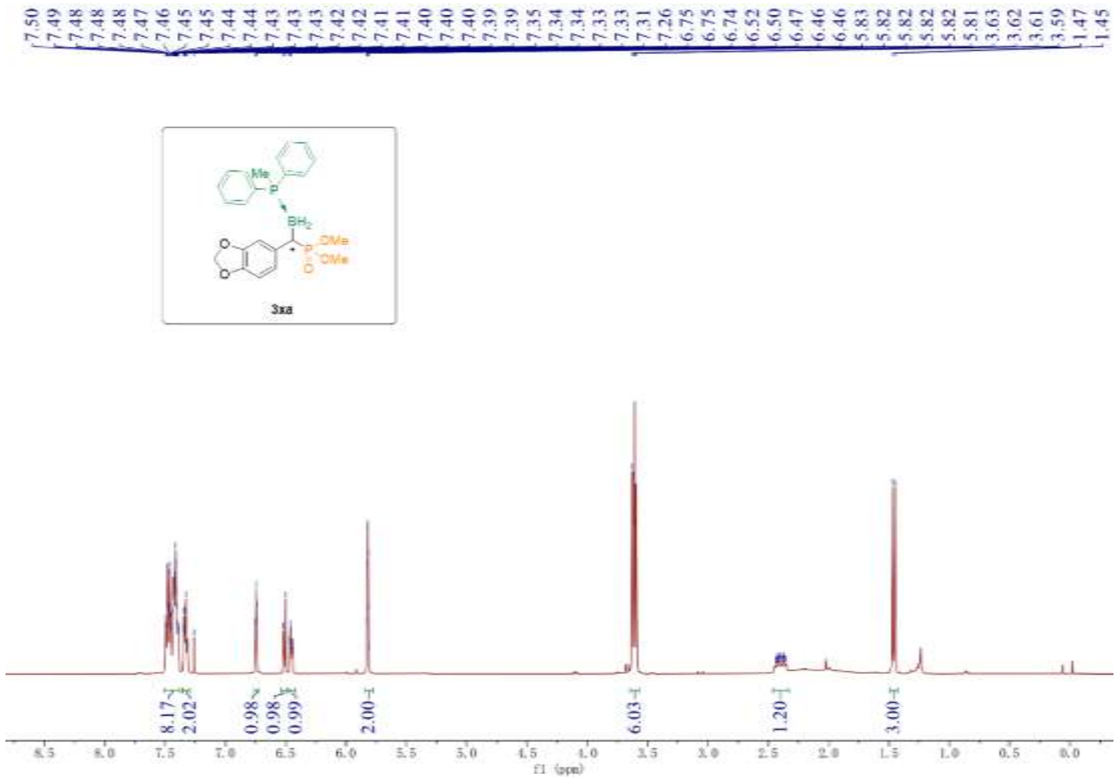

^{13}C NMR (126 MHz, Chloroform-*d*)

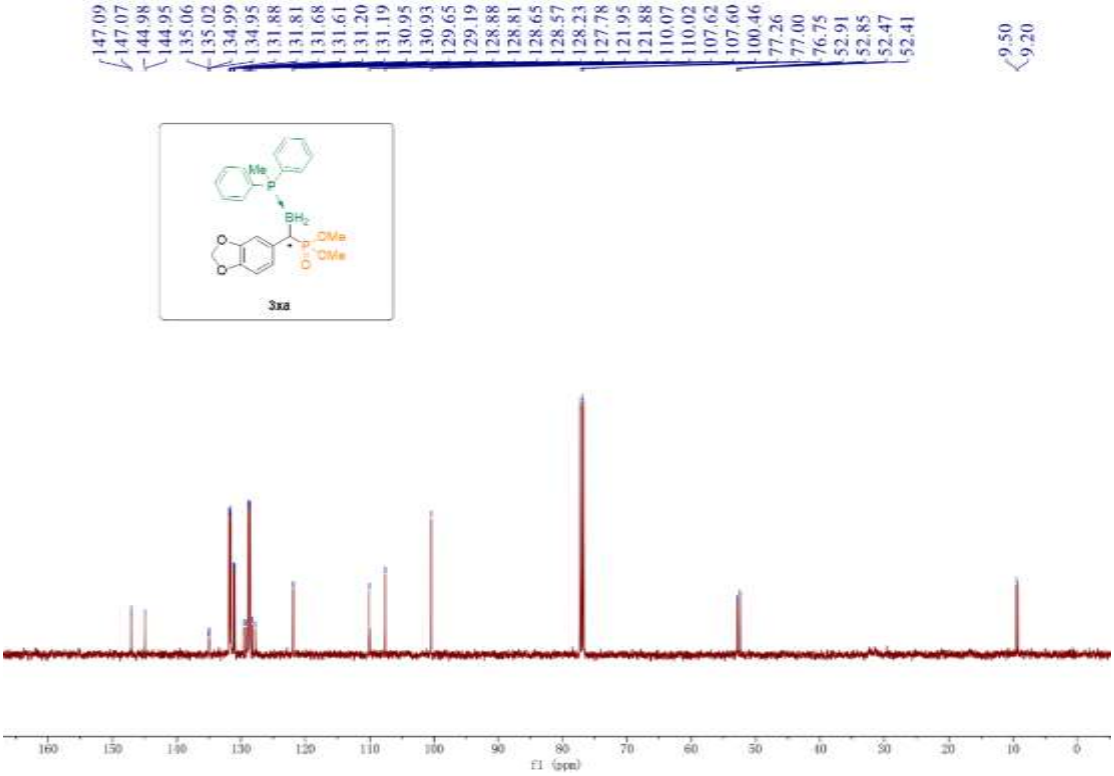

^{11}B NMR (160 MHz, Chloroform-*d*)

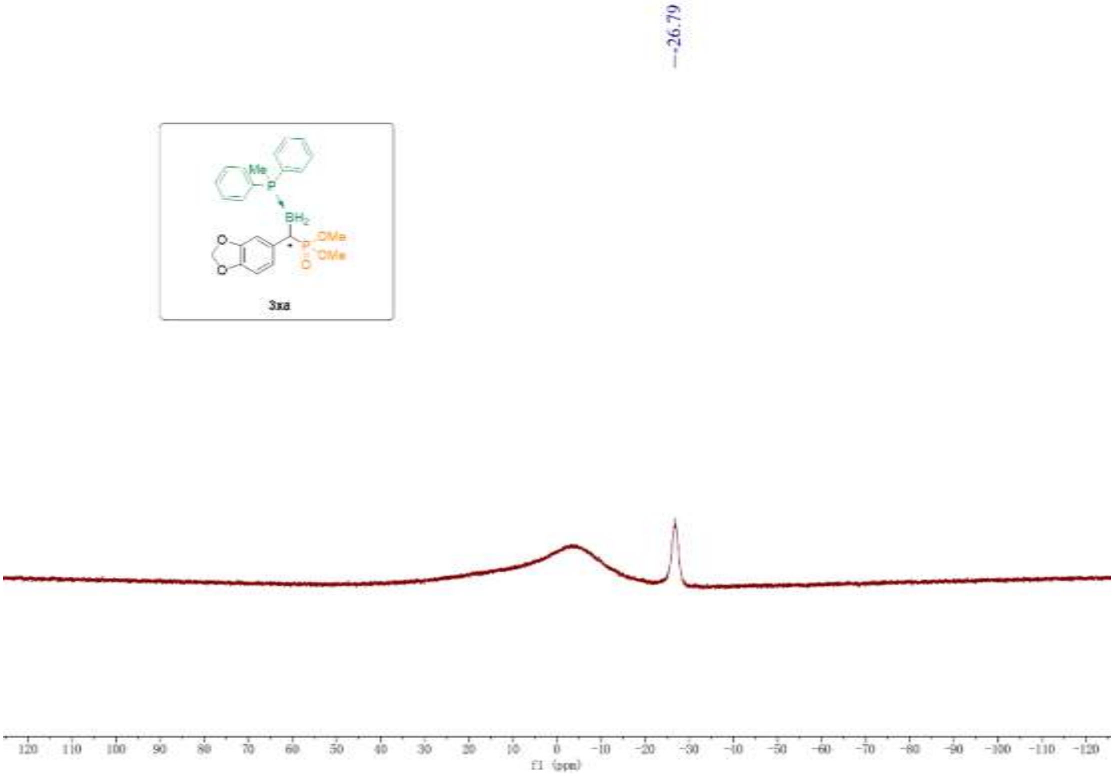

^{31}P NMR (202 MHz, Chloroform-*d*)

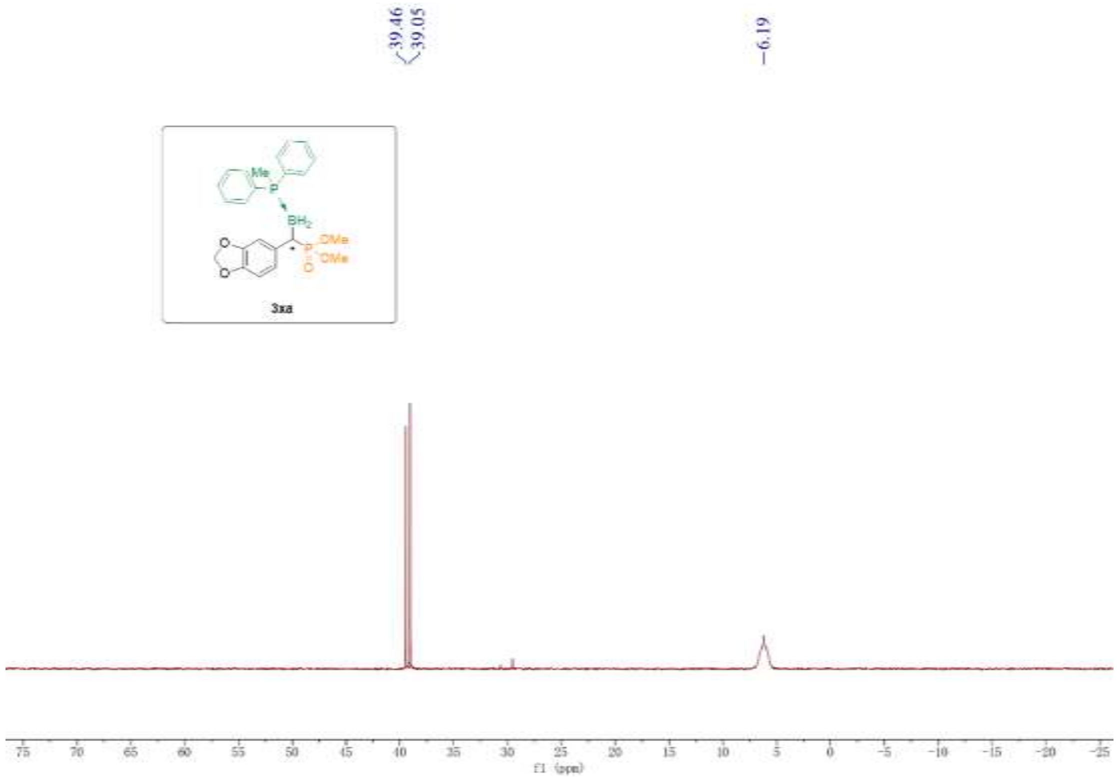

(S)-dimethyl(((methyldiphenylphosphane)boryl)(dibenzo[b,d]furan-1-yl)methyl) phosphonate(3ya)

^{1}H NMR (400 MHz, Chloroform-*d*)

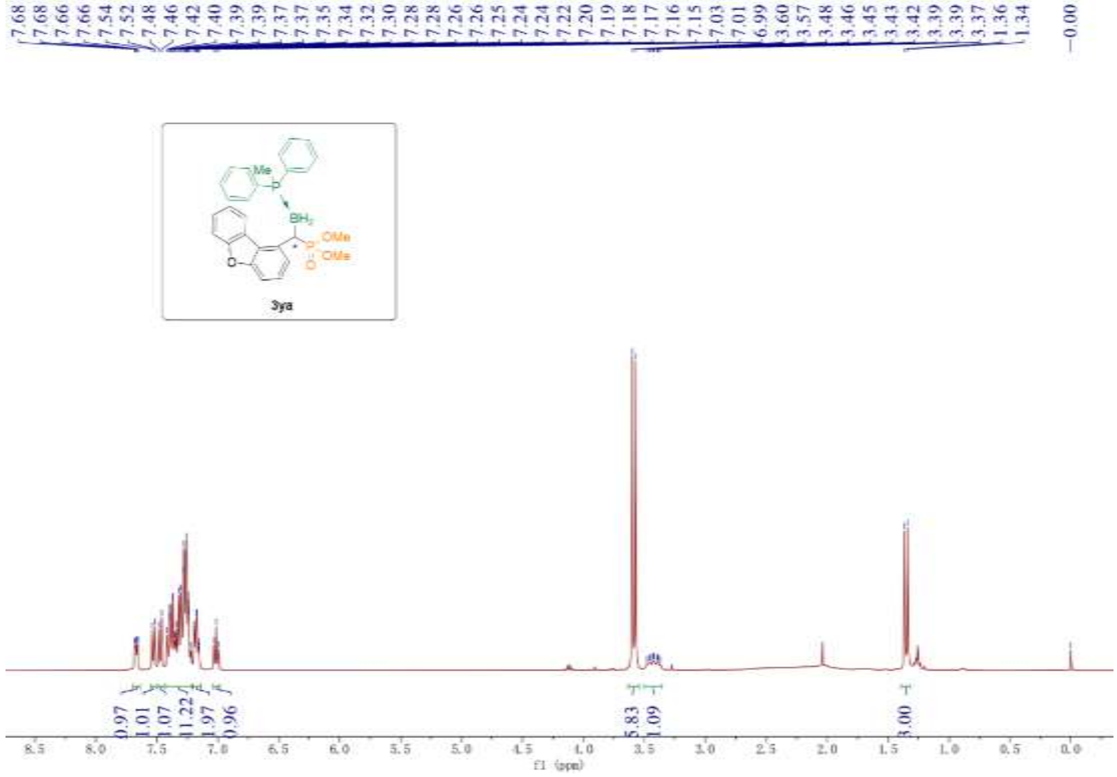

^{13}C NMR (101 MHz, Chloroform-*d*)

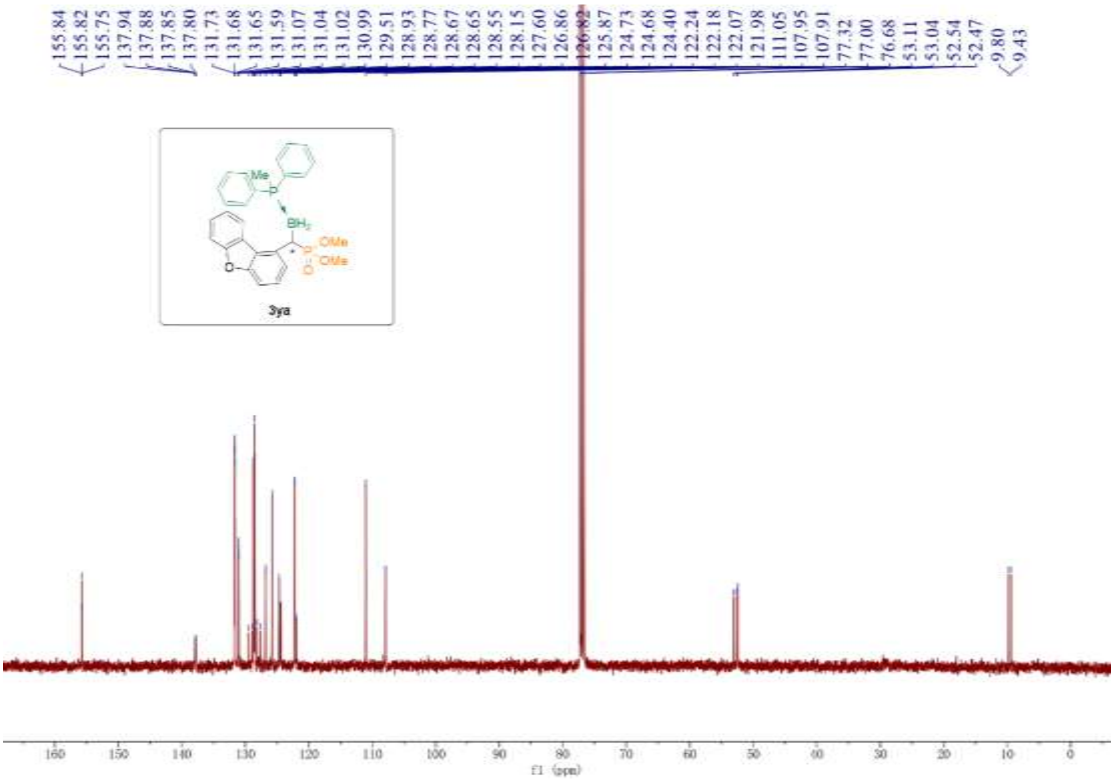

^{11}B NMR (128 MHz, Chloroform-*d*)

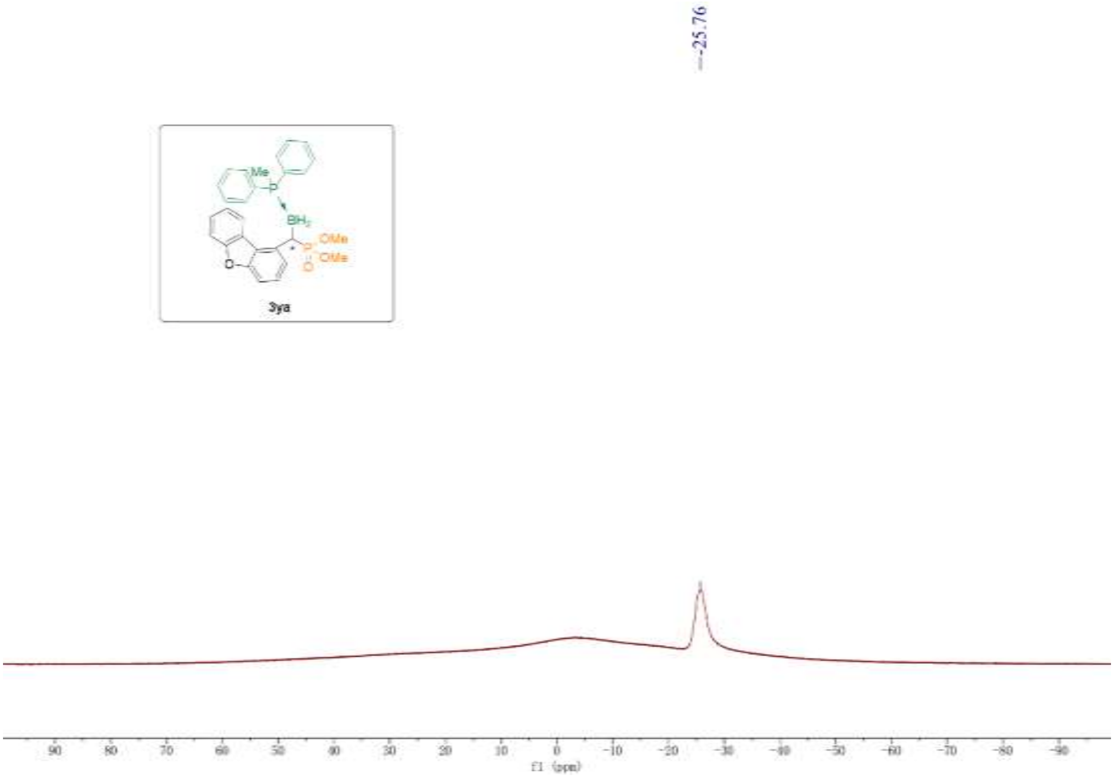

^{31}P NMR (162 MHz, Chloroform-*d*)

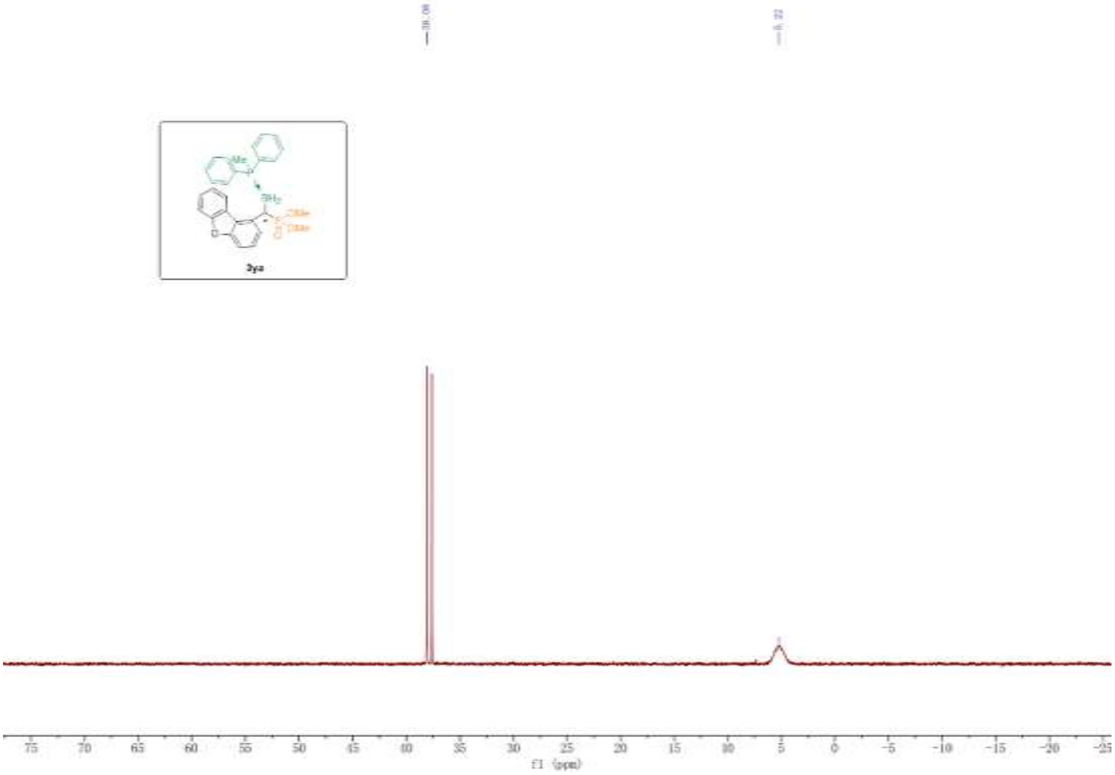

(S)-dimethyl(((methyldiphenylphosphane)boryl)(dibenzo[b,d]thiophen-4-yl)methyl)

phosphonate(3za)

^{1}H NMR (500 MHz, Chloroform-*d*)

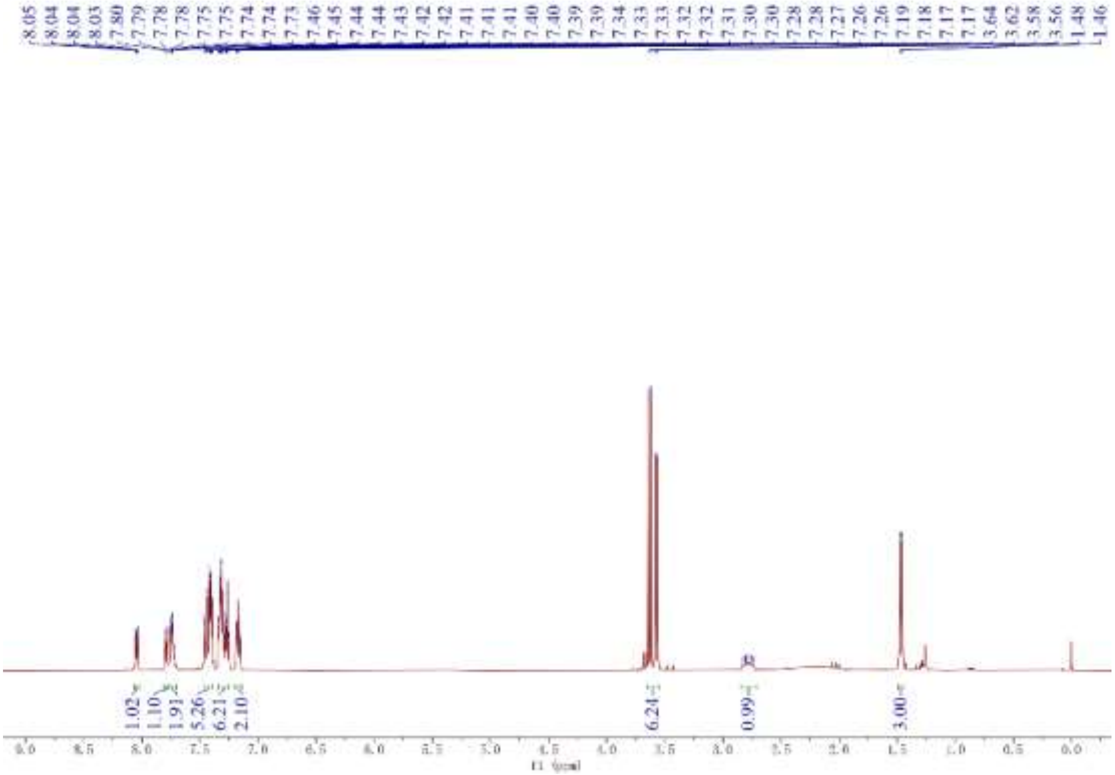

^{13}C NMR (126 MHz, Chloroform-*d*)

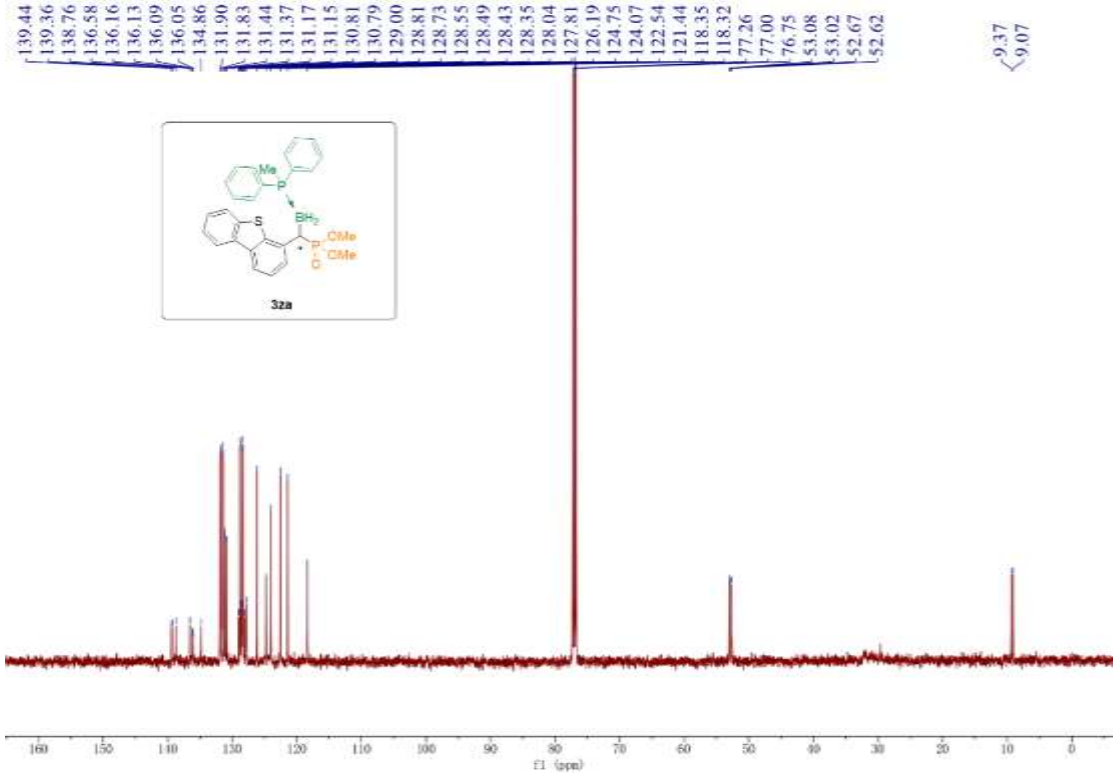

^{11}B NMR (160 MHz, Chloroform-*d*)

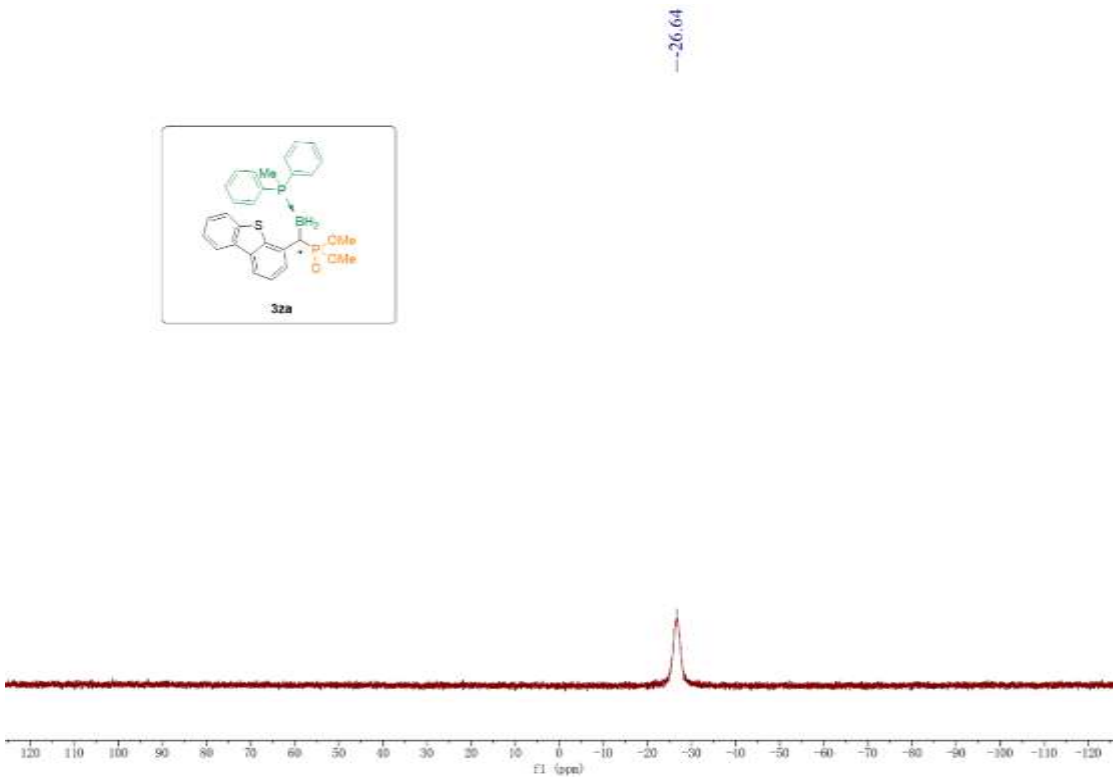

^{31}P NMR (202 MHz, Chloroform-*d*)

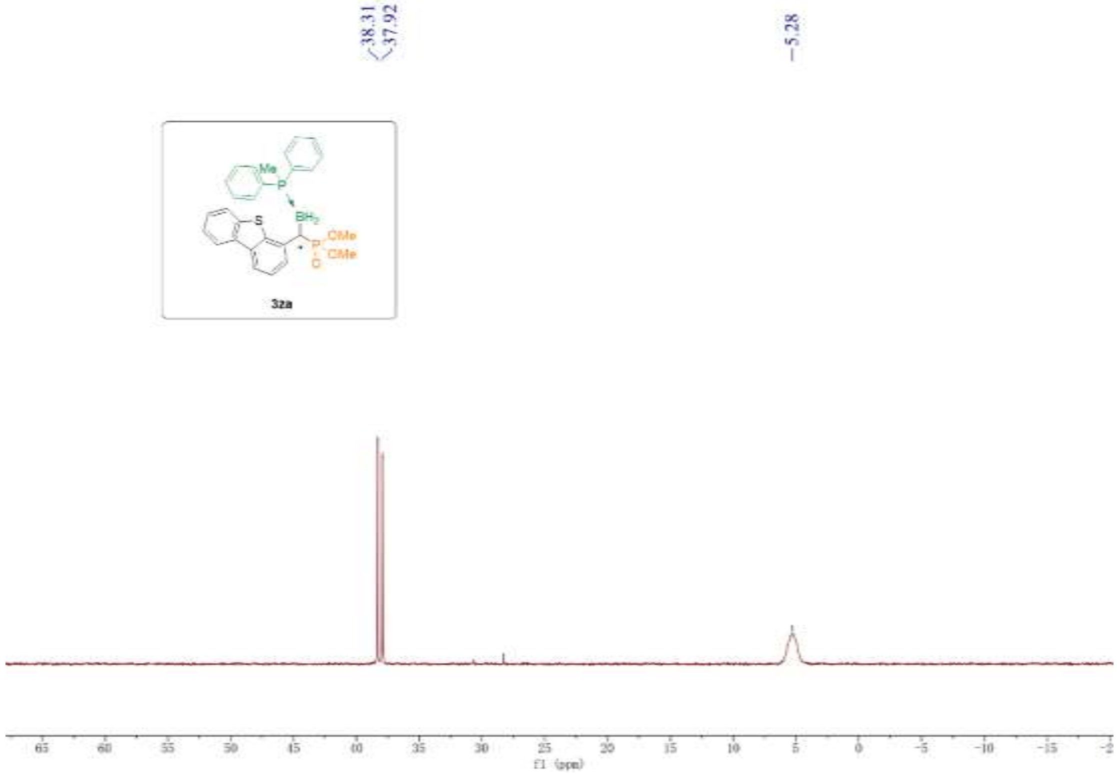

(S)-dimethyl(((methyldiphenylphosphane)boryl)(dibenzo[b,d]thiophen-4-yl)methyl) phosphonate(3Aa)

^{1}H NMR (400 MHz, Chloroform-*d*)

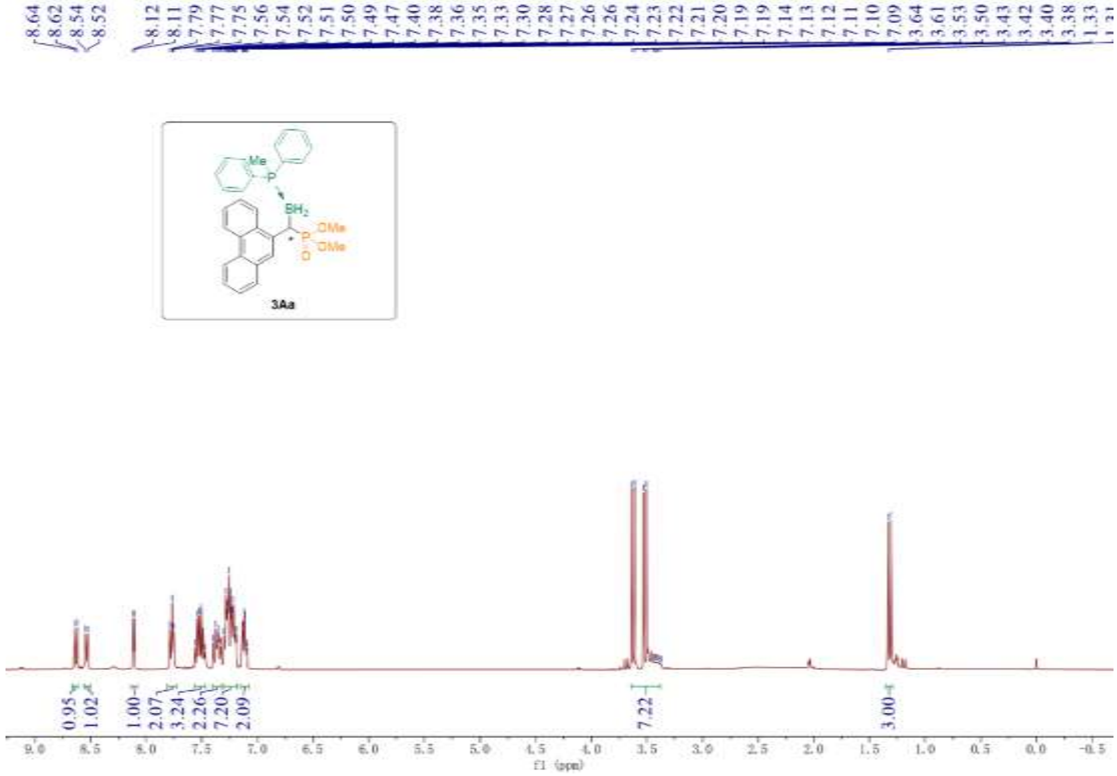

^{13}C NMR (101 MHz, Chloroform-*d*)

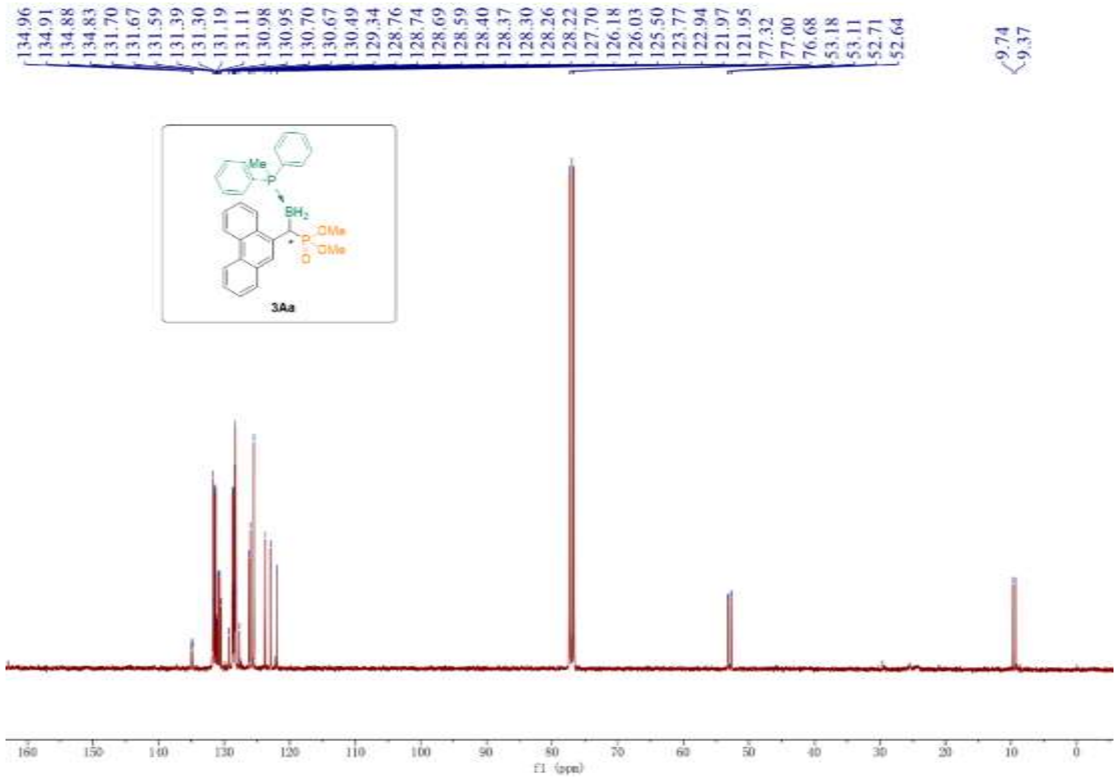

^{11}B NMR (128 MHz, Chloroform-*d*)

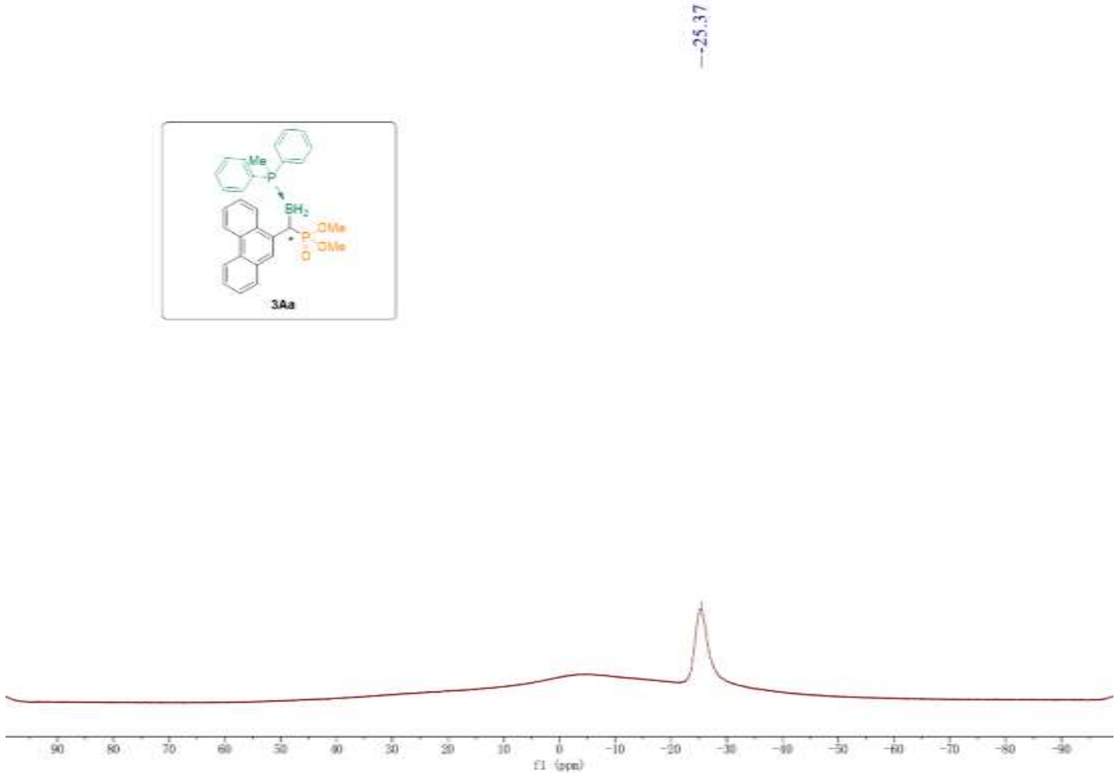

^{31}P NMR (162 MHz, Chloroform-*d*)

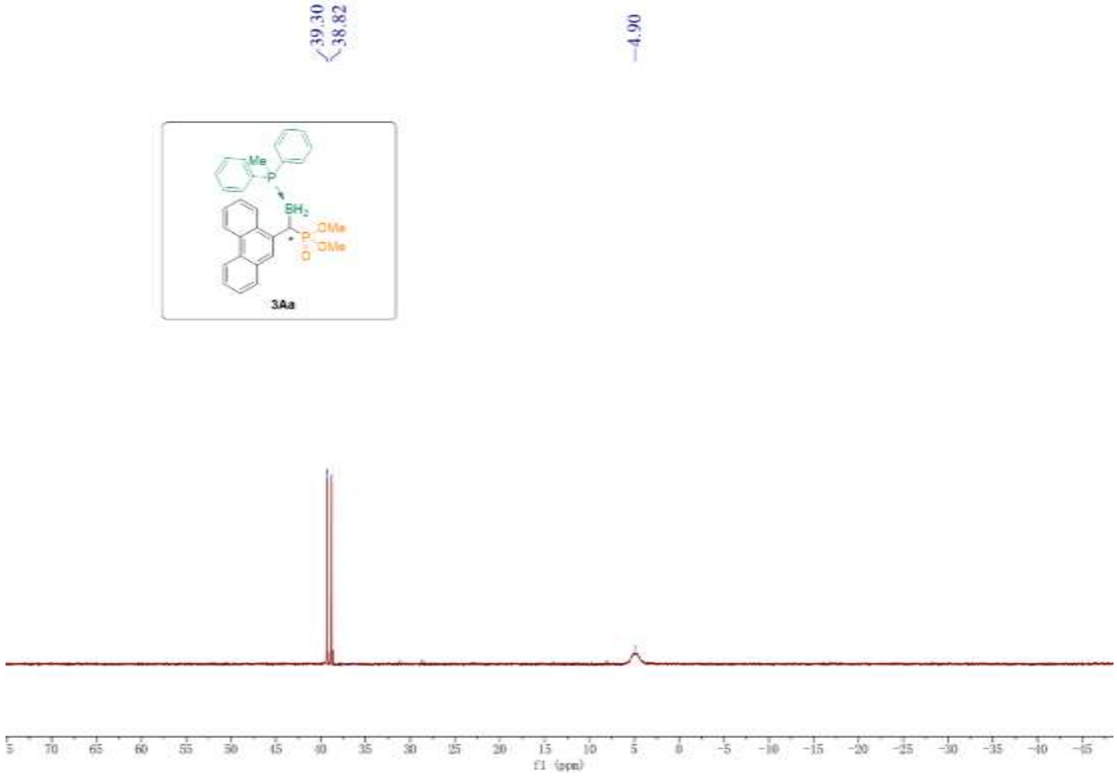

(S)-dimethyl(((methyldiphenylphosphane)boryl)(naphthalen-2-yl)methyl)phosphonate(3Ba)

^{1}H NMR (400 MHz, Chloroform-*d*)

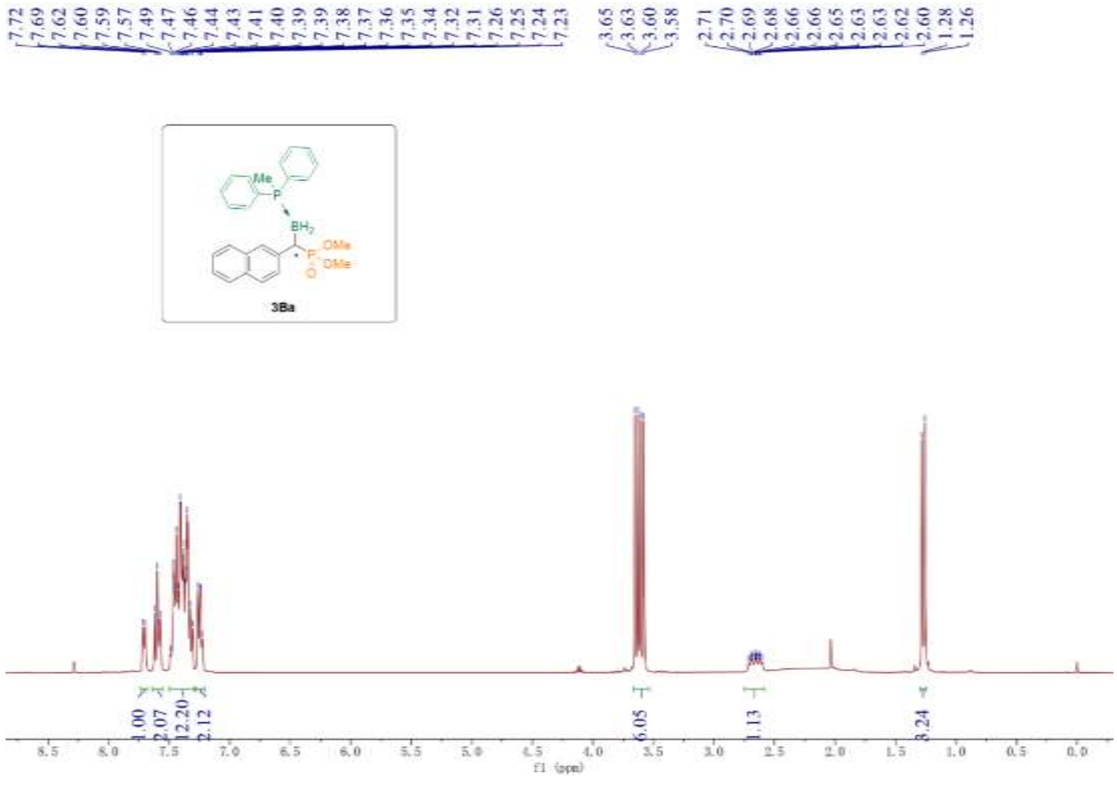

^{13}C NMR (101 MHz, Chloroform-*d*)

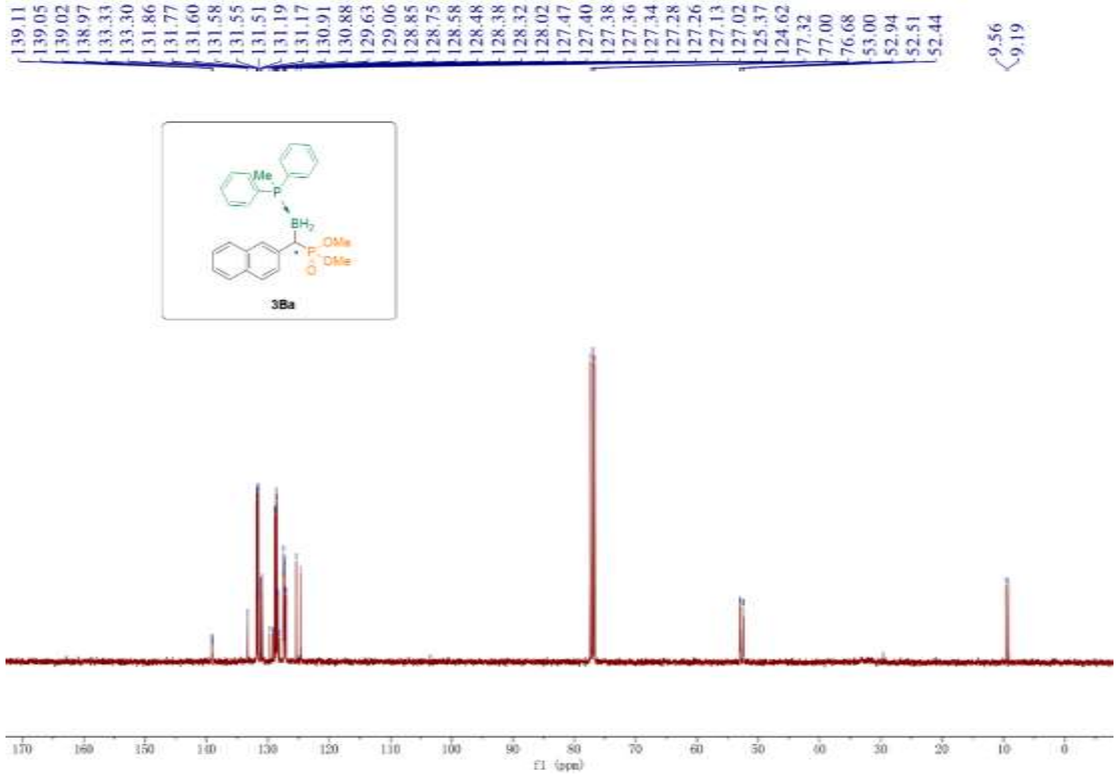

^{11}B NMR (128 MHz, Chloroform-*d*)

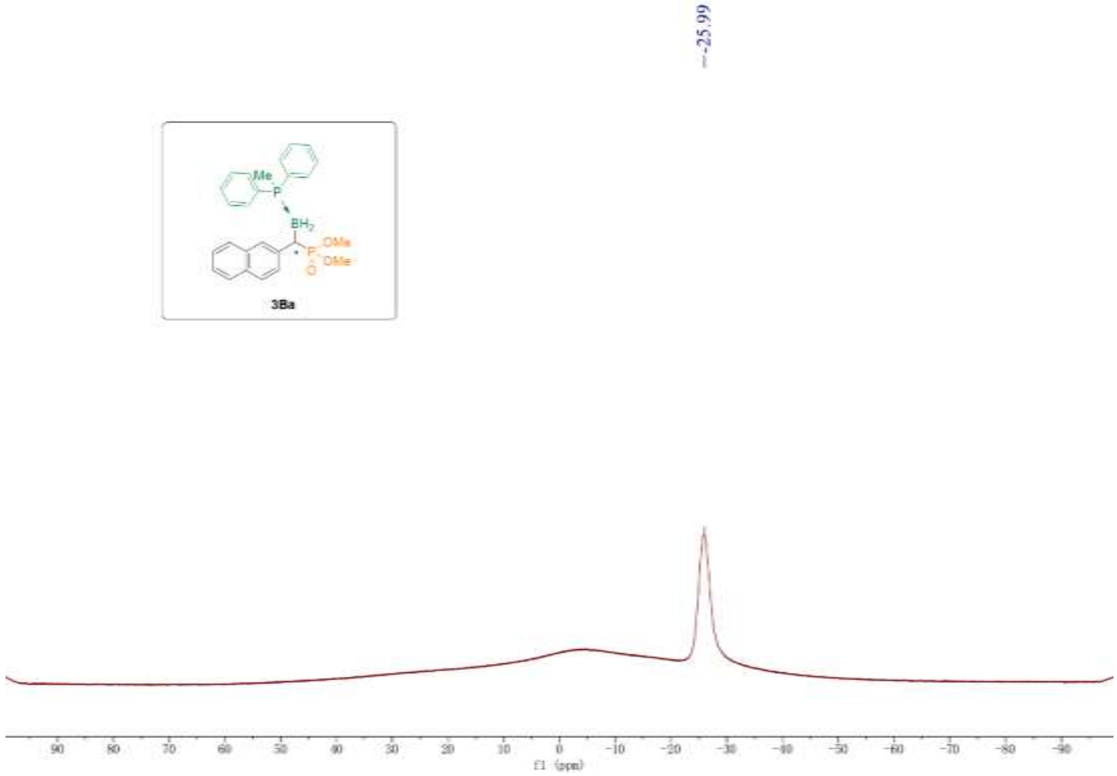

^{31}P NMR (162 MHz, Chloroform-*d*)

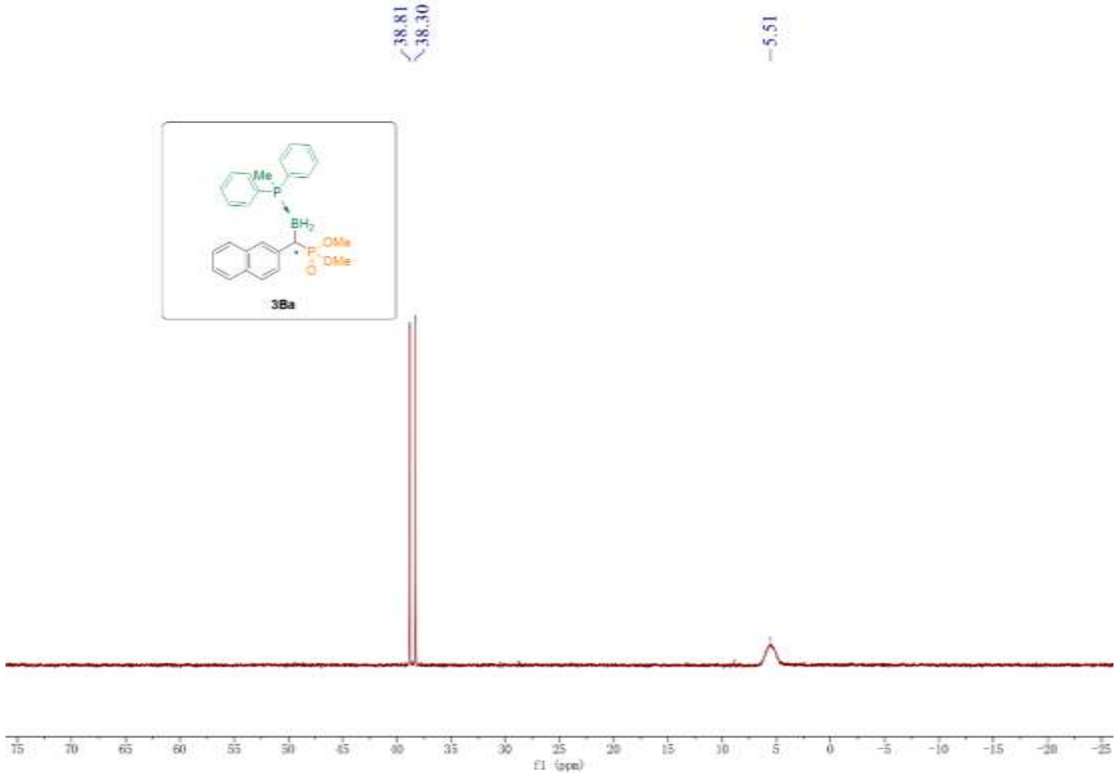

(S)-Dimethyl((methyldiphenylphosphane-boryl)(naphthalen-2-yl)phosphonate(3Ca)

^{1}H NMR (400 MHz, Chloroform-*d*)

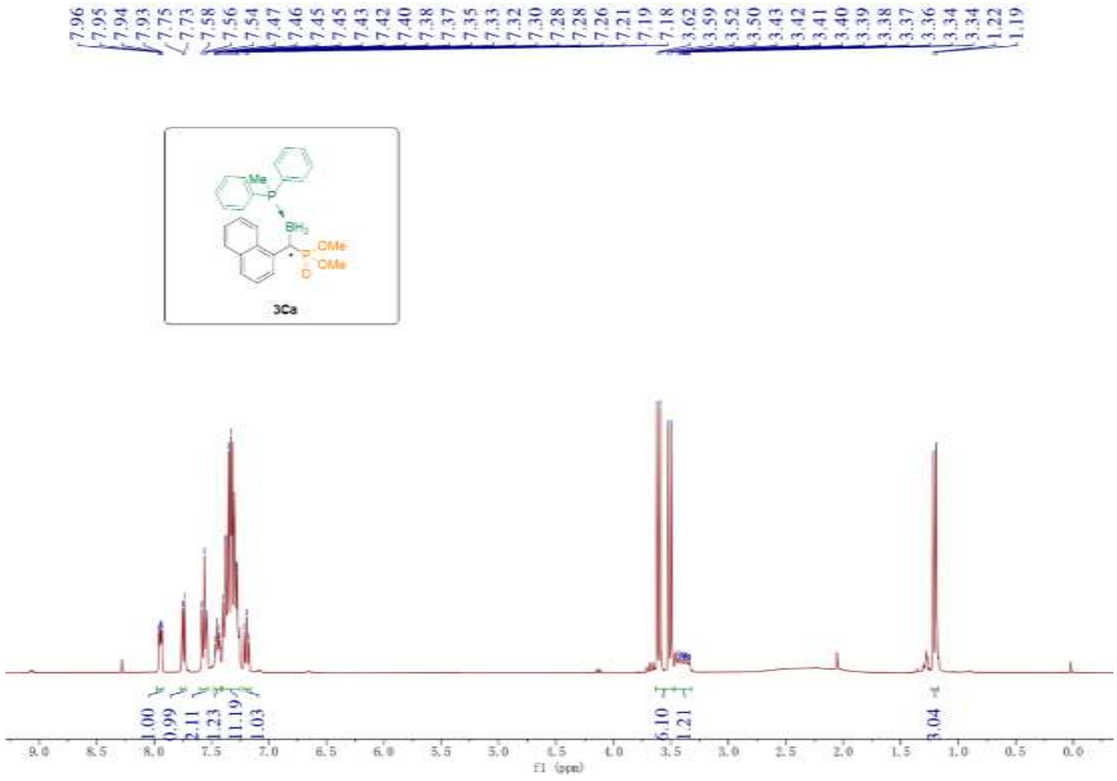

^{13}C NMR (101 MHz, Chloroform-*d*)

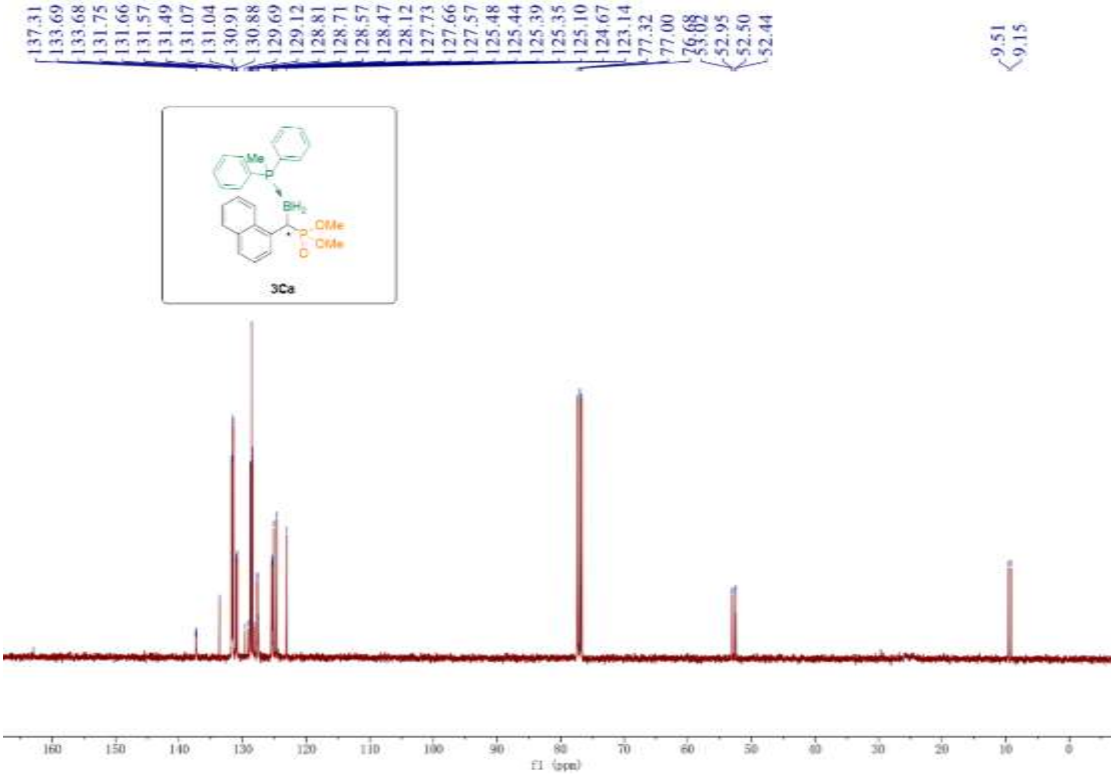

^{11}B NMR (128 MHz, Chloroform-*d*)

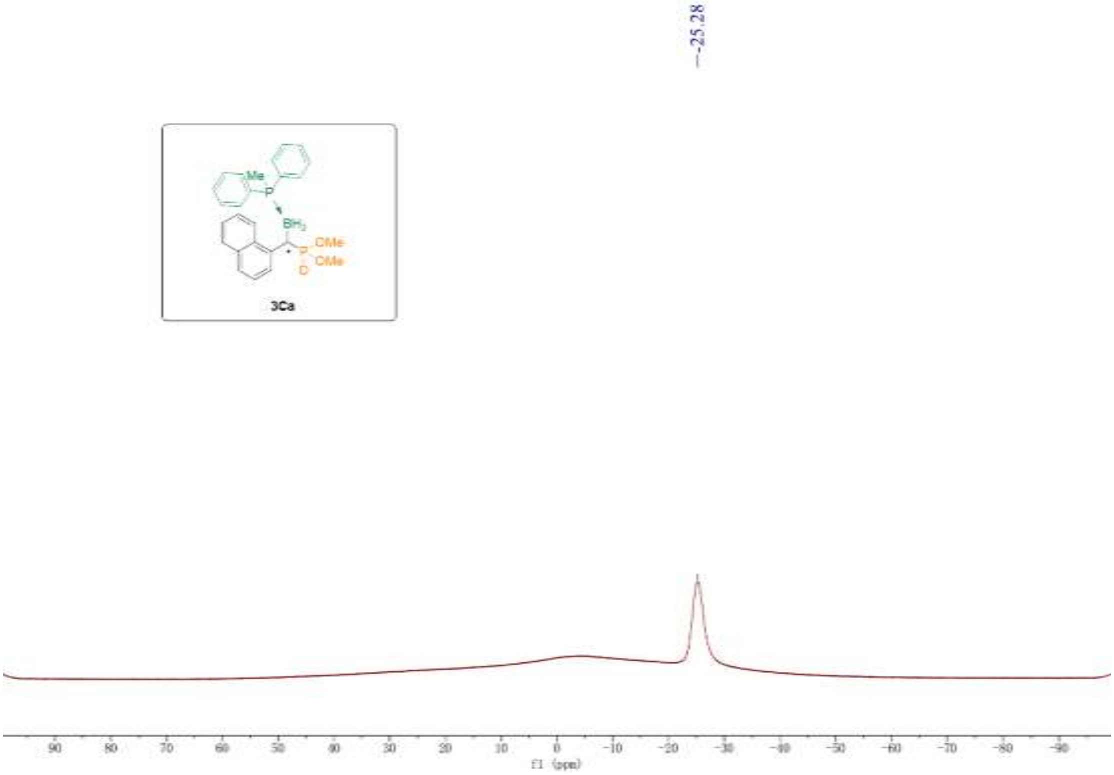

^{31}P NMR (162 MHz, Chloroform-*d*)

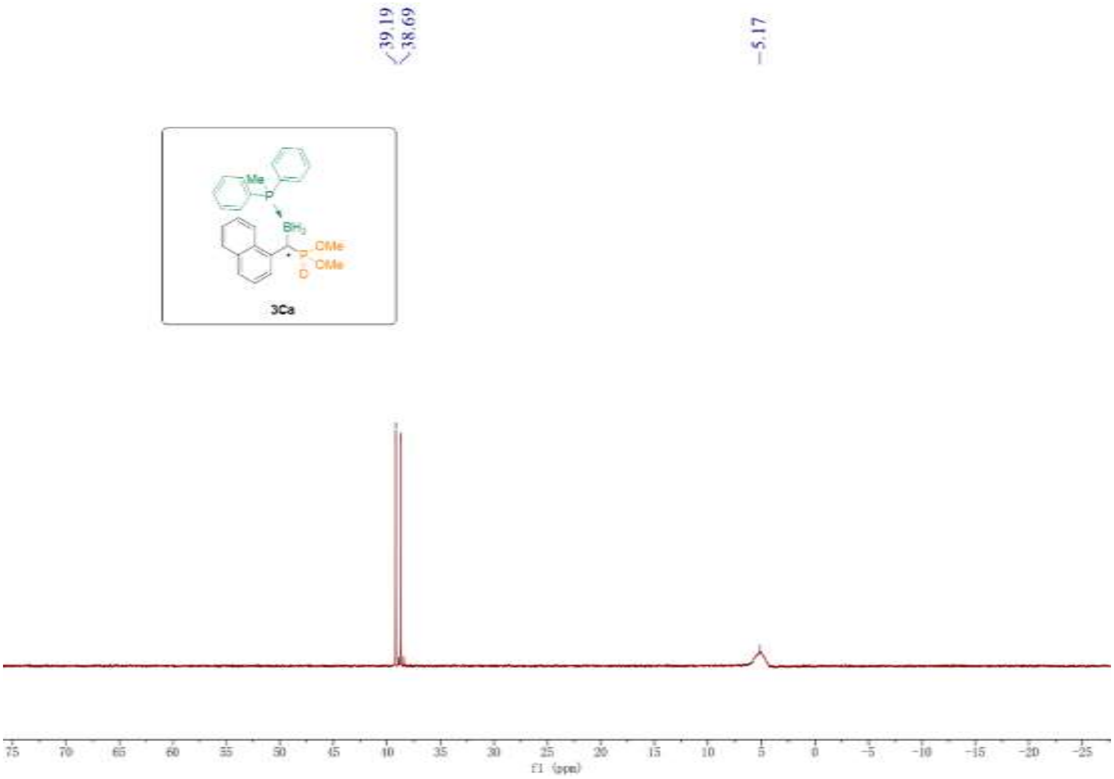

(S)-dimethyl(((ethyldiphenylphosphane)boryl)(phenyl)methyl)phosphonate(3ab)

^{1}H NMR (400 MHz, Chloroform-*d*)

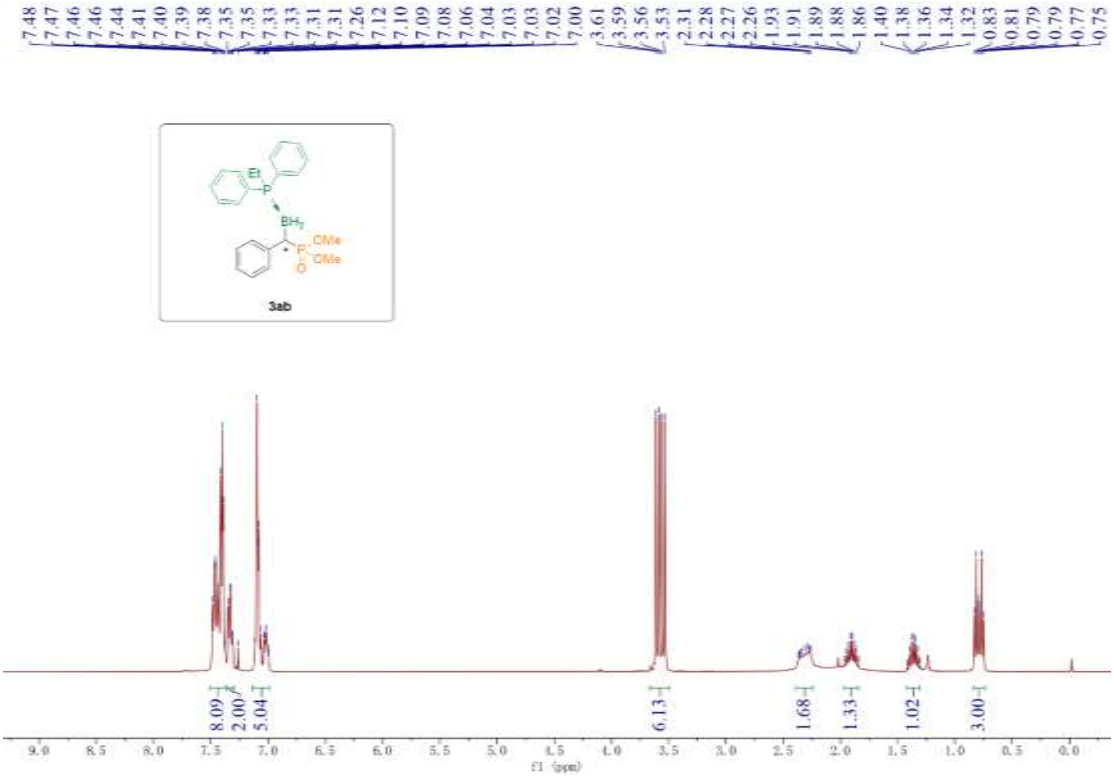

^{13}C NMR (101 MHz, Chloroform-d)

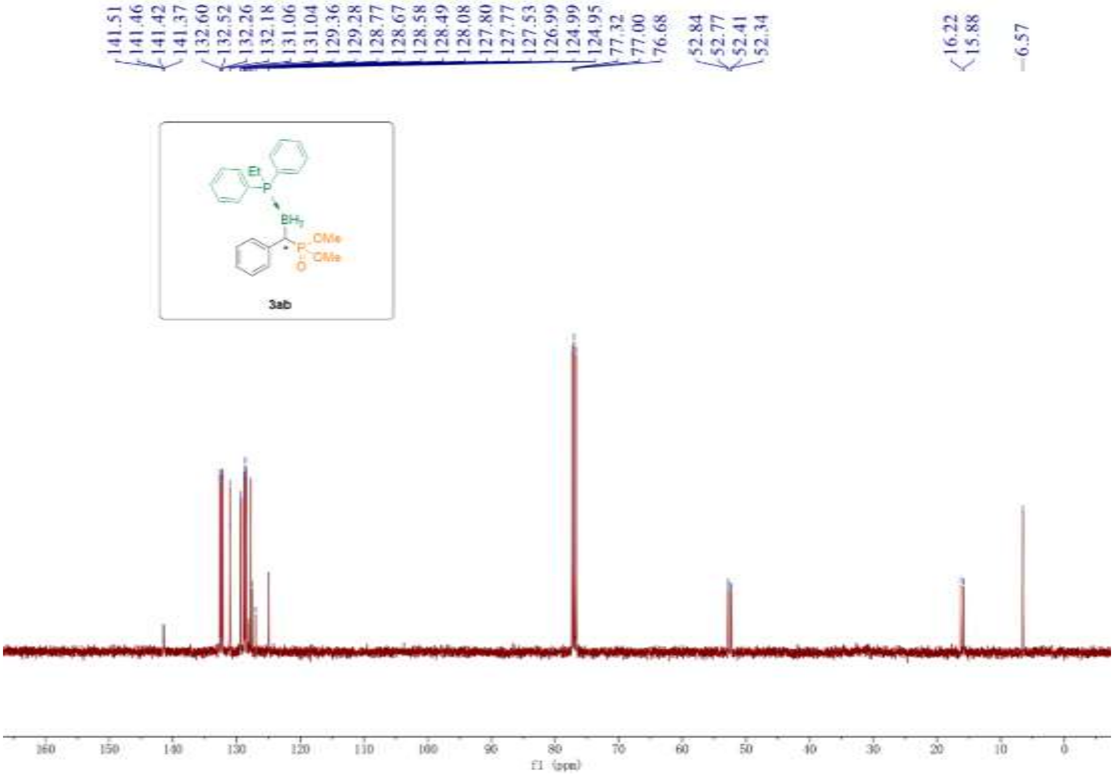

^{11}B NMR (128 MHz, Chloroform-d)

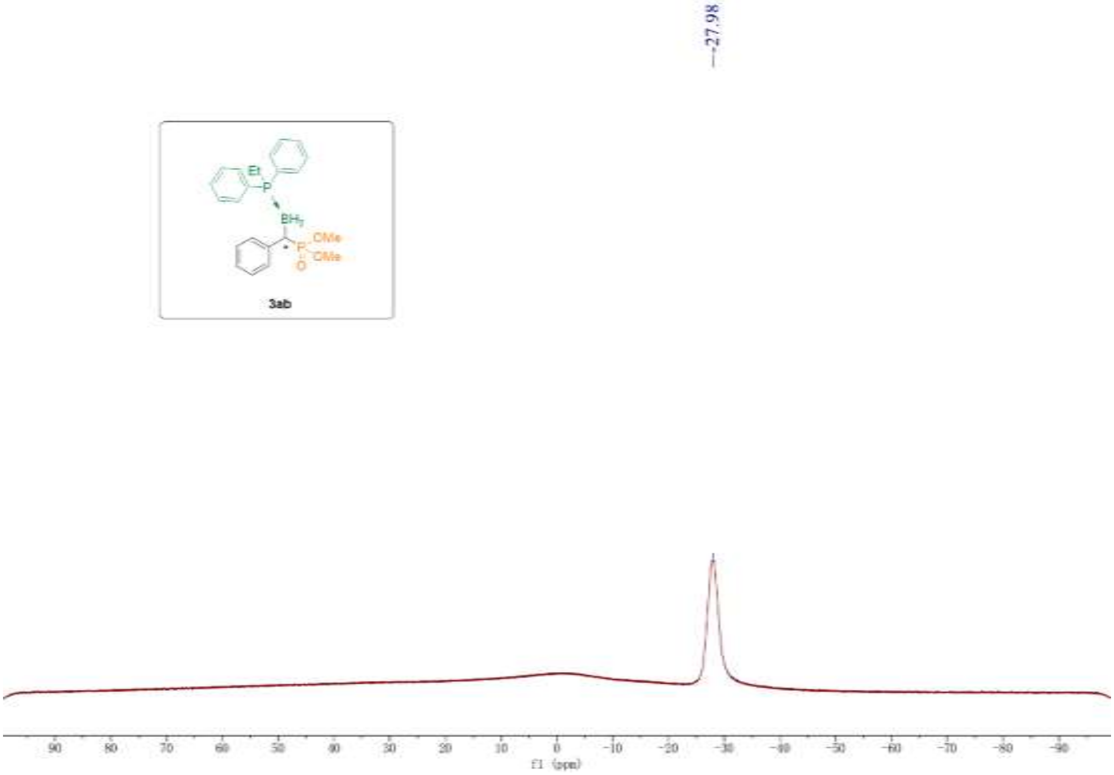

^{31}P NMR (162 MHz, Chloroform-*d*)

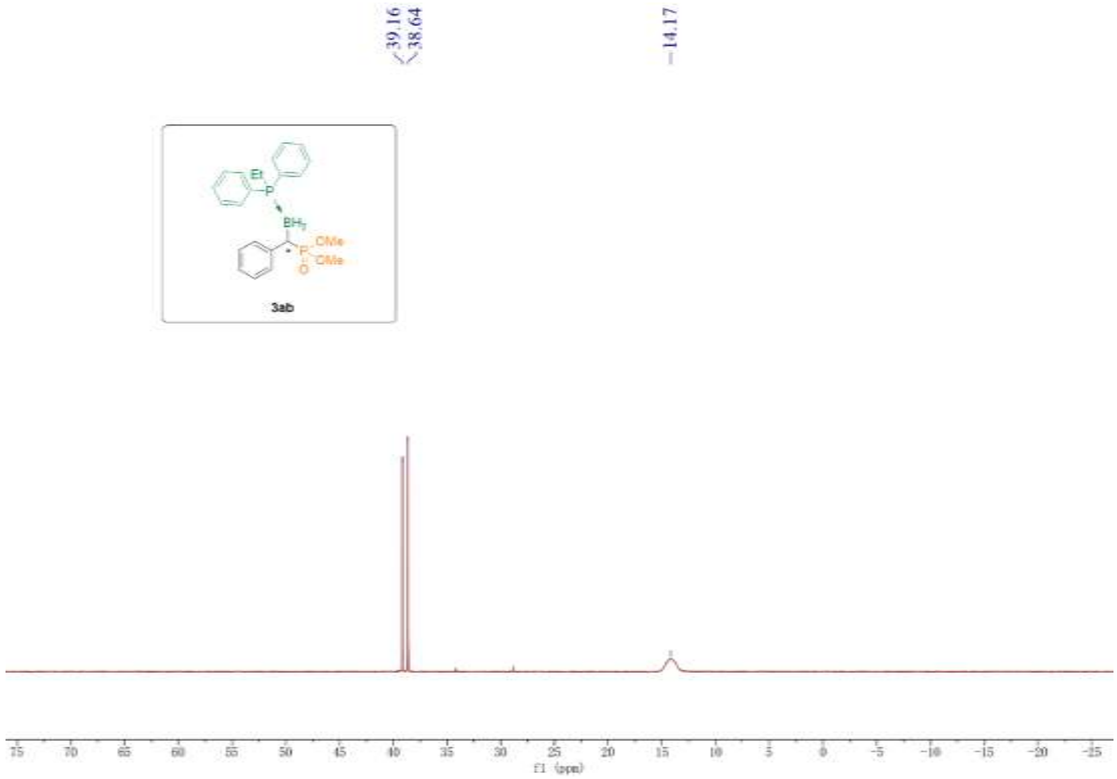

(S)-dimethyl(((isopropyldiphenylphosphane)boryl)(phenyl)methyl)phosphonate(3ac)

^{1}H NMR (400 MHz, Chloroform-*d*)

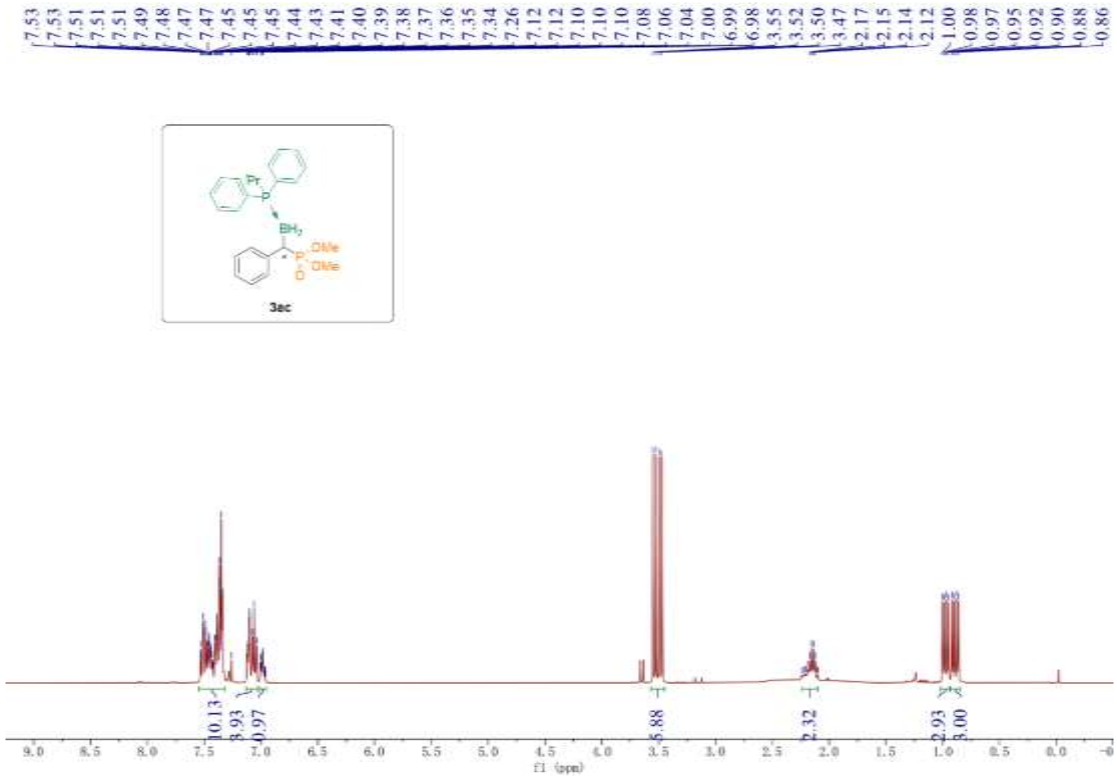

^{13}C NMR (101 MHz, Chloroform-*d*)

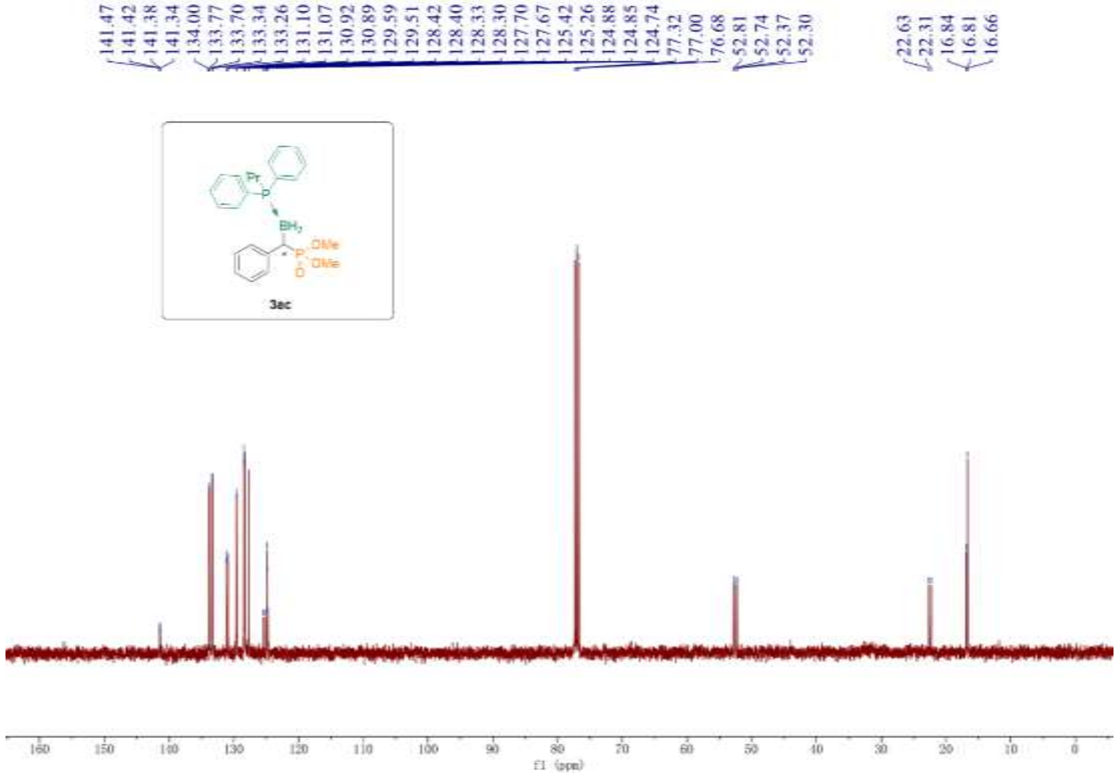

^{11}B NMR (128 MHz, Chloroform-*d*)

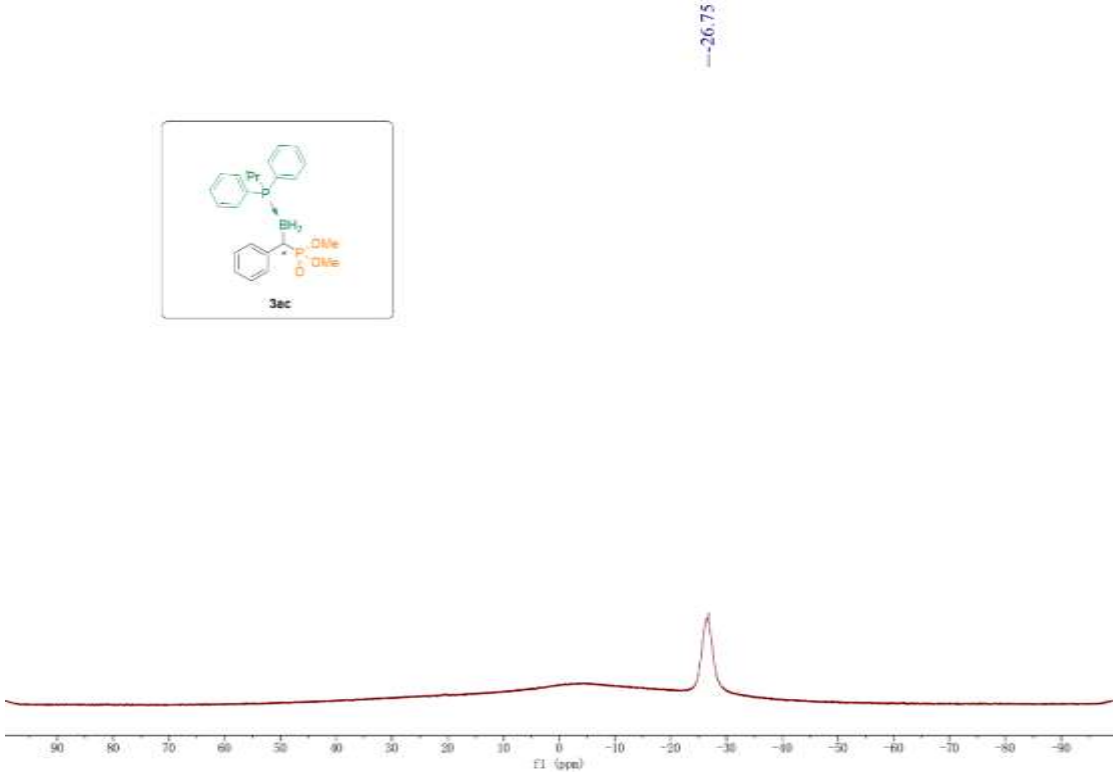

^{31}P NMR (162 MHz, Chloroform-*d*)

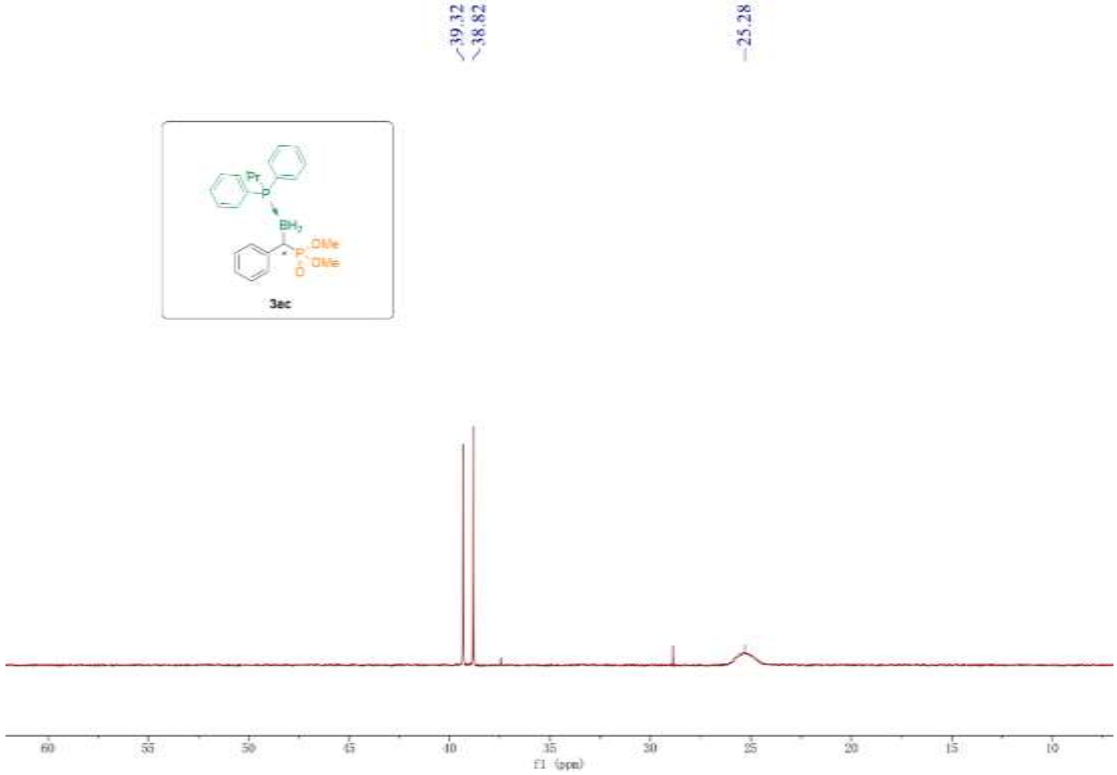

(S)-dimethyl(((dimethyl(phenyl)phosphane)boryl)(phenyl)methyl)phosphonate(3ad)

^{1}H NMR (500 MHz, Chloroform-*d*)

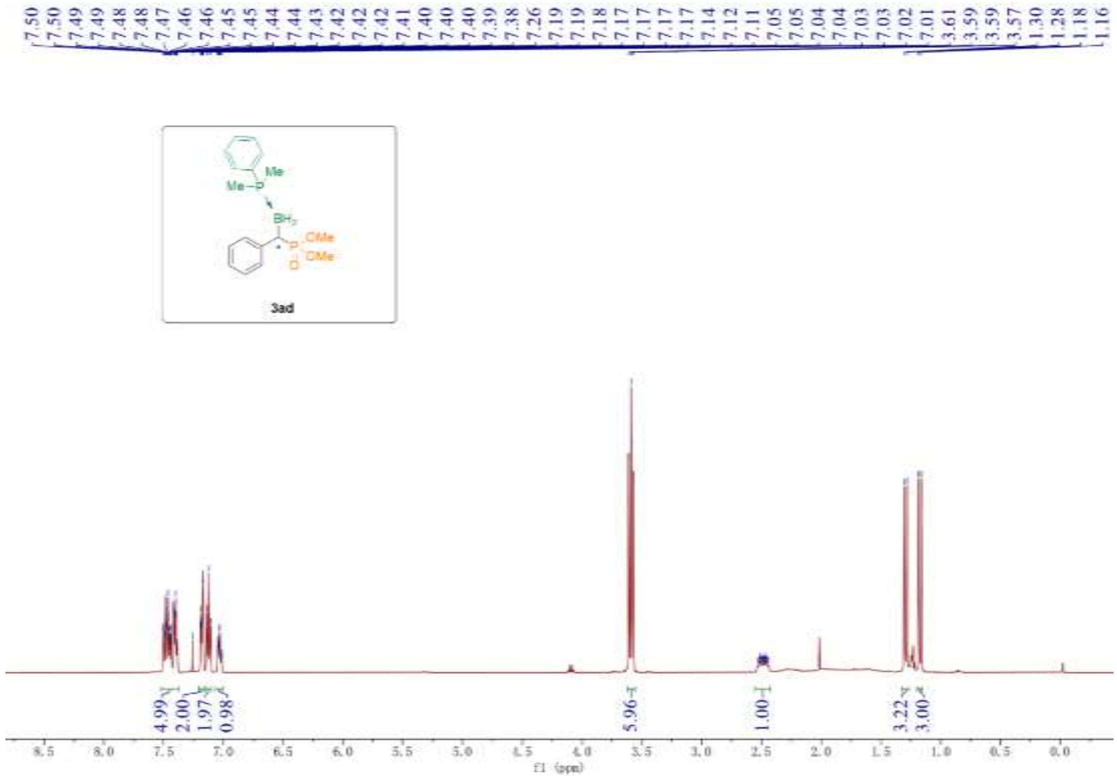

^{13}C NMR (126 MHz, Chloroform-*d*)

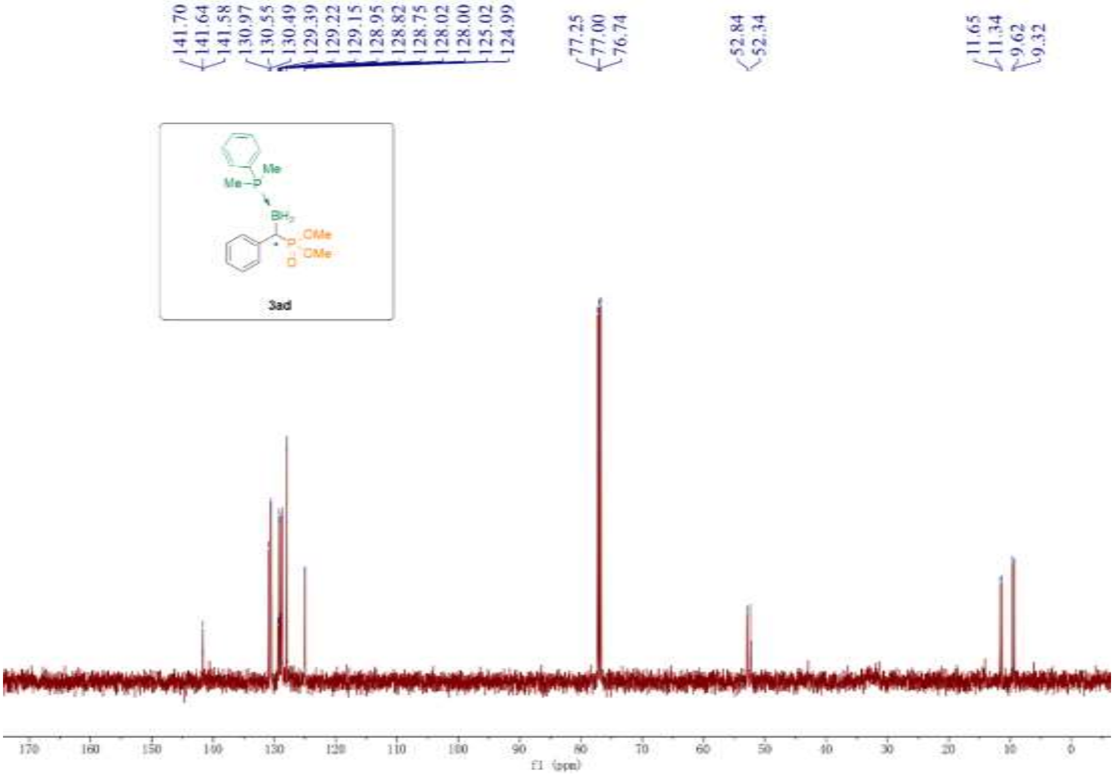

^{11}B NMR (202 MHz, Chloroform-*d*)

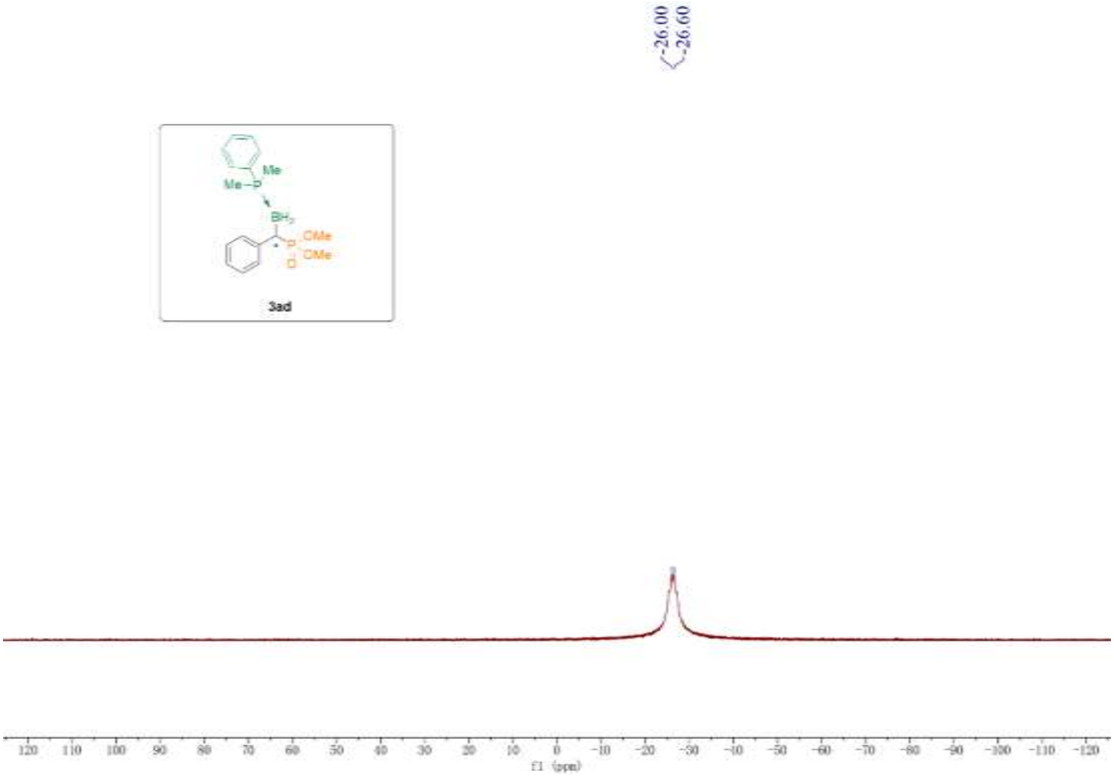

^{31}P NMR (160 MHz, Chloroform-*d*)

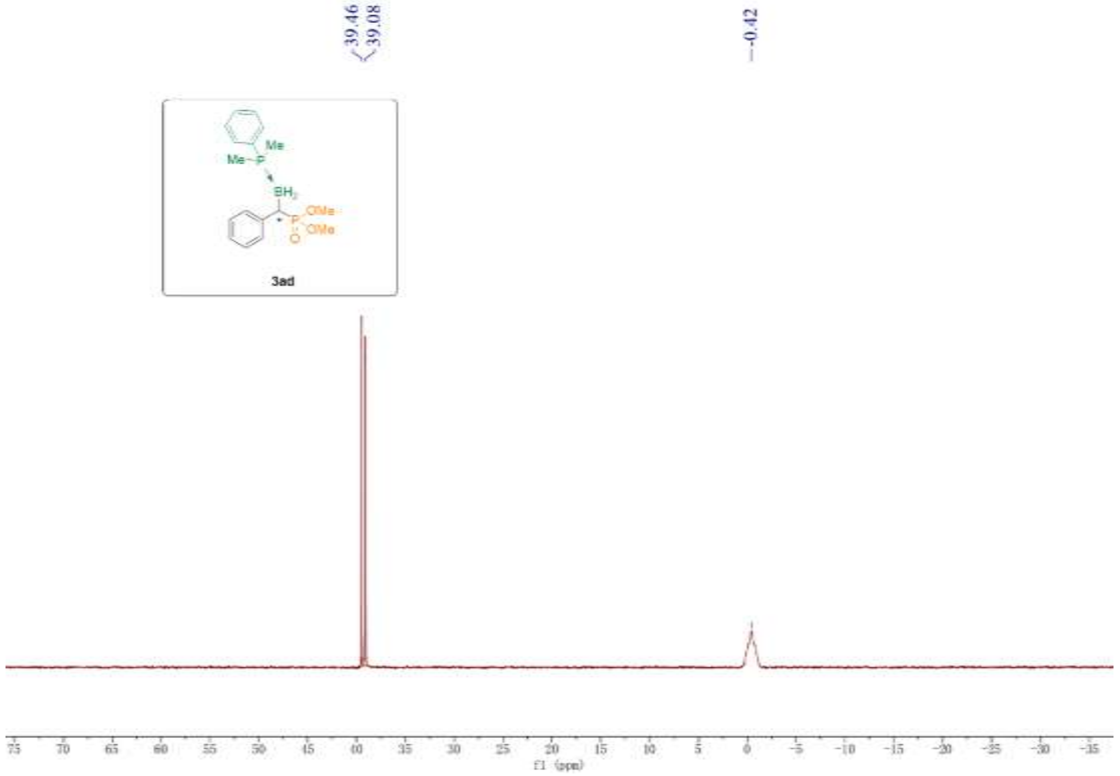

(S)-dimethyl(((diethyl(phenyl)phosphane)boryl)(phenyl)methyl)phosphonate(3ae)

^{1}H NMR (500 MHz, Chloroform-*d*)

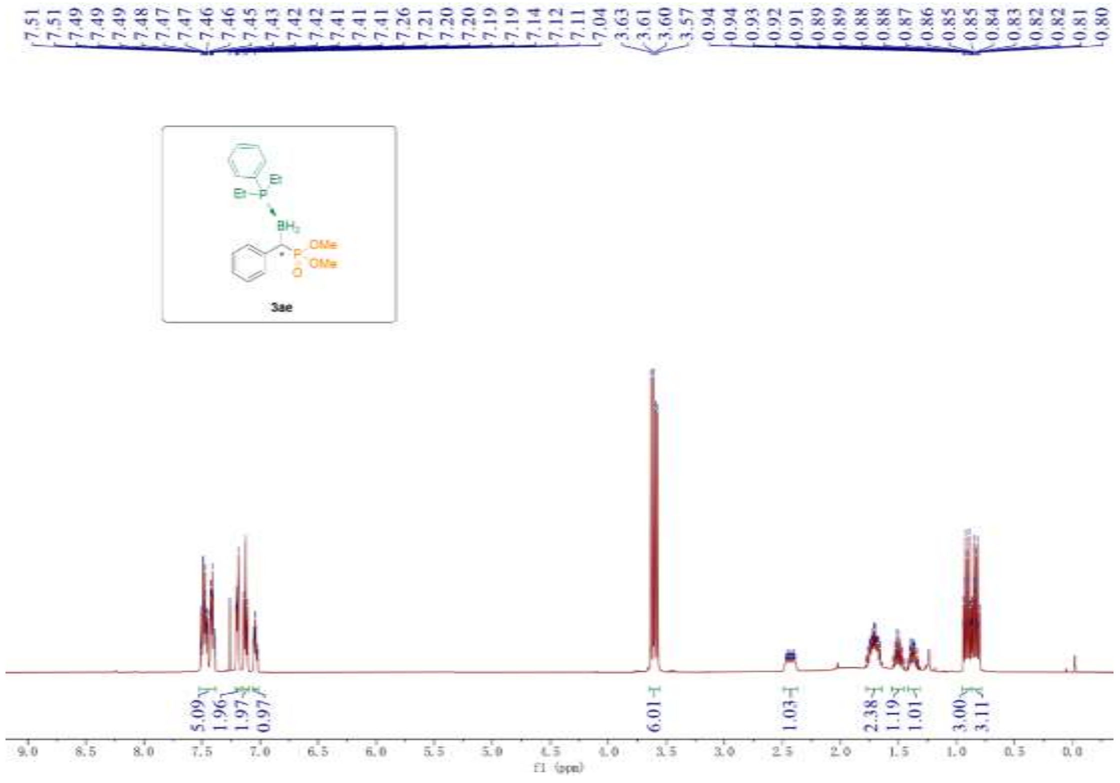

^{13}C NMR (126 MHz, Chloroform-*d*)

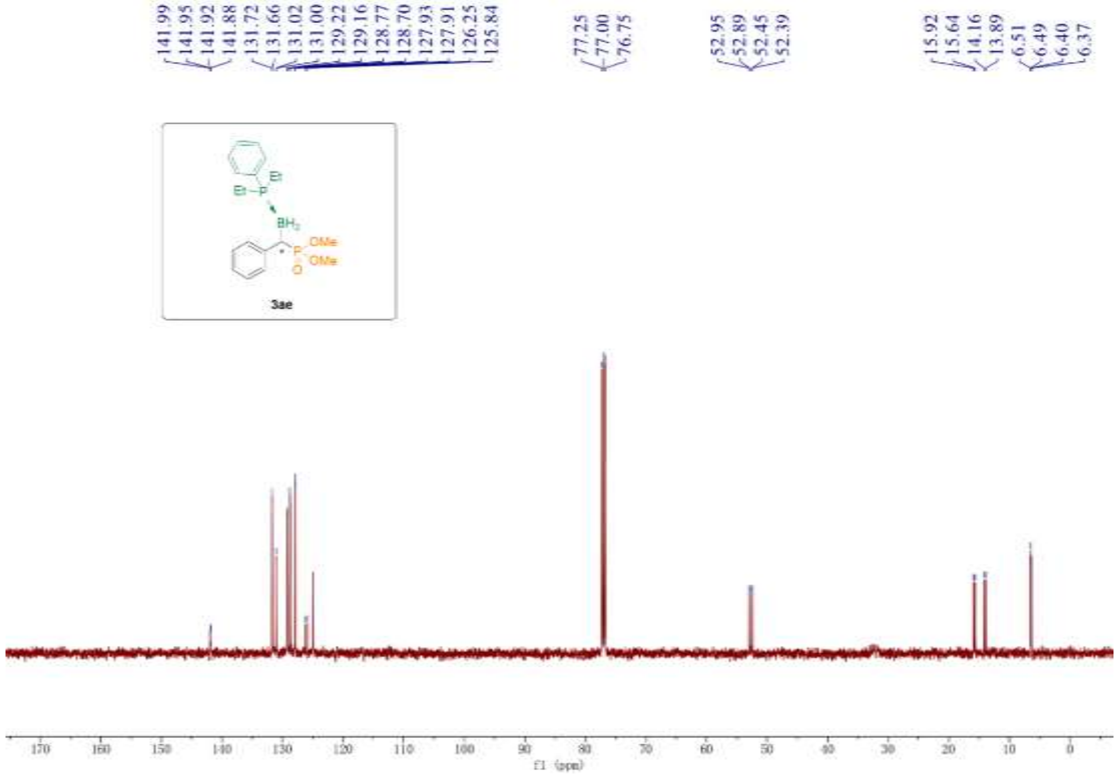

^{11}B NMR (128 MHz, Chloroform-*d*)

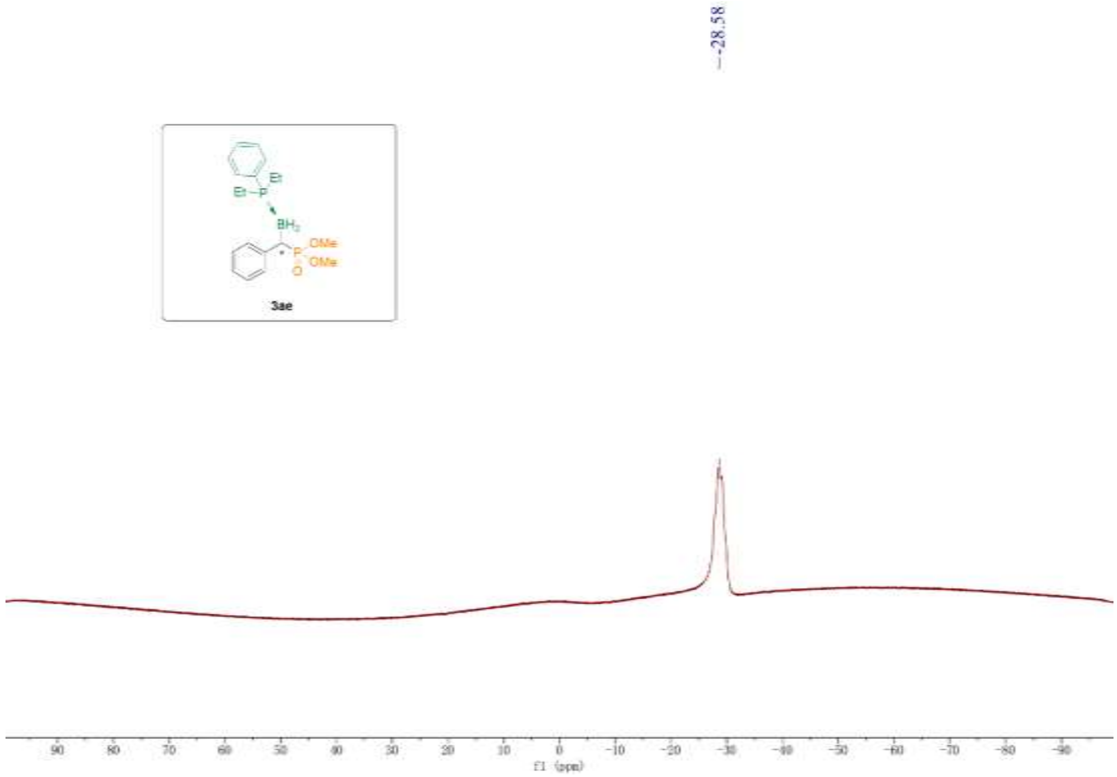

^{31}P NMR (202 MHz, Chloroform-*d*)

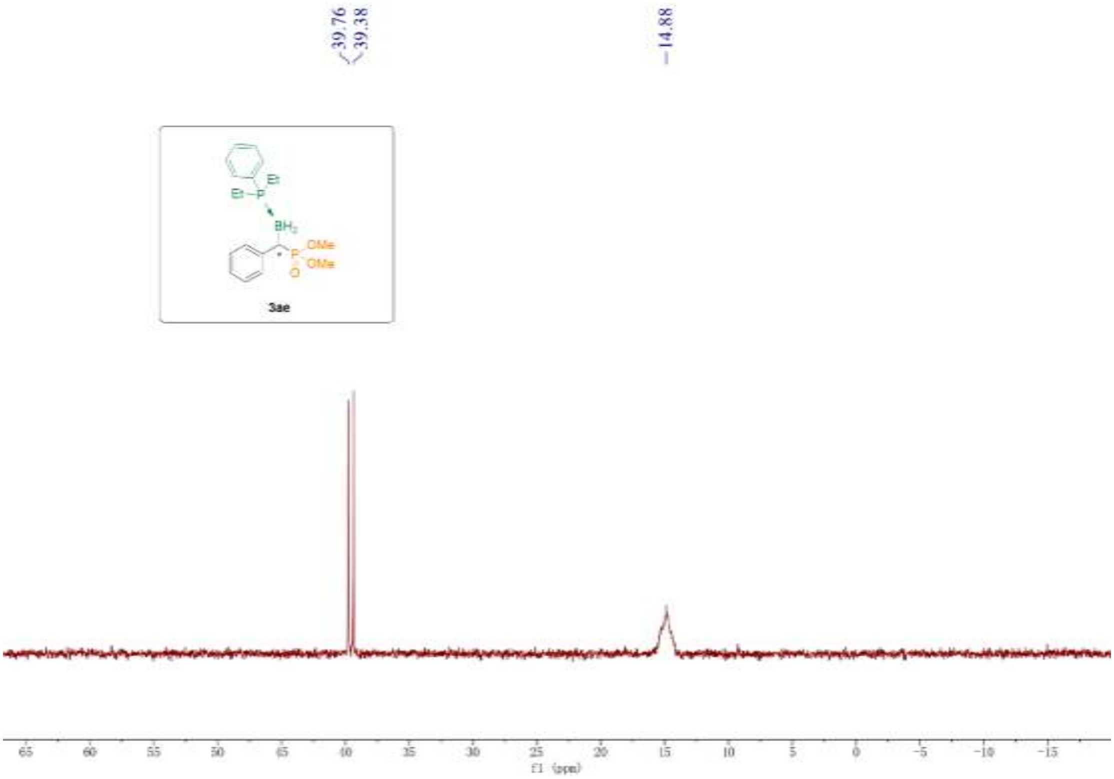

(S)-dimethyl(((dicyclohexyl(phenyl)phosphane)boryl)(phenyl)methyl)phosphonate(3af)

^{1}H NMR (400 MHz, Chloroform-*d*)

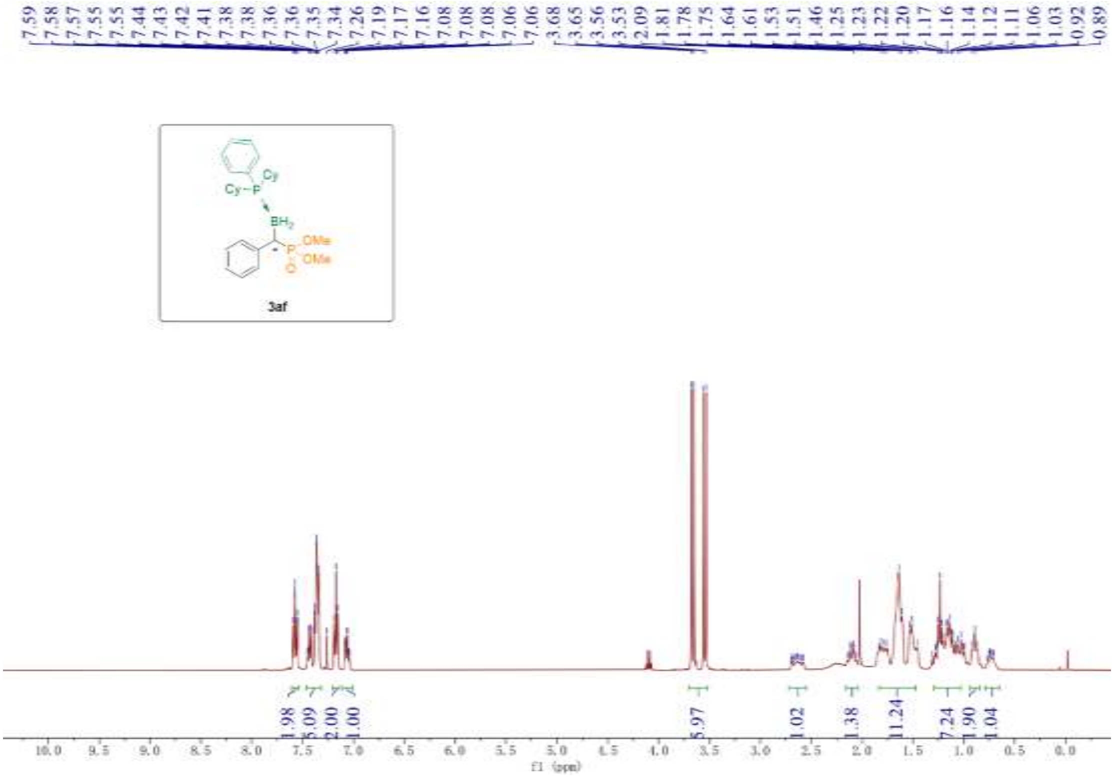

^{13}C NMR (101 MHz, Chloroform-*d*)

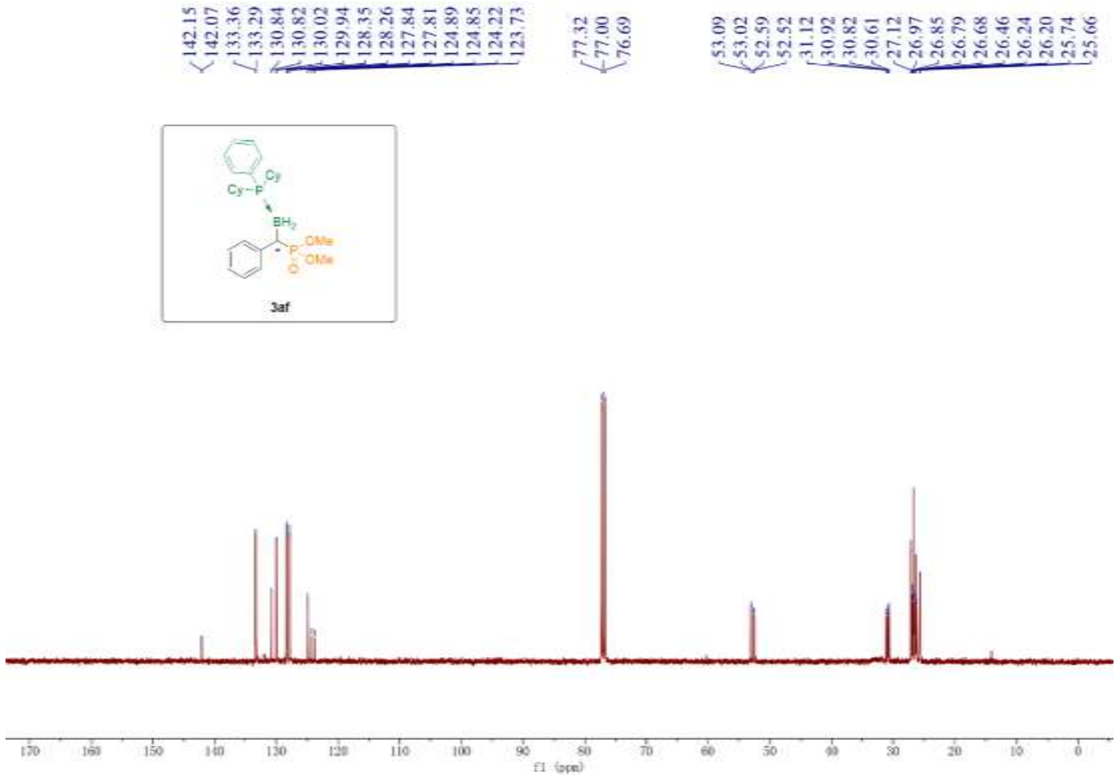

^{11}B NMR (128 MHz, Chloroform-*d*)

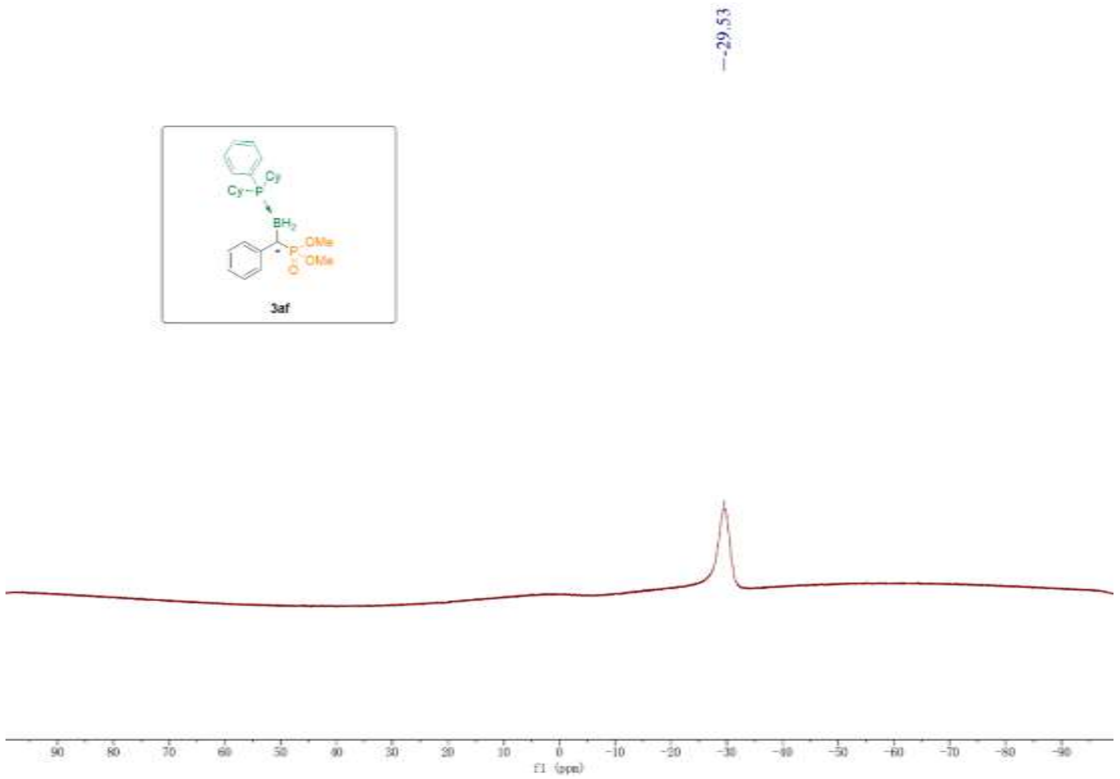

^{31}P NMR (162 MHz, Chloroform-*d*)

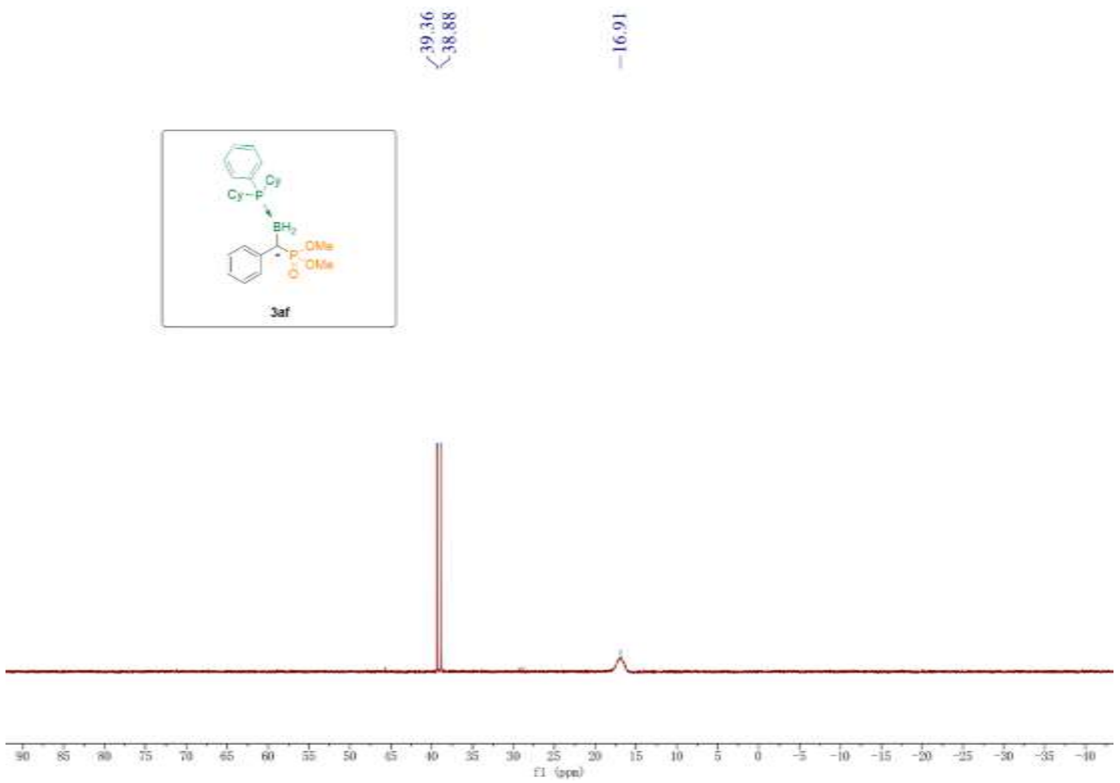

(S)-dimethyl(((trimethylphosphane)boryl)([1,1'-biphenyl]-4-yl)methyl)phosphonate(3lg)

^{1}H NMR (400 MHz, Chloroform-*d*)

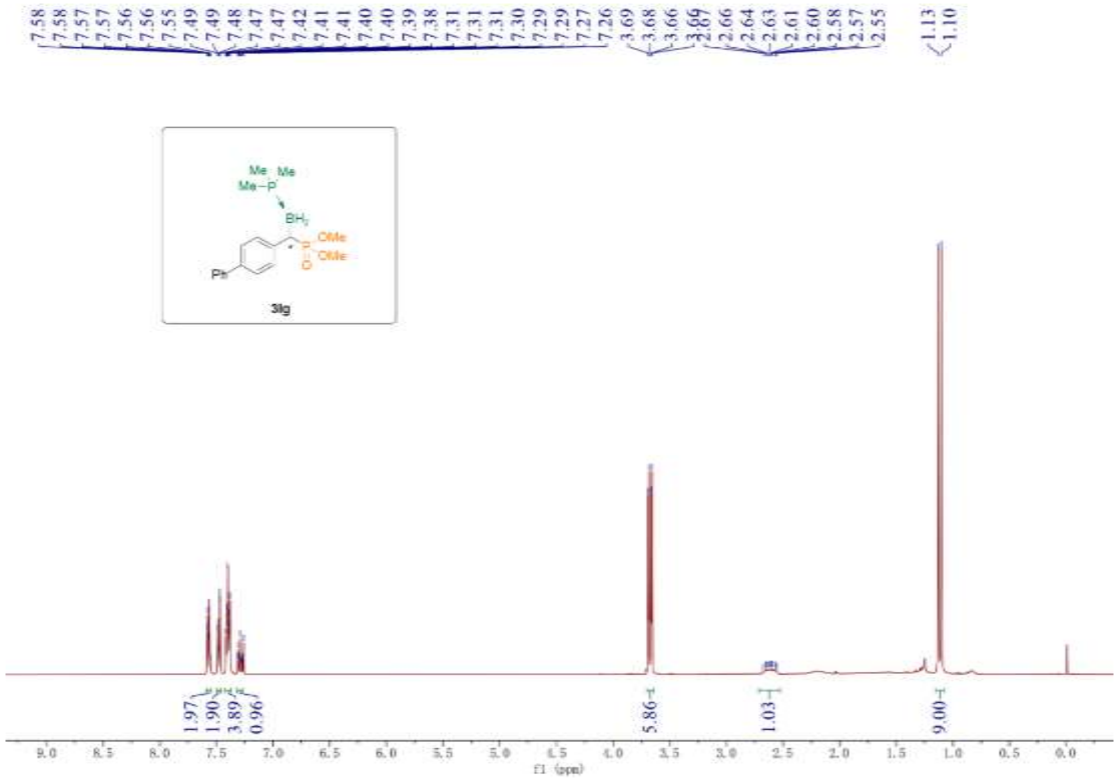

^{13}C NMR (101 MHz, Chloroform-*d*)

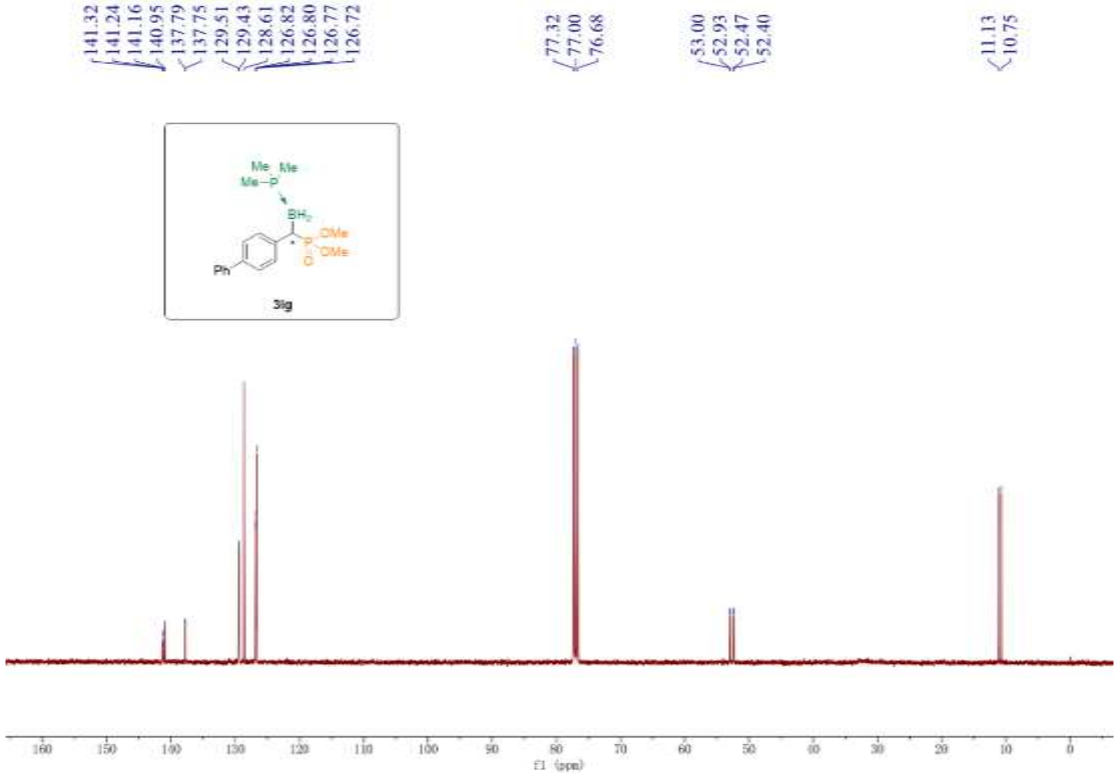

^{11}B NMR (128 MHz, Chloroform-*d*)

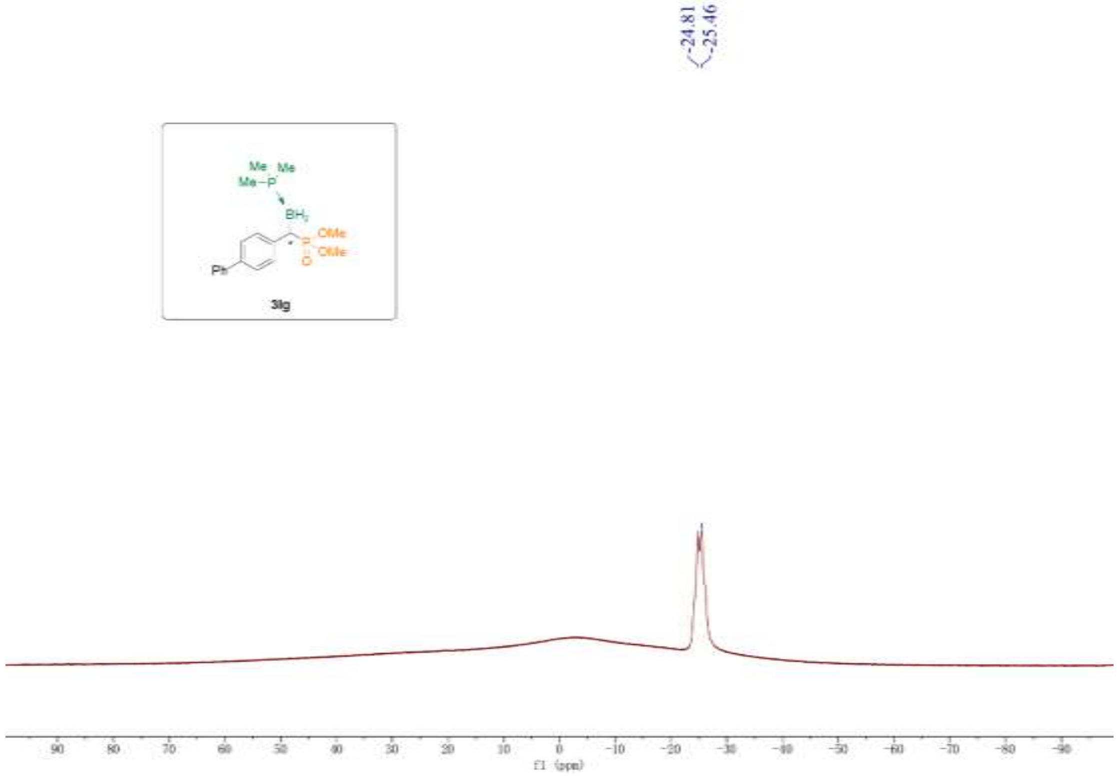

^{31}P NMR (162 MHz, Chloroform-*d*)

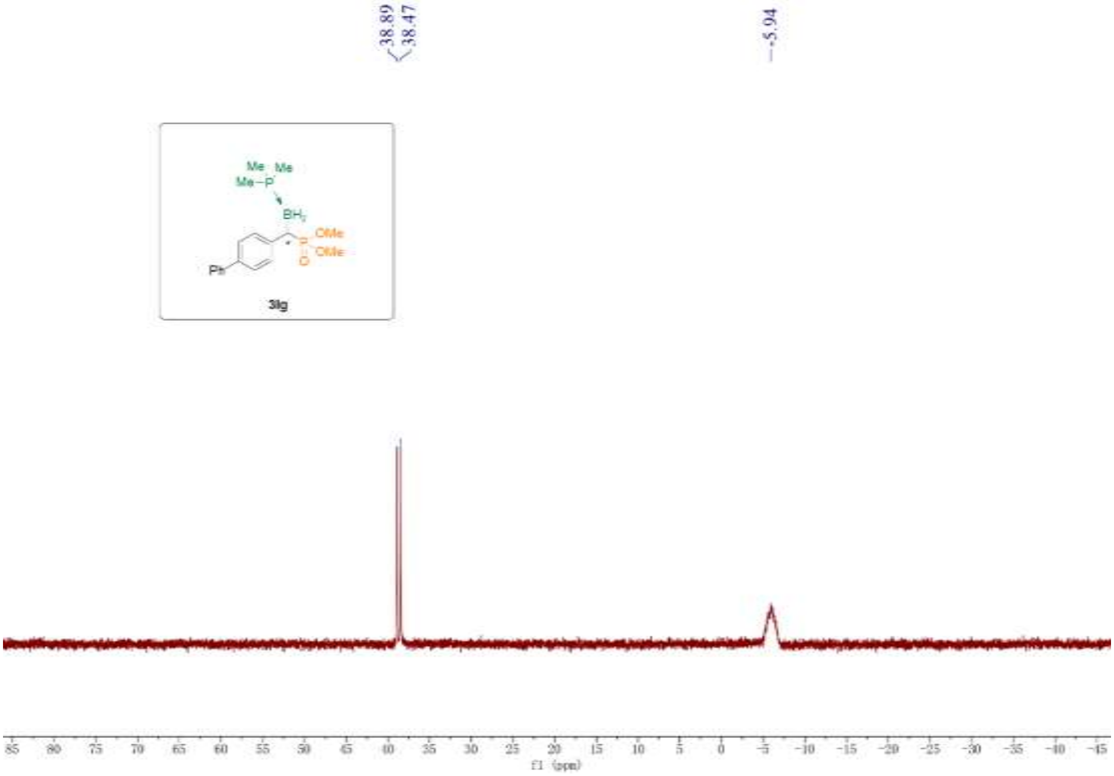

(S)-dimethyl(((tributylphosphane)boryl)([1,1'-biphenyl]-4-yl)methyl)phosphonate(3lh)

^{1}H NMR (500 MHz, Chloroform-*d*)

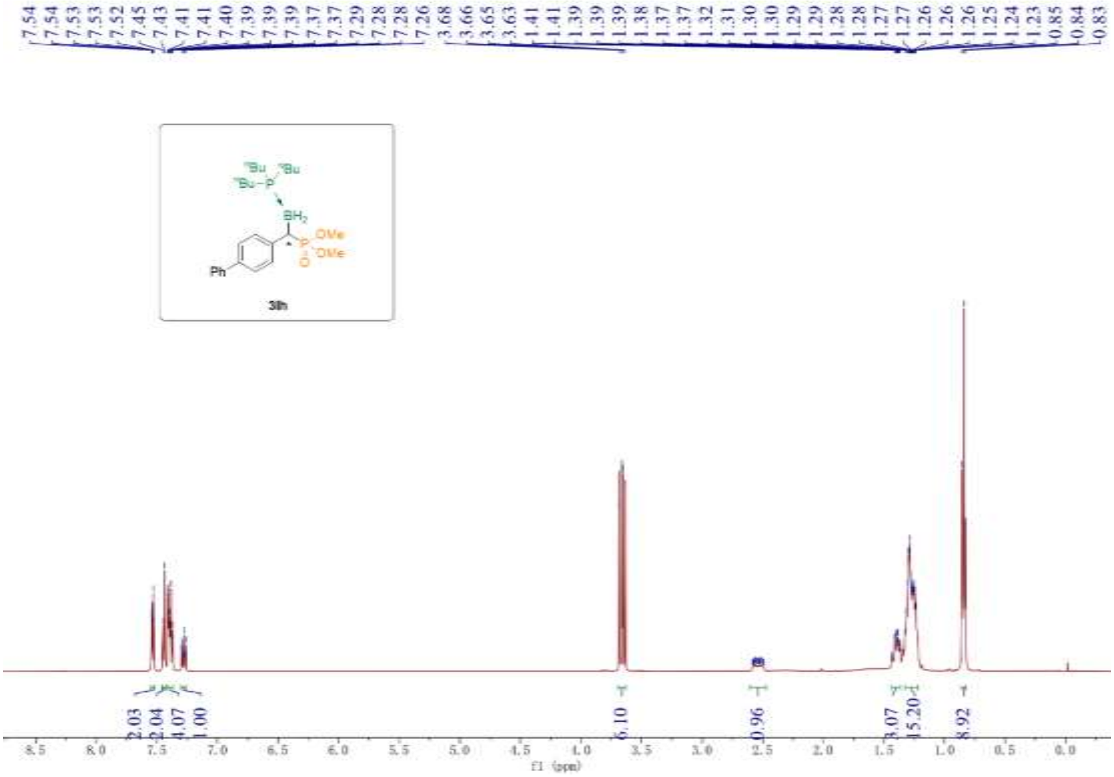

^{13}C NMR (126 MHz, Chloroform-*d*)

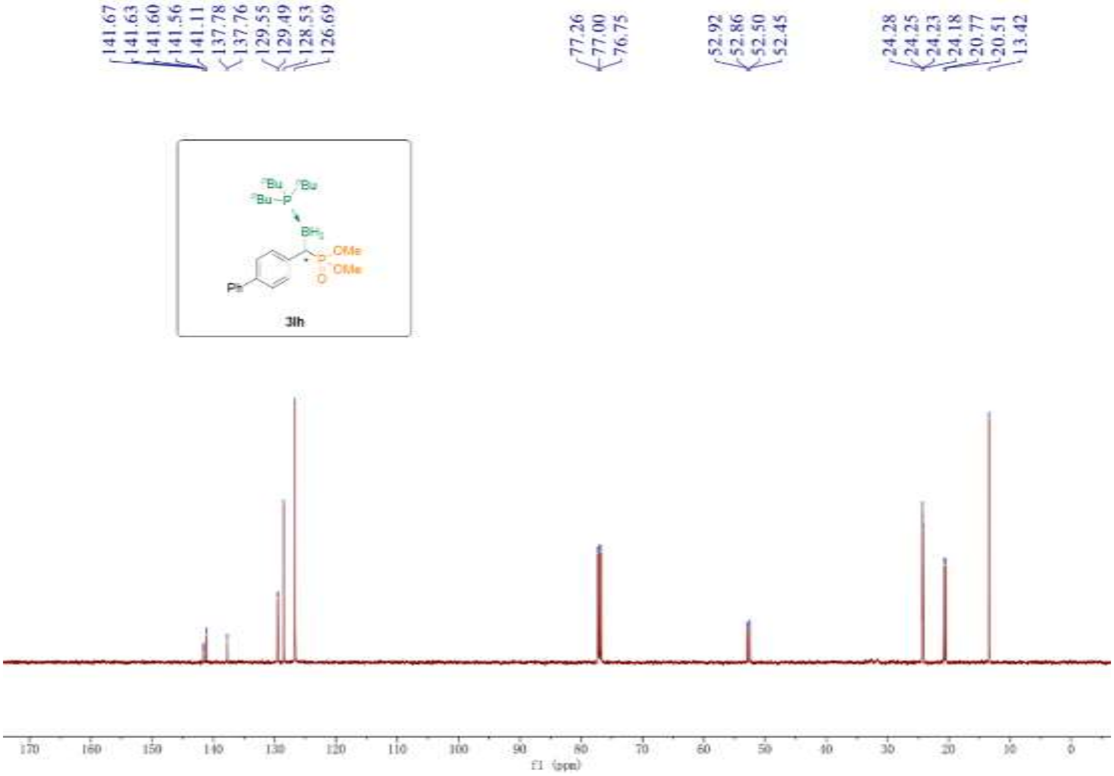

^{11}B NMR (128 MHz, Chloroform-*d*)

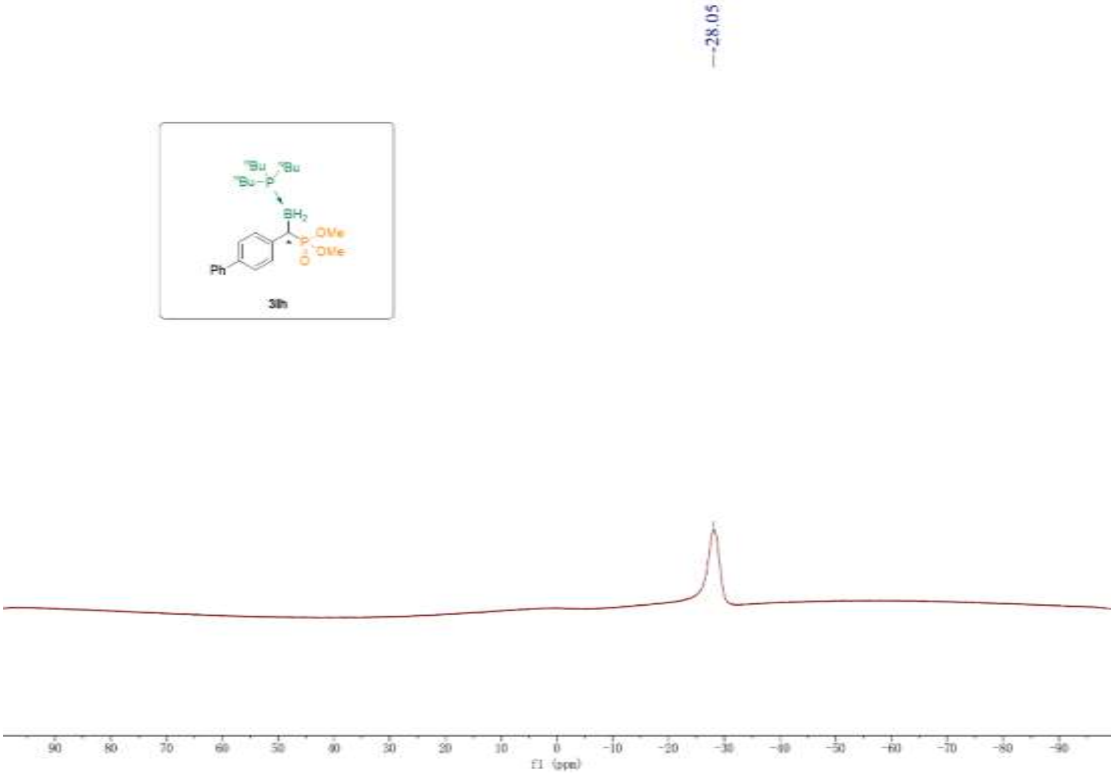

^{31}P NMR (202 MHz, Chloroform-*d*)

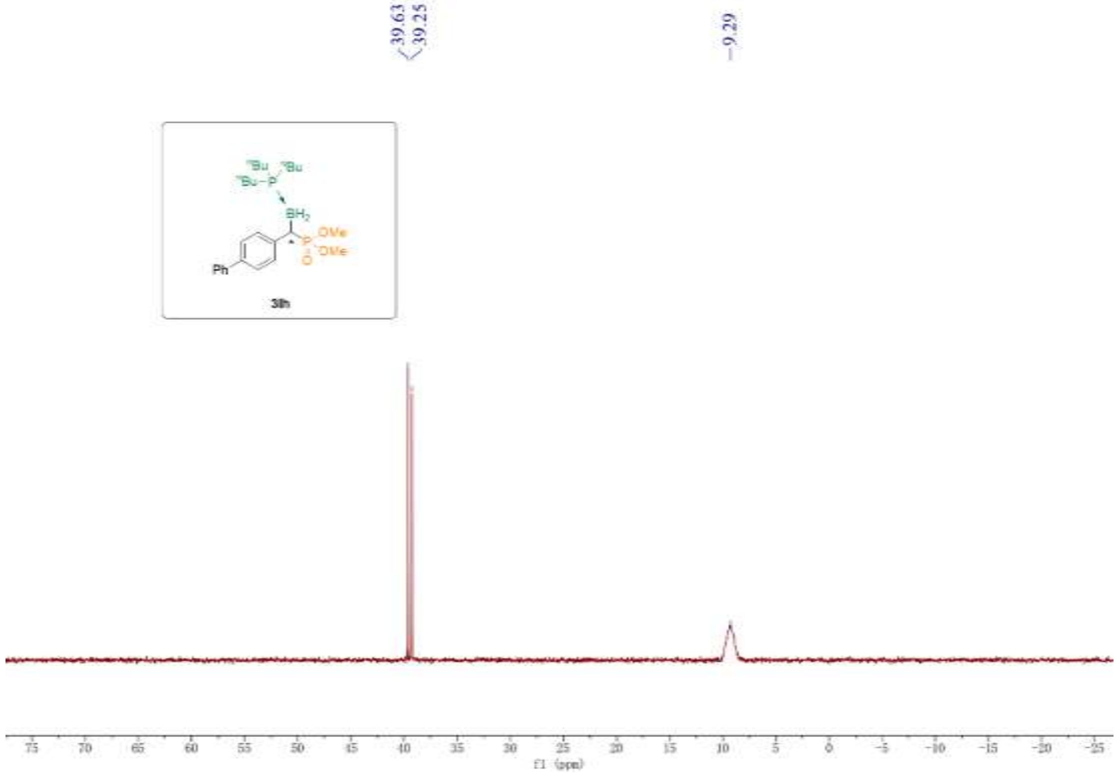

(S)-dimethyl(((trimethylphosphite)boryl)([1,1'-biphenyl]-4-yl)methyl)phosphonate(3li)

^{1}H NMR (500 MHz, Chloroform-*d*)

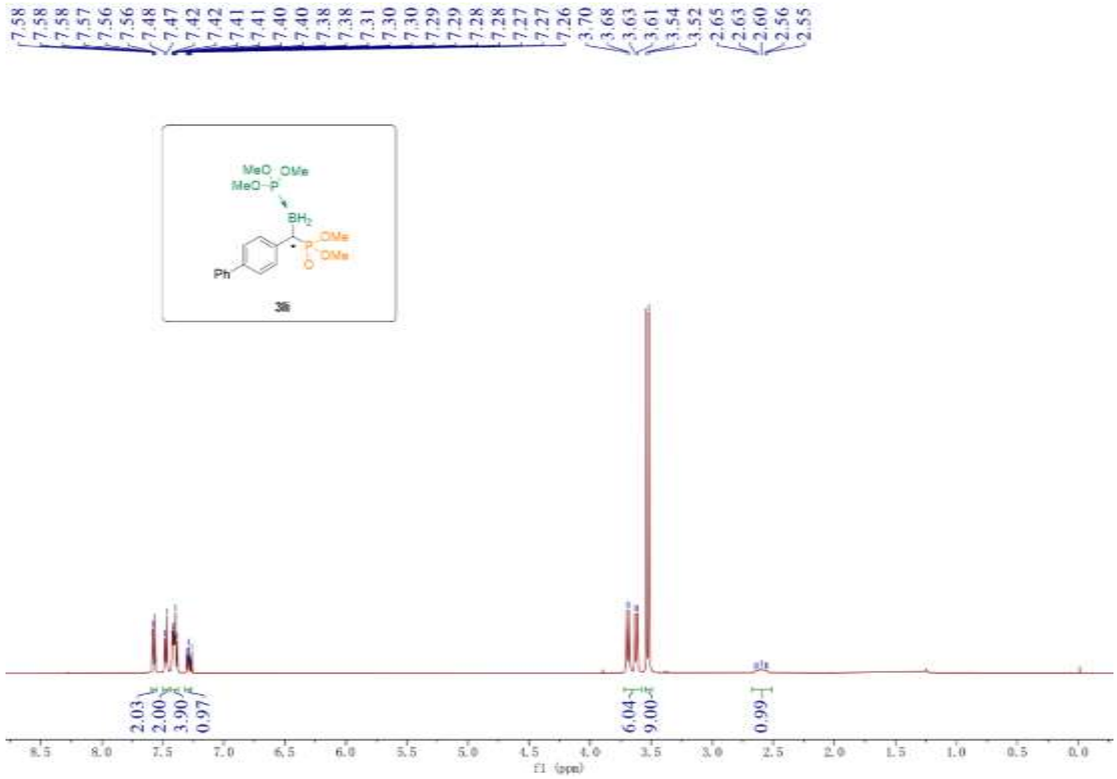

^{13}C NMR (101 MHz, Chloroform-*d*)

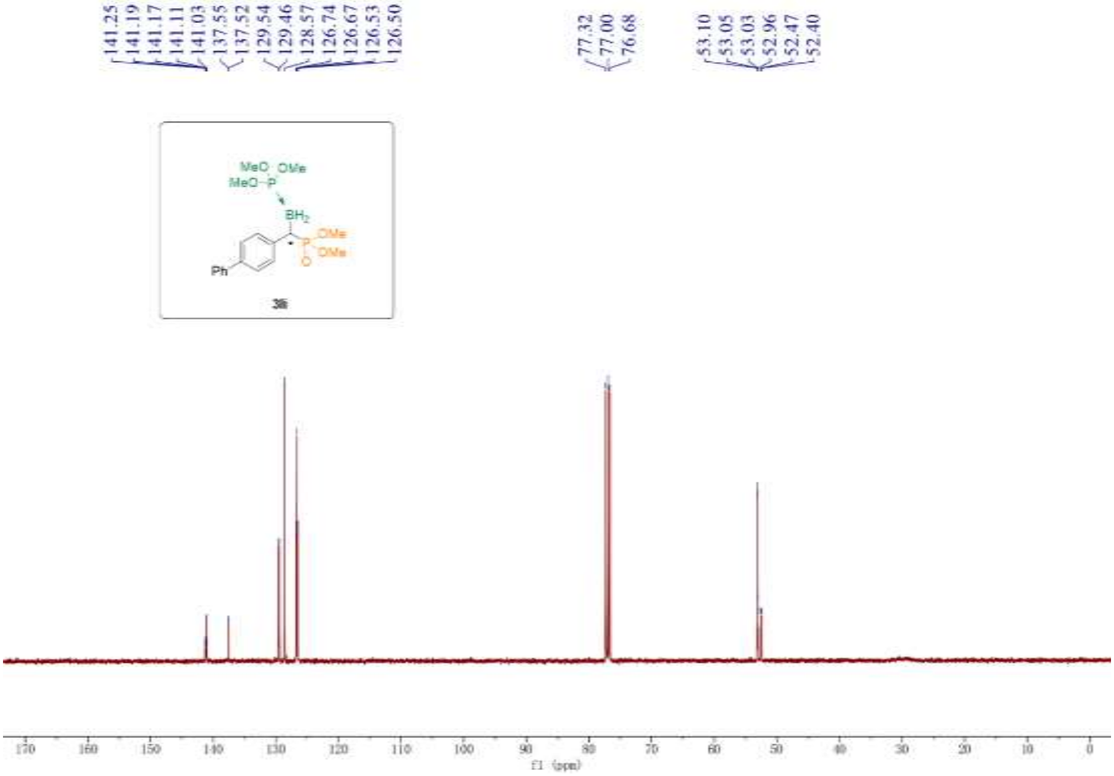

^{11}B NMR (160 MHz, Chloroform-*d*)

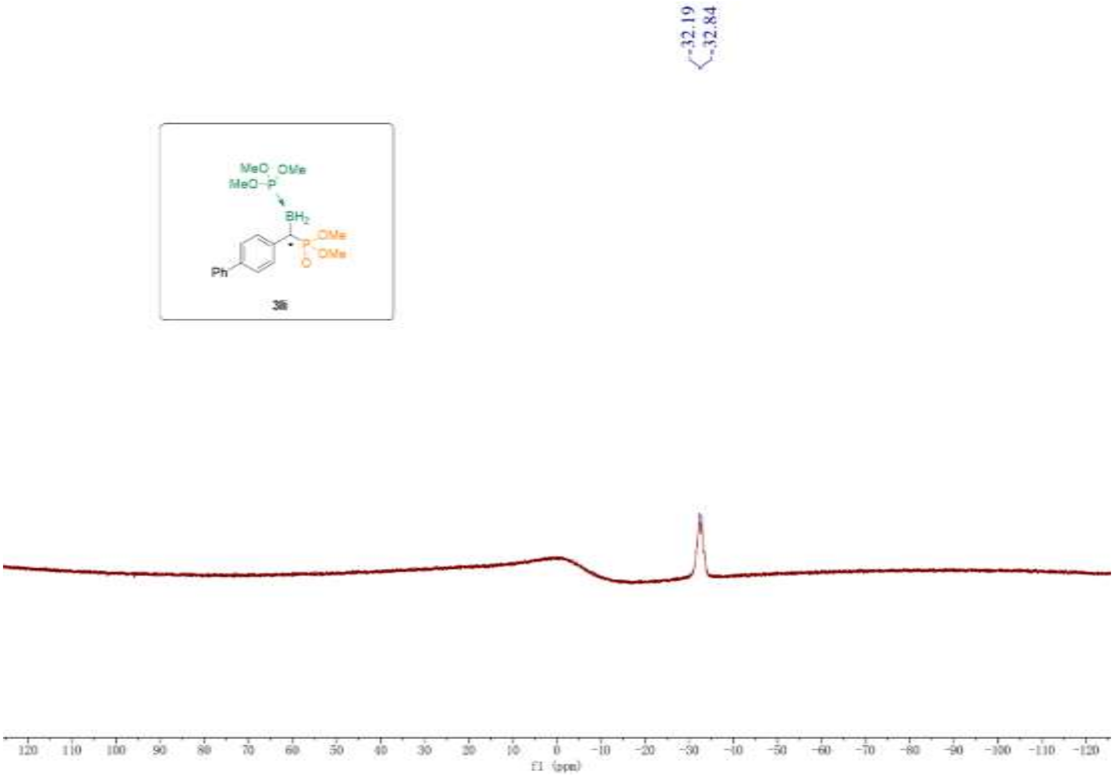

^{31}P NMR (202 MHz, Chloroform-*d*)

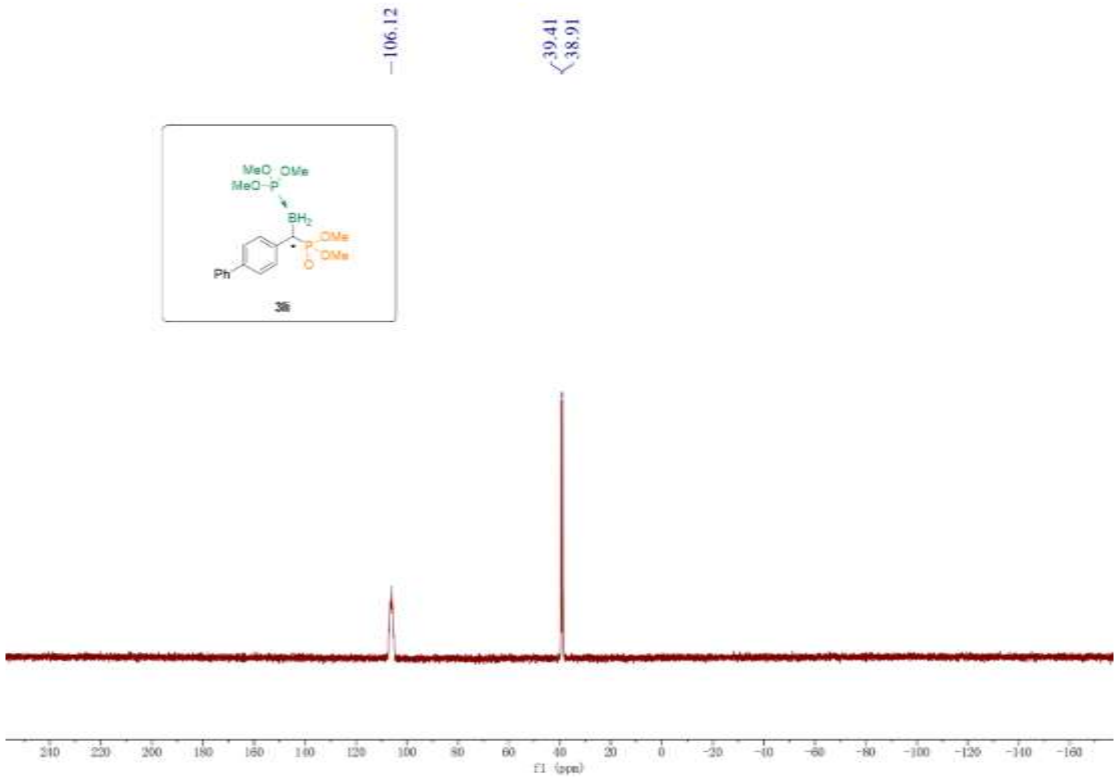

(S)-diethyl(((methyldiphenylphosphane)boryl)(phenyl)methyl)phosphonate(5aa)

^{1}H NMR (500 MHz, Chloroform-*d*)

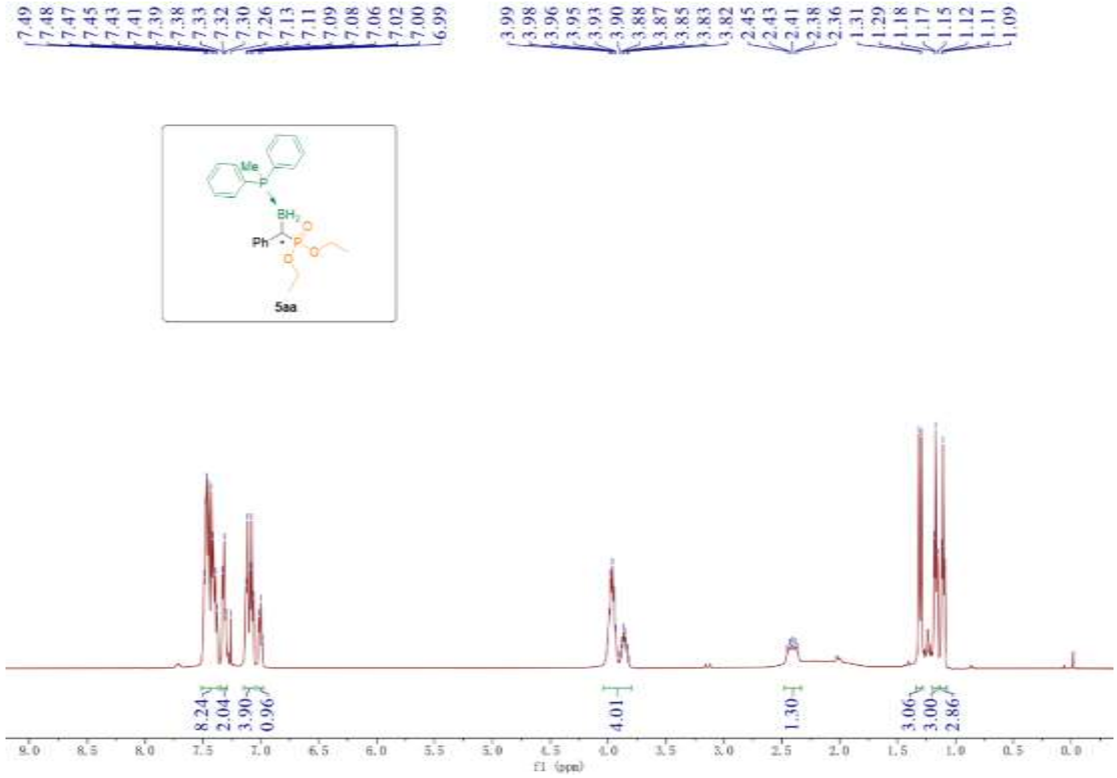

^{13}C NMR (101 MHz, Chloroform-*d*)

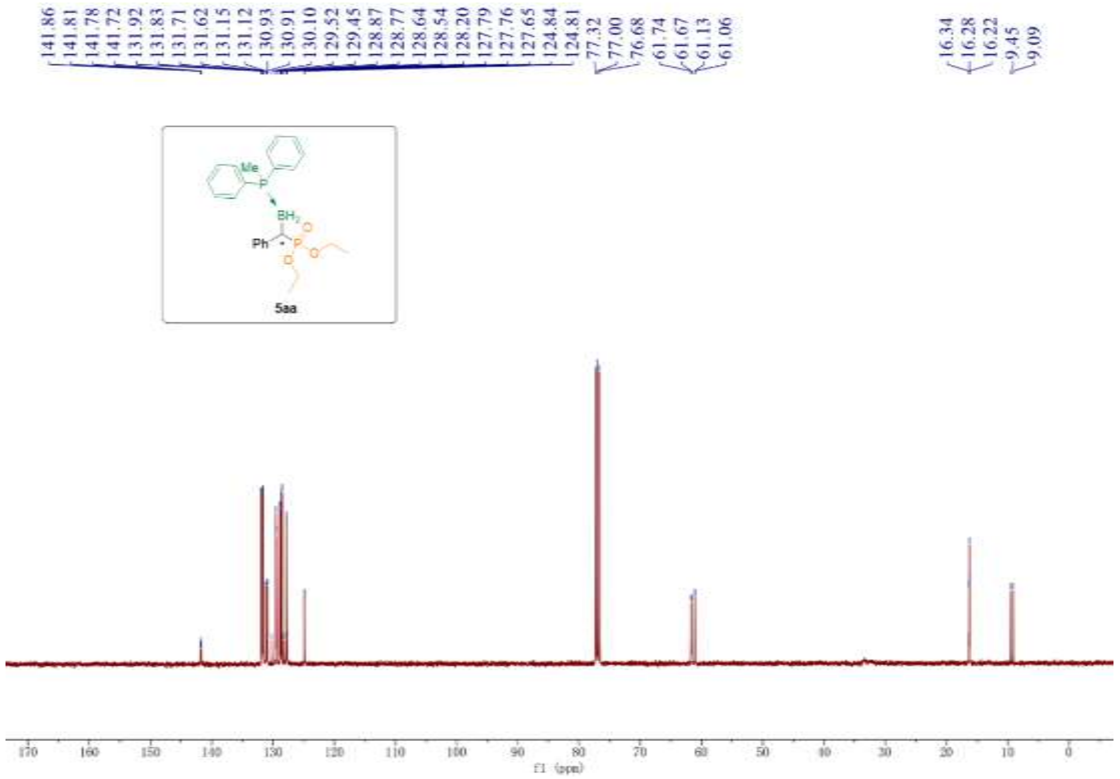

^{11}B NMR (160 MHz, Chloroform-*d*)

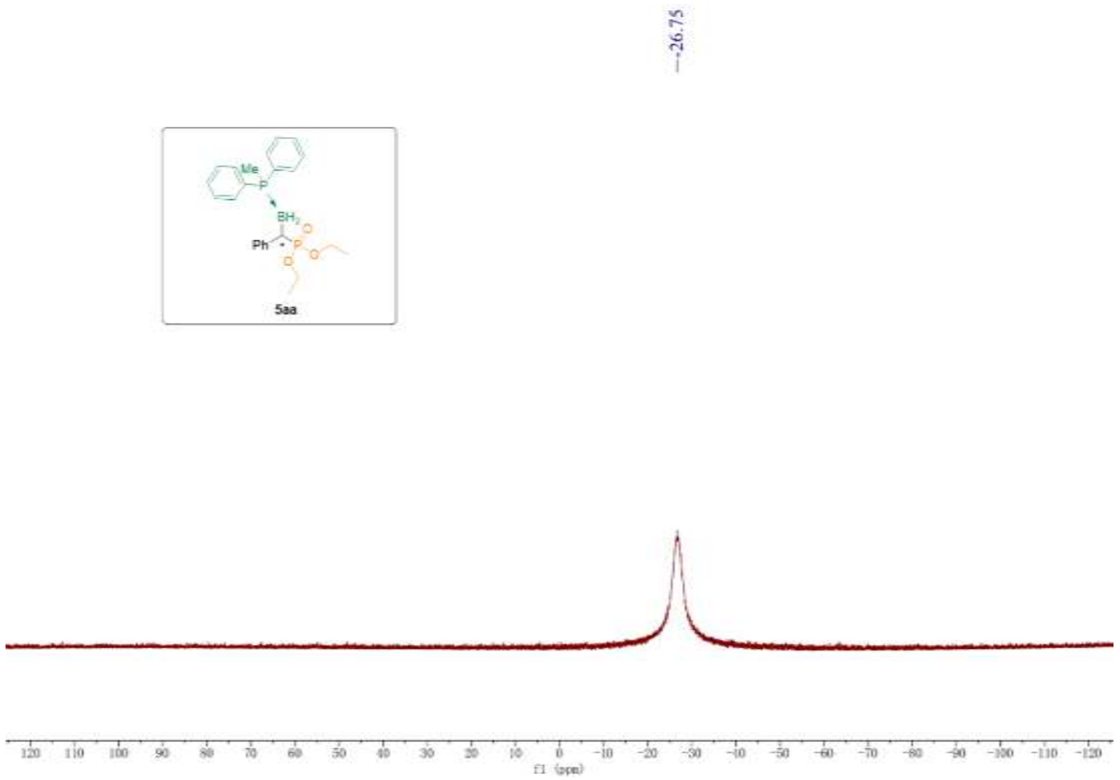

^{31}P NMR (202 MHz, Chloroform-*d*)

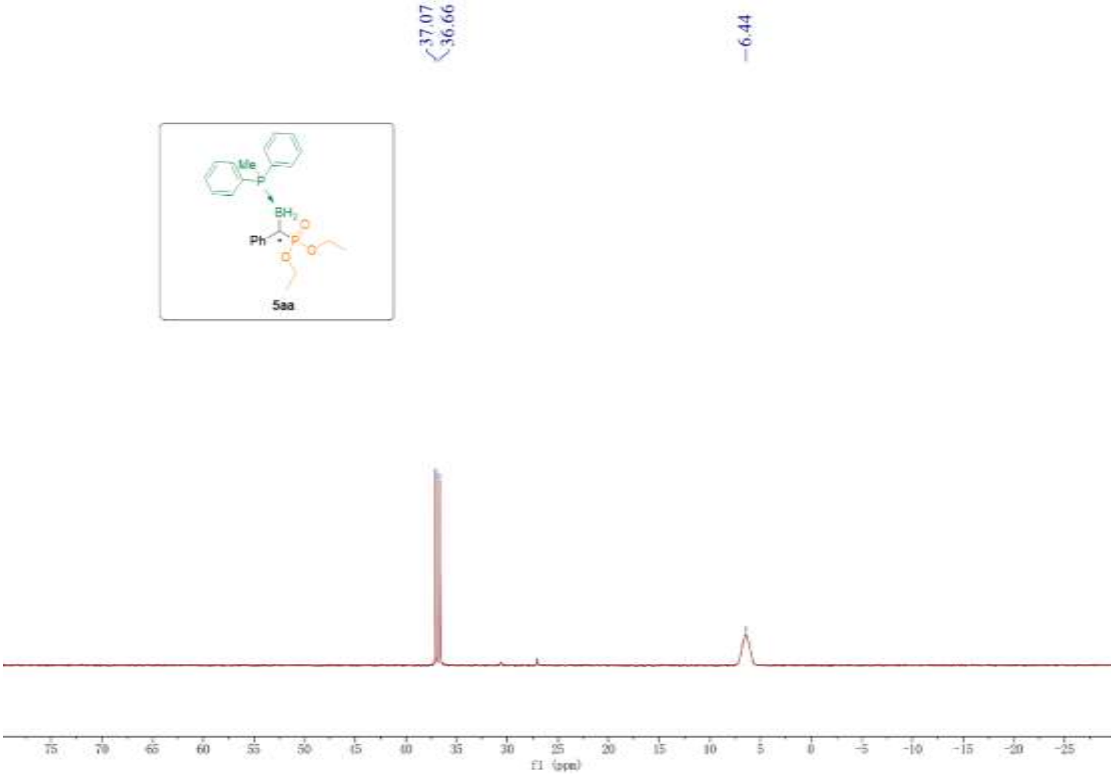

(S)-diisopropyl(((methyldiphenylphosphane)boryl)(phenyl)methyl)phosphonate(5ba)

^{1}H NMR (400 MHz, Chloroform-*d*)

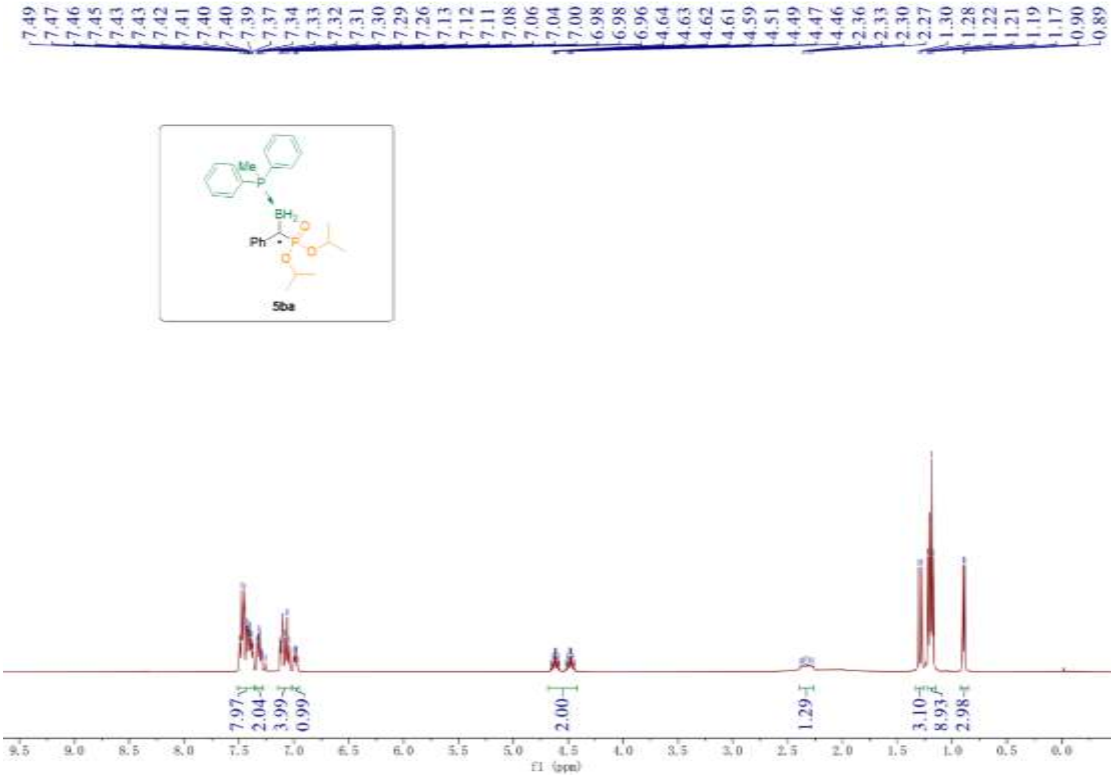

^{13}C NMR (101 MHz, Chloroform-*d*)

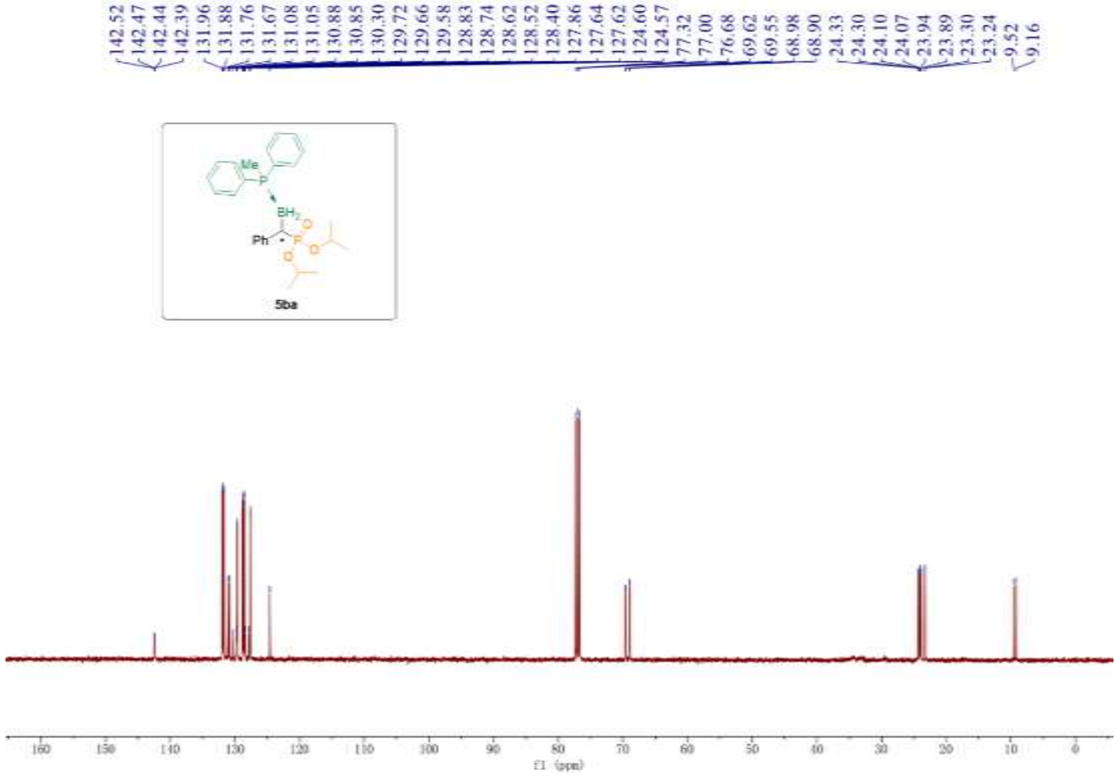

^{11}B NMR (128 MHz, Chloroform-*d*)

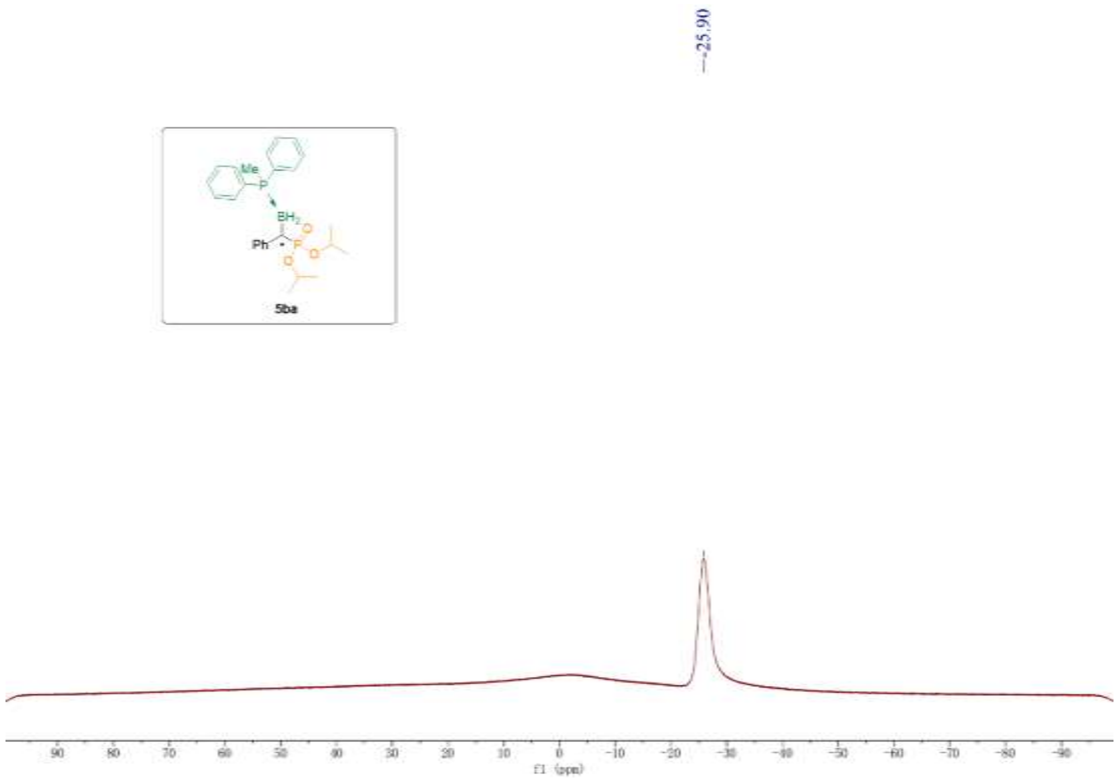

^{31}P NMR (162 MHz, Chloroform-*d*)

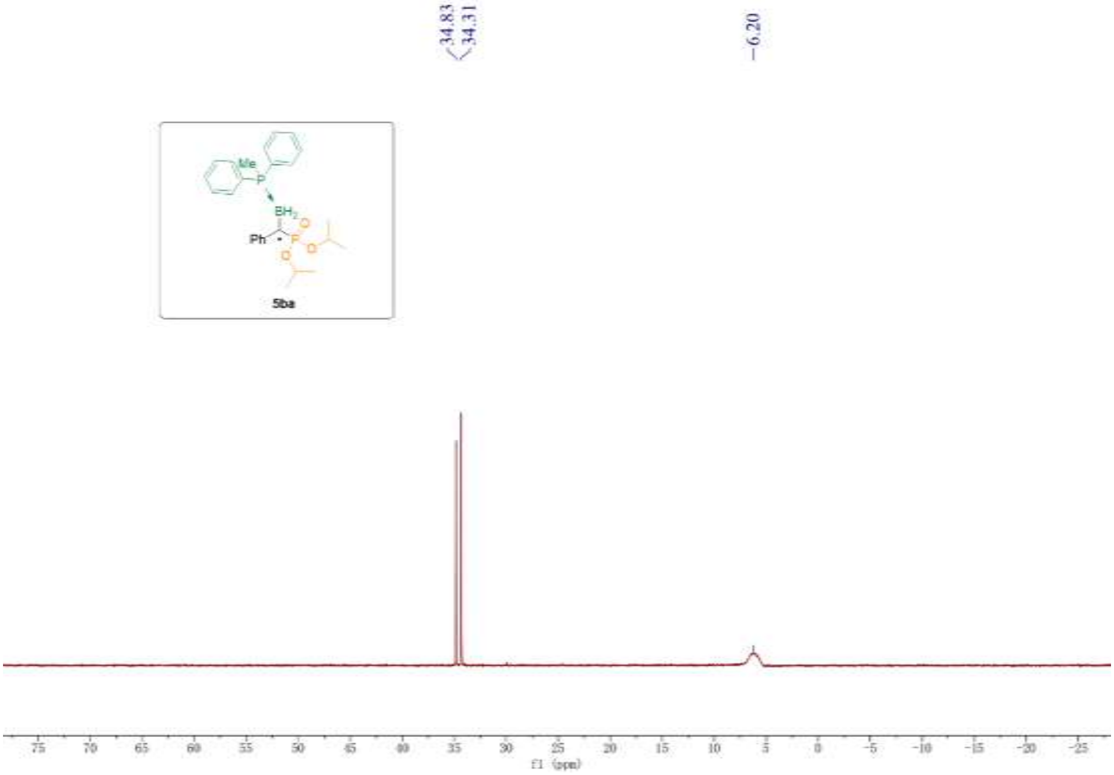

(S)-dihexyl(((methyldiphenylphosphane)boryl)(phenyl)methyl)phosphonate(5ca)

^{1}H NMR (400 MHz, Chloroform-*d*)

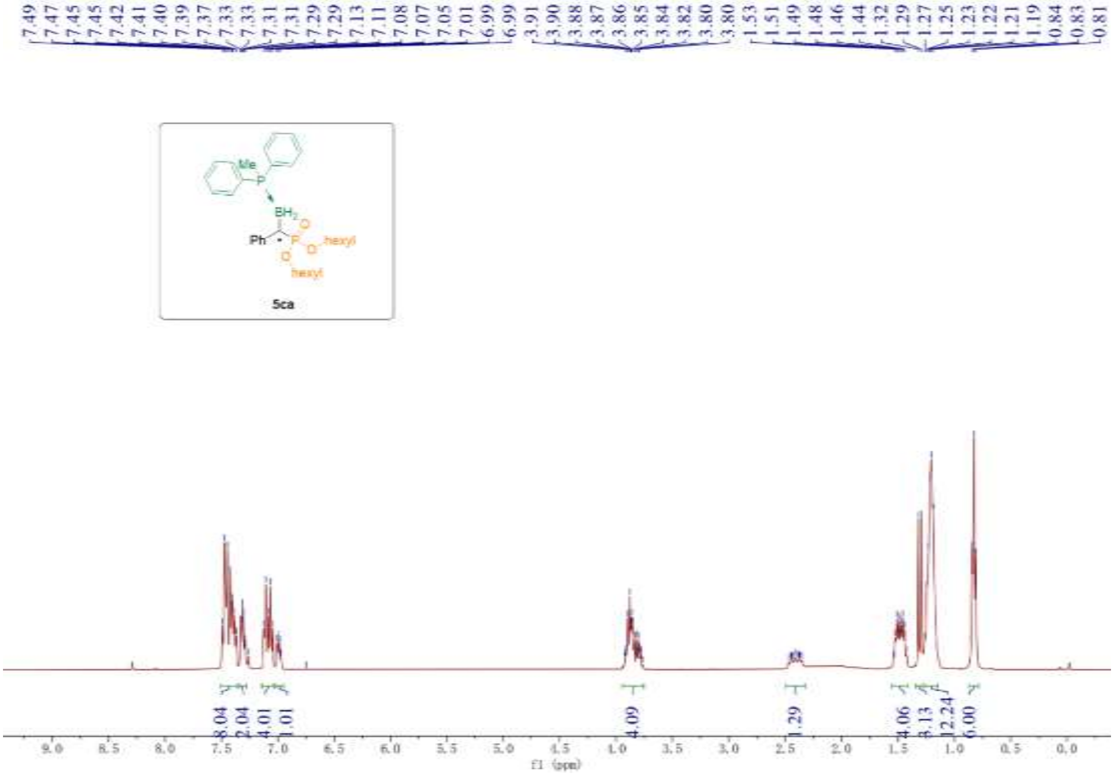

^{13}C NMR (101 MHz, Chloroform-*d*)

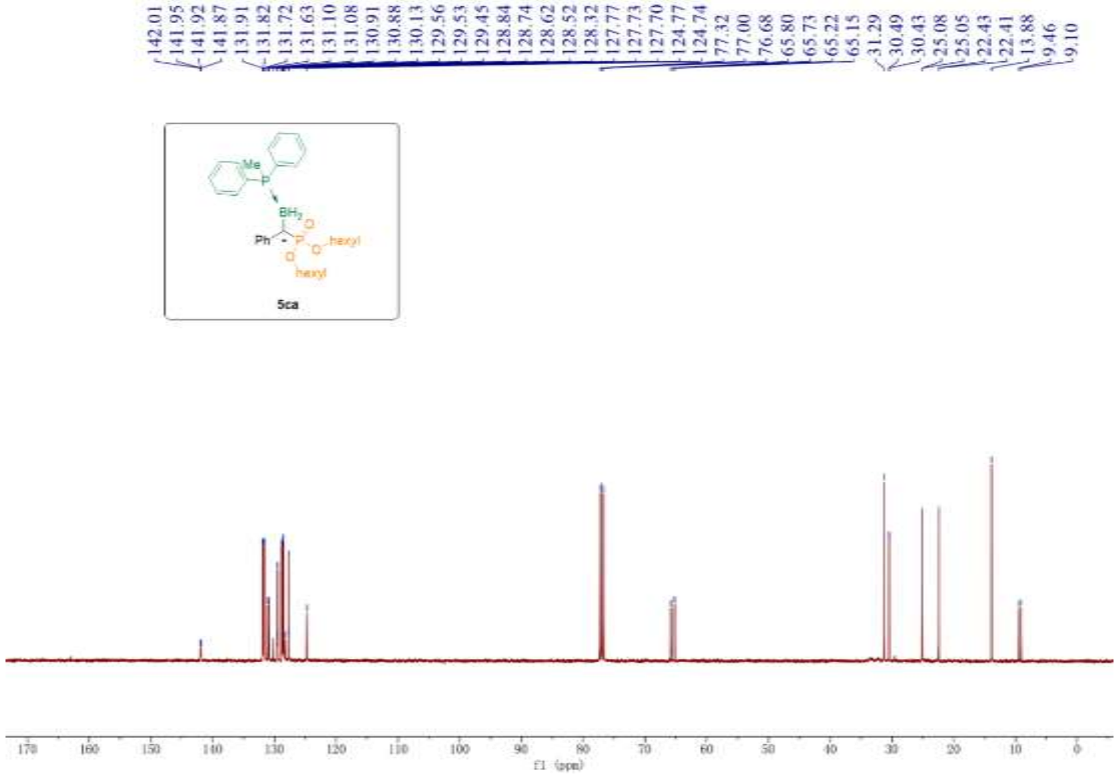

^{11}B NMR (128 MHz, Chloroform-*d*)

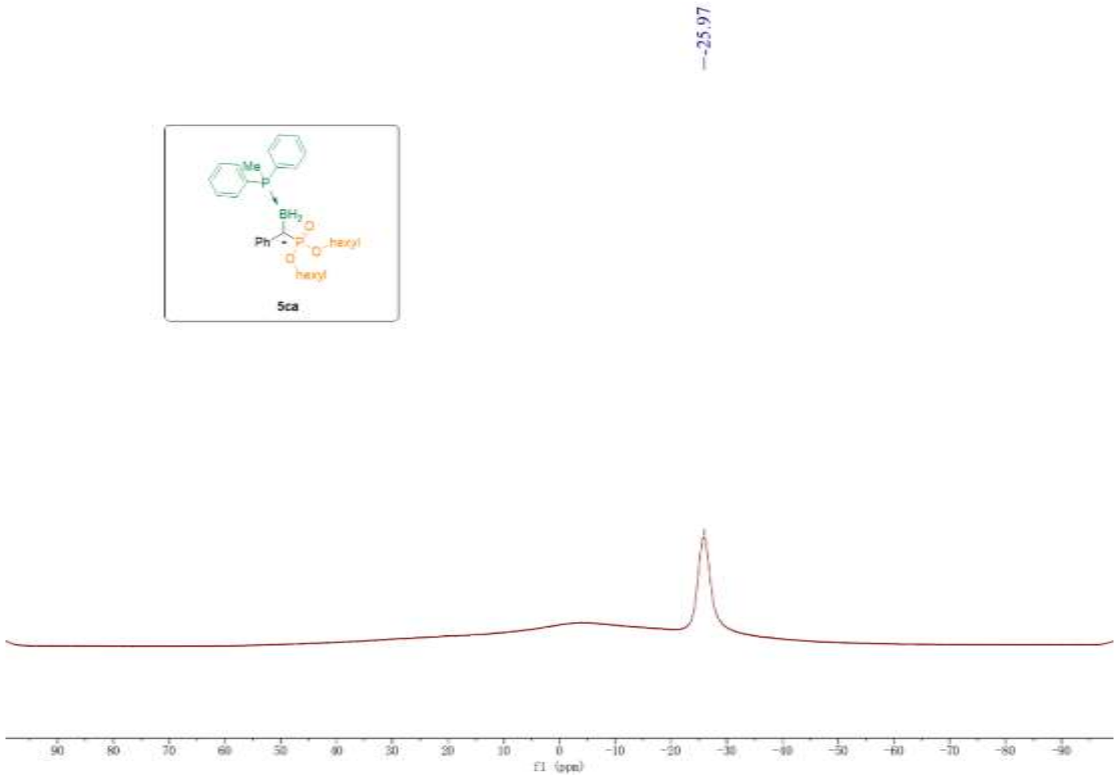

^{31}P NMR (162 MHz, Chloroform-*d*)

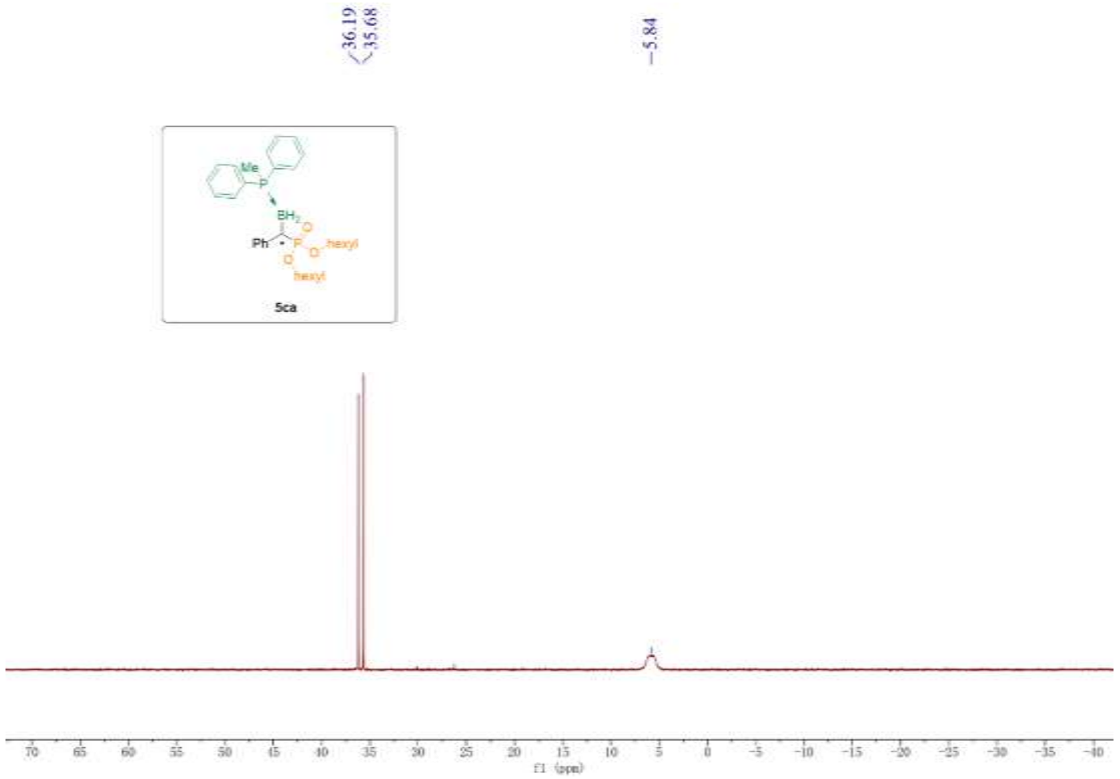

(S)- ((methyldiphenylphosphane-boryl)(4-bromophenyl)methyl) diphenylphosphine oxide (6a)

^{1}H NMR (500 MHz, Chloroform-*d*)

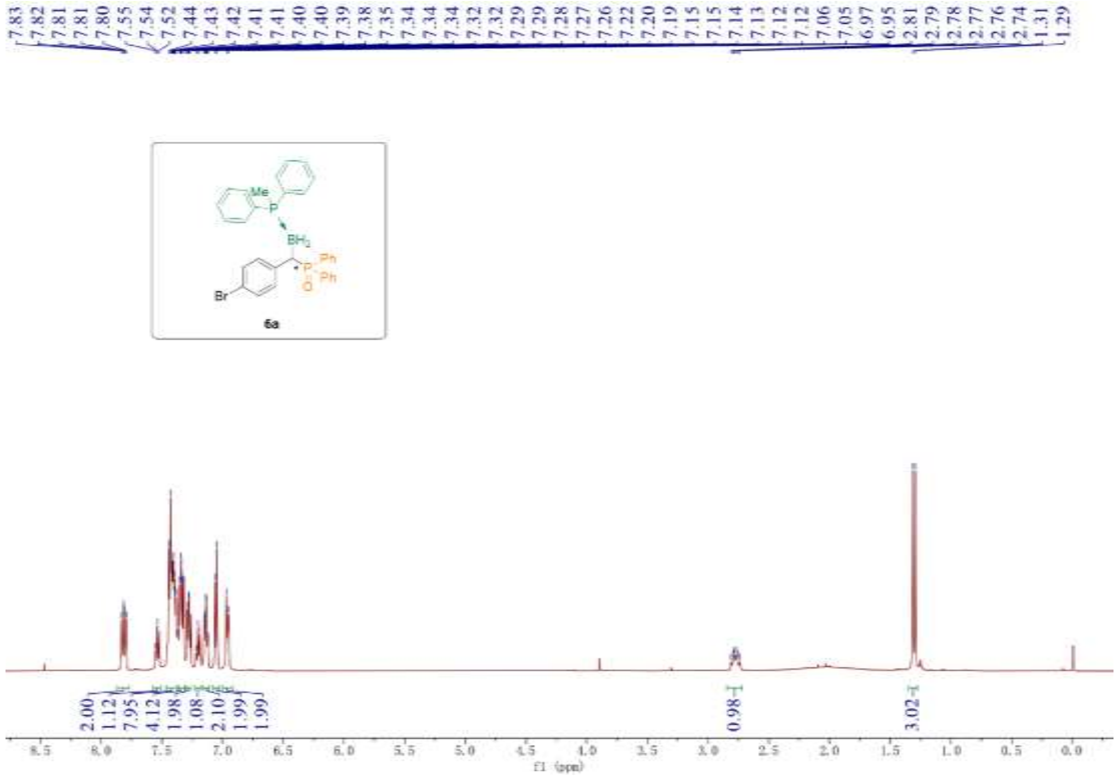

^{13}C NMR (126 MHz, Chloroform-*d*)

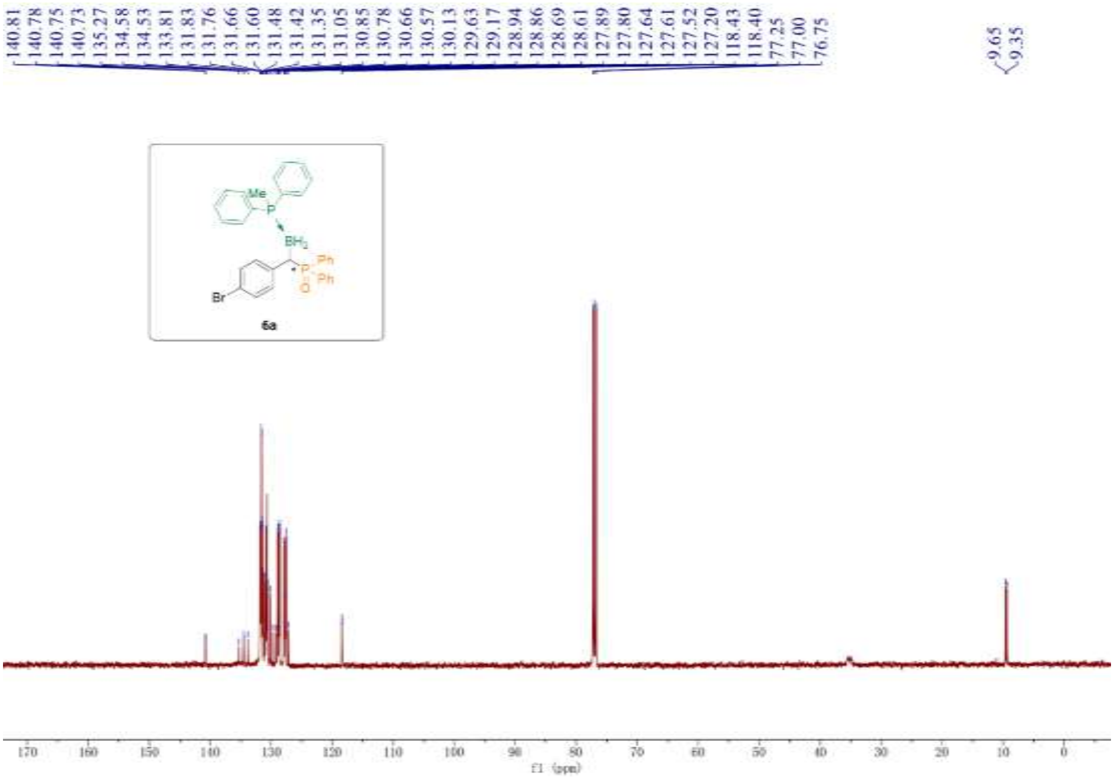

^{11}B NMR (160 MHz, Chloroform-*d*)

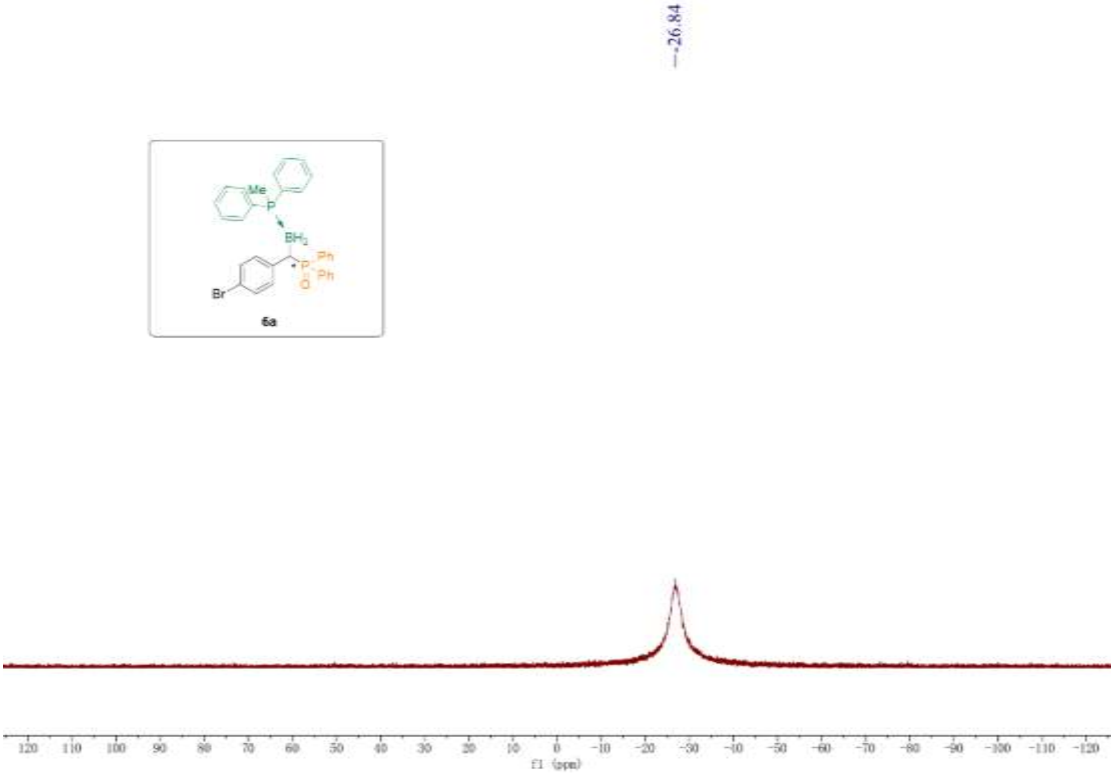

^{31}P NMR (162 MHz, Chloroform-*d*)

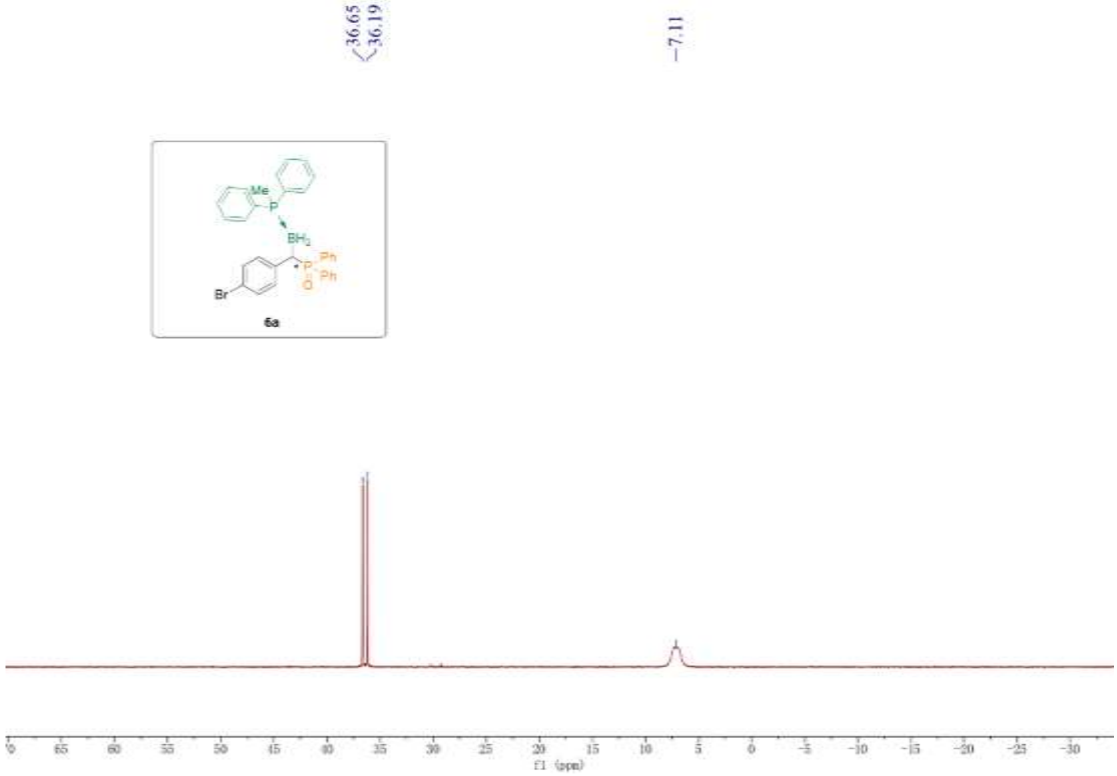

(S)- ((methyldiphenylphosphane-boryl)(4-bromophenyl)methyl) diphenylphosphane-borane (6b)

^{1}H NMR (400 MHz, Chloroform-*d*)

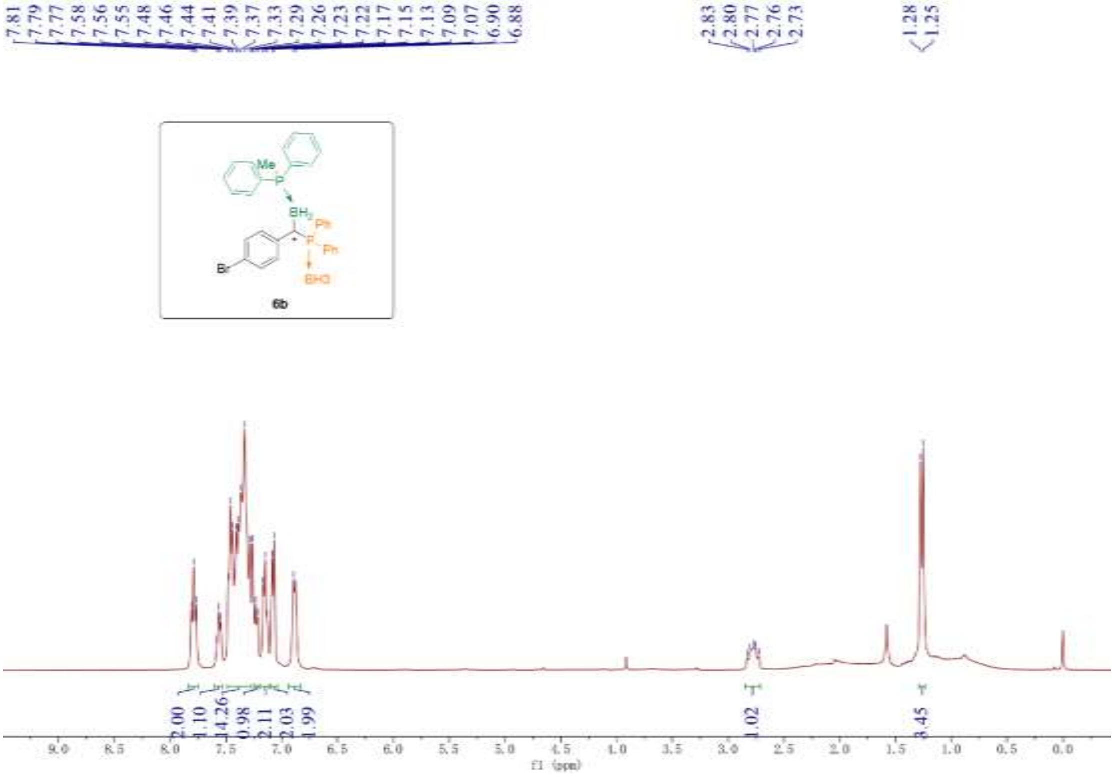

^{13}C NMR (101 MHz, Chloroform-*d*)

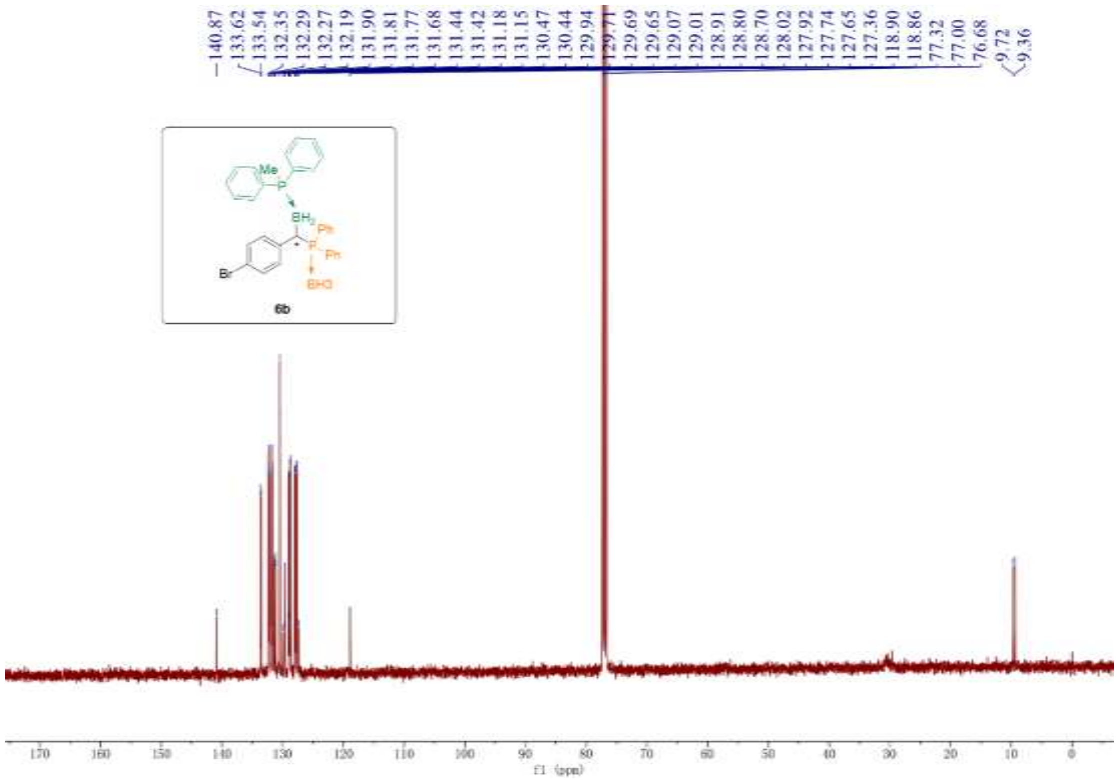

^{11}B NMR (160 MHz, Chloroform-*d*)

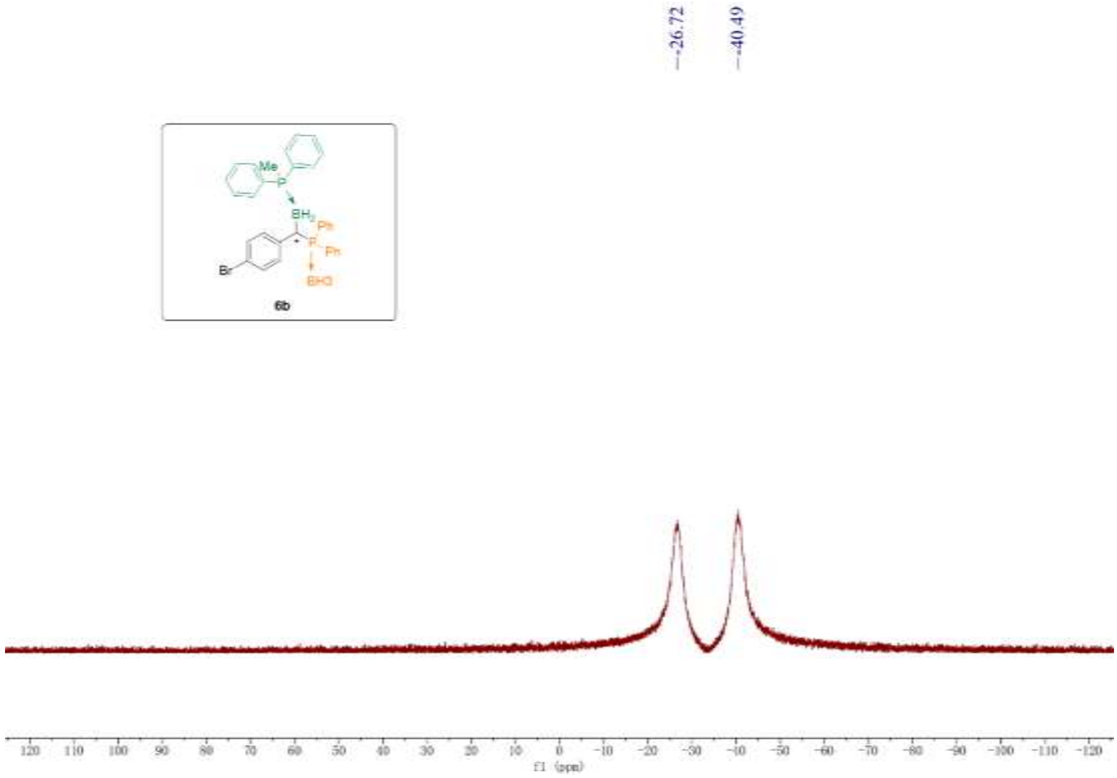

^{31}P NMR (202 MHz, Chloroform-*d*)

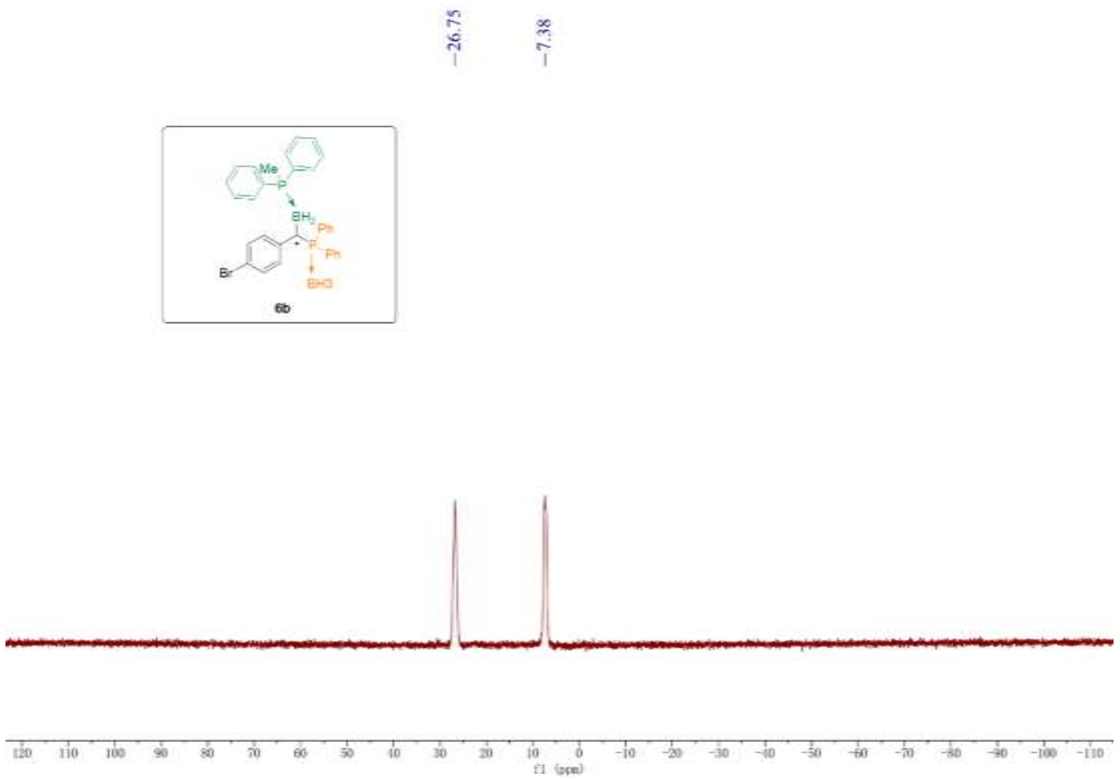

(S)-Dimethyl ([1,1'-biphenyl]-4-yl(hydroxy)methyl)phosphonate(6c)

^{1}H NMR (400 MHz, Chloroform-*d*)

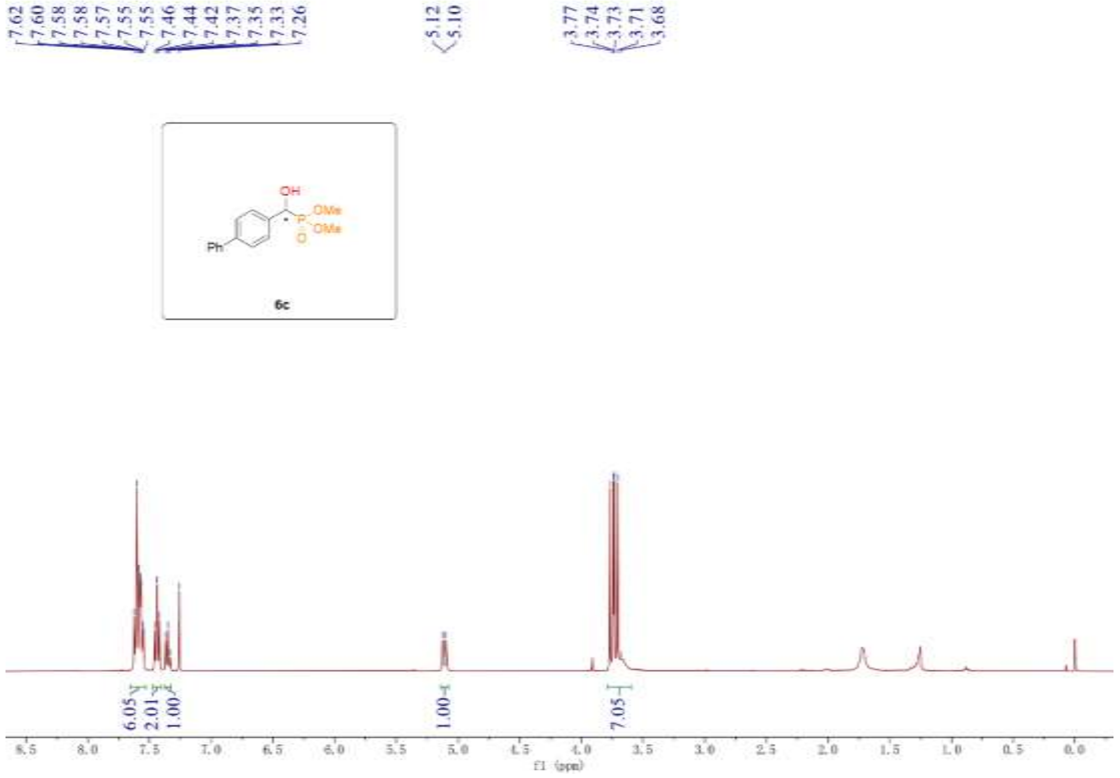

^{13}C NMR (101 MHz, Chloroform-*d*)

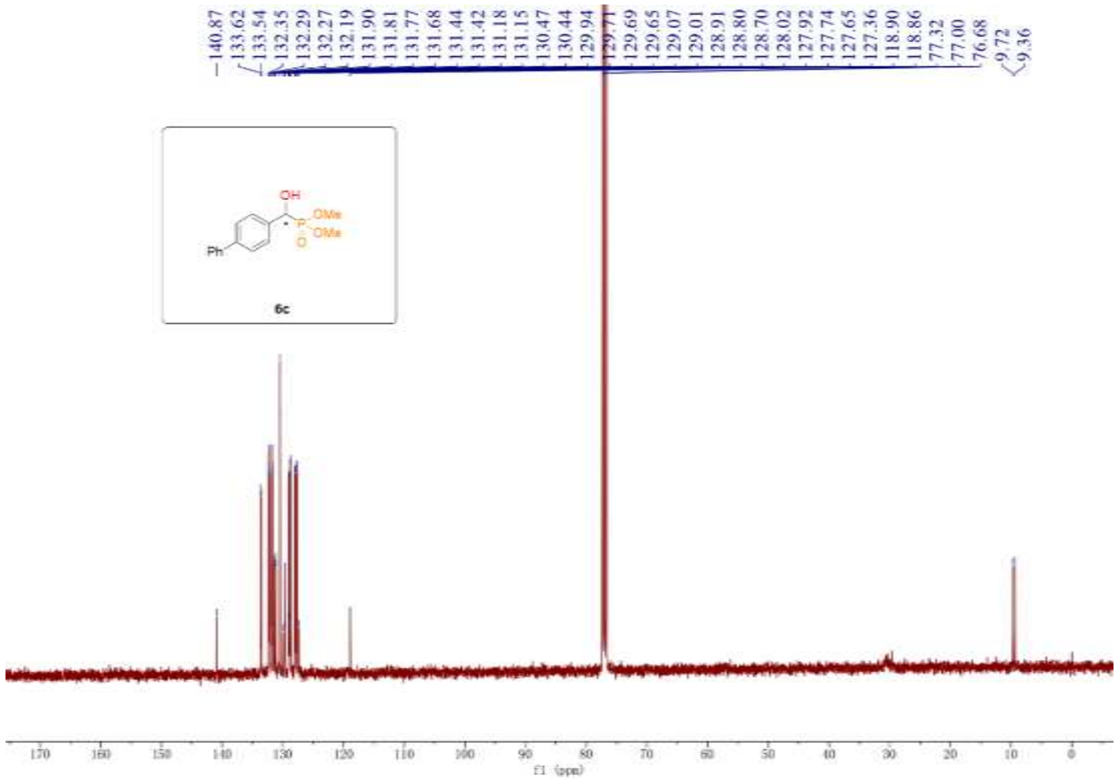

^{31}P NMR (162 MHz, Chloroform-*d*)

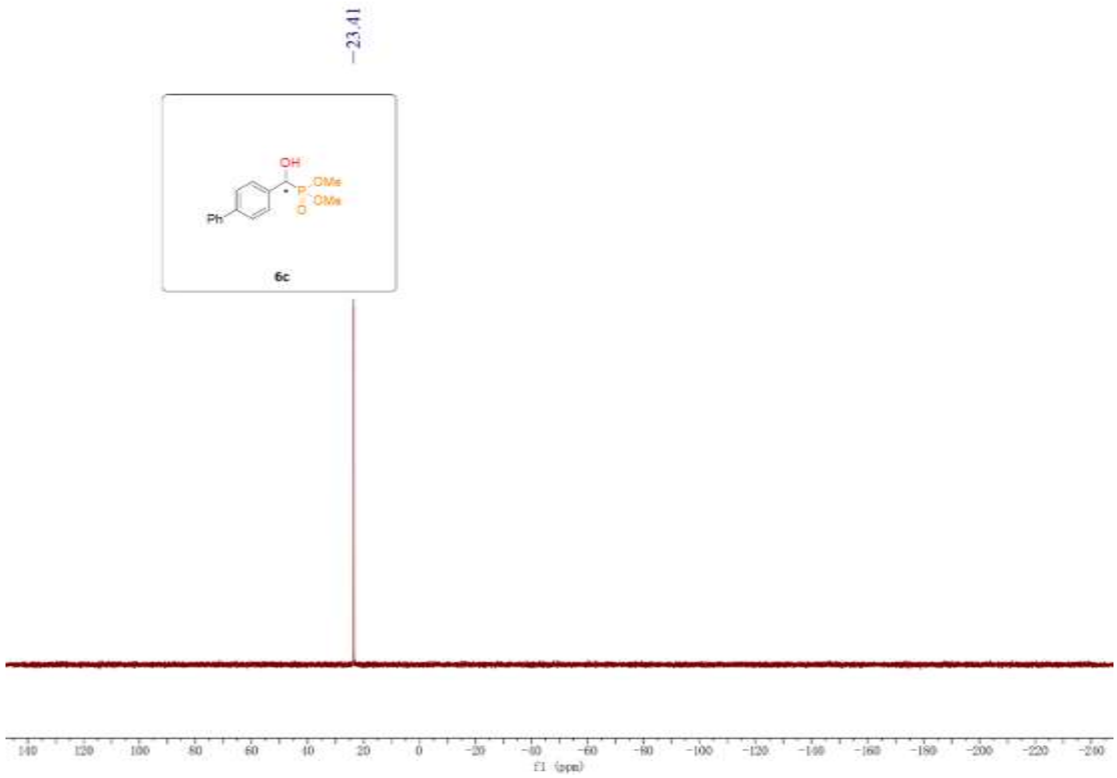

Dimethyl ([1,1'-biphenyl]-4-ylmethyl-d)phosphonate(6d)

^{1}H NMR (400 MHz, Chloroform-*d*)

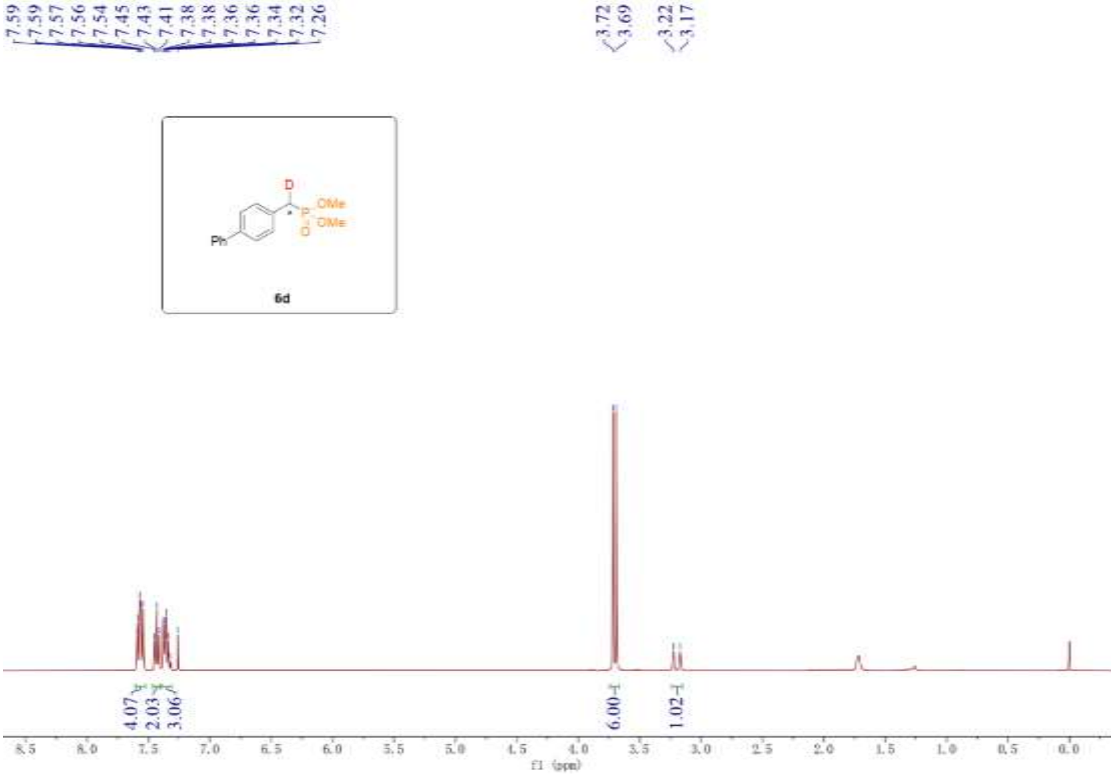

^{13}C NMR (101 MHz, Chloroform-*d*)

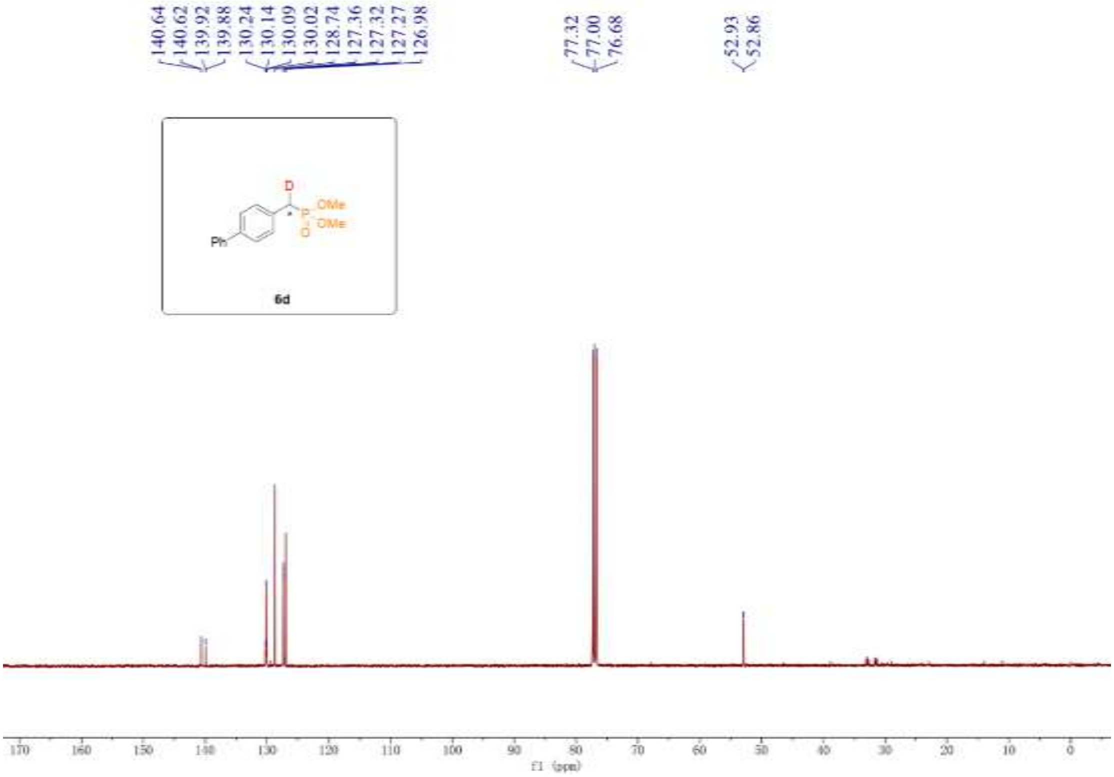

^{31}P NMR (162 MHz, Chloroform-*d*)

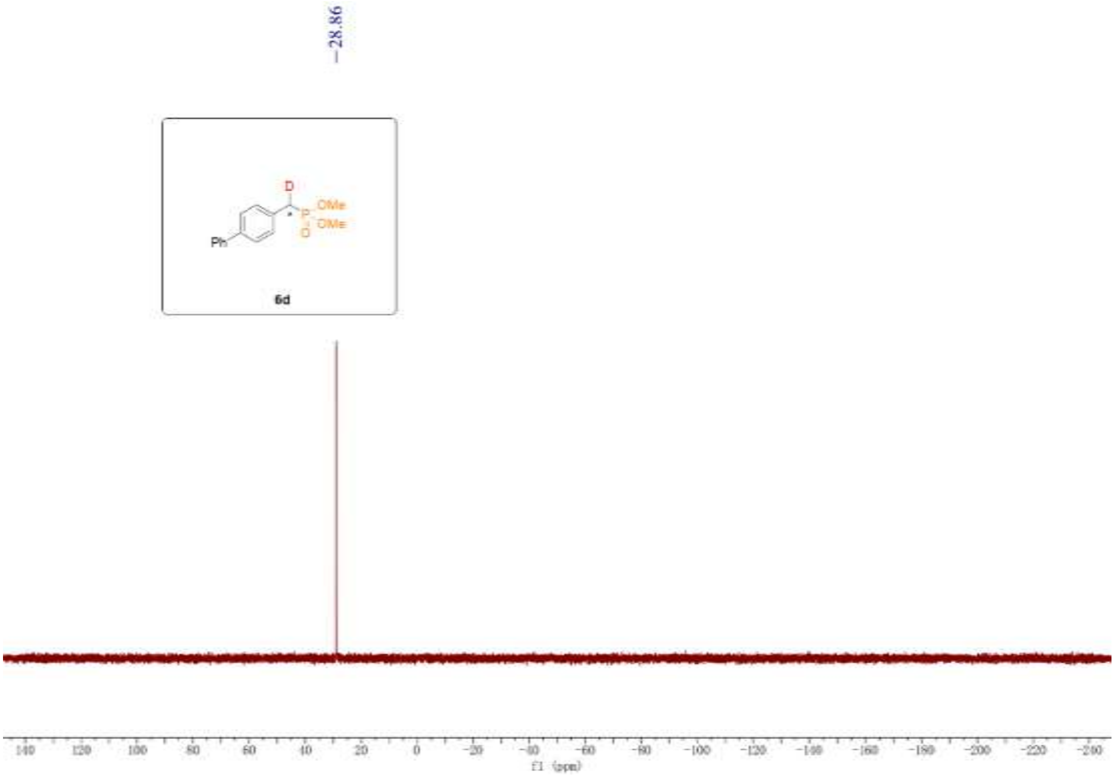

Supplement: SC-015-D4SC01271B-s001 [file SC-015-D4SC01271B-s001.pdf]
